# Photoinduced Remote Functionalization of Amides and Amines Using Electrophilic Nitrogen Radicals

**DOI:** 10.1002/anie.201807941

**Published:** 2018-08-29

**Authors:** Sara P. Morcillo, Elizabeth M. Dauncey, Ji Hye Kim, James J. Douglas, Nadeem S. Sheikh, Daniele Leonori

**Affiliations:** ^1^ School of Chemistry University of Manchester Oxford Road Manchester M13 9PL UK; ^2^ Early Chemical Development Pharmaceutical Sciences IMED Biotech Unit AstraZeneca Macclesfield SK10 2NA UK; ^3^ Department of Chemistry Faculty of Science King Faisal University P.O. Box 380 Al-Ahsa 31982 Saudi Arabia

**Keywords:** amines, halogenation, photochemistry, radicals, reaction mechanisms

## Abstract

The selective functionalization of C(sp^3^)−H bonds at distal positions to functional groups is a challenging task in synthetic chemistry. Reported here is a photoinduced radical cascade strategy for the divergent functionalization of amides and protected amines. The process is based on the oxidative generation of electrophilic amidyl radicals and their subsequent transposition by 1,5‐H‐atom transfer, resulting in remote fluorination, chlorination and, for the first time, thioetherification, cyanation, and alkynylation. The process is tolerant of most common functional groups and delivers useful building blocks that can be further elaborated. The utility of this strategy is demonstrated through the late‐stage functionalization of amino acids and a dipeptide.

The selective functionalization of C(sp^3^)−H bonds is a long sought‐after goal in synthetic chemistry.^1^ In general, when a directing group is present, products of α‐ and β‐functionalization are easy to access through ionic (e.g. enolate chemistry, 1,4‐addition) and transition metal mediated reactivity (e.g. directed metallation; Scheme 1 A). Targeting more distal positions, such as the γ‐ and δ‐positions, is more difficult.^2^ One way of achieving this is to use nitrogen radicals, harnessing their ability to undergo transposition processes by 1,5‐H atom transfer (1,5‐HAT).^3^ This reactivity constitutes a key step of the named reaction developed by Hoffman and Löffler,^4^ and has been pioneered in classical radical processes by the groups of Forrester,^5^ Minisci,^6^ Suarez,^7^ and others.^8^ More recently, the advent of visible‐light photocatalysis^9^ has propelled the development of milder ways for exploring this reactivity. For example, the group of Muñiz^10^ has identified an array of protocols for the preparation of nitrogen heterocycles using catalytically generated N−I and N−Br amides. The group of Nagib^11^ has used N−I imine‐type derivatives to access 1,2‐amino‐alcohols and the group of Nevado^12^ has employed electron‐poor O‐acyl oximes in the assembly of tetralones. Most relevant here is the work of the groups of Knowles^13^ and Rovis,^14^ who through proton‐coupled electron transfer have generated amidyl radicals from amides and developed cascades based on 1,5‐HAT and subsequent radical addition to Michael acceptors.

**Scheme 1:**
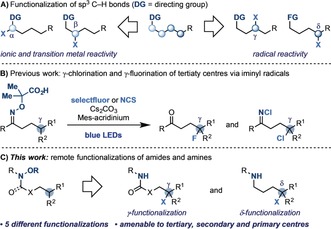
A) Ionic versus radical reactivity for the functionalization of C(sp^3^)−H bonds. B) γ‐Fluorination and chlorination of tertiary centers by 1,5‐HAT of iminyl radicals. C) γ‐ and δ‐Functionalizations by 1,5‐HAT of amidyl radicals. DG=directing group, FG=functional group, NCS=*N*‐chlorosuccinimide.

We and the group of Studer have recently developed a reaction for the oxidative generation of iminyl radicals and the following 1,5‐HAT‐functionalization (Scheme 1 B).^15^ In our hands this process enabled γ‐chlorination and γ‐fluorination, but was only feasible at tertiary centers, thus limiting the synthetic applicability to a narrow range of substrates. Herein we demonstrate how changing the nature of the nitrogen radical to amidyl and *N*‐protected aminyl radicals unlocks distal functionalizations of amides, carbamates, and amines (Scheme 1 C). This methodology targets tertiary, secondary, and even primary centers and has been applied to five different functionalizations spanning distal C−X (X=heteroatom) and C−C bond formation.

At the outset of our work, we surmized that to achieve efficient 1,5‐HAT‐functionalization cascades at room temperature, both enthalpic and polar effects had to be maximized.^16^ We therefore turned our interest towards the use of amidyl radicals because of their high electrophilic character^17^ and the strong N−H bonds of the corresponding amides. These properties were expected to synergistically render the reaction exothermic (Δ*G*°<0; enthalpic contribution) and lower the activation barrier (Δ*G* ^≠^; polar effects contribution). To obtain more information on these aspects, we have conducted computational studies on a range of model 1,5‐HAT processes spanning amidyl, carbamoyl, *N*‐Ts‐aminyl, and iminyl radicals (Scheme 2 A).^18^ For example, while 1,5‐HAT of the iminyl radical **A** is endothermic, amidyls (**B**, **C**, and **E**) and the N‐Ts‐aminyl radical **D** ought to undergo exothermic abstraction of H‐atoms at tertiary, secondary, and even primary positions. Indeed, a good correlation was found between the variation of bond dissociation energies (BDEs)^19^ between the incipient N−H bond and the γ‐sp^3^ C−H bond (δBDE=BDE_N‐H_−BDE_C‐H_), and the Δ*G*° values of the processes. Correlation between the δBDE and the activation barrier for 1,5‐HAT (Δ*G* ^≠^) was less obvious but a clear trend was observed within the amidyl series (γ‐abstractions, light blue squares). The high exothermicity characterizing these reactions means that early transition states are likely operating,^20^ which makes the reaction parameters sensitive to polar effects. Indeed, a good correlation between the nitrogen‐radical electrophilicity indices (*ω* ^+^_rc_) and both Δ*G*° and Δ*G* ^≠^ was observed. These results make the evaluation of this parameter of relevance when planning similar reactions.

**Scheme 2:**
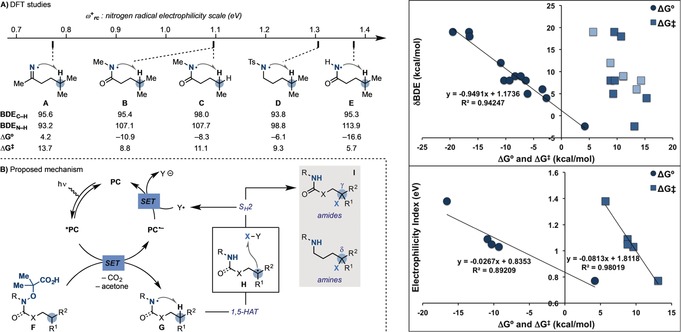
A) DFT studies on the 1,5‐HAT of nitrogen radicals and correlations with the reaction parameters (Δ*G*° and Δ*G* ^≠^). B) Proposed mechanism for the divergent remote functionalization of amides and amines.

Our mechanistic proposal was centered on a photoredox cycle generating an amidyl (or an *N*‐protected aminyl) radical (**G**) by SET oxidation and fragmentation of the precursor **F** (Scheme 2 B). At this point, enthalpy‐favorable 1,5‐HAT would deliver the distal radical **H**, which should display nucleophilic character. Homolytic atom/group‐transfer (e.g. S_H_2 reaction) with a polarized SOMOphile (X‐Y) would furnish the targeted amides and protected amines **I** as well as the electron‐poor radical Y^.^, which should close the photoredox cycle by SET with the PC^.−^.^21^

Initial efforts focused on the development of γ‐fluorinations using the amide **1 a**, which was prepared on gram scale (Scheme 3).^18^ Pleasingly, blue LED irradiation of **1 a** with Fuzukumi's acridinium (**2 a**) as the photocatalyst (*E ^ox^= +2.1 V vs. SCE),^22^ selectfluor^23^ (**3 a**) as the SOMOphile, and Cs_2_CO_3_ as the base in CH_3_CN/H_2_O gave the desired product **4** in 36 % yield (entry 1). By changing the photocatalyst to the more chemically robust Ir^III^ complex **2 b** the yield was improved to 87 % (entry 2). Using NCS (**3 b**) as the SOMOphile, we developed a γ‐chlorinating process (**5**). Also in this case, the use of **2 b** led to better results than **2 a** (entries 3 and 4), and the organic dye **2 c** was identified as optimum for this transformation (entry 5). A similar trend in photocatalyst‐based efficiency was observed for the γ‐thioetherification using the S‐donor **3 c**, which lead to the formation of **6** in high yield (entries 6–8). Finally, by employing the IBX‐reagents **3 d** 24 and **3 e**,^25^ in the presence of **2 c** as the photocatalyst, we developed cascade reactions resulting in sp^3^ γ‐cyanation (**7**) and γ‐alkynylation (**8**). It is worth mentioning that while the γ‐fluorination of amides has been developed using iron catalysts,^26^ and γ‐chlorinations have been reported using *N*‐Cl‐amides by radical‐chain propagations,^27^ γ‐thioetherification, γ‐cyanation, and γ‐alkynylation are, to the best of our knowledge, unprecedented.

**Scheme 3:**
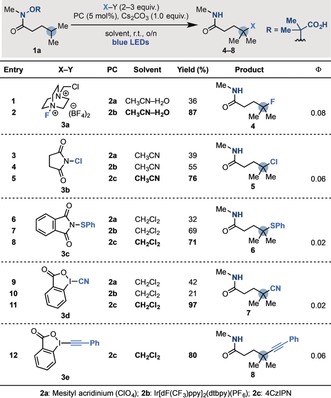
Optimization of the divergent radical functionalization strategy using **1 a. 2 a**=Mesityl acridinium (ClO_4_), **2 b**=Ir[dF(CF_3_)ppy]_2_(dtbpy)(PF_6_), **2 c**=4CzIPN.

To obtain mechanistic insights we conducted luminescence‐quenching studies (Stern–Volmer analysis) which showed that **1** (as its Cs salt) quenches all three photocatalysts **2 a**–**c** at significantly higher rates than all the other SOMOphiles (**3 a**–**e**).^18^ Quantum yields (*Φ*) were determined for all optimized reactions (Scheme 3). The very low values obtained in these studies suggest that mechanisms based on radical‐chain propagations (dark reactivity) should not account for the full reaction productivity, thus corroborating our proposed mechanism.^18^

We then evaluated the scope for this divergent functionalization strategy using a host of functionalized amides and *N*‐protected‐amines (**1 a**–**z** and **1 aa**–**ae**; Scheme 4). In accordance with our computational studies, we performed γ‐fluorination at tertiary (**4**) and both secondary alkyl (**9**) and benzylic (**10**) positions. The process could also be expanded to γ‐terminal centers by the generation of a primary radical, albeit in lower yield (**11**). The *N*‐substitution was also evaluated and we successfully engaged amide precursors with a removable *N*‐Bn group (**12**) as well as an unprotected substrate, which gave the corresponding fluorinated primary amide **13** in good yield.

**Scheme 4:**
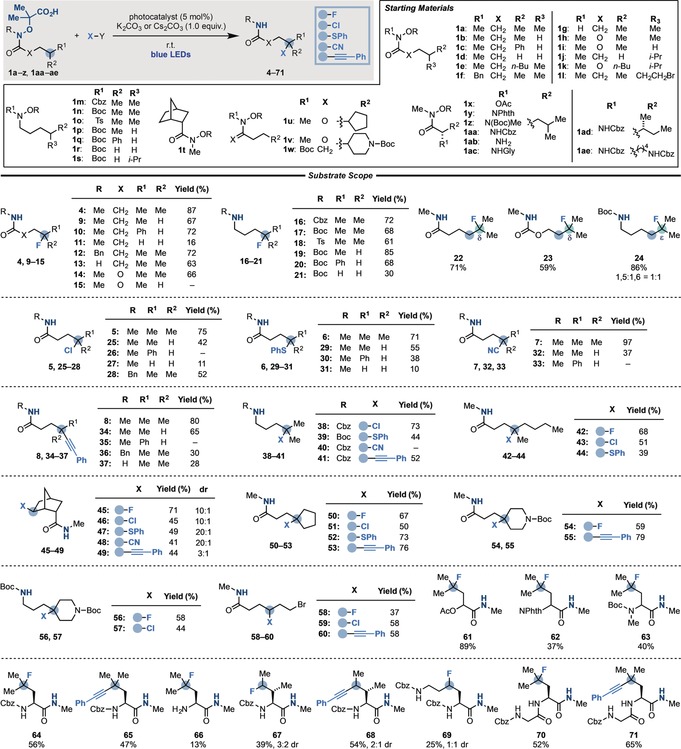
Substrate scope for the divergent remote functionalization of amides and amines. Boc=*tert*‐butoxymethyl, Cbz=benzyloxycarbonyl, Ts=4‐toluenesulfonyl.

The possibility to engage carbamoyl radicals was investigated next, but in this case the reaction was found feasible for only a tertiary center (**14** vs. **15**).

As another element of our substrate scope investigation, we evaluated the development of δ‐fluorination of *N*‐protected amines. We quickly discovered that commonly used *N*‐protecting groups like Cbz (**16**), Boc (**17**), and Ts (**18**) were compatible and provided the desired δ‐fluoro amines in good yields.^28^ The process was also extended to the functionalization of δ‐secondary alkylic (**19**) and benzylic, (**20**) as well as primary centers (**21**).

In general, 1,5‐HAT processes are favored over 1,6‐HATs because of their optimum chairlike six‐membered cyclic transition state. Nevertheless, 1,6‐translocations could be efficiently achieved by providing an enthalpic bias to the system.^20^, ^29^ As demonstrated by the formation of **22** and **23**, there is a preference for 1,6‐HAT when this position is a tertiary center. This observation is in line with an energetically more favorable reaction profile: ΔΔ*G*°(1,6^tert^−1,5^sec^)=−4.7 kcal mol^−1^ and ΔΔ*G* ^≠^(1,6^tert^−1,5^sec^)=−2.4 kcal mol^−1^.^18^ The increased chain flexibility of *N*‐Boc‐amine, however, resulted in mixtures of 1,5‐ (δ) and 1,6‐(*ϵ*)‐fluorination (**24**).

The γ‐chlorination (**5**, **25**–**28**), γ‐thioetherification (**6**, **29**–**31**), γ‐cyanation (**7**, **32**, **33**), and γ‐alkynylation (**8**, **34**–**37**) were also attempted on the same precursors and, much to our liking, were successful on secondary alkyl centers. The functionalization of γ‐secondary benzylic and primary positions was in general less efficient.

The possibility of using the SOMOphiles **3 b**–**e** to obtain δ‐functionalized amines was evaluated next and found to be feasible in good to moderate yields (**38**, **39** and **41**), with the exception of the cyanation process (**40**) that consistently resulted in complex mixtures.

More structurally diverse starting materials were then evaluated with the intention of accessing complex building blocks and obtaining information on the functional groups compatible with the process. We selectively functionalized unsymmetrical tertiary centers (**42**–**44**), as well as γ‐carbon atoms embedded within cyclic motifs such as the rigid norbornene (**45**–**49**) and the cyclopentyl ring (**50**–**53**). This procedure also streamlined access to several C4‐functionalized *N*‐Boc‐piperidines (**54**–**57**) that are valuable building blocks with frequent applications in medicinal chemistry.^30^ The successful formation of **58**–**61** showed that alkyl halides as well as protected alcohols are tolerated, thus giving access to products with handles for further functionalization.

Strategies for the selective modification of natural amino‐acid side chains are a valuable tool for accessing novel and high‐value chemotypes.^31^ In general, this modification is achieved by oxidation followed by stepwise functionalization, and several strategies based on selective H‐atom abstraction have been reported.^16^, ^32^ Using a range of differentially protected leucine derivatives, we were able to perform γ‐fluorination (**62**–**64**) and γ‐alkynylation (**65**). The γ‐fluorination could also be achieved on an unprotected substrate (**66**) albeit in low yield. The successful γ‐alkynylation might have applications given the importance of preparing side‐chain‐modified amino acids with functional groups that undergo click‐type reactions such as azide–alkyne cycloaddition and thiol‐yne reaction.^33^ Pleasingly, this strategy could be extended to the selective late‐stage modification of l‐isoleucine (**67**, **68**) and l‐lysine (**69**), which normally give an isomeric‐mixture under other protocols. Finally, the l‐leucine γ‐functionalization was also expanded to a dipeptide building block (**70**, **71**) in good yield.

In conclusion, we have developed a divergent strategy for the selective functionalization of amides, carbamates, and amines at distal positions. The methodology involves the photoinduced SET oxidation of α‐oxiamido acids to generate electrophilic amidyl radicals. Through a cascade of 1,5‐HAT and S_H_2‐functionalization, fluorine, chlorine, SPh, cyano, and alkyne functionalities can easily be installed. Applications to the selective modification of aminoacids and a dipeptide highlight the compatibility of this approach to motifs relevant to medicinal chemistry.

## Conflict of interest

The authors declare no conflict of interest.

## Supporting information

As a service to our authors and readers, this journal provides supporting information supplied by the authors. Such materials are peer reviewed and may be re‐organized for online delivery, but are not copy‐edited or typeset. Technical support issues arising from supporting information (other than missing files) should be addressed to the authors.

Supplementary
