# Supplementary material for: Photoinduced Remote Functionalization of Amides and Amines Using Electrophilic Nitrogen Radicals
Source: Angew Chem Int Ed Engl. 2018 Aug 29;57(39):12945–9. doi: 10.1002/anie.201807941 (PMC6221136; doi:10.1002/anie.201807941)
Supplement: Supplementary file 1 — Supplementary [file ANIE-57-12945-s001.pdf]

Angewandte Chemie
Eine Zeitschrift der Gesellschaft Deutscher Chemiker

Supporting Information

Photoinduced Remote Functionalization of Amides and Amines Using Electrophilic Nitrogen Radicals

*Sara P. Morcillo[+], Elizabeth M. Dauncey[+], Ji Hye Kim, James J. Douglas, Nadeem S. Sheikh, and Daniele Leonori**

anie_201807941_sm_miscellaneous_information.pdf

Table of Contents

1 General Experimental Details 3

2 Starting Material Synthesis 4

3 Reaction Optimizations 19

- 3.1 Cascade 1,5-HAT Abstraction–Fluorination 19
- 3.2 Cascade 1,5-HAT Abstraction–Chlorination 20
- 3.3 Cascade 1,5-HAT Abstraction–Thioetherification 21
- 3.4 Cascade 1,5-HAT Abstraction–Cyanation 22
- 3.5 Cascade 1,5-HAT Abstraction–Alkynylation 23

4 Reaction Products 24

5 Picture of Reaction set-Up 45

6 Mechanistic Considerations 46

- 6.1 Emission Quenching Experiments 46
- 6.2 Quantum Yield Determination 49
- 6.3 1,5-HAT Abstraction: DFT Studies 50

7 Computational Studies 55

- 7.1 Computational Methods 55
- 7.2 Activation Energy ($\Delta G^{\ddagger}$) and Reaction Energy (ΔG^{o}) for Hydrogen-atom Abstraction 56
- 7.3 Bond Dissociation Enthalpies 96
- 7.4 Electronic Properties of Radicals 126

8 NMR Spectra **132**

9 References **247**

1 General Experimental Details

All required fine chemicals were used directly without purification unless stated otherwise. All air and moisture sensitive reactions were carried out under nitrogen atmosphere using standard Schlenk manifold technique. THF was distilled from sodium/benzophenone, CH_2Cl_2 and was distilled from CaH_2, CH_3CN was distilled from activated 4Å molecular sieves, EtN(*i*-Pr)$_2$ was distilled over KOH. ^{1}H and ^{13}C Nuclear Magnetic Resonance (NMR) spectra were acquired at various field strengths as indicated and were referenced to $CHCl_3$ (7.26 and 77.0 ppm for ^{1}H and ^{13}C respectively). ^{1}H NMR coupling constants are reported in Hertz and refer to apparent multiplicities and not true coupling constants. Data are reported as follows: chemical shift, integration, multiplicity (s = singlet, br s = broad singlet, d = doublet, t = triplet, q = quartet, qi = quintet, sx = sextet, sp = septet, m = multiplet, dd = doublet of doublets, etc.), proton assignment (determined by 2D NMR experiments: COSY, HSQC and HMBC) where possible. High-resolution mass spectra were obtained using a JEOL JMS-700 spectrometer or a Fissions VG Trio 2000 quadrupole mass spectrometer. Spectra were obtained using electron impact ionization (EI) and chemical ionization (CI) techniques, or positive electrospray (ES). Infra-red spectra were recorded using a JASCO FT/IR 410 spectrometer or using an ATI Mattson Genesis Seris FTIR spectrometer as evaporated films or liquid films. Analytical TLC: aluminum backed plates pre-coated (0.25 mm) with Merck Silica Gel 60 F254. Compounds were visualized by exposure to UV-light or by dipping the plates in permanganate ($KMnO_4$) stain followed by heating. Flash column chromatography was performed using Merck Silica Gel 60 (40–63 μm). All mixed solvent eluents are reported as v/v solutions. UV/Vis spectra were obtained using an Agilent 6453 spectrometer and 1 mm High Precision Cell made of quartz from Hellma Analytics.

The LEDs were bought from LEDLightZone.

All the reactions were conducted in CEM 10 mL glass microwave tubes.

2 Starting Material Synthesis

GP1 – General Procedure From Carboxylic Acids via Acid Chlorides

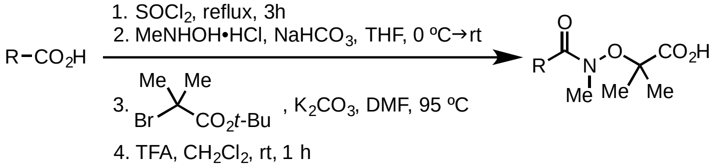

Step 1) To a dry Schlenk tube, under a N_2 atmosphere, was added the carboxylic acid (1.0 equiv.) and thionyl chloride (3.5 equiv.), and the reaction was heated under reflux for 3 h and then evaporated to give the crude acid chloride.

Step 2) To a second dry Schlenk tube, under a N_2 atmosphere, was added *N*-methylhydroxylamine hydrochloride (1.1 equiv.), $NaHCO_3$ (2.0 equiv.) and THF (0.5 M). The mixture was cooled to 0 °C and the crude acid chloride was added dropwise and the reaction mixture which was allowed to warm to room temperature overnight. The reaction was diluted with H_2O, EtOAc and $NaHCO_3$sat. The layers were separated and the aqueous layer was extracted with EtOAc (x 3). The combined organic layers were dried ($MgSO_4$), filtered and evaporated to give the crude hydroxamic acid.

Step 3) To a third Schlenk tube, under a N_2 atmosphere, was added K_2CO_3 (2.0 equiv.) and DMF (0.5 M). The crude hydroxamic acid was solubilised in DMF (0.5 M) and added dropwise to the Schlenk tube. *tert*-Butyl α-bromoisobutyrate (1.1 equiv.) was added and the reaction heated to 95 °C. Once the reaction was complete, the mixture was cooled to room temperature and diluted with brine and Et_2O. The organic layer was separated, washed with brine (x 3), dried ($MgSO_4$), filtered and evaporated to give the crude α-oxyester.

Step 4) The crude α-oxyester was diluted with CH_2Cl_2 (0.2 M), treated with TFA (15 equiv.) and stirred at room temperature for 1 h. The reaction was evaporated and the crude was purified by column chromatography on silica gel eluting with petrol-EtOAc (9:1→1:1).

In few instances Step 1) and Step 2) were replaced with a EDC coupling:

Step 1') A solution of the carboxylic acid (1 equiv.) in CH_2Cl_2 (0.05 M) was cooled to 0 ºC and EDC (1.5 equiv.), N-methyl-hydroxylamine hydrochloride (3.0 equiv.) and Et_3N (4.5 equiv.) were added. The reaction mixture was allowed to warm to room temperature overnight. The mixture was evaporated, diluted with 1N HCl and EtOAc. The layers were separated and the acqueous layer was extracted with EtOAc (x 3), The combined organic layers were dried ($MgSO_4$), filtered and evaporated. Purification by column chromatography on silica gel eluting with petrol-EtOAc (9:1 → 1:1) gave the hydroxyamides.

GP2 – Alternative Method to Synthesise Starting Materials from Commercially Available Acid Chlorides

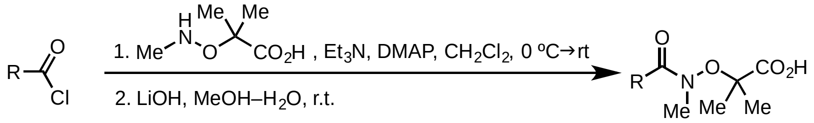

Step 1) A solution of hydroxylamine (1.0 equiv.), DMAP (0.2 equiv.) and Et_3N (2.0 equiv.) in CH_2Cl_2 (0.2M) was cooled to 0 °C. The acid chloride (1.0 equiv.) was added dropwise and the reaction allowed to warm to room temperature overnight. The reaction was diluted with sat. $NaHCO_3$ and extracted with CH_2Cl_2 (x 3). The combined organic layers were dried ($MgSO_4$), filtered and evaporated.

Step 2) The crude methyl ester was dissolved in MeOH–H_2O (0.1 M, 16:1) and LiOH (5.0 equiv.) was added. The reaction was stirred and monitored by TLC analysis until no starting material was observed (4-24 h). The mixture was acidified with HCl (0.1 M) to pH < 2 and diluted with CH_2Cl_2. The layers were separated and the aqueous layer was extracted with CH_2Cl_2 (x 3). The combined organic layers were dried ($MgSO_4$), filtered and evaporated. The residue purified by column chromatography on silica gel eluting with petrol-EtOAc (9:1→1:1).

GP3 – General Procedure from Commercially Available Carboxylic Acids

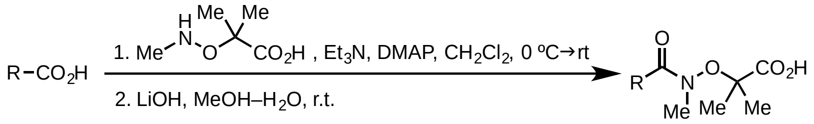

Step 1) A solution of carboxylic acid (1.0 equiv.) hydroxylamine (2.0 equiv.), DMAP (0.3 equiv.) and Et_3N (2.0 equiv.) was prepared in CH_2Cl_2 (0.1M) at room temperature. DCC (2.0 equiv.) was added and the reaction was stirred at room temperature overnight. The reaction was diluted with sat. $NaHCO_3$ and extracted with CH_2Cl_2 (x 3). The combined organic layers were dried ($MgSO_4$), filtered and evaporated.

Step 2) The crude methyl ester was dissolved in MeOH–H_2O (0.1 M, 16:1) and LiOH (5.0 equiv.) was added. The reaction was stirred and monitored by TLC analysis until no starting material was observed (4-24 h). The mixture was acidified with HCl (0.1 M) to pH < 2 and diluted with CH_2Cl_2. The layers were separated and the aqueous layer was extracted with CH_2Cl_2 (x 3). The combined organic layers were dried ($MgSO_4$), filtered and evaporated. The residue purified by column chromatography on silica gel eluting with petrol-EtOAc (9:1→1:1).

GP4 – General Procedure for the Preparation of the Carbamates

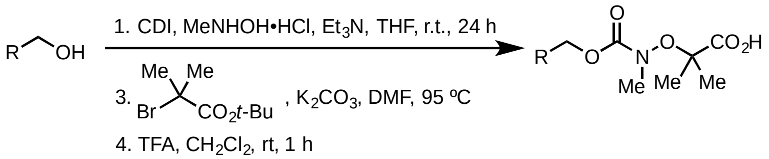

Step 1) A solution of alcohol (1.0 equiv.) in THF (1.0M) was treated with CDI (1.25 equiv.) and stirred at room temperature overnight. *N*-Methylhydroxylamine hydrochloride (1.20 equiv.) and NEt_3 (1.20 equiv.) were added and mixture was stirred at room temperature for 24 h. The mixture was diluted with H_2O and EtOAc and the layers separated. The aqueous layer was extracted with EtOAc (x 3) and the combined organics layers were washed with brine, dried ($MgSO_4$), filtered and evaporated.

Step 2) To a Schlenk tube, under a N_2 atmosphere, was added K_2CO_3 (2.0 equiv.) and DMF (0.5 M). The crude hydroxamic acid was solubilised in DMF (0.5 M) and added dropwise to the Schlenk tube. *tert*-Butyl α-bromoisobutyrate (1.1 equiv.) was added and the reaction heated to 95 °C. Once the reaction was complete, the mixture was cooled to room temperature and diluted with brine and Et_2O. The organic layer was separated, washed with brine (x 3), dried ($MgSO_4$), filtered and evaporated to give the crude α-oxyester.

Step 3) The crude α-oxyester was diluted with CH_2Cl_2 (0.2 M), treated with TFA (15 equiv.) and stirred at room temperature for 1 h. The reaction was evaporated and the crude was purified by column chromatography on silica gel eluting with petrol-EtOAc (9:1→1:1).

***tert*-Butyl 2-((((benzyloxy)carbonyl)amino)oxy)-2-methylpropanoate (S1)**

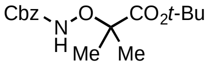

A solution of benzyl hydroxycarbamate (6.5 g, 21 mmol, 1.0 equiv.) in DMF (10 mL) was treated with K_2CO_3 (8 g, 58 mmol, 2.7 equiv.) and *tert*-butyl 2-bromo-2-methylpropanoate (4.6 g, 21 mmol, 1.05 equiv.). The reaction mixture was stirred at rt for 6 h. The mixture was filtered through a pad of Celite washing with CH_2Cl_2 (100 mL). The organic layer was evaporated to give the **S1** (8.4 g, quant.), which was used without further purification. ^{1}H NMR (500 MHz, $CDCl_3$) 7.43–7.29 (5H, m), 5.16 (2H, s), 1.46 (9H, s), 1.45 (6H, s); ^{13}C NMR (126 MHz, $CDCl_3$) δ 173.2, 157.6, 135.9, 128.8, 128.6, 128.5, 84.1, 82.1, 67.6, 28.2, 23.1; HRMS (ESI) Found MNa^+ 332.1468, $C_{16}H_{23}NO_5Na$ requires 332.1474.

methyl 2-(((tert-butoxycarbonyl)amino)oxy)-2-methylpropanoate (S2)

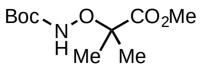

A solution of tert-butyl hydroxycarbamate (6.8 g, 38 mmol, 1.0 equiv.) in DMF (10 mL) was treated with K_2CO_3 (5.2 g, 38 mmol, 1.05 equiv.) and methyl 2-bromo-2-methylpropanoate (5g, 38 mmol, 1.05 equiv.). The reaction mixture was stirred at rt for 6 h. The mixture was filtered through a pad of Celite washing with CH_2Cl_2 (100 mL). The organic layer was evaporated to give the **S2** (6.5 g, 74%), which was used without further purification. ^{1}H NMR ($CDCl_3$, 500 MHz) δ 3.76 (3H, s), 1.50 (6H, s), 1.48 (9H, s); ^{13}C NMR (126 MHz, $CDCl_3$) δ 174.3, 156.9, 83.7, 81.6, 52.4, 28.2, 23.1; HRMS (ESI) Found MNa^+ 298.1638, $C_{13}H_{25}NO_5Na$ requires 298.1630.

Methyl 2-Methyl-2-(((4-methylphenyl)sulfonamido)oxy)propanoate (S3)

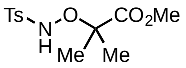

To a solution of 1-carboxy-1-methylethoxyammonium chloride (1.0 g, 6.4 mmol, 2.0 equiv.) in pyridine (2 mL) at 0 ºC was added TsCl (484 mg, 3.2 mmol, 1.0 equiv.) and DMAP (788 mg, 6.4 mmol, 2.0 equiv.). The reaction mixture was stirred at rt for 5 min. The mixture was diluted with AcOEt (100 mL) and 1N HCl until pH ~ 2. The layers were separated and the aqueous layer was extracted with AcOEt (3 x 50mL). The combined organic layers were dried ($MgSO_4$), filtered and evaporated to give the pure product. The crude was solubilised in MeOH (5 mL) and 5 drops of conc H_2SO_4 were added. The solvent was evaporated and the crude passed through a short pad of silica eluting with EtOAc to give **S3** (460 mg, 50%) as an oil. IR ν_{max} (film)/cm^{-1} 1710, 1358, 1220; ^{1}H NMR (500 MHz, $CDCl_3$) δ 7.43 (2H, d, *J* = 7.9, 1.5 Hz), 6.95 (2H, d, *J* = 7.9 Hz), 3.34 (3H, s), 2.06 (3H, s), 1.10 (6H, d, *J* = 1.5 Hz); ^{13}C NMR (126 MHz, $CDCl_3$) δ 174.1, 144.8, 133.8, 129.6, 128.7, 84.1, 52.5, 23.5, 21.7; HRMS (ESI) Found MNa^+ 310.0720, $C_{10}H_{17}NO_5NaS$ requires 310.0725.

GP5 – General Procedure for the Preparation of Protected Hydroxylamines

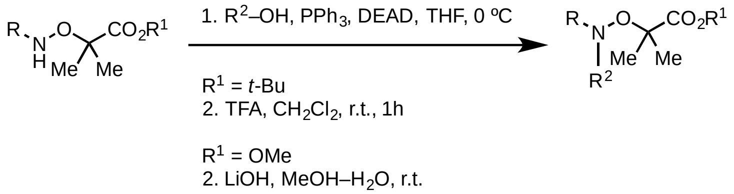

Step 1) A solution of **S1–3** (1.0 equiv.), the alcohol (1.05 equiv.) and PPh_3 (2.0 equiv.) in THF (0.05 M) was cooled to 0 ºC and treated with DEAD (1.5 equiv.). The mixture was

stirred at 0 ºC for 2 h and then evaporated. The residue was purified by silica gel column chromatography on silica gel eluting with petrol–EtOAc (95:5) to give the pure α-oxyester.

Step 2) The α-oxy-*t*-Bu-ester was diluted with CH_2Cl_2 (0.2 M), treated with TFA (15 equiv.) and stirred at room temperature for 1 h. The reaction was evaporated and the crude was purified by column chromatography on silica gel eluting with petrol-EtOAc (9:1→1:1).

Step 2') The α-oxy-Me-ester was solubilised in MeOH–H_2O (0.1 M, 16:1 and treated with LiOH (2.0 equiv.). The reaction was stirred at rt for 1 h. The mixture was evaporated and diluted with EtOAc and 1N HCl until pH ~ 2. The layers were separated and the aqueous layer was extracted with EtOAc (x 3). The combined organic layers were dried ($MgSO_4$), filtered and evaporated. Purification by column chromatography on silica gel eluting with petrol–EtOAc (9:1 → 1:1) gave the pure products.

2-((*N*,4-Dimethylpentanamido)oxy)-2-methylpropanoic Acid (1a)

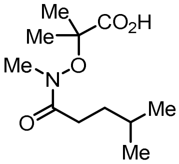

Following **GP1**, 4-methyl pentanoic acid (2.32 g, 20 mmol) gave **1a** (1.85 mg, 40%) as an oil. FT-IR ν_{max} (film)/cm^{-1} 2956, 2871, 1738, 1615, 1410, 1386, 1215, 1180, 1135, 748; ^{1}H NMR (400 MHz, $CDCl_3$) δ 3.37 (3H, s), 2.40–2.32 (2H, m), 1.65–1.52 (3H, m), 1.56 (6H, s), 0.92 (6H, d, *J* = 6.2 Hz); ^{13}C NMR (101 MHz, $CDCl_3$) δ 175.9, 175.2, 86.7, 40.5, 33.4, 30.8, 27.7, 24.2, 22.2; HRMS (ESI) Found MH$^+$ 232.1543, $C_{11}H_{22}NO_4$ requires 232.1543.

2-Methyl-2-((*N*-methylpentanamido)oxy)propanoic Acid (1b)

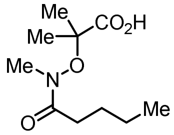

Following **GP1**, pentanoic acid (2.04 g, 20 mmol) gave **1b** (1.55 g, 36%) as an oil. FT-IR ν_{max} (film)/cm^{-1} 2959, 2873, 1737, 1614, 1412, 1386, 1179, 1135, 751; ^{1}H NMR (400 MHz, $CDCl_3$) δ 3.37 (3H, s), 2.37 (2H, t, *J* = 7.5 Hz), 1.71–1.60 (2H, m), 1.55 (6H, s), 1.45–1.32 (2H, m), 0.94 (3H, t, *J* = 7.3 Hz); ^{13}C NMR (101 MHz, $CDCl_3$) δ 175.7, 175.3, 86.7, 40.5, 32.5, 26.7, 24.2, 22.3, 13.7; HRMS (ESI) Found MNa$^+$ 240.1206, $C_{10}H_{19}O_4NNa$ requires 240.1206.

2-Methyl-2-((*N*-methyl-4-phenylbutanamido)oxy)propanoic Acid (1c)

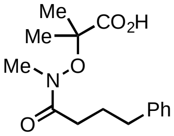

Following **GP1**, 4-phenylbutanoic acid (3.28 g, 20 mmol) gave **1c** (1.89 g, 34%) as an oil. FT-IR ν_{max} (film)/cm^{-1} 3022, 1737, 1615, 1453, 1386, 1215, 1180, 1138, 747; ^{1}H NMR (400 MHz, $CDCl_3$) δ 7.32–7.27 (2H, m), 7.23–7.20 (1H, m), 7.20–7.15 (2H, m), 3.28 (3H, s), 2.70 (2H, t, *J* = 7.4 Hz), 2.35 (2H, t, *J* = 7.4 Hz), 2.09–1.93 (2H, m), 1.54 (6H, s); ^{13}C NMR (101 MHz, $CDCl_3$) δ 175.3, 140.8, 128.6, 128.5, 126.3, 86.7, 40.5, 34.9, 31.6, 26.0, 24.2; HRMS (APCI) Found MH$^+$ 280.1553, $C_{15}H_{22}NO_4$ requires 280.1543.

2-Methyl-2-((*N*-methylbutyramido)oxy)propanoic Acid (1d)

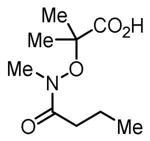

Following **GP1**, butyryl chloride (1.80 g, 17 mmol) gave **1d** (264 mg, 7%) as an oil. FT-IR ν_{max} (film)/cm^{-1} 2968, 1737, 1617, 1465, 1412, 1386, 1216, 1181, 1136, 752; ^{1}H NMR (400 MHz, $CDCl_3$) δ 3.37 (3H, s), 2.35 (2H, t, *J* = 7.4 Hz), 1.81–1.63 (2H, m), 1.55 (6H, s), 0.99 (3H, t, *J* = 7.4 Hz); ^{13}C NMR (101 MHz, $CDCl_3$) δ 175.5, 175.1, 86.7, 40.5, 34.6, 24.2, 18.2, 13.7; HRMS (APCI) Found MH$^+$ 204.1229, $C_9H_{18}NO_4$ requires 204.1230.

2-((*N*,4-Dimethyloctanamido)oxy)-2-methylpropanoic Acid (1e)

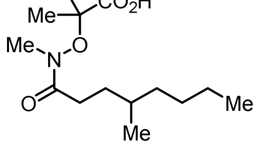

Following **GP3**, 4-methyloctanoic acid (1.0 g, 6.3 mmol) gave **1e** (491 mg, 29%) as an oil. FT-IR ν_{max} (film)/cm^{-1} 2928, 1738, 1619, 1464, 1385, 1215, 1181, 1136, 750; ^{1}H NMR (500 MHz, $CDCl_3$) δ 3.37 (3H, s), 2.39–2.32 (2H, m), 1.73–1.67 (2H, m), 1.56 (6H, s), 1.49–1.44 (3H, m), 1.31–1.26 (4H, m), 0.91–0.87 (6H, m); ^{13}C NMR (126 MHz, $CDCl_3$) δ 176.0, 175.5, 86.8, 40.6, 36.3, 32.4, 31.6, 30.6, 29.1, 27.8, 22.9, 21.1, 19.3, 14.1; HRMS (ASAP) Found MH$^+$ 274.2005, $C_{14}H_{28}O_4N$ requires 274.2013.

2-((*N*-Benzyl-4-methylpentanamido)oxy)-2-methylpropanoic Acid (1f)

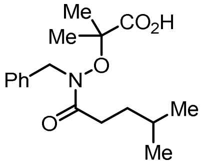

Following **GP1**, 4-methylpentanoic acid (2.32 g, 20 mmol) and *N*-benzylhydroxylamine hydrochloride (3.50 g, 22 mmol) gave **1f** (2.50 g, 41%) as an oil. FT-IR ν_{max} (film)/cm^{-1} 2960, 1740, 1661, 1468, 1371, 1226, 1139, 754; ^{1}H NMR (500 MHz, $CDCl_3$) δ 7.42–7.33 (3H, m), 7.28–7.26 (2H, m), 4.81 (2H, s), 2.38 (2H, t, *J* = 7.5 Hz), 1.73–1.38 (m, 3H), 1.52 (6H, s), 0.86 (6H, d, *J* = 6.0 Hz); ^{13}C NMR (126 MHz, $CDCl_3$, rotamers) δ 176.6, 175.3, 129.1, 128.8, 128.5, 127.2, 87.0, 60.5 & 56.8, 33.7, 30.9, 27.6, 24.3, 22.2; HRMS (ASAP) Found MH$^+$ 308.1849, $C_{17}H_{26}O_4N$ requires 308.1856.

2-(((Isobutoxycarbonyl)(methyl)amino)oxy)-2-methylpropanoic Acid (1h)

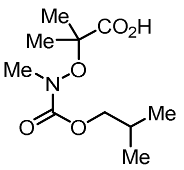

Following **GP4**, 2-methylpropan-1-ol (740 mg, 10 mmol) gave **1h** (110 mg, 5%) as an oil. FT-IR ν_{max} (film)/cm^{-1} 2966, 1694, 1470, 1383, 1330, 1172, 752; ^{1}H NMR (400 MHz, $CDCl_3$) δ 4.01 (2H, d, *J* = 6.7 Hz), 3.23 (3H, s), 2.05–1.95 (1H, m), 1.57 (6H, s), 0.96 (6H, d, *J* = 6.8 Hz); ^{13}C NMR (101 MHz, $CDCl_3$) δ 175.0, 161.7, 86.3, 74.1, 40.6, 27.8, 24.5, 18.8; HRMS (ASAP) Found MH^{+} 234.1331, $C_{10}H_{20}NO_5$ requires 234.1336.

2-((*N*,5-Dimethylhexanamido)oxy)-2-methylpropanoic Acid (1j)

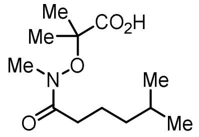

Following **GP1**, 5-methylhexanoic acid (2.21 g, 17 mmol) gave **1j** (1.50 g, 31%) as an oil. FT-IR ν_{max} (film)/cm^{-1} 2955, 1738, 1615, 1466, 1411, 1386, 1215, 1179, 1136, 749; ^{1}H NMR (400 MHz, $CDCl_3$) δ 3.37 (3H, s), 2.34 (2H, t, *J* = 7.6 Hz), 1.72–1.63 (2H, m), 1.60–1.51 (1H, m), 1.55 (6H, s), 1.25–1.18 (2H, m), 0.89 (6H, d, *J* = 6.6 Hz); ^{13}C NMR (101 MHz, $CDCl_3$) δ 175.7, 175.2, 86.7, 40.5, 38.4, 33.0, 27.8, 24.1, 22.6, 22.5; HRMS (ESI) Found MNa^{+} 268.1519, $C_{12}H_{23}O_4NNa$ requires 268.1519.

2-((((*iso*-Pentyloxy)carbonyl)(methyl)amino)oxy)-2-methylpropanoic Acid (1k)

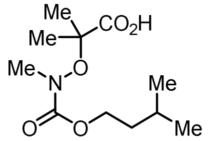

Following **GP4**, 3-methylbutan-1-ol (1.0 g, 11.4 mmol) gave **1k** (659 mg, 23%) as an oil. FT-IR ν_{max} (film)/cm^{-1} 2960, 1693, 1468, 1368, 1214, 1166, 754; ^{1}H NMR (400 MHz, $CDCl_3$) δ 4.26 (2H, t, *J* = 6.8 Hz), 3.21 (3H, s), 1.75–1.64 (1H, m), 1.61–1.55 (2H, m), 1.57 (6H, s), 0.94 (6H, d, *J* = 6.5 Hz); ^{13}C NMR (101 MHz, $CDCl_3$) δ 176.6, 161.8, 86.1, 67.1, 40.6, 37.3, 25.0, 24.1, 22.4; HRMS (ASAP) Found MH^{+} 248.1490, $C_{11}H_{22}NO_5$ requires 248.1492.

2-((6-Bromo-*N*-methylhexanamido)oxy)-2-methylpropanoic Acid (1l)

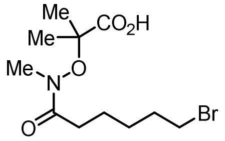

Following **GP2**, 6-bromohexanoyl chloride (1.1 g, 5.1 mmol) gave **1l** (350 mg, 22%) as an oil. FT-IR ν_{max} (film)/cm^{-1} 2939, 1739, 1615, 1412, 1386, 1180, 1137, 751; ^{1}H NMR (500 MHz, $CDCl_3$) δ 3.42 (2H, t, *J* = 6.6 Hz), 3.36 (3H, s), 2.39 (2H, t, *J* = 7.4 Hz), 1.94–1.85 (2H, m), 1.76–1.66 (2H, m), 1.55 (6H, s), 1.54–1.48 (2H, m); ^{13}C NMR (126 MHz, $CDCl_3$) δ 175.2, 175.0, 86.8, 40.8, 33.4, 32.5, 32.3, 27.7, 23.8, 23.70; HRMS (ASAP) Found MH$^+$ 310.0641, $C_{11}H_{21}O_4NBr$ requires 310.0648.

2-((((benzyloxy)carbonyl)(4-methylpentyl)amino)oxy)-2-methylpropanoic Acid (1m)

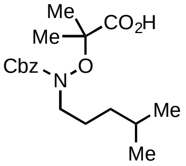

Following **GP5**, **S1** (1.5 g, 6 mmol) gave **1m** (1.3 g, 63%) as an oil. FT-IR ν_{max} (film)/cm^{-1} 1021; ^{1}H NMR (500 MHz, $CDCl_3$) δ 10.81 (1H, br s), 7.37 (5H, br s), 5.24 (2H, s), 3.50 (2H, br s) 1.61 (2H, m), 1.54 (6H, s), 1.52–1.44 (1H, m), 1.15–1.05 (2H, m), 0.83 (6H, d, *J* = 6.6 Hz); ^{13}C NMR (126 MHz, $CDCl_3$) δ 175.6, 161.0, 134.9, 128.9, 128.8, 128.6, 86.4, 69.5, 61.0, 53.2, 35.7, 27.7, 24.3, 22.5; HRMS (APCI) found MH$^+$ 336.1816, $C_{18}H_{26}NO_5$ requires 336.1811.

2-(((*tert*-Butoxycarbonyl)(4-methylpentyl)amino)oxy)-2-methylpropanoic Acid (1n)

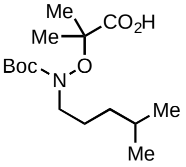

Following **GP5**, **S2** (1.5 g, 6.4 mmol) gave **1n** (1.5 g, 76%) as an oil. FT-IR ν_{max} (film)/cm^{-1} 1709, 1360, 1220; ^{1}H NMR (400 MHz, $CDCl_3$) δ 12.87 (1H, br s), 3.43 (2H, s), 1.71–1.60 (2H, m), 1.61–1.53 (1H, m), 1.53 (6H, s), 1.51 (9H, s), 1.19–1.11 (2H, m), 0.89 (6H, d, *J* = 6.6 Hz); ^{13}C NMR (126 MHz, $CDCl_3$, rotamers) δ 175.5, 160.3, 86.0, 84.2, 53.2, 35.8, 28.2, 27.6, 27.2–21.0 (br s), 24.2, 22.5; HRMS (APCI) Found MH$^+$ 302.1962, $C_{15}H_{28}NO_5$ requires 302.1963.

2-Methyl-2-(((4-methyl-*N*-(4-methylpentyl)phenyl)sulfonamido)oxy)propanoic Acid (1o)

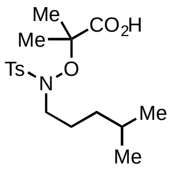

Following **GP5**, **S3** (900 mg, 3.3 mmol) gave **1o** (160 mg, 14%) as an oil. FT-IR ν_{max} (film)/cm^{-1} 1711, 1360, 1220, 800; ^{1}H NMR (500 MHz, $CDCl_3$) δ 9.54 (1H, br s), 7.79 (2H, d, *J* = 7.8 Hz), 7.34 (2H, d, *J* = 7.7 Hz), 3.23 (1H, br s), 2.69 (1H, br s), 2.44 (3H, s), 1.62 (6H, s), 1.64–1.42 (3H, m), 1.19–1.04 (2H, m), 0.83 (6H, d, *J* = 6.6 Hz); ^{13}C NMR (126 MHz, $CDCl_3$) δ 178.2, 145.0, 130.7, 129.8, 129.7, 84.7, 55.4, 36.1, 27.8, 25.6, 24.3, 22.6, 21.8; HRMS (APCI) Found MH^{+} 356.1542, $C_{17}H_{26}NO_5S$ requires 356.1532.

2-(((*tert*-Butoxycarbonyl)(pentyl)amino)oxy)-2-methylpropanoic Acid (1p)

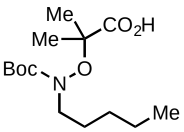

Following **GP5**, **S2** (500 mg, 2.1 mmol) gave **1p** (190 mg, 31%) as an oil. FT-IR ν_{max} (film)/cm^{-1} 2969, 2360, 1739, 1368, 1216, 1136; ^{1}H NMR (400 MHz, CDCl3) δ 3.76–3.21 (2H, m), 1.66–1.56 (1H, m), 1.50 (6H, s), 1.47 (9H, s), 1.37–1.06 (4H, m), 0.87 (3H, t, *J* = 7.1 Hz); ^{13}C NMR (101 MHz, $CDCl_3$) δ 175.8, 160.6, 86.4, 84.6, 53.2, 29.2, 28.5, 26.4, 22.6, 14.3; HRMS (APCI) Found MH^{+} 290.196 $C_{14}H_{28}NO_5$ requires 290.1955.

2-(((*tert*-Butoxycarbonyl)(4-phenylbutyl)amino)oxy)-2-methylpropanoic Acid (1q)

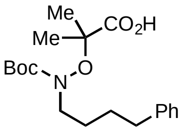

Following **GP5**, **S2** (700 mg, 3.0 mmol) gave **1q** (330 mg, 36%) as an oil. FT-IR ν_{max} (film)/cm^{-1} 1709, 1362, 1220, 1143; ^{1}H NMR (500 MHz, $CDCl_3$) δ 12.81 (1H, br s), 7.36–7.23 (2H, m), 7.22–7.11 (3H, m), 3.47 (2H, br s), 2.64 (2H, t, *J* = 7.0 Hz), 1.79–1.57 (4H, m), 1.51 (6H, s), 1.46 (9H, s); ^{13}C NMR (126 MHz, $CDCl_3$) δ 175.1, 160.1, 141.6, 128.3, 128.2, 125.8, 86.0, 84.3, 52.7, 35.2, 28.2, 28.0, 25.7; HRMS (APCI) Found MH^{+} 350.1973, $C_{19}H_{28}NO_5$ requires 350.1967.

2-(((*tert*-Butoxycarbonyl)(butyl)amino)oxy)-2-methylpropanoic Acid (1r)

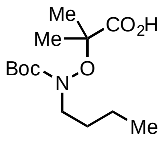

Following **GP5**, **S2** (500 mg, 2.1 mmol) gave **1r** (253 mg, 44%) as an oil. FT-IR ν_{max} (film)/cm^{-1} 2252, 1372, 1259, 1037, 801, 737; ^{1}H NMR (500 MHz, $CDCl_3$) δ 12.93 (1H, s), 3.91–2.98 (2H, m), 1.70–1.57 (2H, m), 1.53 (6H, s), 1.51 (9H, s), 1.32 (2H, sx, *J* = 7.4 Hz), 0.93 (3H, t, *J* = 7.3 Hz); ^{13}C NMR (126 MHz, $CDCl_3$) δ 175.2, 160.2, 86.0, 84.2, 52.5, 28.3, 28.0, 19.7, 13.6; HRMS (APCI) Found MH$^+$ 274.1660, $C_{13}H_{24}NO_5$ requires 274.1654.

2-(((*tert*-Butoxycarbonyl)(5-methylhexyl)amino)oxy)-2-methylpropanoic Acid (1s)

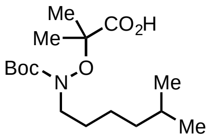

Following **GP5**, **S2** (1.0 g, 4.2 mmol) gave **1s** (650 mg, 44%) as an oil. ^{1}H NMR (400 MHz, $CDCl_3$) δ 3.74–3.10 (2H, m), 1.64–1.56 (1H, m), 1.53 (6H, s), 1.50 (9H, s), 1.31–1.09 (6H, m), 0.87 (6H, d, *J* = 6.6 Hz); ^{13}C NMR (101 MHz, $CDCl_3$) δ 175.5, 160.2, 85.9, 84.1, 52.9, 38.5, 28.1, 27.8, 26.6, 24.5, 22.5, 22.4; HRMS (APCI) Found MH$^+$ 530.2479 $C_{27}H_{36}N_3O_8$ requires 530.2497.

2-Methyl-2-(((1*R*,2*S*,4*S*)-*N*-methylbicyclo[2.2.1]heptane-2-carboxamido)oxy)propanoic Acid (1t)

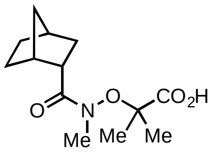

Following **GP1’**, bicyclo[2.2.1]heptane-2-carboxylic acid (1.6 g, 11 mmol) gave **1t** (470 mg, 17 %) as an oil. *endo*:*exo* = 4:1. FT-IR ν_{max} (film)/cm^{-1} 1710, 1359, 1220; ^{1}H NMR (400 MHz, $CDCl_3$, *endo:exo* isomers and rotamers) δ 3.40 (2.2H, s), 3.38 (0.8H, s), 2.85–2.79 (0.8H, m), 2.52–2.44 (0.8H, m), 2.43– 2.40 (0.2H, m), 2.36–2.33 (0.2H, m), 2.39–2.35 (0.2H, m), 2.33–2.28 (0.8H, m), 1.92–1.86 (0.2H, m), 1.85–1.80 (0.8H, m), 1.70–1.60 (3H, m), 1.54 (7H, s), 1.48–1.31 (4H, m); ^{13}C NMR (500 MHz, $CDCl_3$, *endo* isomer) δ 177.1, 175.5, 87.0, 44.1, 43.8, 41.0, 37.0, 36.7, 36.0, 29.6, 29.0, 28.7, 24.5; HRMS (APCI) Found MH$^+$ 256.1543, $C_{13}H_{22}NO_5$ requires 256.1549.

2-((3-Cyclopentyl-N-methylpropanamido)oxy)-2-methylpropanoic Acid (1u)

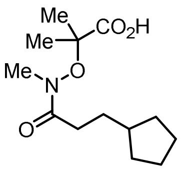

Following **GP3**, 3-cyclopentylpropanoic acid (1.0 g, 7 mmol) gave **1u** (545 mg, 30%) as an oil. FT-IR ν_{max} (film)/cm^{-1} 2944, 2864, 1738 1614, 1453, 1410, 1386, 1215, 1180, 1137, 750; ^{1}H NMR (500 MHz, $CDCl_3$) δ 3.38 (3H, s), 2.37 (2H, t, *J* = 7.8 Hz), 1.84–1.73 (3H, m), 1.71–1.65 (2H, m), 1.65–1.60 (2H, m), 1.58–1.50 (2H, m), 1.56 (6H, s), 1.16–1.06 (2H, m); ^{13}C NMR (126 MHz, $CDCl_3$) δ 175.8, 175.3, 86.8, 40.6, 39.6, 32.4, 32.1, 30.9, 25.1, 24.2; HRMS (ASAP) Found MH$^+$ 258.1694, $C_{13}H_{24}O_4N$ requires 258.1700.

2-((3-(1-(*N*-Butoxycarbonyl)piperidin-4-yl)-*N*-methylpropanamido)oxy)-2-methyl propanoic Acid (1v)

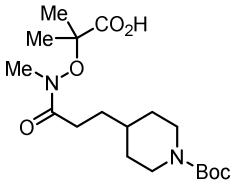

Following **GP3**, 3-(1-(*tert*-butoxycarbonyl)piperidin-4-yl)propanoic acid (500 mg, 1.95 mmol) gave **1v** (285 mg, 39%) as an oil. FT-IR ν_{max} (film)/cm^{-1} 3012, 2932, 1737, 1677, 1427, 1366, 1244, 1216, 1164, 752; ^{1}H NMR (500 MHz, $CDCl_3$) δ 4.17–3.98 (2H, m), 3.35 (3H, s), 2.74–2.57 (2H, m), 2.39 (2H, t, *J* = 7.7 Hz), 1.72–1.58 (4H, m), 1.55 (6H, s), 1.44 (9H, s), 1.36–1.23 (1H, m), 1.19–1.10 (2H, m); ^{13}C NMR (101 MHz, $CDCl_3$) δ 175.5, 175.0, 154.8, 79.5, 77.2, 43.8, 35.4, 31.8, 29.8, 28.5, 25.5, 24.9, 24.1; HRMS (ASAP) Found M$^-$ 371.2183, $C_{18}H_{31}O_6N_2$ requires 371.2188.

2-(((*tert*-Butoxycarbonyl)(3-(1-(*tert*-butoxycarbonyl)piperidin-4-yl)propyl)amino)oxy)-2-methylpropanoic Acid (1w)

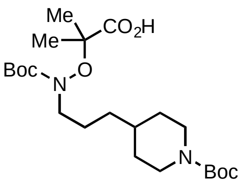

Following **GP5**, **S2** (500 mg, 2.1 mmol) gave **1w** (450 mg, 47%) as an oil. FT-IR ν_{max} (film)/cm^{-1} 1264, 731, 703; ^{1}H NMR (500 MHz, $CDCl_3$) δ 12.54 (1H, br s), 4.03 (2H, s), 3.42 (2H, s), 2.60 (2H, s), 1.58 (4H, d, *J* = 10.9 Hz), 1.46 (6H, s), 1.44 (9H, s), 1.38 (9H, s), 1.37–1.26 (1H, m), 1.24–1.11 (2H, m), 1.07–0.97 (2H, m); ^{13}C NMR (126 MHz, $CDCl_3$, rotamers)

δ 175.6, 160.8, 155.3, 86.5, 84.8, 79.7, 53.4, 47.2–41.6 (br s), 36.1, 33.9, 32.5 (br s), 28.9, 28.6, 23.8; HRMS (ESI) Found MH^+ 443.2763, $C_{22}H_{39}N_2O_7$ requires 443.2751.

2-((2-Acetoxy-*N*,4-dimethylpentanamido)oxy)-2-methylpropanoic Acid (1x)

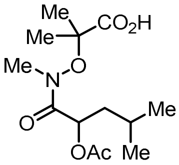

Following **GP1**, (*S*)-2-acetoxy-4-methylpentanoic acid (1.4 g, 8.7 mmol) gave **1x** (636 mg, 25%) as an oil. FT-IR ν_{max} (film)/cm^{-1} 2958, 1743, 1620, 1454, 1387, 1216, 1181, 754; ^1H NMR (500 MHz, $CDCl_3$) δ 5.07 (1H, br s), 3.45 (3H, s), 2.15 (3H, s), 1.89–1.78 (2H, m), 1.59 (3H, s), 1.56 (3H, s), 1.48–1.44 (1H, s), 0.98 (3H, d, *J* = 6.6 Hz), 0.95 (3H, d, *J* = 6.6 Hz); ^{13}C NMR (101 MHz, $CDCl_3$) δ 177.3, 174.7, 171.0, 87.9, 39.1, 25.1, 24.5, 23.2, 21.4, 20.5; HRMS (APCI) Found MH^+ 290.1588, $C_{13}H_{24}O_6N$ requires 290.1598.

2-((2-(1,3-Dioxoisoindolin-2-yl)-*N*,4-dimethylpentanamido)oxy)-2-methylpropanoic Acid (1y)

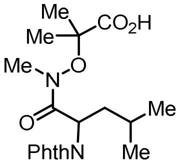

Following **GP3**, 2-(1,3-dioxoisoindolin-2-yl)-4-methylpentanoic acid (260 mg, 1 mmol) gave **1y** (115 mg, 50%) as an oil. FT-IR ν_{max} (film)/cm^{-1}; 3291, 2962, 1708, 1623, 1540, 1388, 1274, 1377, 753; ^1H NMR (500 MHz, $CDCl_3$) δ 7.96–7.84 (1H, m), 7.60 (1H, td, *J* = 7.5, 1.3 Hz), 7.46 (d, *J* = 7.4 Hz, 2H), 5.57 (0.7H, br s), 5.06 (0.3H, br s), 3.61 (1H, s), 3.20 (2H, s), 1.82–1.72 (1H, m), 1.67–1.58 (6H, m), 1.57–1.47 (2H, m), 1.03 (3H, d, *J* = 6.5 Hz), 0.96 (3H, d, *J* = 6.6 Hz); HRMS (APCI) Found MH^+ 377.1704, $C_{19}H_{25}O_6N_2$ requires 377.1707.

6-*isoB*utyl-2,2,4,7,10,10-hexamethyl-5,8-dioxo-3,9-dioxa-4,7-diazaundecanoic Acid (1z)

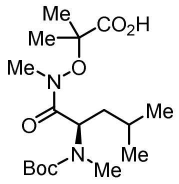

Following **GP3**, *N*-(tert-butoxycarbonyl)-*N*-methyl-*L*-leucine (500 mg, 2 mmol) gave **1z** (315 mg, 44%) as an oil. FT-IR ν_{max} (film)/cm^{-1} 3018, 1744, 1678, 1388, 1368, 1320, 1215, 1155, 751; ^1H NMR (400 MHz, $CDCl_3$, rotamers) δ 5.06 (0.8H, s), 4.83 (0.2H, s), 3.49 (2.4H, s),

3.43 (0.6H, s), 2.80 (3H, s), 1.97–1.77 (1H, m), 1.76–1.64 (2H, m), 1.56 (4.8H, s), 1.53 (1.6H, s), 1.45 (9H, s), 0.95 (6H, t, J = 6.1 Hz); ^{13}C NMR (126 MHz, $CDCl_3$) δ 174.8, 158.2, 158.1, 80.8, 78.3, 51.9, 33.9, 29.6, 28.3, 24.9, 24.7, 22.9, 22.2; HRMS (APCI) Found MH^+ 359.2193, $C_{17}H_{31}O_6N_2$ requires 359.2188.

(*S*)-5-isobutyl-7,9,9-trimethyl-3,6-dioxo-1-phenyl-2,8-dioxa-4,7-diazadecan-10-oic Acid (1aa)

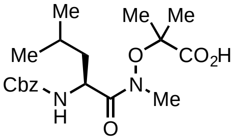

Following **GP1'**, ((benzyloxy)carbonyl)-*L*-leucine (1.6 g, 6.0 mmol) gave **1aa** (764 mg, 33%) as an oil. FT-IR ν_{max} (film)/cm^{-1} 2955, 2360, 2336, 1734, 1653, 1539, 1260, 1137; ^{1}H NMR (DMSO-d_6, 500 MHz) δ 13.15 (1H, br s), 7.54 (1H, d, J = 8.4 Hz), 7.38–7.30 (5H, m), 5.02 (2H, s), 4.63–4.59 (1H, m), 3.10 (3H, s), 1.71–1.63 (1H, m), 1.49 (3H, s), 1.47 (3H, s), 1.43–1.37 (1H, m), 1.34–1.28 (1H, m), 0.865 (3H, d, J = 6.4 Hz), 0.855 (3H, d, J = 6.4 Hz); ^{13}C NMR (126 MHz, DMSO-d_6) δ 175.0, 174.3, 156.2, 137.1, 128.4, 127.8, 127.6, 83.0, 65.4, 50.2, 38.8, 35.8 (br), 24.7, 24.2, 23.3, 21.1, 20.9; HRMS (ESI) Found MH^+ 381.2017, $C_{19}H_{29}N_2O_6$ requires 381.2020.

(*S*)-8-isobutyl-10,12,12-trimethyl-3,6,9-trioxo-1-phenyl-2,11-dioxa-4,7,10-triazatridecan-13-oic Acid (1ac)

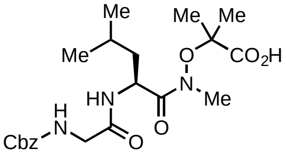

Following **GP1'**, ((benzyloxy)carbonyl)glycyl-*L*-leucine (2.3 g, 7.1 mmol) gave **1ac** (345 mg, 11%) as a solid. FT-IR ν_{max} (film)/cm^{-1} 2963, 2361, 2341, 1718, 1652, 1258, 1016, 865, 793; ^{1}H NMR (500 MHz, DMSO-d_6) δ 13.09 (1H, br s), 8.03 (1H, d, J = 8.2 Hz), 7.39 (1H, t, J = 6.3 Hz), 7.37–7.28 (5H, m), 5.06–4.98 (2H, m), 4.94 (1H, br s), 3.72–3.57 (2H, m), 3.10 (3H, s), 1.67–1.54 (1H, m), 1.48 (3H, s), 1.46 (3H, s), 1.39–1.32 (2H, m), 0.87 (3H, d, J = 6.6 Hz), 0.85 (3H, d, J = 6.5 Hz); ^{13}C NMR (126 MHz, DMSO-d_6) δ 174.5, 174.3, 169.0, 156.4, 137.1, 128.4, 127.8, 127.7, 83.0, 65.4, 47.7, 43.1, 39.8 (overlapped with solvent), 35.5 (br), 24.7, 24.1, 23.3, 21.2, 21.0 (br); HRMS (ESI) Found MH^+ 438.2217, $C_{21}H_{32}N_3O_7$ requires 438.2235.

(*S*)-5-((*S*)-*sec*-butyl)-7,9,9-trimethyl-3,6-dioxo-1-phenyl-2,8-dioxa-4,7-diazadecan-10-oic Acid (1ad)

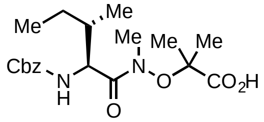

Following **GP1'**, ((benzyloxy)carbonyl)-L-isoleucine (1 g, 3.77 mmol) gave **1ad** (360 mg, 25 %) as an oil. FT-IR ν_{max} (film)/cm^{-1} 2252, 1436, 1374, 1038, 917, 737; ^{1}H NMR (400 MHz, DMSO-d_6, rotamers) δ 13.12 (1H, br s), 7.73–6.87 (5H, m), 5.03 (2H, s), 4.61 (1H, br s), 3.13 (3H, br s), 1.75 (1H, br s), 1.52–1.29 (7H, m), 1.24–1.07 (2H, m) 1.01–0.29 (6H, m); ^{13}C NMR (500 MHz, DMSO-d_6, rotamers) δ 174.4, 174.1 (br s), 156.2, 137.1, 128.3, 127.7, 127.6, 83.0, 65.4, 54.9, 36.4 (br s), 35.4 (br s), 24.3 (br s), 23.9, 21.33 (br s), 15.2, 10.8; HRMS (ESI) Found MH$^+$ 379.1875 $C_{19}H_{27}N_2O_6$ requires 379.1865.

(*S*)-9-(((Benzyloxy)carbonyl)amino)-11,13,13-trimethyl-3,10-dioxo-1-phenyl-2,12-dioxa-4,11-diazatetradecan-14-oic Acid (1ae)

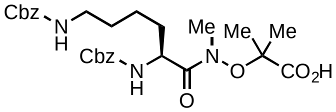

Following **GP1'**, N^2,N^6-bis((benzyloxy)carbonyl)-L-lysine (2 g, 4.8 mmol) gave **1ae** (350 mg, 14%) as an oil. FT-IR ν_{max} (film)/cm^{-1} 1716, 1511, 1264, 895; ^{1}H NMR (500 MHz, DMSO-d_6 rotamers) δ 7.39–7.27 (10H, m), 5.00 (4H, d, *J*=10.3 Hz), 4.49 (1H, br s), 3.11 (3H, s), 2.96 (2H, q, *J*=6.6 Hz), 1.75–1.52 (2H, m), 1.50–1.32 (10H, m); ^{13}C NMR (126 MHz, DMSO-d_6 rotamers) δ 174.7, 174.3, 156.2, 156.1, 137.3, 137.0, 128.4, 127.8, 127.8, 127.7, 127.7, 83.1, 65.4, 65.1, 51.9, 35.9 (br s), 30.7, 29.7, 29.0, 22.9, 21.5; HRMS (APCI) Found MH$^+$ 530.2479, $C_{27}H_{36}N_3O_8$ requires 530.2497.

3 Reaction Optimizations

3.1 Cascade 1,5-HAT Abstraction–Fluorination

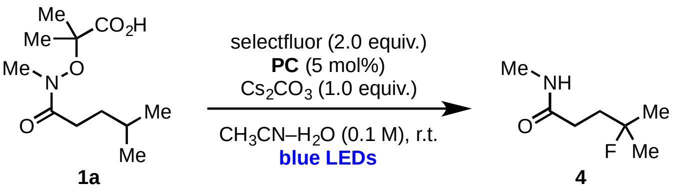

Entry	PC	Time	Yield (%)
**1**	**2a**	2 h	36
**2**	**2a**	16 h	57
**3**	Rh.6G	16 h	12
**4**	**2b**	1 h	87
Control Experiments			
**5**	–	1 h	–
**6**[a]	**2b**	1 h	–
**7**[b]	**2b**	1 h	–

[a] no Cs_2CO_3; [b] no light

2a: $MesAcrClO_4$

2b: IR[dF(CF_3)ppy]$_2$(bpy)(PF_6)

2c: 4CzIPN

3.2 Cascade 1,5-HAT Abstraction–Chlorination

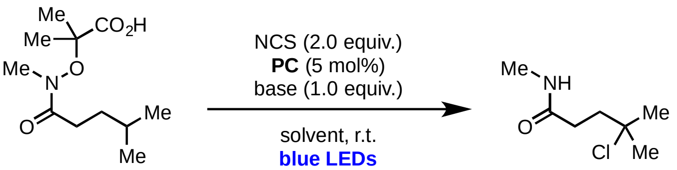

Entry	Base	PC	Solvent (M)	Time	Yield (%)
**1**	K_2CO_3	**2a**	toluene (0.1)	1.5 h	14
**2**	K_2CO_3	**2a**	toluene (0.1)	16 h	19
**3**	K_2CO_3	**2a**	CH_2Cl_2 (0.1)	3 h	22
**4**	K_2CO_3	**2a**	CH_3CN (0.1)	3 h	45
**5**	Cs_2CO_3	**2a**	CH_3CN (0.1)	3 h	58
**6**	Cs_2CO_3	**2b**	CH_3CN (0.1)	3 h	38
**7**	Cs_2CO_3	**2b**	CH_3CN (0.25)	3 h	64
**8**	Cs_2CO_3	**2c**	CH_3CN (0.25)	2 h	76
Control Experiments					
**9**	–	**2c**	CH_3CN (0.25)	2 h	–
**10**	Cs_2CO_3	–	CH_3CN (0.25)	2 h	–
**11**[a]	Cs_2CO_3	**2c**	CH_3CN (0.25)	2 h	–

[a] no light

3.3 Cascade 1,5-HAT Abstraction–Thioetherification

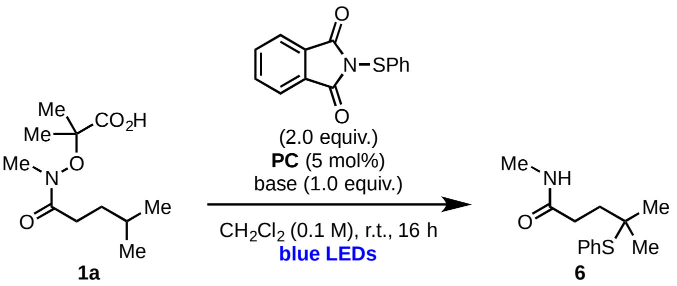

Entry	Base	PC	Yield (%)
**1**	Cs_2CO_3	**2b**	32
**2**	Cs_2CO_3	**2c**	59
**3**	K_2CO_3	**2c**	71
Control Experiments			
**4**	–	**2c**	–
**5**	K_2CO_3	–	–
**6**[a]	K_2CO_3	**2c**	–

[a] no light

3.4 Cascade 1,5-HAT Abstraction–Cyanation

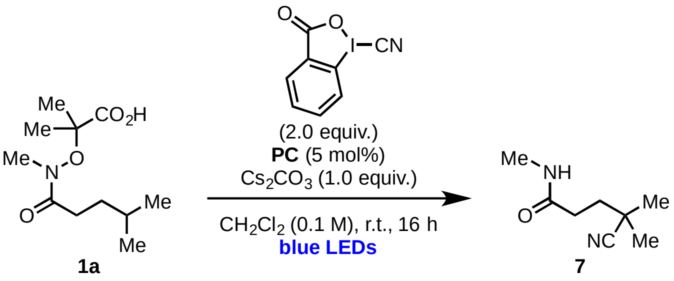

Entry	PC	Yield (%)
**1**	**2a**	42
**2**	**2b**	21
**3**	**2c**	64
Control experiments		
**4**[a]	**2c**	–
**5**	–	–
**6**[b]	**2c**	–

[a] no Cs_2CO_3; [b] no light

3.5 Cascade 1,5-HAT Abstraction–Alkynylation

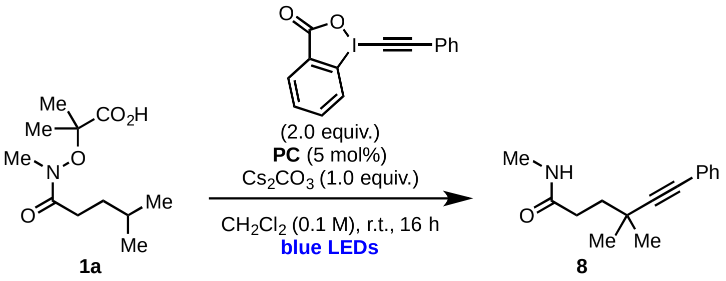

Entry	PC	Yield (%)
**1**	**2c**	80
Control experiments		
**2**[a]	**2c**	–
**3**	–	–
**4**[b]	**2c**	–
[a] no Cs_2CO_3; [b] no light		

4 Reaction Products

GP6 – General Procedure for Remote Functionalization[i]

A dry tube equipped with a stirring bar was charged with the starting material (0.1 mmol, 1.0 equiv.), the photocatalyst (**2b**, **c**) (5 μmol, 5 mol%), the base (0.1 mmol, 1.0 equiv.) and the SOMOphile (**3a**–**e**) (0.2 mmol, 2.0 equiv.) The reaction vessel was sealed, evacuated and back-filled with N_2 (x 3), then sealed with parafilm. The degassed solvent was added, the blue LEDs were switched on and the reaction was stirred under irradiation for the given amount of time. The mixture was diluted with H_2O (1 mL) and EtOAc (1 mL) and the layers were separated. The aqueous layer was extracted with EtOAc (x 2). The combined organic layers were dried ($MgSO_4$), filtered and evaporated. The crude was purified by column chromatography on silica gel.

4-Fluoro-*N*,4-dimethylpentanamide (4)

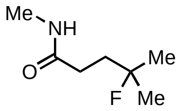

Following **GP6**, **1a** (23 mg, 0.1 mmol) gave **9** (87%) as an oil. FT-IR ν_{max} (film)/cm^{-1} 3320, 2979, 2936, 1646, 1556, 1374; H NMR (400 MHz, $CDCl_3$) δ 5.49 (1H, br s), 2.81 (3H, d, *J* = 4.8 Hz), 2.32–2.26 (2H, m), 2.02–1.94 (2H, m), 1.35 (6H, d, *J* = 21.4 Hz); ^{13}C NMR (126 MHz, $CDCl_3$) δ 173.1, 95.0 (d, *J* = 166.1 Hz), 36.7 (d, *J* = 22.6 Hz), 31.0 (d, *J* = 4.1 Hz), 26.6 (d, *J* = 24.8 Hz), 26.4; ^{19}F NMR (376 MHz, $CDCl_3$) δ –140.9; HRMS (ESI) Found MK$^+$ 186.0688, $C_7H_{14}FNOK$ requires 186.0691.

4-Chloro-*N*,4-dimethylpentanamide (5)

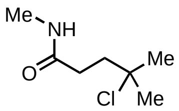

Following **GP6**, **1a** (23 mg, 0.1 mmol) gave **5** (75%) as an oil. FT-IR ν_{max} (film)/cm^{-1} 3245, 2928, 1708, 1651, 1180; ^{1}H NMR (400 MHz, $CDCl_3$) δ 5.51 (1H, br s), 2.75 (3H, d, *J* = 4.9 Hz), 2.37–2.31 (2H, m), 2.06–1.99 (2H, m), 1.51 (6H, s); ^{13}C NMR (126 MHz, $CDCl_3$) δ 172.9, 70.4, 41.1, 32.5, 32.4, 26.4; HRMS (ASAP) Found MH$^+$ 164.0833, $C_7H_{15}ONCl$ requires 164.0837

***N*,4-Dimethyl-4-(phenylthio)pentanamide (6)**

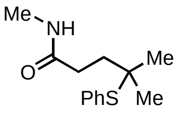

[i] See optimized reaction conditions in Section 3.1–5 for photocatalyst, base, solvent and time.

Following **GP6**, **1a** (23 mg, 0.1 mmol) gave **6** (71%) as an oil. FT-IR ν_{max} (film)/cm^{-1} 3302, 2960, 2927, 1646, 1559, 1264; ^{1}H NMR (400 MHz, $CDCl_3$) δ 7.53–7.48 (2H, m), 7.40–7.29 (3H, m), 5.43 (1H, br s), 2.81 (3H, d, *J* = 4.8 Hz), 2.47–2.38 (2H, m), 1.87–1.78 (2H, m), 1.23 (6H, s); ^{13}C NMR (126 MHz, $CDCl_3$) δ 173.4, 137.4, 131.8, 128.8, 128.6, 48.9, 37.4, 32.3, 28.8, 26.4; HRMS (ASAP) Found MH$^+$ 238.1256, $C_{13}H_{20}ONS$ requires 238.1260.

4-Cyano-*N*,4-dimethylpentanamide (7)

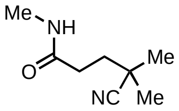

Following **GP6**, **1a** (26 mg, 0.1 mmol) gave **7** (97%) as an oil. FT-IR ν_{max} (film)/cm^{-1} 2252, 1441, 1374, 1038, 917, 737; ^{1}H NMR (500 MHz, $CDCl_3$) δ 5.49 (1H, br s), 2.83 (3H, d, *J* = 4.8 Hz), 2.45–2.23 (2H, m), 1.99–1.82 (2H, m), 1.36 (6H, s); ^{13}C NMR (126 MHz, $CDCl_3$) δ 171.9, 124.5, 36.3, 32.5, 32.2, 29.7, 26.6; HRMS (APCI) Found MH$^+$ 155.1179, $C_8H_{15}N_2O$ requires 155.1179.

***N*,4,4-Trimethyl-6-phenylhex-5-ynamide (8)**

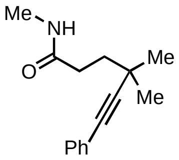

Following **GP6**, **1a** (26 mg, 0.1 mmol) gave **8** (80%) as an oil. FT-IR ν_{max} (film)/cm^{-1} 2253, 1436, 1375, 1038, 917, 737; ^{1}H NMR (500 MHz, $CDCl_3$) δ 7.41–7.31 (2H, m), 7.29–7.22 (3H, m), 5.51 (1H, br s), 2.80 (3H, d, *J* = 4.9 Hz), 2.51–2.35 (2H, m), 1.89–1.81 (2H, m), 1.30 (6H, s); ^{13}C NMR (126 MHz, $CDCl_3$) δ 174.1, 132.0, 128.6, 128.1, 124.1, 96.5, 81.6, 39.2, 33.6, 31.9, 29.6, 26.8; HRMS (APCI) Found MH$^+$ 230.1539, $C_{15}H_{20}NO$ requires 230.1539.

4-Fluoro-*N*-methylpentanamide (9)

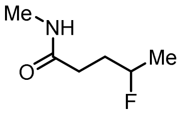

Following **GP6**, **1b** (22 mg, 0.1 mmol) gave **9** (67%) as an oil. FT-IR ν_{max} (film)/cm^{-1} 3321, 2970, 1650, 1546, 1371; ^{1}H NMR (400 MHz, $CDCl_3$) δ 5.53 (1H, br s), 4.79–4.54 (1H, m), 2.81 (3H, d, *J* = 4.8 Hz), 2.41–2.22 (2H, m), 2.10–1.80 (2H, m), 1.34 (3H, dd, *J* = 24.0, 6.1 Hz); ^{13}C NMR (101 MHz, $CDCl_3$) δ 172.8, 90.3 (d, *J* = 164.9 Hz), 32.7 (d, *J* = 20.7 Hz), 32.1 (d, *J* = 3.6 Hz), 26.5, 21.1 (d, *J* = 22.5 Hz); ^{19}F NMR (376 MHz, $CDCl_3$) δ –175.7; HRMS (ESI) Found MNa$^+$ 156.0798, $C_6H_{12}FNONa$ requires 156.0795.

4-Fluoro-*N*-methyl-4-phenylbutanamide (10)

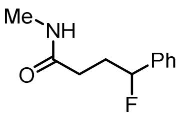

Following **GP6**, **1c** (28 mg, 0.1 mmol) gave **10** (72%) as an oil. FT-IR ν_{max} (film)/cm^{-1} 3301, 2948, 1651, 1567, 1027; ^{1}H NMR (400 MHz, $CDCl_3$) δ 7.39–7.29 (5H, m), 5.50 (1H, ddd, *J* = 48.6, 7.9, 4.7 Hz), 5.41 (1H, br s), 2.81 (3H, d, *J* = 4.9 Hz), 2.38–2.17 (4H, m); ^{13}C NMR (126 MHz, $CDCl_3$) δ 172.5, 139.7 (d, *J* = 19.6 Hz), 128.5, 128.4 (d, *J* = 2.1 Hz), 125.4 (d, *J* = 7.2 Hz), 93.6 (d, *J* = 170.7 Hz), 32.8 (d, *J* = 23.7 Hz), 31.7 (d, *J* = 3.8 Hz), 26.4; ^{19}F NMR (376 MHz, $CDCl_3$) δ –178.3; HRMS (ESI) Found MH^+ 218.0945, $C_{11}H_{14}FNONa$ requires 218.0952.

4-Fluoro-*N*-methylbutanamide (11)

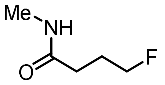

Following **GP6**, **1d** (20 mg, 0.1 mmol) gave **11** (16%) as an oil. FT-IR ν_{max} (film)/cm^{-1} 3303, 2976, 1655, 1215, 1101; ^{1}H NMR (400 MHz, $CDCl_3$) δ 5.45 (1H, br s), 4.46 (2H, dt, *J* = 47.3, 5.7 Hz), 2.79 (3H, d, *J* = 4.7 Hz), 2.29 (1H, t, *J* = 7.3 Hz), 2.20–2.09 (3H, m); ^{19}F NMR (376 MHz, $CDCl_3$) δ –220.8; HRMS (ESI) Found MNa^+ 142.0635, $C_5H_{10}ONFNa$ requires 142.0639.

***N*-Benzyl-4-fluoro-4-methylpentanamide (12)**

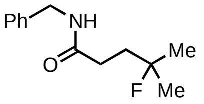

Following **GP6**, **1f** (31 mg, 0.1 mmol) gave **12** (72%) as an oil. FT-IR ν_{max} (film)/cm^{-1} 3294, 2981, 2929, 1647, 1546, 1374, 1216, 1132, 753; ^{1}H NMR (400 MHz, $CDCl_3$) δ 7.37–7.31 (2H, m), 7.31–7.27 (3H, m), 5.77 (1H, br s), 4.44 (2H, d, *J* = 5.7 Hz), 2.39–2.32 (2H, m), 2.07–1.94 (2H, m), 1.57–1.49 (1H, m), 1.36 (6H, d, *J* = 21.4 Hz); ^{13}C NMR (126 MHz, $CDCl_3$) δ 172.3, 138.2, 128.8, 127.9, 127.6, 95.0 (d, *J* = 166.2 Hz), 43.7, 36.6 (d, *J* = 22.6 Hz), 31.0 (d, *J* = 3.9 Hz), 26.6 (d, *J* = 24.8 Hz); ^{19}F NMR (376 MHz, $CDCl_3$) δ –140.8; HRMS (ESI) Found MK^+ 262.0998, $C_{13}H_{18}ONFK$ requires 262.1004.

4-Fluoro-4-methylpentanamide (13)

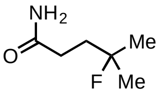

Following **GP6**, **1g** (22 mg, 0.1 mmol) gave **13** (63%) as an oil. ^{1}H NMR (400 MHz, $CDCl_3$) δ 5.57 (2H, br d, *J* = 18.4 Hz), 2.53–2.20 (2H, m), 2.03–1.91 (2H, m), 1.36 (6H, d, *J* = 21.4 Hz); ^{13}C NMR (101 MHz, $CDCl_3$) δ 175.1 95.3 (d, *J* = 166.5 Hz), 36.3 (d, *J* = 23.2 Hz), 30.2 (d, *J* = 4.0 Hz), 26.6 (d, *J* = 24.2 Hz); ^{19}F NMR (376 MHz, $CDCl_3$) δ –141.0. Data in accordance with the literature.[1]

5-Fluoro-*N*,5-dimethylhexanamide (14)

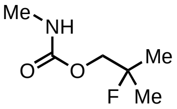

Following **GP6**, **1h** (23 mg, 0.1 mmol) gave **14** (66%). ^{1}H NMR (400 MHz, $CDCl_3$) δ 4.72 (1H, br s), 4.09 (2H, d, *J* = 20.4 Hz), 2.81 (3H, d, *J* = 4.9 Hz), 1.37 (6H, d, *J* = 21.2 Hz); ^{13}C NMR (126 MHz, $CDCl_3$) δ 156.9, 93.9 (d, *J* = 169.2 Hz), 69.7 (d, *J* = 23.9 Hz), 27.8, 23.7 (d, *J* = 24.5 Hz); ^{19}F NMR (376 MHz, $CDCl_3$) δ –146.2; HRMS (ESI) Found MNa^+ 172.0739, $C_6H_{12}O_2NFNa$ requires 172.0744.

Benzyl (4-Fluoro-4-methylpentyl)carbamate (16)

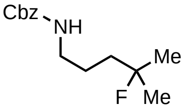

Following **GP6**, **1m** (25 mg, 0.1 mmol) gave **16** (72%) as an oil. ^{1}H NMR (500 MHz, $CDCl_3$) δ 7.52–7.26 (5H, m), 5.10 (2H, br s), 4.84 (1H, br s), 3.69–2.78 (2H, m), 1.97–1.47 (4H, m), 1.34 (6H, d, *J* = 21.4 Hz); ^{13}C NMR (126 MHz, $CDCl_3$) δ 156.5, 136.7, 128.6, 128.2, 95.4 (d, *J* = 165.2 Hz), 66.8, 41.3, 38.4 (d, *J* = 23.3 Hz), 26.8 (d, *J* = 24.8 Hz), 24.7 (d, *J* = 4.7 Hz); ^{19}F NMR ($CDCl_3$, 376 MHz) δ –138.5; HRMS (APCI) Found MH^+ 254.1558, $C_{14}H_{21}FNO_2$ requires 254.1556.

***tert*-Butyl (4-fluoro-4-methylpentyl)carbamate (17)**

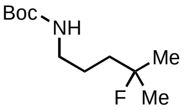

Following **GP6**, **1n** (30 mg, 0.1 mmol) gave **17** (68%) as an oil. FT-IR ν_{max} (film)/cm^{-1} 1710, 1359, 1219; ^{1}H NMR (500 MHz, $CDCl_3$, rotamers) δ 4.55 (1H, s), 3.13 (2H, br s), 1.67–1.55 (4H, m), 1.49–1.41 (9H, s), 1.34 (6H, d, *J*=21.4 Hz); ^{13}C NMR (126 MHz, $CDCl_3$) δ 155.9,

95.3 (d, J = 165.1 Hz), 79.0, 40.6, 38.3 (d, J = 23.3 Hz), 28.3, 26.5 (d, J = 24.8 Hz), 24.6 (d, J = 4.2 Hz); ^{19}F NMR ($CDCl_3$, 376 MHz) δ –138.2; HRMS (APCI) Found MH^+ 220.1707 , $C_{11}H_{23}NO_2F$ requires 220.1705.

_N_-(4-Fluoro-4-methylpentyl)-4-methylbenzenesulfonamide (18)

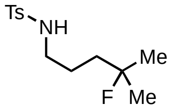

Following **GP6**, **1o** (36 mg, 0.1 mmol) gave **18** (61%) as an oil. FT-IR ν_{max} (film)/cm^{-1} 1259, 1035, 799; ^{1}H NMR (500 MHz, $CDCl_3$) δ 7.74 (2H, d, J = 8.2 Hz), 7.31 (2H, d, J = 8.2 Hz), 4.86–4.26 (1H, m), 2.96 (2H, q, J = 6.2 Hz), 2.43 (3H, s), 1.63–1.51 (4H, m), 1.29 (6H, d, J = 21.4 Hz); ^{13}C NMR (126 MHz, $CDCl_3$) δ 143.8, 137.1, 129.9, 127.2, 95.4 (d, J = 165.3 Hz), 43.5, 38.2 (d, J = 23.3 Hz), 26.7 (d, J = 24.7 Hz), 24.4 (d, J = 4.3 Hz), 21.7; ^{19}F NMR ($CDCl_3$, 376 MHz) δ –138.9; HRMS (APCI) Found MH^+ 274.1272, $C_{13}H_{21}NO_2FS$ requires 274.1270.

***tert*-Butyl (4-Fluoropentyl)carbamate (19)**

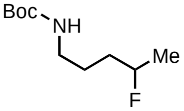

Following **GP6**, **1p** (28 mg, 0.1 mmol) gave **19** (85%) as an oil. FT-IR ν_{max} (film)/cm^{-1} 1709, 1362, 1220, 1170, 1091, 804; ^{1}H NMR (500 MHz, $CDCl_3$) δ 4.76–4.58 (1H, m), 4.54 (1H, br s), 3.38–2.91 (2H, m), 1.75–1.50 (4H, m), 1.44 (9H, s), 1.32 (3H, dd, J = 23.9, 6.1 Hz); ^{13}C NMR (126 MHz, $CDCl_3$) δ 156.2, 90.8 (d, J = 164.7 Hz), 79.4, 40.5, 34.3 (d, J = 20.9 Hz), 28.7, 26.1, 21.3 (d, J = 22.7 Hz); ^{19}F NMR ($CDCl_3$, 376 MHz) δ –175.3; HRMS (APCI) Found MH^+ 204.1405, $C_{11}H_{21}NO_2F$ requires 204.1398.

***tert*-Butyl (4-Fluoro-4-phenylbutyl)carbamate (20)**

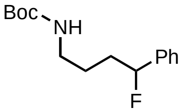

Following **GP6**, **1q** (35 mg, 0.1 mmol) gave **20** (68%) as an oil. FT-IR ν_{max} (film)/cm^{-1} 1711, 1362, 1219, 1171, 1091, 804; ^{1}H NMR (500 MHz, $CDCl_3$) δ 7.40–7.36 (2H, m), 7.35–7.28 (3H, m), 5.46 (1H, ddd, J = 47.8, 8.2, 4.6 Hz), 4.52 (1H, s), 3.27–3.05 (2H, m), 2.10–1.75 (2H, m), 1.70–1.53 (2H, m), 1.44 (9H, s); ^{13}C NMR (126 MHz, $CDCl_3$) δ 155.8, 140.0 (d, J = 19.5 Hz), 128.3, 128.2 (d, J = 2.0 Hz), 125.3 (d, J = 7.0 Hz), 94.1 (d, J = 170.8 Hz), 79.1,

40.0, 34.3 (d, J = 23.9 Hz), 28.3, 25.7 (d, J = 3.8 Hz); ^{19}F NMR ($CDCl_3$, 376 MHz) δ –175.3; HRMS (APCI) Found MH^+ 268.2716, $C_{15}H_{23}NO_2F$ requires 268.1713.

tert-Butyl (4-Fluorobutyl)carbamate (21)

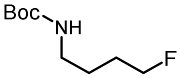

Following **GP6**, **1r** (27 mg, 0.1 mmol) gave **21** (30%) as an oil. FT-IR ν_{max} (film)/cm^{-1} 1022; ^{1}H NMR (500 MHz, $CDCl_3$) δ 4.46 (2H, dt, J = 47.2, 5.9 Hz), 3.27–3.03 (2H, m), 2.07–1.54 (4H, m), 1.44 (9H, s); ^{13}C NMR (126 MHz, $CDCl_3$) δ 155.8, 83.6 (d, J = 164.7 Hz), 79.1, 40.0, 28.3, 27.6 (d, J = 20.1 Hz), 26.0; ^{19}F NMR ($CDCl_3$, 376 MHz) δ –218.4; HRMS (APCI) Found MH^+ 214.1214, $C_9H_{18}NO_2FNa$ requires 214.1208.

5-Fluoro-_N_,5-dimethylhexanamide (22)

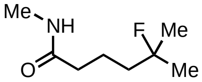

Following **GP6**, **1j** (25 mg, 0.1 mmol) gave **22** (71%) as an oil. FT-IR ν_{max} (film)/cm^{-1} 3319, 2956, 1666, 1401, 1360; ^{1}H NMR (400 MHz, $CDCl_3$) δ 5.48 (1H, br s), 2.81 (3H, d, J = 4.9 Hz), 2.19 (2H, t, J = 7.3 Hz), 1.81–1.69 (2H, m), 1.69–1.57 (2H, m), 1.34 (6H, d, J = 21.5 Hz); ^{13}C NMR (126 MHz, $CDCl_3$) δ 173.2, 95.6 (d, J = 164.6 Hz), 40.6 (d, J = 22.9 Hz), 36.5, 26.6 (d, J = 24.8 Hz), 26.3, 20.2 (d, J = 4.9 Hz); ^{19}F NMR (376 MHz, $CDCl_3$) δ –137.7; HRMS (ESI) Found MNa^+ 184.1110, $C_8H_{16}ONFNa$ requires 184.1108.

3-Fluoro-3-methylbutyl Methylcarbamate (23)

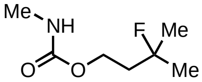

Following **GP6**, **1k** (25 mg, 0.1 mmol) gave **23** (37%). ^{1}H NMR (400 MHz, $CDCl_3$) δ 5.68 (1H, s), 4.27–4.16 (2H, m), 2.78 (3H, d, J = 4.7 Hz), 1.73–1.61 (2H, m), 1.38 (6H, d, J = 21.4 Hz); ^{13}C NMR (126 MHz, $CDCl_3$) δ 157.1, 94.3 (d, J = 165.7 Hz), 60.9 (d, J = 6.5 Hz), 40.2 (d, J = 23.1 Hz), 27.0 (d, J = 24.6 Hz), 26.9; ^{19}F NMR (376 MHz, $CDCl_3$) δ –137.8; HRMS (ESI) Found MNa^+ 186.0901, $C_7H_{14}O_2NFNa$ requires 186.0901.

tert-Butyl (4-Fluoro-5-methylhexyl)carbamate (24)

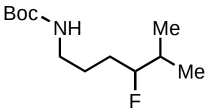

Following **GP6**, **1s** (52 mg, 0.1 mmol) gave **24** (44%) as an oil. FT-IR ν_{max} (film)/cm^{-1} 1738, 1365, 1264, 1217, 906, 728, 649; ^{1}H NMR (500 MHz, $CDCl_3$) δ 4.54 (1H, s), 4.32–4.07 (1H, m), 3.23–3.09 (2H, m), 1.86–1.72 (1H, m), 1.63–1.51 (2H, m), 1.44 (9H, s), 1.31–1.14 (2H, m), 0.93 (6H, dd, *J* = 10.2, 6.8 Hz); ^{13}C NMR (126 MHz, $CDCl_3$) δ 156.1, 98.5 (d, *J* = 170.8 Hz), 79.2, 40.5, 32.5 (d, *J* = 20.1 Hz), 29.5 (d, *J* = 21.7 Hz), 28.6, 22.7, 18.4 (d, *J* = 5.5 Hz), 17.3 (d, *J* = 6.3 Hz); ^{19}F NMR ($CDCl_3$, 376 MHz) δ –186.9; HRMS (ASAP) Found MH$^+$ 232.1898, $C_{12}H_{23}NOF$ requires 232.1713.

tert-Butyl (5-Fluoro-5-methylhexyl)carbamate (24')

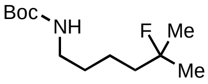

Following **GP6**, **1s** (52 mg, 0.1 mmol) gave **24'** (42%) as an oil. FT-IR ν_{max} (film)/cm^{-1} 1738, 1365, 1264, 1217, 906, 728, 649; ^{1}H NMR (500 MHz, $CDCl_3$) δ 4.51 (1H, s), 3.13 (2H, q, *J* = 6.7 Hz), 1.66–1.54 (2H, m), 1.52–1.38 (4H, m), 1.44 (9H, s), 1.33 (6H, d, *J* = 21.4 Hz); ^{13}C NMR (126 MHz, $CDCl_3$) δ 156.1, 95.7 (d, *J* = 164.8 Hz), 79.3, 41.1 (d, *J* = 23.0 Hz), 40.6, 30.5, 28.6, 26.8 (d, *J* = 24.9 Hz), 21.3 (d, *J* = 5.2 Hz); ^{19}F NMR ($CDCl_3$, 376 MHz) δ –138.0; HRMS (ASAP) Found MH$^+$ 232.1898, $C_{12}H_{23}NOF$ requires 232.1713.

4-Chloro-_N_-methylpentanamide (25)

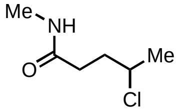

Following **GP6**, **1b** (22 mg, 0.1 mmol) gave **25** (42%) as an oil. FT-IR ν_{max} (film)/cm^{-1} 3159, 2991, 1750, 1708, 1182, 1807; ^{1}H NMR (400 MHz, $CDCl_3$) δ 5.57 (1H, br s), 4.14–3.99 (1H, m), 2.81 (3H, d, *J* = 4.7 Hz), 2.46–2.28 (2H, m), 2.24–2.13 (2H, m), 1.53 (3H, d, *J* = 6.5 Hz); ^{13}C NMR (126 MHz, $CDCl_3$) δ 172.6, 58.4, 35.7, 33.5, 26.4, 25.5; HRMS (ASAP) Found MH$^+$ 150.0677, $C_6H_{13}NOCl$ requires 150.0680.

_N_-Benzyl-4-chloro-4-methylpentanamide (28)

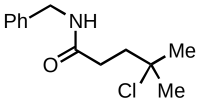

Following **GP6**, **1f** (31 mg, 0.1 mmol) gave **28** (75%) as an oil. FT-IR ν_{max} (film)/cm^{-1} 3304, 2925, 2853, 1644, 1543; ^{1}H NMR (500 MHz, $CDCl_3$) δ 7.37–7.32 (2H, m), 7.31–7.27 (3H,

m), 5.75 (1H, br s), 4.45 (2H, d, J = 5.7 Hz), 2.49–2.42 (2H, m), 2.17–2.11 (2H, m), 1.59 (6H, s); ^{13}C NMR (126 MHz, $CDCl_3$) δ 172.0, 138.2, 128.8, 127.9, 127.6, 70.3, 43.8, 41.0, 32.6, 32.5; HRMS (ASAP) Found MH^+ 240.1146, $C_{13}H_{19}ONCl$ requires 240.1150.

***N*-methyl-4-(phenylthio)pentanamide (29)**

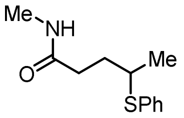

Following **GP6**, **1b** (22 mg, 0.1 mmol) gave **29** (55%) as an oil. FT-IR ν_{max} (film)/cm^{-1} 2360, 2253, 1436, 1375, 1038, 918, 737; ^{1}H NMR (500 MHz, $CDCl_3$) δ 7.39 (2H, d, J = 7.4 Hz), 7.29 (2H, t, J = 7.4 Hz), 7.23 (1H, t, J = 7.3 Hz), 5.45 (1H, br s), 3.31–3.16 (1H, m), 2.78 (3H, d, J = 4.8 Hz), 2.39–2.35 (2H, m), 2.01–1.92 (1H, m), 1.88–1.77 (1H, m), 1.29 (3H, d, J = 6.8 Hz); ^{13}C NMR (126 MHz, $CDCl_3$) δ 172.9, 134.7, 132.1, 128.9, 126.9, 43.1, 33.8, 32.3, 26.3, 21.5; HRMS (ESI) Found MH^+ 224.1097, $C_{12}H_{18}ONS$ requires 224.1104.

***N*-methyl-4-phenyl-4-(phenylthio)butanamide (30)**

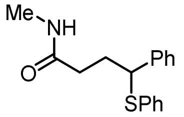

Following **GP6**, **1c** (28 mg, 0.1 mmol) gave **30** (38%) as an oil. FT-IR ν_{max} (film)/cm^{-1} 2926, 2357, 2339, 1651, 1264, 906, 729; ^{1}H NMR (500 MHz, $CDCl_3$) δ 7.26–7.16 (10H, m), 5.27 (1H, br s), 4.21–4.16 (1H, m), 2.76 (3H, d, J = 4.9 Hz), 2.36–2.16 (4H, m); ^{13}C NMR (126 MHz, $CDCl_3$) δ 172.5, 141.3, 134.5, 132.4, 128.7, 128.5, 127.8, 127.3, 127.2, 52.7, 34.2, 31.8, 26.3; HRMS (ESI) Found MNa^+ 308.1077, $C_{17}H_{19}ONSNa$ requires 308.1080.

4-Cyano-*N*-methylpentanamide (32)

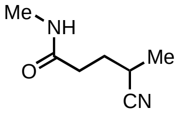

Following **GP6**, **1b** (26 mg, 0.1 mmol) gave **32** (37%) as an oil. FT-IR ν_{max} (film)/cm^{-1} 2253, 1441, 1374, 1038, 917, 737; ^{1}H NMR (500 MHz, $CDCl_3$) δ 5.50 (1H, br s), 2.82 (3H, d, J = 4.9 Hz), 2.79–2.69 (1H, m), 2.53–2.25 (2H, m), 2.11–1.99 (1H, m), 1.89–1.77 (1H, m), 1.34 (3H, d, J = 7.1 Hz); ^{13}C NMR (126 MHz, $CDCl_3$) δ 172.0, 122.9, 33.8, 30.0, 26.8, 25.5, 18.5; HRMS (APCI) Found MH^+ 141.1022, $C_7H_{13}N_2O$ requires 141.1025.

N,4-Dimethyl-6-phenylhex-5-ynamide (34)

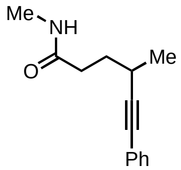

Following **GP6**, **1b** (24 mg, 0.1 mmol) gave **34** (65%) as an oil. FT-IR ν_{max} (film)/cm^{-1} 2253, 1436, 1374, 1038, 917, 737; ^{1}H NMR (500 MHz, $CDCl_3$) δ 7.39–7.35 (2H, m), 7.32–7.19 (3H, m), 5.52 (1H, br s), 2.79 (3H, d, J = 4.9 Hz), 2.68 (1H, dqd, J = 9.9, 6.9, 4.8 Hz), 2.47–2.29 (2H, m), 2.04–1.88 (1H, m), 1.80–1.68 (1H, m), 1.26 (3H, d, J = 6.9 Hz); ^{13}C NMR (126 MHz, $CDCl_3$) δ 173.5, 131.8, 128.5, 127.9, 123.9, 93.7, 81.8, 34.78, 32.8, 26.6, 26.5, 21.3; HRMS (APCI) Found MH$^+$ 216.1383, $C_{14}H_{18}NO$ requires 216.1383.

N-Benzyl-4,4-dimethyl-6-phenylhex-5-ynamide (36)

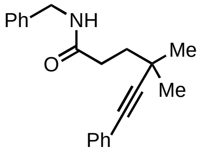

Following **GP6**, **1f** (31 mg, 0.1 mmol) gave **36** (30%) as an oil. FT-IR ν_{max} (film)/cm^{-1} 3315, 2962, 1651, 1538, 1454, 908; ^{1}H NMR (400 MHz, $CDCl_3$) δ 7.41–7.31 (6H, m), 7.30–7.24 (4H, m), 5.76 (1H, br s), 4.44 (2H, d, J = 5.6 Hz), 2.53–2.41 (2H, m), 1.92–1.81 (2H, m), 1.30 (6H, s); ^{13}C NMR (126 MHz, $CDCl_3$) δ 172.8, 138.3, 131.6, 128.7, 128.2, 127.9, 127.6, 127.5, 123.6, 96.0, 81.2, 43.8, 38.7, 31.5, 29.2, 22.2; HRMS (ASAP) Found MH$^+$ 306.1843, $C_{21}H_{24}ON$ requires 306.1852.

Benzyl (4-Chloro-4-methylpentyl)carbamate (38)

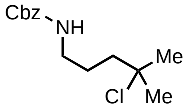

Following **GP6**, **1m** (34 mg, 0.1 mmol) gave **38** (73%) as an oil. ^{1}H NMR (500 MHz, $CDCl_3$) δ 7.41–7.29 (5H, m), 5.10 (2H, s), 4.79 (1H, s), 3.30–3.16 (2H, m), 1.83–1.66 (4H, m), 1.56 (6H, s); ^{13}C NMR (126 MHz, $CDCl_3$) δ 156.8, 136.9, 128.9, 128.5, 70.8, 67.1, 43.3, 41.4, 32.8, 26.2; HRMS (APCI) Found MH$^+$172.1129, $C_9H_{15}NO_2F$ requires 172.1132.

tert-Butyl (4-methyl-4-(phenylthio)pentyl)carbamate (39)

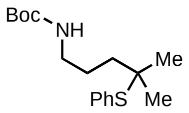

Following **GP6**, **1n** (32 mg, 0.1 mmol) gave **39** (44%) as an oil. FT-IR ν_{max} (film)/cm^{-1} 3323, 2941, 28311447, 1022; ^{1}H NMR (500 MHz, $CDCl_3$) δ; 7.56–7.44 (2H, m), 7.39–7.29 (3H, m), 4.52 (1H, s), 3.11 (2H, q, *J*=6.8 Hz), 1.73–1.63 (2H, m), 1.48–1.42 (2H, m), 1.45 (9H, s), 1.22 (6H, s);^{13}C NMR (126 MHz, $CDCl_3$) δ 155.8, 137.4, 132.0, 128.6, 128.4, 79.11, 48.8, 40.7, 39.1, 28.7, 28.3, 25.5; HRMS (ESI) Found MNa^{+} 332.1655, $C_{17}H_{27}NO_2SNa$ requires 332.1643.

Benzyl (4,4-Dimethyl-6-phenylhex-5-yn-1-yl)carbamate (41)

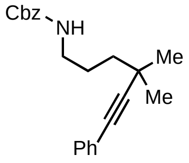

Following **GP6**, **1m** (34 mg, 0.1 mmol) gave **41** (52%) as an oil. FT-IR ν_{max} (film)/cm^{-1} 1709, 1359, 1220; ^{1}H NMR (500 MHz, $CDCl_3$) δ 7.63–7.30 (5H, m), 7.26 (5H, br s), 5.10 (2H, s), 4.77 (1H, br s), 3.31–3.17 (2H, m), 1.81–1.65 (2H, m), 1.52–1.43 (2H, m), 1.28 (6H, s); ^{13}C NMR (126 MHz, $CDCl_3$) δ 156.5, 136.8, 131.7, 128.7, 128.3, 127.7, 124.0, 96.9, 80.8, 66.8, 41.5, 40.5, 31.6, 29.4, 26.3; HRMS (APCI) Found MH^{+} 336.1945, $C_{22}H_{26}NO_2$ requires 336.1958.

4-Fluoro-*N*,4-dimethyloctanamide (42)

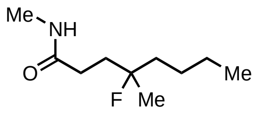

Following **GP6**, **1e** (27 mg, 0.1 mmol) gave **42** (68%) as an oil. FT-IR ν_{max} (film)/cm^{-1} 2990, 2935, 2861, 1647, 1557, 1448, 1251, 1047, 908, 732; ^{1}H NMR (500 MHz, $CDCl_3$) δ 5.47 (1H, br s), 2.81 (3H, d, *J* = 4.9 Hz), 2.33–2.23 (2H, m), 2.04–1.85 (2H, m), 1.67–1.51 (4H, m), 1.35–1.31 (2H, m), 1.03 (3H, d, *J* = 21.8 Hz), 0.91 (3H, t, *J* = 7.0 Hz); ^{13}C NMR (126 MHz, $CDCl_3$) δ 173.2, 97.0 (d, *J* = 167.8 Hz), 39.5 (d, *J* = 22.6 Hz), 35.0 (d, *J* = 22.8 Hz), 30.7 (d, *J* = 4.3 Hz), 26.4, 25.9 (d, *J* = 6.2 Hz), 23.9 (d, *J* = 24.9 Hz), 23.1, 14.0; ^{19}F NMR (376 MHz, $CDCl_3$) δ –145.9; HRMS (ESI) Found MNa^{+} 212.1416, $C_{10}H_{20}ONFNa$ requires 212.1421.

4-Chloro-*N*,4-dimethyloctanamide (43)

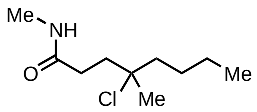

Following **GP6**, **1e** (27 mg, 0.1 mmol) gave **43** (51%) as an oil. FT-IR ν_{max} (film)/cm^{-1} 3314, 2959, 2360, 1713, 1651, 1557, 909; ^{1}H NMR (500 MHz, $CDCl_3$) δ 5.48 (1H, br s), 2.82 (3H, d, *J* = 4.9 Hz), 2.42–2.35 (2H, m), 2.17–2.02 (2H, m), 1.80–1.68 (2H, m), 1.51 (3H, s), 1.47–1.38 (2H, m), 1.35–1.29 (2H, m), 0.92 (3H, t, *J* = 7.3 Hz); ^{13}C NMR (126 MHz, $CDCl_3$) δ 173.1, 74.4, 44.4, 39.5, 32.3, 29.6, 27.0, 26.6, 23.0, 14.1; HRMS (APCI) Found MH^{+} 206.1305, $C_{10}H_{21}ONCl$ requires 206.1306.

***N*,4-Dimethyl-4-(phenylthio)octanamide (44)**

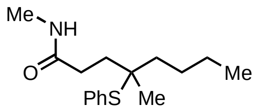

Following **GP6**, **1e** (27 mg, 0.1 mmol) gave **44** (39%) as an oil. FT-IR ν_{max} (film)/cm^{-1} 3302, 2928, 2857, 1644, 1555, 1466, 906, 730; ^{1}H NMR (400 MHz, $CDCl_3$) δ 7.46–7.38 (2H, m), 7.33–7.21 (3H, m), 5.35 (1H, br s), 2.74 (3H, d, *J* = 4.8 Hz), 2.38–2.24 (2H, m), 1.75–1.68 (2H, m), 1.38–1.32 (4H, m), 1.27–1.20 (2H, m), 1.19 (3H, s), 0.84 (3H, t, *J* = 7.3 Hz); ^{13}C NMR (126 MHz, $CDCl_3$) δ 137.4, 128.8, 128.6, 52.5, 39.8, 35.2, 31.9, 26.4, 26.4, 26.0, 23.1, 14.1; HRMS (ASAP) Found MH^{+} 280.1728, $C_{16}H_{26}ONS$ requires 280.1730.

6-Fluoro-*N*-methylbicyclo[2.2.1]heptane-2-carboxamide (45)

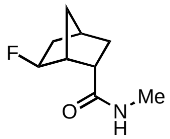

Following **GP6**, **1t** (25 mg, 0.1 mmol) gave **45** (71%) as an oil. dr 10:1. FT-IR ν_{max} (film)/cm^{-1} 1711, 1418, 1360, 1219, 736; ^{1}H NMR (500 MHz, $CDCl_{3,}$ major isomer) δ 5.57 (1H, br s), 4.81 (1H, dd, *J* = 55.1, 6.3 Hz), 2.81 (3H, d, *J* = 4.9 Hz), 2.66–2.51 (2H, m), 2.39–2.32 (1H, m), 1.89 (1H, dddd, *J* = 20.2, 14.1, 6.5, 2.5 Hz), 1.73 (1H, d, *J* = 10.1 Hz), 1.69–1.64 (1H, m), 1.60–1.56 (2H, m, 1.33 (1H, d, *J* = 10.1 Hz); ^{13}C NMR (126 MHz, $CDCl_3$, major isomer) δ 173.0, 93.1 (d, *J* = 178.5 Hz), 46.3 (d, *J* = 21.1 Hz), 42.8 (d, *J* = 9.9 Hz), 39.5 (d, *J* = 19.3 Hz), 36.8, 35.8, 30.5, 26.3; ^{19}F NMR ($CDCl_3$, 376 MHz) δ –167.7^{M}, –177.1^{m}; HRMS (APCI) Found MH^{+} 172.1132, $C_9H_{15}ONF_2$ requires 172.1129.

6-Chloro-*N*-methylbicyclo[2.2.1]heptane-2-carboxamide (46)

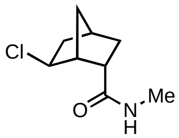

Following **GP6**, **1t** (26 mg, 0.1 mmol) gave **46** (45%) as an oil. dr 10:1. FT-IR ν_{max} (film)/cm^{-1} 1738, 1365, 1265, 1217, 732, 703; ^{1}H NMR (500 MHz, $CDCl_3$, major isomer) δ 5.49 (1H, s), 4.19 (1H, d, *J* = 7.5 Hz), 2.83 (3H, d, *J* = 4.8 Hz), 2.65 (1H, p, *J* = 5.1 Hz), 2.58 (1H, d, *J* = 4.6 Hz), 2.40–2.35 (1H, m), 2.13 (1H, ddd, *J* = 13.9, 7.5, 2.3 Hz), 1.95 (1H, dd, *J* = 10.2, 1.9 Hz), 1.86–1.79 (1H, m), 1.67–1.57 (2H, m), 1.41 (1H, d, *J* = 10.2 Hz); ^{13}C NMR (126 MHz, $CDCl_3$, major isomer) δ 173.5, 58.9, 50.6, 45.9, 43.5, 37.9, 37.8, 30.9, 26.9; HRMS (APCI) Found [M+] 187.0758, $C_9H_{14}NClO$ requires 187.0764.

***N*-Methyl-6-(phenylthio)bicyclo[2.2.1]heptane-2-carboxamide (47)**

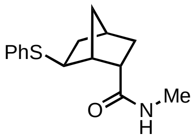

Following **GP6**, **1t** (26 mg, 0.1 mmol) gave **47** (49%) as an oil. FT-IR ν_{max} (film)/cm^{-1} 1739, 1365, 1264, 1217, 732, 702; ^{1}H NMR (500 MHz, $CDCl_3$) δ 7.25 (2H, d, *J* = 7.5 Hz), 7.19 (2H, t, *J*=7.2 Hz), 7.09 (1H, t, *J* = 7.2 Hz), 5.24 (1H, br s), 3.48–3.37 (1H, m), 2.72 (3H, d, *J* = 4.8 Hz), 2.63–2.56 (1H, m), 2.34–2.27 (2H, m), 1.89 (1H, ddd, *J* = 13.2, 8.4, 2.5 Hz), 1.82–1.73 (1H, m), 1.70–1.55 (4H, m), 1.48–1.42 (1H, m); ^{13}C NMR (126 MHz, $CDCl_3$) δ 173.7, 137.5, 129.5, 128.9, 126.0, 46.8, 46.7, 43.3, 38.0, 37.8, 37.4, 30.6, 26.5; HRMS (ESI) Found MNa^+ 284.1069, $C_{15}H_{19}NOSNa$ requires 284.1080.

6-Cyano-*N*-methylbicyclo[2.2.1]heptane-2-carboxamide (48)

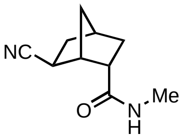

Following **GP6**, **1t** (26 mg, 0.1 mmol) gave **48** (41%) as an oil. FT-IR ν_{max} (film)/cm^{-1} 1739, 1440, 1374, 1228, 1217, 1037, 917, 737, 667; ^{1}H NMR (500 MHz, $CDCl_3$) δ 5.48 (1H, s), 2.97 (1H, ddd, *J* = 9.2, 4.8, 1.5 Hz), 2.83 (3H, d, *J* = 4.8 Hz), 2.70 (1H, d, *J* = 4.3 Hz), 2.59 (1H, dt, *J* = 11.1, 4.8 Hz), 2.46–2.41 (1H, m), 1.93–1.75 (4H, m), 1.74–1.66 (2H, m); ^{13}C NMR (126 MHz, $CDCl_3$) δ 173.3, 123.7, 45.9, 45.9, 39.5, 37.0, 35.8, 31.3, 26.6, 26.3; HRMS (ESI) Found MK^+ 217.0730, $C_{10}H_{14}N_2IOK$ requires 217.0738.

N-Methyl-6-(Phenylethynyl)bicyclo[2.2.1]heptane-2-carboxamide (49)

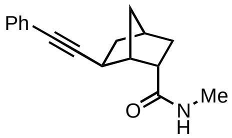

Following **GP6**, **1t** (26 mg, 0.1 mmol) gave **49** (44%) as an oil. dr 3:1. FT-IR ν_{max} (film)/cm^{-1} 1711, 1362, 1262, 1221, 1091, 804, 733, 702; ^{1}H NMR (500 MHz, $CDCl_3$, major isomer) δ 7.39–7.33 (2H, m), 7.25 (3H, d, *J* = 5.9 Hz), 5.48 (1H, s), 2.85 (3H, d, *J* = 4.9 Hz), 2.66–2.60 (1H, m), 2.55 (1H, d, *J* = 4.3 Hz), 2.37 (1H, d, *J* = 3.9 Hz), 1.93–1.80 (2H, m), 1.76–1.49 (3H, m), 1.45 (1H, d, *J* = 9.7, 1.7 Hz); ^{13}C NMR (126 MHz, $CDCl_3$, major isomer) δ 173.9, 131.6, 128.3, 127.6, 124.1, 95.2, 80.5, 47.8, 46.4, 39.1, 39.0, 37.0, 31.0, 28.4, 26.6; HRMS (APCI) Found MH^+ 254.1539, $C_{17}H_{20}NO$ requires 254.1539.

3-(1-Fluorocyclopentyl)-N-methylpropanamide (50)

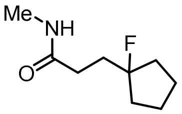

Following **GP6**, **1u** (26 mg, 0.1 mmol) gave **50** (67%) as an oil. FT-IR ν_{max} (film)/cm^{-1} 3330, 2981, 1656, 1395; ^{1}H NMR (400 MHz, $CDCl_3$) δ 5.41 (1H, br s), 2.75 (3H, d, *J* = 4.9 Hz), 2.33–2.22 (2H, m), 2.01 (2H, dt, *J* = 22.3, 8.1 Hz), 1.92–1.78 (2H, m), 1.76–1.68 (2H, m), 1.63–1.55 (2H, m), 1.44–1.35 (2H, m); ^{13}C NMR (126 MHz, $CDCl_3$) δ 173.3, 106.6 (d, *J* = 173.0 Hz), 37.5 (d, *J* = 23.8 Hz), 34.2 (d, *J* = 24.0 Hz), 31.7 (d, *J* = 3.0 Hz), 26.4, 23.9; ^{19}F NMR (471 MHz, $CDCl_3$) δ –144.8; HRMS (ESI) Found MK^+ 212.0844, $C_9H_{16}ONFK$ requires 212.0848.

3-(1-Chlorocyclopentyl)-N-methylpropanamide (51)

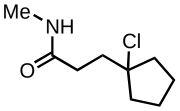

Following **GP6**, **1u** (26 mg, 0.1 mmol) gave **51** (50%) as an oil. FT-IR ν_{max} (film)/cm^{-1} 3295, 2993, 1715, 1660, 959; ^{1}H NMR (400 MHz, $CDCl_3$) δ 5.53 (1H, s), 2.81 (3H, d, *J* = 4.8 Hz), 2.53–2.40 (2H, m), 2.24–2.16 (2H, m), 1.99–1.84 (2H, m), 1.80–1.72 (4H, m), 1.60–1.51 (2H, m); ^{13}C NMR (126 MHz, $CDCl_3$) δ 177.1, 60.6, 39.0, 32.6, 26.5, 23.2, 22.8; HRMS (ASAP) Found MH^+ 190.0995, $C_9H_{17}ONCl$ requires 190.0993.

N-Methyl-3-(1-(phenylthio)cyclopentyl)propanamide (52)

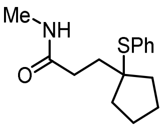

Following **GP6**, **1u** (26 mg, 0.1 mmol) gave **52** (73%) as an oil. FT-IR ν_{max} (film)/cm^{-1} 3302, 2926, 2854, 2239, 1644, 1557, 1449, 907, 731; ^{1}H NMR (500 MHz, $CDCl_3$) δ 7.48 (2H, dd, *J* = 7.4, 2.0 Hz), 7.37–7.30 (3H, m), 5.44 (1H, br s), 2.81 (3H, d, *J* = 4.9 Hz), 2.58–2.48 (2H, m), 1.92–1.88 (2H, m), 1.88–1.84 (2H, m), 1.78–1.71 (2H, m), 1.71–1.64 (2H, m), 1.55–1.48 (2H, m); ^{13}C NMR (126 MHz, $CDCl_3$) δ 173.6, 136.3, 133.0, 128.7, 128.5, 61.2, 38.5, 35.3, 33.2, 26.4, 23.8; HRMS (ASAP) Found MH$^+$ 264.1412, $C_{15}H_{22}ONS$ requires 264.1417.

N-Methyl-3-(1-(phenylethynyl)cyclopentyl)propanamide (53)

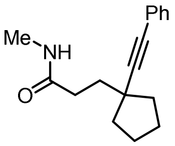

Following **GP6**, **1u** (26 mg, 0.1 mmol) gave **53** (76%) as an oil. FT-IR ν_{max} (film)/cm^{-1} 3302, 2956, 2924, 2855, 1644, 1559, 1490, 907; ^{1}H NMR (400 MHz, $CDCl_3$) δ 7.40–7.33 (2H, m), 7.31–7.23 (3H, m), 5.48 (1H, br s), 2.80 (3H, d, *J* = 4.9 Hz), 2.49–2.40 (2H, m), 2.01–1.92 (2H, m), 1.96–1.87 (2H, m), 1.89–1.79 (2H, m), 1.75–1.64 (2H, m), 1.64–1.54 (2H, m); ^{13}C NMR (126 MHz, $CDCl_3$) δ 173.8, 131.7, 128.3, 127.7, 124.0, 96.2, 81.8, 43.0, 40.2, 36.5, 34.2, 26.5, 24.4; HRMS (ASAP) Found MH$^+$ 256.1694, $C_{17}H_{22}ON$ requires 256.1696.

***tert*-Butyl 4-Fluoro-4-(3-(methylamino)-3-oxopropyl)piperidine-1-carboxylate (54)**

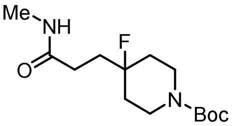

Following **GP6**, **1v** (37 mg, 0.1 mmol) gave **54** (59%) as an oil. FT-IR ν_{max} (film)/cm^{-1}; 2924, 2854, 1662, 1418, 1156, 909, 757, 734; ^{1}H NMR (500 MHz, $CDCl_3$) δ 5.45 (1H, s), 3.91 (2H, br.s), 3.06 (2H, br.s), 2.82 (3H, d, *J* = 4.8 Hz), 2.33–2.26 (2H, m), 2.03–1.92 (2H, m), 1.82–1.74 (2H, m), 1.45 (9H, s); ^{19}F NMR (376 MHz, $CDCl_3$) δ -164.60; ^{13}C NMR (126 MHz, $CDCl_3$) δ 172.9, 154.8, 93.7 (d, *J* = 172.1 Hz), 79.8, 35.6 (d, *J* = 22.0 Hz), 34.7, 29.8 (d, *J* = 4.1 Hz), 28.6, 26.6; HRMS (ESI) Found MK$^+$ 327.1471, $C_{14}H_{25}O_3N_2FK$ requires 327.1481.

tert-Butyl 4-(3-(Methylamino)-3-oxopropyl)-4-(phenylethynyl)piperidine-1-carboxylate (55)

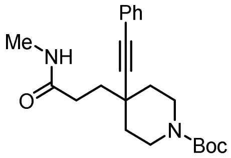

Following **GP6**, **1v** (37 mg, 0.1 mmol) gave **55** (79%) as an oil. FT-IR ν_{max} (film)/cm^{-1} 3322, 2926, 1674, 1424, 1279, 1170, 1148, 906, 730; ^{1}H NMR (400 MHz, $CDCl_3$) δ 7.44–7.35 (2H, m), 7.34–7.26 (3H, m), 5.45 (1H, br s), 4.04 (2H, br s), 3.18–3.06 (2H, m), 2.81 (3H, d, *J* = 4.8 Hz), 2.47–2.39 (2H, m), 1.91–1.84 (2H, m), 1.79–1.71 (2H, m), 1.48–1.38 (2H, m), 1.46 (9H, s); ^{13}C NMR (126 MHz, $CDCl_3$) δ 173.4, 154.9, 131.8, 128.4, 128.2, 123.3, 91.9, 85.5, 79.7, 38.0, 37.0, 36.0, 32.0, 31.1, 28.6, 26.6; HRMS (ASAP) Found MH^{+} 371.2325, $C_{22}H_{31}O_3N_2$ requires 371.2329.

tert-Butyl 4-(3-((tert-Butoxycarbonyl)amino)propyl)-4-fluoropiperidine-1-carboxylate (56)

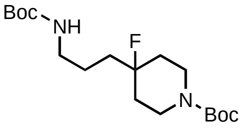

Following **GP6**, **1w** (44 mg, 0.1 mmol) gave **56** (58%) as an oil. FT-IR ν_{max} (film)/cm^{-1} 1022; ^{1}H NMR (500 MHz, $CDCl_3$, rotamers) δ 4.55 (1H, br s), 4.00–3.73 (2H, m), 3.66–3.25 (1H, m), 3.21–2.94 (3H, m), 1.79 (2H, br t, *J* = 11.9 Hz), 1.72–1.49 (6H, m), 1.45 (9H, s), 1.44 (9H, s); ^{13}C NMR (126 MHz, $CDCl_3$) δ 156.3, 155.1, 132.5 (d, *J* = 10.1 Hz), 129.0 (d, *J* = 21.5 Hz), 128.8, 94.1 (d, *J* = 171.3 Hz), 80.0, 41.0, 39.7 (br s), 37.7 (d, *J* = 22.5 Hz), 34.9 (d, *J* = 22.0 Hz), 28.8, 28.8, 23.9 (d, *J* = 3.7 Hz); ^{19}F NMR ($CDCl_3$, 376 MHz, rotamers) δ –161.8^{M}, –163.0^{m}; HRMS (APCI) Found MH^{+} 399.2056, $C_{18}H_{33}N_2O_4FK$ requires 399.2044.

tert-Butyl 4-(3-((tert-Butoxycarbonyl)amino)propyl)-4-chloropiperidine-1-carboxylate (57)

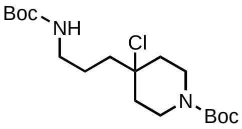

Following **GP6**, **1w**(44 mg, 0.1 mmol) gave **57** (44%) as an oil. FT-IR ν_{max} (film)/cm^{-1} 1709, 1359, 1220; ^{1}H NMR (500 MHz, $CDCl_3$) δ 4.54 (1H, br s), 3.97 (2H, br s), 3.28–3.01 (4H, m), 2.01–1.83 (2H, m), 1.80–1.68 (4H, m), 1.67–1.58 (2H, m), 1.45 (9H, s), 1.44 (9H, s); ^{13}C

NMR (126 MHz, $CDCl_3$) δ 156.4, 155.0, 80.1, 79.7, 73.2, 42.9, 40.9, 39.1, 30.1, 28.8, 28.8, 24.8, 23.1; HRMS (APCI) Found MH^+ 377.2202, $C_{18}H_{34}N_2O_4Cl$ requires 377.2189.

6-Bromo-4-fluoro-*N*-methylhexanamide (58)

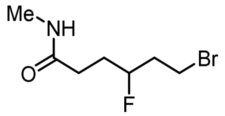

Following **GP6**, **1l** (31 mg, 0.1 mmol) gave **58** (42%) as an oil. FT-IR ν_{max} (film)/cm^{-1} 3304, 2935, 1645, 1557, 1412, 906, 729; ^{1}H NMR (500 MHz, $CDCl_3$) δ 5.48 (1H, br s), 4.82–4.59 (1H, m), 3.49 (2H, dd, *J* = 8.0, 5.4 Hz), 2.82 (3H, d, *J* = 4.8 Hz), 2.41–2.26 (2H, m), 2.26–2.18 (1H, m), 2.13–1.85 (3H, m); ^{13}C NMR (126 MHz, $CDCl_3$) δ 172.5, 91.5 (d, *J* = 169.4 Hz), 38.4 (d, *J* = 21.0 Hz), 31.9 (d, *J* = 3.5 Hz), 30.7 (d, *J* = 20.4 Hz), 28.5 (d, *J* = 4.6 Hz), 26.5; ^{19}F NMR (376 MHz, $CDCl_3$) δ –187.3; HRMS (ASAP) Found MH^+ 226.0239, $C_7H_{14}ONBrF$ requires 226.0237.

6-Bromo-4-chloro-*N*-methylhexanamide (59)

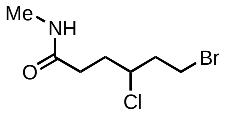

Following **GP6**, **1l** (31 mg, 0.1 mmol) gave **59** (58%) as an oil. FT-IR ν_{max} (film)/cm^{-1} 3153, 3077, 1771, 1699, 1372, 1187, 850; ^{1}H NMR (400 MHz, $CDCl_3$) δ 5.51 (1H, br s), 4.18–4.06 (2H, m), 3.56 (2H, dd, *J* = 7.3, 5.7 Hz), 2.82 (3H, d, *J* = 4.9 Hz), 2.50–2.31 (2H, m), 2.28–2.15 (3H, m); ^{13}C NMR (126 MHz, $CDCl_3$) δ 172.2, 60.4, 41.1, 33.7, 33.1, 29.8, 26.4; HRMS (ASAP) Found MH^+ 241.9941, $C_7H_{14}ONBrCl$ requires 241.9942.

4-(2-Bromoethyl)-*N*-methyl-6-phenylhex-5-ynamide (60)

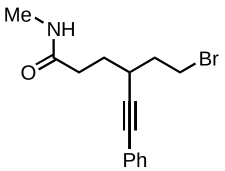

Following **GP6**, **1l** (31 mg, 0.1 mmol) gave **60** (58%) as an oil. FT-IR ν_{max} (film)/cm^{-1} 3320, 2932, 1645, 1557, 908; ^{1}H NMR (400 MHz, $CDCl_3$) δ 7.41–7.37 (2H, m), 7.32–7.28 (3H, m), 5.49 (1H, br s), 3.69–3.50 (2H, m), 2.82 (3H, d, *J* = 4.8 Hz), 2.54–2.44 (1H, m), 2.42–2.31 (1H, m), 2.12–2.04 (2H, m), 2.03–1.94 (1H, m), 1.91–1.64 (2H, m); ^{13}C NMR (126 MHz, $CDCl_3$) δ 173.0, 131.8, 128.4, 128.2, 123.3, 90.3, 83.7, 38.2, 34.5, 31.4, 31.1, 30.6, 26.5; HRMS (ASAP) Found MH^+ 308.0634, $C_{15}H_{19}ONBr$ requires 308.0645.

(*R*)-4-Fluoro-4-methyl-1-(methylamino)-1-oxopentan-2-yl Acetate (61)

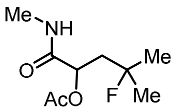

Following **GP6**, **1x** (29 mg, 0.1 mmol) gave **61** (89%) as an oil. FT-IR ν_{max} (film)/cm^{-1} 3326, 2928, 1738, 1667, 1543, 1217, 754; ^{1}H NMR (500 MHz, $CDCl_3$) δ 6.08 (1H, br s), 5.41 (1H, dd, *J* = 8.8, 3.1 Hz), 2.84 (3H, d, *J* = 4.8 Hz), 2.27 (1H, ddd, *J* = 23.4, 15.4, 3.2 Hz), 2.18–2.09 (1H, m), 2.14 (3H, s), 1.42 (3H, d, *J* = 11.7 Hz), 1.37 (3H, d, *J* = 11.8 Hz); ^{13}C NMR (126 MHz, $CDCl_3$) δ 170.2, 169.8, 94.3 (d, *J* = 167.3 Hz), 70.5 (d, *J* = 3.0 Hz), 42.3 (d, *J* = 21.9 Hz), 27.4 (d, *J* = 24.5 Hz), 26.7 (d, *J* = 24.5 Hz), 26.2, 21.1; ^{19}F NMR (376 MHz, $CDCl_3$) δ –139.5; HRMS (ASAP) Found MH$^+$ 206.1188, $C_9H_{17}O_3NF$ requires 206.1187.

(*R*)-2-(1,3-Dioxoisoindolin-2-yl)-4-fluoro-*N*,4-dimethylpentanamide (62)

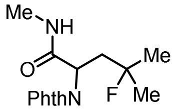

Following **GP6**, **1y** (38 mg, 0.1 mmol) gave **62** (37%) as an oil. FT-IR ν_{max} (film)/cm^{-1} 2622, 2852, 1652, 1466, 1329, 1142, 907; ^{1}H NMR (400 MHz, $CDCl_3$) δ 8.01 (1H, dd, *J* = 7.8, 1.3 Hz), 7.59 (1H, td, *J* = 7.5, 1.4 Hz), 7.51 (1H, td, *J* = 7.7, 1.4 Hz), 7.45 (1H, dd, *J* = 7.5, 1.4 Hz), 4.84–4.79 (1H, m), 2.85 (3H, d, *J* = 4.8 Hz), 2.49–2.36 (1H, m), 2.19–2.11 (1H, m), 1.47 (3H, d, *J* = 21.7 Hz), 1.45 (3H, d, *J* = 21.9 Hz); ^{13}C NMR (101 MHz, $CDCl_3$) δ 171.9, 170.4, 137.8, 136.0, 132.4, 130.9, 129.8, 127.2, 96.1 (d, *J* = 164.6 Hz), 51.2, 41.2 (d, *J* = 20.5 Hz), 28.0 (d, *J* = 24.3 Hz), 26.6; ^{19}F NMR (376 MHz, $CDCl_3$) δ –135.8; HRMS (ESI) Found MNa$^+$ 315.1107, $C_{15}H_{17}O_3N_2FNa$ requires 315.1115.

***tert*-Butyl (*R*)-(4-fluoro-4-methyl-1-(methylamino)-1-oxopentan-2-yl)(methyl)carbamate (63)**

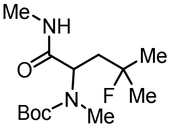

Following **GP6**, **1z** (36 mg, 0.1 mmol) gave **63** (40%) as an oil. FT-IR ν_{max} (film)/cm^{-1} 3346, 2976, 2932, 1668, 1367, 1156; ^{1}H NMR (400 MHz, $CDCl_3$, rotamers) δ 6.20 (0.6H, br s), 6.00 (0.4H, br s), 4.90 (1H, dd, *J* = 8.5, 4.8 Hz), 2.86–2.78 (3H, m), 2.77 (1.2H, s), 2.73 (1.8H, s), 2.42–2.26 (1H, m), 2.08–1.93 (1H, m), 1.47 (9H, s), 1.39 (3.6H, d, *J* = 21.4 Hz), 1.37 (2.4H, d, *J* = 21.6 Hz); ^{13}C NMR (126 MHz, $CDCl_3$, rotamers) δ 171.5, 156.6, 94.6 (d, *J* = 166.4 Hz), 80.6, 56.7, 54.7, 37.9 (d, *J* = 21.2 Hz), 29.7, 28.4, 27.9 (d, *J* = 25.0 Hz), 26.3;

^{19}F NMR (376 MHz, $CDCl_3$, rotamers) δ –140.72^{M}, –142.30^{m}; HRMS (ESI) Found MNa^+ 299.1734, $C_{16}H_{25}O_3N_2FNa$ requires 299.1741.

Benzyl (*S*)-(4-Fluoro-4-methyl-1-(methylamino)-1-oxopentan-2-yl)carbamate (64)

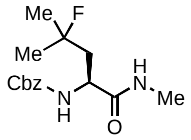

Following **GP6**, **1aa** (38 mg, 0.1 mmol) gave **64** (56%) as a solid. FT-IR ν_{max} (film)/cm^{-1} 2360, 2341, 1653, 1539; ^1H NMR (400 MHz, $CDCl_3$) δ 7.38–7.30 (5H, m), 6.29 (1H, br s), 5.44 (1H, br s), 5.12 (2H, s), 4.32 (1H, q, J=7.8 Hz), 2.80 (3H, d, *J* = 4.7 Hz), 2.24 (1H, ddd, *J* = 25.7, 15.2, 4.6 Hz), 2.02–1.93 (1H, m), 1.42 (3H, d, *J* = 21.7 Hz), 1.39 (3H, d, *J* = 21.7 Hz); ^{13}C NMR (126 MHz, $CDCl_3$) δ 172.2, 171.3, 136.2, 128.7, 128.4, 128.2, 96.0 (d, *J* = 164.1 Hz), 67.4, 52.5, 42.5 (d, *J* = 20.2 Hz), 27.8 (d, *J* = 24.2 Hz), 26.5, 26.3 (d, *J* = 24.7 Hz); ^{19}F NMR (376 MHz, $CDCl_3$) δ –136.7; HRMS (APCI) Found MH^+ 297.1597, $C_{15}H_{22}O_3N_2F$ requires 297.1609.

Benzyl (*S*)-(4,4-Dimethyl-1-(methylamino)-1-oxo-6-phenylhex-5-yn-2-yl)carbamate (65)

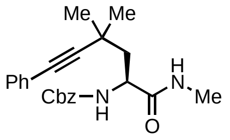

Following **GP6**, **1aa** (38 mg, 0.1 mmol) gave **65** (47%) as a solid. FT-IR ν_{max} (film)/cm^{-1} 2360, 2341, 2253, 1700, 1653, 1521, 1456, 1375, 1260, 1040, 918, 805, 761, 695; ^1H NMR (500 MHz, $CDCl_3$) δ 7.35–7.19 (10H, m), 6.32 (1H, br s), 5.73 (1H, br s), 5.11–4.99 (2H, m), 4.41–4.36 (1H, m), 2.79 (3H, d, *J* = 4.3 Hz), 2.10 (1H, dd, *J* = 14.5, 4.2 Hz), 1.91–1.86 (1H, m), 1.36 (3H, s), 1.33 (3H, s); ^{13}C NMR (126 MHz, $CDCl_3$) δ 171.5, 155.3, 134.9, 130.5, 127.5, 127.2, 127.2, 127.1, 126.9, 122.0, 95.0, 81.0, 66.2, 52.7, 43.0, 29.9, 29.2, 27.2, 25.3; HRMS (ESI) Found MH^+ 379.2002, $C_{23}H_{27}O_3N_2$ requires 379.2016.

Benzyl ((2*S*,3*R*)-4-Fluoro-3-methyl-1-(methylamino)-1-oxopentan-2-yl)carbamate (67)

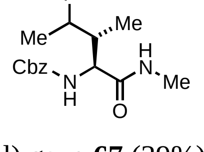

Following **GP6**, **1ad** (36 mg, 0.1 mmol) gave **67** (39%) as an oil. dr 3:2.

The two diastereomers were separated by column chromatography on silica gel.

Data for first isomer: FT-IR ν_{max} (film)/cm^{-1} 2253, 1739, 1436, 1374, 1037, 917, 737; ^1H NMR (500 MHz, $CDCl_3$) δ 7.39–7.29 (5H, m), 5.98 (1H, s), 5.67–5.59 (1H, m), 5.17– 5.08

(2H, m), 4.62–4.44 (1H, m), 4.41–4.30 (1H, m), 2.83 (3H, d, *J* = 5.0 Hz), 2.41–2.30 (1H, m), 1.39 (3H, dd, *J* = 25.5, 6.2 Hz), 0.91 (3H, d, *J* = 7.1 Hz); ^{13}C NMR (126 MHz, $CDCl_3$) δ 170.3, 155.9, 136.3, 128.7, 128.4, 128.2, 93.0 (d, *J* = 166.3 Hz), 67.2, 55.7, 41.7 (d, *J* = 18.2 Hz), 31.1, 26.4, 19.18 (d, *J* = 23.4 Hz); ^{19}F NMR ($CDCl_3$, 376 MHz) δ –173.0; HRMS (APCI) Found MH^+ 297.1609, $C_{15}H_{22}N_2O_3F$ requires 297.1601.

Data for second isomer: ^{1}H NMR (500 MHz, $CDCl_3$) δ 7.43–7.30 (5H, m), 6.34 (1H, s), 5.55 (1H, s), 5.17– 5.08 (2H, m), 4.99–4.82 (1H, m), 4.30–4.23 (1H, m), 2.83 (3H, d, *J* = 5.0 Hz), 2.45–2.36 (1H, m), 1.31 (3H, dd, *J* = 25.1, 6.4 Hz), 1.02 (3H, d, *J* = 7.1 Hz);^{13}C NMR (126 MHz, $CDCl_3$) δ 171.4, 156.9, 136.1, 128.8, 128.5, 128.3, 92.05 (d, *J* = 164.6 Hz), 67.5, 59.5, 38.9 (d, *J* = 18.0 Hz), 29.9, 26.4, 18.81 (d, *J* = 22.8 Hz); ^{19}F NMR ($CDCl_3$, 376 MHz) δ –186.3; HRMS (APCI) Found MH^+ 297.1608, $C_{15}H_{22}N_2O_3F$ requires 297.1601.

Benzyl ((2*S*,3*S*)-3,4-Dimethyl-1-(methylamino)-1-oxo-6-phenylhex-5-yn-2-yl)carbamate (68)

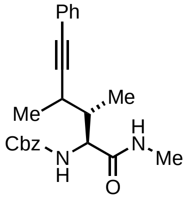

Following **GP6**, **1ad** (36 mg, 0.1 mmol) gave **68** (54%) as a solid. dr 2:1.

The two diastereomers were separated by column chromatography on silica gel.

Data for first isomer: FT-IR ν_{max} (film)/cm^{-1} 2253, 1712, 1365, 1223, 1033, 736; ^{1}H NMR (500 MHz, $CDCl_3$) δ 7.49–7.39 (2H, m), 7.38–7.31 (5H, m), 7.31–7.26 (3H, m), 6.15 (1H, br s), 5.76 (1H, d, *J* = 8.6 Hz), 5.11 (2H, s), 4.47 (1H, dd, *J* = 8.6, 6.1 Hz), 2.81 (3H, d, *J* = 4.8 Hz), 2.64 (1H, q, *J* = 7.1 Hz), 2.20–2.08 (1H, m), 1.28 (3H, d, *J* = 6.9 Hz), 1.01 (3H, d, *J* = 6.9 Hz); ^{13}C NMR (126 MHz, $CDCl_3$) δ 171.1, 156.3, 136.6, 132.0, 128.9, 128.7, 128.6, 128.4, 123.5, 92.8, 84.0, 67.4, 57.9, 40.9, 29.6, 26.7, 18.2, 13.7; HRMS (APCI) Found MH^+ 379.2016, $C_{23}H_{27}N_2O_3$ requires 379.2003.

Data for second isomer: FT-IR ν_{max} (film)/cm^{-1} 2253, 1712, 1365, 1223, 1033, 736; ^{1}H NMR (500 MHz, $CDCl_3$) δ 7.40–7.26 (9H, m), 7.18 (1H, t, *J* = 7.6 Hz), 6.31 (1H, s), 6.17 (1H, d, *J* = 8.9 Hz), 5.22–4.97 (2H, m), 4.26 (1H, dd, *J* = 8.9, 5.9 Hz), 2.95–2.89 (1H, m), 2.84 (3H, d, *J* = 4.9 Hz), 2.40–2.26 (1H, m). 1.26 (3H, d, *J* = 7.1 Hz), 1.11 (3H, d, *J* = 6.9 Hz); ^{13}C NMR (126 MHz, $CDCl_3$) δ 171.9, 156.9, 136.2, 131.7, 128.8, 128.5, 128.5, 128.3, 128.3, 123.1, 91.7, 84.3, 67.6, 60.9, 38.6, 31.2, 27.3, 26.5, 20.1, 12.0; HRMS (APCI) Found MH^+ 379.2016, $C_{23}H_{27}N_2O_3$ requires 379.2003.

Dibenzyl ((5*S*)-3-Fluoro-6-(methylamino)-6-oxohexane-1,5-diyl)dicarbamate (69)

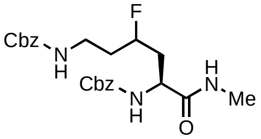

Following **GP6**, **1ae** (53 mg, 0.1 mmol) gave **69** (25%) as a solid. dr 1:1. FT-IR ν_{max} (film)/cm^{-1} 2253, 1739, 1713, 1436, 1365, 1224, 1217, 1038, 917, 737; ^{1}H NMR (500 MHz, DMSO-d_6, 373K) δ 7.42–7.26 (10H, m), 6.98 (2H, br s), 6.87 (1H, br s), 5.10–4.99 (4H, m), 4.73–4.52 (1H, m), 3.72 (2H, q, *J*=5.4 Hz), 4.18– 4.07 (1H, m), 2.64–2.59 (3H, m), 2.05–1.38 (4H, m); ^{13}C NMR (126 MHz, $CDCl_3$, diasteremers and rotamers) δ 171.7, 171.4, 156.7, 156.6, 156.5, 156.3, 136.6, 136.5, 136.1, 136.1, 128.7, 128.7, 128.7, 128.5, 128.4, 128.3, 128.3, 128.3, 128.2, 90.6 (d, *J* = 167.3 Hz), 67.5, 67.4, 67.0, 66.9, 52.4, 52.3, 37.5, 37.4, 35.7 (d, *J* = 19.8 Hz), 35.3 (d, *J* = 20.0 Hz), 30.5, 29.8, 26.5, 26.4; ^{19}F NMR ($CDCl_3$, 376 MHz, diasteremers and rotamers) δ –181.7, –183.4; HRMS (ESI) Found MK$^+$ 484.1628, $C_{23}H_{28}N_3O_5FK$ requires 484.1645.

Benzyl (*S*)-(2-((4-Fluoro-4-methyl-1-(methylamino)-1-oxopentan-2-yl)amino)-2-oxoethyl)carbamate (70)

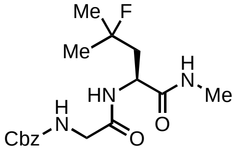

Following **GP6**, **1ac** (44 mg, 0.1 mmol) gave **70** (52%) as a solid. FT-IR ν_{max} (film)/cm^{-1} 3298, 2918, 2852, 2359, 2339, 1713, 1652, 1538, 1258, 1158, 1051; ^{1}H NMR (400 MHz, $CDCl_3$) δ 7.38–7.31 (5H, m), 6.81 (1H, br s), 6.63 (1H, br s), 5.50 (1H, t, *J* = 5.4 Hz), 5.13–5.12 (2H, m), 4.59– 4.54 (1H, m), 3.85 (2H, dd, *J* = 5.2, 3.4 Hz), 2.76 (3H, d, *J* = 4.6 Hz), 2.22 (1H, ddd, *J* = 26.0, 15.0, 3.9 Hz), 2.08–1.97 (1H, m), 1.39 (3H, d, *J* = 21.8 Hz), 1.36 (3H, d, *J* = 21.8 Hz); ^{13}C NMR (126 MHz, $CDCl_3$) δ 171.3, 169.1, 156.9, 135.9, 128.6, 128.4, 128.1, 67.5, 50.6, 44.9, 41.5 (d, *J* = 20.0 Hz), 27.7 (d, *J* = 24.6 Hz), 26.41, 25.9 (d, *J* = 24.4 Hz); ^{19}F NMR (376 MHz, $CDCl_3$) δ –136.0; HRMS (ESI) Found MK$^+$ 392.1377, $C_{17}H_{24}O_4N_3FK$ requires 392.1382.

Benzyl (*S*)-(2-((4,4-Dimethyl-1-(methylamino)-1-oxo-6-phenylhex-5-yn-2-yl)amino)-2-oxoethyl)carbamate (71)

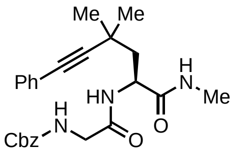

Following **GP6**, **1ac** (44 mg, 0.1 mmol) gave **71** (65%) as a solid. FT-IR ν_{max} (film)/cm^{-1} 2360, 2341, 2254, 1652, 1521, 1436, 1375, 1260, 1039, 919, 805, 762, 697; ^{1}H NMR (400 MHz, $CDCl_3$) δ 7.37–7.27 (10H, m), 6.86 (1H, d, *J* = 7.9 Hz), 6.59 (1H, br s), 5.22 (1H, br s), 5.12–5.04 (2H, m), 4.69–4.64 (1H, m), 3.80–3.64 (2H, m), 2.73 (3H, d, *J* = 4.5 Hz), 2.17–2.12 (1H, m), 1.89–1.80 (1H, m), 1.33 (3H, s), 1.30 (3H, s); ^{13}C NMR (101 MHz, $CDCl_3$) δ 171.9, 168.8, 156.7 (br s), 136.0, 123.1, 81.6, 67.3, 51.5, 44.7, 43.7, 30.6, 30.3, 28.3, 26.4; HRMS (ESI) Found MH$^+$ 436.2233, $C_{25}H_{30}O_4N_3$ requires 436.2231.

5 Picture of Reaction Set-Up

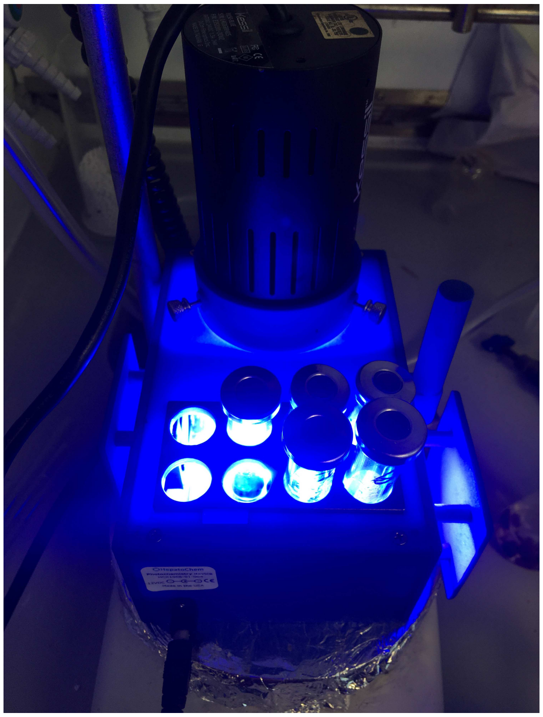

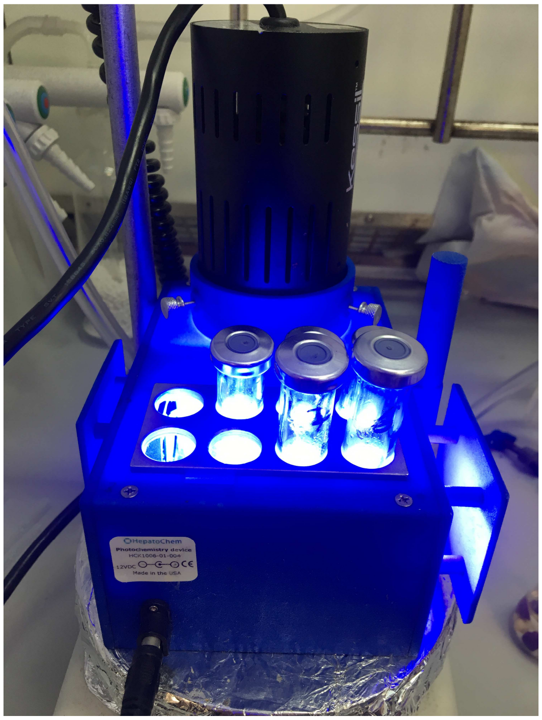

6 Mechanistic Considerations

6.1 Emission Quenching Experiments

Emission intensities were recorded using a Steady State emission spectra were recorded on an Edinburgh Instrument FP920 Phosphorescence Lifetime Spectrometer equipped with a 5 watt microsecond pulsed xenon flash lamp and a 450 watt steady state xenon lamp and a red sensitive photomultiplier in peltier (air cooled) housing, (Hamamatsu R928P) spectrophotometer.

The **2b** solutions were excited at 435 nm and the emission intensity was collected at 473 nm. The **2c** solutions were excited at 435 nm and the emission intensity was collected at 543 nm.

Experimental procedures:

A screw-top quartz cuvette was charged with a 1.6 x 10^{-5} M solution of **2b** in MeCN (2.0 mL) and the initial emission was collected then the appropriate amount of the quencher as a 1.6 x 10^{-2} M solution in MeCN was added. The sample was shaken for 1 min and then the emission of the sample was collected.

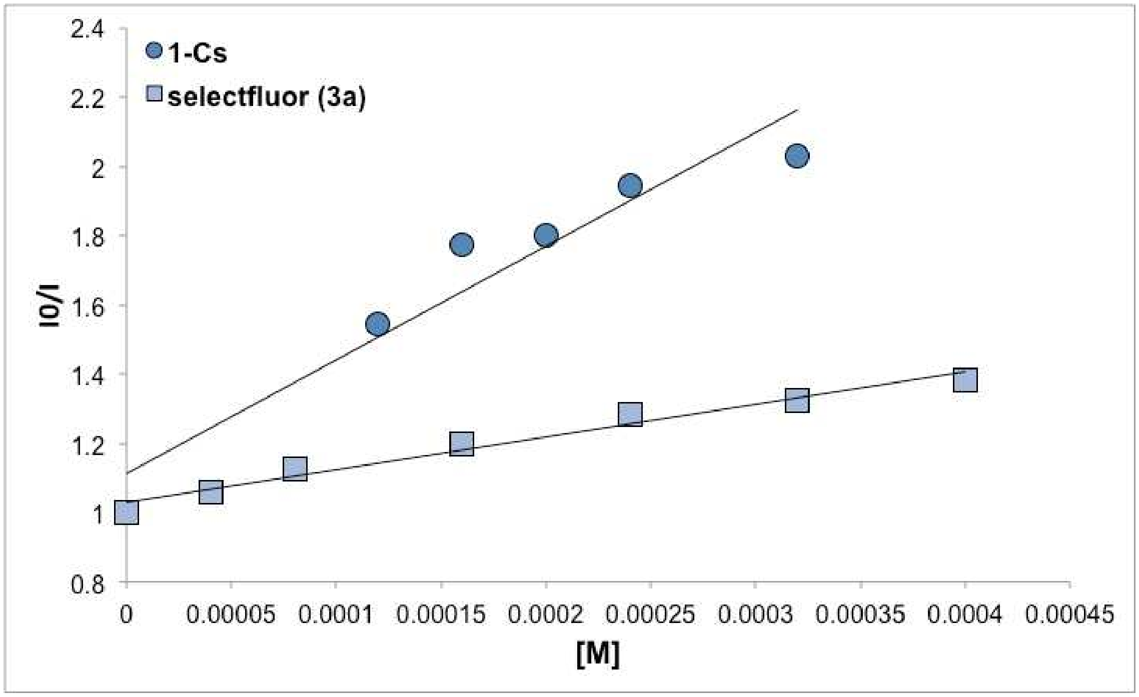

Experimental procedures:

A screw-top quartz cuvette was charged with a 1.6 x 10^{-5} M solution of **2c** in CH_2Cl_2 (2.0 mL) and the initial emission was collected then the appropriate amount of the quencher as a

1.6 x 10^{-2} M solution in CH_2Cl_2 was added. The sample was shaken for 1 min and then the emission of the sample was collected.

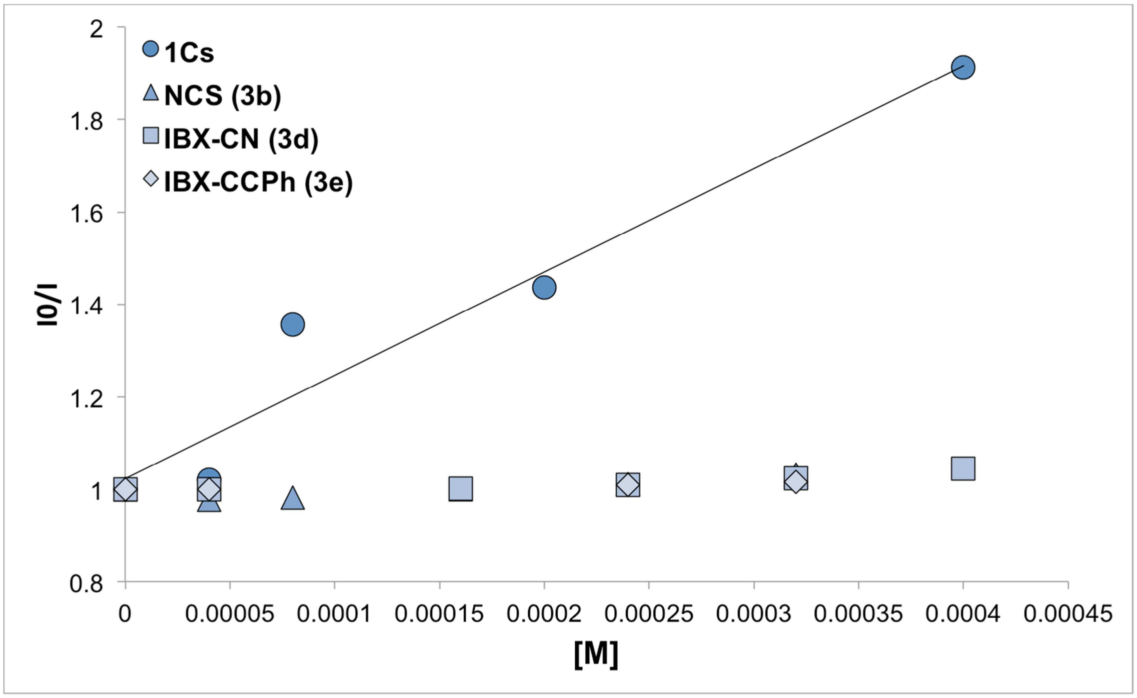

The quenching constants were obtained using the Stern-Volmer relationship:

$$\frac{I_o}{I} = 1 + k_{q\tau_0}[Quencher]$$

Photocatalyst	Quencher	k_q (M^{-1} s^{-1})
**2b**	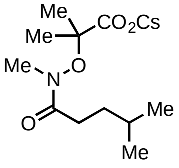 **1a**	1.7 x 10^9
**2b**	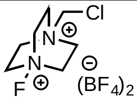	3.3 x 10^3
**2c**	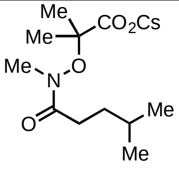 **1a**	4.4 x 10^8

Photocatalyst	Quencher	k_q (M^{-1} s^{-1})
**2c**	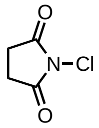	2.6 x 10^7
**2c**	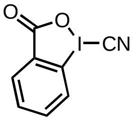	9.6 x 10^6
**2c**	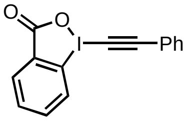	2.1 x 10^6

6.2 Quantum Yield Determination

The quantum yield determination was performed following the procedure reported by Yoon[2] and are the average of two runs.

Reaction	Quantum Yield (Φ)
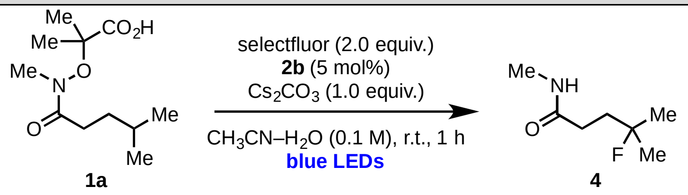	0.08
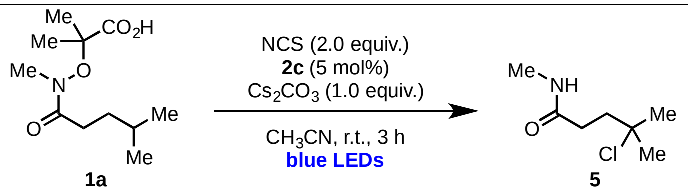	0.06
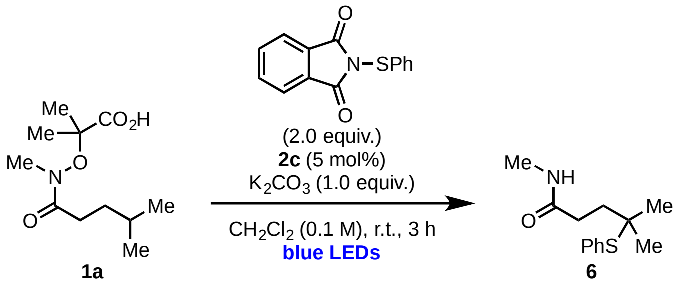	0.02
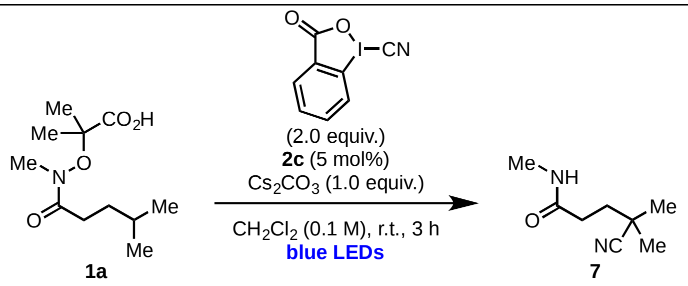	0.02
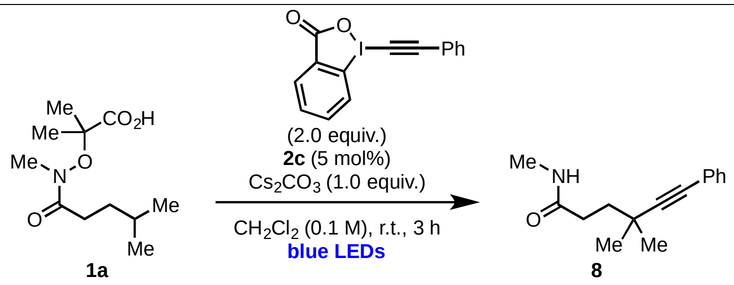	0.06

6.3 1,5-HAT Abstraction: DFT Studies

Correlations between BDEs and reaction parameters

Process	BDE N–H (Kcal mol^{-1})	BDE C–H (Kcal mol^{-1})	δBDE (Kcal mol^{-1})	ΔG^{o} (Kcal mol^{-1})	$\Delta G^{\ddagger}$ (Kcal mol^{-1})
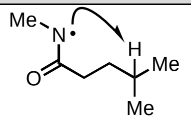	107	95	12	-10.9	8.8
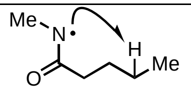	107	98	9	-8.3	11.1
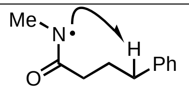	109	90	19	-19.5	9.5
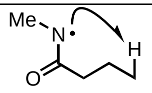	108	102	6	-3.4	13.5
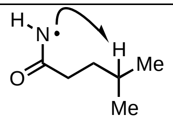	114	95	19	-16.6	5.7
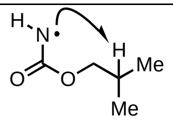	103	95	8	-6.4	14.3

Process	BDE N–H (Kcal mol^{-1})	BDE C–H (Kcal mol^{-1})	δBDE (Kcal mol^{-1})	ΔG° (Kcal mol^{-1})	ΔG‡ (Kcal mol^{-1})
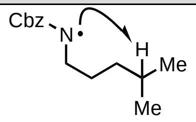	105	97	8	-10.3	8.8
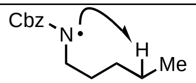	106	97	9	-7.3	11.1
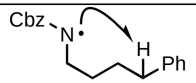	105	87	18	-16.5	10.7
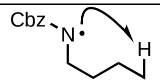	105	101	4	-2.7	15.3
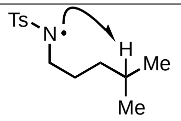	99	94	5	-6.1	9.3
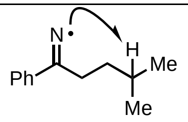	93.2	95.6	-2.4	4.2	13.1
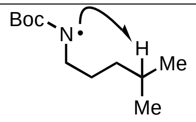	105	97	8	-9.3	9.6

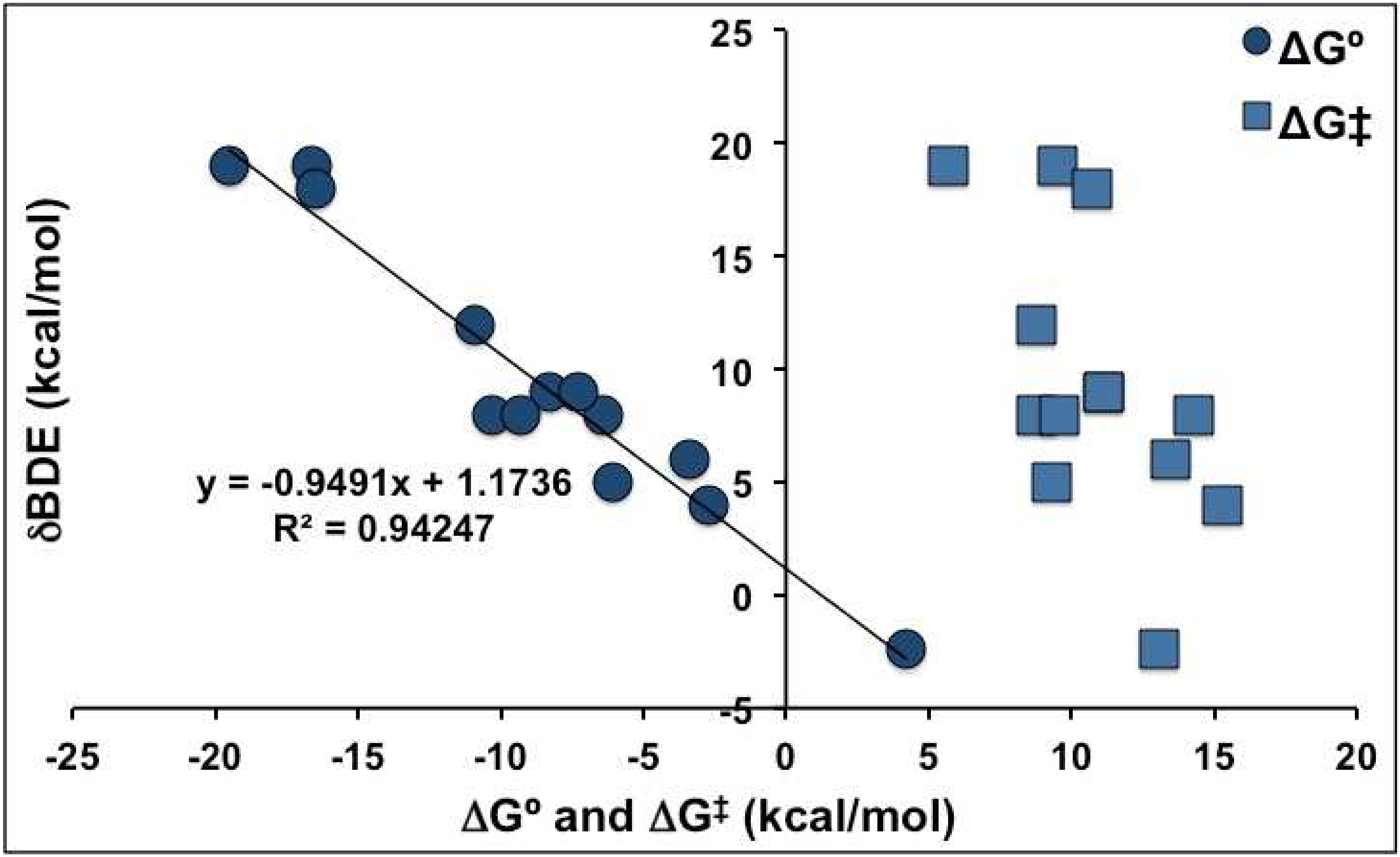

ΔGº
ΔG‡
δBDE (kcal/mol)
y = -0.9491x + 1.1736
R² = 0.94247
25
20
15
10
5
0
-5
-25
-20
-15
-10
-5
0
5
10
15
20
ΔGº and ΔG‡ (kcal/mol)

Correlations between electrophilicity index for nitrogen radicals and reaction parameters

**Process**	**electrophilicity index (eV)**	**ΔG^o (Kcal mol^{-1})**	**$\Delta G^{\ddagger}$ (Kcal mol^{-1})**
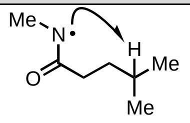	1.09	-10.9	8.8
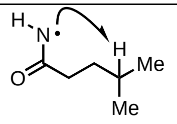	1.38	-16.6	5.7
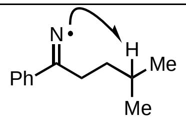	0.77	4.2	13.1
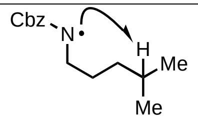	1.03	-9.3	9.6
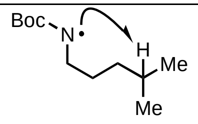	1.05	-10.3	8.8

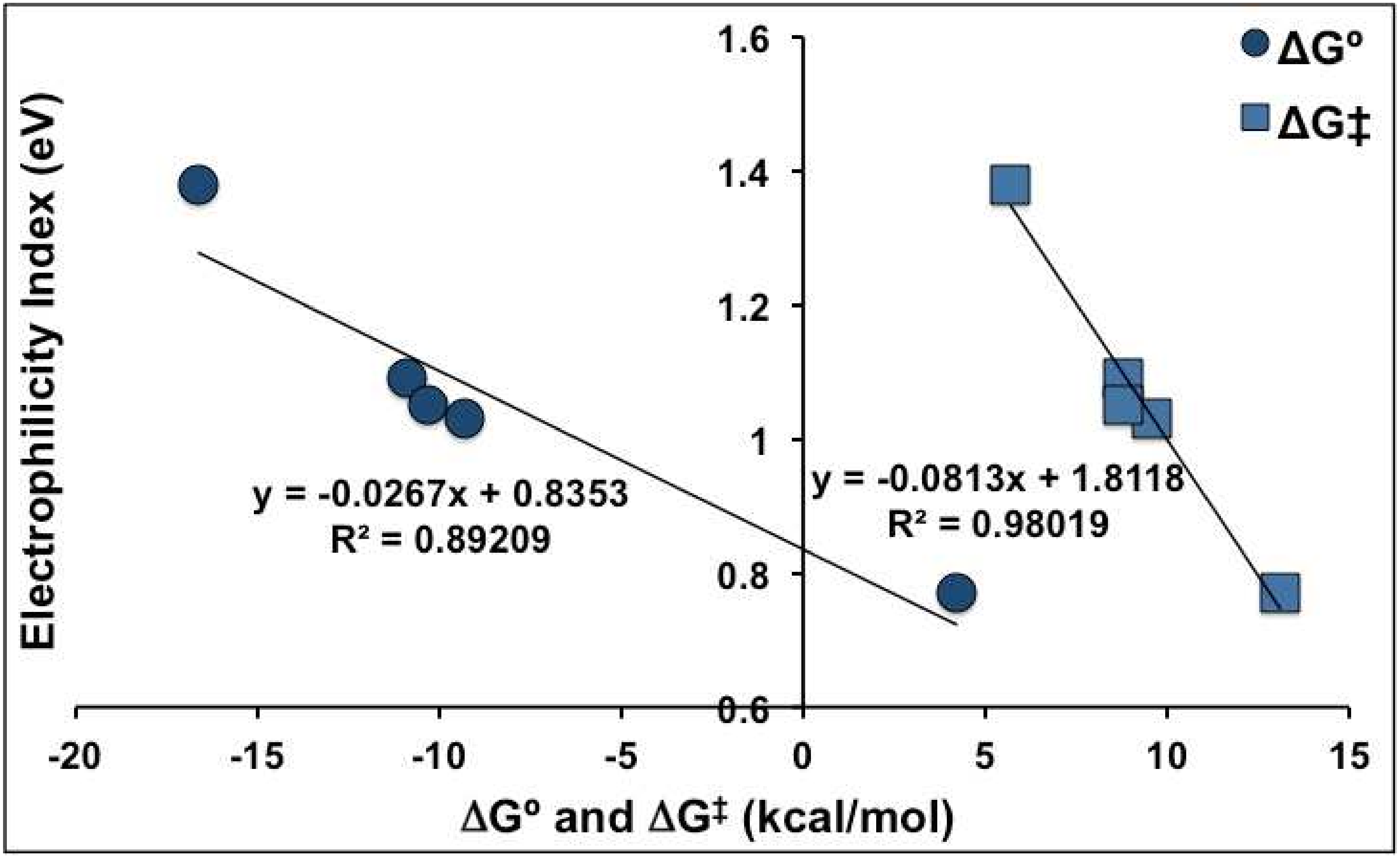

ΔGº
ΔG‡
Electrophilicity Index (eV)
1.6
1.4
1.2
1
0.8
0.6
y = -0.0267x + 0.8353
R² = 0.89209
y = -0.0813x + 1.8118
R² = 0.98019
-20
-15
-10
-5
0
5
10
15
ΔGº and ΔG‡ (kcal/mol)

7 Computational Studies

7.1 Computational Methods

Density functional theory (DFT)[3] calculations were performed using Gaussian 09 (revision E.01)[4] and the Gaussview[5] was used to generate input geometries and visualize output structures. Geometry optimizations and frequency calculations for the ring-opening and 1,5-H atom abstraction reactions, B3LYP functional[6] was used with the UB3LYP/6-31+G(d,p) basis set.[7] All stationary points were characterized as minima or transitions states based on normal vibrational mode analysis. Thermal corrections were computed from unscaled frequencies, assuming a standard state of 298.15 K and 1 atm.

Electronic properties of radicals, global and local electrophilicity index were calculated at the UB3LYP/6-311+G(d,p) level of theory, followed by frequency calculations at the same level.[8] Hirshfeld charges were also computed at the same level of theory.[9] For substrates having more than one conformations, low energy conformation of the transition state could possibly be different from the low energy ground state.[10] The structures described herein are the lowest energy-optimized conformers.

7.2 Activation Energy ($\Delta G^{\ddagger}$) and Reaction Energy (ΔG^{o}) for Hydrogen-atom Abstraction

DFT Method: UB3LYP/6-31+G(d,p) [values are in Kcal mol^{-1}]

No.	Hydrogen-atom Abstraction	$\Delta G^{\ddagger}$	ΔG^{o}
1	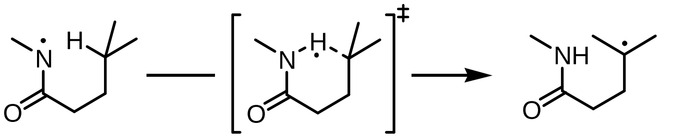	8.8	-10.9
2	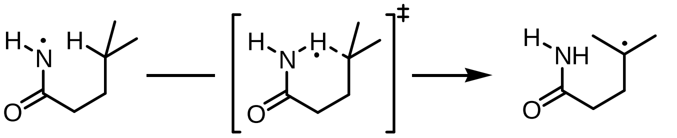	5.7	-16.6
3	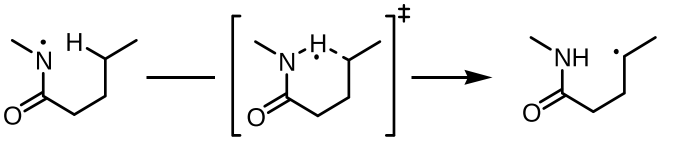	11.1	-8.3
4	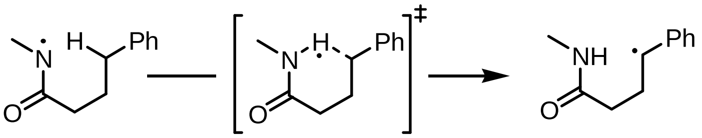	9.5	-19.5
5	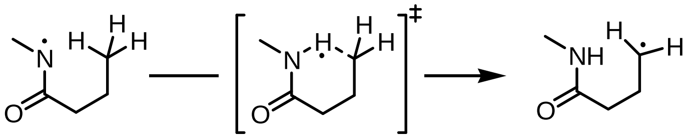	13.5	-3.4
6	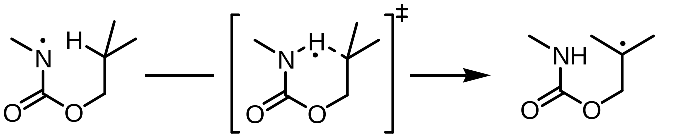	14.3	-6.4
7	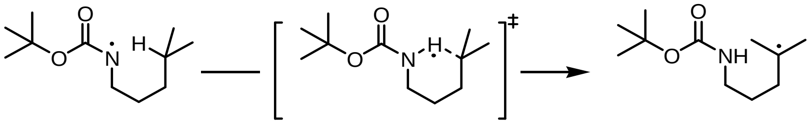	9.6	-9.3
8	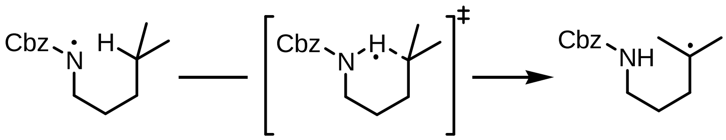	8.8	-10.3
9	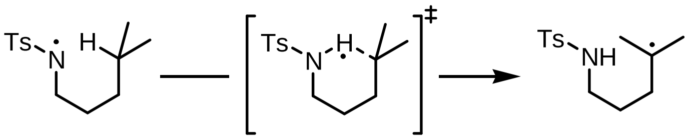	9.3	-6.1

10	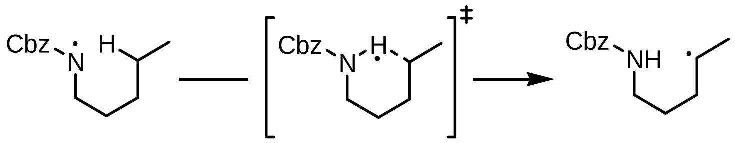	11.1	-7.3
11	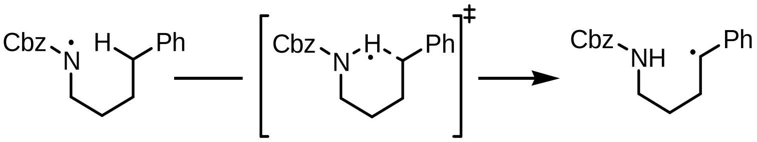	10.7	-16.5
12	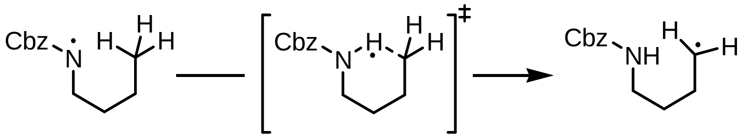	15.3	-2.7
13	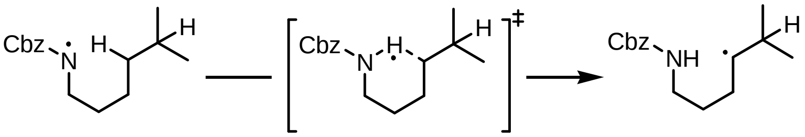	11.7	-7.4
14	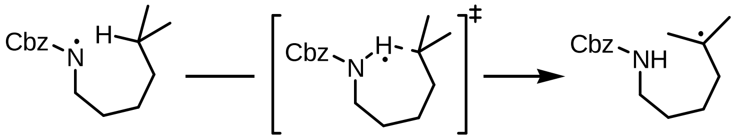	6.5	-10.6
15	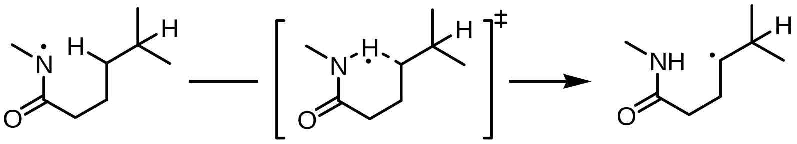	11.8	-7.3
16	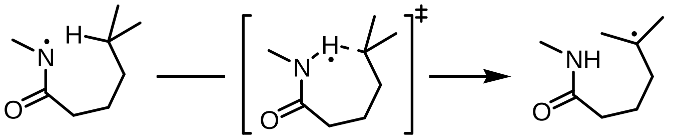	7.1	-9.7
17	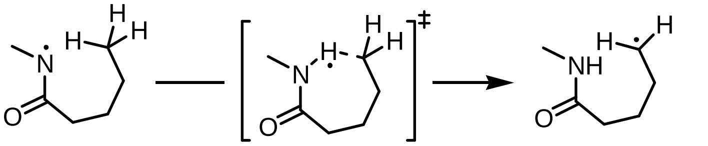	11.3	-4.8

Computed Energies [values are in Hartree]

No.	Species	Total Electronic Energy	Sum of Electronic and Zero-point Energies	Sum of Electronic and Thermal Enthalpies	Gibbs Free Energy
1	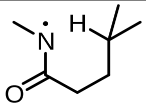	-405.1392408	-404.938041	-404.925566	-404.977418
2	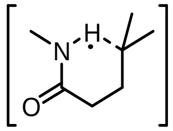	-405.1235298	-404.926821	-404.915158	-404.963401
3	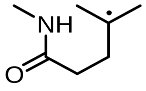	-405.1561433	-404.954893	-404.941977	-404.994742
4	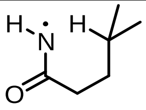	-365.8200537	-365.646892	-365.636171	-365.683288
5	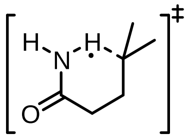	-365.8093068	-365.640236	-365.630389	-365.674283
6	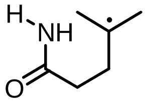	-365.845606	-365.672620	-365.661322	-365.709705
7	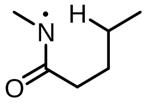	-365.8210819	-365.647793	-365.636657	-365.685506
8	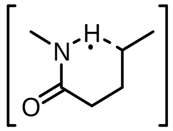	-365.8021355	-365.633151	-365.622990	-365.667883
9	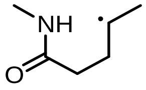	-365.8329075	-365.660100	-365.648456	-365.698669
10	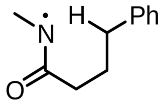	-557.568494	-557.342111	-557.328057	-557.385317

11	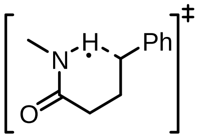	-557.5523182	-557.329813	-557.316685	-557.370156
12	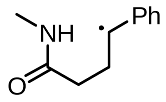	-557.6004704	-557.373084	-557.358917	-557.416355
13	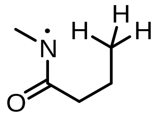	-326.5047873	-326.360180	-326.350276	-326.395713
14	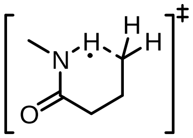	-326.4817348	-326.341252	-326.332510	-326.374223
15	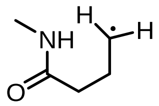	-326.5106406	-326.366189	-326.356173	-326.401094
16	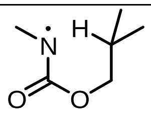	-441.0490874	-440.871389	-440.859239	-440.910705
17	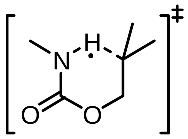	-441.0243343	-440.851481	-440.840018	-440.887988
18	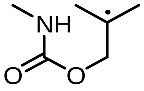	-441.0600166	-440.882377	-440.869932	-440.920960
19	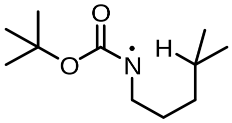	-637.6461827	-637.327685	-637.309160	-637.375481
20	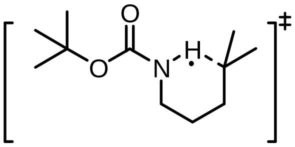	-637.6297133	-637.315624	-637.297951	-637.360186
21	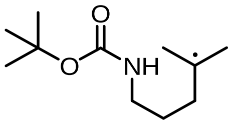	-637.6610661	-637.342579	-637.323588	-637.390381

22	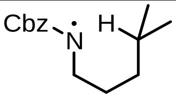	-750.7518722	-750.435894	-750.416910	-750.487447
23	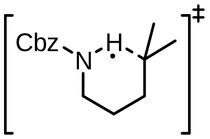	-750.7364764	-750.424752	-750.406614	-750.473462
24	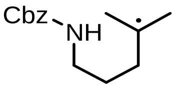	-750.7678063	-750.451790	-750.432278	-750.503847
25	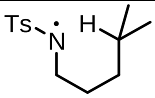	-1110.7572548	-1110.446874	-1110.426957	-1110.497771
26	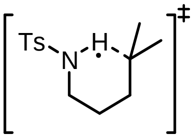	-1110.7407871	-1110.434838	-1110.415747	-1110.482888
27	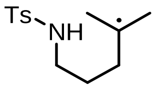	-1110.7654372	-1110.455420	-1110.435125	-1110.507421
28	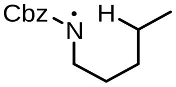	-711.4335934	-711.145598	-711.127929	-711.195621
29	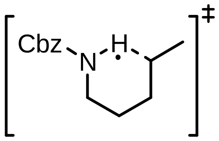	-711.4149798	-711.131142	-711.114478	-711.177970
30	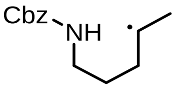	-711.4448527	-711.157095	-711.138958	-711.207230
31	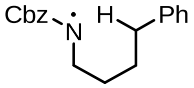	-903.1817215	-902.840526	-902.819902	-902.896031
32	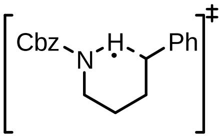	-903.1652311	-902.827395	-902.807944	-902.879007
33	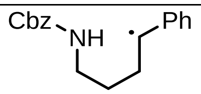	-903.209851	-902.867614	-902.846963	-902.922267

34	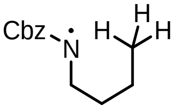	-672.117281	-671.857839	-671.841423	-671.906299
35	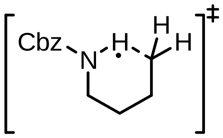	-672.0943693	-671.838128	-671.823293	-671.881917
36	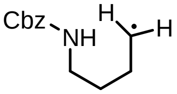	-672.1224046	-671.863014	-671.846453	-671.910622
37	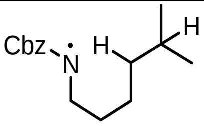	-790.0687479	-789.724277	-789.703967	-789.777573
38	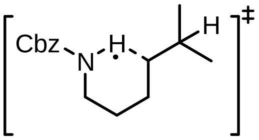	-790.0499636	-789.709271	-789.690167	-789.758852
39	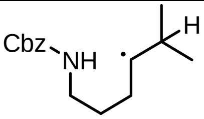	-790.079646	-789.735204	-789.714567	-789.789327
40	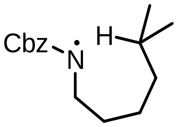	-790.0646401	-789.720174	-789.699836	-789.773551
41	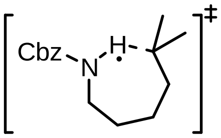	-790.0537343	-789.713301	-789.694060	-789.763212
42	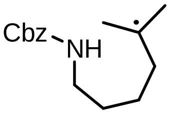	-790.0817426	-789.737056	-789.716388	-789.790433
43	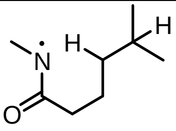	-444.4561655	-444.226602	-444.212757	-444.268344
44	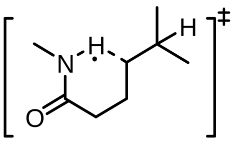	-444.4361985	-444.210842	-444.197991	-444.249517

45	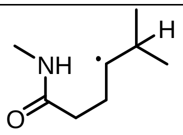	-444.4678182	-444.238348	-444.224214	-444.280041
46	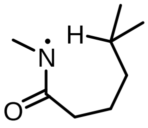	-444.4547057	-444.225202	-444.211526	-444.265904
47	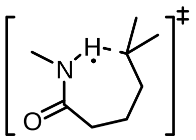	-444.4422138	-444.216748	-444.204034	-444.254623
48	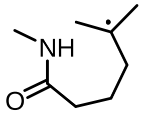	-444.4692162	-444.239494	-444.225298	-444.281309
49	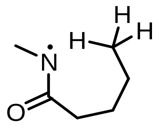	-365.8204239	-365.647381	-365.636304	-365.684374
50	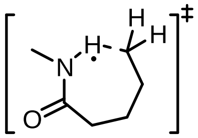	-365.8015136	-365.632217	-365.622443	-365.666417
51	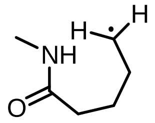	-365.827751	-365.654903	-365.643507	-365.691999

Optimized Structures and Cartesian Coordinates

<table>
<tr><th>No.</th><th>Species</th><th>Optimized Structure</th></tr>
<tr><td>1</td><td>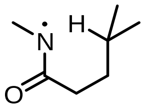</td><td>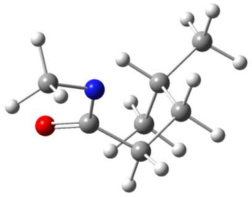</td></tr>
<tr><td colspan="3">Cartesian Coordinates
C -1.30367900 0.40421600 -0.15979800
C -0.25820000 0.38041400 -1.25284700
H -0.72794200 0.01513600 -2.17475100
H 0.06300300 1.41199700 -1.42085200
C 0.94654300 -0.53411600 -0.92566500
H 1.61825700 -0.52121200 -1.79544400
H 0.57986300 -1.56267200 -0.82774200
C 1.75406100 -0.16445000 0.33429900
H 1.06970400 -0.17682600 1.19438800
N -2.08076700 -0.73823400 -0.04865200
O -1.40470300 1.31412900 0.66833500
C -3.42415200 -0.60006800 0.44944700
H -3.69610100 0.43132500 0.69861000
H -4.10537000 -0.99502300 -0.31944400
H -3.55363300 -1.24814100 1.32548800
C 2.83833000 -1.22520300 0.58477800
C 2.37524400 1.23865200 0.24918600
H 1.61378500 2.02097800 0.17886100
H 2.97745800 1.44912300 1.13995800
H 3.03551700 1.32183300 -0.62435200
H 3.55933800 -1.25147800 -0.24250000
H 3.39565100 -1.01007800 1.50319000
H 2.40458800 -2.22703300 0.68207300</td></tr>
<tr><td>2</td><td>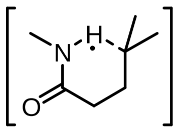</td><td>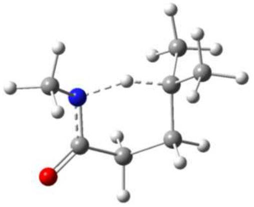</td></tr>
<tr><td colspan="3">Cartesian Coordinates
C 1.47199700 -0.51734800 0.06170600
C 0.43422300 -1.63615200 0.10712100
H 0.87549300 -2.51708600 -0.36398200
H 0.23623300 -1.88548500 1.15699500
C -0.87100800 -1.21383700 -0.60212300
H -1.62182500 -2.01234400 -0.51420100
H -0.66322300 -1.09598800 -1.67294000
C -1.42888000 0.10386500 -0.04614600
H -0.36180300 0.66688400 0.26007300
N 1.01502800 0.69632000 0.51627300
O 2.63645200 -0.69111200 -0.30494200
C 1.69540500 1.91293300 0.10417800
H 1.08154800 2.77413800 0.38119400
H 2.64415900 1.98537400 0.64948100
H 1.92252200 1.94129400 -0.96882000
C -2.15922100 0.96301200 -1.06589500</td></tr>
</table>

C	-2.16513200	-0.03305200	1.28061000
H	-3.07389900	0.45425200	-1.40605100
H	-2.46120400	1.92630600	-0.64079800
H	-1.54350200	1.15366500	-1.95134300
H	-1.57774200	-0.58593500	2.01992700
H	-2.40611300	0.94619500	1.70724500
H	-3.11176000	-0.57314200	1.13213900

3	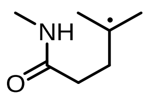	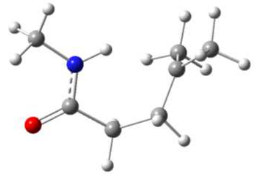

Cartesian Coordinates

C	-1.49906100	0.55163300	-0.03519000
C	-0.28814500	1.48462700	-0.09944600
H	-0.63079600	2.36187800	-0.65529800
H	-0.10266600	1.83704500	0.92300900
C	1.01266400	0.95133900	-0.73556400
H	1.68109300	1.82447700	-0.86792900
H	0.79570900	0.60019600	-1.75262000
C	1.77539100	-0.11713500	0.00833300
N	-1.25145000	-0.78718600	-0.02098000
O	-2.64300900	1.00463100	0.01648500
C	-2.32719600	-1.75937200	0.10408000
H	-3.11131200	-1.54515700	-0.62656500
H	-1.92426500	-2.75749300	-0.08091000
H	-2.78126300	-1.73316900	1.10149400
H	-0.28766000	-1.09777000	0.01327600
C	2.69233500	-0.99269500	-0.79420300
C	2.10079000	0.08320000	1.45958500
H	3.62468400	-0.46018500	-1.06045100
H	2.99433100	-1.88737100	-0.23758900
H	2.23577400	-1.31016700	-1.73892600
H	1.24531900	0.43859600	2.04330200
H	2.46254500	-0.84208000	1.92124400
H	2.90205800	0.83487600	1.58737200

4	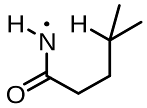	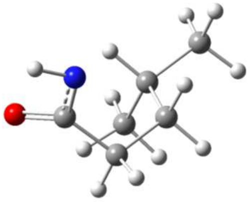

Cartesian Coordinates

C	1.79500500	-0.08654000	0.01129200
C	0.83942500	-0.01769100	-1.15228000
H	1.33188900	0.54404800	-1.95640600
H	0.67249500	-1.03817200	-1.50757300
C	-0.49476600	0.68562500	-0.80812000
H	-1.08843300	0.73864700	-1.73118600
H	-0.27304600	1.71880100	-0.51706300
C	-1.34231500	0.02127300	0.29537600
H	-0.73178600	-0.03246200	1.20818200
N	2.34181400	1.08087300	0.47445800
O	2.00577100	-1.11939100	0.67059400
C	-2.56716400	0.89658200	0.60541200
C	-1.77277500	-1.40840000	-0.06854000

H	-0.91590800	-2.07709400	-0.19403000
H	-2.40427100	-1.83421200	0.71889700
H	-2.35354900	-1.41716700	-1.00036400
H	-3.22010800	0.98218500	-0.27274300
H	-3.16133300	0.46749400	1.41962600
H	-2.27166400	1.90907300	0.90278100
H	3.23238800	0.88277800	0.94508000

5	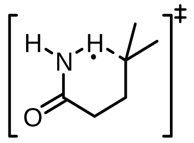	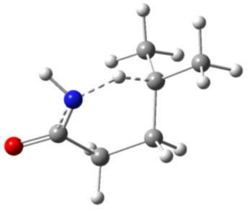

Cartesian Coordinates

C	1.69342900	0.16924700	-0.03740100
C	0.95597200	-1.14504300	-0.29097400
H	1.54250900	-1.71542200	-1.01908800
H	1.01942600	-1.71042400	0.64657600
C	-0.50181200	-0.98361600	-0.75476600
H	-1.02609700	-1.94801700	-0.68820200
H	-0.51132100	-0.69224600	-1.81130300
C	-1.25827900	0.08350900	0.04927200
H	-0.40906300	0.95714000	0.00799600
N	0.97554000	1.32027000	-0.26235200
O	2.87922400	0.20011700	0.29573300
C	-2.48513400	0.63676200	-0.66078700
C	-1.50628100	-0.26873400	1.51091900
H	-3.24178500	-0.15323300	-0.77865200
H	-2.94706100	1.45141500	-0.09319800
H	-2.23628900	1.00934100	-1.65954900
H	-0.59322600	-0.58003400	2.02742800
H	-1.93082100	0.57931000	2.05829600
H	-2.22486400	-1.09836400	1.57994100
H	1.30865400	2.10496200	0.30278600

6	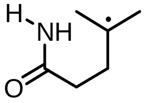	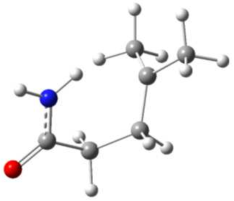

Cartesian Coordinates

C	1.83684500	0.11277500	0.03486400
C	0.87942400	-1.03538800	-0.28758000
H	1.42701100	-1.68417200	-0.97614800
H	0.75412900	-1.61553000	0.63551400
C	-0.49422500	-0.67733700	-0.89493900
H	-0.93654200	-1.62623400	-1.25554200
H	-0.33869300	-0.07169200	-1.79669700
C	-1.50307900	0.00415500	-0.00368900
N	1.26997000	1.30167400	0.39202700
O	3.05407100	-0.04257700	-0.00616100
H	0.26616000	1.41093500	0.45517000
C	-2.55732900	0.84069700	-0.66715100
C	-1.83168500	-0.59675200	1.33180200
H	-3.34844900	0.21007100	-1.11451900
H	-3.05852100	1.50640700	0.04513800
H	-2.14741000	1.44961600	-1.48116800

H	-0.94198700	-0.89724300	1.89509300
H	-2.40564600	0.09816900	1.95452100
H	-2.45208200	-1.50518300	1.21694400
H	1.87996300	2.06486200	0.64695100

7

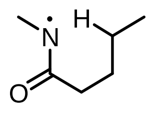

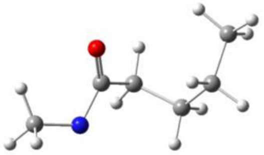

Cartesian Coordinates

C	-0.87936000	0.20761600	-0.33787200
C	0.21877000	-0.58595300	-1.00951700
H	-0.23895100	-1.40674100	-1.57557400
H	0.71940200	0.08067700	-1.71741300
C	1.23039000	-1.18693200	-0.00282700
H	1.93682300	-1.80451000	-0.57334100
H	0.68888300	-1.86536700	0.66625200
C	2.01425300	-0.16202700	0.83354800
H	1.31601100	0.45092100	1.41734100
N	-1.85876600	-0.54158400	0.29479800
O	-0.86743000	1.43804300	-0.23615600
C	-3.18462500	0.01095600	0.38648700
H	-3.28218800	0.99878700	-0.07661700
H	-3.87431800	-0.70089300	-0.09167100
H	-3.48520400	0.05626600	1.44100000
C	2.93684300	0.75329600	0.01897800
H	2.37133300	1.41269900	-0.64706100
H	3.53198400	1.39307000	0.67925600
H	3.63441600	0.16870500	-0.59393200
H	2.61498500	-0.71860700	1.56465400

8

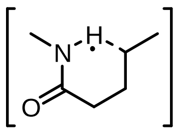

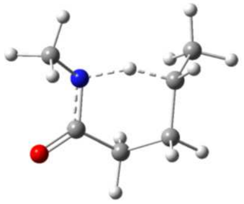

Cartesian Coordinates

C	-1.09290900	0.58068300	0.21392500
C	0.13724100	1.47920800	0.31370400
H	-0.18554700	2.50624200	0.13082000
H	0.52257600	1.42924300	1.33984200
C	1.22848800	1.04215100	-0.69041600
H	2.12479600	1.66463000	-0.55915300
H	0.86357800	1.22654000	-1.70765800
C	1.57673400	-0.43835700	-0.53080600
H	0.45994700	-0.89266400	-0.16629200
N	-0.81906200	-0.76435200	0.30093900
O	-2.24151400	1.01606000	0.11251900
C	-1.77201100	-1.72248600	-0.23626400
H	-2.12825200	-1.46306100	-1.24087100
H	-1.31146500	-2.71374700	-0.24569900
H	-2.64228000	-1.75869300	0.42950300
C	2.54562600	-0.80139100	0.57909600
H	2.22774200	-0.39403700	1.54470800
H	2.64598900	-1.88588100	0.68843100
H	3.54433700	-0.39626300	0.36093300
H	1.80510300	-0.93917800	-1.47671800

9	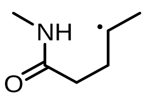	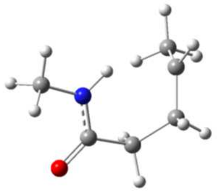

Cartesian Coordinates

C	-1.06761400	0.60738900	0.14049800
C	0.28752900	1.31742200	0.12737400
H	0.06435900	2.36060500	-0.11219000
H	0.65982400	1.32012700	1.15999800
C	1.37623500	0.78688900	-0.83513200
H	2.17238000	1.55425100	-0.85655000
H	0.97220500	0.75350700	-1.85366400
C	2.00034400	-0.53643200	-0.49397400
N	-1.07693800	-0.71796300	-0.17127900
O	-2.09661700	1.21332300	0.44101900
C	-2.30308100	-1.50105200	-0.12915200
H	-3.07865100	-1.02255200	-0.73335400
H	-2.09861900	-2.49676600	-0.52835300
H	-2.68409800	-1.59385200	0.89406000
H	-0.19356600	-1.17819800	-0.35277000
C	2.65682100	-0.78352600	0.82675000
H	2.01052500	-0.52050600	1.67365900
H	2.95192600	-1.83082100	0.94190100
H	3.57195400	-0.17512700	0.93974200
H	2.26186000	-1.19565000	-1.31986200

10	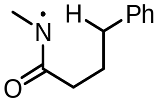	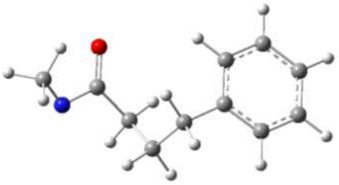

Cartesian Coordinates

C	-2.31460900	-0.43040800	0.56452800
C	-1.26377200	0.38783300	1.28484200
H	-1.72807600	0.79717600	2.18997200
H	-0.46118700	-0.28871400	1.58872200
C	-0.71970800	1.55203900	0.43245200
H	-0.08760600	2.18045300	1.07078300
H	-1.55843900	2.18091100	0.10801200
C	0.09130100	1.11317000	-0.80941400
H	-0.52294900	0.45275400	-1.43465700
N	-3.46932800	0.25197300	0.20913600
O	-2.23374000	-1.64856500	0.38361700
C	-4.19093300	-0.18857600	-0.95540500
H	-3.70500800	-1.00863400	-1.49490800
H	-5.20534100	-0.48594700	-0.65875800
H	-4.31681500	0.68192300	-1.61625000
H	0.29545100	2.00602600	-1.41292300
C	1.40120200	0.42318500	-0.48368600
C	2.54273000	1.17860100	-0.17170100
C	1.50575000	-0.97567700	-0.46859300
C	3.75149500	0.55772300	0.15060000
H	2.48634200	2.26502200	-0.18926200
C	2.71370700	-1.60174900	-0.14676900
H	0.63456300	-1.58096200	-0.70628300

C	3.84082900	-0.83745100	0.16456400
H	4.62380600	1.16210500	0.38377300
H	2.77198000	-2.68657500	-0.14119900
H	4.78054900	-1.32297500	0.41157900

11

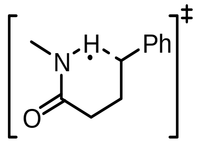

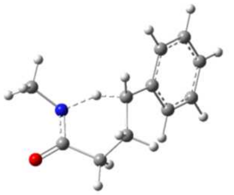

Cartesian Coordinates

C	-2.68201600	0.32897200	0.32825800
C	-1.81517600	1.53814800	-0.00514100
H	-2.48042500	2.37777100	-0.21808800
H	-1.22915500	1.79120300	0.88645500
C	-0.88090800	1.25243700	-1.20544600
H	-0.20998100	2.10443000	-1.36684100
H	-1.49891200	1.17098900	-2.10667000
C	-0.08080400	-0.05220200	-1.04333700
H	-0.78119500	-0.66414200	-0.25141800
N	-1.97266700	-0.82517000	0.56879500
O	-3.90998900	0.38415500	0.43114500
C	-2.64711100	-2.10276900	0.42281500
H	-3.25830600	-2.27783200	1.31755300
H	-3.31009400	-2.15010000	-0.44967600
H	-1.89636100	-2.89628200	0.37413200
H	-0.10534300	-0.67226400	-1.94435600
C	1.28611900	-0.02024200	-0.45790900
C	1.72066500	1.00206700	0.40818100
C	2.18473600	-1.06910800	-0.74245200
C	3.00239800	0.97978800	0.95855800
H	1.05663200	1.82525200	0.65074900
C	3.46473400	-1.09377100	-0.19229500
H	1.87073800	-1.86830600	-1.40965300
C	3.88015600	-0.06775300	0.66327100
H	3.31663200	1.78192600	1.62020800
H	4.14018300	-1.90971400	-0.43296400
H	4.87742100	-0.08338000	1.09282100

12

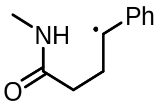

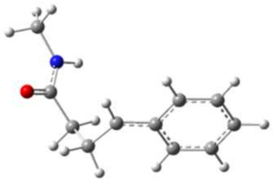

Cartesian Coordinates

C	-2.65980500	-0.28328400	-0.05736700
C	-1.60910800	-1.25189200	-0.59538600
H	-2.14877600	-2.14008300	-0.94912000
H	-1.08091400	-0.82625300	-1.45633400
C	-0.59779900	-1.68023000	0.49800300
H	0.01209100	-2.49773800	0.09738900
H	-1.17581200	-2.08576100	1.33488100
C	0.26108700	-0.55486700	0.98965500
N	-2.95051400	0.78215900	-0.85935300
O	-3.23166200	-0.47533300	1.01464800
C	-3.92953900	1.79231900	-0.48536700
H	-3.48886200	2.57623400	0.14272000

H	-4.34381000	2.24812600	-1.38799500
H	-4.73031800	1.31283400	0.07984600
H	-2.37924100	0.93988000	-1.67581300
H	-0.14335400	0.05194200	1.79696500
C	1.52298800	-0.17698900	0.46651600
C	2.16347800	-0.86054300	-0.61131600
C	2.21688900	0.94094600	1.02398700
C	3.40229000	-0.44990700	-1.08835000
H	1.67885200	-1.71841900	-1.06648000
C	3.45338800	1.34204500	0.54022700
H	1.75615700	1.48014900	1.84779800
C	4.05984300	0.65177500	-0.52153600
H	3.86533600	-0.99113300	-1.90910700
H	3.95504700	2.19558300	0.98794900
H	5.02822100	0.96596200	-0.89880300

13	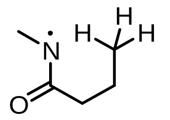	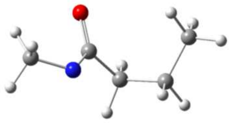

Cartesian Coordinates

C	-0.48576700	0.23829900	-0.34600400
C	0.72020200	-0.40936600	-0.99001400
H	0.39265100	-1.31847000	-1.50961900
H	1.11154100	0.29359400	-1.73282000
C	1.82186900	-0.78314800	0.02986300
H	2.60028700	-1.33440900	-0.51189400
H	1.39998700	-1.47717500	0.76580200
C	2.44587100	0.42270600	0.74004400
H	1.70275100	0.98457100	1.31496500
N	-1.33042800	-0.62289300	0.33803400
O	-0.66791000	1.45823300	-0.30942100
C	-2.72938000	-0.29120400	0.40483600
H	-2.97283300	0.66962700	-0.06176100
H	-3.28606100	-1.09972700	-0.09402000
H	-3.05650600	-0.30148400	1.45161800
H	3.22971900	0.09801800	1.43268600
H	2.89797300	1.11611900	0.02182100

14	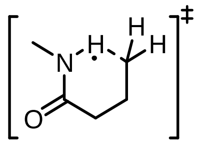	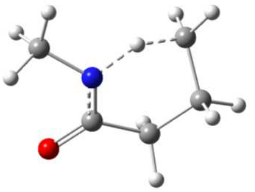

Cartesian Coordinates

C	-0.50635900	0.72754400	-0.02065700
C	0.95940600	1.15085100	0.09703300
H	1.08413900	2.06964200	-0.48436700
H	1.10355200	1.43484800	1.14833700
C	1.97665100	0.07135000	-0.30919000
H	2.98889800	0.38331100	-0.01639800
H	1.98341800	-0.03791700	-1.39979300
C	1.61162700	-1.26006300	0.32355600
H	0.37643100	-1.26031900	0.00386300
N	-0.72743600	-0.59037600	-0.33839800
O	-1.42860100	1.52992400	0.13193800
C	-1.99424800	-1.21363100	0.00341800
H	-2.77992800	-0.76333500	-0.61397400
H	-1.94158200	-2.27972900	-0.23033700

H	-2.27208800	-1.07165700	1.05523000
H	2.04694700	-2.15266100	-0.12996800
H	1.64860900	-1.28525300	1.41572700
15	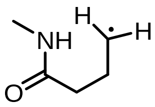	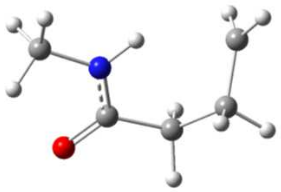	
Cartesian Coordinates			
C	-0.57814200	0.64905500	0.04810000
C	0.88837700	1.08059100	0.12854800
H	0.92932800	2.05263400	-0.37077500
H	1.10490100	1.27573900	1.18791600
C	1.96003300	0.13973200	-0.45662100
H	2.89775600	0.72189400	-0.52416900
H	1.71162800	-0.11514800	-1.49562700
C	2.22392000	-1.10129100	0.33168300
N	-0.83068000	-0.68507300	-0.06756900
O	-1.48083800	1.48452300	0.09411200
C	-2.19006200	-1.20558600	-0.07290600
H	-2.81227000	-0.59092000	-0.72682200
H	-2.17629300	-2.23228200	-0.44549100
H	-2.63632000	-1.19174300	0.92886200
H	-0.05556700	-1.33143600	0.00896400
H	2.69027900	-1.96461400	-0.13366900
H	2.18326300	-1.07979900	1.41808200
16	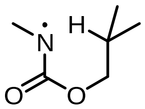	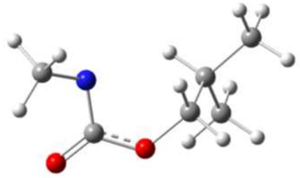	
Cartesian Coordinates			
C	1.40955000	-0.57999500	-0.06453200
C	-0.77779300	0.10401200	-0.87145400
H	-1.28295600	-0.23724400	-1.78068000
H	-0.30170300	1.06741100	-1.07350500
C	-1.76427300	0.21080900	0.29333600
H	-1.18412900	0.48775400	1.18349700
N	1.58057900	0.74288500	0.32000900
O	2.23917000	-1.44205400	0.19125700
C	2.91106400	1.28360300	0.20378400
H	3.64754600	0.57246600	-0.18331200
H	2.85473200	2.16460700	-0.45383100
H	3.23075900	1.66103500	1.18328700
C	-2.76841200	1.33715500	0.00289200
C	-2.47281600	-1.12471900	0.56054400
H	-1.75485300	-1.92619400	0.75600800
H	-3.13643600	-1.04280700	1.42808800
H	-3.08448900	-1.42180100	-0.30095100
H	-3.35671200	1.12105000	-0.89793200
H	-3.47091200	1.45048300	0.83492200
H	-2.26663700	2.30038600	-0.14350800
O	0.23805700	-0.90576200	-0.63370300

17	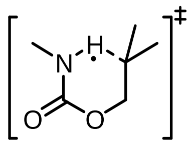	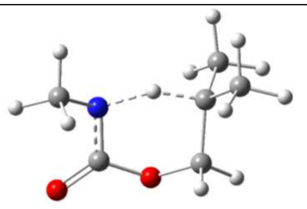

Cartesian Coordinates

C	1.43560000	-0.52169700	0.03590200
C	-0.78819100	-1.08024000	-0.72340200
H	-1.41094600	-1.98082700	-0.68266400
H	-0.61089800	-0.83329800	-1.77830200
C	-1.43535800	0.09354700	0.00295300
H	-0.38015400	0.72030000	0.24857900
N	0.98258600	0.71572700	0.43928000
O	2.61310700	-0.81732800	-0.05898400
C	1.69855000	1.90476700	0.00684300
H	1.68097300	2.05414200	-1.08127000
H	1.27286900	2.77932900	0.50395000
H	2.74574400	1.80196700	0.31356400
C	-2.36108500	0.92689600	-0.86528800
C	-1.98201200	-0.25068800	1.38001300
H	-3.24749000	0.34354400	-1.15713500
H	-2.71960000	1.81016500	-0.32696900
H	-1.87045700	1.26002700	-1.78607300
H	-1.24696200	-0.80021000	1.97473800
H	-2.26764400	0.65052900	1.93200400
H	-2.87858100	-0.88062700	1.28316500
O	0.46814500	-1.47900200	-0.13360000

18	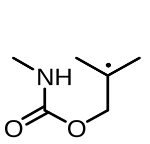	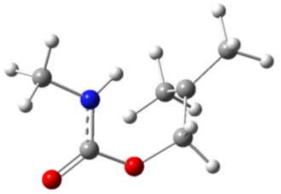

Cartesian Coordinates

C	1.39037400	-0.56876400	-0.09575800
C	-0.91365500	-0.65786100	-0.92835300
H	-1.48367800	-1.51058400	-1.33150400
H	-0.62809900	-0.02755000	-1.78057000
C	-1.74266600	0.11184100	0.06045500
N	1.26964000	0.79714200	-0.12950900
O	2.42854000	-1.15649400	0.13960800
C	2.40526400	1.62160100	0.26890700
H	3.29713000	1.28698700	-0.26300600
H	2.19544700	2.65892200	-0.00208700
H	2.60715900	1.56024900	1.34531500
H	0.34338700	1.18451200	0.00271500
C	-2.74028100	1.08398600	-0.49283500
C	-1.97856900	-0.47329500	1.41855000
H	-3.67067100	0.57677800	-0.80695400
H	-3.03423500	1.82598300	0.25872800
H	-2.35934900	1.61625400	-1.37233800
H	-1.10193200	-1.00973400	1.79169400
H	-2.25676500	0.29940000	2.14435700
H	-2.81226200	-1.19883000	1.39602300
O	0.25815900	-1.28693600	-0.37230800

19	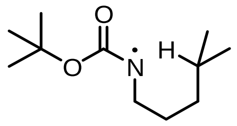	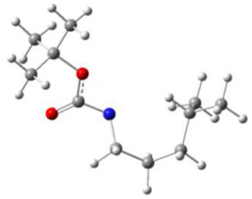

Cartesian Coordinates

C	-1.05913400	-1.69227400	-0.49661000
C	-2.56057000	-1.71961600	-0.20172600
H	-2.97254100	-2.60327700	-0.70641900
H	-2.70291000	-1.88501300	0.87244400
C	-3.35138800	-0.48767900	-0.67893200
H	-4.42393300	-0.70889800	-0.57647900
H	-3.17387300	-0.36221300	-1.75766900
C	-3.06749600	0.85553400	0.02636200
H	-1.99410200	1.07086000	-0.05765500
N	-0.31971100	-0.80112500	0.36951500
C	1.06937200	-0.85888300	0.25136100
C	-3.83398200	1.98690000	-0.67781500
C	-3.41491200	0.81380200	1.52208600
H	-2.80678900	0.08108000	2.06045100
H	-3.24006800	1.78979100	1.98889100
H	-4.47255100	0.55832200	1.67185300
H	-4.91799100	1.82149400	-0.62191400
H	-3.62247500	2.95699300	-0.21413000
H	-3.56267400	2.05641600	-1.73785600
H	-0.62165700	-2.69796300	-0.38082800
H	-0.87712400	-1.41416400	-1.55152400
O	1.70328500	-1.88285700	0.46056500
O	1.56576800	0.35328500	-0.02488100
C	3.02802400	0.60896100	-0.08305200
C	3.64919200	0.33289700	1.28931200
C	3.66154000	-0.23265700	-1.19496000
C	3.08664500	2.09936000	-0.42269200
H	3.13305600	0.90811300	2.06446200
H	3.60156200	-0.72722300	1.54272500
H	4.69948900	0.64309900	1.27828000
H	4.71186000	0.05501100	-1.31079000
H	3.61588000	-1.29818600	-0.96471400
H	3.15467600	-0.05132500	-2.14841700
H	2.59418200	2.29796700	-1.37928100
H	2.59030600	2.69202600	0.35124200
H	4.12947300	2.42345800	-0.49476200

20	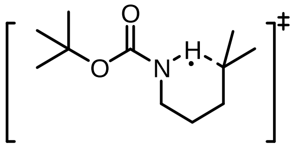	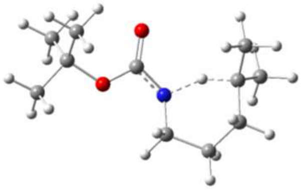

Cartesian Coordinates

C	-0.83805600	1.71850500	-0.05993100
C	-2.35344800	1.96865100	-0.00725600
H	-2.53855100	2.93444500	0.47591700
H	-2.74354300	2.04048700	-1.02971400
C	-3.07335200	0.84059300	0.75963200
H	-4.15363700	1.04659700	0.79433100
H	-2.71909600	0.84055200	1.79892000

C	-2.81963600	-0.53366700	0.13019400
H	-1.69282200	-0.34492200	-0.35285400
N	-0.58350400	0.43656600	-0.70886800
C	0.55091600	-0.29762300	-0.42465900
C	-2.71588400	-1.68723900	1.11452800
C	-3.68285800	-0.84145000	-1.08701600
H	-3.69034300	-1.86851500	1.59333900
H	-2.41088800	-2.61117800	0.61385900
H	-1.99156800	-1.47795500	1.90859700
H	-3.67861600	-0.02021900	-1.81039100
H	-3.33465400	-1.74366800	-1.59966100
H	-4.72485800	-1.01172300	-0.77788300
H	-0.40930600	1.76756400	0.95120200
H	-0.35535400	2.50614200	-0.65497800
O	1.58896300	0.51135600	-0.08211700
O	0.60960900	-1.51529800	-0.52718700
C	2.95567700	-0.01937200	0.10302700
C	2.98386600	-0.98658300	1.29149700
C	3.44574500	-0.67311200	-1.19359300
C	3.76340100	1.24402800	0.41191100
H	3.70910400	1.95133200	-0.42121700
H	3.38052100	1.73740600	1.31052900
H	4.81350000	0.98462500	0.57947900
H	2.58458000	-0.49967500	2.18755600
H	2.39879100	-1.88337400	1.08400700
H	4.01915000	-1.27929600	1.49791100
H	4.50183700	-0.94359800	-1.08690400
H	2.87545800	-1.57369700	-1.42534700
H	3.35802300	0.02786100	-2.03019800

21	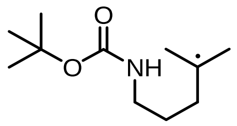	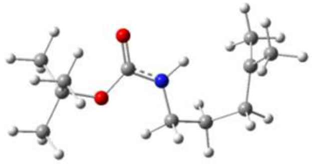

Cartesian Coordinates

C	0.74484000	1.41498400	-0.19017300
C	2.14047000	1.81181900	0.30416900
H	2.24578800	2.89150100	0.14165400
H	2.19833900	1.65348200	1.38794900
C	3.31840800	1.10349900	-0.40840700
H	4.23764300	1.66418700	-0.15340000
H	3.18923700	1.22219300	-1.49248100
C	3.53575200	-0.35301600	-0.08159600
N	0.42690400	0.02854100	0.13807900
C	-0.81166200	-0.54546400	0.12414900
C	3.89779700	-1.29497300	-1.19091600
C	3.95105600	-0.72406700	1.31208400
H	3.45497100	-0.11670500	2.07634200
H	3.74381100	-1.77781000	1.53135200
H	5.03922700	-0.58143000	1.45149700
H	4.96355600	-1.19788200	-1.47228600
H	3.74721900	-2.34167400	-0.90205600
H	3.31582000	-1.10280900	-2.09961400
H	-0.00600600	2.05614700	0.27785600
H	0.67830300	1.58472700	-1.27662200
O	-0.99890000	-1.74729500	0.27065900
O	-1.78033700	0.38966800	-0.05181200
C	-3.20866300	0.02302900	-0.04248000
H	1.18883600	-0.63890100	0.13683200

C	-3.89876000	1.37330900	-0.25613800
C	-3.51611400	-0.93108700	-1.20215800
C	-3.59194300	-0.57158400	1.31741900
H	-3.19243700	-0.49205600	-2.15182300
H	-3.01638500	-1.89092700	-1.06612400
H	-4.59710600	-1.09970400	-1.25733000
H	-3.64828000	2.06829800	0.55125700
H	-3.58845200	1.81942500	-1.20602000
H	-4.98487200	1.23939300	-0.27394400
H	-4.67657600	-0.72021300	1.35656900
H	-3.09991600	-1.53101000	1.48262000
H	-3.31424000	0.11430000	2.12472400

22	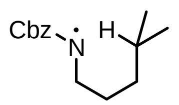	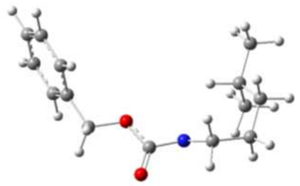

Cartesian Coordinates

C	2.55075300	1.58861000	-0.61565100
C	3.91872100	0.94973200	-0.36890900
H	4.65135000	1.51644200	-0.95809400
H	4.18931200	1.09678600	0.68296000
C	4.03374300	-0.53388100	-0.76463100
H	5.09402100	-0.82136400	-0.70995000
H	3.75203400	-0.62976200	-1.82405200
C	3.21528500	-1.55236000	0.05695400
H	2.15712200	-1.26070400	0.01744400
N	1.55109900	1.19362300	0.35114700
C	0.32265300	1.83855900	0.24507800
C	3.34237500	-2.95050900	-0.56931300
C	3.63276700	-1.58050400	1.53499100
H	3.45868700	-0.61896000	2.02676400
H	3.06094600	-2.33663100	2.08466900
H	4.69752200	-1.83000300	1.63684500
H	4.38487700	-3.29482800	-0.55325300
H	2.74182800	-3.68528800	-0.02153300
H	3.00643000	-2.95493200	-1.61286900
H	2.62105900	2.68879200	-0.57445400
H	2.19239000	1.35998500	-1.63624100
O	0.18689200	3.05216800	0.28547100
O	-0.68472900	0.95122700	0.18601900
C	-2.03035900	1.52017700	0.17164800
H	-2.16917800	2.07984500	1.10058400
H	-2.10163700	2.22020500	-0.66487200
C	-3.01217300	0.38914100	0.04333200
C	-3.47906700	-0.00847000	-1.21648900
C	-3.46907200	-0.28803000	1.18237500
C	-4.38360000	-1.06577200	-1.33826100
H	-3.13359200	0.51367900	-2.10527200
C	-4.37227500	-1.34611300	1.06457800
H	-3.11380500	0.01481600	2.16402100
C	-4.83102300	-1.73692400	-0.19697100
H	-4.74032400	-1.36254900	-2.32027500
H	-4.71997600	-1.86231200	1.95468600
H	-5.53642000	-2.55768300	-0.28944300

23	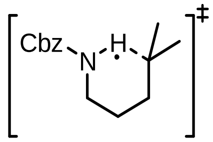	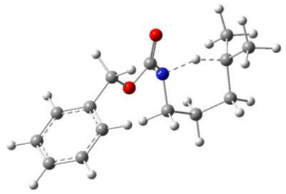

Cartesian Coordinates

C	1.39999800	1.48734100	0.27566300
C	2.78084700	2.14757200	0.14258600
H	2.65318700	3.16465400	-0.24405900
H	3.23652900	2.23541900	1.13618900
C	3.69608400	1.33162900	-0.79349100
H	4.67411600	1.82806800	-0.87949100
H	3.25561300	1.32593800	-1.79919100
C	3.88089600	-0.10939200	-0.30481900
H	2.80044700	-0.28320900	0.27223100
N	1.56221100	0.13452700	0.79977700
C	0.63425400	-0.84594400	0.53136000
C	3.99498900	-1.15502300	-1.40232500
C	4.90916000	-0.27224300	0.80801000
H	4.92897800	-1.01285400	-1.96698100
H	4.00421100	-2.16799800	-0.98857000
H	3.16524900	-1.08727500	-2.11365900
H	4.75712600	0.45112500	1.61507200
H	4.86582800	-1.27589000	1.24261800
H	5.92337100	-0.12240200	0.40913300
H	0.87521200	1.50200100	-0.68989200
H	0.78572200	2.05728900	0.98640500
O	-0.61520600	-0.32714400	0.34482700
O	0.87313300	-2.04472300	0.51677600
C	-1.68369600	-1.30397900	0.21392200
H	-1.46180700	-1.95508700	-0.63562000
H	-1.69928200	-1.92015100	1.11786400
C	-2.97494200	-0.55526400	0.02200900
C	-3.50569100	-0.36621900	-1.26062300
C	-3.66277400	-0.02787600	1.12427900
C	-4.69873000	0.33845200	-1.44178600
H	-2.98246900	-0.77518200	-2.12142400
C	-4.85316400	0.67946100	0.94763000
H	-3.26079400	-0.17227400	2.12389500
C	-5.37393200	0.86395100	-0.33721900
H	-5.10048400	0.47432900	-2.44182700
H	-5.37678600	1.08177300	1.81015000
H	-6.30264300	1.41017900	-0.47527700

24	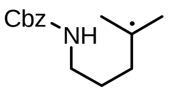	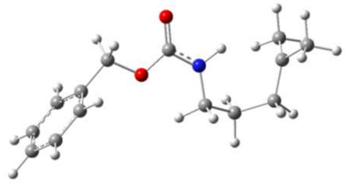

Cartesian Coordinates

C	1.36638000	-1.11962600	0.12972900
C	2.60787000	-1.85350100	-0.38924100
H	2.41195100	-2.92826100	-0.29095900
H	2.72224700	-1.65581400	-1.46191300
C	3.92262100	-1.53744400	0.36329000
H	4.66122100	-2.30637500	0.06584200

H	3.75254300	-1.69112400	1.43732200
C	4.53081400	-0.17497500	0.14025900
N	1.44383100	0.31885500	-0.11351300
C	0.39881400	1.19108800	-0.13309400
C	5.17381200	0.52031000	1.30335300
C	5.00689400	0.18780200	-1.23616700
H	4.33441100	-0.16739400	-2.02374700
H	5.12096500	1.27149800	-1.35368200
H	5.99843100	-0.25779100	-1.44143200
H	6.17425800	0.10087300	1.52153100
H	5.32120600	1.58886800	1.10847700
H	4.58479800	0.41657600	2.22189000
H	0.47566400	-1.49992200	-0.37516100
H	1.23891500	-1.32556700	1.20408000
O	0.51043100	2.40742700	-0.21016100
O	-0.79868700	0.54263300	-0.07641900
C	-1.96846500	1.39898600	-0.14957800
H	-1.91883700	2.12935700	0.66227400
H	-1.94371300	1.94375200	-1.09759200
C	-3.18864500	0.52397100	-0.04057100
C	-3.76646100	-0.04009700	-1.18620900
C	-3.76155000	0.25363600	1.20949100
C	-4.89079600	-0.86248400	-1.08546700
H	-3.33303800	0.16820400	-2.16124000
C	-4.88636600	-0.56773700	1.31506800
H	-3.32365200	0.69048500	2.10350900
C	-5.45289600	-1.12807100	0.16664500
H	-5.33020400	-1.29093400	-1.98176900
H	-5.32200000	-0.76639300	2.29012900
H	-6.33014500	-1.76373200	0.24628800
H	2.35205200	0.76006900	-0.03166700

25

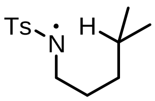

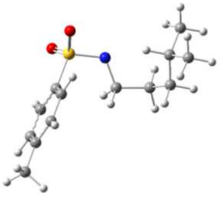

Cartesian Coordinates

C	0.97397700	0.17733400	-1.43966400
C	2.31725700	-0.37654700	-1.93142300
H	2.13610900	-0.83049500	-2.91413700
H	3.00355800	0.46084700	-2.09742000
C	2.95276200	-1.43981800	-1.01775500
H	3.80294600	-1.88584100	-1.55422600
H	2.22614500	-2.25446000	-0.87956700
C	3.44636900	-0.97419600	0.36830000
H	2.60785300	-0.49861200	0.89586000
N	1.14619500	1.11884900	-0.35331300
C	3.89510300	-2.18803100	1.19795900
C	4.57473300	0.06342500	0.26901000
H	4.24590700	0.97812700	-0.23247200
H	4.92619700	0.34895900	1.26646600
H	5.43247900	-0.34302400	-0.28337200
H	4.74106000	-2.69879900	0.71961600
H	4.21500200	-1.88392900	2.20060800
H	3.08606500	-2.91932300	1.31142600
H	0.47704800	0.72326500	-2.26239600
H	0.28795000	-0.64130300	-1.16877100
S	-0.23252800	1.72852800	0.43050800

C	-1.59891900	0.57386800	0.25575300
C	-2.51112300	0.73318400	-0.78948800
C	-1.73681700	-0.46688400	1.17914500
C	-3.56911200	-0.16913100	-0.91009400
H	-2.40575200	1.56616200	-1.47651200
C	-2.79835500	-1.35962200	1.04059400
H	-1.03757600	-0.55562800	2.00398200
C	-3.72738500	-1.22961300	-0.00513200
H	-4.28848700	-0.03889300	-1.71411800
H	-2.91475300	-2.16242500	1.76394700
O	-0.56797500	2.94812300	-0.32273300
O	0.12549300	1.78032900	1.85123200
C	-4.85990200	-2.21680700	-0.15558900
H	-5.68247700	-1.79643900	-0.74081000
H	-4.51933700	-3.12468500	-0.66899900
H	-5.25451600	-2.52248000	0.81827800

26	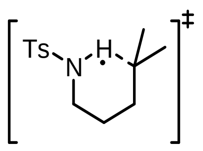	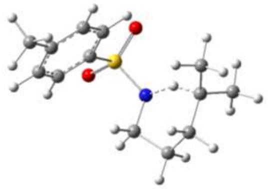

Cartesian Coordinates

C	1.41645200	0.67477100	-1.66034400
C	2.56225100	-0.26902500	-2.05501600
H	2.48791300	-0.48675900	-3.12616500
H	3.51899000	0.24111400	-1.89577400
C	2.50754000	-1.57921300	-1.24380500
H	3.34581000	-2.22948000	-1.53702900
H	1.58645100	-2.11694600	-1.50420700
C	2.54488200	-1.33544200	0.26722000
H	1.94012500	-0.21434200	0.30960200
N	1.53591900	0.97479800	-0.23068500
C	1.76948900	-2.34097800	1.10000000
C	3.91714100	-0.99688500	0.83126900
H	2.25027000	-3.32957300	1.04052300
H	1.74384300	-2.05194100	2.15569100
H	0.74030200	-2.45589900	0.74506900
H	4.41721700	-0.21276600	0.25573200
H	3.84491600	-0.65589900	1.86872400
H	4.56025500	-1.88974700	0.81525100
H	0.45061900	0.21437400	-1.92117700
H	1.49586000	1.61999200	-2.21247900
S	0.22761500	1.65500100	0.57454400
C	-1.24544300	0.65653300	0.28048800
C	-2.07744300	0.94866600	-0.80304500
C	-1.54756100	-0.39865300	1.14643800
C	-3.21113900	0.16433700	-1.02501800
H	-1.85147800	1.79361800	-1.44464300
C	-2.68510600	-1.17001100	0.91149900
H	-0.91144600	-0.59132100	2.00358000
C	-3.53356200	-0.90413500	-0.17572800
H	-3.85996000	0.39435300	-1.86620000
H	-2.92440600	-1.98480100	1.59031500
O	0.54714900	1.55605400	2.00342300
O	-0.00905200	2.95639200	-0.07354600
C	-4.78265400	-1.72361700	-0.39776800
H	-5.11743500	-1.66900200	-1.43760400
H	-4.62104100	-2.77633300	-0.14555200

H	-5.60394700	-1.35990600	0.23227600

27	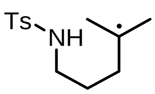	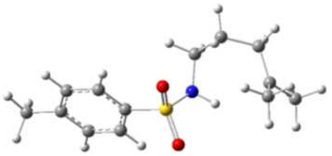

Cartesian Coordinates

C	1.18343300	1.42984600	-0.71813000
C	2.46760400	1.88918300	-0.01312000
H	2.51576700	2.98281900	-0.09892700
H	2.39111800	1.66311900	1.05503100
C	3.76555500	1.30299400	-0.61370500
H	4.61302900	1.85422400	-0.16215300
H	3.79636700	1.54378300	-1.68535300
C	3.99827900	-0.17575500	-0.42421600
N	0.85862100	-0.00309200	-0.58294500
H	1.67104500	-0.61310000	-0.69549800
C	4.68106500	-0.93415000	-1.52415700
C	4.12978800	-0.71218700	0.97174600
H	5.77264900	-0.75207800	-1.52170400
H	4.54822200	-2.01679300	-1.41481200
H	4.31834600	-0.64022700	-2.51611800
H	3.41762100	-0.26187600	1.66994200
H	3.98849700	-1.79812800	1.00397400
H	5.14074300	-0.51128600	1.37356900
H	0.32738200	2.00079600	-0.34609900
H	1.25202000	1.63278500	-1.79459100
S	-0.00157000	-0.52946200	0.76756100
C	-1.70857000	-0.25903900	0.27666900
C	-2.48963500	0.65624200	0.97919500
C	-2.24514500	-1.00035700	-0.78164300
C	-3.82596300	0.83605000	0.60840800
H	-2.05693900	1.20860000	1.80615700
C	-3.57660700	-0.80865800	-1.13660400
H	-1.62737500	-1.71651700	-1.31292900
C	-4.38885900	0.11238400	-0.44979300
H	-4.43712300	1.54820700	1.15628800
H	-3.99671200	-1.38493900	-1.95713700
O	0.26110500	-1.97439900	0.83428300
O	0.24586700	0.32969800	1.93857000
C	-5.83190200	0.30564400	-0.85125100
H	-6.33333700	1.03095200	-0.20496500
H	-5.90942900	0.66462700	-1.88412900
H	-6.38714800	-0.63750800	-0.79412800

28	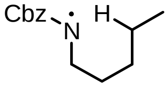	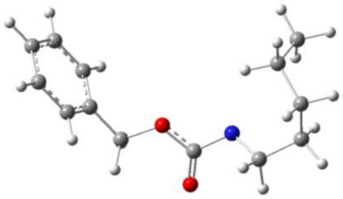

Cartesian Coordinates

C	2.91844500	1.21794100	-0.50538700
C	4.19981700	0.39168100	-0.38213700
H	4.99846300	0.95275200	-0.88440400
H	4.47932100	0.33535200	0.67637700
C	4.13321800	-1.01505800	-1.00601200
H	5.15042100	-1.43105200	-1.01690700

H	3.83932500	-0.91208000	-2.06083400
C	3.19606500	-2.02813400	-0.32388400
H	2.16931400	-1.64309900	-0.31640800
N	1.87597200	0.79476700	0.40264200
C	0.71133200	1.55588200	0.37910800
C	3.60275200	-2.39921300	1.10726500
H	3.53516200	-1.53999900	1.78208700
H	2.94765000	-3.18021400	1.50830300
H	4.63201800	-2.77818600	1.14221900
H	3.11813300	2.28074000	-0.28555300
H	2.54291900	1.20442100	-1.54489200
O	0.68359400	2.76344000	0.56336500
O	-0.37089900	0.77522300	0.21945100
C	-1.66043200	1.45838200	0.28204300
H	-1.73694800	1.94277700	1.25932000
H	-1.68116100	2.23396800	-0.48793200
C	-2.74042300	0.43331200	0.07444200
C	-3.32071800	0.25478300	-1.18792100
C	-3.17860800	-0.36195500	1.14275200
C	-4.32013300	-0.70202800	-1.38240000
H	-2.98954300	0.86868800	-2.02177400
C	-4.17456900	-1.32096900	0.95160200
H	-2.73505300	-0.22931600	2.12619400
C	-4.74781100	-1.49220500	-0.31241300
H	-4.76402500	-0.82873600	-2.36556200
H	-4.50605600	-1.93063400	1.78712300
H	-5.52606800	-2.23517400	-0.46110700
H	3.17915200	-2.93738800	-0.93962200

29

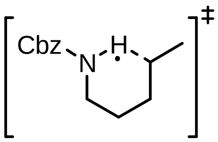

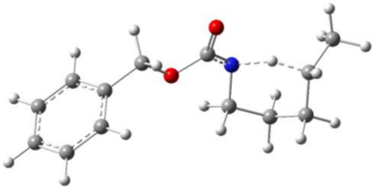

Cartesian Coordinates

C	1.71495800	1.25393800	0.50891900
C	3.10549100	1.90552800	0.49171800
H	2.98995000	2.99263900	0.42196500
H	3.61667300	1.69509200	1.43911900
C	3.94526100	1.37770100	-0.69243200
H	4.93213900	1.86345800	-0.68598300
H	3.45499300	1.66865100	-1.62986400
C	4.10092500	-0.13919500	-0.63544400
H	3.03394600	-0.46326100	-0.06085200
N	1.86277100	-0.19618000	0.60340400
C	0.88952400	-1.04232200	0.11894900
C	5.21014800	-0.68995100	0.24111400
H	5.17777300	-0.26711800	1.25100500
H	5.14418000	-1.77861000	0.32909300
H	6.19306700	-0.44759300	-0.18852000
H	1.14069400	1.55712000	-0.37754300
H	1.15684900	1.59833500	1.39029800
O	-0.34819900	-0.46753500	0.16256400
O	1.08641400	-2.18923100	-0.25299800
C	-1.45483000	-1.34163400	-0.19119500
H	-1.27918900	-1.73892600	-1.19426500
H	-1.46838200	-2.18205800	0.50900300
C	-2.72292100	-0.53437600	-0.12202700
C	-3.23471900	0.08760200	-1.26868200

C	-3.40706800	-0.38449500	1.09235600
C	-4.40477200	0.84819700	-1.20473800
H	-2.71419800	-0.02694500	-2.21625700
C	-4.57606500	0.37595500	1.16117100
H	-3.02042700	-0.86655600	1.98670800
C	-5.07731300	0.99428200	0.01159400
H	-4.79214300	1.32202200	-2.10217400
H	-5.09764100	0.48224200	2.10806500
H	-5.98911800	1.58242600	0.06293600
H	4.04400500	-0.63090500	-1.61091800

30	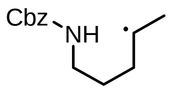	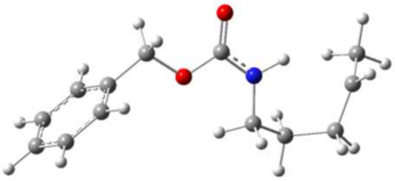

Cartesian Coordinates

C	1.72948700	-1.04985600	0.29707800
C	2.98485300	-1.73483700	-0.25325600
H	2.80094600	-2.81609500	-0.23073800
H	3.11397100	-1.46047700	-1.30745000
C	4.29199100	-1.45690700	0.53647300
H	5.03863300	-2.19128000	0.18540500
H	4.11422900	-1.68537800	1.59486300
C	4.86923500	-0.07750500	0.40293100
N	1.78165000	0.40115600	0.13532700
C	0.71812500	1.25149600	0.11958000
C	5.48603900	0.38858200	-0.87653600
H	4.87134200	0.13691300	-1.75029600
H	5.64976200	1.47070700	-0.88079200
H	6.46896300	-0.08703800	-1.04495200
H	0.84850400	-1.41480000	-0.23515300
H	1.59845400	-1.31818500	1.35723900
O	0.80618400	2.47208200	0.11469900
O	-0.46583500	0.57768600	0.08801700
C	-1.65011800	1.41433000	0.01573800
H	-1.66970700	2.06888200	0.89128300
H	-1.57926700	2.04321700	-0.87598700
C	-2.85214300	0.50936800	-0.03269900
C	-3.32865200	0.02423500	-1.25845800
C	-3.50856400	0.13094800	1.14608600
C	-4.43498800	-0.82655700	-1.30603400
H	-2.82965200	0.31641200	-2.17907100
C	-4.61622700	-0.71913600	1.10329200
H	-3.14984700	0.50578000	2.10144200
C	-5.08113800	-1.20022700	-0.12387500
H	-4.79529700	-1.19309300	-2.26303200
H	-5.11740300	-1.00185700	2.02462800
H	-5.94475400	-1.85814300	-0.15951500
H	2.67385400	0.85944400	0.27428800
H	5.06553100	0.49514400	1.30689800

31	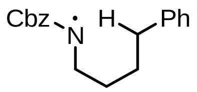	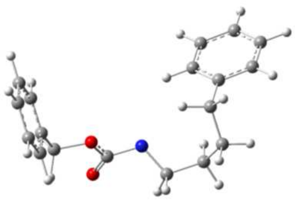

Cartesian Coordinates

C	-1.02256400	-2.53350300	-0.23978400
C	-2.48049200	-2.27873400	-0.62770400
H	-2.91681200	-3.25510000	-0.87483600
H	-3.02460100	-1.90762400	0.24859500
C	-2.69219000	-1.33815900	-1.82783500
H	-3.72055800	-1.46698900	-2.18853200
H	-2.03788900	-1.65901600	-2.65130200
C	-2.45174500	0.16803200	-1.56622700
H	-1.43443400	0.32100000	-1.19355300
N	-0.37830900	-1.40315900	0.39026300
C	0.88516000	-1.65154100	0.92002500
H	-0.94822000	-3.38403200	0.45850000
H	-0.43478900	-2.83565100	-1.12686500
O	1.11334400	-2.51513700	1.75335500
O	1.78302400	-0.76338900	0.46231600
C	3.12731400	-0.87384900	1.02583500
H	3.05598500	-0.69618300	2.10228900
H	3.48487000	-1.89471100	0.86893700
C	4.00313100	0.13967200	0.34408200
C	4.75404500	-0.21413700	-0.78480500
C	4.07666800	1.45426300	0.82473600
C	5.56169500	0.72830200	-1.42524400
H	4.70706100	-1.23263000	-1.16216600
C	4.88224300	2.39936800	0.18667700
H	3.49952100	1.73742900	1.70126000
C	5.62646800	2.03738800	-0.94009000
H	6.14108200	0.44099700	-2.29785500
H	4.93194400	3.41455000	0.56945200
H	6.25658600	2.77079700	-1.43487000
H	-2.51816400	0.68842500	-2.53028800
C	-3.44243800	0.78943300	-0.60113500
C	-4.74005200	1.11967400	-1.02429100
C	-3.09639800	1.04254700	0.73464300
C	-5.66623500	1.67989000	-0.14170600
H	-5.02589700	0.94401400	-2.05946500
C	-4.01985100	1.60444400	1.62181000
H	-2.09647300	0.79174000	1.07756900
C	-5.30862400	1.92428500	1.18790300
H	-6.66318900	1.93211400	-0.49295400
H	-3.72983600	1.79362700	2.65188200
H	-6.02577900	2.36331600	1.87567200

32

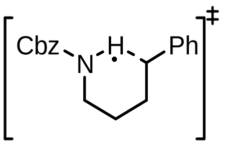

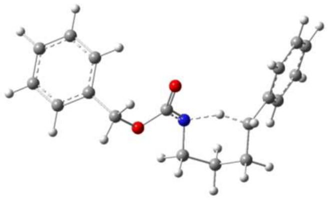

Cartesian Coordinates

C	0.40197600	-2.26186900	-0.65316000
C	1.86871700	-2.55949600	-0.99530800
H	1.95064400	-3.59980400	-1.32971700
H	2.17468200	-1.92267600	-1.83356300
C	2.78871000	-2.31953300	0.22268800
H	3.82692500	-2.55252600	-0.04482200
H	2.50620900	-3.02553000	1.01296100
C	2.67195200	-0.89166500	0.76851000
H	1.56039100	-0.57492100	0.39221800
N	0.27015400	-0.87024100	-0.23973800

C	-0.72577800	-0.49215300	0.63401800
H	0.03511500	-2.96348200	0.10900200
H	-0.21775700	-2.40560100	-1.55042000
O	-1.84233200	-1.26518100	0.49657600
O	-0.64146600	0.45297500	1.40499100
C	-3.02286000	-0.85633700	1.24596800
H	-3.51908600	-1.79729900	1.49315800
H	-2.69437000	-0.35880000	2.16005700
C	-3.92644100	0.03030200	0.42557200
C	-4.96872600	-0.52542600	-0.32913900
C	-3.73402700	1.42006200	0.40064600
C	-5.80378600	0.28790200	-1.09888500
H	-5.12736700	-1.60112800	-0.31265000
C	-4.56643900	2.23430400	-0.37034600
H	-2.92470500	1.85462400	0.97993400
C	-5.60277000	1.67075800	-1.12101500
H	-6.60935700	-0.15583700	-1.67705700
H	-4.40774300	3.30887300	-0.38298800
H	-6.25142600	2.30586700	-1.71769600
H	2.57472300	-0.85574200	1.85734900
C	3.58551000	0.17619000	0.27730200
C	4.30207200	0.07833600	-0.93073200
C	3.71966100	1.36057400	1.03069600
C	5.13046000	1.11618500	-1.35976100
H	4.21992700	-0.81618300	-1.53953000
C	4.54417200	2.39849800	0.60215000
H	3.16250900	1.46087000	1.95851100
C	5.25565900	2.28095900	-0.59693300
H	5.67890700	1.01532700	-2.29212700
H	4.63275400	3.29957200	1.20225500
H	5.89993500	3.08818300	-0.93286300

33

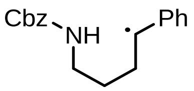

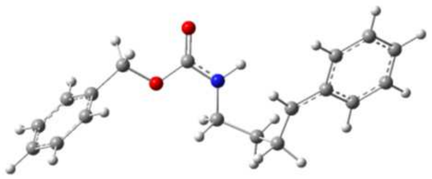

Cartesian Coordinates

C	-0.10804100	1.26530500	-0.95403100
C	-1.42052800	2.03046200	-0.76428900
H	-1.20600000	3.08610800	-0.97283700
H	-2.14992000	1.70995700	-1.52010800
C	-2.05796700	1.93009500	0.64956400
H	-2.80571400	2.73013200	0.73019200
H	-1.28356400	2.15919800	1.39233100
C	-2.68613500	0.60904900	0.98271100
N	-0.29589900	-0.18066300	-0.85563000
C	0.68205100	-1.10977900	-0.66727900
H	0.32155200	1.52947700	-1.93119100
H	0.61848300	1.56931300	-0.19506600
O	0.50284700	-2.31988400	-0.68792000
O	1.88841800	-0.52308500	-0.43291300
C	2.98503200	-1.44265100	-0.18412600
H	2.72629800	-2.07199700	0.67157700
H	3.10149600	-2.08934600	-1.05810000
C	4.22213600	-0.62571500	0.07645500
C	5.04377800	-0.21954800	-0.98397300
C	4.56709200	-0.25156000	1.38218400
C	6.18445000	0.55021200	-0.74605500

H	4.78752600	-0.50954600	-1.99987800
C	5.70753700	0.51762400	1.62496700
H	3.93957900	-0.56694400	2.21208400
C	6.51826000	0.92055200	0.56005300
H	6.81402000	0.85621100	-1.57669100
H	5.96507700	0.79781800	2.64236000
H	7.40776900	1.51530000	0.74707500
H	-1.18880200	-0.57591500	-1.11479400
H	-2.08545900	-0.10960600	1.53399000
C	-3.99179600	0.20161800	0.60732800
C	-4.89249900	1.03407300	-0.12391400
C	-4.45940200	-1.10051700	0.96355100
C	-6.16346100	0.58964500	-0.46741500
H	-4.58411700	2.03304300	-0.41557600
C	-5.72989100	-1.53397800	0.61443900
H	-3.79496000	-1.75827200	1.51790500
C	-6.59556200	-0.69428600	-0.10459300
H	-6.82784100	1.24600900	-1.02296800
H	-6.05500200	-2.53068300	0.89951800
H	-7.58957200	-1.03546700	-0.37720100

34	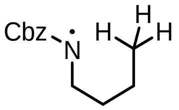	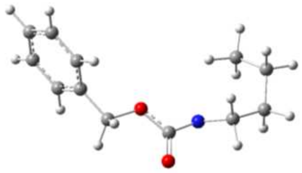

Cartesian Coordinates

C	3.23792300	0.67560200	-0.50389700
C	4.46613500	-0.16222400	-0.14279000
H	5.30540200	0.22014600	-0.73829100
H	4.71725200	0.02143200	0.90977500
C	4.33245500	-1.67390100	-0.39994300
H	5.32842800	-2.12460100	-0.29940500
H	4.03721900	-1.83037700	-1.44787300
C	3.35639300	-2.41270400	0.52459200
H	2.33383700	-2.03589200	0.42947800
N	2.15735600	0.55310200	0.44821100
C	1.05667400	1.37261700	0.21777500
H	3.49897900	1.74644100	-0.55480400
H	2.87613100	0.41843100	-1.51634400
O	1.12179900	2.58982600	0.13368000
O	-0.08086500	0.65748500	0.19337300
C	-1.31321900	1.43075700	0.06097600
H	-1.38733800	2.09573700	0.92579900
H	-1.24208400	2.04583400	-0.83979300
C	-2.46275700	0.46451000	-0.00754000
C	-2.96012400	0.03714400	-1.24569700
C	-3.04743100	-0.02918600	1.16690400
C	-4.02046300	-0.86974900	-1.31137000
H	-2.51556400	0.41765000	-2.16193700
C	-4.10635400	-0.93685700	1.10530600
H	-2.66920500	0.29803100	2.13199600
C	-4.59464700	-1.35896100	-0.13497800
H	-4.39872800	-1.19126300	-2.27741900
H	-4.55178400	-1.31161200	2.02226300
H	-5.42103700	-2.06215600	-0.18393200
H	3.34537600	-3.48420800	0.29549200
H	3.64665900	-2.29609900	1.57505100

35

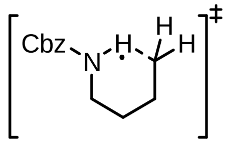

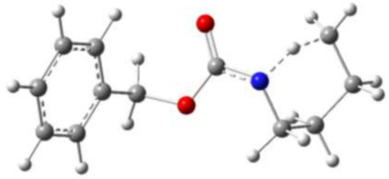

Cartesian Coordinates

C	2.50111100	-1.13484500	0.84276800
C	3.74409000	-1.21213000	-0.06946300
H	4.23040400	-2.18621600	0.05998800
H	3.42739600	-1.14343200	-1.11866100
C	4.72282200	-0.06854800	0.26085000
H	5.58568500	-0.11902800	-0.41965100
H	5.11407800	-0.21109500	1.27618300
C	4.04175600	1.28302700	0.15094900
H	2.86447800	1.02804700	0.60074000
N	1.85723800	0.17472800	0.77295800
C	0.91222500	0.46258700	-0.19148200
H	2.80025800	-1.30418500	1.88403800
H	1.77677000	-1.90664700	0.56515900
O	0.17357600	-0.63393100	-0.53155900
O	0.71508900	1.57998400	-0.64466400
C	-0.97755900	-0.40650900	-1.39447000
H	-0.76014200	0.44503000	-2.04130200
H	-1.04483500	-1.31885600	-1.99105200
C	-2.23991000	-0.18902000	-0.59762900
C	-3.06533400	-1.27299500	-0.26814000
C	-2.60202100	1.09722400	-0.16953100
C	-4.22985100	-1.08082700	0.47892300
H	-2.79471200	-2.27265400	-0.59986100
C	-3.76512300	1.29082100	0.57886300
H	-1.96185700	1.93868200	-0.41733000
C	-4.58127000	0.20321400	0.90443700
H	-4.86179800	-1.92943100	0.72514300
H	-4.03538500	2.29081600	0.90598400
H	-5.48719400	0.35578700	1.48422200
H	4.42388200	2.07789800	0.79449900
H	3.84738100	1.63177700	-0.86546100

36

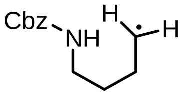

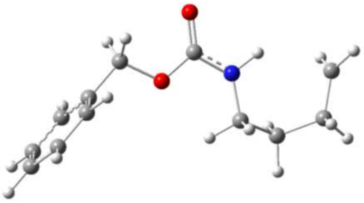

Cartesian Coordinates

C	2.15389100	-0.96923000	0.11471000
C	3.41502200	-1.63672400	-0.44586600
H	3.24941800	-2.72019500	-0.40674500
H	3.52672100	-1.37164100	-1.50536900
C	4.72349900	-1.31774100	0.31292500
H	5.47550800	-2.06438100	-0.00370000
H	4.57645800	-1.49320400	1.38723100
C	5.27995000	0.04992900	0.08689200
N	2.17574600	0.48170000	-0.05297500
C	1.09332900	1.30928600	-0.03741200
H	1.27423400	-1.35327500	-0.40608700
H	2.03990800	-1.23630900	1.17706400

O	1.15506800	2.53116600	-0.04093500
O	-0.07560200	0.60958800	-0.03839400
C	-1.27995600	1.41978000	-0.07795900
H	-1.26475800	2.10909100	0.77032500
H	-1.27186500	2.01264000	-0.99691800
C	-2.46283800	0.49038000	-0.02390100
C	-3.03069800	-0.01104700	-1.20296800
C	-3.00919100	0.10467600	1.20785500
C	-4.11936500	-0.88478100	-1.15364700
H	-2.61780700	0.28641800	-2.16367700
C	-4.09750300	-0.76889900	1.26204500
H	-2.57930200	0.49257200	2.12796900
C	-4.65439300	-1.26608700	0.08012900
H	-4.55166500	-1.26355200	-2.07540200
H	-4.51295700	-1.05716500	2.22343700
H	-5.50372600	-1.94209400	0.12020500
H	3.05851800	0.96127900	0.07373000
H	5.83079600	0.56699500	0.86624400
H	5.33409300	0.45765000	-0.91967000

37

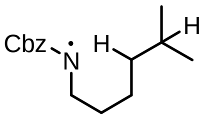

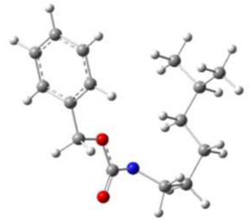

Cartesian Coordinates

C	2.24091500	2.24708200	-0.40705000
C	3.63918700	1.74745000	-0.03768600
H	4.36061700	2.38266500	-0.56776200
H	3.79893500	1.91591500	1.03491700
C	3.92769200	0.27763500	-0.39470500
H	5.00725900	0.10492200	-0.28188300
H	3.70911400	0.12902100	-1.46116200
C	3.17393600	-0.75850800	0.45279000
H	2.08992800	-0.63243900	0.33092100
N	1.20801500	1.77504300	0.48688000
C	-0.07735300	2.23356500	0.21594500
C	3.54481800	-2.22662500	0.15634000
H	2.20304300	3.34956000	-0.37563000
H	1.99422100	1.98099400	-1.45079900
O	-0.37345600	3.41503300	0.11670400
O	-0.95193700	1.21381900	0.17024000
C	-2.35085200	1.58754500	-0.02197300
H	-2.64273800	2.24307400	0.80263500
H	-2.43239100	2.15185700	-0.95487600
C	-3.16800800	0.32613800	-0.05497000
C	-3.41432100	-0.33095000	-1.26796800
C	-3.68777000	-0.21493500	1.12873600
C	-4.16252400	-1.50971600	-1.29845100
H	-3.01876800	0.08339300	-2.19196300
C	-4.43689500	-1.39306800	1.10224400
H	-3.50300800	0.28885300	2.07406300
C	-4.67495900	-2.04286800	-0.11228700
H	-4.34818300	-2.00869800	-2.24516400
H	-4.83591000	-1.80136400	2.02621900
H	-5.26030700	-2.95755700	-0.13477900
H	3.37689600	-0.55088700	1.51265400

C	2.91621000	-3.16106100	1.20222300
H	1.82089600	-3.10446700	1.16790800
H	3.20122400	-4.20461100	1.02521200
H	3.23113100	-2.89407600	2.21744400
C	3.14032000	-2.66121000	-1.26201900
H	3.40360500	-3.71011700	-1.44005900
H	2.05600300	-2.56235900	-1.40197800
H	3.63375300	-2.06356900	-2.03535400
H	4.63934300	-2.31907300	0.23872200

38	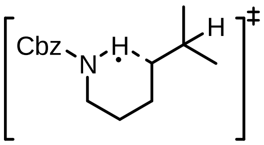	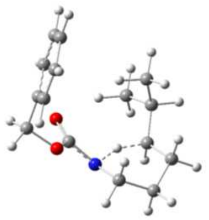

Cartesian Coordinates

C	1.49524200	-2.13217400	0.59804900
C	2.89291600	-2.05884100	1.23181300
H	2.89180200	-2.63837400	2.16146000
H	3.62141500	-2.52597300	0.55694700
C	3.29850400	-0.59723600	1.50706800
H	4.28994500	-0.57390800	1.98339200
H	2.59478200	-0.16044700	2.22604500
C	3.32031200	0.21092900	0.21792500
H	2.38356500	-0.33409900	-0.42495800
N	1.48329100	-1.35749700	-0.63941500
C	0.31790400	-0.84323600	-1.15211000
C	3.14424100	1.72632300	0.25979900
H	0.73532400	-1.78482500	1.31180800
H	1.25784800	-3.17590700	0.35114800
O	-0.75929900	-1.61468500	-0.82040200
O	0.24960600	0.14501600	-1.87093900
C	-2.03831400	-1.24052000	-1.40690300
H	-1.85059900	-0.76154600	-2.36930300
H	-2.54965900	-2.19340700	-1.55960500
C	-2.83259700	-0.34277200	-0.49033100
C	-3.70074100	-0.89396700	0.46263000
C	-2.71245700	1.05253400	-0.57411800
C	-4.43258200	-0.07041400	1.32137900
H	-3.80486500	-1.97429900	0.53036500
C	-3.44423800	1.87769100	0.28305900
H	-2.03541100	1.48207700	-1.30631100
C	-4.30467700	1.31869600	1.23279800
H	-5.10381600	-0.51100700	2.05312900
H	-3.34481100	2.95689300	0.20775000
H	-4.87541200	1.96178100	1.89677900
H	4.16139300	-0.08366000	-0.42297400
C	3.33262200	2.33436700	-1.13996400
H	2.55160700	1.97828000	-1.82065000
H	3.27719400	3.42780000	-1.10075500
H	4.30594100	2.06298600	-1.56457700
C	1.80507700	2.17507300	0.87029000
H	0.96783400	1.83681600	0.25069700
H	1.65914100	1.79305900	1.88567900
H	1.76100000	3.26832900	0.92425100
H	3.95301900	2.11054600	0.90800200

39

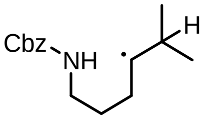

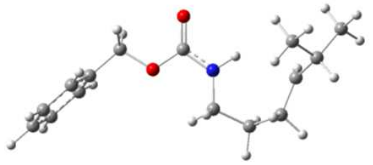

Cartesian Coordinates

C	0.88929700	-1.32012800	0.01922200
C	2.04931200	-2.20541100	-0.45076900
H	1.78441700	-3.23867100	-0.19571900
H	2.11977300	-2.16050100	-1.54559400
C	3.42417300	-1.88607200	0.17304800
H	4.07921600	-2.76225900	0.00290700
H	3.32212300	-1.82110200	1.26676800
C	4.11533100	-0.66581600	-0.35416100
N	1.03663500	0.06320500	-0.42728400
C	0.04653800	0.99644500	-0.48810100
C	5.18209900	0.04417100	0.43338400
H	-0.05097900	-1.70499200	-0.38162800
H	0.81314600	-1.36819500	1.11676200
O	0.22439900	2.18255200	-0.73131200
O	-1.17877500	0.44419300	-0.26446300
C	-2.29844500	1.36286700	-0.36459200
H	-2.14652600	2.17831300	0.34754700
H	-2.31093500	1.78977900	-1.37130800
C	-3.55654300	0.59031200	-0.07042900
C	-4.25243900	-0.05689800	-1.10058100
C	-4.04713400	0.49897700	1.23915600
C	-5.41246300	-0.78549400	-0.82821200
H	-3.88296400	0.01232800	-2.12067400
C	-5.20704600	-0.22829000	1.51627800
H	-3.51733400	1.00160600	2.04450100
C	-5.89182400	-0.87276200	0.48205100
H	-5.94356300	-1.27984400	-1.63663900
H	-5.57765400	-0.28837800	2.53559700
H	-6.79617900	-1.43556800	0.69502100
H	1.97062400	0.45218300	-0.46245700
H	4.02487500	-0.45197700	-1.41987100
C	6.10226600	0.87492800	-0.47516100
H	5.53303400	1.65604300	-0.99367100
H	6.89039300	1.36690700	0.10507400
H	6.58230000	0.24822700	-1.23461900
C	4.58555100	0.92391300	1.55924000
H	4.00659000	1.75378800	1.13814300
H	3.92288000	0.34825300	2.21376700
H	5.38338500	1.34811600	2.17989200
H	5.79990200	-0.72491100	0.93115700

40

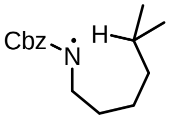

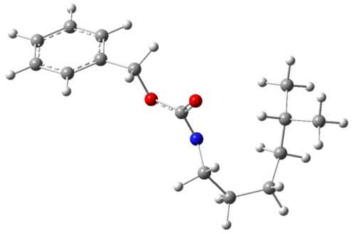

Cartesian Coordinates

C	1.84401800	-1.98916500	0.05391400
C	3.03598600	-2.22219700	-0.87934700

H	3.36665500	-3.25700100	-0.71935300
H	2.67874300	-2.16747100	-1.91574200
C	4.25716100	-1.29891300	-0.70004900
H	5.06394500	-1.72867000	-1.30684700
H	4.60638900	-1.36662400	0.33931300
C	4.06504200	0.18327300	-1.10265500
N	1.03950000	-0.84453100	-0.30703400
C	0.15597300	-0.39602100	0.67026200
H	4.97863600	0.54004300	-1.59944600
H	3.26788300	0.24521600	-1.85385600
C	3.74730800	1.16541400	0.04669000
H	2.93693300	0.74349700	0.65474500
H	1.17839100	-2.87242200	0.01644500
H	2.15622200	-1.90253200	1.10722000
C	3.25346200	2.50638200	-0.51740500
H	3.02058600	3.21468600	0.28595000
H	4.01812900	2.96790800	-1.15623000
H	2.34943700	2.37217500	-1.12175000
C	4.96043600	1.38452300	0.96585300
H	5.78959500	1.84377700	0.41143700
H	4.70651500	2.05007600	1.79859600
H	5.32905500	0.44743600	1.39723900
O	-1.09335300	-0.30169900	0.18649400
O	0.50098500	-0.05541000	1.79258400
C	-2.08661600	0.25461400	1.10225400
H	-2.09435700	-0.34781700	2.01431500
H	-1.77151700	1.26854900	1.36335300
C	-3.42018900	0.23995600	0.40881400
C	-3.78392800	1.28440100	-0.45254500
C	-4.31593400	-0.81842100	0.60962400
C	-5.01819700	1.26946300	-1.10442900
H	-3.09584500	2.11035200	-0.61320800
C	-5.55284900	-0.83581900	-0.03940800
H	-4.04412800	-1.63113900	1.27864400
C	-5.90536400	0.20851400	-0.89837300
H	-5.28917300	2.08498100	-1.76861000
H	-6.23980000	-1.66046900	0.12711700
H	-6.86770300	0.19802300	-1.40192100

41

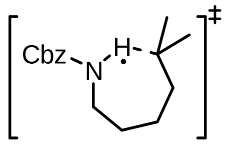

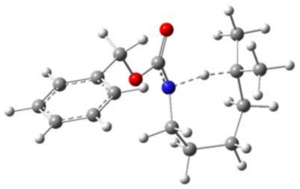

Cartesian Coordinates

C	-1.16236500	1.47095200	-0.35583300
C	-2.40232400	2.34141200	-0.60105200
H	-2.09362000	3.38356600	-0.45005000
H	-2.69475400	2.25054700	-1.65557100
C	-3.62011500	2.06861200	0.30511700
H	-4.31897600	2.90656000	0.18856700
H	-3.30138000	2.09298300	1.35613400
C	-4.39680600	0.77047700	0.01227700
N	-1.35236900	0.10212900	-0.83036800
C	-0.41884500	-0.86616700	-0.52123800
H	-5.32578600	0.76863000	0.60470200
H	-4.70746500	0.78257900	-1.04101100
C	-3.65021900	-0.53888100	0.28731200
H	-2.58769700	-0.37093900	-0.28306700

H	-0.31159500	1.88210600	-0.91564000
H	-0.87734200	1.50573000	0.70705800
C	-4.26370300	-1.74666900	-0.40930500
H	-3.63890200	-2.63510900	-0.28064000
H	-5.25751300	-1.96195900	0.01177000
H	-4.38734600	-1.56840300	-1.48251000
C	-3.32751600	-0.80096700	1.75334800
H	-4.25486100	-0.98029600	2.31718500
H	-2.69606000	-1.68854400	1.85961600
H	-2.81426700	0.04123600	2.22850100
O	0.83331000	-0.34479400	-0.37619800
O	-0.66058200	-2.06194300	-0.44098900
C	1.90016300	-1.31741300	-0.19844800
H	1.65820500	-1.94446300	0.66357700
H	1.93623800	-1.95741000	-1.08481700
C	3.18603500	-0.56157300	-0.00016600
C	3.98152800	-0.20554600	-1.09776100
C	3.60211500	-0.19212000	1.28625800
C	5.16727900	0.50951200	-0.91516600
H	3.66927300	-0.49080400	-2.09921000
C	4.78693500	0.52316100	1.47344300
H	2.99441200	-0.46803200	2.14444100
C	5.57179400	0.87595000	0.37178700
H	5.77577600	0.77684200	-1.77432700
H	5.09914100	0.80074400	2.47615100
H	6.49554500	1.42899100	0.51577500

42	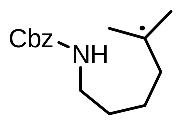	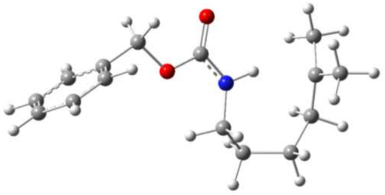

Cartesian Coordinates

C	-1.07747200	1.34115800	0.13530100
C	-2.19003400	2.21413500	-0.46160800
H	-1.85646300	3.25187200	-0.33413700
H	-2.24169000	2.03888300	-1.54471100
C	-3.60288800	2.10522700	0.15617400
H	-4.11972300	3.05004400	-0.05239900
H	-3.51989300	2.05227100	1.24994300
C	-4.52362900	0.97494900	-0.36869500
N	-1.22772000	-0.07286400	-0.19436100
C	-0.23613000	-1.00467600	-0.21461000
H	-5.56205200	1.26481500	-0.11376800
H	-4.48397500	0.97635900	-1.46592800
C	-4.30295200	-0.42804400	0.13658000
H	-2.16315100	-0.46887900	-0.13964200
H	-0.11300400	1.67078000	-0.25551400
H	-1.04637900	1.48502100	1.22720000
C	-4.61983700	-1.56952100	-0.78508800
H	-4.15629700	-2.50432300	-0.45049900
H	-5.70970900	-1.75419600	-0.83058000
H	-4.29035600	-1.37238800	-1.81127100
C	-4.38814000	-0.69314500	1.61182600
H	-5.44296400	-0.75261000	1.93945600
H	-3.92249900	-1.64879600	1.87733300
H	-3.91855500	0.09206000	2.21308900
O	0.99873000	-0.43754200	-0.10205700
O	-0.41877900	-2.20923000	-0.33965200

C	2.11272700	-1.36480800	-0.17525700
H	2.00863000	-2.10143700	0.62601400
H	2.06465900	-1.89487000	-1.13052400
C	3.38431600	-0.56998200	-0.04148700
C	4.01027200	-0.03142600	-1.17404500
C	3.95770700	-0.34885100	1.21798300
C	5.18239400	0.71795800	-1.05138000
H	3.57635000	-0.20204600	-2.15614100
C	5.13020700	0.39951700	1.34555400
H	3.48225000	-0.76631600	2.10202700
C	5.74458800	0.93515600	0.21001000
H	5.65855000	1.12721700	-1.93783000
H	5.56533000	0.56073800	2.32774000
H	6.65858800	1.51413300	0.30680500

43

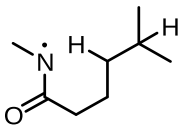

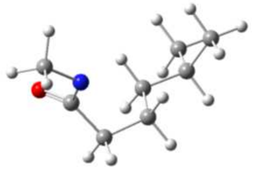

Cartesian Coordinates

C	-1.90177700	-0.48034600	-0.02504100
C	-1.04742300	-0.97011300	1.12364700
H	-1.59494000	-0.79893700	2.05921700
H	-0.91329700	-2.04893800	0.99405800
C	0.32390800	-0.25989100	1.21079300
H	0.81971800	-0.60809600	2.12675300
H	0.15167600	0.81448300	1.33422500
C	1.23718300	-0.52998600	0.00690300
H	0.77017800	-0.14554600	-0.91058400
N	-2.45360700	0.78131000	0.13748800
C	-3.71309300	1.05862400	-0.50173700
C	2.65668200	0.06098000	0.13006100
H	1.32053500	-1.61701500	-0.13148000
C	3.54970200	-0.44916900	-1.01212200
H	3.16058200	-0.12620600	-1.98603400
H	4.57194500	-0.06523000	-0.91884200
H	3.60115800	-1.54382900	-1.02130700
H	-4.10427500	0.21707800	-1.08374800
H	-4.43015700	1.33721500	0.28556300
H	-3.60866000	1.94528400	-1.13952300
O	-2.02402100	-1.08603200	-1.09315100
H	3.08708400	-0.29891600	1.07774000
C	2.65488700	1.59816100	0.16586200
H	3.67625300	1.98799000	0.24242500
H	2.21094000	2.00691100	-0.75098500
H	2.08827700	1.99328300	1.01512200

44

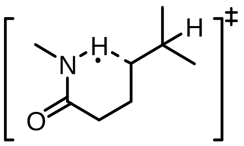

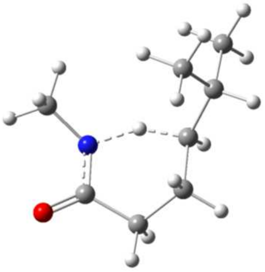

Cartesian Coordinates

C	-1.98646700	-0.15030700	-0.06288500

C	-1.48472700	-1.57712000	-0.27454800
H	-2.14786400	-2.24688900	0.27686800
H	-1.57162200	-1.82124300	-1.34148500
C	-0.01756800	-1.72612700	0.18092700
H	0.33536700	-2.74731100	-0.02114600
H	0.03134300	-1.58901700	1.26750500
C	0.87438400	-0.70928500	-0.52263500
H	0.08073900	0.27408700	-0.61123200
N	-1.17525300	0.82036800	-0.59695300
C	-1.27135800	2.18670800	-0.10993100
C	2.20047800	-0.30817600	0.11613300
H	0.98596700	-0.94446700	-1.58896600
C	3.04029500	0.54979600	-0.84382500
H	2.53840800	1.50111200	-1.05866900
H	4.01937400	0.78098600	-0.41077500
H	3.20684600	0.03685600	-1.79745600
C	2.03684800	0.39493400	1.47530400
H	1.51422300	1.35161200	1.35784100
H	1.47307300	-0.20958600	2.19220400
H	3.01594100	0.60332000	1.91942600
H	2.75396300	-1.24755500	0.29477000
H	-0.45764300	2.77570000	-0.54092600
H	-2.21997400	2.61336000	-0.45604300
H	-1.25002500	2.26039500	0.98457200
O	-3.05408300	0.11294100	0.49511800

45

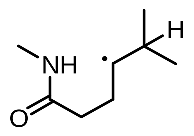

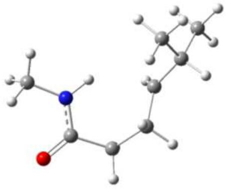

Cartesian Coordinates

C	-2.11208200	-0.41362800	-0.04735900
C	-1.15195100	-1.60414200	0.00105500
H	-1.67149900	-2.36589500	0.58880400
H	-1.08680400	-2.00361200	-1.02061400
C	0.26532700	-1.37914200	0.56046000
H	0.69755300	-2.37678400	0.76523000
H	0.20431800	-0.89811200	1.54808700
C	1.20726500	-0.62765500	-0.32980100
N	-1.56005700	0.82976700	-0.11694100
C	-2.38525300	2.02301500	-0.23490000
C	2.52408400	-0.10489600	0.17615200
H	-0.55577800	0.90910300	-0.21144500
H	1.04869500	-0.68549600	-1.40726800
C	3.53400000	0.09337300	-0.96540200
H	3.16586600	0.83529500	-1.68488600
H	4.49684700	0.44930700	-0.58332800
H	3.70990800	-0.84175900	-1.50770700
H	-3.16923600	2.00988500	0.52622900
H	-1.75570100	2.90354600	-0.08955300
H	-2.87009300	2.08438800	-1.21622800
O	-3.33104900	-0.58565500	-0.03554600
H	2.93836100	-0.85772900	0.87001000
C	2.35981000	1.19933100	0.99438700
H	3.31677900	1.50201400	1.43482000
H	2.01215700	2.01796500	0.35272800
H	1.64021700	1.07722300	1.81051900

46	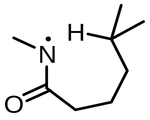	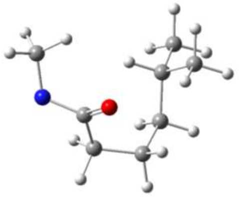

Cartesian Coordinates

C	1.59084600	0.42391000	0.14881300
C	0.81058700	1.53270000	-0.53289700
H	1.45491400	2.42062600	-0.46347000
H	0.73382700	1.30804000	-1.60401400
C	-0.56590900	1.83457300	0.08442300
H	-0.81195000	2.87966200	-0.13764000
H	-0.48876900	1.76173700	1.17434100
C	-1.71773400	0.96053400	-0.44615100
N	2.53483200	-0.20764400	-0.64609500
O	1.52215000	0.18032300	1.35541800
C	2.83841100	-1.58872100	-0.37655100
H	2.32815900	-1.98031800	0.51033900
H	2.54375800	-2.17361900	-1.26189800
H	3.92370600	-1.71106800	-0.27971100
H	-2.66453900	1.36790900	-0.06263900
H	-1.76175200	1.07554600	-1.53917500
C	-1.67589800	-0.54475700	-0.11102000
H	-0.71039200	-0.95024400	-0.45123800
C	-1.79699200	-0.81189700	1.39741200
C	-2.77791500	-1.28842300	-0.88356600
H	-2.74684100	-2.36498300	-0.68131400
H	-3.77134200	-0.92502300	-0.59080600
H	-2.67718000	-1.14605100	-1.96579500
H	-0.97822500	-0.35700500	1.96163400
H	-2.74449400	-0.41371300	1.78354100
H	-1.78227500	-1.88809000	1.60439000

47	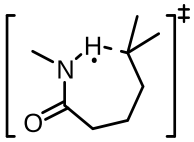	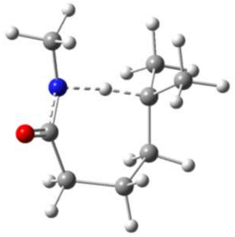

Cartesian Coordinates

C	1.71761100	-0.18567500	0.13991400
C	1.17515500	-1.57998400	0.43437600
H	1.97875400	-2.27202600	0.16968100
H	1.00207400	-1.66655900	1.51484500
C	-0.11392700	-1.98035200	-0.32081800
H	-0.22180300	-3.06763500	-0.22949800
H	0.01115800	-1.78145000	-1.39259100
C	-1.40482900	-1.32808400	0.20497000
N	0.95233100	0.87848100	0.56103400
O	2.82853700	-0.01523600	-0.37033800
C	1.27179800	2.19803400	0.03718700
H	1.46380600	2.20203900	-1.04283700
H	0.45104100	2.87903000	0.27863700
H	2.17414300	2.56957000	0.53682600
H	-2.27033600	-1.80969300	-0.27790000
H	-1.49268100	-1.54672000	1.27756800

C	-1.51711700	0.18426600	-0.00621600
H	-0.40155800	0.58519600	0.34753400
C	-2.50135000	0.85934400	0.94053100
C	-1.69737100	0.60652300	-1.45947900
H	-2.26182000	0.64660000	1.98754900
H	-2.51208000	1.94618200	0.80483300
H	-3.52249400	0.49529800	0.75172100
H	-0.93619500	0.17551800	-2.11693900
H	-2.67936500	0.27705000	-1.82951500
H	-1.65708100	1.69567900	-1.56723200

48

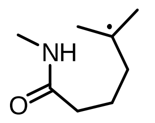

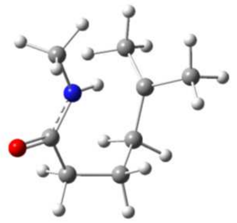

Cartesian Coordinates

C	1.85749700	-0.30975200	0.09595300
C	1.05823600	-1.60822600	0.23480200
H	1.75411500	-2.38378000	-0.09734400
H	0.91501200	-1.78102100	1.31108300
C	-0.28816300	-1.78127900	-0.50327200
H	-0.42735500	-2.85897900	-0.64748100
H	-0.22124400	-1.35477700	-1.51174900
C	-1.55758400	-1.25627200	0.22034000
N	1.16300700	0.84314500	-0.09906800
O	3.08713300	-0.32147000	0.17641500
C	1.83875900	2.12976800	-0.17543400
H	2.63282900	2.09508200	-0.92608500
H	1.11043100	2.89335500	-0.45749600
H	2.29529000	2.40348400	0.78280600
H	-2.41434900	-1.83491000	-0.17226200
H	-1.48516000	-1.51924600	1.28356300
C	-1.89297600	0.20767600	0.08073900
H	0.14734500	0.81604700	-0.06567800
C	-2.38909100	0.94823900	1.28801600
C	-2.32138700	0.70989600	-1.26878500
H	-2.37139600	2.03423800	1.13984800
H	-3.43744800	0.68092800	1.51764100
H	-1.80333700	0.71217300	2.18365500
H	-1.74817000	0.26036000	-2.08648900
H	-3.38367200	0.46693600	-1.45665400
H	-2.23275100	1.79955200	-1.34935200

49

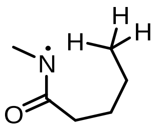

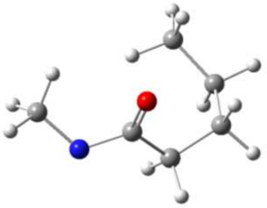

Cartesian Coordinates

C	0.83500700	-0.53253200	-0.17205500
C	-0.33148300	-1.17790400	0.55427500
H	-0.09369700	-2.25048800	0.58602500
H	-0.32891100	-0.84561200	1.60003400
C	-1.70169700	-0.96123000	-0.10975500
H	-2.35109400	-1.80192800	0.16174000

H	-1.57395700	-1.00175200	-1.19772700
C	-2.41142600	0.34449000	0.29020900
N	1.95060400	-0.28240500	0.61318300
O	0.87582100	-0.36818600	-1.39260600
C	2.79022500	0.83365900	0.26426500
H	2.49379400	1.32369200	-0.66969800
H	2.74349500	1.55777900	1.09287000
H	3.83379800	0.50205400	0.20977800
H	-3.40260200	0.35139300	-0.18133300
H	-2.59199000	0.33555700	1.37468900
C	-1.67160200	1.63351100	-0.08918900
H	-0.71236800	1.72313900	0.43399000
H	-1.46585600	1.67357500	-1.16425600
H	-2.26554700	2.51495500	0.17595400

50

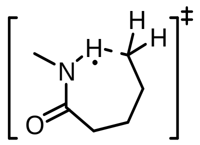

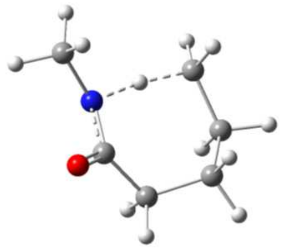

Cartesian Coordinates

C	0.88788500	-0.74278000	0.10225900
C	-0.44262000	-1.43223800	0.39047600
H	-0.28402800	-2.48525200	0.14401400
H	-0.63845900	-1.37086200	1.46908500
C	-1.67680700	-0.90648000	-0.38022600
H	-2.46793000	-1.65951200	-0.28517800
H	-1.44270400	-0.84216100	-1.45159800
C	-2.22298700	0.44369600	0.11638000
N	0.99377900	0.58487400	0.44984300
O	1.85794500	-1.36515800	-0.33623300
C	2.11338600	1.35363400	-0.07630600
H	2.28780900	1.18458700	-1.14576200
H	1.92642900	2.41418900	0.11212300
H	3.02451100	1.06794900	0.46061200
H	-3.19326200	0.63440700	-0.36810300
H	-2.42691500	0.37666000	1.19306700
C	-1.29535500	1.60929800	-0.15901900
H	-0.15484300	1.21395500	0.25868100
H	-1.13809300	1.82295300	-1.22009100
H	-1.49354000	2.51945300	0.41273400

51

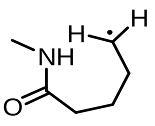

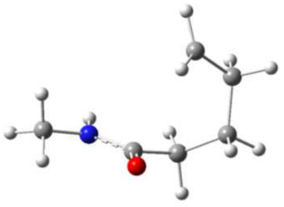

Cartesian Coordinates

C	0.72415300	-0.52509700	-0.14259000
C	-0.46071100	-1.18379200	0.56154000
H	-0.28025400	-2.26463200	0.49859000
H	-0.47902700	-0.92854400	1.62932900
C	-1.81593400	-0.86308000	-0.09091300
H	-2.54232800	-1.62183400	0.22325800
H	-1.70402300	-0.95716300	-1.17697400

C	-2.37956500	0.52714100	0.26104900
N	1.67981400	0.00917200	0.67389100
O	0.83808000	-0.53234800	-1.36764600
C	2.86180900	0.67671200	0.14975400
H	2.66648600	1.73038700	-0.08580600
H	3.66858700	0.61782000	0.88465900
H	3.16993600	0.17408200	-0.76832000
H	-3.40027800	0.58785100	-0.16209100
H	-2.52298400	0.60281500	1.34970900
C	-1.57047000	1.68318600	-0.22549600
H	-1.01496300	1.61732000	-1.15571500
H	-1.65788200	2.65632300	0.24826300
H	1.47769300	0.08973000	1.65896300

7.3 Bond Dissociation Enthalpies

DFT Methods:

Structure optimization: (RO)B3P86/6-311G(d,p)

Frequency calculations: (RO)B3P86/6-311G(d,p) scaled by a factor of 0.9806

No.	Reactions	Kcal mol^{-1}
1	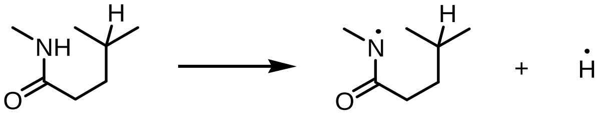	107.1
2	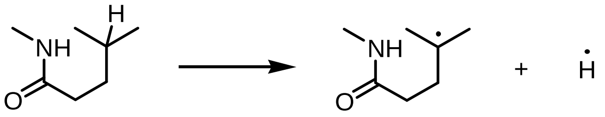	95.4
3	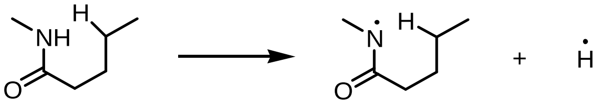	107.7
4	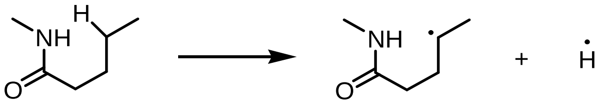	98.0
5	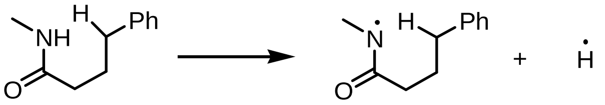	109.1
6	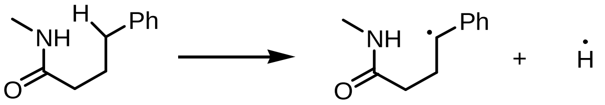	90.0
7	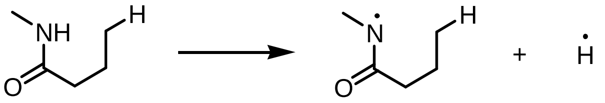	107.7
8	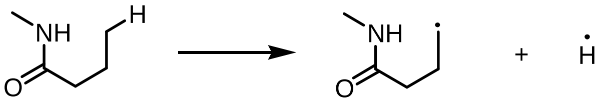	102.0
9	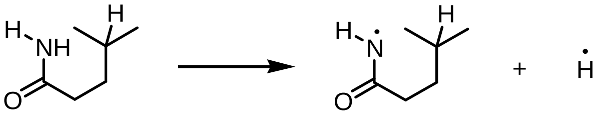	113.9
10	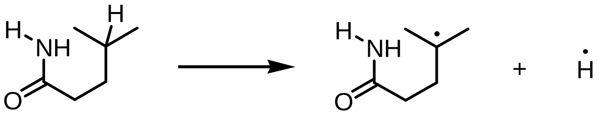	95.3

11	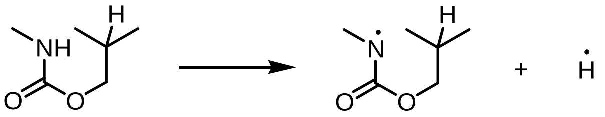	103.2
12	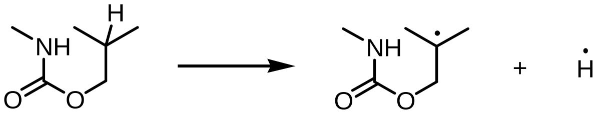	94.6
13	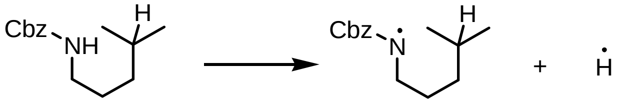	105.2
14	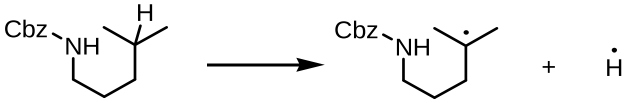	94.0
15	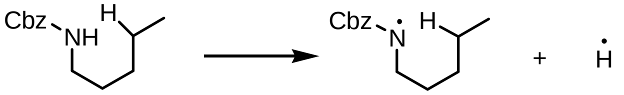	105.6
16	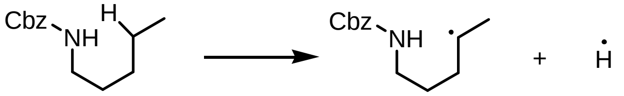	97.2
17	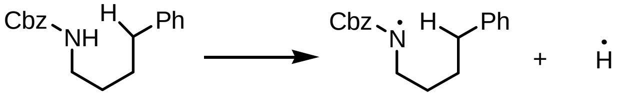	105.3
18	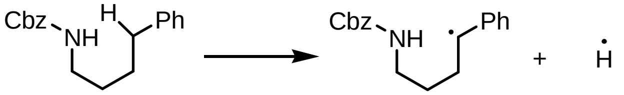	87.4
19	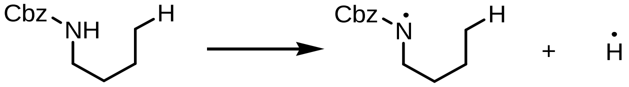	105.5
20	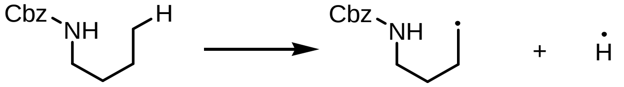	101.1
21	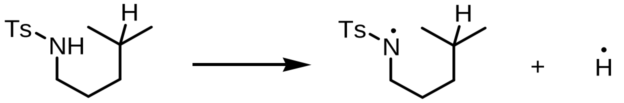	98.8
22	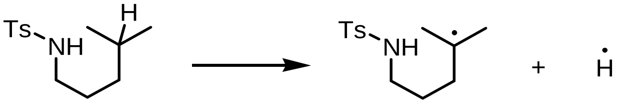	93.8
23	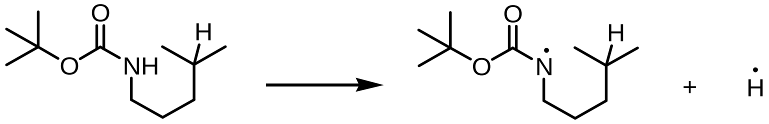	104.8

24	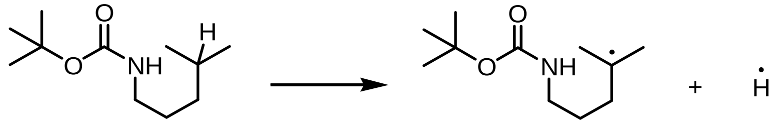	94.2

Computed Energies [values are in Hartree]

No.	Species	Total Electronic Energy	Sum of Electronic and Zero-point Energies	Sum of Electronic and Thermal Enthalpies	Gibbs Free Energy
1	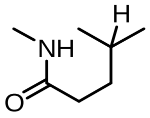	-407.1974608	-406.985718	-406.973125	-407.024023
2	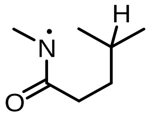	-406.4958348	-406.298801	-406.286220	-406.337930
3	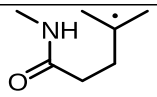	-406.5154416	-406.317876	-406.304958	-406.357129
4	H•	-0.5185156	-0.518516	-0.516155	-0.529170
5	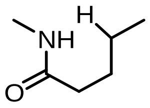	-367.7264905	-367.542030	-367.530791	-367.578561
6	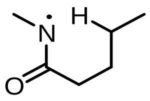	-367.0241303	-366.854235	-366.842965	-366.891755
7	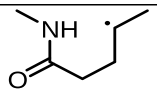	-367.0397695	-366.869952	-366.858487	-366.907192
8	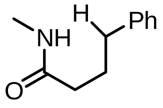	-560.0803374	-559.843993	-559.829619	-559.886875
9	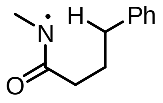	-559.3759517	-559.153916	-559.139553	-559.198157
10	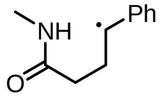	-559.4073349	-559.184222	-559.170025	-559.226900
11	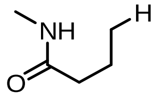	-328.2559971	-328.099453	-328.089536	-328.133912

12	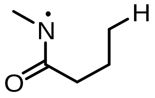	-327.5537407	-327.411705	-327.401802	-327.446869
13	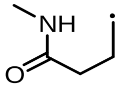	-327.5627113	-327.420691	-327.410759	-327.455068
14	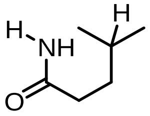	-367.7334181	-367.549594	-367.538325	-367.586046
15	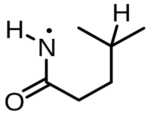	-367.0210659	-366.851569	-366.840593	-366.888599
16	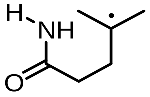	-367.0514748	-366.881577	-366.870265	-366.918253
17	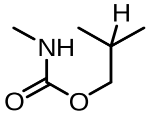	-443.1062991	-442.917680	-442.905398	-442.955423
18	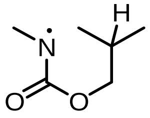	-442.4114122	-442.237027	-442.224822	-442.275836
19	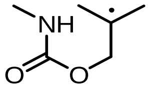	-442.4255897	-442.251051	-442.238553	-442.289633
20	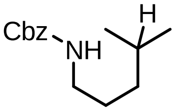	-753.8761219	-753.551268	-753.532089	-753.601289
21	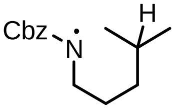	-753.177627	-752.867384	-752.848209	-752.919096
22	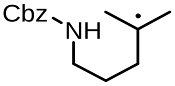	-753.1961947	-752.885735	-752.866080	-752.937433
23	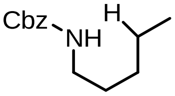	-714.405832	-714.108519	-714.090588	-714.157593
24	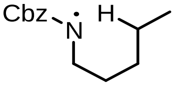	-713.7066993	-713.423990	-713.406071	-713.474190

25	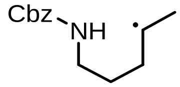	-713.7205701	-713.437726	-713.419541	-713.487957
26	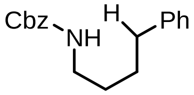	-906.7575669	-906.408116	-906.387086	-906.463328
27	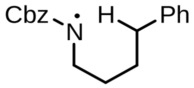	-906.0589106	-905.724085	-905.703075	-905.779841
28	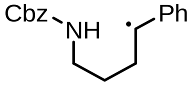	-906.0888906	-905.752501	-905.731727	-905.807210
29	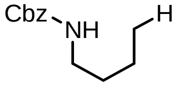	-674.9353658	-674.665874	-674.649329	-674.712803
30	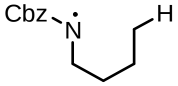	-674.2363972	-673.981571	-673.965019	-674.029079
31	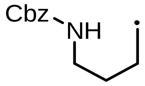	-674.2436603	-673.988779	-673.972051	-674.038422
32	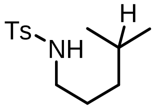	-1114.0929965	-1113.774189	-1113.754053	-1113.824737
33	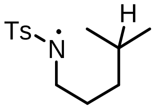	-1113.4052511	-1113.100643	-1113.080418	-1113.152414
34	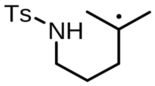	-1113.4135171	-1113.108871	-1113.088436	-1113.160401
35	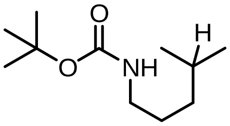	-640.4735676	-640.146748	-640.127958	-640.194538
36	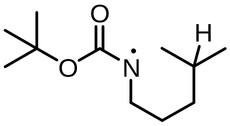	-639.7758637	-639.463453	-639.444748	-639.511048
37	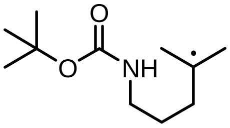	-639.7935228	-639.480847	-639.461716	-639.528673

Optimized Structures and Cartesian Coordinates

No.	Species	Optimized Structure
1	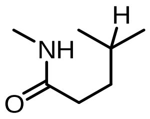	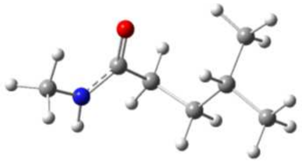

Cartesian Coordinates

C	1.24673800	0.39165300	0.24682900
C	0.20771600	0.16004900	1.32450400
H	0.65877700	-0.32549100	2.19661900
H	-0.14243800	1.14264900	1.64568800
C	-0.97138100	-0.69062000	0.83081800
H	-1.66625200	-0.82388400	1.67001600
H	-0.60569200	-1.69327400	0.57699000
C	-1.74905400	-0.13281300	-0.36624900
H	-1.05744500	-0.05605300	-1.21310900
N	2.27915600	-0.49711400	0.24374400
O	1.14464300	1.28581600	-0.57660900
C	3.33373800	-0.45627900	-0.74190400
H	3.12404600	0.38788300	-1.39784600
H	4.31095800	-0.30920000	-0.27339600
H	3.36053900	-1.37279000	-1.33849100
C	-2.86737200	-1.09981000	-0.74602100
C	-2.29851700	1.26493600	-0.09658800
H	-1.49091700	1.98887400	0.02588400
H	-2.91776500	1.60362700	-0.93179000
H	-2.92269700	1.27665200	0.80462100
H	-3.59606900	-1.19443600	0.06676600
H	-3.40453600	-0.75207100	-1.63253900
H	-2.47875000	-2.09968100	-0.96193400
H	2.30580300	-1.20223400	0.96084700

No.	Species	Optimized Structure
2	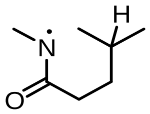	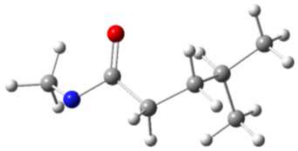

Cartesian Coordinates

C	1.33997200	-0.38340400	0.24282600
C	0.27541700	0.02947900	1.23009900
H	0.61154800	-0.33573900	2.20780200
H	0.28960500	1.12142400	1.32034800
C	-1.10824800	-0.51222600	0.89829000
H	-1.76436000	-0.35802900	1.76371900
H	-1.02308500	-1.59315200	0.75301500
C	-1.77196800	0.09938100	-0.34056500
H	-1.07823400	-0.01861600	-1.18160900
N	2.55607300	0.25761800	0.42143000
O	1.20765700	-1.27833500	-0.57269600
C	3.30587400	0.55837800	-0.75621400
H	2.99278500	-0.05639300	-1.60736000
H	3.13757300	1.61514100	-1.01561300
H	4.37456700	0.44718900	-0.56144100
C	-3.04821300	-0.66606300	-0.67867400
C	-2.07266700	1.58598200	-0.16299200

H	-1.17130700	2.17623800	0.02309500
H	-2.54971300	1.99679900	-1.05705000
H	-2.75520800	1.74615100	0.67917900
H	-3.77076700	-0.59988600	0.14237500
H	-3.52768700	-0.26241700	-1.57471100
H	-2.84048000	-1.72451600	-0.85681600

3	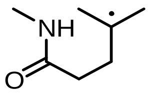	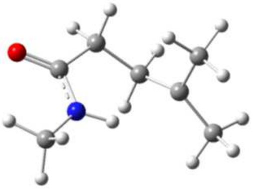

Cartesian Coordinates

C	1.48930300	-0.54573800	-0.02994000
C	0.29260100	-1.48496500	-0.08594800
H	0.64467000	-2.36160800	-0.63318900
H	0.11685100	-1.83187700	0.93824000
C	-1.00512900	-0.97114000	-0.71427100
H	-1.67872400	-1.84048500	-0.82379200
H	-0.80467100	-0.64053500	-1.74052600
C	-1.74777600	0.10879400	0.01381200
N	1.20968100	0.78225800	-0.08307600
O	2.62119000	-0.98614900	0.07332400
C	2.24173500	1.78837200	0.00074300
H	3.19681300	1.26720300	0.05711000
H	2.23797800	2.43499500	-0.88128000
H	2.12292800	2.40950700	0.89362800
C	-2.77119700	0.86949100	-0.75829500
C	-1.90765600	0.02410700	1.49388100
H	-0.96247100	-0.17051600	2.00793300
H	-2.32896800	0.94643400	1.90198300
H	-2.59323500	-0.79204000	1.77792100
H	-3.70415400	0.29065300	-0.86743300
H	-3.04331800	1.80536700	-0.26151600
H	-2.42930500	1.10194300	-1.77119600
H	0.23703100	1.06081500	-0.12282700

4	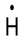	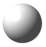

Cartesian Coordinates

H	0.00000000	0.00000000	0.00000000

5	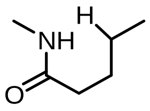	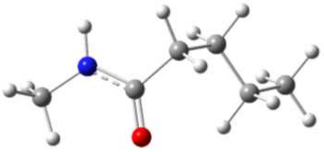

Cartesian Coordinates

C	-1.00756700	0.09456700	0.42833100
C	0.04883800	1.17617600	0.53524000
H	-0.35501500	2.14189300	0.21377100
H	0.31025100	1.26597700	1.59323700
C	1.30613600	0.84508200	-0.28031300
H	1.93882800	1.74000500	-0.31343300
H	1.01803300	0.63933500	-1.31917700
C	2.12045200	-0.32356100	0.26549400
H	1.48104400	-1.20618500	0.34670200
N	-2.05203400	0.39235500	-0.39460900
O	-0.91357000	-0.97253100	1.01104600

C	-3.12530500	-0.53949500	-0.64926300
H	-2.92436900	-1.42778600	-0.05136300
H	-4.09236900	-0.12228600	-0.35483500
H	-3.16781200	-0.82362100	-1.70485600
C	3.34311000	-0.62856300	-0.58953700
H	4.00939100	0.23733500	-0.65817700
H	3.92086500	-1.45867300	-0.17574600
H	3.05471100	-0.90341100	-1.60890900
H	-2.06943300	1.29292200	-0.84218500
H	2.43468700	-0.08697100	1.28915500

6

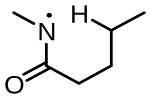

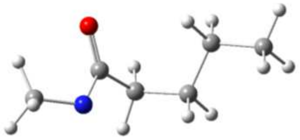

Cartesian Coordinates

C	-1.02929000	0.02792200	0.41984100
C	-0.00510200	1.10063000	0.67647400
H	-0.44112700	2.07380100	0.42964700
H	0.23146600	1.08214300	1.74361200
C	1.26635800	0.88697200	-0.15906300
H	1.90659800	1.76818800	-0.03852600
H	0.98922600	0.84684400	-1.21816800
C	2.04987600	-0.36422400	0.22094700
H	1.40404200	-1.24392000	0.13761600
N	-1.78008800	0.21391200	-0.72777300
O	-1.12557500	-0.99441700	1.07841200
C	-3.12991500	-0.25144100	-0.70941600
H	-3.32728700	-0.91864700	0.13614500
H	-3.79251300	0.62378800	-0.63168700
H	-3.37128600	-0.73824600	-1.65771100
C	3.29435700	-0.55415500	-0.63658500
H	3.97601200	0.29711100	-0.54395500
H	3.84523200	-1.45216400	-0.34652100
H	3.03340300	-0.65305300	-1.69458700
H	2.33374400	-0.30212000	1.27806100

7

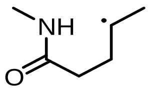

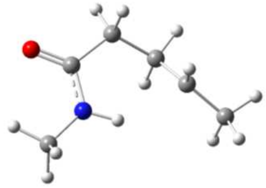

Cartesian Coordinates

C	1.25508600	-0.54663100	0.03165600
C	0.04515800	-1.46559000	0.13054900
H	0.33613400	-2.37612700	-0.39617200
H	-0.05395700	-1.74761600	1.18582300
C	-1.29428300	-0.94814500	-0.39555100
H	-1.97725600	-1.81185400	-0.47664500
H	-1.17871700	-0.58828200	-1.42688200
C	-1.94514700	0.10168900	0.43656000
N	0.99195400	0.78526600	-0.01817000
O	2.38433000	-1.00439600	0.01137600
C	2.04374100	1.77348500	-0.06046600
H	2.98776700	1.23657700	-0.14649200
H	1.92767600	2.43673300	-0.92196600
H	2.06333000	2.37904200	0.85098000

C	-3.11282500	0.86477100	-0.07236700
H	-4.03021900	0.25390000	-0.06411100
H	-3.32033800	1.74985700	0.53383300
H	-2.96672500	1.18415600	-1.10977800
H	0.02942900	1.08266700	0.07342400
H	-1.78583700	0.06178000	1.51187600

8

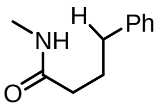

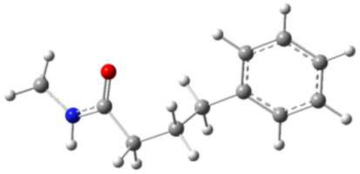

Cartesian Coordinates

C	-2.72039500	0.02609600	0.29355500
C	-1.89197400	-1.17319700	0.73076900
H	-2.28806000	-1.49647800	1.70091300
H	-2.04345900	-2.01056200	0.03910600
C	-0.41305200	-0.83979500	0.86648300
H	0.10184000	-1.66050300	1.37578700
H	-0.31652100	0.04541700	1.50003800
C	0.26769200	-0.56704600	-0.47837100
H	-0.27120600	0.23861200	-0.98630800
N	-3.89808700	-0.28627300	-0.31127300
O	-2.36433700	1.17409300	0.49413100
C	-4.84753300	0.71938300	-0.72811100
H	-4.39869300	1.68966800	-0.51949500
H	-5.05779200	0.64637600	-1.79862700
H	-5.78808000	0.63755900	-0.17528000
H	-4.12701100	-1.25784400	-0.43897100
H	0.19277300	-1.45724500	-1.11354800
C	1.71308100	-0.17984800	-0.32259900
C	2.73458200	-1.11623700	-0.47884800
C	2.05944000	1.12914400	0.01840900
C	4.06623500	-0.75882100	-0.29974500
H	2.48312200	-2.13748400	-0.74996600
C	3.38815700	1.49068200	0.19874800
H	1.27417000	1.86927100	0.13946000
C	4.39725700	0.54679700	0.04064300
H	4.84644300	-1.50103500	-0.43025000
H	3.63763100	2.51361800	0.45929700
H	5.43520700	0.82883900	0.17809400

9

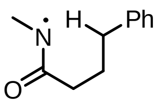

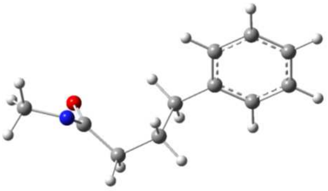

Cartesian Coordinates

C	2.79939400	-0.31686600	0.16194300
C	1.96693800	-1.32405000	-0.58545200
H	2.41971500	-1.50412400	-1.56539200
H	1.98324400	-2.25587700	-0.01384300
C	0.52370100	-0.83651600	-0.78020900
H	0.00700200	-1.53400000	-1.44721500
H	0.53884100	0.13037000	-1.29260500
C	-0.26205400	-0.71536600	0.52924200
H	0.26774600	-0.04828100	1.21535400
N	3.29541100	0.71116400	-0.61823700

O	2.93169900	-0.30897200	1.37496800
C	4.56294100	1.26023300	-0.25708600
H	4.88676800	0.94186800	0.73926200
H	5.30431800	0.92232000	-0.99669800
H	4.53223000	2.35052800	-0.32884500
H	-0.29657400	-1.69462500	1.01853900
C	-1.66103300	-0.21009400	0.30124100
C	-2.72020800	-1.09468900	0.09647400
C	-1.92371700	1.15973400	0.25100600
C	-4.00484900	-0.62674100	-0.15316300
H	-2.53581800	-2.16405000	0.14041500
C	-3.20572800	1.63274000	0.00088400
H	-1.11238800	1.86240100	0.41627000
C	-4.25161800	0.73991700	-0.20299600
H	-4.81567200	-1.33112100	-0.30436400
H	-3.38988100	2.70117800	-0.02966100
H	-5.25360400	1.10722200	-0.39460600

10	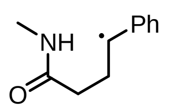	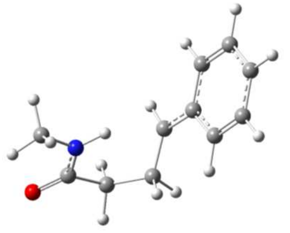

Cartesian Coordinates

C	2.91046900	-0.09389200	-0.00065200
C	2.27085500	-1.46935800	0.13332100
H	2.91006700	-2.13793700	-0.44663700
H	2.39419100	-1.77281800	1.17966000
C	0.80864700	-1.63245600	-0.29051700
H	0.60019300	-2.71241600	-0.34829100
H	0.68031300	-1.25989700	-1.31237600
C	-0.17124500	-1.00252000	0.63753900
N	2.06226000	0.96465300	-0.07817000
O	4.12318700	0.02115500	-0.02785100
C	2.54286700	2.32457000	-0.15823700
H	3.62674900	2.27912800	-0.25655100
H	2.12235800	2.83868000	-1.02638600
H	2.29270300	2.89208100	0.74331400
H	1.07150400	0.79973900	0.02469500
H	0.07883700	-1.02017100	1.69582600
C	-1.43366200	-0.46698100	0.28651200
C	-2.27410200	0.06662500	1.29662000
C	-1.91879600	-0.43079700	-1.04437200
C	-3.50985800	0.60159300	0.99483600
H	-1.92756500	0.04728100	2.32492200
C	-3.15864100	0.10736100	-1.33541700
H	-1.31534300	-0.83695900	-1.84752600
C	-3.96364500	0.62775900	-0.32410800
H	-4.13083900	1.00189000	1.78869300
H	-3.50722700	0.12089700	-2.36235800
H	-4.93459600	1.04726000	-0.56014200

11

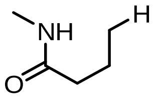

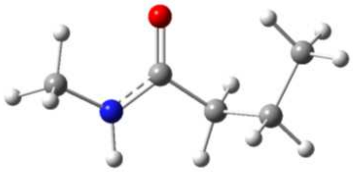

Cartesian Coordinates

C	-0.45305700	0.08644600	-0.39827000
C	0.74484300	-0.78379100	-0.72109400
H	0.43898500	-1.82607100	-0.86051400
H	1.15041700	-0.42961300	-1.67267300
C	1.82923700	-0.70445300	0.36391400
H	2.58235900	-1.46925600	0.14632700
H	1.38779200	-0.97872000	1.32929800
C	2.50071700	0.65862500	0.46859900
H	1.77177800	1.44580100	0.66594600
N	-1.53385400	-0.58285000	0.09271100
O	-0.43715400	1.29783700	-0.53701500
C	-2.74284800	0.09453500	0.49829800
H	-2.59721700	1.15479000	0.29429000
H	-3.60781600	-0.26247500	-0.06764500
H	-2.93820900	-0.03815400	1.56651600
H	-1.48265900	-1.58401700	0.17479000
H	3.00914200	0.91357900	-0.46579500
H	3.24628300	0.66321400	1.26793200

12

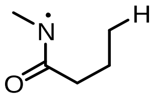

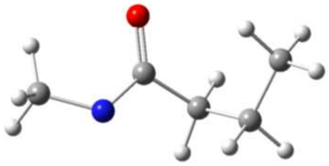

Cartesian Coordinates

C	0.47600000	0.20813100	0.33387100
C	-0.69595500	-0.52528100	0.92881200
H	-0.34843300	-1.48069400	1.33480900
H	-1.08480800	0.08468700	1.74828100
C	-1.79386300	-0.78574600	-0.11444300
H	-2.55175000	-1.42738400	0.34632200
H	-1.36068800	-1.35837900	-0.94027000
C	-2.44522400	0.48649900	-0.63928400
H	-1.71530700	1.14725900	-1.11223200
N	1.34977700	-0.59051700	-0.38282000
O	0.60201800	1.42136800	0.35392500
C	2.72921300	-0.22191000	-0.37710400
H	2.89077100	0.78457600	0.02259600
H	3.27295200	-0.94533600	0.24884300
H	3.14463100	-0.31403800	-1.38408800
H	-2.91720200	1.05001000	0.17066700
H	-3.21576700	0.25181500	-1.37770000

13

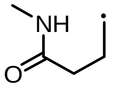

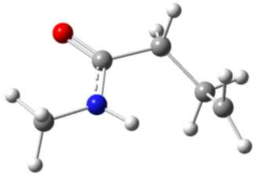

Cartesian Coordinates

C	-0.57930700	0.65618100	0.04821400
C	0.88167400	1.08068300	0.11438100
H	0.92082300	2.04444500	-0.39664300
H	1.10213500	1.29029100	1.16797000
C	1.93563500	0.13019200	-0.45761500
H	2.88002200	0.69476000	-0.53877500
H	1.68146300	-0.13576900	-1.49077500
C	2.18171700	-1.09574400	0.34373800
N	-0.81416400	-0.67730300	-0.06313500
O	-1.46866800	1.48723500	0.10403000
C	-2.15221600	-1.22027200	-0.07902500
H	-2.84204400	-0.37727300	-0.09359800
H	-2.31716700	-1.83579800	-0.96743000
H	-2.35091400	-1.82455400	0.81145200
H	-0.02341300	-1.30606600	-0.02973000
H	2.11413900	-1.06091300	1.42608300
H	2.67842900	-1.95212300	-0.09700700

14	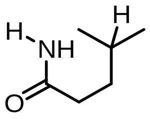	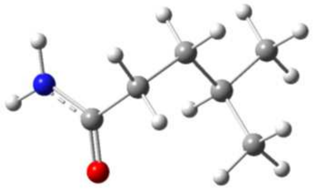

Cartesian Coordinates

C	1.69888900	0.20286100	-0.03625600
C	0.80708600	-0.19006300	1.12467100
H	1.33444200	-0.87472200	1.79720600
H	0.59813700	0.72258100	1.68425900
C	-0.50096100	-0.84721400	0.66009700
H	-1.09601000	-1.08425200	1.55122800
H	-0.27122900	-1.80793500	0.18333200
C	-1.35907000	-0.01670500	-0.29967300
H	-0.77395000	0.15897300	-1.21057500
N	2.51750400	-0.78417400	-0.49628600
O	1.66500600	1.30482700	-0.55001800
H	2.56424200	-1.68959100	-0.06443700
H	3.10081000	-0.58926900	-1.29328000
C	-2.60956600	-0.80428500	-0.68253600
C	-1.72365400	1.34634100	0.28154300
H	-0.83680600	1.96769500	0.41893500
H	-2.39944500	1.88295600	-0.39007800
H	-2.23216900	1.23904600	1.24667500
H	-3.23914200	-0.98762000	0.19515700
H	-3.21177600	-0.25740200	-1.41295100
H	-2.35603000	-1.77547200	-1.11840000

15	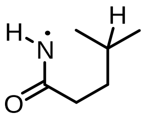	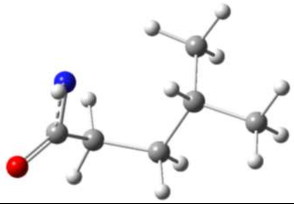

Cartesian Coordinates

C	1.81513400	0.05820800	-0.04349600
C	0.86605900	-0.54306000	0.95208200
H	1.37860300	-1.39271900	1.40693400
H	0.67110300	0.19686800	1.73364100
C	-0.45461300	-1.00423000	0.31766500

C	-1.36398000	0.09966100	-0.23225800
H	-0.78905600	0.68360100	-0.96637300
N	1.50599100	1.29584100	-0.55611800
O	2.89898900	-0.42747400	-0.33901100
H	1.85113400	1.36348600	-1.51752400
C	-2.55512400	-0.52226900	-0.95553500
C	-1.83026000	1.06201800	0.85620400
H	-3.16599000	-1.10968600	-0.26114300
H	-3.19812900	0.24601800	-1.39268700
H	-2.23265800	-1.18924500	-1.76012800
H	-0.99466100	1.58967100	1.32132400
H	-2.50184100	1.81912200	0.44279400
H	-2.37705200	0.52600000	1.63992600
H	-0.22754500	-1.71623300	-0.48457300
H	-1.01105600	-1.56995000	1.07475100

16	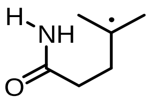	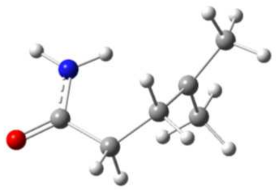

Cartesian Coordinates

C	1.81056400	0.11812100	-0.02540700
C	0.86419200	-1.07137100	-0.10932100
H	1.41042300	-1.83244600	-0.66996700
H	0.77086300	-1.46886700	0.90723600
C	-0.51495200	-0.85289000	-0.73479100
H	-0.97203400	-1.84974700	-0.87161900
H	-0.39349900	-0.45787600	-1.75061100
C	-1.48635600	0.00912400	0.01520900
N	1.23181100	1.34624100	-0.06585900
O	3.01124700	-0.03862400	0.08189200
H	0.22724000	1.45363100	-0.07972100
H	1.82652000	2.15171800	0.04081200
C	-2.66340800	0.52239100	-0.74165600
C	-1.61547900	-0.14039800	1.49326200
H	-0.64870800	-0.12122400	2.00398000
H	-2.23746200	0.65031200	1.92068700
H	-2.09189900	-1.09894400	1.75996100
H	-3.43529000	-0.25701300	-0.86051800
H	-3.14528100	1.35941400	-0.22776100
H	-2.39089600	0.84648800	-1.75038300

17	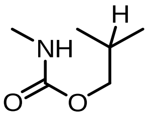	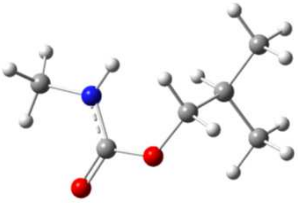

Cartesian Coordinates

C	1.38115800	-0.57914200	-0.15672800
C	-0.88438100	-0.13616900	-0.96574100
H	-1.51733200	-0.70909000	-1.64859200
H	-0.54124100	0.74416300	-1.52667500
C	-1.69110600	0.26277900	0.26598100
H	-1.02246400	0.79369700	0.95480100
N	1.53020900	0.76883500	-0.01335600
O	2.21924400	-1.38851000	0.14894700

C	2.78487200	1.32926900	0.43719300
H	3.36543200	0.52099000	0.87803400
H	3.36039100	1.76096900	-0.38808600
H	2.61047000	2.09966200	1.19170600
C	-2.81241800	1.21196100	-0.14673400
C	-2.23109700	-0.97071900	0.98190500
H	-1.42718800	-1.66180800	1.24065100
H	-2.75180200	-0.69058600	1.90101600
H	-2.94231600	-1.50705400	0.34448000
H	-3.49525100	0.72649000	-0.85171600
H	-3.40039900	1.51821400	0.72163300
H	-2.42860300	2.11861900	-0.62437500
H	0.83254000	1.39356400	-0.37665100
O	0.20127400	-1.01118400	-0.67619500

18	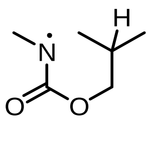	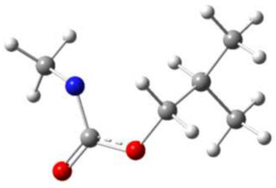

Cartesian Coordinates

C	1.45162400	-0.55761600	-0.17760200
C	-0.81987400	-0.34720700	-0.94332000
H	-1.43978200	-1.05032200	-1.50469700
H	-0.46033000	0.43330600	-1.61637800
C	-1.61077400	0.25264200	0.21030200
H	-0.93151200	0.91687800	0.76157500
N	1.51847800	0.81149600	-0.38107500
O	2.40527500	-1.21630200	0.16747200
C	2.34029400	1.53815400	0.53364900
H	2.93044400	0.89236000	1.18948400
H	2.98906800	2.22137800	-0.02246000
H	1.68400300	2.17685100	1.14360200
C	-2.75054900	1.09938700	-0.34853400
C	-2.12072600	-0.82482900	1.16081700
H	-1.30485600	-1.44931300	1.52938300
H	-2.62747500	-0.37967300	2.02089000
H	-2.83791300	-1.47784600	0.65237200
H	-3.44750700	0.48238000	-0.92532200
H	-3.31812000	1.56842400	0.45857300
H	-2.38194400	1.89295200	-1.00449700
O	0.29180000	-1.14257700	-0.49583200

19	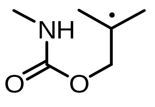	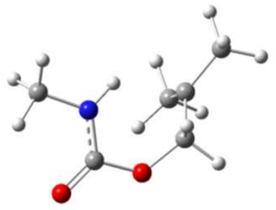

Cartesian Coordinates

C	1.38221000	-0.56593700	-0.08726600
C	-0.89457200	-0.68627900	-0.91027800
H	-1.47439700	-1.53624500	-1.29939400
H	-0.61171100	-0.07024600	-1.77306700
C	-1.71246200	0.10523000	0.05823700
N	1.22462000	0.78897300	-0.14338700
O	2.42022700	-1.12771200	0.14922000

C	2.32920400	1.64812700	0.22637900
H	3.25510700	1.16526500	-0.08111500
H	2.23558900	2.60633100	-0.28731300
H	2.37992000	1.82316200	1.30674300
C	-2.73795900	1.02409400	-0.50867700
C	-1.89332200	-0.41598700	1.44000900
H	-1.00417600	-0.93856600	1.79649000
H	-2.13872900	0.38676900	2.14160500
H	-2.72706100	-1.13619200	1.48356500
H	-3.66353900	0.48538100	-0.77044800
H	-3.02542500	1.79516100	0.21228000
H	-2.39200200	1.51793600	-1.42175400
H	0.28584300	1.14979500	-0.03252400
O	0.26347900	-1.30064500	-0.34694300

20	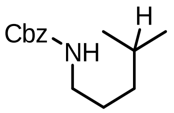	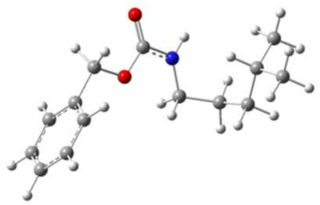

Cartesian Coordinates

C	1.40475200	-0.37486100	1.54526500
C	2.78159700	0.18695600	1.88265400
H	2.70153300	0.63397500	2.87920800
H	3.49640000	-0.63797700	1.99455100
C	3.32791600	1.25108300	0.92890300
H	4.18404400	1.73848200	1.41461400
H	2.56574300	2.03238100	0.81409400
C	3.77782300	0.79679000	-0.46543200
H	2.92985000	0.31142000	-0.96017100
N	1.43717800	-1.27408100	0.40373400
C	4.17986400	2.01192500	-1.29778100
C	4.92890000	-0.20412100	-0.40112900
H	4.66255000	-1.11640200	0.13966900
H	5.24356200	-0.50299900	-1.40461300
H	5.79752200	0.23445600	0.10289700
H	5.02635000	2.53526400	-0.83930800
H	4.47859800	1.71986300	-2.30828800
H	3.35571400	2.72566400	-1.38526400
H	2.21081600	-1.91488000	0.32259800
H	1.00110300	-0.89227800	2.42587400
H	0.71359800	0.43682200	1.31147100
C	0.37624300	-1.61762300	-0.37315500
O	-0.69816300	-0.83929200	-0.10864500
O	0.38920100	-2.49320300	-1.20901300
C	-1.84879500	-1.11429500	-0.92851700
H	-1.59754200	-0.91799000	-1.97322200
H	-2.09958000	-2.17350400	-0.83899100
C	-2.96618600	-0.23642700	-0.45749500
C	-3.73980600	-0.60658500	0.64206700
C	-3.23709000	0.97169000	-1.09590300
C	-4.76441100	0.21398600	1.09399000
H	-3.53626600	-1.54699800	1.14416500
C	-4.26325700	1.79502200	-0.64849400
H	-2.63930900	1.26725400	-1.95223300
C	-5.02822700	1.41726300	0.44827500
H	-5.36146400	-0.08657600	1.94782000
H	-4.46664700	2.73086300	-1.15699000

H	-5.83105600	2.05687100	0.79775200

21

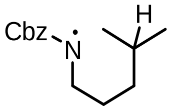

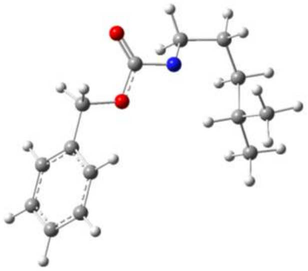

Cartesian Coordinates

C	-2.60604800	-1.58893000	-0.51558400
C	-3.94403500	-0.91295600	-0.26770800
H	-4.71160000	-1.49101700	-0.79370800
H	-4.17907900	-0.98770800	0.79840000
C	-4.02330400	0.53798700	-0.74383900
H	-5.05968400	0.88317200	-0.63340000
H	-3.81484600	0.56084600	-1.82211200
C	-3.10374000	1.54700900	-0.04671100
H	-2.06626600	1.20849200	-0.16178400
N	-1.58149100	-1.15602500	0.38903900
C	-3.23181700	2.91244700	-0.71767100
C	-3.38864600	1.64789500	1.44854500
H	-3.19076200	0.70388600	1.95989000
H	-2.75616100	2.40964800	1.91280500
H	-4.43298400	1.92769200	1.62958000
H	-4.25231900	3.29990700	-0.62111800
H	-2.55605800	3.64162200	-0.26254000
H	-2.99658000	2.85858700	-1.78478000
H	-2.68797200	-2.68155200	-0.39817400
H	-2.28014500	-1.44249400	-1.55992400
C	-0.36614600	-1.80645900	0.26132400
O	0.63716900	-0.92581300	0.20897800
O	-0.23438400	-3.00931500	0.27454000
C	1.95847700	-1.51093800	0.16058600
H	2.10455800	-2.09564100	1.07224600
H	2.01517700	-2.19514500	-0.68828800
C	2.95485000	-0.40144500	0.04432400
C	3.62440300	-0.17273800	-1.15478100
C	3.22288000	0.42229000	1.13787500
C	4.55251100	0.85671400	-1.26175300
H	3.41853700	-0.80741200	-2.01079200
C	4.14441100	1.45412800	1.03284100
H	2.70112300	0.25281300	2.07399900
C	4.81303200	1.67187500	-0.16785600
H	5.07044000	1.02240700	-2.19972700
H	4.34550200	2.08823100	1.88903100
H	5.53632500	2.47559200	-0.24858100

22

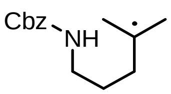

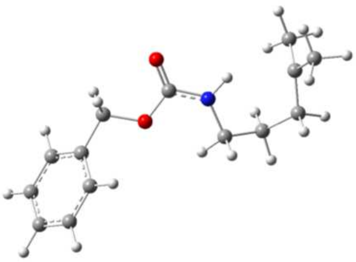

Cartesian Coordinates

C	-1.39816400	-1.15459900	0.06970900
C	-2.58605000	-1.66177100	0.87839100
H	-2.41129600	-2.72048100	1.09585300
H	-2.60814200	-1.14729500	1.84429800
C	-3.94190600	-1.53584000	0.16867300
H	-4.67700000	-2.12208000	0.74875200
H	-3.87365500	-2.03550900	-0.80518600
C	-4.48137600	-0.14991000	-0.02424300
N	-1.45746400	0.27905800	-0.13615400
C	-5.28018500	0.12840000	-1.25152100
C	-4.73231600	0.70049100	1.17507800
H	-3.93715900	0.62310900	1.92065400
H	-4.83770500	1.75522600	0.90558800
H	-5.66934000	0.40797500	1.67892700
H	-6.30241800	-0.27918600	-1.16788600
H	-5.38792900	1.20202600	-1.43127600
H	-4.83461200	-0.32681800	-2.14092900
H	-0.46784500	-1.37753800	0.59201100
H	-1.36063000	-1.68434700	-0.89347300
H	-2.35535600	0.68181200	-0.36941500
C	-0.40918200	1.12924500	-0.24766100
O	0.76895400	0.49636600	-0.01798300
O	-0.49961600	2.30913400	-0.50526100
C	1.92340000	1.34615300	-0.06721500
H	1.87580000	1.94785000	-0.97875800
H	1.90603600	2.03471400	0.78128800
C	3.14725800	0.48211500	-0.03553100
C	4.11336800	0.65688300	0.95101800
C	3.34642200	-0.49834900	-1.00859300
C	5.26397400	-0.12461700	0.96422900
H	3.96329800	1.41088900	1.71727000
C	4.48889600	-1.28495500	-0.99340200
H	2.59439300	-0.64613000	-1.77613600
C	5.45309700	-1.09831600	-0.00710900
H	6.00867500	0.02441000	1.73821000
H	4.63163400	-2.04423700	-1.75447800
H	6.34739300	-1.71137900	0.00276700

23	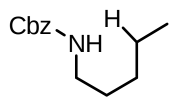	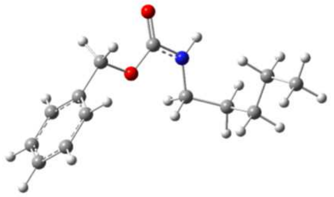

Cartesian Coordinates

C	-1.75944800	0.46683200	1.51444800
C	-3.12837200	-0.13911900	1.80442300
H	-3.07341600	-0.56510000	2.81187900
H	-3.87788500	0.66200200	1.86520900
C	-3.60752100	-1.22588200	0.84047300
H	-4.43024800	-1.76918100	1.32138400
H	-2.80367900	-1.96121800	0.70509200
C	-4.08868800	-0.74403000	-0.52630300
H	-3.27264000	-0.24220000	-1.05262000
N	-1.76549300	1.32435500	0.34182300
C	-4.63290700	-1.87823400	-1.38503300
H	-5.47584400	-2.37761300	-0.89710300
H	-4.97885100	-1.51471800	-2.35579400
H	-3.86492800	-2.63541300	-1.57042500
H	-2.54515800	1.94950800	0.21115600

H	-1.42201700	1.02925400	2.39553900
H	-1.02649300	-0.32331000	1.34436900
C	-0.69100200	1.64092600	-0.42707200
O	0.38206100	0.87973000	-0.11108500
O	-0.69150400	2.48108600	-1.29874400
C	1.54629600	1.12914200	-0.91956000
H	1.30186300	0.93898700	-1.96672800
H	1.81936000	2.18290000	-0.82707300
C	2.63996100	0.22771600	-0.43730000
C	3.38554600	0.56280100	0.69261400
C	2.91706700	-0.96725800	-1.09721200
C	4.38815900	-0.27961000	1.15325500
H	3.17703100	1.49266600	1.21199300
C	3.92200000	-1.81211200	-0.64137600
H	2.34108200	-1.23562000	-1.97711600
C	4.65870000	-1.46952800	0.48569200
H	4.96323700	-0.00654900	2.03108100
H	4.13043200	-2.73736700	-1.16692200
H	5.44466700	-2.12603600	0.84208800
H	-4.87126200	0.01212500	-0.38243200

24	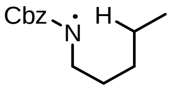	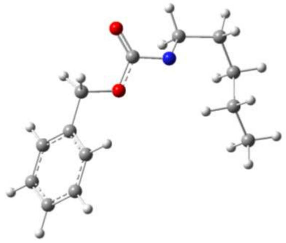

Cartesian Coordinates

C	2.89692800	-1.32379900	0.42408900
C	4.20013600	-0.60992700	0.10393900
H	5.00222500	-1.13262300	0.63628200
H	4.40571800	-0.72524700	-0.96588300
C	4.23348800	0.86874800	0.49030100
H	5.26708700	1.22601400	0.40762700
H	3.96966600	0.96657400	1.55266400
C	3.33291600	1.77932200	-0.33914600
H	2.29427600	1.44055800	-0.27379400
N	1.82870300	-0.98419400	-0.46908900
C	3.43017600	3.23844600	0.08702400
H	4.45469400	3.61233200	-0.00659500
H	2.78669100	3.87704100	-0.52316100
H	3.12795800	3.36662400	1.13110400
H	3.02145800	-2.41708800	0.36389600
H	2.59496400	-1.13306700	1.46860000
C	0.64954900	-1.68155500	-0.27175200
O	-0.39163800	-0.84519000	-0.22743600
O	0.57284600	-2.88836700	-0.22524700
C	-1.68204400	-1.48522700	-0.11092400
H	-1.82503300	-2.12272100	-0.98729300
H	-1.68581600	-2.12694300	0.77234200
C	-2.72611100	-0.41740800	-0.02236500
C	-3.43924600	-0.21939700	1.15663700
C	-2.99928600	0.39391200	-1.12415300
C	-4.41616200	0.76689700	1.23577100
H	-3.22907000	-0.84346500	2.01936600
C	-3.96861900	1.38327300	-1.04618200
H	-2.44226900	0.24965100	-2.04393100
C	-4.68126700	1.57009700	0.13445800

H	-4.96732000	0.90885700	2.15858100
H	-4.17255300	2.00872900	-1.90805700
H	-5.44168400	2.34073600	0.19368300
H	3.60568800	1.68156200	-1.39652900

25

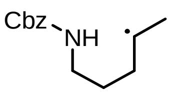

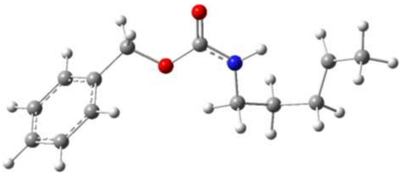

Cartesian Coordinates

C	1.70141800	-1.07495100	-0.08277700
C	2.91418900	-1.68330700	-0.77725500
H	2.74391400	-2.76210800	-0.85122600
H	2.96970400	-1.30206000	-1.80306100
C	4.24887900	-1.44582000	-0.06227900
H	4.99372100	-2.13807300	-0.49417200
H	4.15974500	-1.75003500	0.98994400
C	4.78203000	-0.05723400	-0.14131700
N	1.74269300	0.37398800	-0.08643700
C	5.80029100	0.42855700	0.82385800
H	6.78592500	-0.03022800	0.64101200
H	5.93917200	1.51021100	0.76196700
H	5.53120000	0.17578800	1.85519300
H	0.78747900	-1.38217800	-0.59101200
H	1.63974500	-1.46077000	0.94528300
H	2.63013900	0.82208900	0.09655900
C	0.68028500	1.21440200	-0.09929300
O	-0.48581400	0.53441300	-0.23521400
O	0.75285900	2.42026900	-0.01239400
C	-1.65967100	1.35910700	-0.31571000
H	-1.57682400	2.15653400	0.42552000
H	-1.70280600	1.82457600	-1.30388000
C	-2.85573600	0.49058700	-0.07414000
C	-3.69969800	0.12988200	-1.12099700
C	-3.13667700	0.02346900	1.21031700
C	-4.80812300	-0.67806900	-0.89230600
H	-3.48700800	0.48651800	-2.12380700
C	-4.23903300	-0.78653100	1.44211200
H	-2.48374100	0.30017600	2.03173800
C	-5.07876100	-1.13836900	0.38964900
H	-5.45923600	-0.94856400	-1.71614700
H	-4.44852700	-1.14077600	2.44538500
H	-5.94301800	-1.76771900	0.57067500
H	4.68885000	0.46091700	-1.09322500

26

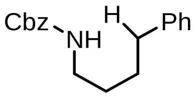

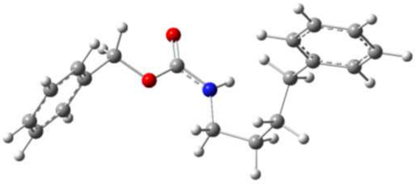

Cartesian Coordinates

C	0.08824400	-2.02376900	-1.10303000
C	-1.30585100	-2.18225100	-1.69935500
H	-1.17121300	-2.30725900	-2.77894100
H	-1.75055200	-3.12361100	-1.34845200
C	-2.28014800	-1.02635700	-1.46643300

H	-3.10912600	-1.12275600	-2.17624100
H	-1.78495000	-0.07942500	-1.71239900
C	-2.87167800	-0.93299800	-0.05379800
H	-2.07103100	-0.78148000	0.67349600
N	0.09131000	-2.14629000	0.34520500
H	-0.47167900	-2.87110100	0.76232700
H	0.75736800	-2.77087200	-1.55066800
H	0.49568100	-1.04352100	-1.35503200
C	1.04645200	-1.66555200	1.18555500
O	1.89335100	-0.83139000	0.54007500
O	1.12192100	-1.93016400	2.36406800
C	2.90519500	-0.24190000	1.37662900
H	2.42879500	0.42280000	2.10085900
H	3.40551100	-1.03716100	1.93359000
C	3.85526100	0.50392100	0.49195700
C	4.79174800	-0.18738700	-0.27697000
C	3.81067700	1.89327900	0.41118600
C	5.66461900	0.49770000	-1.11034900
H	4.83370700	-1.27024700	-0.21904000
C	4.68644000	2.58378200	-0.41889200
H	3.08397300	2.43820000	1.00528000
C	5.61371000	1.88626300	-1.18235600
H	6.39000600	-0.05010700	-1.70159800
H	4.64365200	3.66603500	-0.46947200
H	6.29843200	2.42216900	-1.83018100
H	-3.34744100	-1.88919400	0.19522400
C	-3.88393700	0.17434100	0.06496300
C	-5.22254600	-0.03953500	-0.26710500
C	-3.50090300	1.45190900	0.47462500
C	-6.15149300	0.99104400	-0.19457900
H	-5.54147500	-1.02975600	-0.57940200
C	-4.42566400	2.48659300	0.54818100
H	-2.46537600	1.63418900	0.74584800
C	-5.75516000	2.25949500	0.21297700
H	-7.18817100	0.80226900	-0.45170800
H	-4.10831400	3.47132500	0.87380500
H	-6.47894300	3.06449500	0.27389100
27	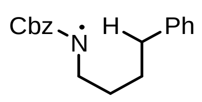	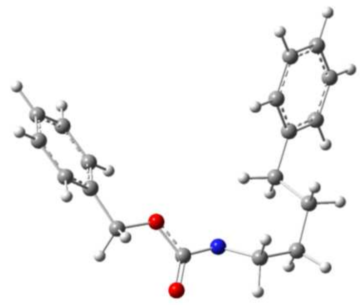	
Cartesian Coordinates			
C	-0.79793700	3.09057200	0.49012200
C	-2.26740200	3.09771100	0.10193300
H	-2.75301500	3.90205500	0.66481500
H	-2.34743200	3.36333100	-0.95788900
C	-3.01125600	1.79084000	0.37629700
H	-4.08701400	1.96746800	0.26904500
H	-2.86083300	1.49842100	1.42328700
C	-2.61601800	0.62176600	-0.53392200
H	-1.54390400	0.42626600	-0.44537400
N	0.01994200	2.31774800	-0.39703000
H	-0.38498800	4.11229400	0.49127900

H	-0.67083300	2.73739300	1.52832200
C	1.37922100	2.36348600	-0.13906100
O	1.89914800	1.13214400	-0.14738400
O	2.01472700	3.38350300	-0.00348000
C	3.32498900	1.06758000	0.02350900
H	3.79145800	1.65540700	-0.77335900
H	3.59959000	1.53170100	0.97391100
C	3.75699300	-0.36684700	-0.02746000
C	4.83048700	-0.78933900	0.75328200
C	3.13533600	-1.27955600	-0.87810100
C	5.28714100	-2.09960700	0.67721000
H	5.31250200	-0.08897200	1.42846700
C	3.58499300	-2.59157500	-0.94638800
H	2.28981300	-0.95944500	-1.47521300
C	4.66417800	-3.00465600	-0.17286300
H	6.12374300	-2.41518400	1.29052600
H	3.09008600	-3.29470800	-1.60705200
H	5.01410500	-4.02929800	-0.22869300
H	-2.78445900	0.91227600	-1.57673700
C	-3.39266300	-0.62844700	-0.22086400
C	-4.65052800	-0.84974400	-0.78403200
C	-2.88980800	-1.58041800	0.66670900
C	-5.38603300	-1.98547100	-0.46884900
H	-5.05426500	-0.12466600	-1.48469300
C	-3.62165900	-2.71782000	0.98640200
H	-1.90793200	-1.43038000	1.10564200
C	-4.87383900	-2.92378300	0.41975900
H	-6.35924700	-2.14111200	-0.92184100
H	-3.21031000	-3.44794100	1.67503700
H	-5.44481800	-3.81245900	0.66455500

28

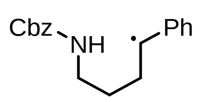

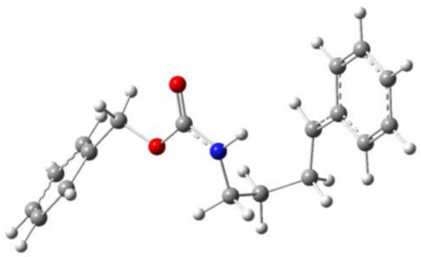

Cartesian Coordinates

C	0.27921600	1.31319300	-1.00795600
C	0.50455500	2.03062300	0.32313900
H	0.12180000	3.05330300	0.23250600
H	-0.10149200	1.53937400	1.09146600
C	1.97352200	2.09575600	0.76434500
H	2.02088500	2.71363000	1.67415200
H	2.54756400	2.63943300	0.00759700
C	2.58494500	0.76988900	1.04802600
N	0.48875300	-0.12225800	-0.96294500
H	-0.74104700	1.48957800	-1.34934200
H	0.95039200	1.71901400	-1.77164000
H	1.43353100	-0.47329000	-0.98814700
C	-0.41190000	-1.03128200	-0.50726400
O	-1.62861600	-0.46391500	-0.31937700
O	-0.17530100	-2.20101500	-0.30650300
C	-2.65269700	-1.37475100	0.11198100
H	-2.66512300	-2.23317200	-0.56435100
H	-2.41024500	-1.74543100	1.11059300
C	-3.96013100	-0.64365200	0.10565200

C	-4.64967000	-0.40869200	1.29169900
C	-4.51020900	-0.19398700	-1.09559100
C	-5.87108600	0.25675900	1.28224000
H	-4.22717400	-0.75102900	2.23103800
C	-5.72462000	0.47660000	-1.10813200
H	-3.97810700	-0.37197000	-2.02426100
C	-6.40950300	0.70189600	0.08240600
H	-6.39918000	0.42991500	2.21330100
H	-6.14244500	0.82048300	-2.04786400
H	-7.36078600	1.22215100	0.07234700
H	2.03281600	0.11687600	1.71937900
C	3.81553200	0.27719600	0.55386800
C	4.23681700	-1.03046600	0.91064200
C	4.67263400	1.01860300	-0.29810100
C	5.42687700	-1.55470400	0.44859600
H	3.59679500	-1.62373900	1.55560200
C	5.86343000	0.48388200	-0.75333300
H	4.39993000	2.02514600	-0.59263500
C	6.25304400	-0.80300300	-0.38720900
H	5.71853700	-2.55854900	0.73745000
H	6.50107600	1.07590300	-1.40112200
H	7.18781000	-1.21552500	-0.74846400

29

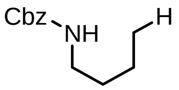

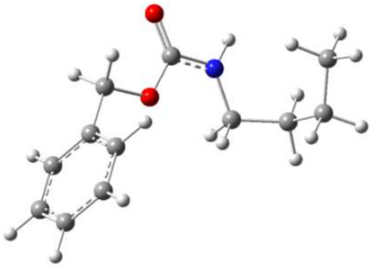

Cartesian Coordinates

C	-2.21324600	-0.25223500	1.24948700
C	-3.52864500	-1.02172500	1.20715000
H	-3.50676700	-1.72162400	2.04922300
H	-4.36128600	-0.33600900	1.41652400
C	-3.81537800	-1.80792000	-0.07418800
H	-4.61723000	-2.52248500	0.14198400
H	-2.93488700	-2.41288800	-0.32197100
C	-4.22065400	-0.97159000	-1.28361600
H	-3.42169700	-0.29987300	-1.60004900
N	-2.21524700	0.90259600	0.36743800
H	-3.05511500	1.45633100	0.30687600
H	-2.01173800	0.05794200	2.28367500
H	-1.38862400	-0.90043400	0.94827300
C	-1.11733100	1.54009300	-0.11663900
O	0.00954600	0.83422600	0.13447000
O	-1.13889500	2.59306000	-0.71356800
C	1.21379100	1.42449500	-0.38705000
H	1.05344000	1.68234700	-1.43592600
H	1.42107300	2.34943500	0.15617100
C	2.32151200	0.43115500	-0.22039800
C	3.22023100	0.53553900	0.83842200
C	2.45805900	-0.62658700	-1.11933200
C	4.24003800	-0.39574000	0.99673700
H	3.12039800	1.35493000	1.54316200
C	3.47274900	-1.56062200	-0.96308100
H	1.76317000	-0.71408900	-1.94839800
C	4.36709000	-1.44596600	0.09623300

H	4.93555800	-0.30067200	1.82315100
H	3.57068600	-2.37658200	-1.67051800
H	5.16318900	-2.17227400	0.21652400
H	-5.10148200	-0.36088800	-1.05926000
H	-4.47647900	-1.61299700	-2.13107100

30	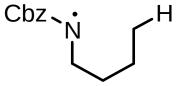	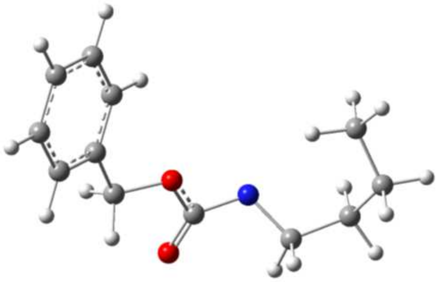

Cartesian Coordinates

C	3.29702900	-0.80112100	0.30370000
C	4.47916200	0.12001000	0.04954500
H	5.36984000	-0.35692900	0.47256900
H	4.63996000	0.18744600	-1.03196900
C	4.33683600	1.51938900	0.64970600
H	5.30202500	2.02991100	0.56212700
H	4.14649400	1.42402000	1.72646700
C	3.25465600	2.38276300	0.01253600
H	2.26346200	1.93337200	0.10723300
N	2.15873800	-0.49763800	-0.51247200
H	3.56269200	-1.84805800	0.08315500
H	3.02241000	-0.80240900	1.37236000
C	1.06053900	-1.31791500	-0.32404000
O	-0.05484500	-0.58832300	-0.21374100
O	1.09810700	-2.52690300	-0.33511400
C	-1.27247100	-1.34280700	-0.09670100
H	-1.35557700	-1.99600400	-0.97108900
H	-1.22061900	-1.98499500	0.78587600
C	-2.42611500	-0.38978000	-0.01082700
C	-3.52265000	-0.70333500	0.78876700
C	-2.44302400	0.78930600	-0.75442000
C	-4.62624300	0.14004600	0.83741600
H	-3.51293800	-1.61316000	1.38108000
C	-3.54131300	1.63680800	-0.69831700
H	-1.58500700	1.04513100	-1.36470400
C	-4.63743100	1.31340000	0.09404700
H	-5.47280800	-0.11577900	1.46473300
H	-3.54156300	2.55445800	-1.27607100
H	-5.49417100	1.97653900	0.13580000
H	3.44219800	2.51980400	-1.05616300
H	3.22248700	3.37135400	0.47827000

31	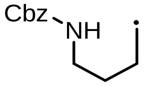	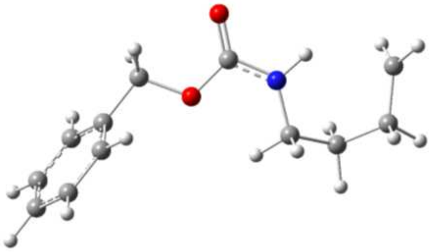

Cartesian Coordinates

C	-2.16558500	-0.98611500	-0.15724000
C	-3.34786200	-1.54651800	0.62580800

H	-3.19293400	-2.62531100	0.73021000
H	-3.34092100	-1.12614700	1.63757700
C	-4.71694600	-1.31175500	-0.02556400
H	-5.44748800	-1.95888100	0.49006600
H	-4.69666800	-1.67419200	-1.06057500
C	-5.20752200	0.08943500	0.01672200
N	-2.16549900	0.46241500	-0.20034600
H	-1.23094700	-1.29954500	0.30669200
H	-2.17382400	-1.40311300	-1.17473800
H	-3.03334700	0.93408000	-0.41242700
C	-1.08508200	1.27903100	-0.14804500
O	0.05798500	0.57781100	0.05411500
O	-1.12630000	2.48426200	-0.25742500
C	1.24414100	1.38022200	0.16700500
H	1.23813800	2.12628700	-0.63075100
H	1.22957200	1.91329100	1.12098700
C	2.43035900	0.47043300	0.07346500
C	3.24186800	0.23967400	1.18067100
C	2.73717700	-0.16319500	-1.13148700
C	4.34483700	-0.60253300	1.08917200
H	3.00785600	0.72444100	2.12320500
C	3.83292000	-1.00877400	-1.22521300
H	2.10843500	0.00952700	-1.99879300
C	4.64106600	-1.22954000	-0.11378900
H	4.97012000	-0.77037200	1.95901200
H	4.06223000	-1.49385000	-2.16761700
H	5.49999900	-1.88714500	-0.18811200
H	-5.10788600	0.67098800	0.92722000
H	-5.88354000	0.46426900	-0.74208300

32

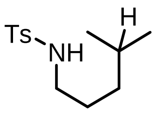

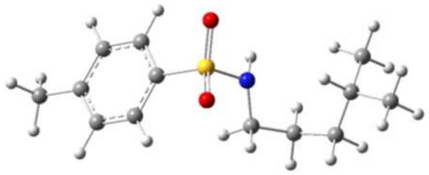

Cartesian Coordinates

C	-1.02674800	-1.11749400	-0.51625900
C	-2.21219600	-1.93933200	-0.02117500
H	-2.08025400	-2.94863200	-0.42566400
H	-2.14908000	-2.05438300	1.06791800
C	-3.59565800	-1.43696200	-0.43557800
H	-4.32513000	-2.22774500	-0.21415600
H	-3.60406400	-1.31893300	-1.52641000
C	-4.09840700	-0.13524900	0.20110100
H	-3.37051200	0.65328100	-0.01394300
N	-0.90308000	0.14486000	0.21279600
C	-5.43299800	0.26178800	-0.42483700
C	-4.23793400	-0.25310000	1.71642200
H	-3.28751500	-0.47973800	2.20706100
H	-4.60765500	0.68104000	2.14745400
H	-4.94601100	-1.04610700	1.98296000
H	-6.19566700	-0.50359000	-0.24118800
H	-5.80125900	1.20330700	-0.00827200
H	-5.34276500	0.38950900	-1.50726400
H	-0.87308300	0.05289800	1.22181600
H	-0.10681000	-1.71324500	-0.42999700
H	-1.16122300	-0.86455100	-1.57052500
S	0.19922600	1.27877000	-0.30454300
O	-0.02525300	1.44445300	-1.72950300

O	0.09573700	2.37332300	0.64336800
C	1.81466000	0.54531600	-0.10667800
C	2.40443100	-0.12078600	-1.17370800
C	2.45268300	0.62193200	1.12696300
C	3.64343800	-0.72171800	-0.99479100
H	1.90325200	-0.14122000	-2.13383800
C	3.68979800	0.01740200	1.28714900
H	1.99282900	1.17490800	1.93767900
C	4.30331500	-0.66490500	0.23305400
H	4.10886300	-1.23885000	-1.82751200
H	4.19389000	0.08194300	2.24597900
C	5.65741400	-1.28762000	0.40840200
H	6.44734500	-0.54968300	0.23440700
H	5.79049100	-1.67512200	1.42057100
H	5.81363000	-2.10726100	-0.29527600

33	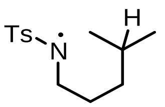	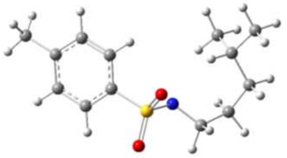

Cartesian Coordinates

C	2.06731700	-1.55134500	-0.73650900
C	3.17516300	-0.76965800	-1.43112300
H	3.87117800	-1.49475200	-1.86573400
H	2.73956100	-0.21755800	-2.26935200
C	3.96013200	0.16061600	-0.50715400
H	4.79418600	0.59115500	-1.07697000
H	4.41863100	-0.44550300	0.28486200
C	3.17841800	1.30570000	0.14503600
H	2.34974600	0.87357100	0.72053800
N	0.87621400	-0.75534400	-0.57134800
C	4.08018100	2.05427700	1.12397100
C	2.58637000	2.26067700	-0.88729300
H	1.85346200	1.76248100	-1.52499600
H	2.07869300	3.09634700	-0.39720200
H	3.37184600	2.68032900	-1.52609100
H	4.92852100	2.51077800	0.60186400
H	3.53479000	2.85403900	1.63238200
H	4.48106700	1.38461500	1.89008300
H	1.77193200	-2.41596800	-1.35327000
H	2.41466000	-1.97879600	0.21598700
S	-0.17182200	-1.37643300	0.59056500
O	-0.43807700	-2.78197600	0.30957800
O	0.37115900	-0.96763000	1.88209900
C	-1.64844900	-0.45001400	0.26510100
C	-2.64957600	-1.02477000	-0.50379600
C	-1.78072800	0.83237100	0.78591800
C	-3.80269400	-0.29429000	-0.75820300
H	-2.52779000	-2.03363400	-0.87868300
C	-2.93752400	1.54528700	0.51987300
H	-0.99418500	1.25051100	1.40185400
C	-3.96333400	0.99682100	-0.25686500
H	-4.59285800	-0.73797100	-1.35436200
H	-3.05118000	2.54548100	0.92508900
C	-5.21158100	1.78382700	-0.52904200
H	-5.74157500	2.01008200	0.40068500
H	-4.97731600	2.73814300	-1.00880100
H	-5.89453100	1.23683900	-1.18039500

34

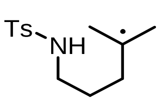

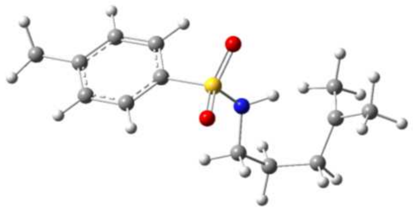

Cartesian Coordinates

C	1.23027400	-0.29991300	1.47090800
C	2.29979600	-1.37991400	1.55072500
H	2.34763000	-1.72168700	2.58930300
H	1.98849100	-2.24311800	0.95422200
C	3.69949100	-0.90724000	1.13207100
H	4.41702400	-1.69610700	1.41946800
H	3.96700800	-0.03206100	1.73561100
C	3.89377900	-0.58673800	-0.32025900
N	0.96986600	0.05733900	0.07510500
C	4.81479600	0.52872100	-0.67943600
C	3.67634500	-1.66500700	-1.32775600
H	2.78512700	-2.26178100	-1.11778600
H	3.57885600	-1.25794200	-2.33787700
H	4.52950900	-2.36418100	-1.34791300
H	5.87113200	0.22380900	-0.58481300
H	4.67520100	0.85267400	-1.71463000
H	4.67973800	1.39587200	-0.02669300
H	0.29601000	-0.67545000	1.89933200
H	1.53133400	0.57707900	2.05742700
S	-0.08888500	1.31415200	-0.21813100
O	-0.06818300	2.28241500	0.87213000
O	0.18475600	1.72358300	-1.58709300
C	-1.66968600	0.49515300	-0.15596400
C	-2.53251100	0.75282300	0.89785900
C	-2.02497300	-0.37777900	-1.17998300
C	-3.77003300	0.11914000	0.92755200
H	-2.23547100	1.45188300	1.67014000
C	-3.26045900	-0.99910800	-1.13426000
H	-1.34204600	-0.55367200	-2.00220000
C	-4.15133700	-0.76322900	-0.08062500
H	-4.45109500	0.31946500	1.74796700
H	-3.54564000	-1.67821000	-1.93135800
C	-5.48884200	-1.44343100	-0.05310100
H	-6.09317700	-1.15367300	-0.91769100
H	-5.37818500	-2.53100000	-0.08588300
H	-6.04853100	-1.18840800	0.84800000
H	1.82776100	0.21985100	-0.45694000

35

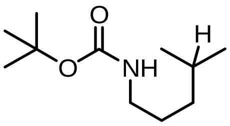

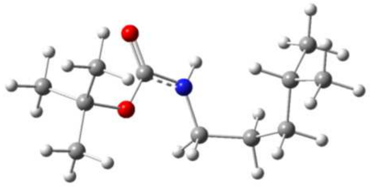

Cartesian Coordinates

C	-0.73036000	-1.66472700	-0.39273000
C	-2.24977200	-1.75456000	-0.49195800
H	-2.47298000	-2.57079900	-1.18722700
H	-2.66096400	-2.07808500	0.47252400
C	-2.96632800	-0.49853500	-0.99207400

H	-4.00466400	-0.76711900	-1.22939000
H	-2.51004100	-0.20163200	-1.94511400
C	-2.99702300	0.72193600	-0.06351400
H	-1.96515300	0.98824300	0.18889700
N	-0.29672900	-0.86944200	0.74267600
C	-3.62880300	1.90737600	-0.78918500
C	-3.74565900	0.43700200	1.23600000
H	-3.29228200	-0.37559800	1.81012900
H	-3.76073000	1.32000900	1.88047500
H	-4.78508300	0.15609600	1.03219900
H	-4.66546600	1.68722500	-1.06788700
H	-3.63822300	2.80013700	-0.15780900
H	-3.08333300	2.15183700	-1.70525100
H	-0.79654700	-0.96983400	1.61220800
H	-0.31243500	-2.67865600	-0.33002500
H	-0.32060400	-1.20965900	-1.29599600
C	0.92064500	-0.27837700	0.89883800
O	1.62366900	-0.32041300	-0.24983500
O	1.28732400	0.22542600	1.93959600
C	2.94386900	0.30398500	-0.33448700
C	3.35016400	0.03273400	-1.77512400
C	2.83255100	1.80138100	-0.07917800
C	3.90611000	-0.37426300	0.63208600
H	2.10445500	2.24787700	-0.76131200
H	2.52566200	1.99819500	0.94680300
H	3.80102600	2.27579500	-0.25771300
H	4.91544400	0.01583900	0.47670500
H	3.61095600	-0.19376300	1.66439200
H	3.92645100	-1.45185300	0.44994500
H	3.38412600	-1.04164900	-1.96827400
H	2.63764500	0.48694100	-2.46717300
H	4.33953800	0.45273200	-1.96996800

36	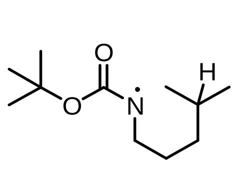	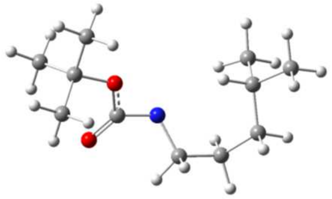

Cartesian Coordinates

C	-1.09951900	-1.71831000	-0.42588400
C	-2.58597000	-1.71389400	-0.10842200
H	-3.02780500	-2.60589800	-0.56579000
H	-2.71049100	-1.82831900	0.97269600
C	-3.34270000	-0.48699300	-0.61800000
H	-4.41567000	-0.64366100	-0.44501100
H	-3.22116000	-0.43340900	-1.70839400
C	-2.95108200	0.86687200	-0.01530300
H	-1.87981400	1.02673300	-0.19115200
N	-0.34493000	-0.82380900	0.40297500
C	-3.71836100	1.98612200	-0.71514500
C	-3.17719900	0.91350200	1.49263800
H	-2.54116900	0.19694700	2.01594800
H	-2.94481100	1.90620900	1.88825000
H	-4.22244300	0.69414900	1.73924500
H	-4.79719600	1.87756200	-0.55679800
H	-3.42501300	2.96710000	-0.33133500
H	-3.53947100	1.98154400	-1.79452400
H	-0.66803600	-2.72171100	-0.28315700
H	-0.92810300	-1.48481900	-1.49129000

C	1.03079300	-0.86940000	0.22655800
O	1.50927100	0.35939300	0.04876700
O	1.66887000	-1.89629000	0.30815000
C	2.95570000	0.59816500	-0.08064000
C	3.02653800	2.10423700	-0.27190300
C	3.65886800	0.17790900	1.20222000
C	3.49430800	-0.13266000	-1.30256300
H	3.20748700	0.67846500	2.06214600
H	3.60047800	-0.89989600	1.34728100
H	4.71080300	0.47057800	1.15119300
H	2.47826300	2.40414700	-1.16733700
H	2.59283000	2.61978500	0.58719500
H	4.06724300	2.41806800	-0.37891300
H	4.53910600	0.14639300	-1.46085900
H	3.43668900	-1.21265500	-1.17284000
H	2.92940900	0.15122800	-2.19405400
37	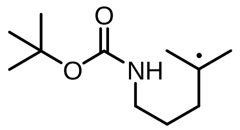	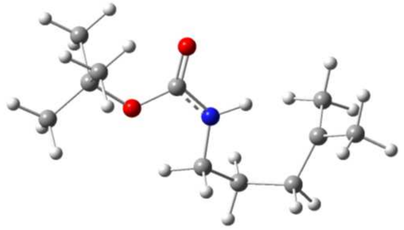	
Cartesian Coordinates			
C	0.75109500	1.40789800	-0.23953900
C	2.10641300	1.80281600	0.33307400
H	2.21798200	2.88407600	0.20313000
H	2.10733200	1.61932600	1.41248300
C	3.30753600	1.11269900	-0.33076500
H	4.21895000	1.64581300	-0.00623500
H	3.24561900	1.27090000	-1.41413100
C	3.48350000	-0.34992600	-0.05139200
N	0.44073500	0.01842000	0.03059000
C	4.01270800	-1.21983800	-1.13959000
C	3.68979300	-0.79366200	1.35721200
H	3.05407400	-0.25518300	2.06441000
H	3.49382500	-1.86317100	1.47581200
H	4.73252400	-0.62611200	1.67685000
H	5.10264900	-1.09612600	-1.26143900
H	3.84196700	-2.27998200	-0.93086000
H	3.56148900	-0.98256600	-2.10751200
H	-0.03315800	2.02078300	0.20599800
H	0.73877000	1.61151200	-1.32073000
H	1.20097600	-0.64683300	-0.02329500
C	-0.79498800	-0.54639700	0.03755900
O	-1.75483200	0.39858900	-0.05743300
O	-0.98521900	-1.74122700	0.13246600
C	-3.16739700	0.02586000	-0.02313600
C	-3.50268200	-0.86777300	-1.21053700
C	-3.50464000	-0.63636700	1.30661200
C	-3.87079800	1.36919600	-0.14712400
H	-3.20839700	-0.38047200	-2.14373300
H	-2.98908900	-1.82460100	-1.13216100
H	-4.58128600	-1.04297800	-1.24345700
H	-4.58290100	-0.80590000	1.36802000
H	-2.98958200	-1.59051800	1.40570600
H	-3.21466500	0.01349700	2.13630700
H	-3.59884700	2.02256600	0.68483500

H	-3.59222000	1.86310700	-1.08059600
H	-4.95399700	1.22798300	-0.13804300

7.4 Electronic Properties of Radicals

DFT Method: UB3LYP/6-311+G(d,p)

No.	Radical	Ionization Potential (IP, eV)	Electron affinity (EA, eV)	Electronegativity (χ, eV)	Electronic Chemical Potential (μ, eV)	Chemical Hardness (η, eV)	Chemical Softness (S, meV)	Global Electrophilicity Index (ω, eV)	Local Electrophilicity Index (ω^{+}_{rc}, eV)	Hirshfeld Charge
1	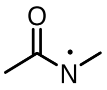	10.36	1.69	6.03	-6.03	8.67	115.38	2.10	1.09	-0.077097
2	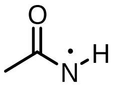	11.48	2.05	6.76	-6.76	9.43	106.03	2.43	1.38	-0.012609
3	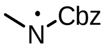	9.40	1.55	5.48	-5.48	7.85	127.31	1.91	1.05	-0.076816
4	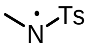	9.81	2.05	5.93	-5.93	7.76	128.82	2.27	1.31	-0.100788
5	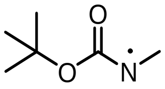	9.88	1.31	5.60	-5.60	8.56	116.79	1.83	1.03	-0.082883
6	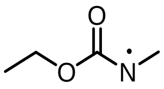	10.19	1.45	5.82	-5.82	8.73	114.53	1.94	1.10	-0.079256
7	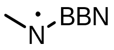	7.37	0.95	4.16	-4.16	6.42	155.80	1.35	0.55	-0.214254
8	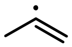	8.62	0.003	4.31	-4.31	8.61	116.10	1.08	0.56	-0.023181

Computed Energies [values are in Hartree]

No.	Species	Total Electronic Energy	Sum of Electronic and Zero-point Energies	Sum of Electronic and Thermal Enthalpies	Gibbs Free Energy
1	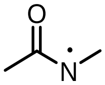	-247.9273344	-247.840528	-247.833081	-247.871657
2	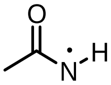	-208.6015663	-208.542704	-208.536940	-208.570681
3	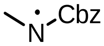	-554.2832106	-554.109276	-554.096877	-554.149694
4	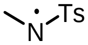	-914.3070131	-914.139233	-914.125609	-914.181148
5	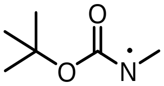	-441.1567648	-440.980738	-440.968630	-441.017949
6	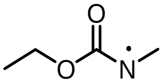	-362.5043557	-362.383442	-362.373951	-362.417946
7	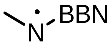	-432.819094	-432.569474	-432.557091	-432.607368
8	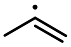	-117.2656483	-117.200573	-117.195319	-117.226230

Optimized Structures and Cartesian Coordinates

No.	Species	Optimized Structure
1	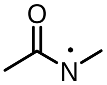	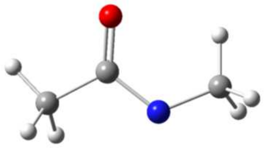

Cartesian Coordinates

C	0.47057200	0.15139700	-0.04295200
O	0.40514900	1.37396700	-0.03448700
N	-0.64343000	-0.61730100	-0.33983000
C	1.75733300	-0.61871000	0.11517500
C	-1.92008000	-0.18370900	0.15698400
H	-2.27195200	-0.93903100	0.87539400
H	-1.88473400	0.79469900	0.64483700
H	-2.64921500	-0.18026300	-0.65920600
H	2.09743500	-0.95382700	-0.86883600
H	2.51723100	0.02402300	0.55809700
H	1.60709900	-1.51010700	0.72918000

No.	Species	Optimized Structure
2	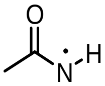	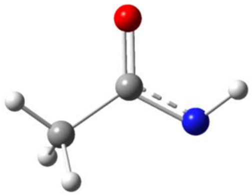

Cartesian Coordinates

C	-0.09202300	0.06000100	-0.03170100
O	-0.62304600	1.17144800	0.01993300
N	-0.90063900	-1.04099000	0.06974500
C	1.39424000	-0.15620500	-0.01492800
H	1.67077200	-0.96772400	-0.69218400
H	1.90789700	0.76278100	-0.29466000
H	1.70198500	-0.44872100	0.99294300
H	-1.80511200	-0.85377000	-0.37400500

No.	Species	Optimized Structure
3	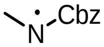	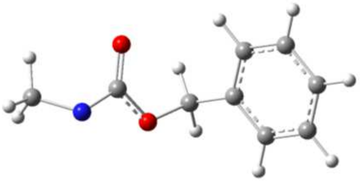

Cartesian Coordinates

C	4.27876800	0.16168300	-0.66400700
N	2.90718000	-0.12932900	-0.98564700
C	1.99021200	0.08647400	0.03526400
O	1.19949300	-0.98284600	0.19877200
O	1.88828300	1.13968400	0.63297600
C	0.11019600	-0.86253700	1.16725900
H	-0.01259600	-1.87772700	1.54229000
H	0.43351200	-0.20720500	1.97528800
C	-1.15464600	-0.36250600	0.52152200
C	-1.46416600	1.00180400	0.51653400
C	-2.03402500	-1.26187000	-0.09055900
C	-2.63275000	1.45645600	-0.09086000
H	-0.78074900	1.70409200	0.97978800
C	-3.20164100	-0.80831000	-0.69904000
H	-1.80220900	-2.32208800	-0.08987400

C	-3.50254700	0.55318800	-0.69992100
H	-2.86365100	2.51579300	-0.08901200
H	-3.87625500	-1.51515100	-1.16888200
H	-4.41208100	0.90805200	-1.17160100
H	4.85165000	0.31268000	-1.57889200
H	4.35827100	1.03796200	-0.00934400
H	4.71524900	-0.69210400	-0.12136900

4	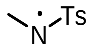	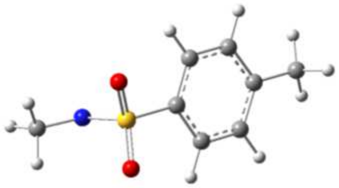

Cartesian Coordinates

C	3.55310800	0.00023300	1.30270400
N	2.10840100	0.00016700	1.20882400
H	3.82952900	0.00060100	2.35646100
H	3.97853700	-0.88307000	0.80730000
H	3.97857900	0.88313100	0.80662500
S	1.56733900	-0.00006500	-0.40918000
O	1.97126400	-1.27302200	-1.01400500
O	1.97128500	1.27268700	-1.01439400
C	-0.20656200	-0.00002600	-0.19559100
C	-0.88302500	-1.21374100	-0.10816900
C	-0.88301900	1.21371100	-0.10833500
C	-2.26176600	-1.20327700	0.07570200
H	-0.33879900	-2.14581000	-0.19343100
C	-2.26175100	1.20328300	0.07554200
H	-0.33879900	2.14577200	-0.19373400
C	-2.97149900	0.00000800	0.17126100
H	-2.79423400	-2.14587500	0.14433600
H	-2.79420400	2.14590000	0.14405200
C	-4.47011800	0.00007100	0.33939900
H	-4.81042200	0.88456400	0.88200400
H	-4.96825700	0.00141100	-0.63618400
H	-4.81076700	-0.88563600	0.87978600

5	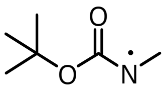	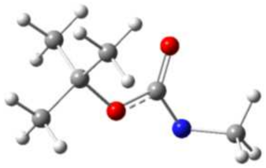

Cartesian Coordinates

C	3.27799700	-0.42257600	0.31863000
N	2.16218700	-0.51541500	-0.58544400
C	1.02161200	0.19163600	-0.20837000
O	-0.04436400	-0.61153900	-0.20985700
O	1.03676500	1.38865800	0.00120100
C	-1.41663200	-0.09829000	0.04137300
H	4.19886800	-0.69888600	-0.19510400
H	3.36337500	0.58545400	0.74268500
H	3.12906900	-1.11950200	1.15889900
C	-2.26235300	-1.36656500	-0.06025900
C	-1.79468200	0.90250300	-1.05208600
C	-1.49917100	0.50600500	1.44440800
H	-1.95308700	-2.09763500	0.68988400
H	-3.31546600	-1.12523500	0.10305100
H	-2.15815400	-1.82036200	-1.04795200
H	-1.66537000	0.45358500	-2.03992700
H	-2.84685200	1.17805300	-0.93969100

H	-1.19035000	1.80661100	-0.99169600
H	-0.89938300	1.41162000	1.52532700
H	-2.53971700	0.75624400	1.66808000
H	-1.15807300	-0.21527900	2.19160600

6	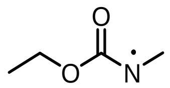	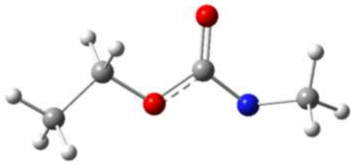

Cartesian Coordinates

C	-2.78441800	-0.50984900	-0.34566800
N	-1.62699100	-0.58757700	0.50467300
C	-0.57618300	0.25897700	0.17090100
O	0.58717500	-0.40373200	0.15245400
O	-0.70225000	1.45607800	0.00691400
C	1.77672400	0.39180500	-0.09631900
H	-3.66379000	-0.87058200	0.18835300
H	-2.94645900	0.50913900	-0.71630700
H	-2.62999700	-1.15842700	-1.22314100
C	2.95849100	-0.55437100	-0.11790000
H	2.85219900	-1.29704500	-0.91165800
H	3.87650200	0.01151400	-0.29756700
H	3.05781100	-1.07773200	0.83532600
H	1.86182800	1.13855600	0.69612800
H	1.65377200	0.91946800	-1.04487200

7	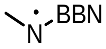	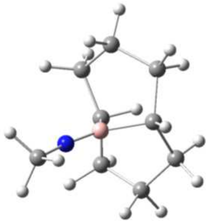

Cartesian Coordinates

C	0.70083800	2.21815700	0.07950200
C	1.11083700	1.36237300	-1.19047900
C	0.66960600	-1.44612300	1.46857200
C	0.32019700	0.00005000	-1.40539400
C	0.70213000	-2.21783400	0.07955000
C	1.11153000	-1.36182300	-1.19051500
H	-0.29905000	2.62177700	-0.10777900
H	2.18957500	1.16609300	-1.17084400
H	-0.17261600	-1.79757100	2.07115200
H	-0.29744000	-2.62220400	-0.10775000
H	1.37032300	3.08163700	0.13806600
H	0.93850300	2.00450000	-2.05962400
H	1.59110100	-1.63172000	2.03463000
H	-0.17844200	-0.00004600	-2.37826600
H	1.37227600	-3.08079700	0.13817500
H	0.93942400	-2.00409800	-2.05959300
H	2.19017700	-1.16501500	-1.17101200
C	0.66887200	1.44648500	1.46855600
H	-0.17346000	1.79753100	2.07122300
H	1.59032900	1.63256000	2.03452200
C	0.57726100	0.00016200	0.99277100
B	-0.62162100	-0.00015600	-0.11516000
H	1.49297800	0.00042000	0.41078600
N	-1.93161800	-0.00062100	0.04800400
C	-3.32235600	-0.00073200	-0.13367000
H	-3.56172200	-0.00078800	-1.21048200

H	-3.79795000 -0.89137300 0.29667300	
H	-3.79806900 0.88993000 0.29653000	
8	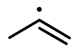	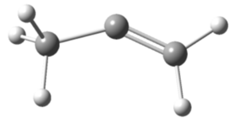

Cartesian Coordinates

C	0.12469500	-0.37734000	0.00016600
C	-1.27194700	0.07570600	-0.00007800
C	1.34458700	0.09714800	0.00015200
H	-1.32946800	1.17551800	0.00079300
H	-1.80676100	-0.28797900	-0.88284900
H	-1.80759900	-0.28932400	0.88168900
H	2.22044000	-0.54613500	-0.00087400
H	1.53937600	1.17483700	-0.00019900

8 NMR Spectra

S1 ^{1}H NMR (400 MHz, $CDCl_3$)

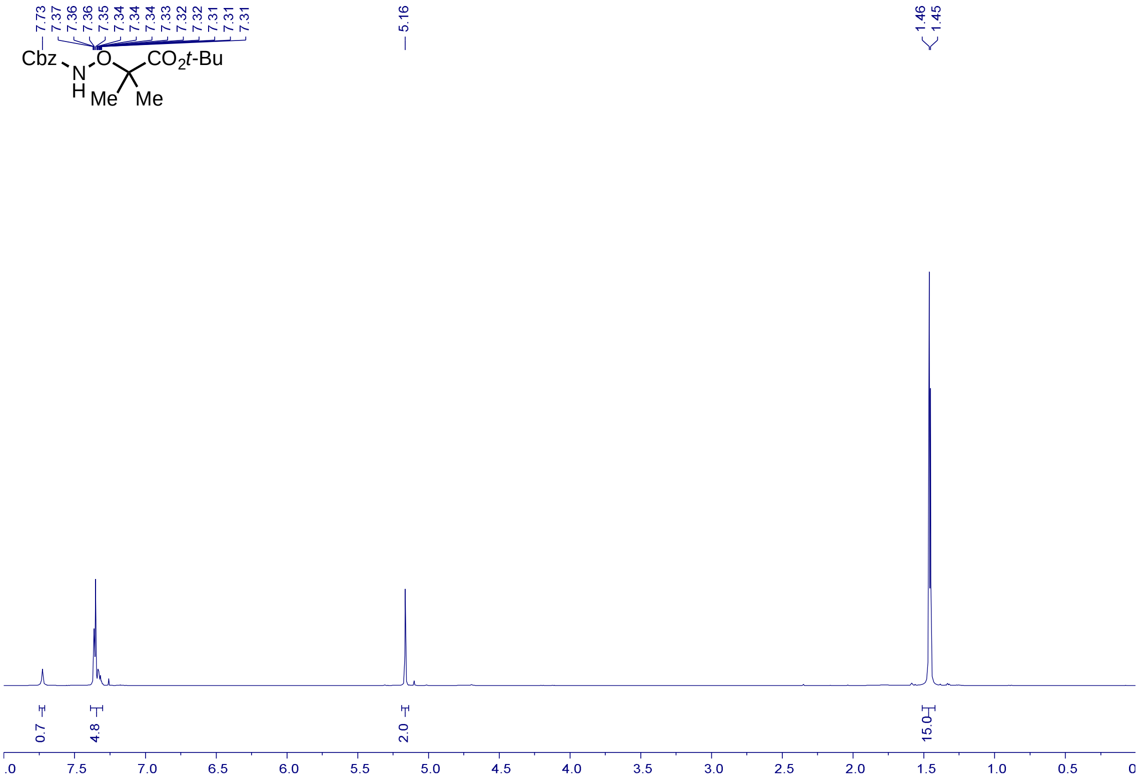

S1 ^{13}C NMR (126 MHz, $CDCl_3$)

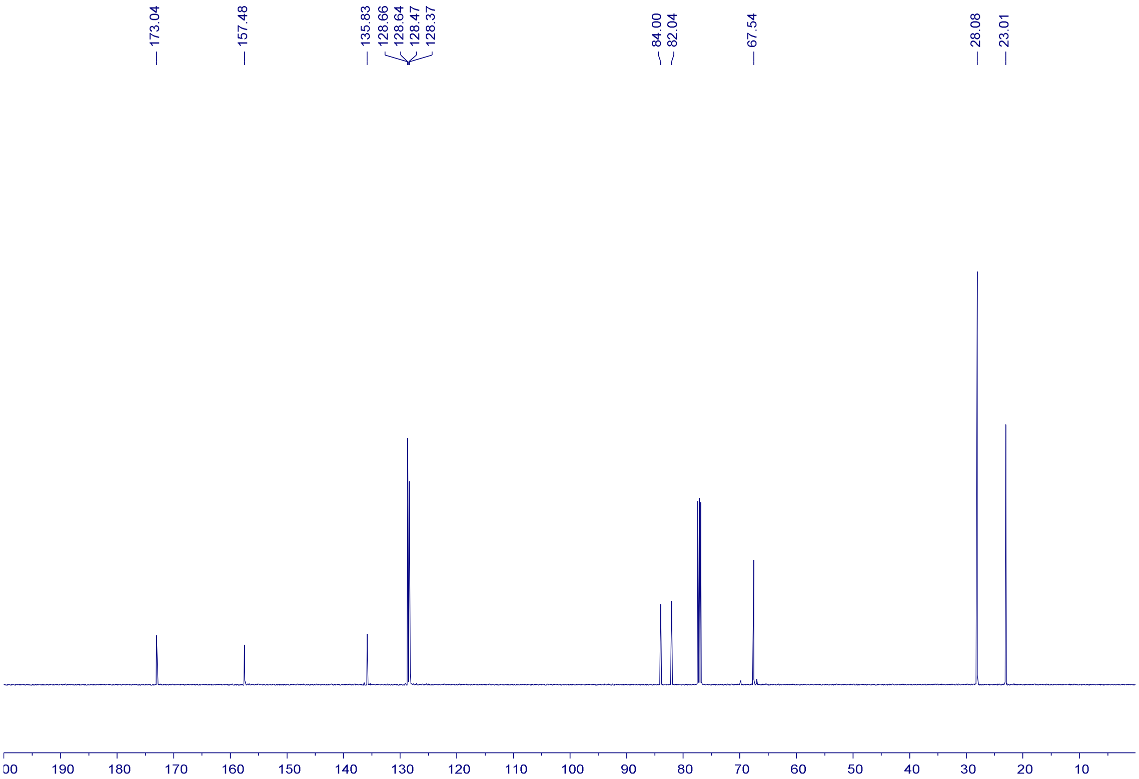

S2 ^{1}H NMR (400 MHz, $CDCl_3$)

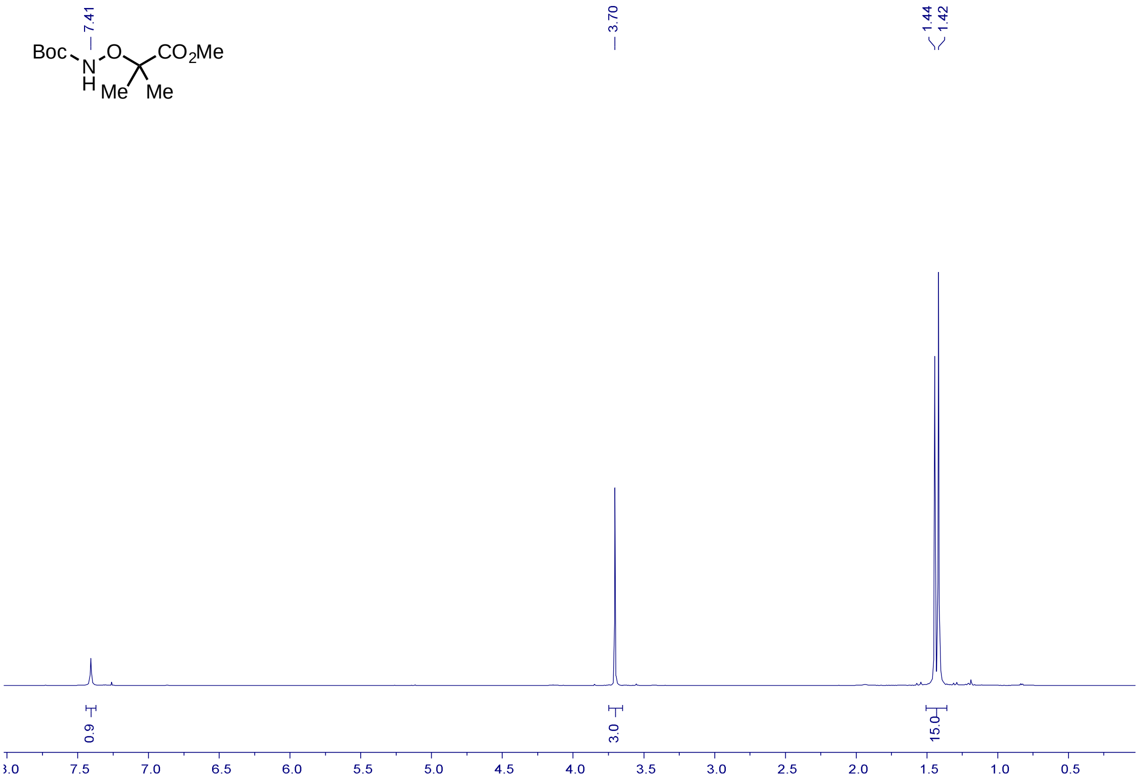

S2 ^{13}C NMR (126 MHz, $CDCl_3$)

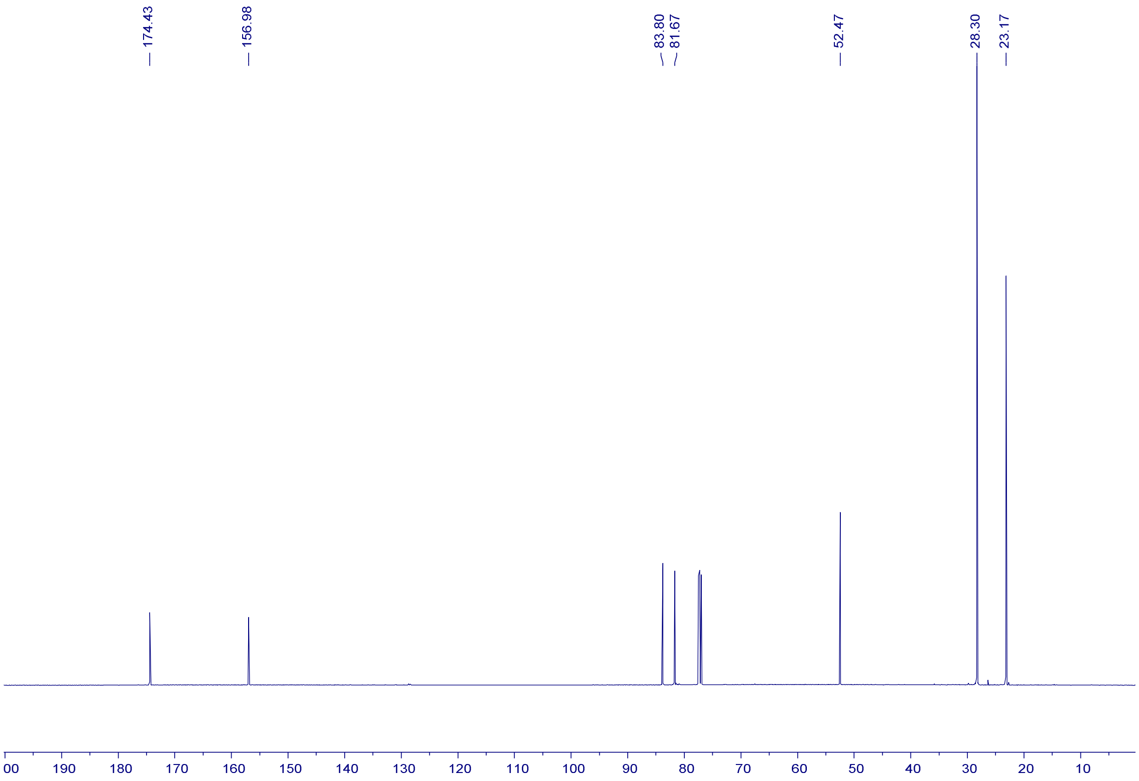

S3 ^{1}H NMR (400 MHz, $CDCl_3$)

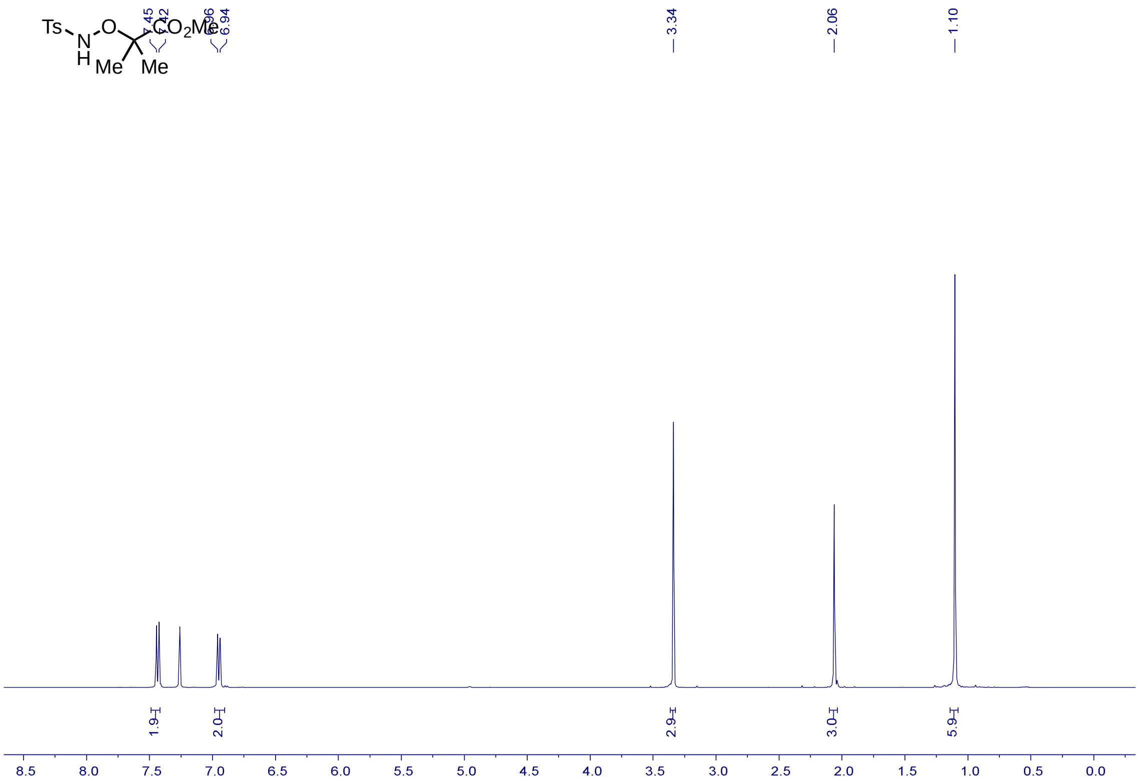

S3 ^{13}C NMR (126 MHz, $CDCl_3$)

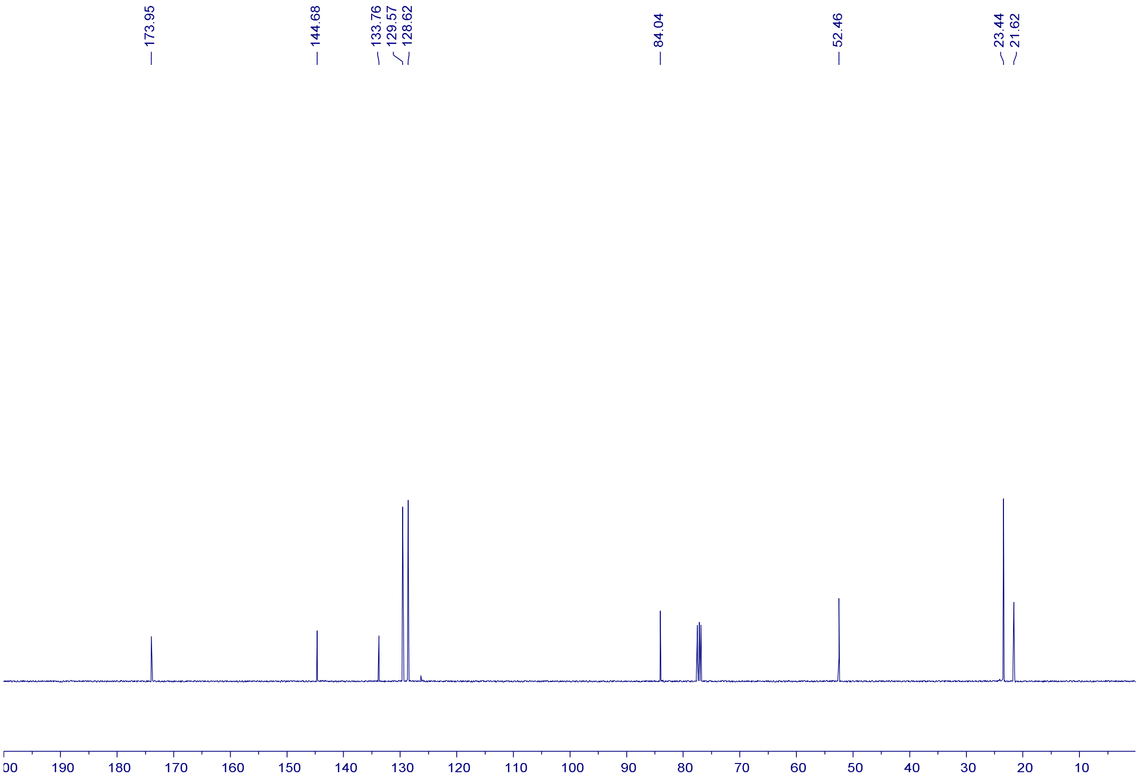

1a ^{1}H NMR (400 MHz, $CDCl_3$)

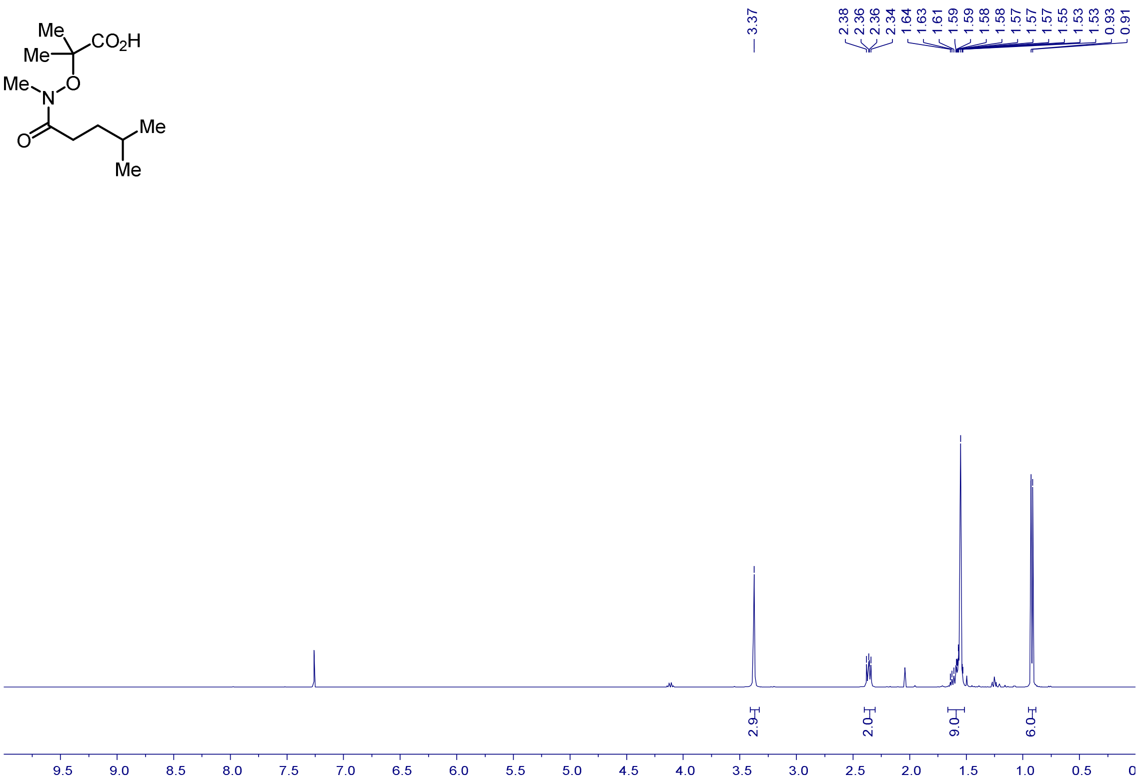

1a ^{13}C NMR (126 MHz, $CDCl_3$)

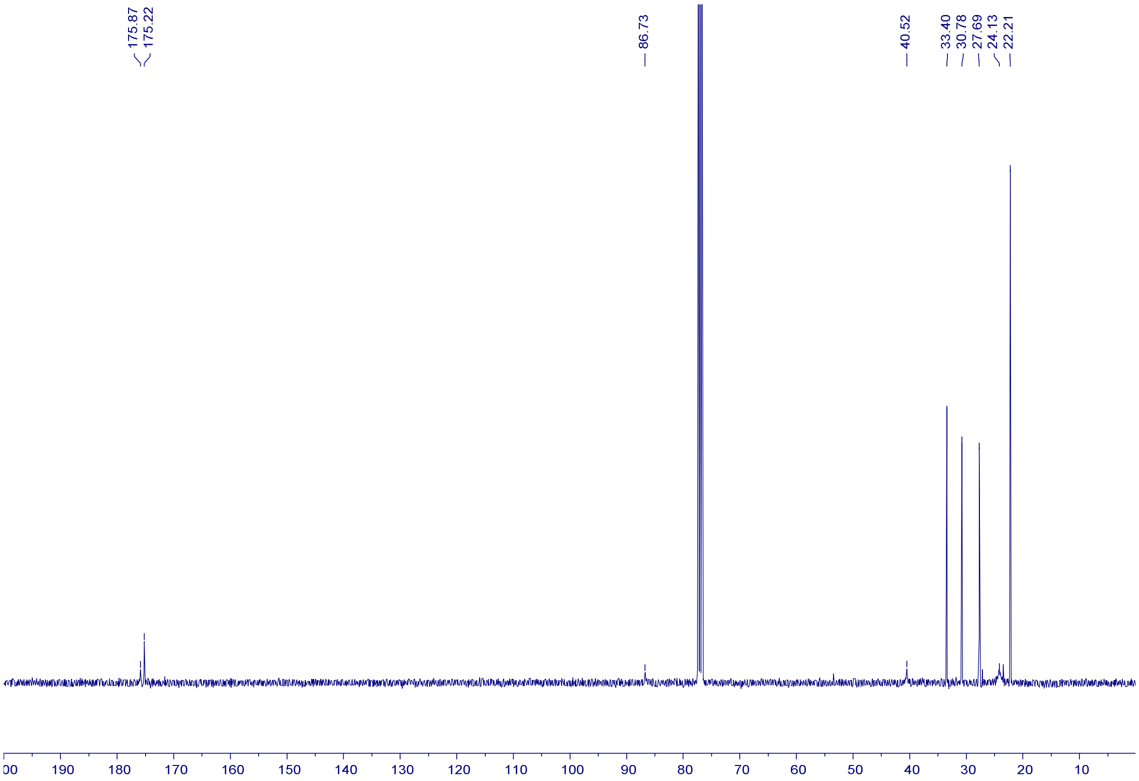

1b ^{1}H NMR (400 MHz, $CDCl_3$)

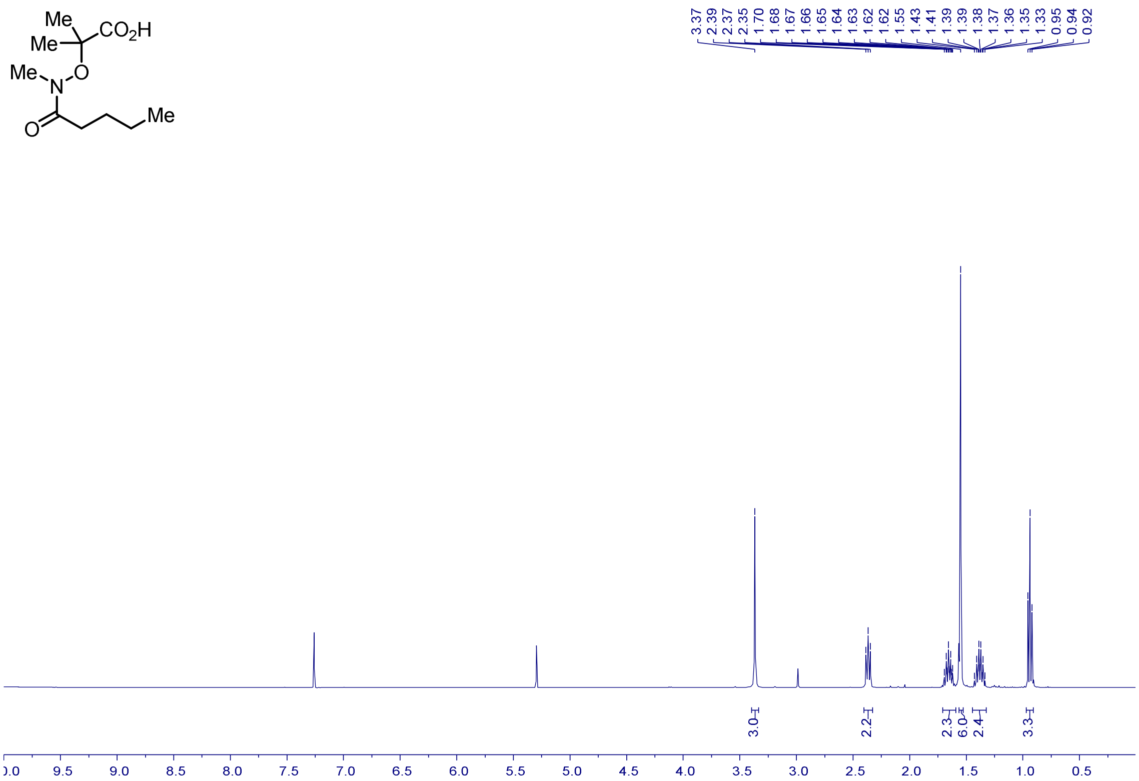

1b ^{13}C NMR (126 MHz, $CDCl_3$)

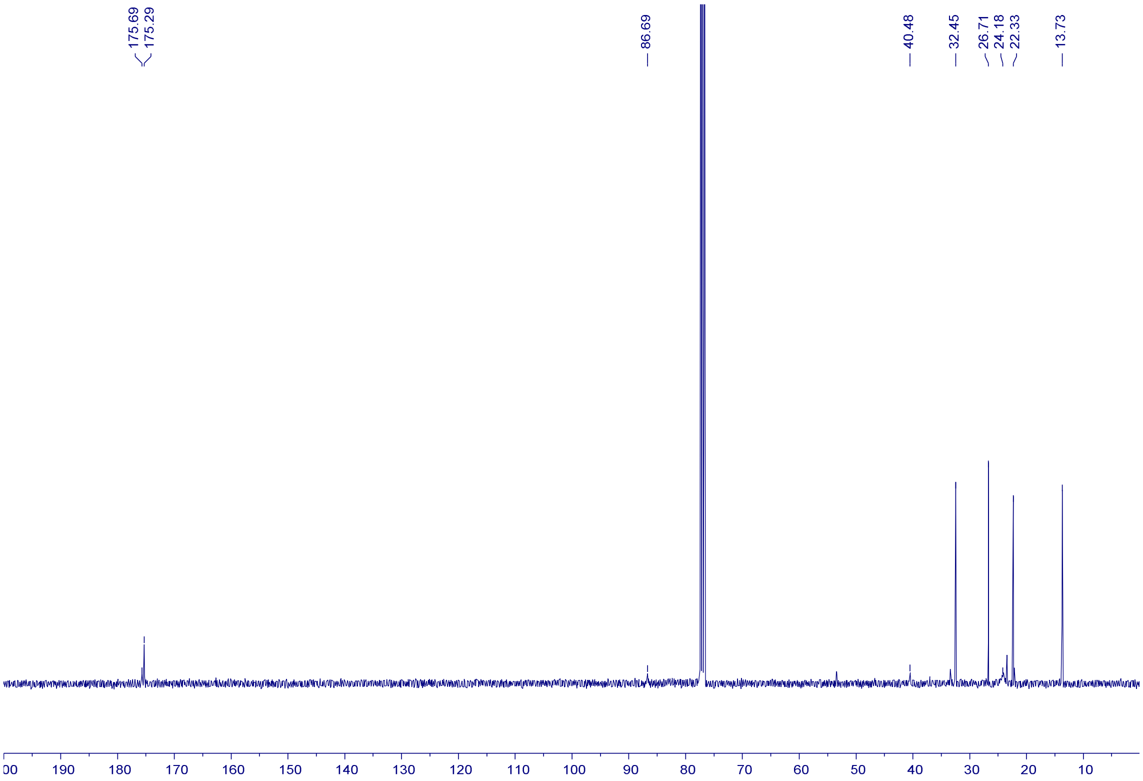

1c ^{1}H NMR (400 MHz, $CDCl_3$)

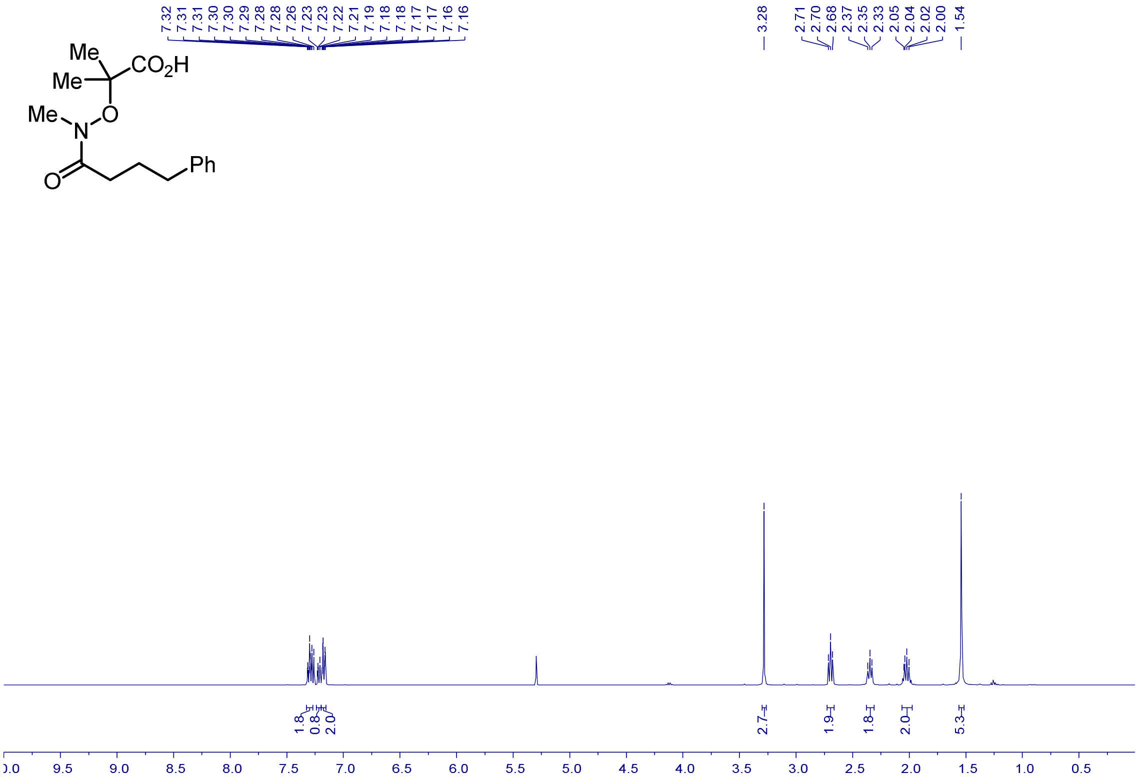

1c ^{13}C NMR (126 MHz, $CDCl_3$)

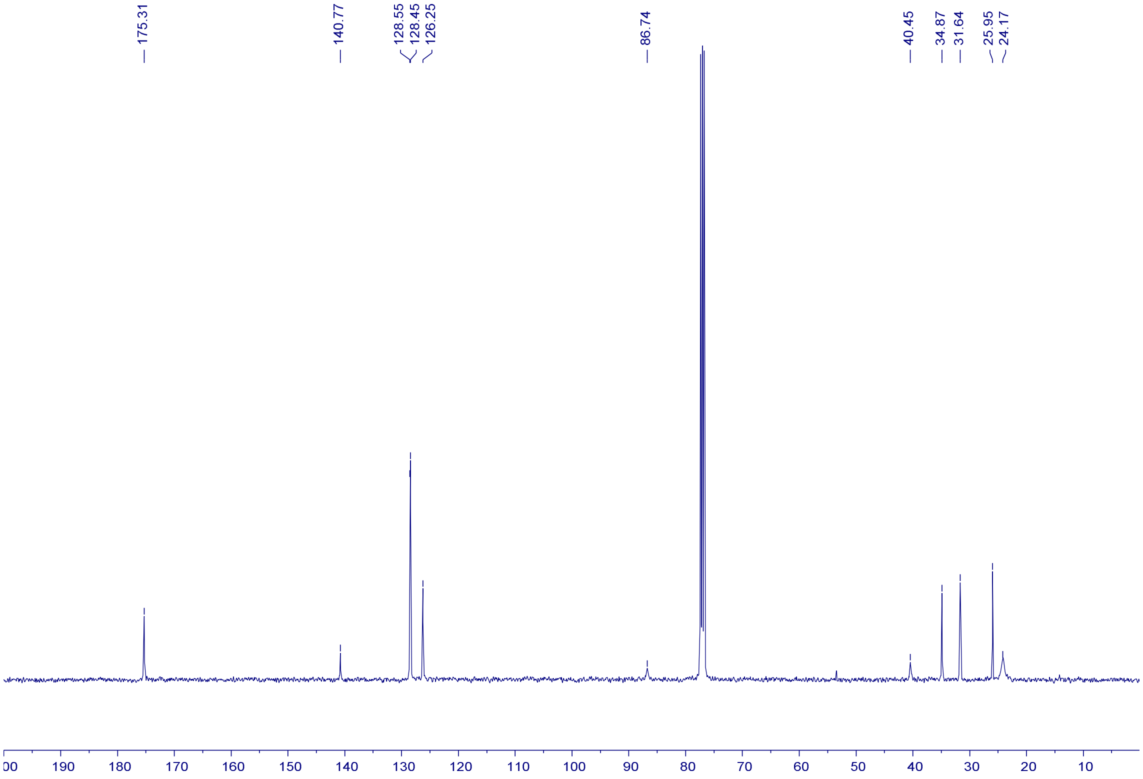

1d ^{1}H NMR (400 MHz, $CDCl_3$)

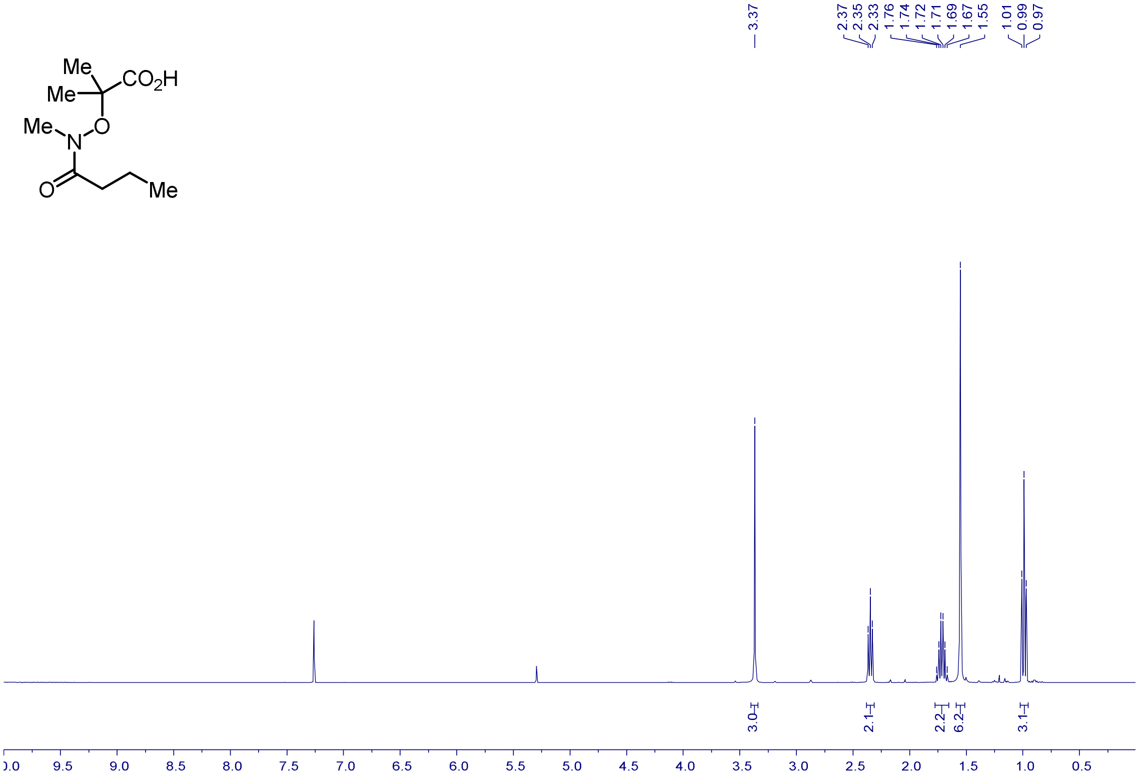

1d ^{13}C NMR (126 MHz, $CDCl_3$)

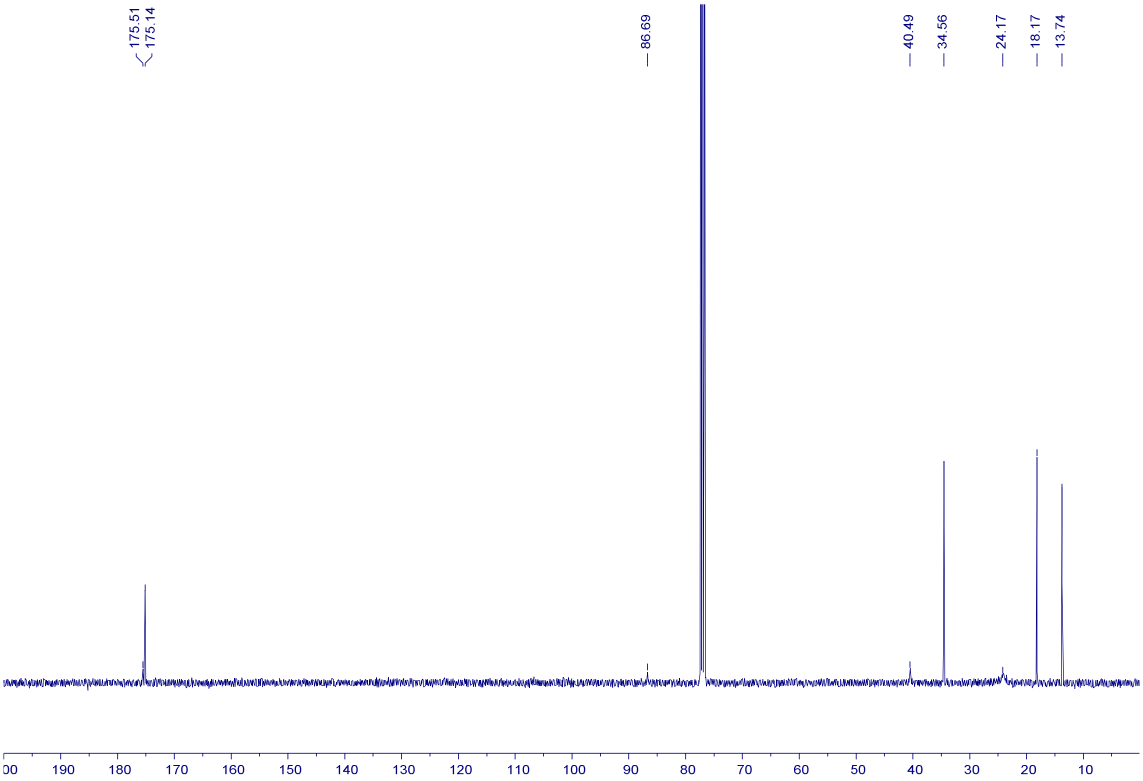

1e ^{1}H NMR (400 MHz, $CDCl_3$)

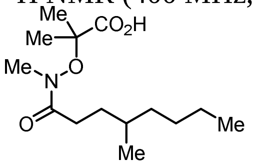

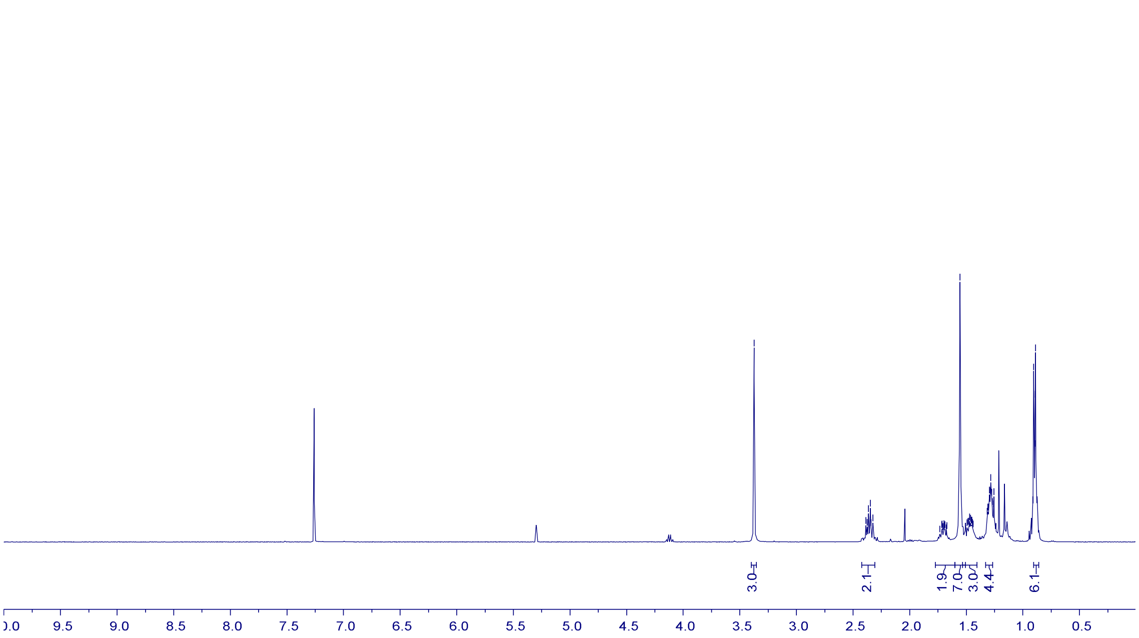

1e ^{13}C NMR (126 MHz, $CDCl_3$)

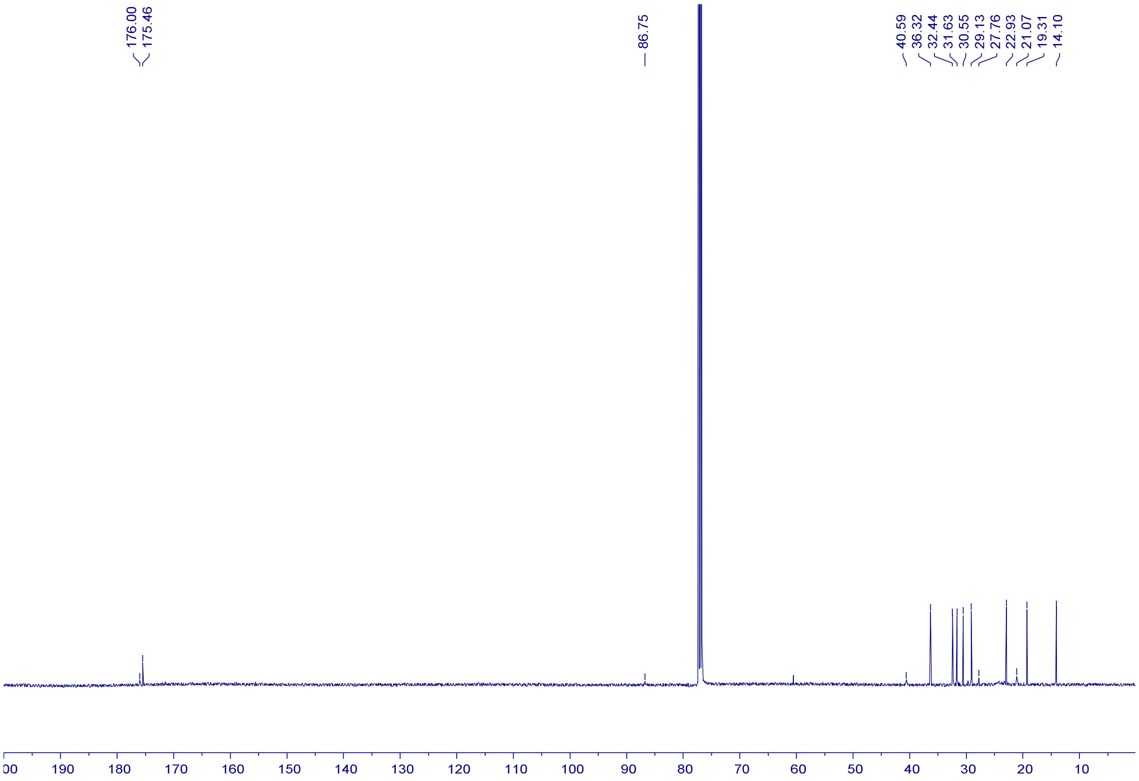

1f ^{1}H NMR (500 MHz, $CDCl_3$)

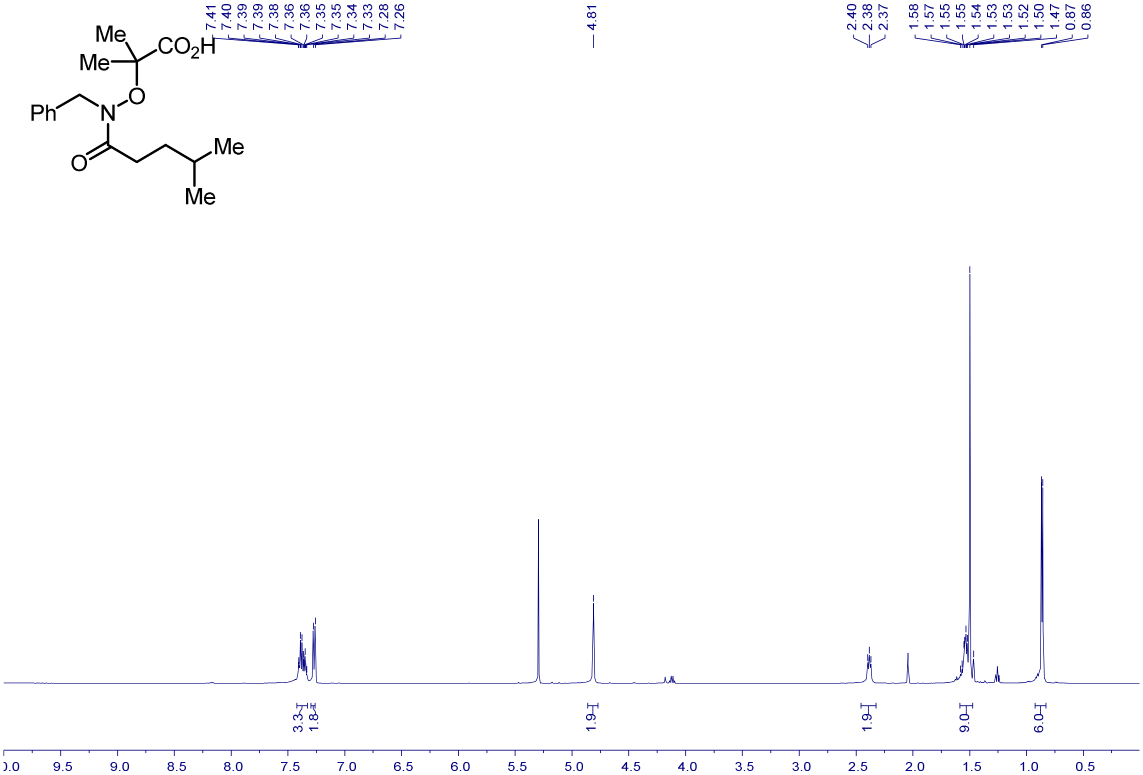

1f ^{13}C NMR (126 MHz, $CDCl_3$)

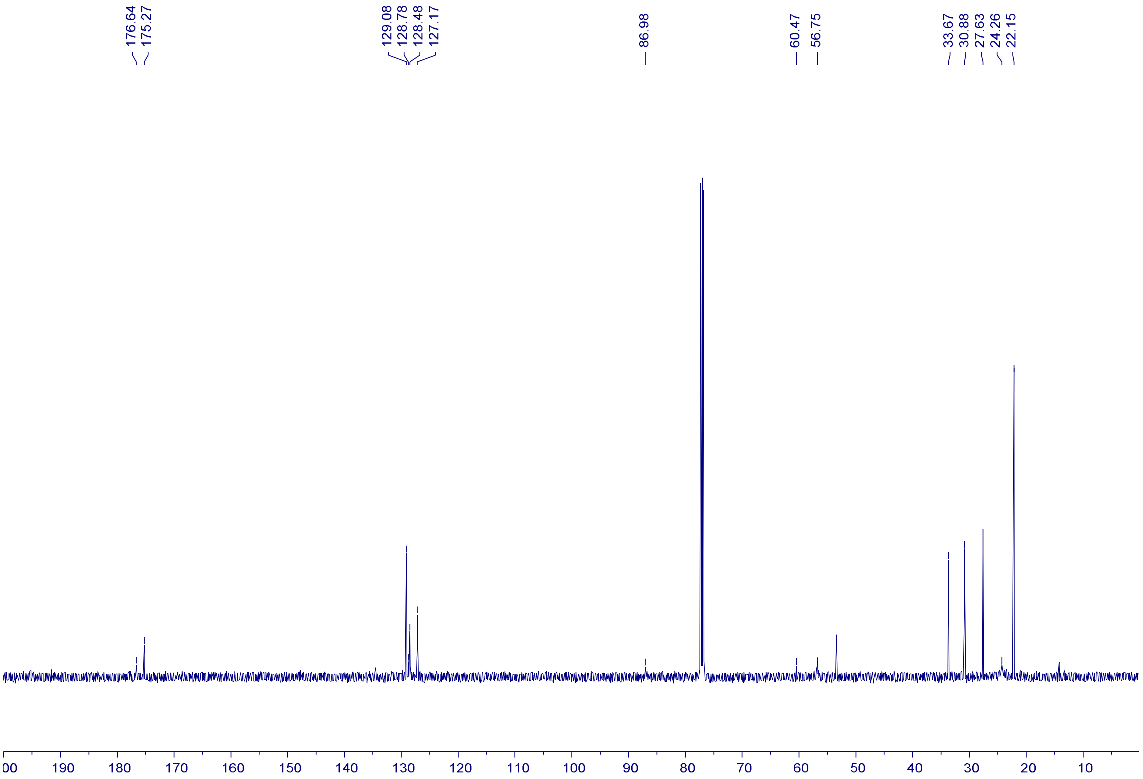

1h ^{1}H NMR (400 MHz, $CDCl_3$)

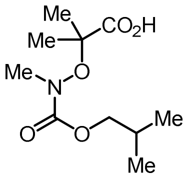

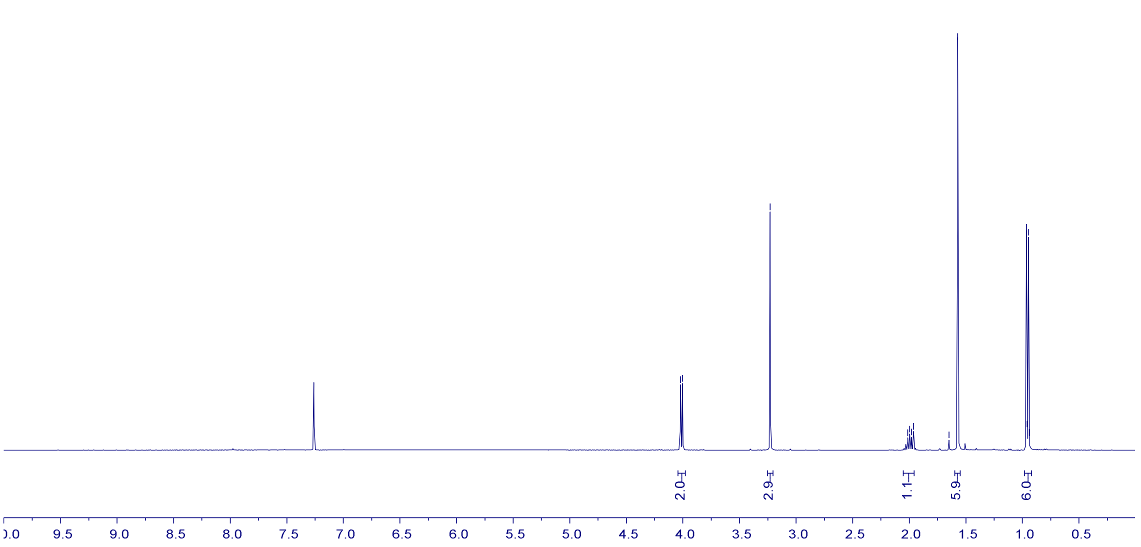

1h ^{13}C NMR (126 MHz, $CDCl_3$)

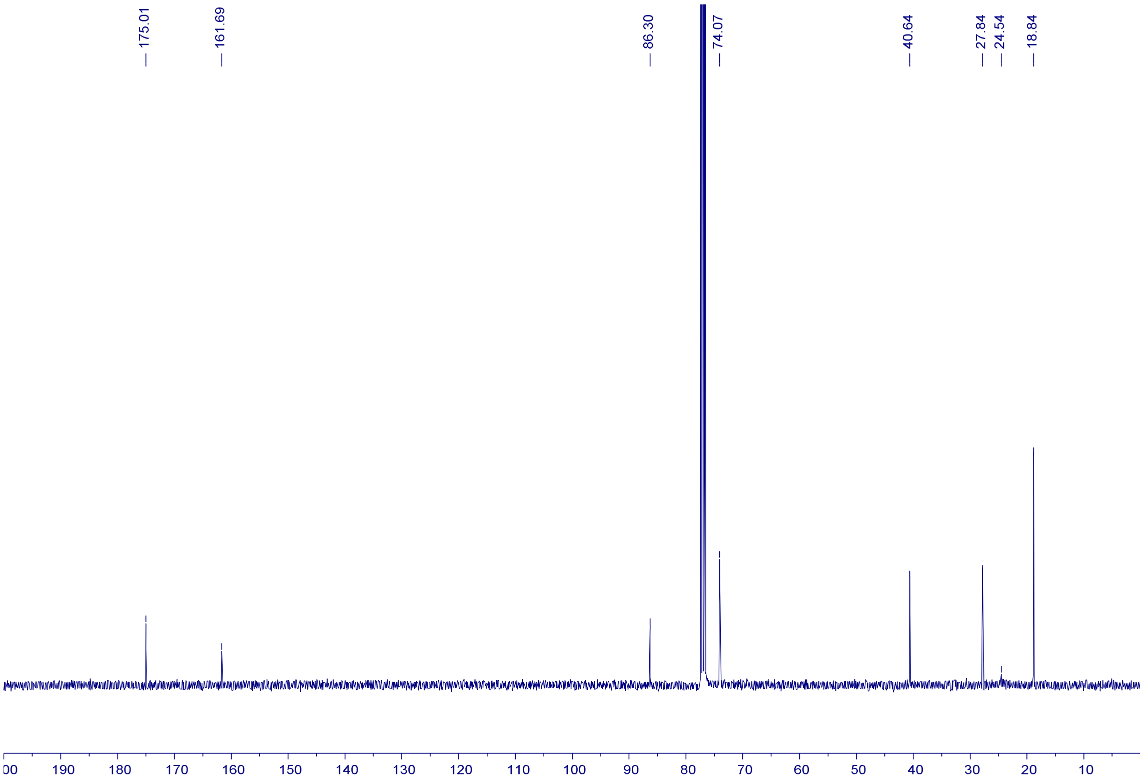

1j ^{1}H NMR (400 MHz, $CDCl_3$)

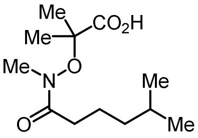

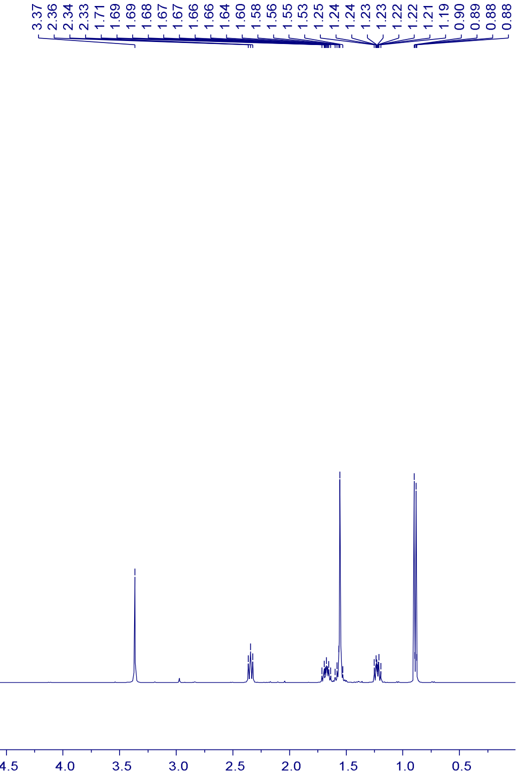

1j ^{13}C NMR (126 MHz, $CDCl_3$)

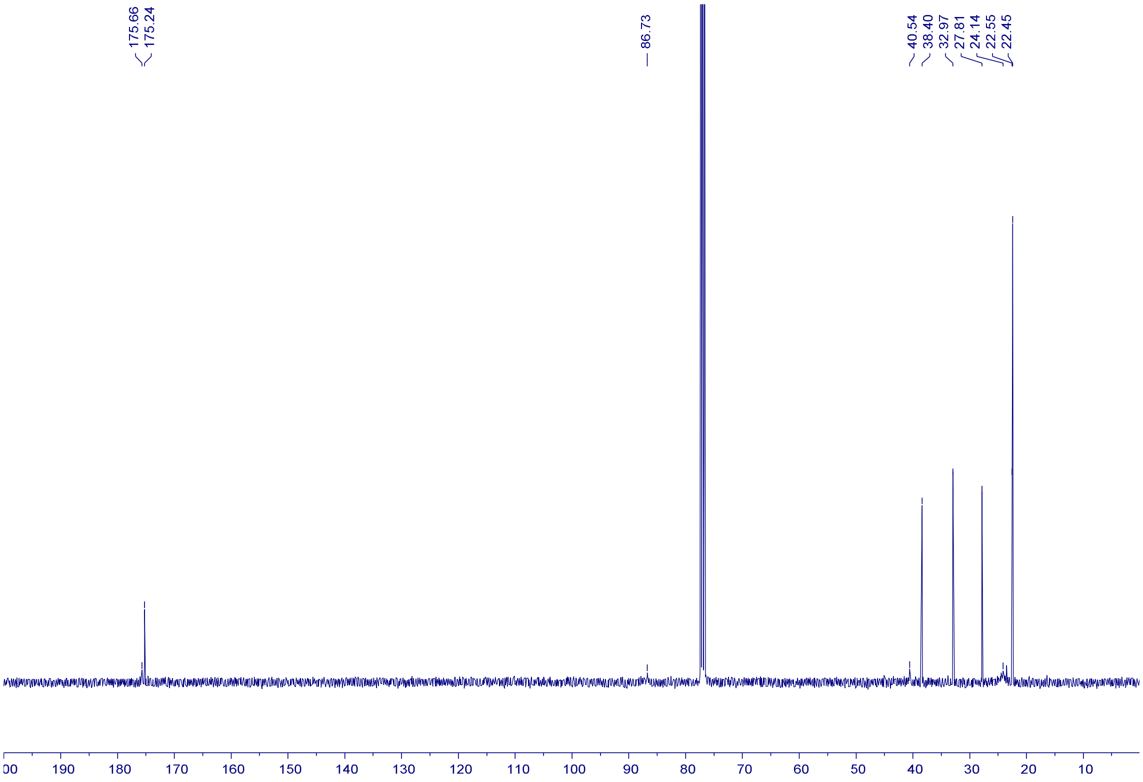

1k ^{1}H NMR (400 MHz, $CDCl_3$)

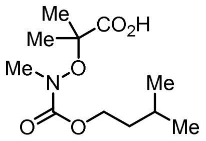

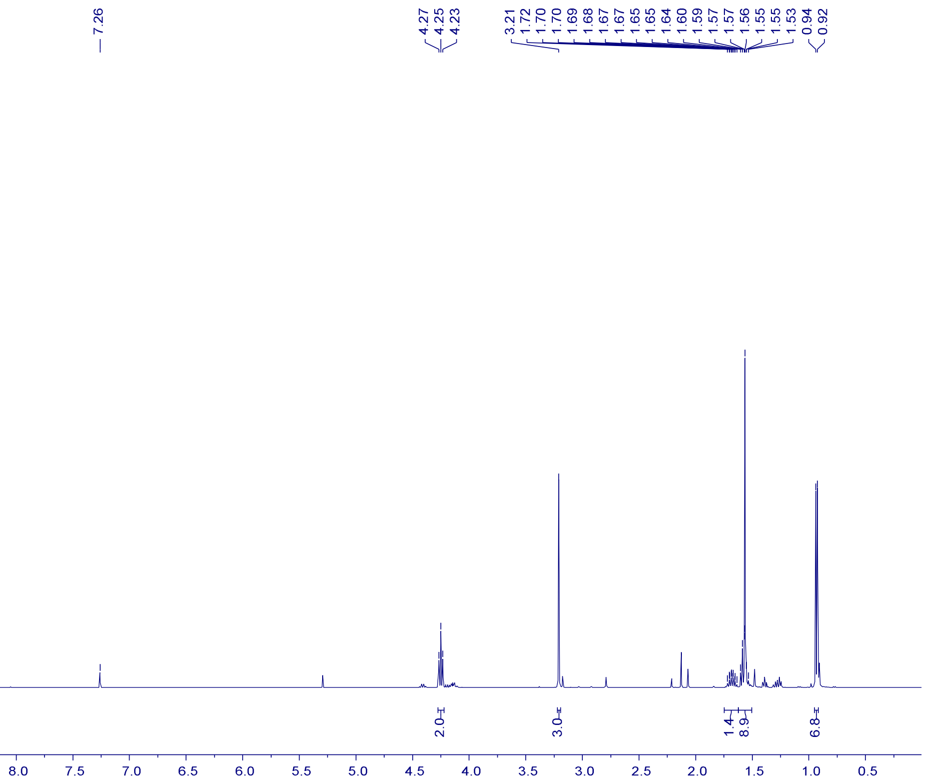

1k ^{13}C NMR (126 MHz, $CDCl_3$)

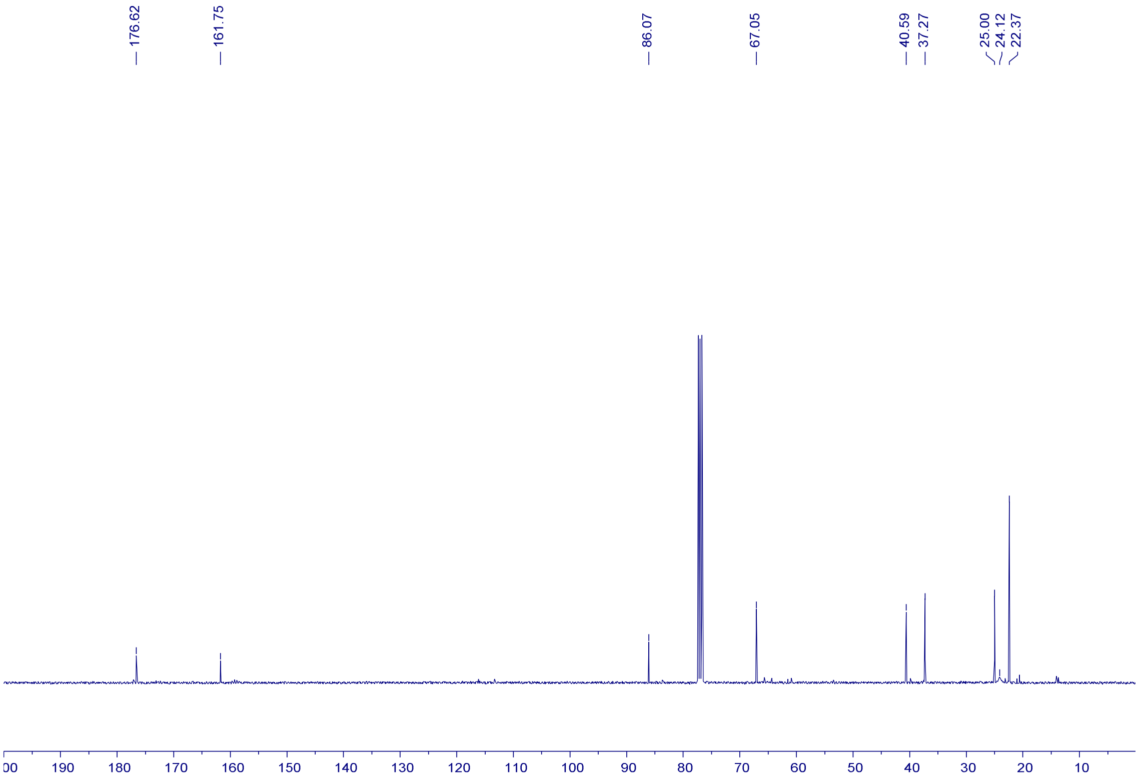

1l ^{1}H NMR (400 MHz, $CDCl_3$)

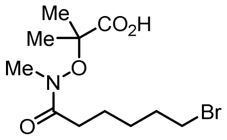

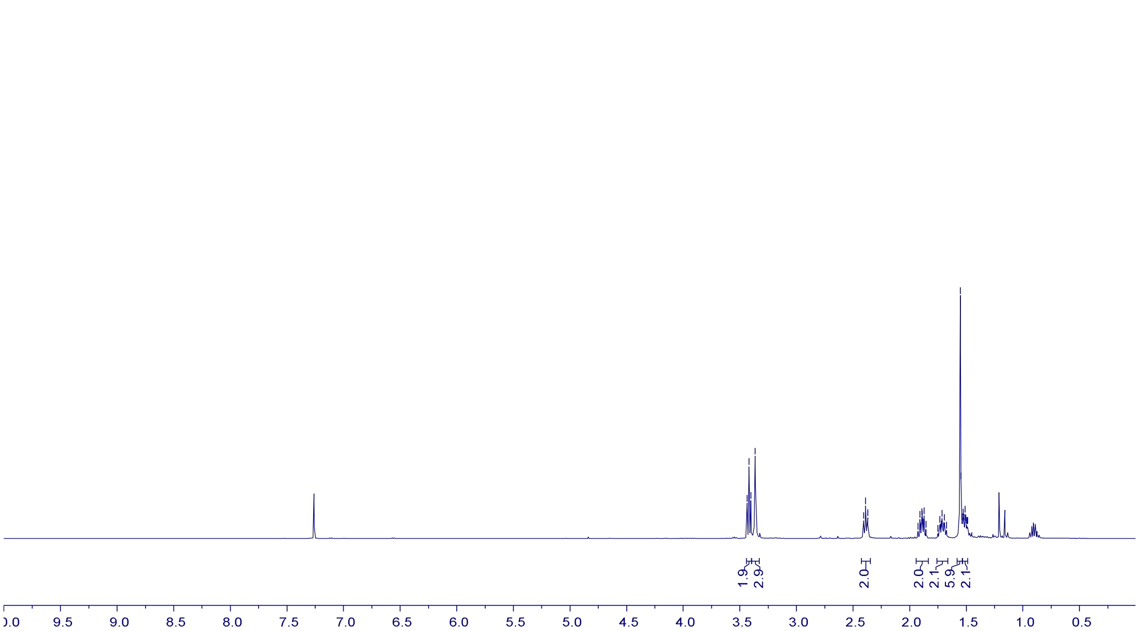

1l ^{13}C NMR (126 MHz, $CDCl_3$)

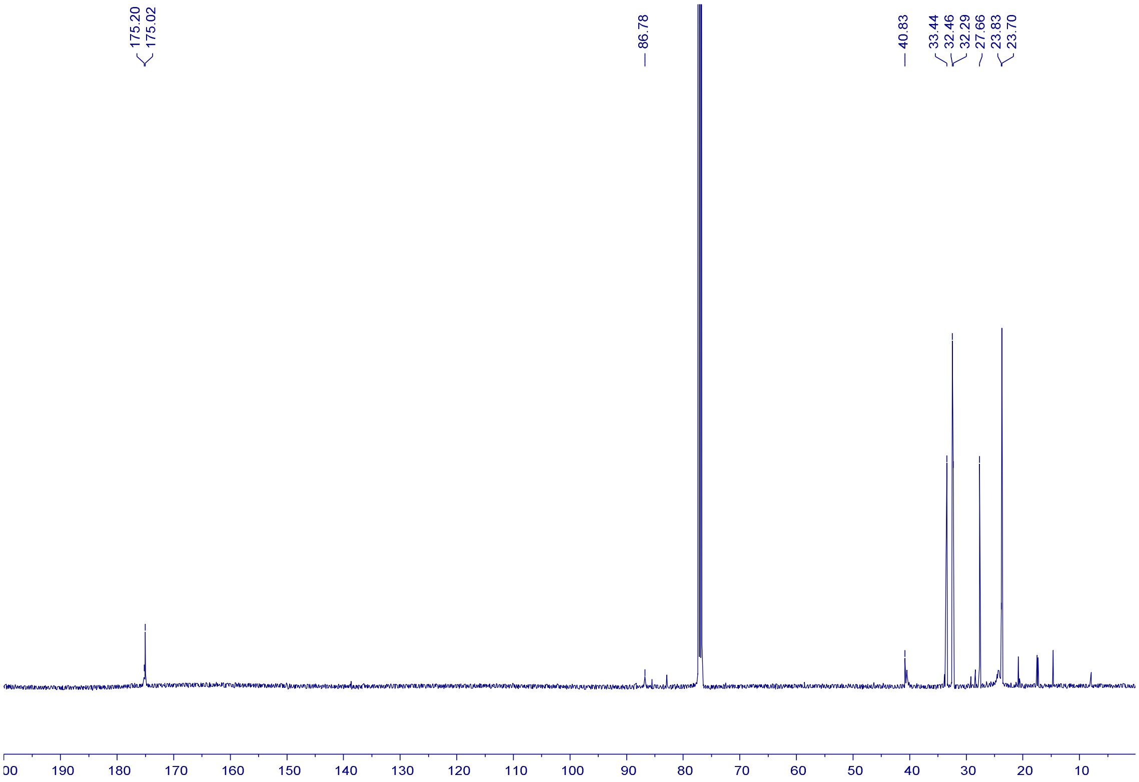

1m ^{1}H NMR (400 MHz, $CDCl_3$)

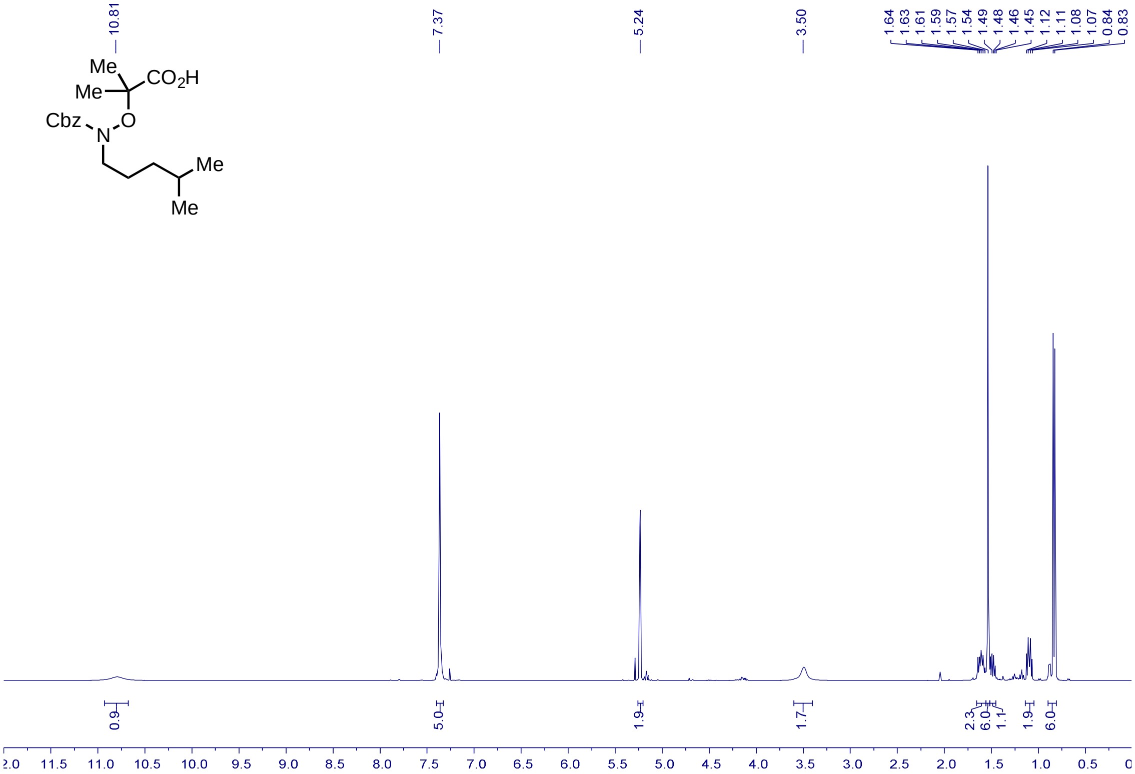

1m ^{13}C NMR (126 MHz, $CDCl_3$)

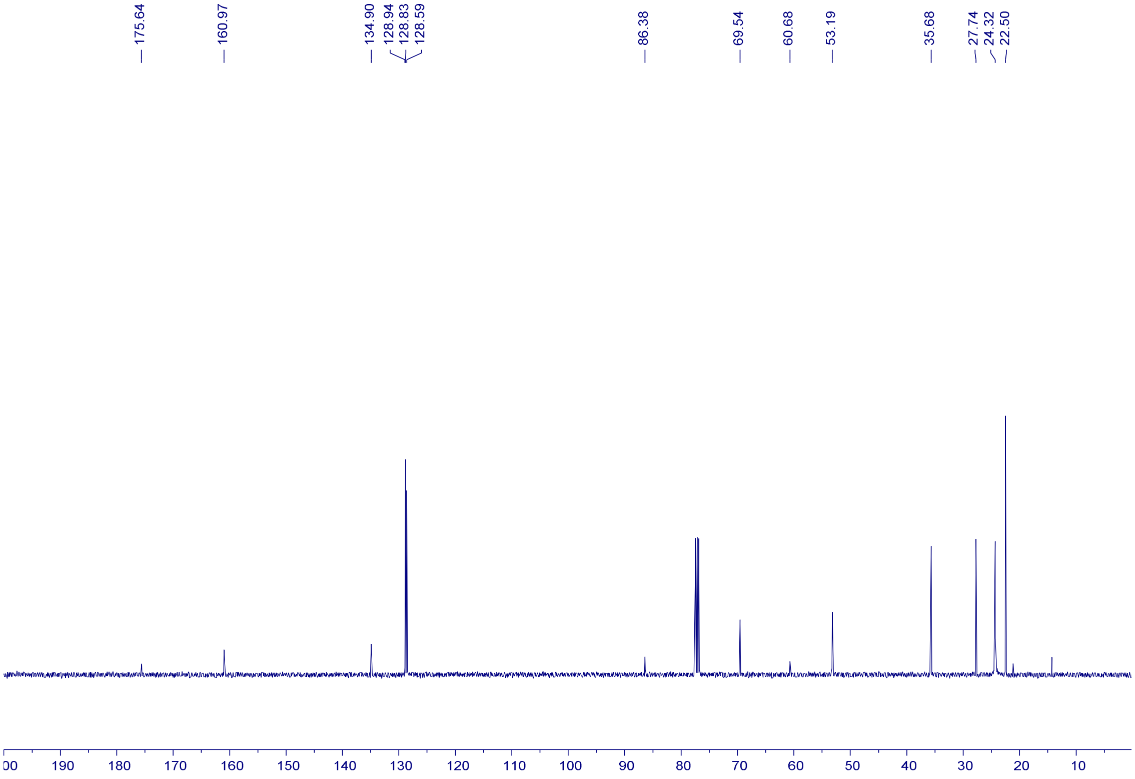

1n ^{1}H NMR (400 MHz, $CDCl_3$)

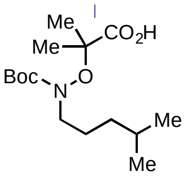

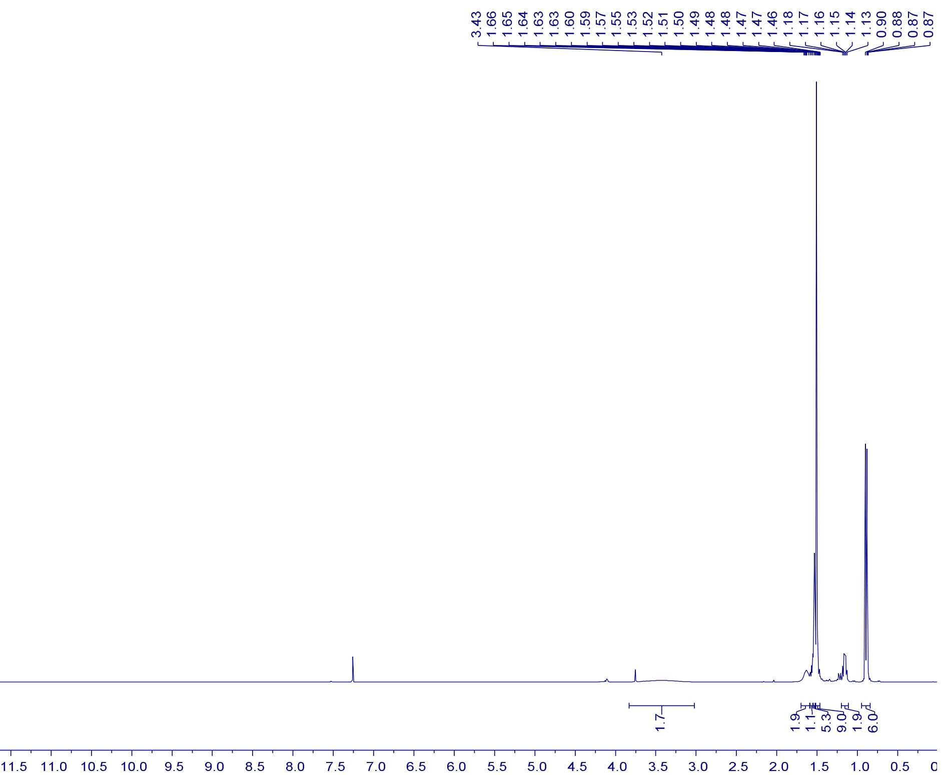

1n ^{13}C NMR (126 MHz, $CDCl_3$)

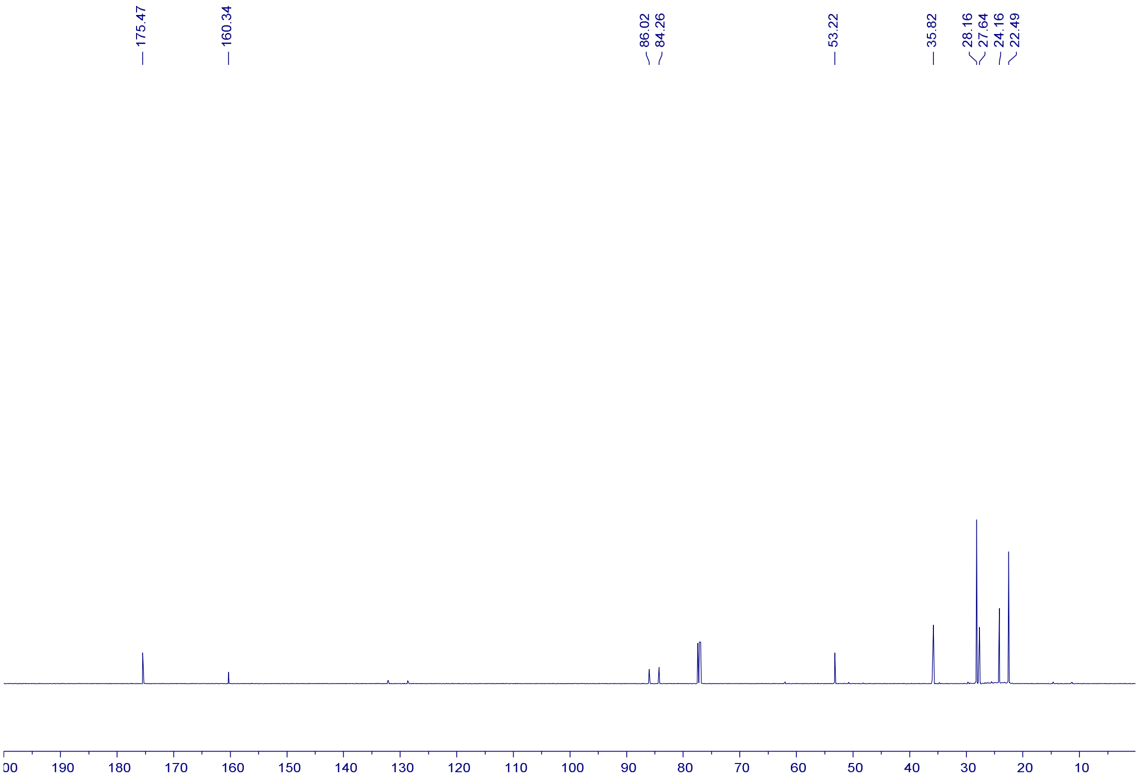

1o ^{1}H NMR (400 MHz, CDCl$_3$)

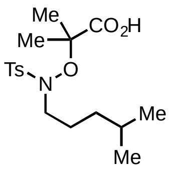

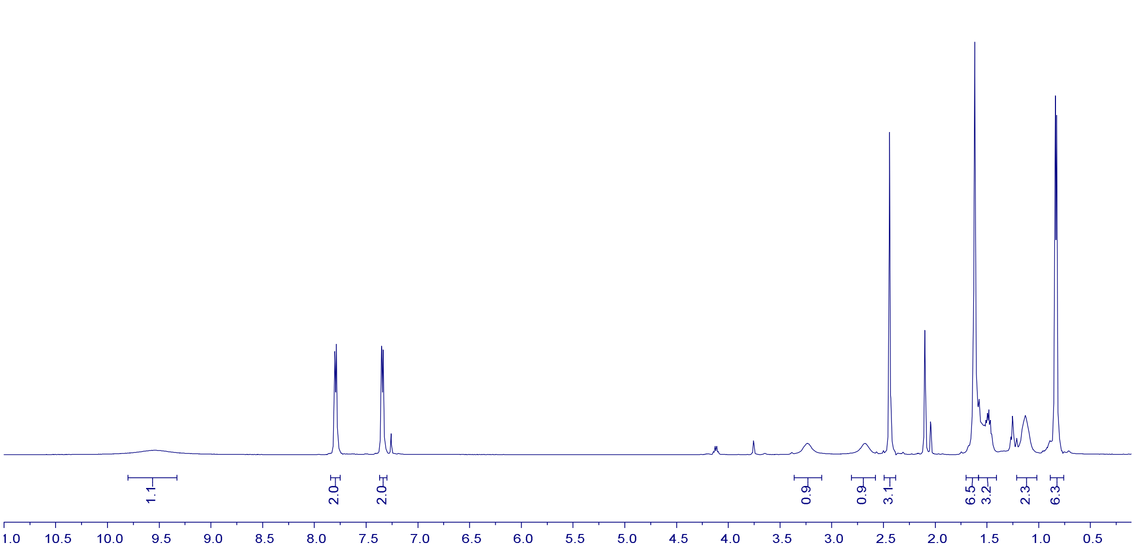

1o ^{13}C NMR (126 MHz, CDCl$_3$)

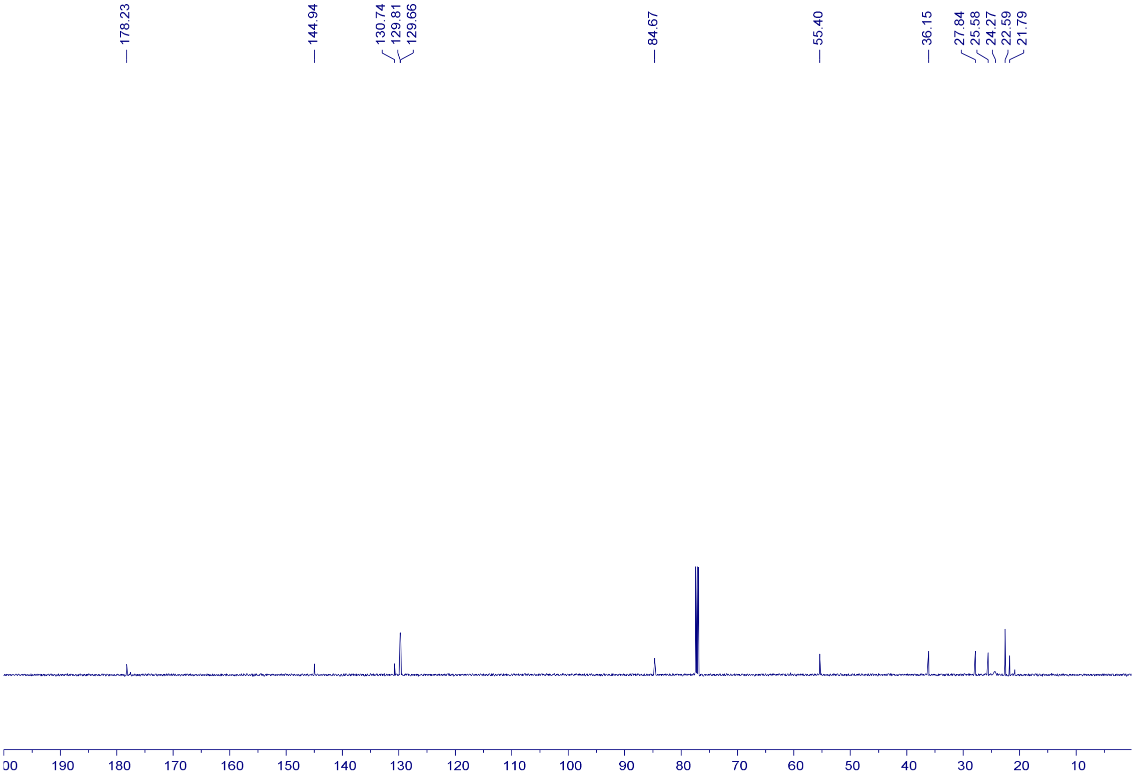

1p ^{1}H NMR (400 MHz, $CDCl_3$)

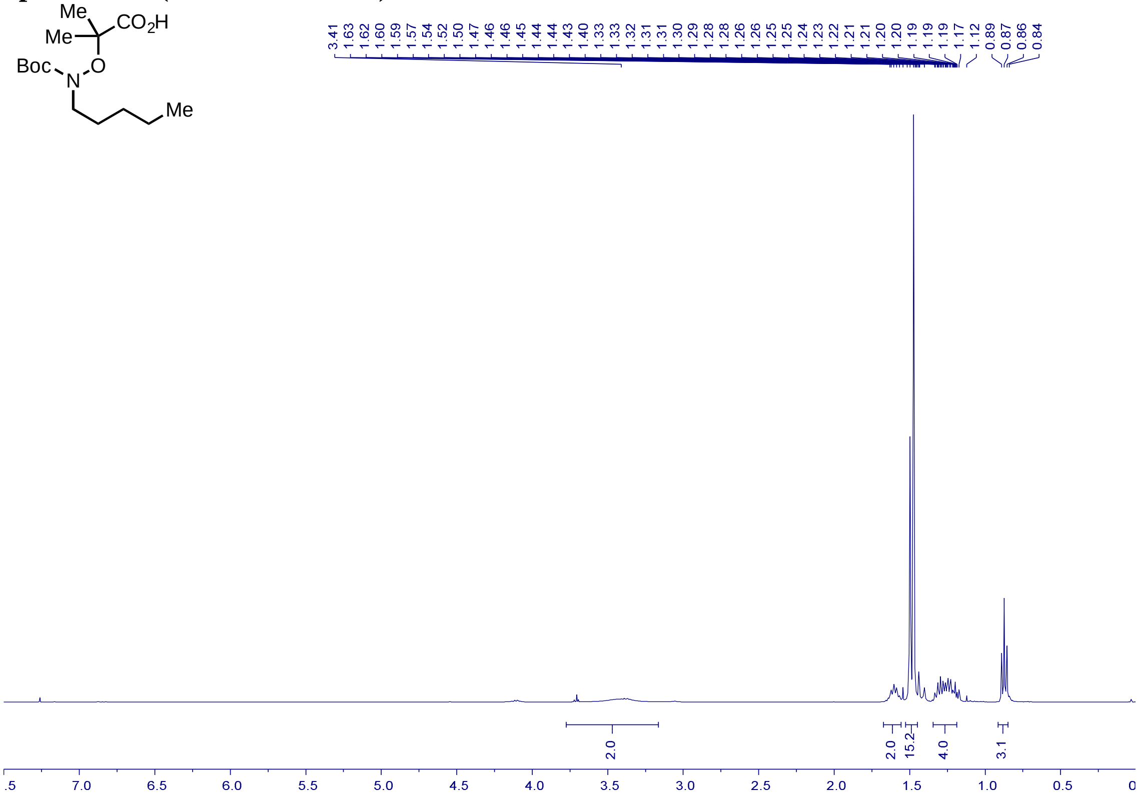

1p ^{13}C NMR (126 MHz, $CDCl_3$)

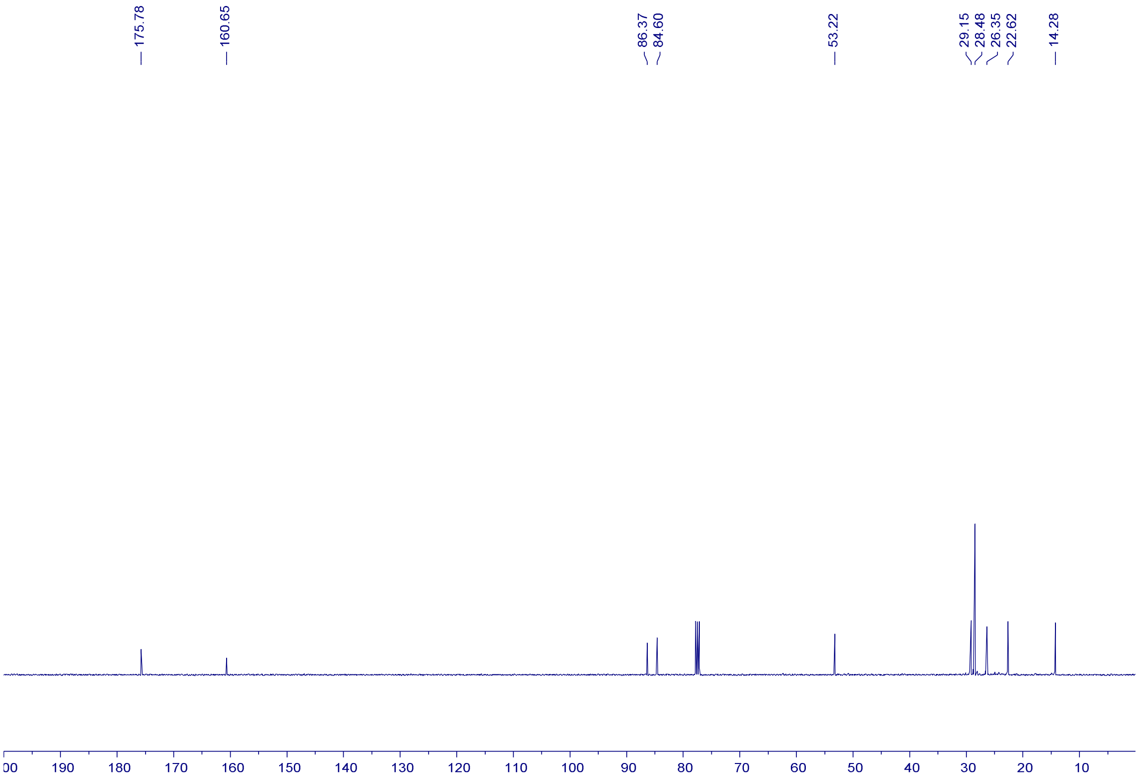

1q ^{1}H NMR (400 MHz, $CDCl_3$)

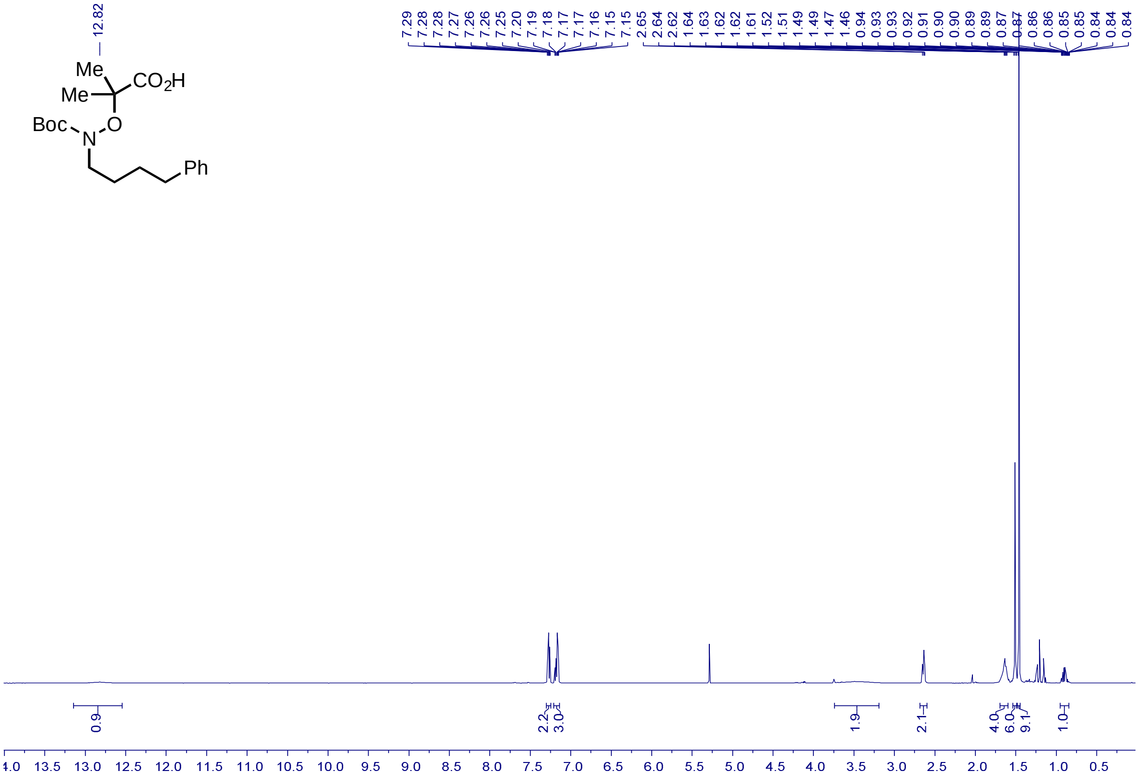

1q ^{13}C NMR (126 MHz, $CDCl_3$)

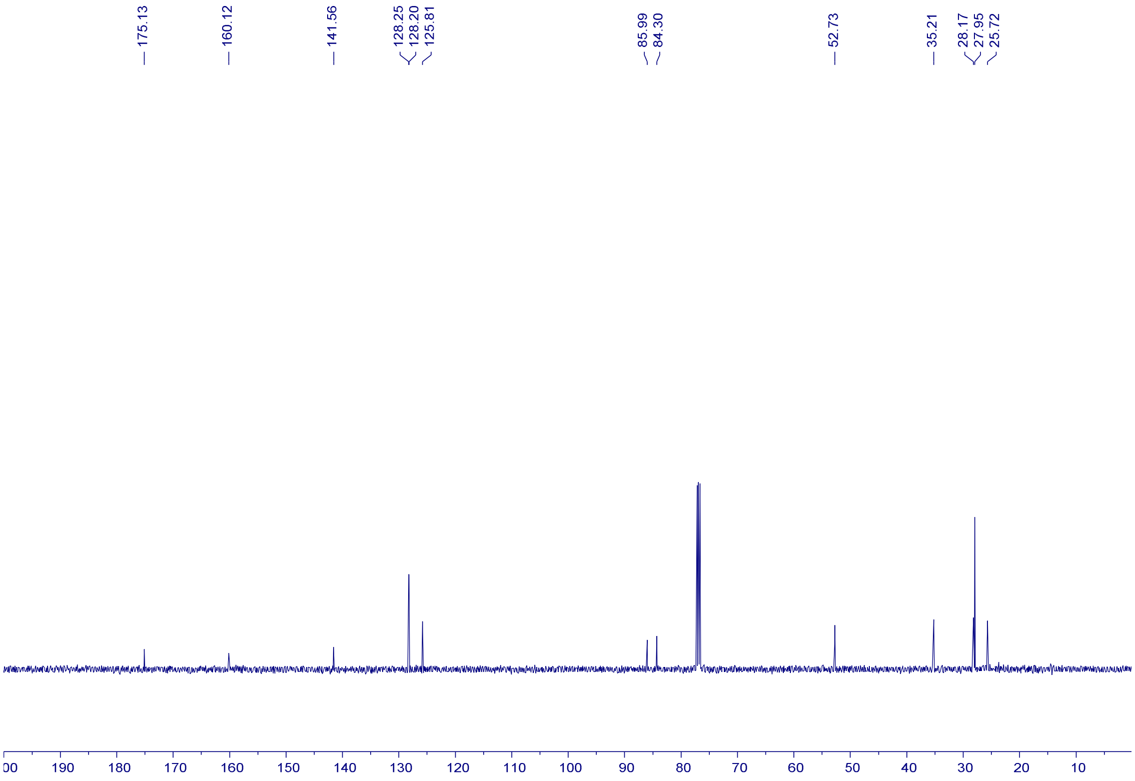

1r ^{1}H NMR (400 MHz, $CDCl_3$)

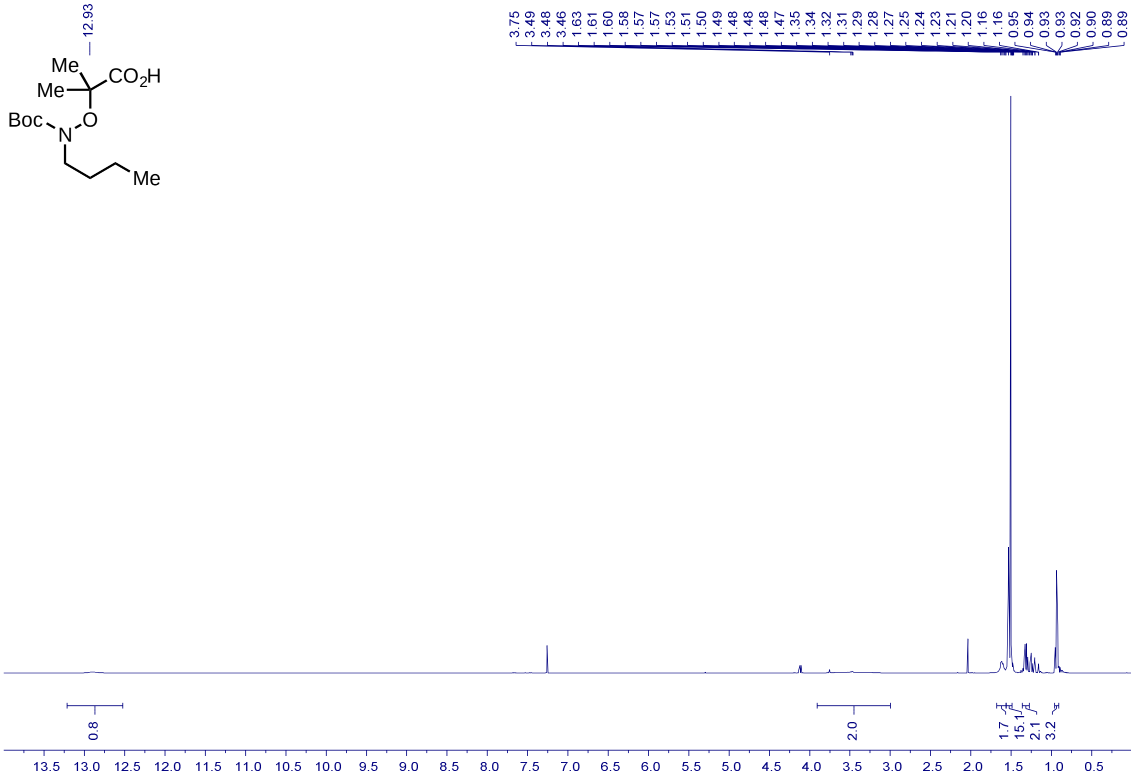

1r ^{13}C NMR (126 MHz, $CDCl_3$)

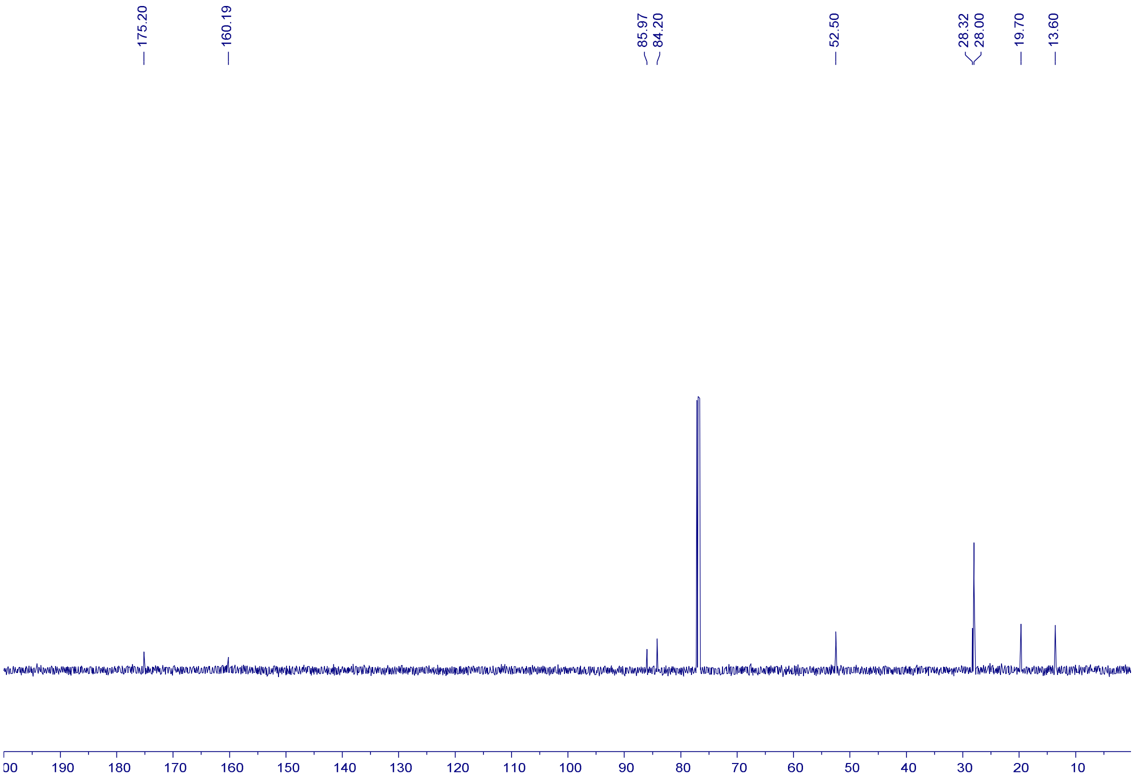

1s ^{1}H NMR (400 MHz, $CDCl_3$)

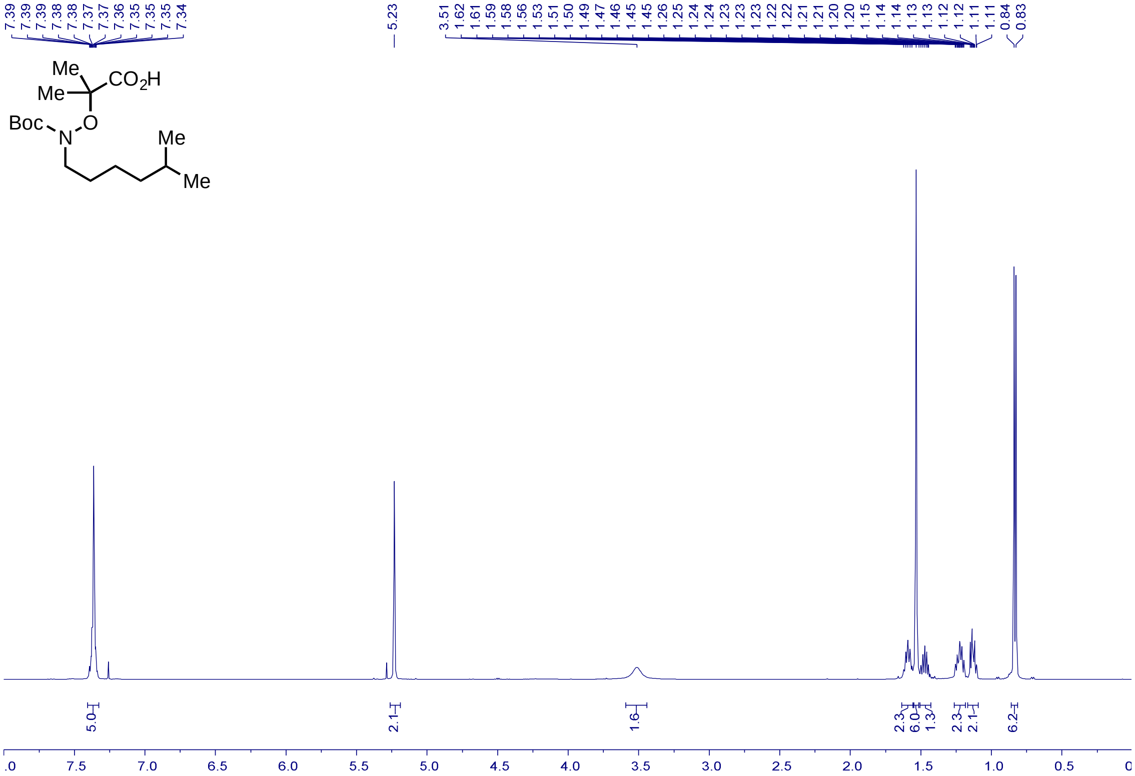

1s ^{13}C NMR (126 MHz, $CDCl_3$)

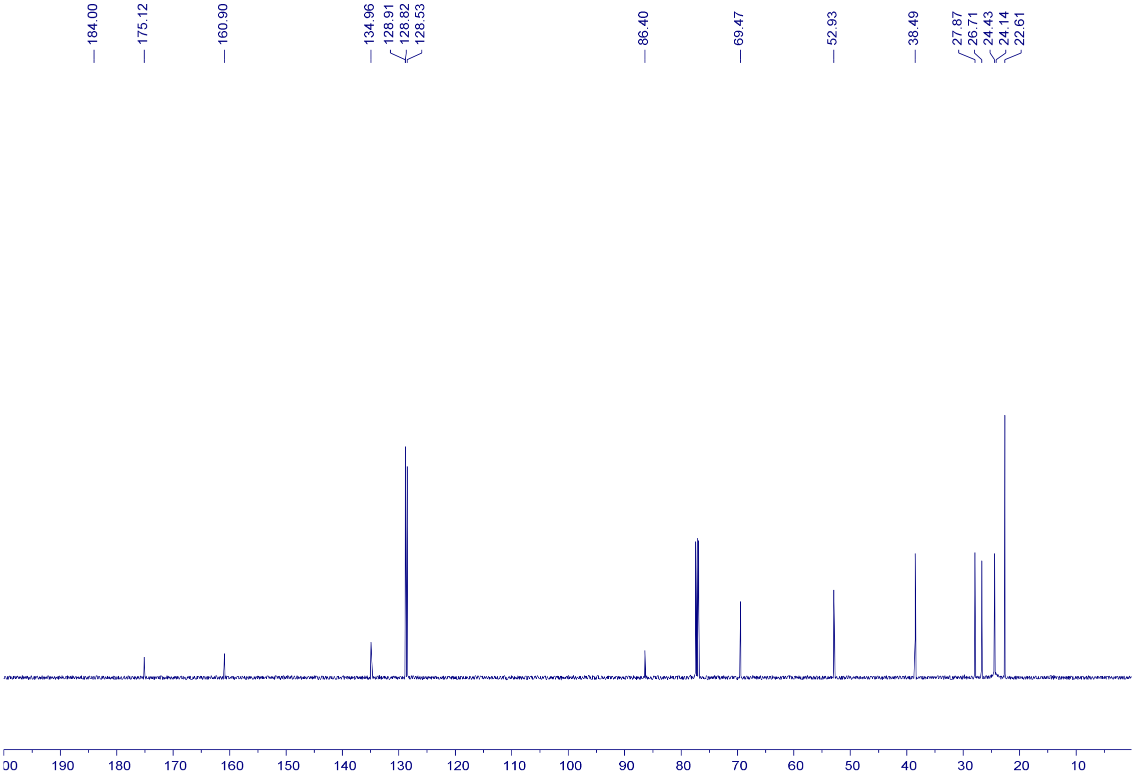

1t ^{1}H NMR (400 MHz, $CDCl_3$)

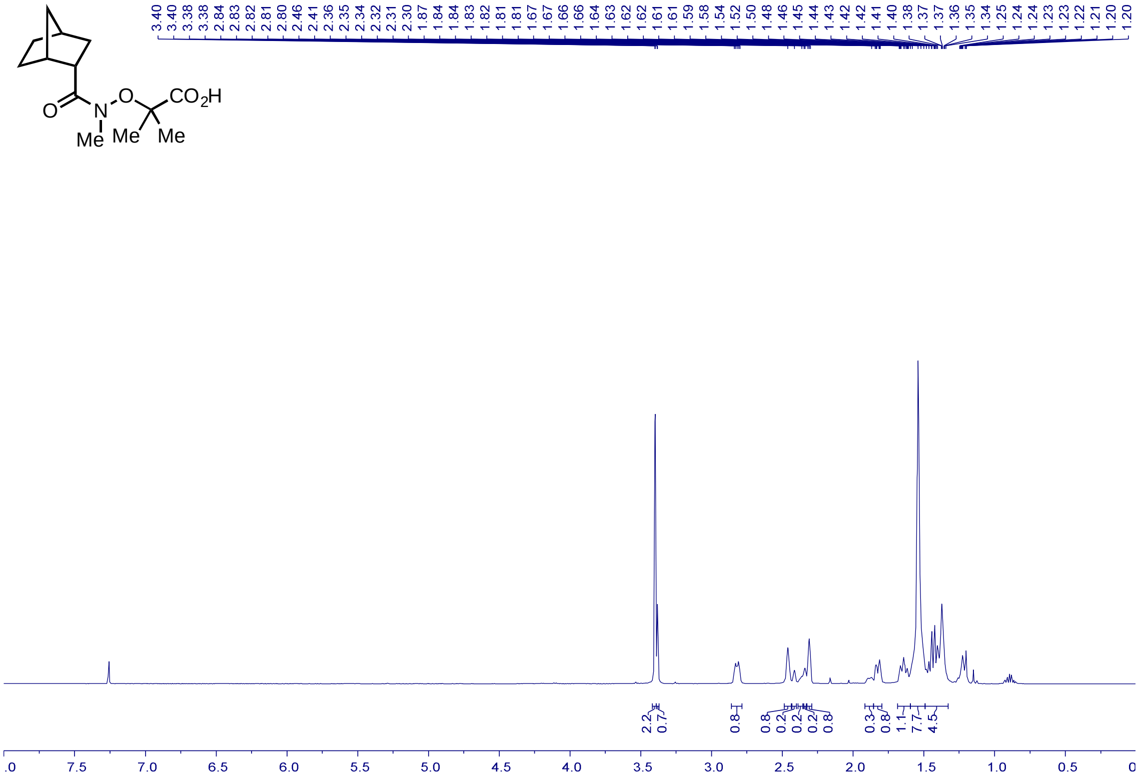

1t ^{13}C NMR (126 MHz, $CDCl_3$)

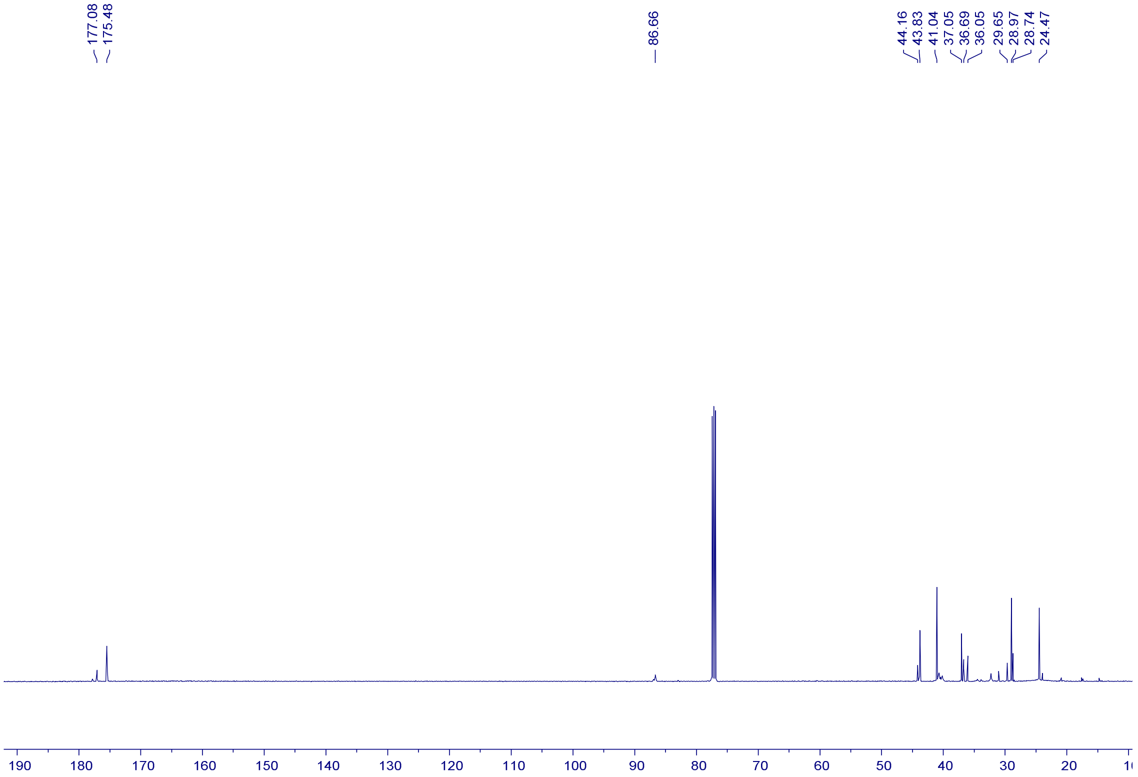

1u ^{1}H NMR (500 MHz, $CDCl_3$)

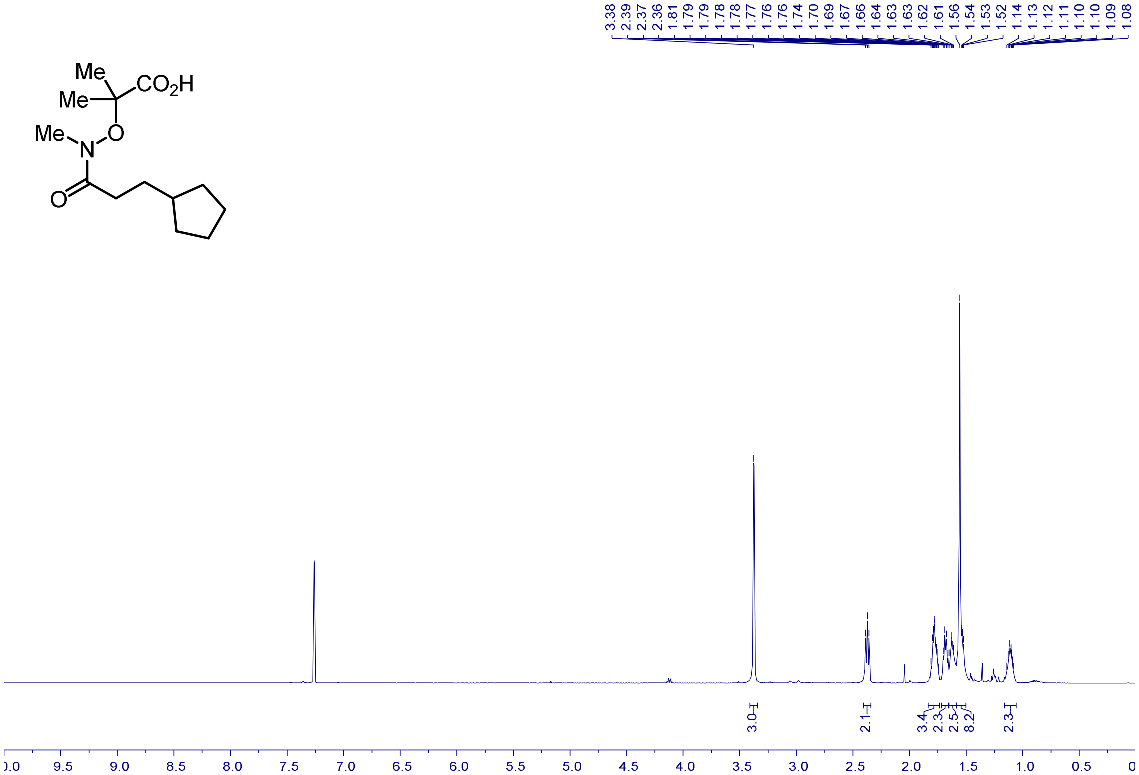

1u ^{13}C NMR (126 MHz, $CDCl_3$)

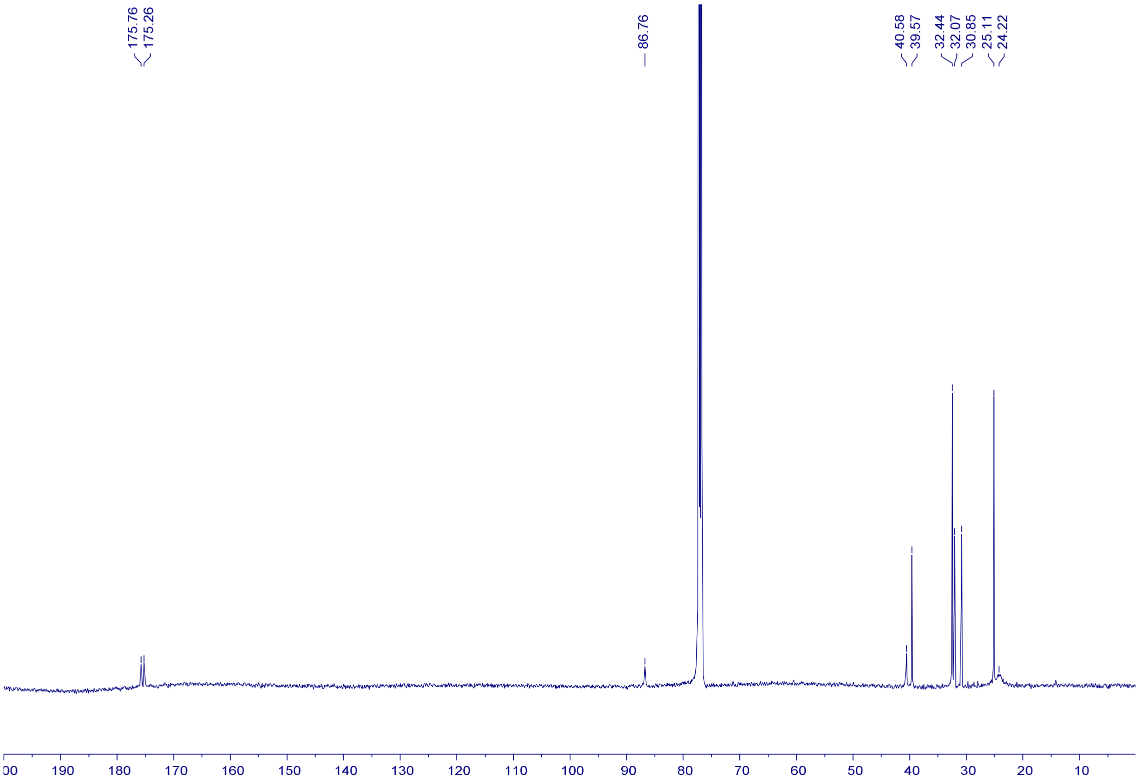

1v ^{1}H NMR (400 MHz, $CDCl_3$)

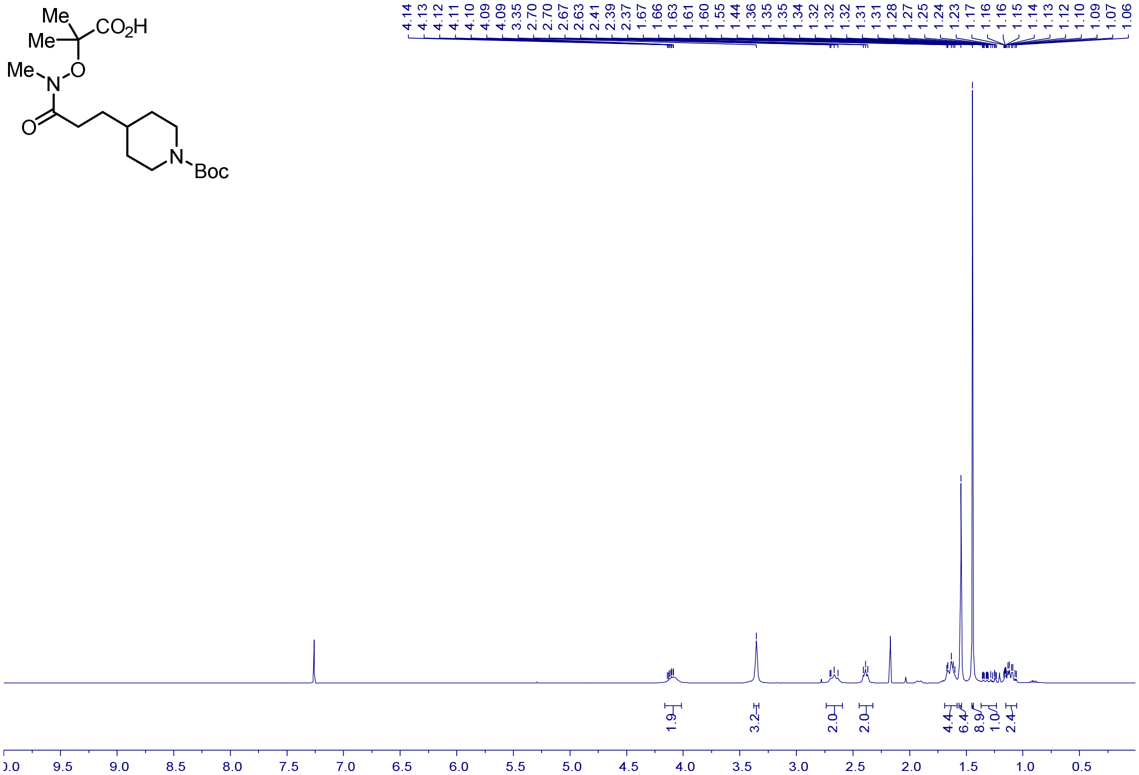

1v ^{13}C NMR (126 MHz, $CDCl_3$)

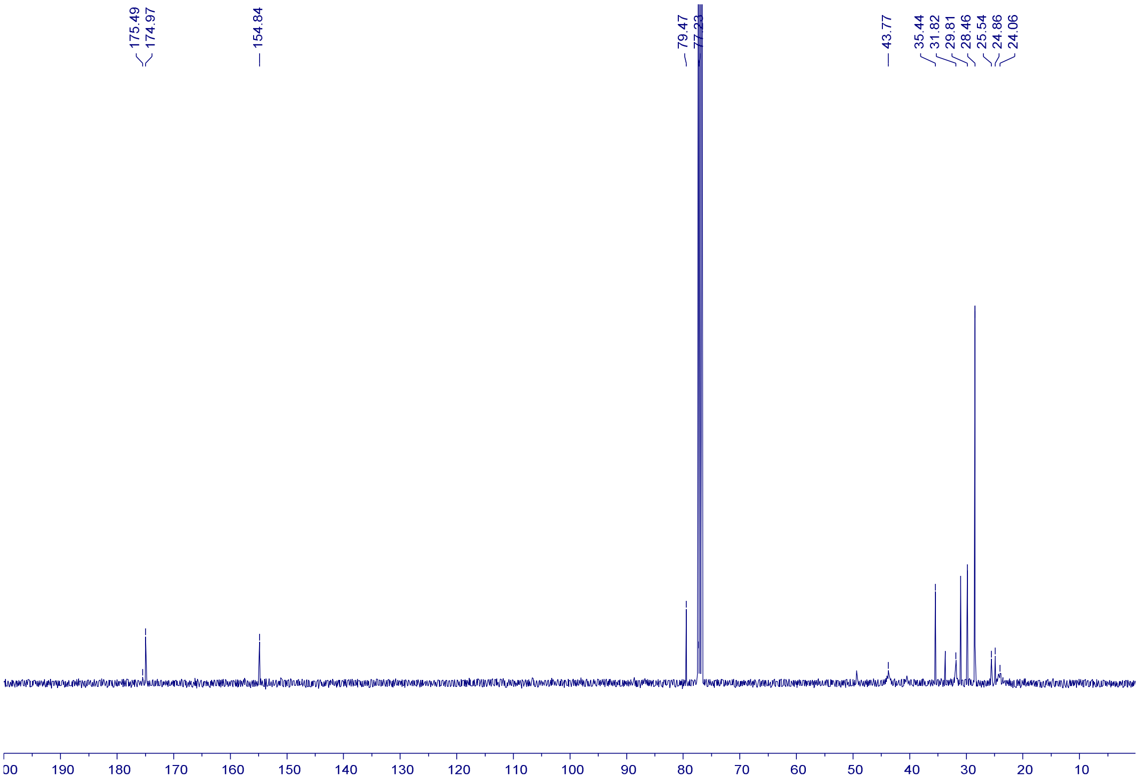

1w ^{1}H NMR (400 MHz, $CDCl_3$)

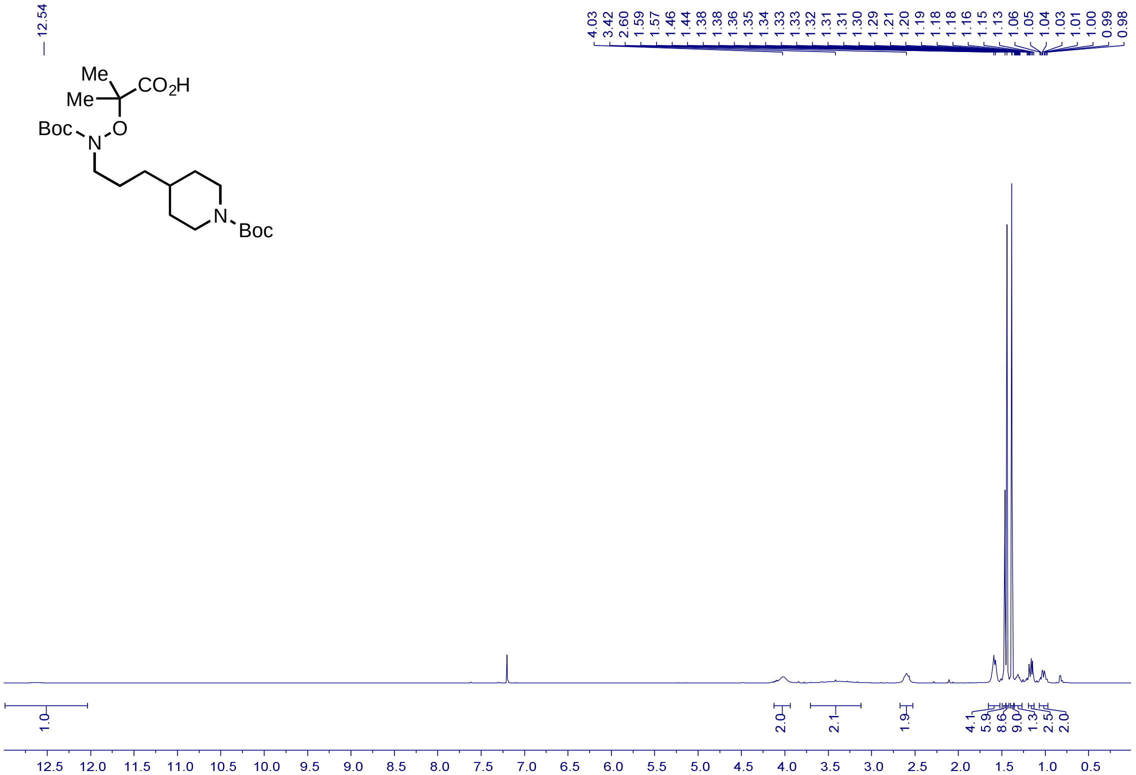

1w ^{13}C NMR (126 MHz, $CDCl_3$)

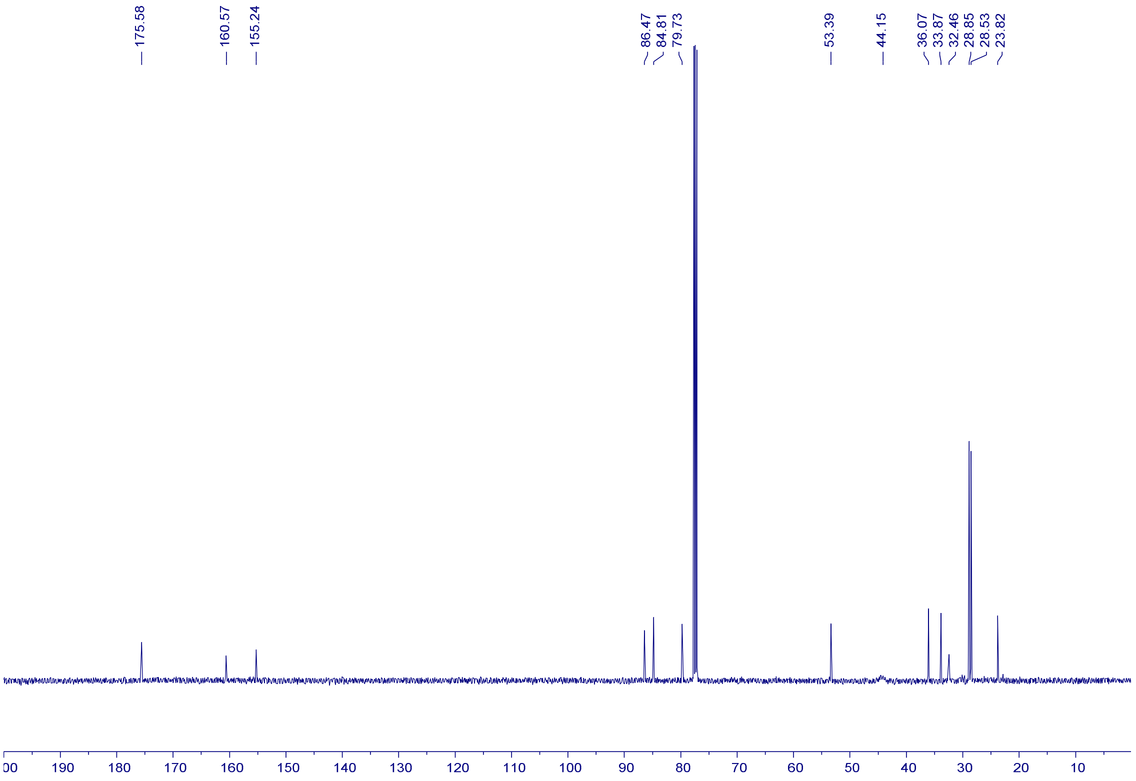

1x ^{1}H NMR (400 MHz, $CDCl_3$)

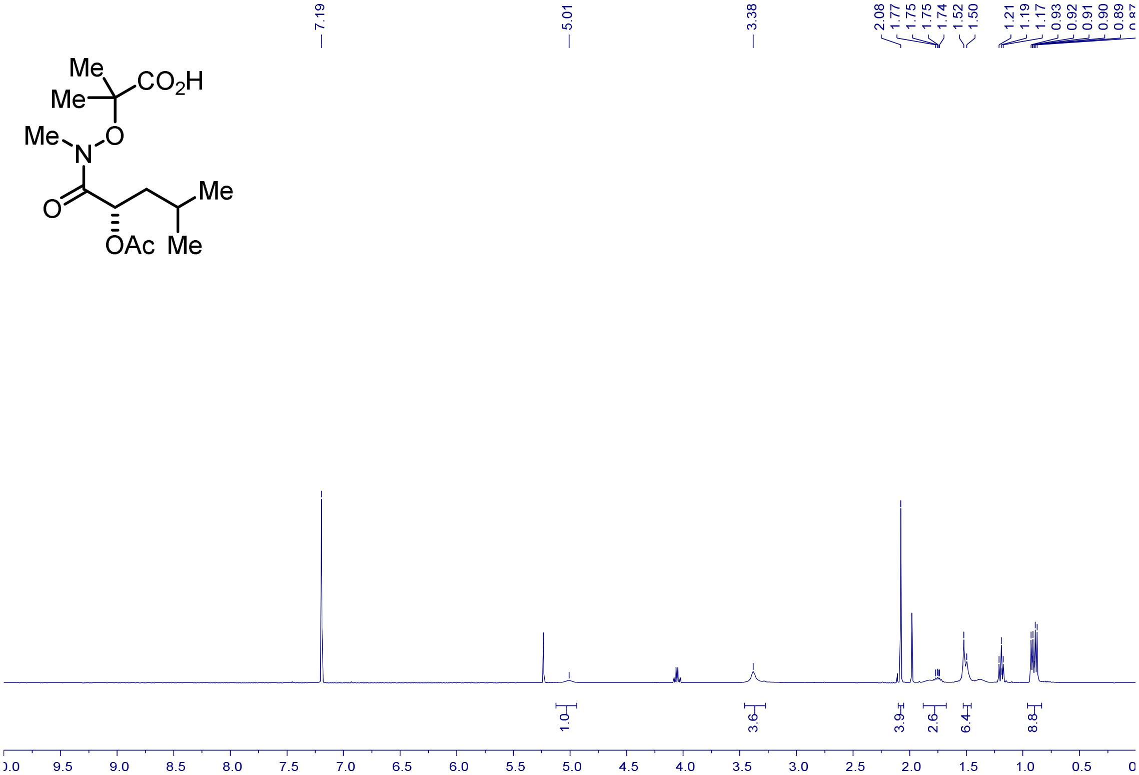

1x ^{13}C NMR (126 MHz, $CDCl_3$)

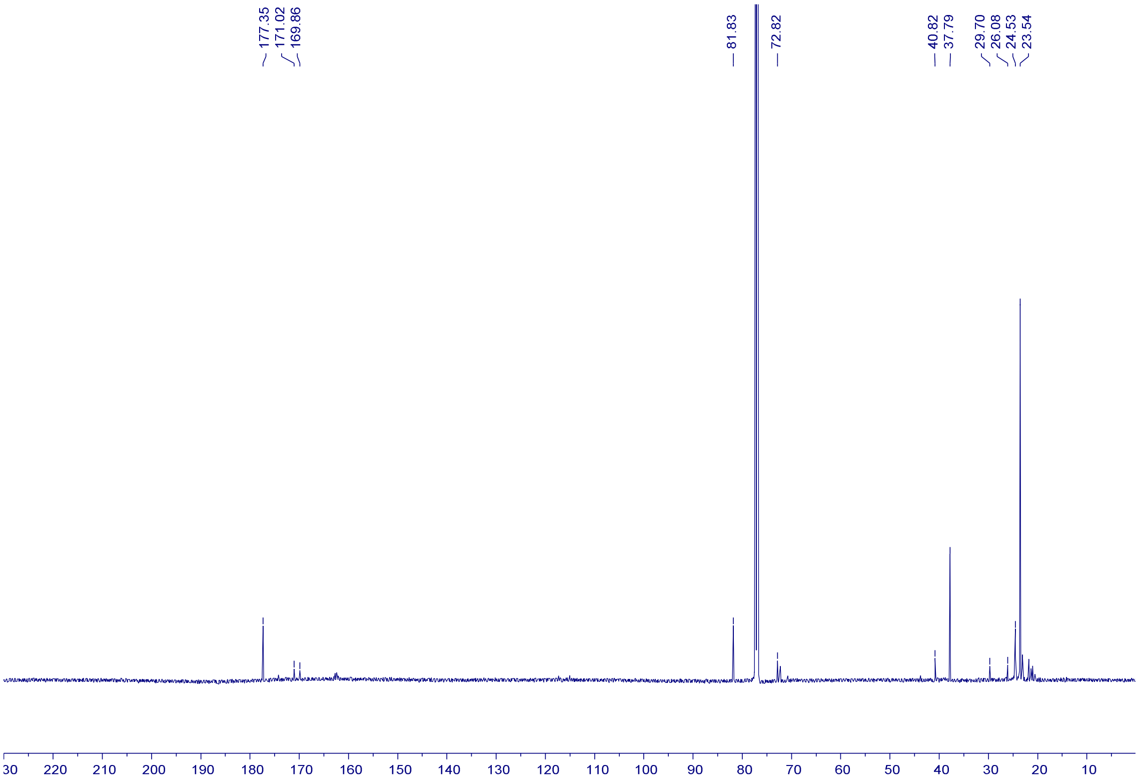

1y ^{1}H NMR (400 MHz, $CDCl_3$)

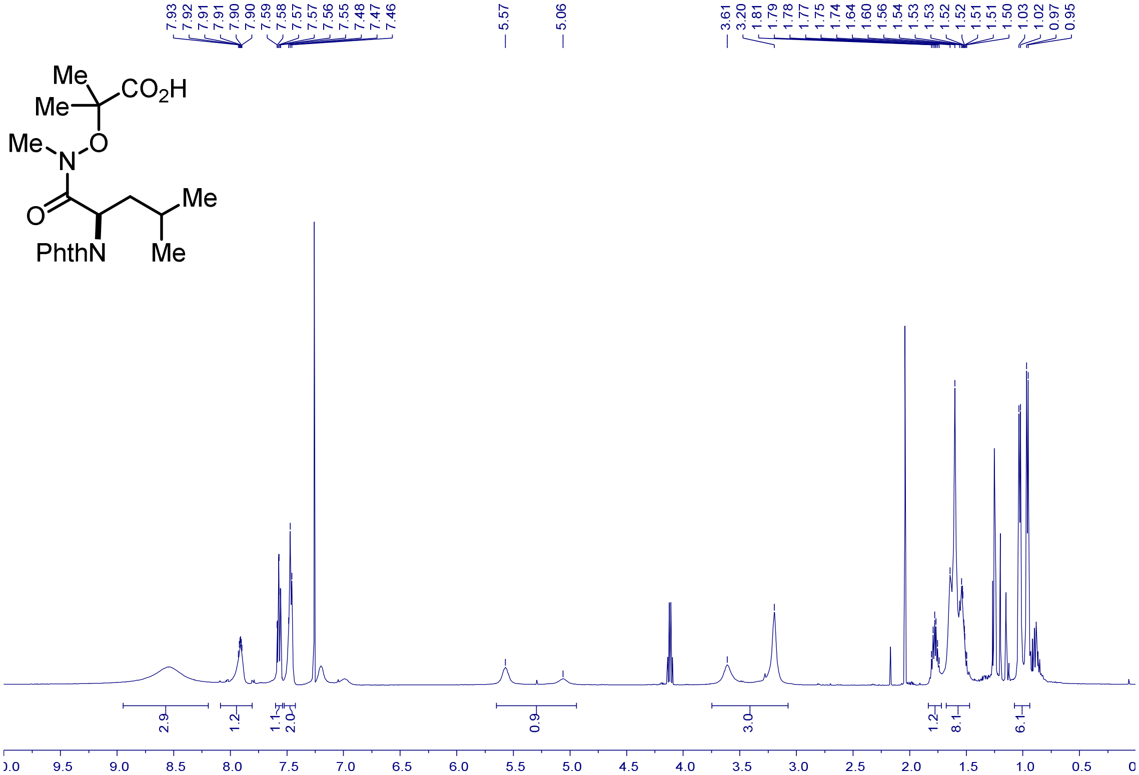

1y ^{13}C NMR (126 MHz, $CDCl_3$)

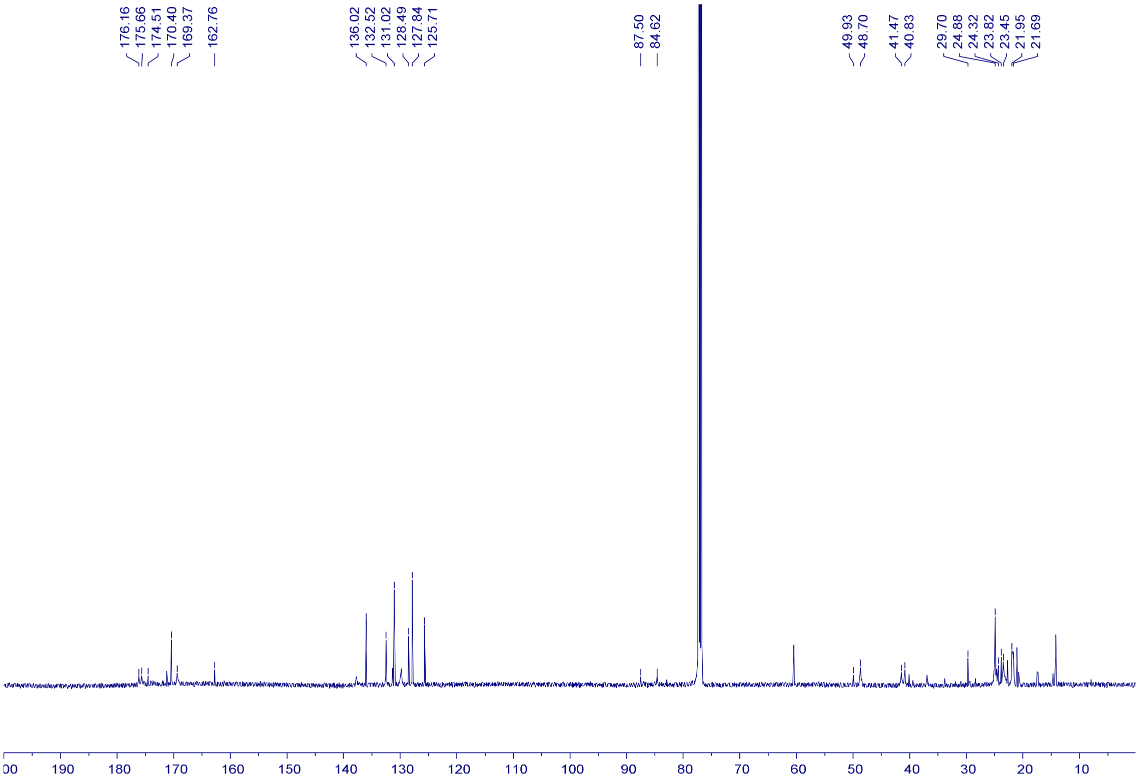

1z ^{1}H NMR (400 MHz, $CDCl_3$)

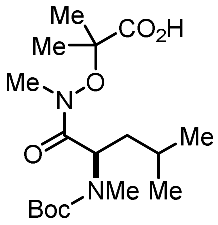

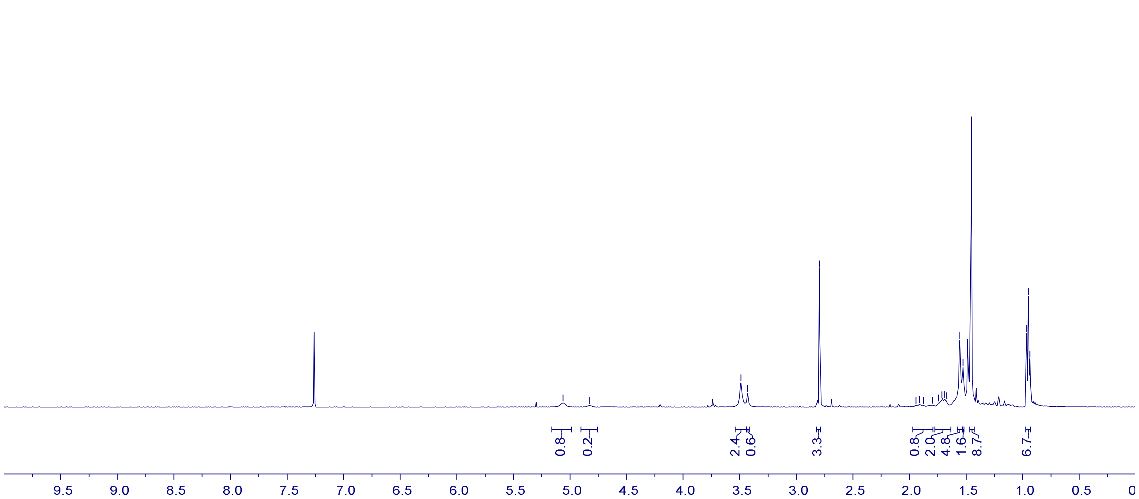

1z ^{13}C NMR (126 MHz, $CDCl_3$)

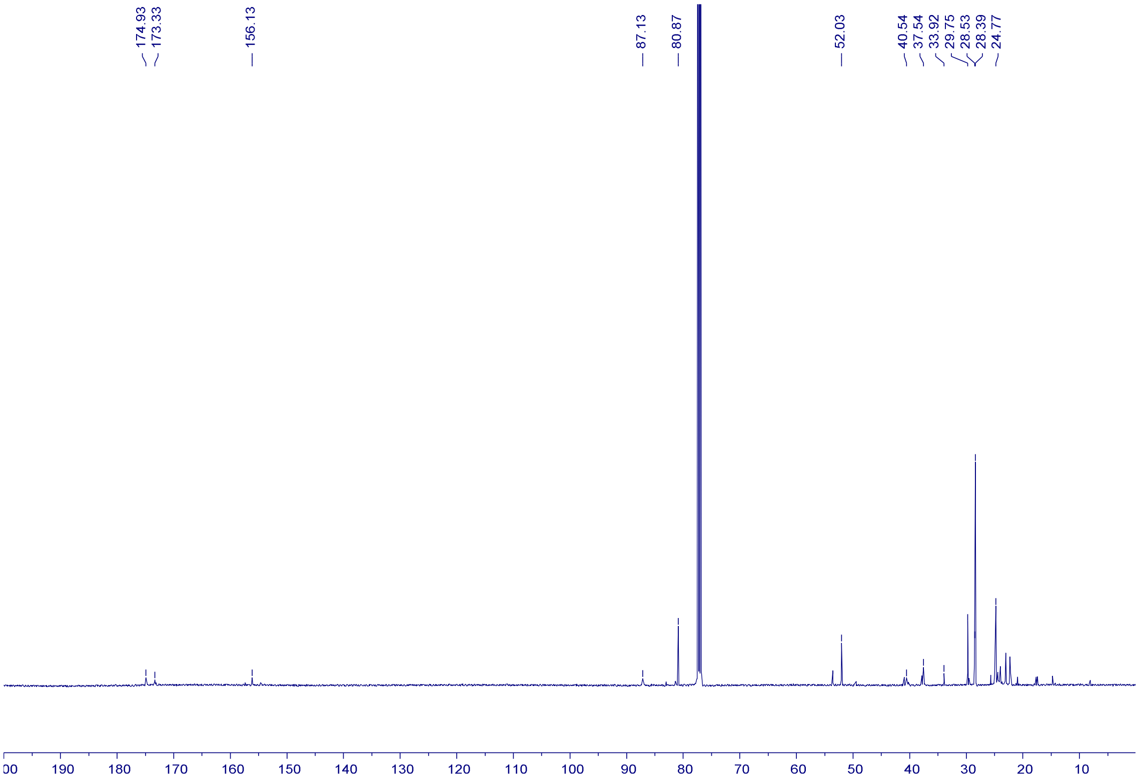

1aa ^{1}H NMR (DMSO-d_6, 500 MHz)

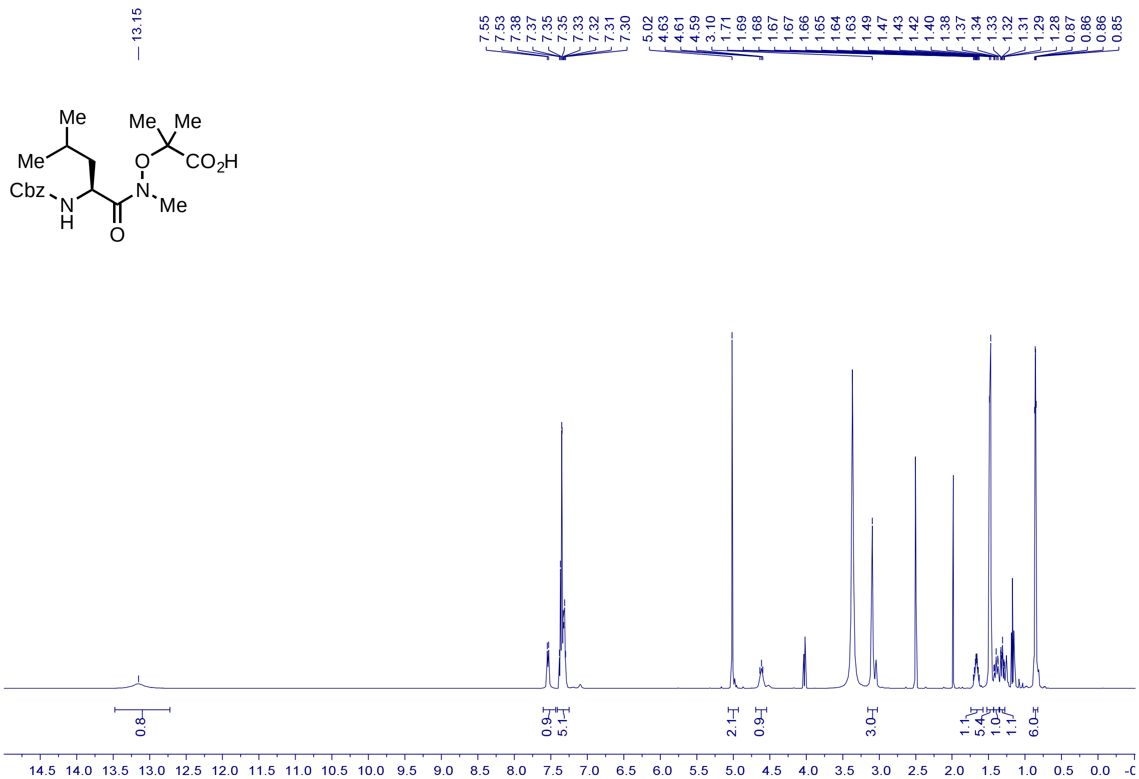

1aa ^{13}C NMR (126 MHz, DMSO-d_6)

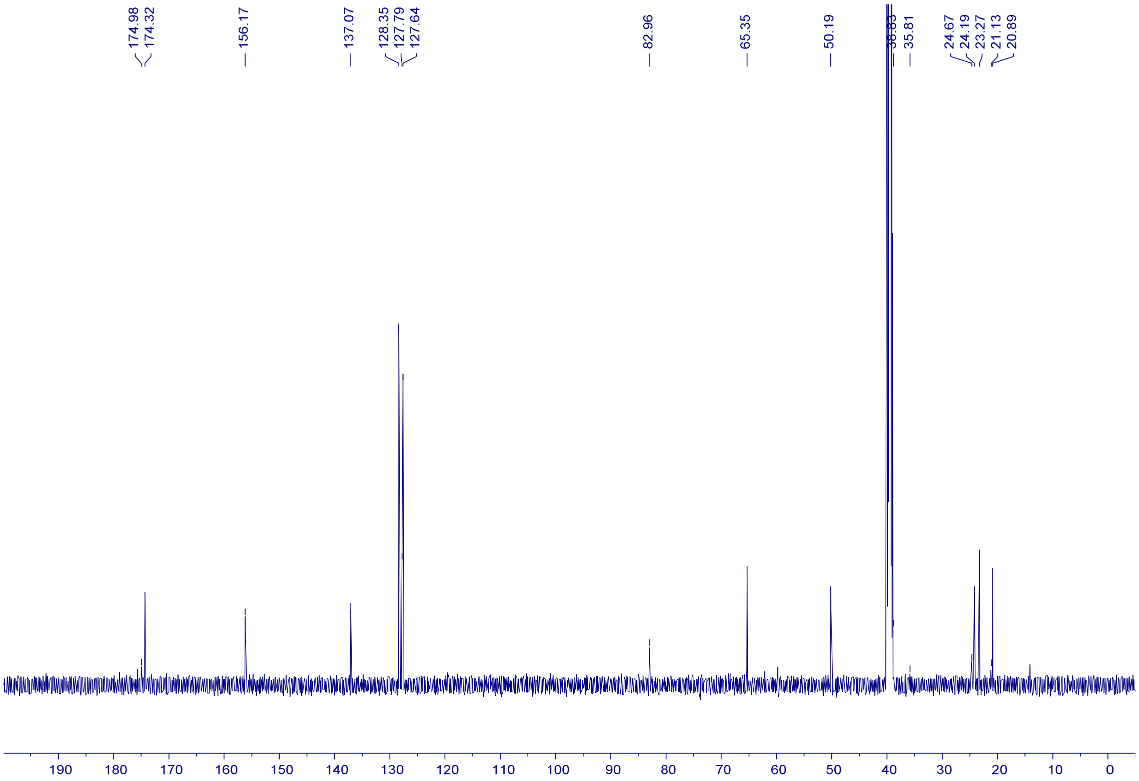

1ac ^{1}H NMR (500 MHz, DMSO-d_6)

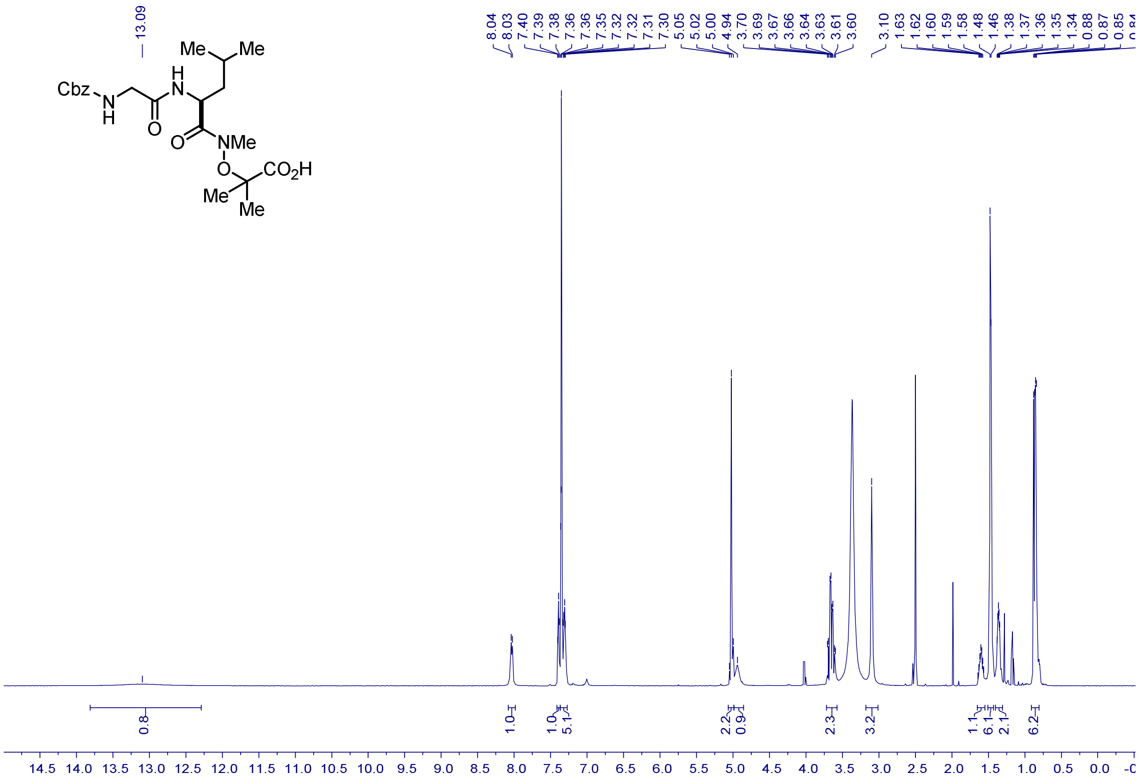

1ac ^{13}C NMR (126 MHz, DMSO-d_6)

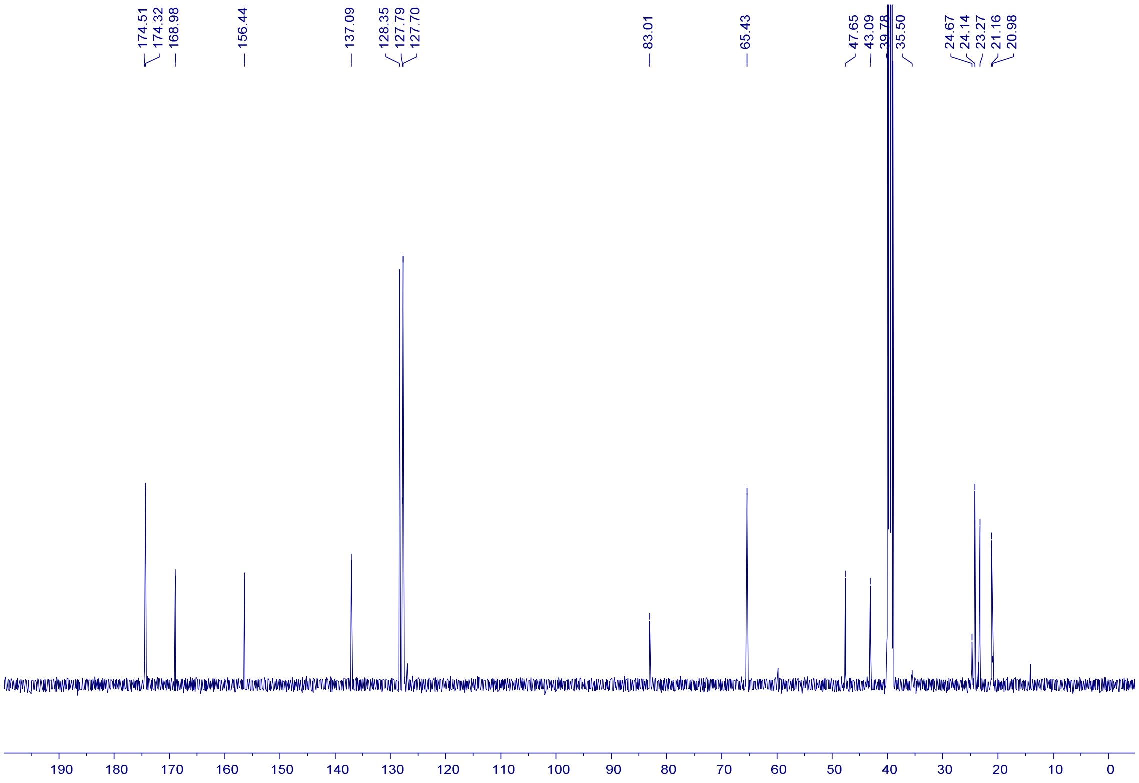

1ad ^{1}H NMR (400 MHz, DMSO-d_6)

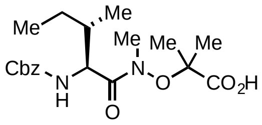

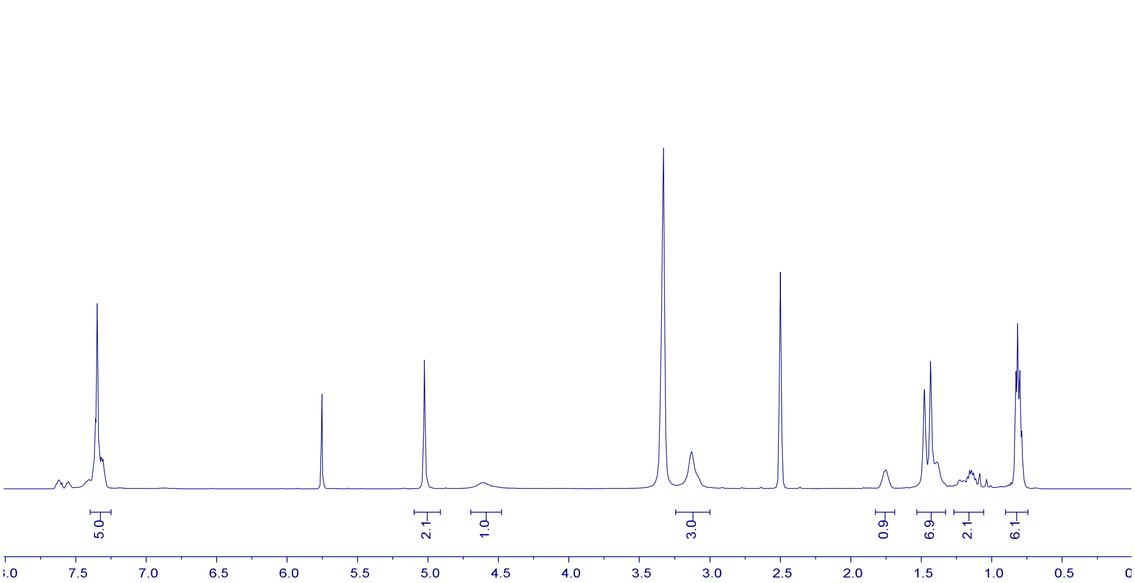

1ad ^{13}C NMR (126 MHz, DMSO-d_6)

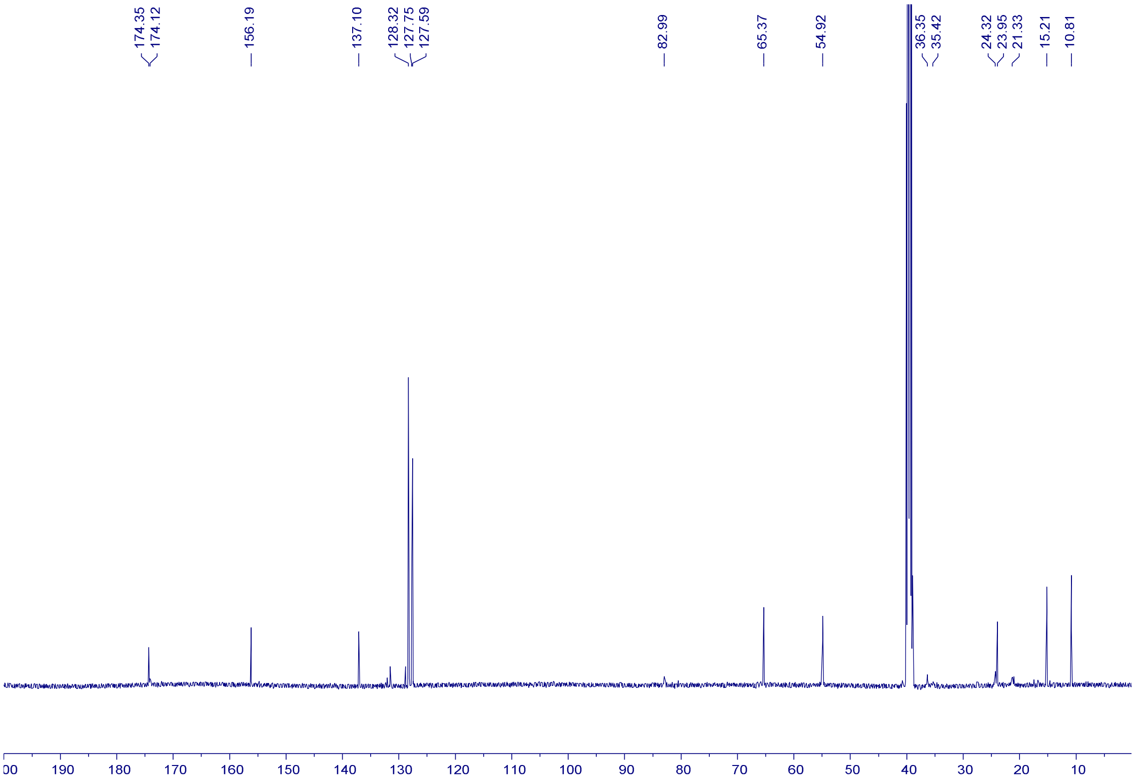

1ae ^{1}H NMR (400 MHz, DMSO-d_6)

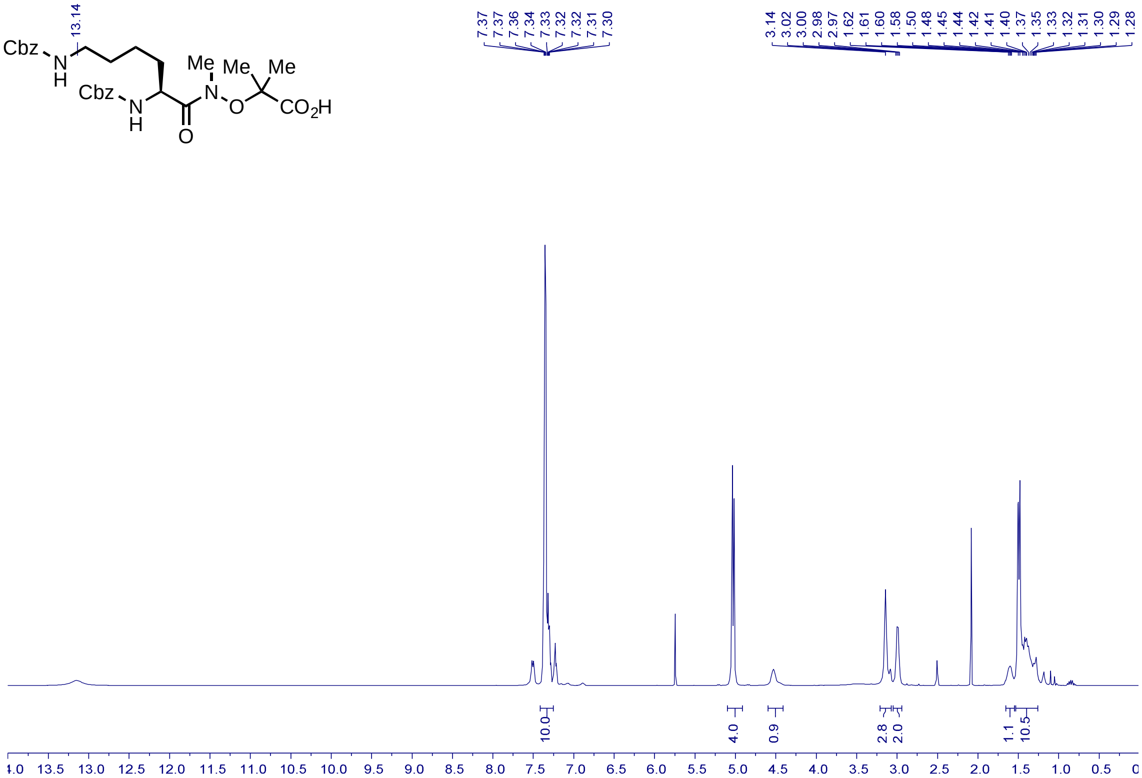

1ae ^{13}C NMR (126 MHz, DMSO-d_6)

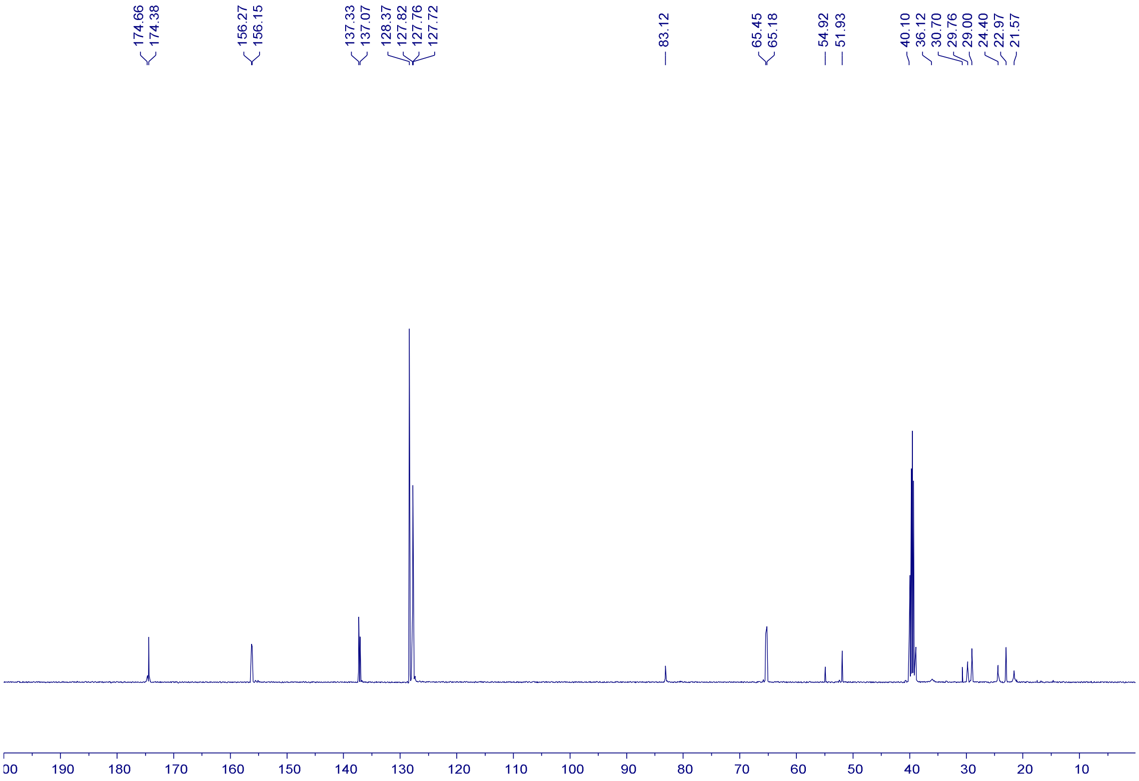

4 ^{1}H NMR (400 MHz, $CDCl_3$)

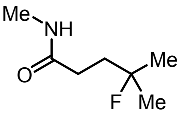

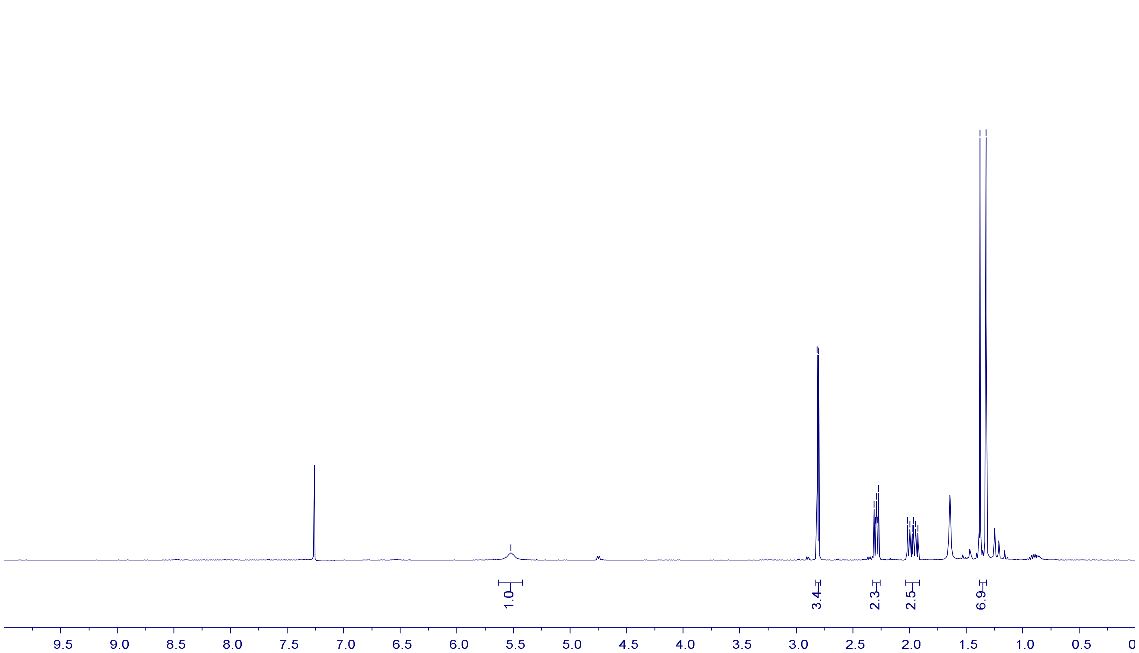

4 ^{13}C NMR (126 MHz, $CDCl_3$)

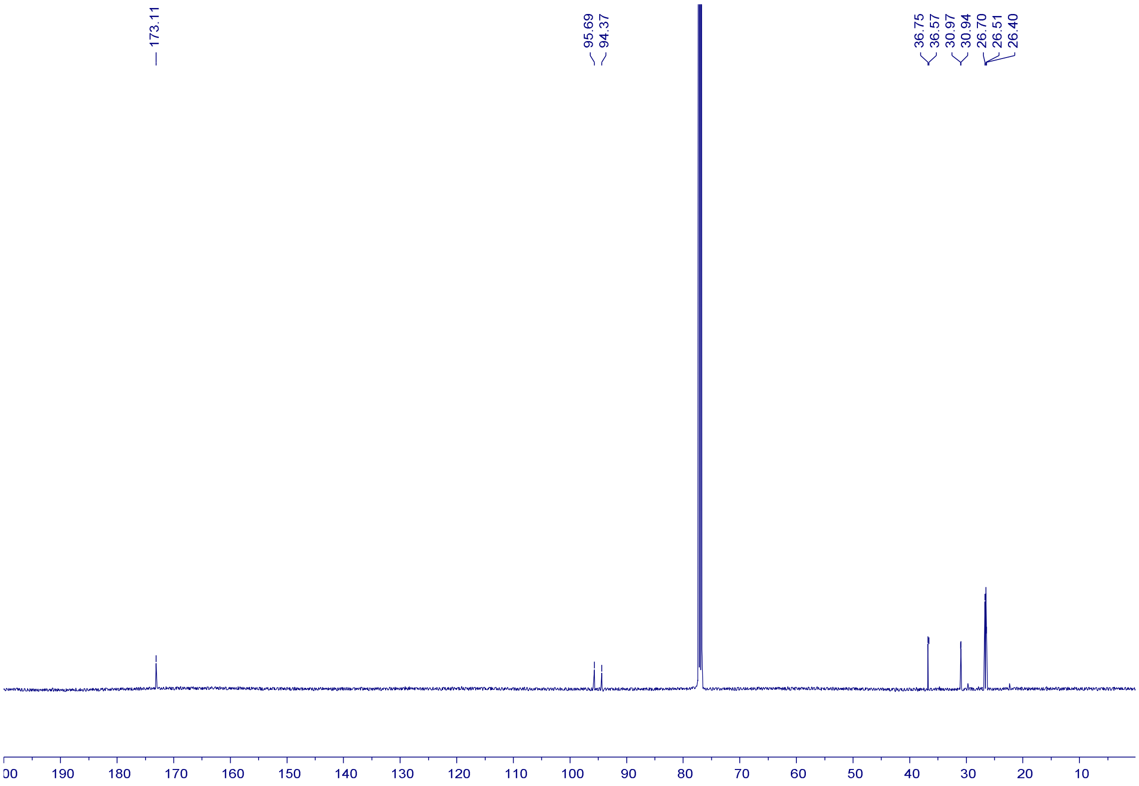

4 ^{19}F NMR (376 MHz, $CDCl_3$)

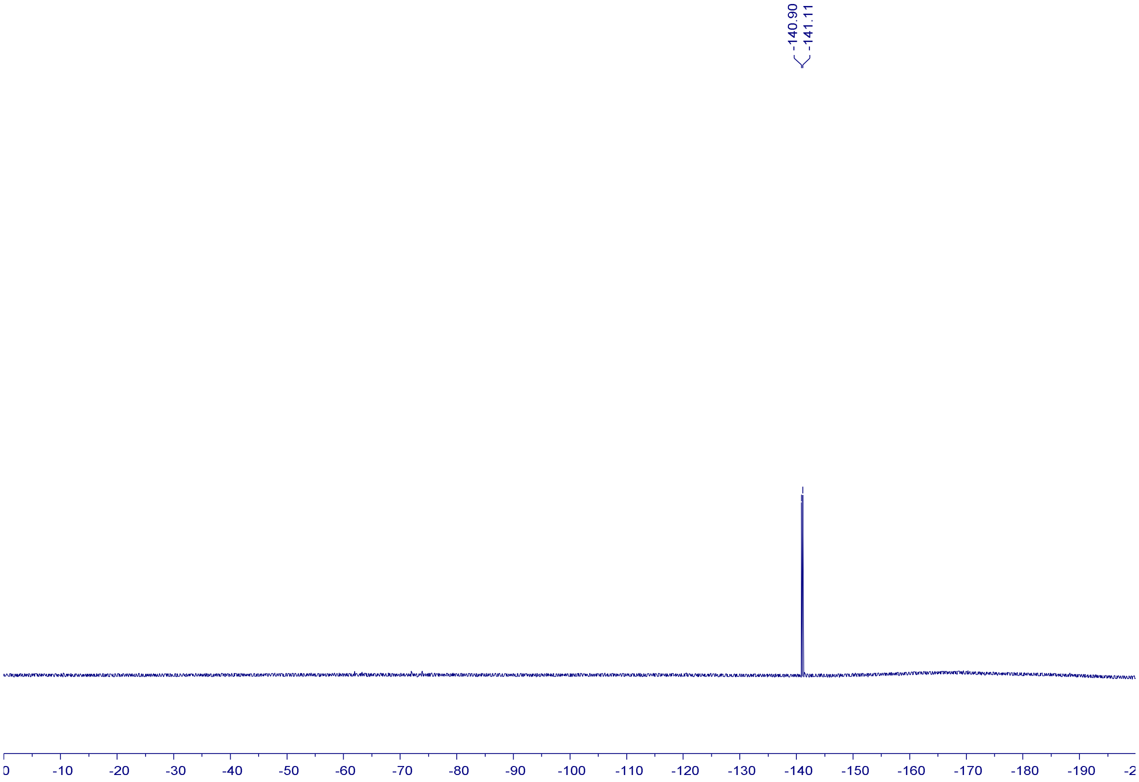

5 ^{1}H NMR (400 MHz, $CDCl_3$)

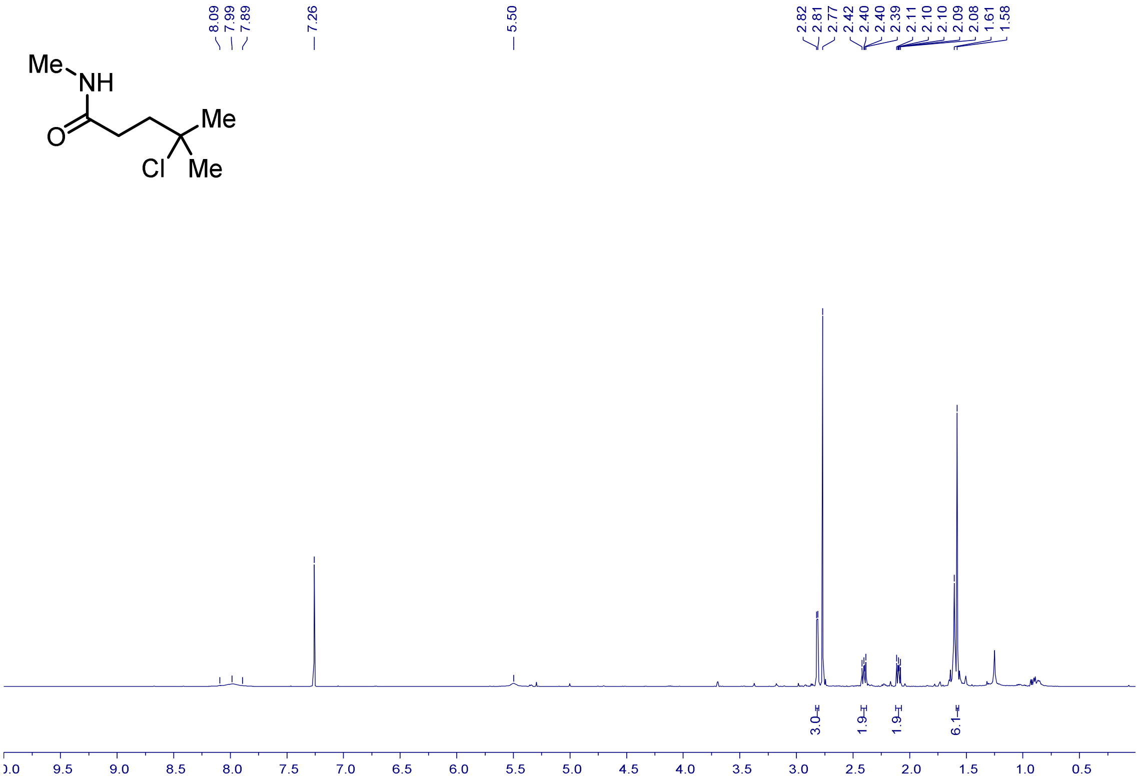

5 ^{13}C NMR (101 MHz, $CDCl_3$)

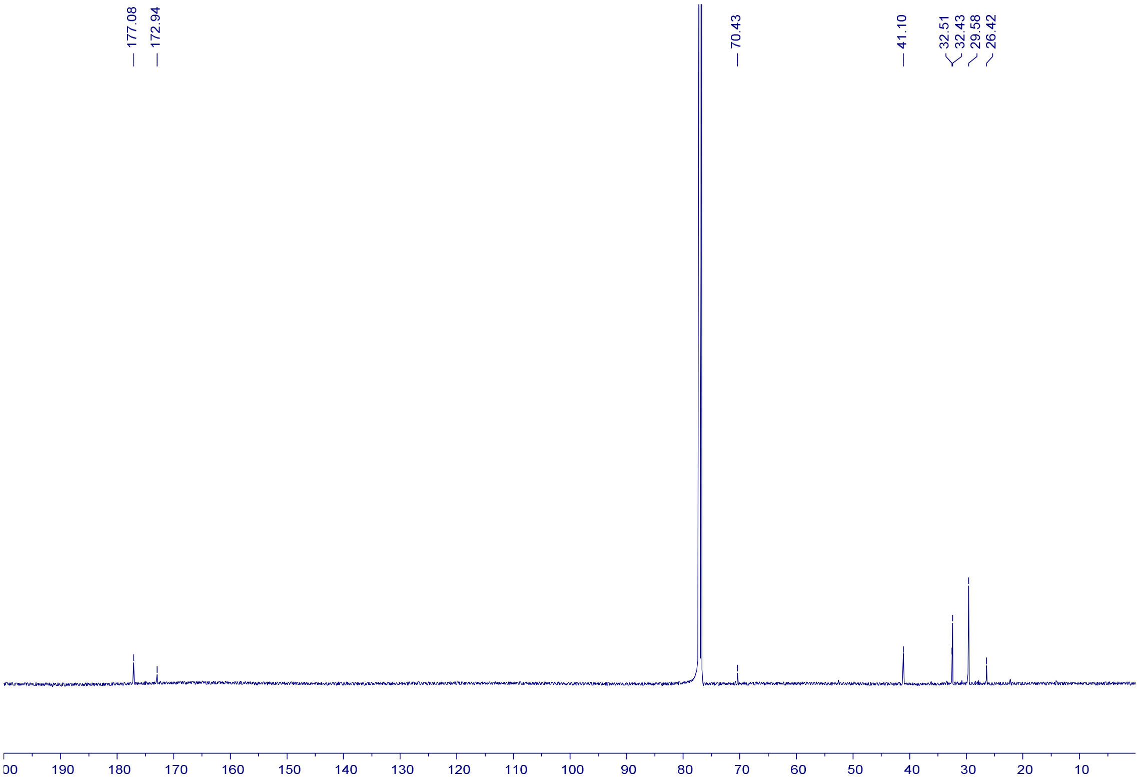

6 ^{1}H NMR (400 MHz, $CDCl_3$)

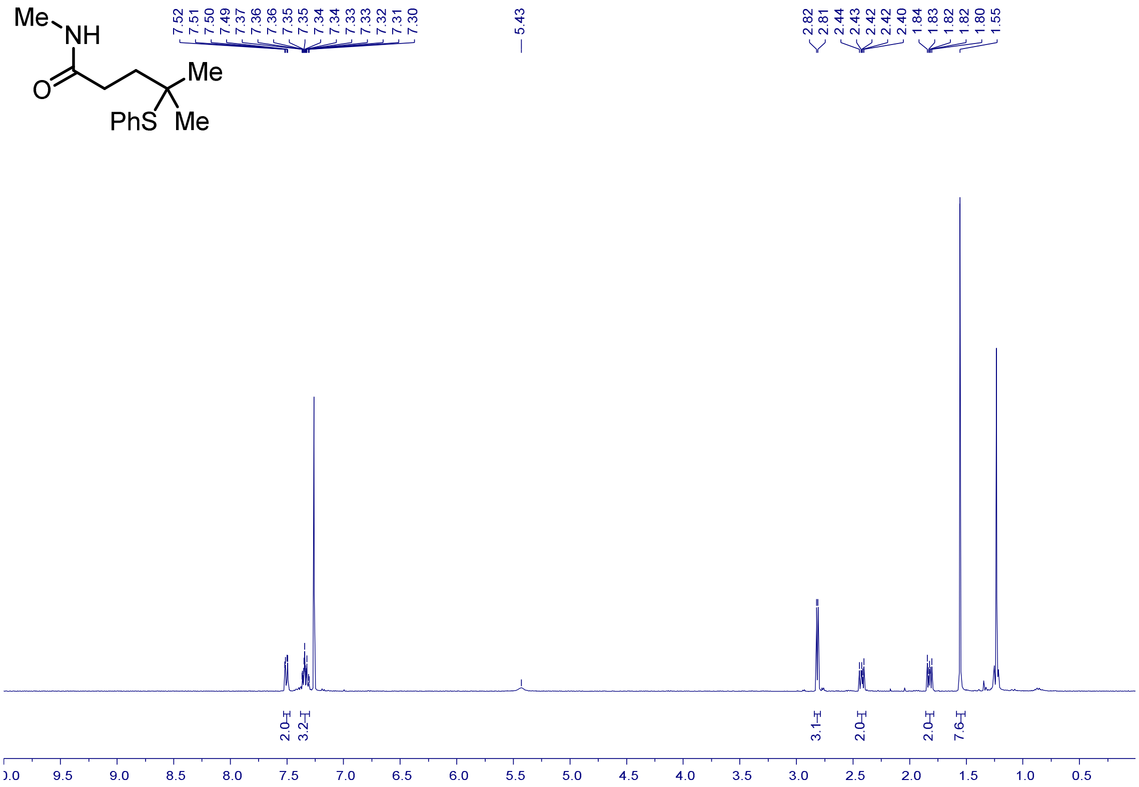

6 ^{13}C NMR (101 MHz, $CDCl_3$)

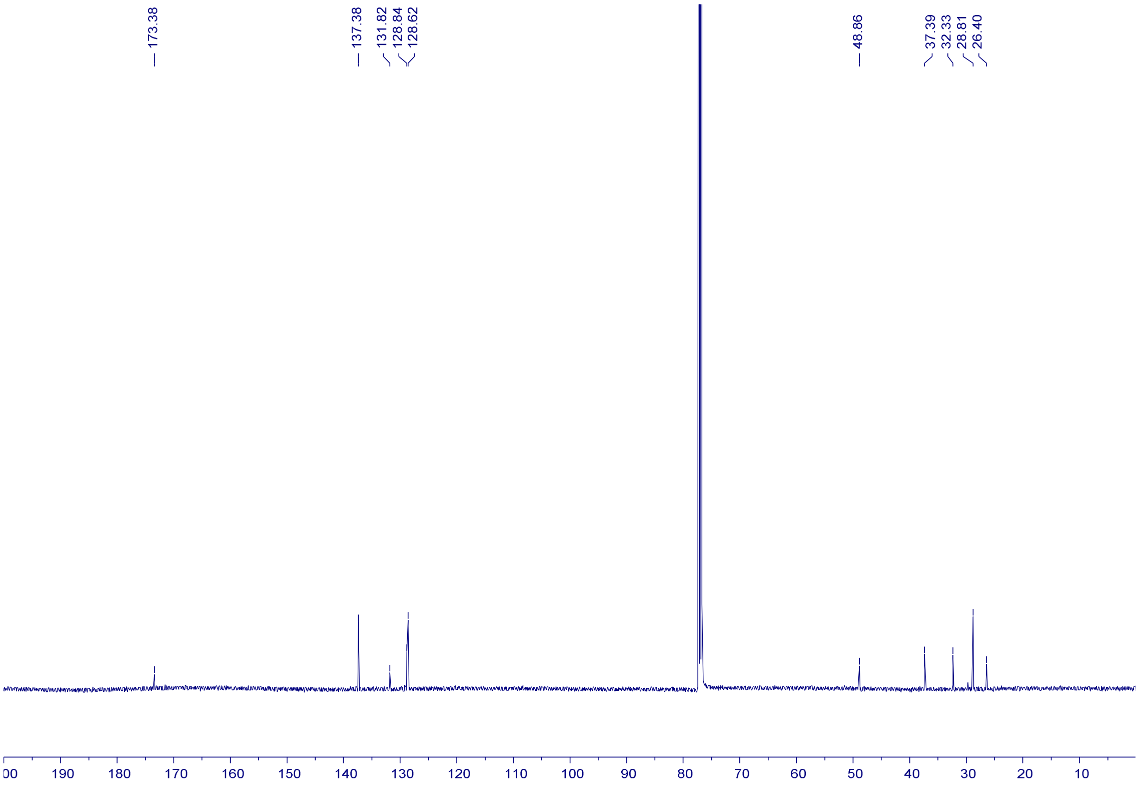

7 ^{1}H NMR (400 MHz, $CDCl_3$)

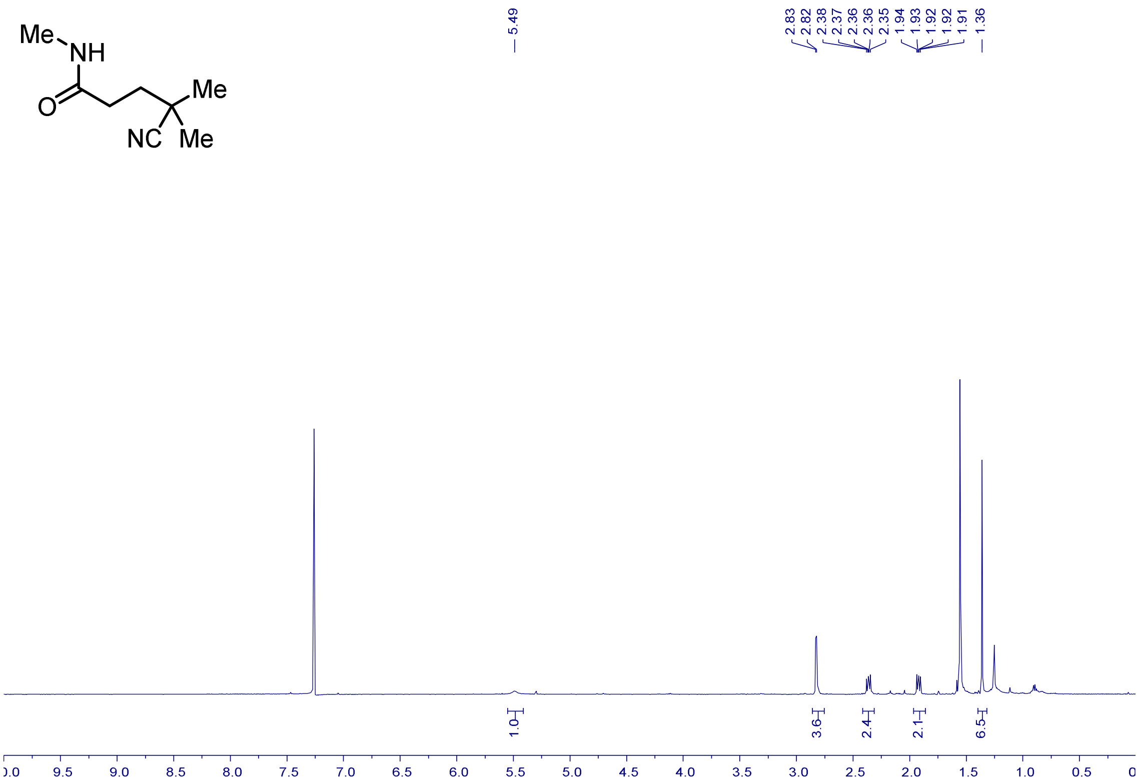

7 ^{13}C NMR (101 MHz, $CDCl_3$)

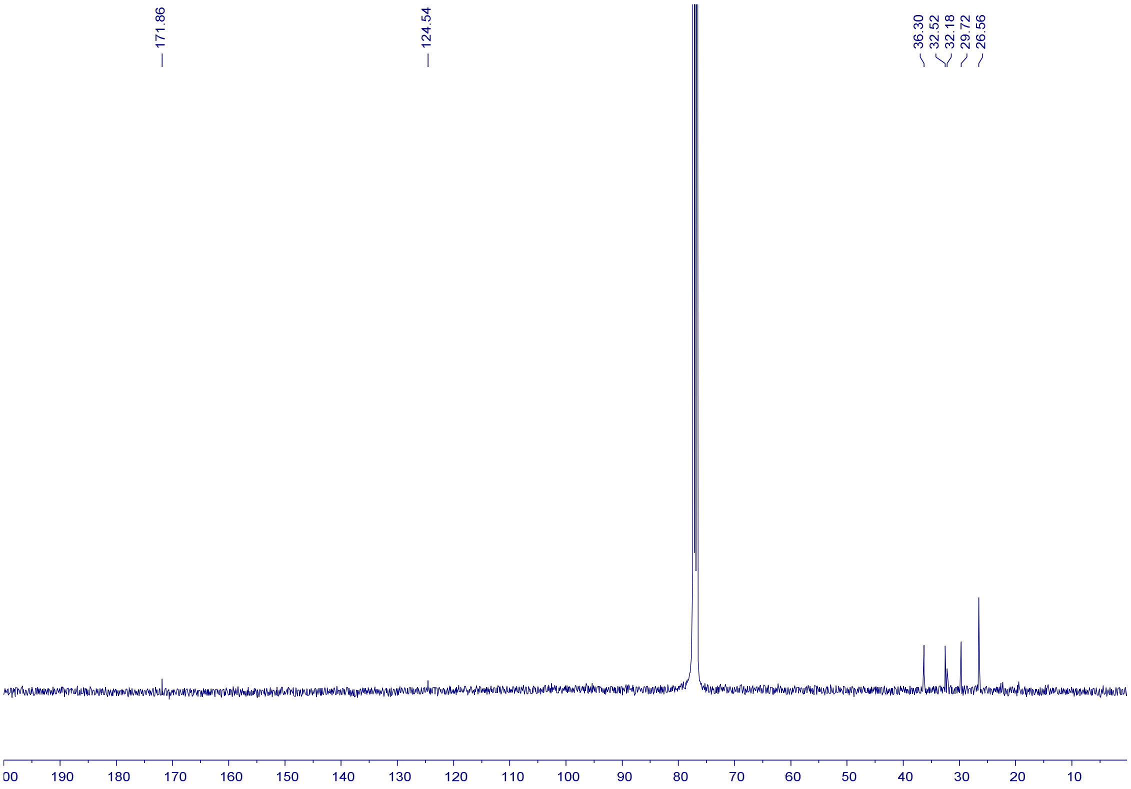

8 ^{1}H NMR (400 MHz, $CDCl_3$)

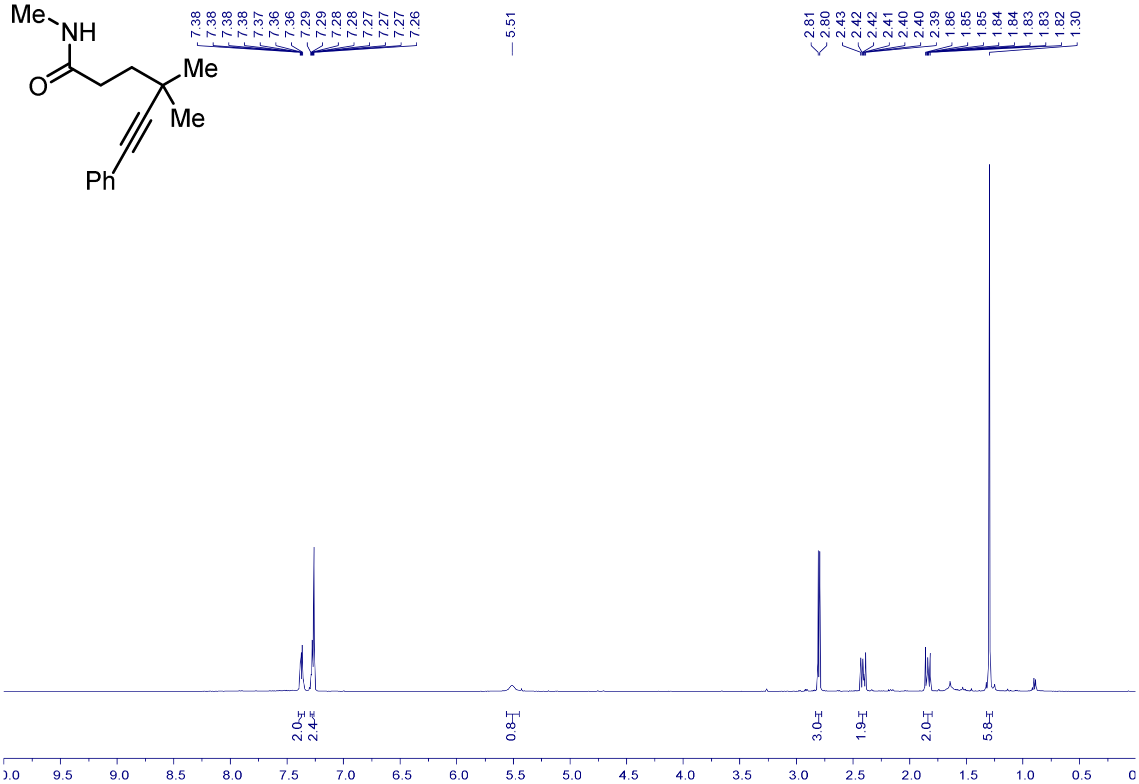

8 ^{13}C NMR (101 MHz, $CDCl_3$)

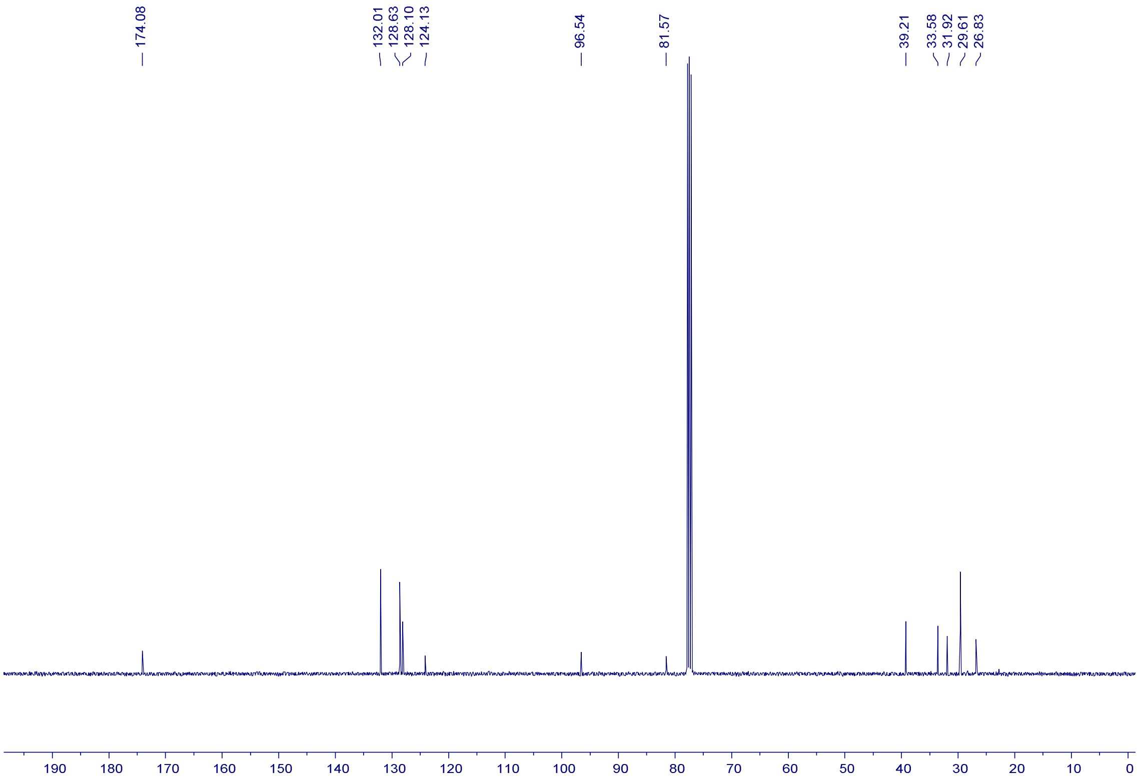

9 ^{1}H NMR (400 MHz, $CDCl_3$)

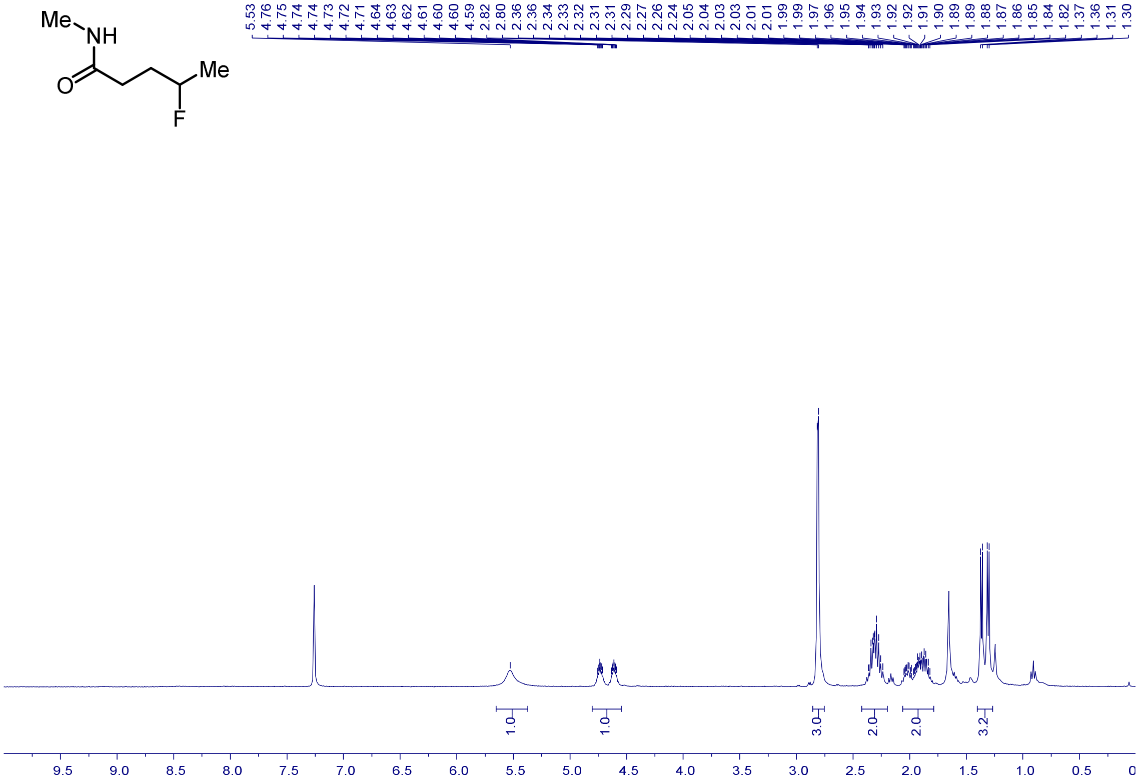

9 ^{13}C NMR (101 MHz, $CDCl_3$)

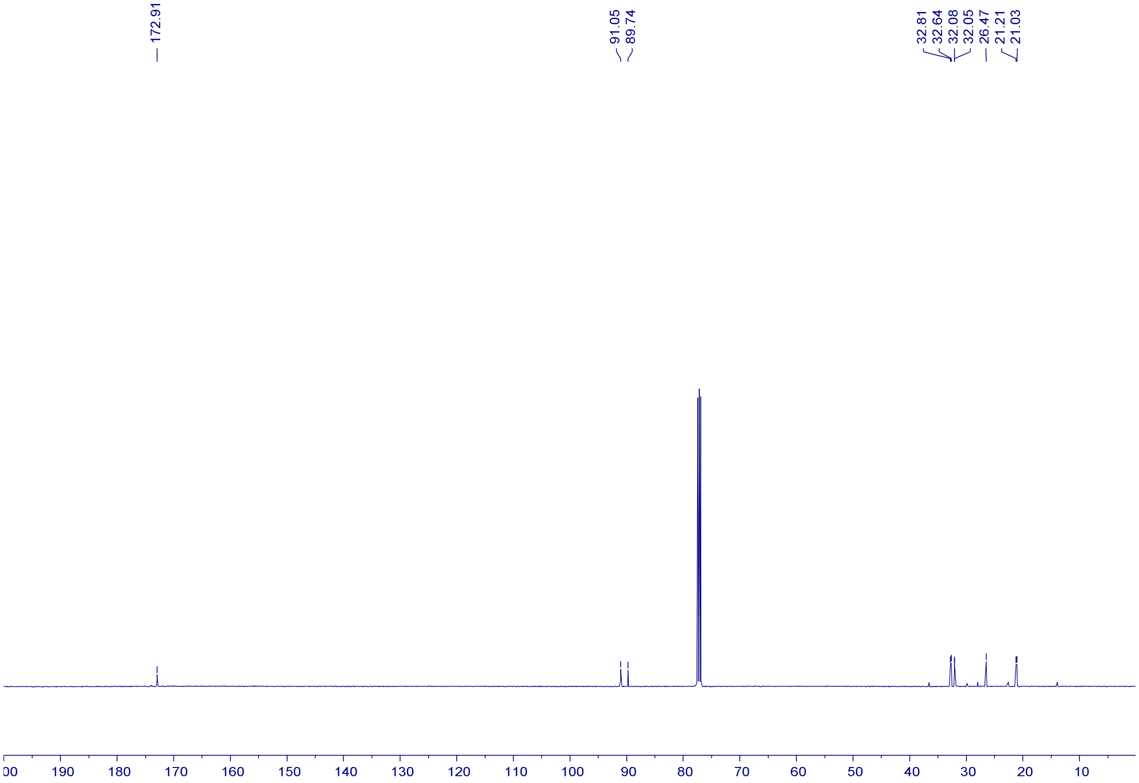

9 ^{19}F NMR (376 MHz, $CDCl_3$)

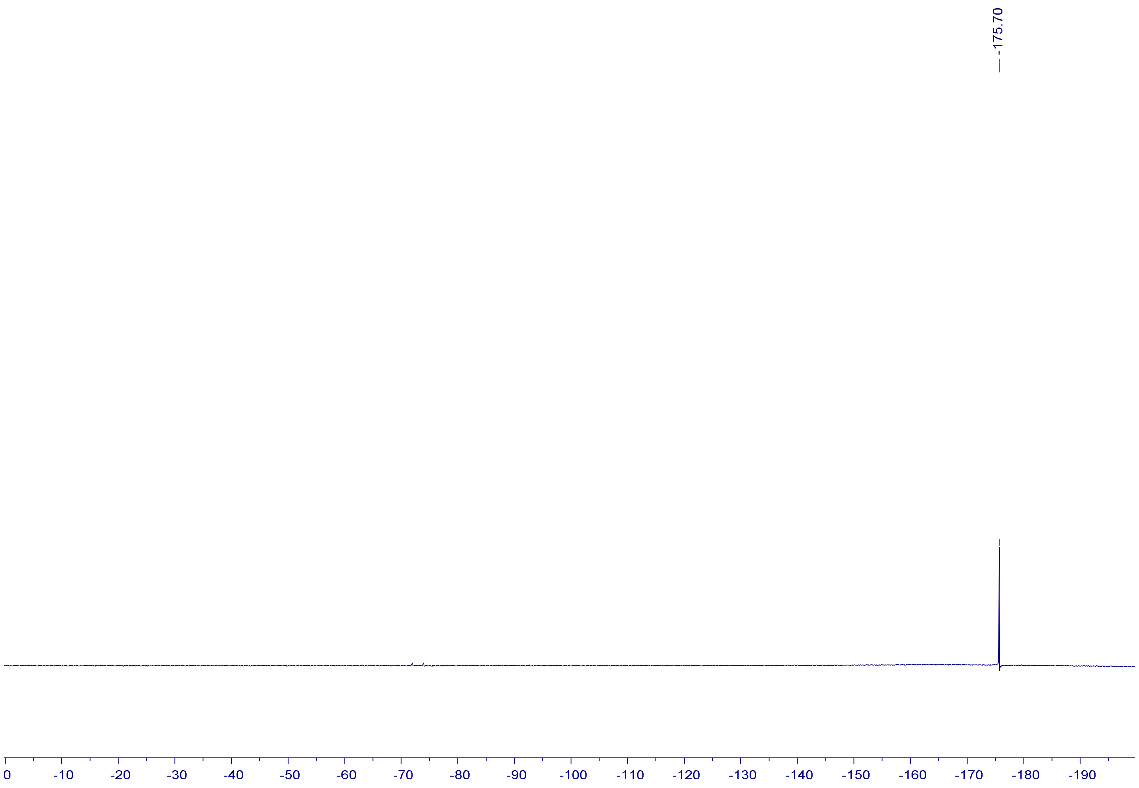

10 ^{1}H NMR (400 MHz, $CDCl_3$)

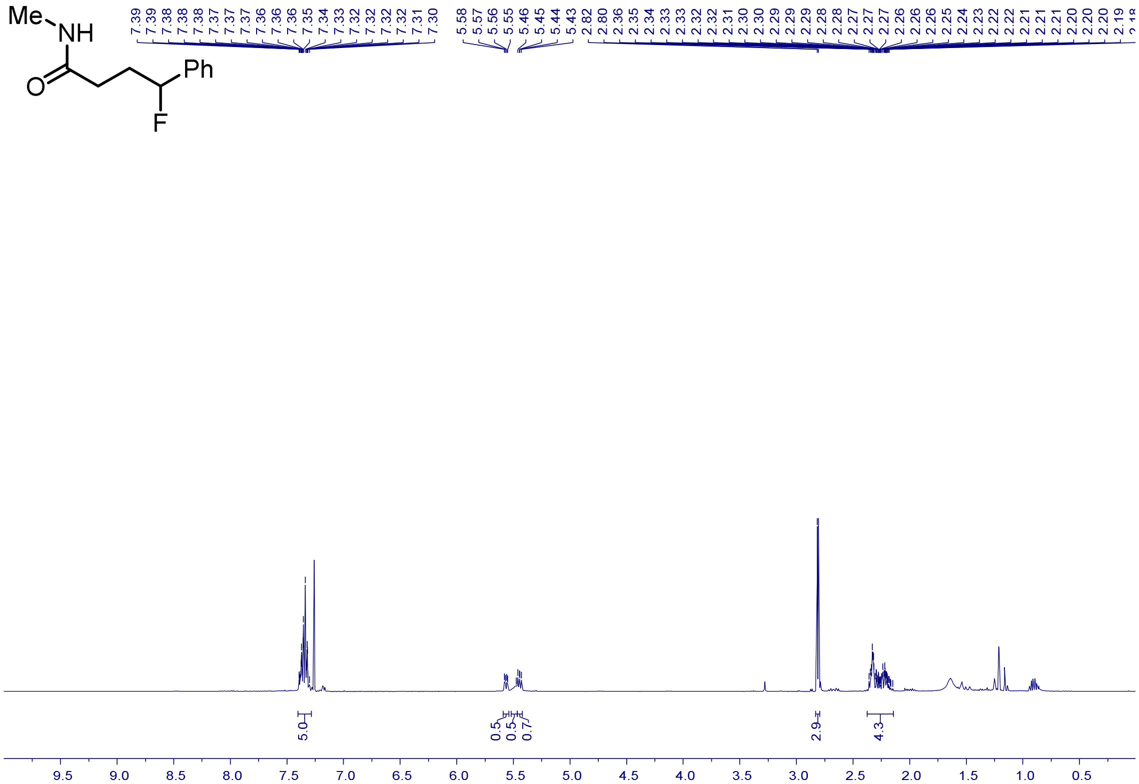

10 ^{13}C NMR (101 MHz, $CDCl_3$)

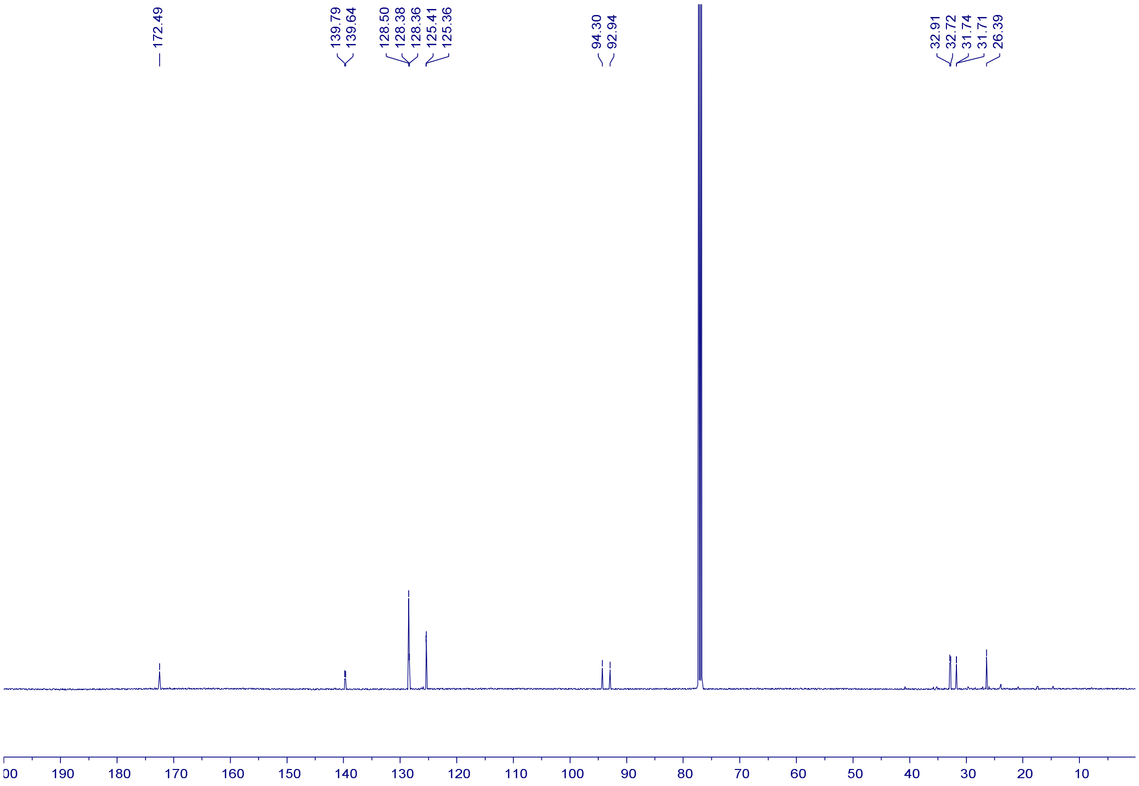

10 ^{19}F NMR (376 MHz, $CDCl_3$)

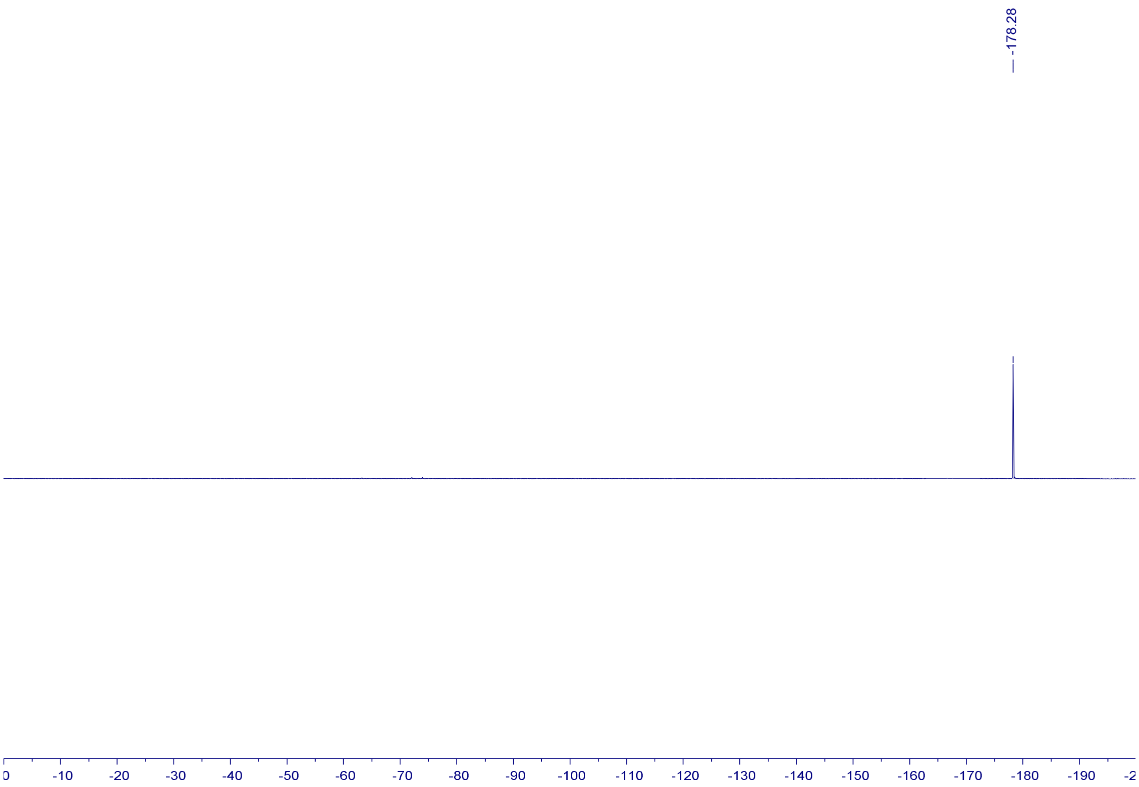

11 ^{1}H NMR (400 MHz, $CDCl_3$)

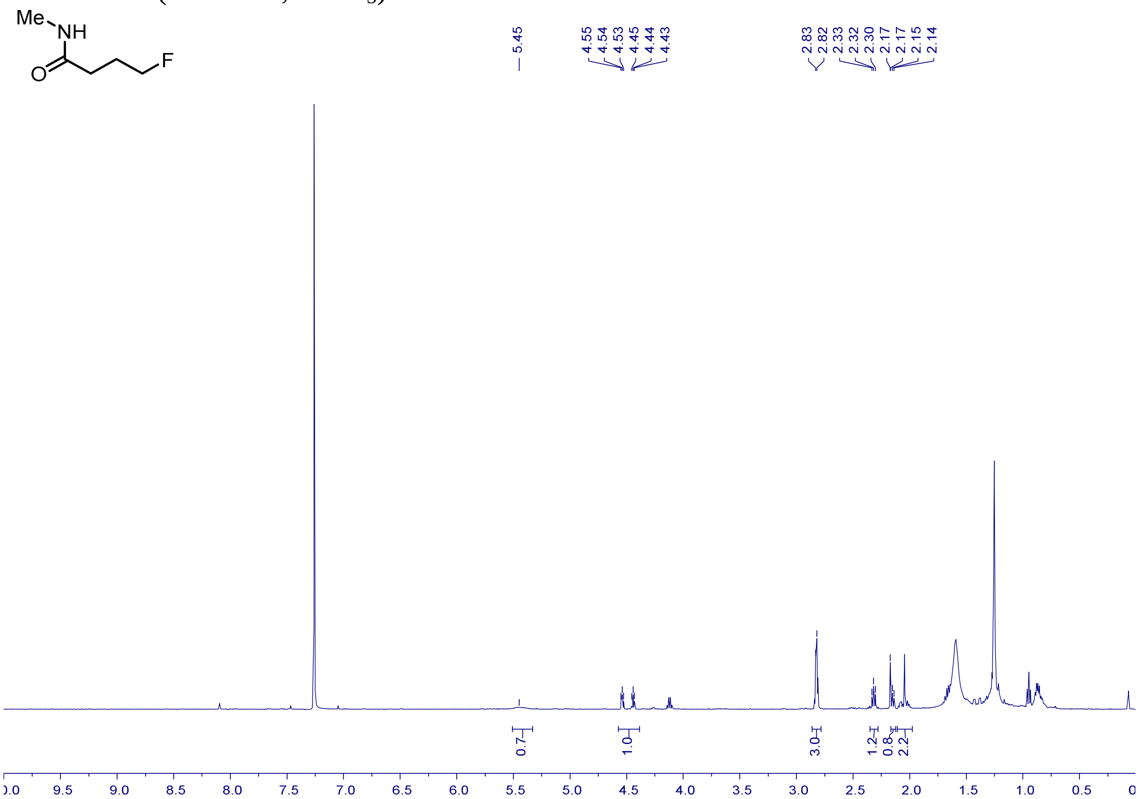

11 ^{19}F NMR (376 MHz, $CDCl_3$)

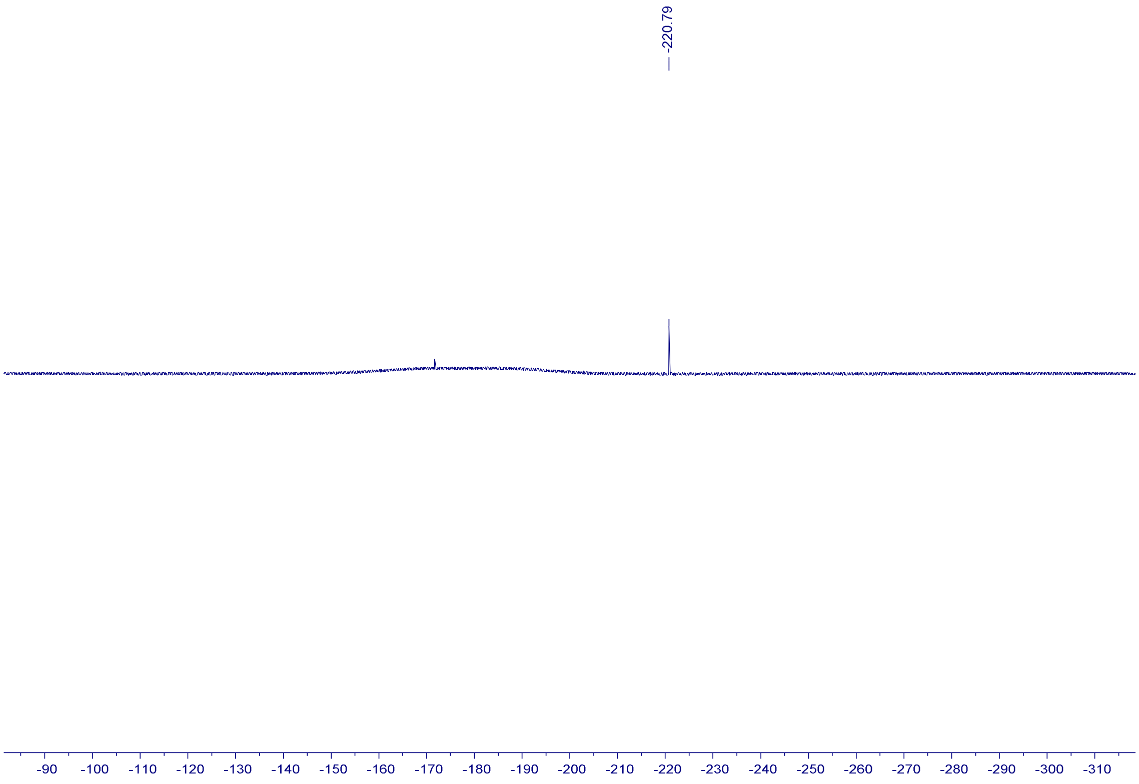

12 ^{1}H NMR (400 MHz, $CDCl_3$)

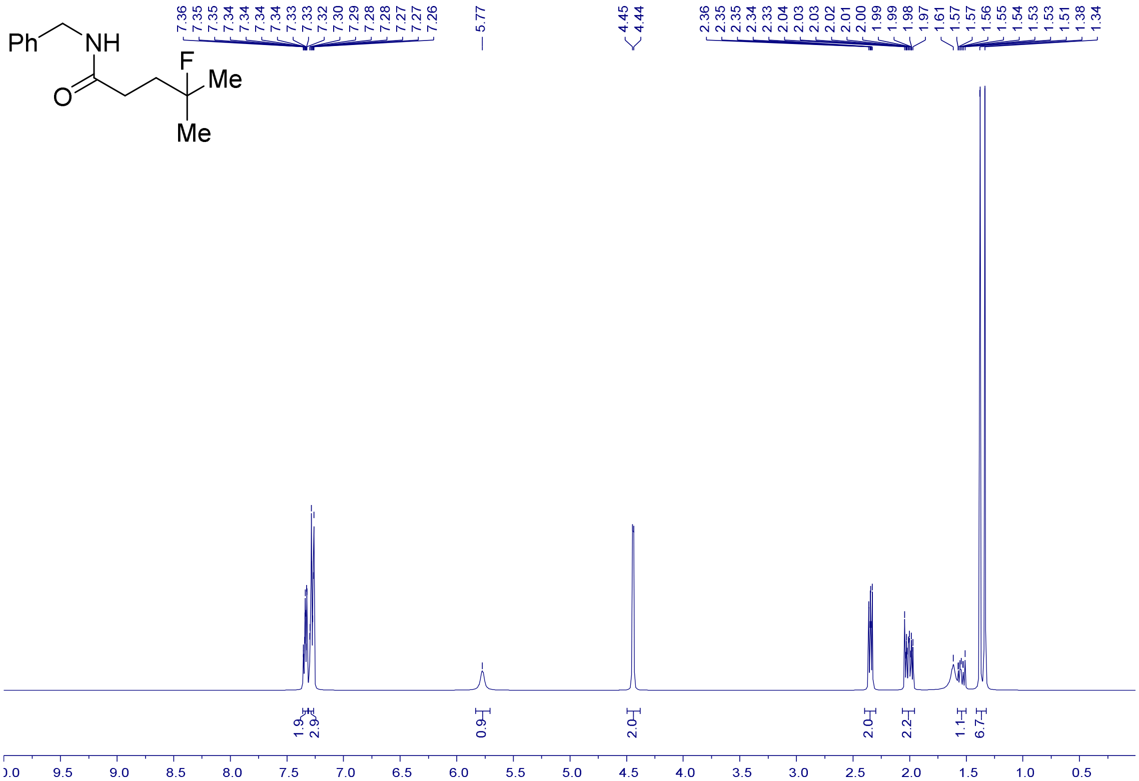

12 ^{13}C NMR (101 MHz, $CDCl_3$)

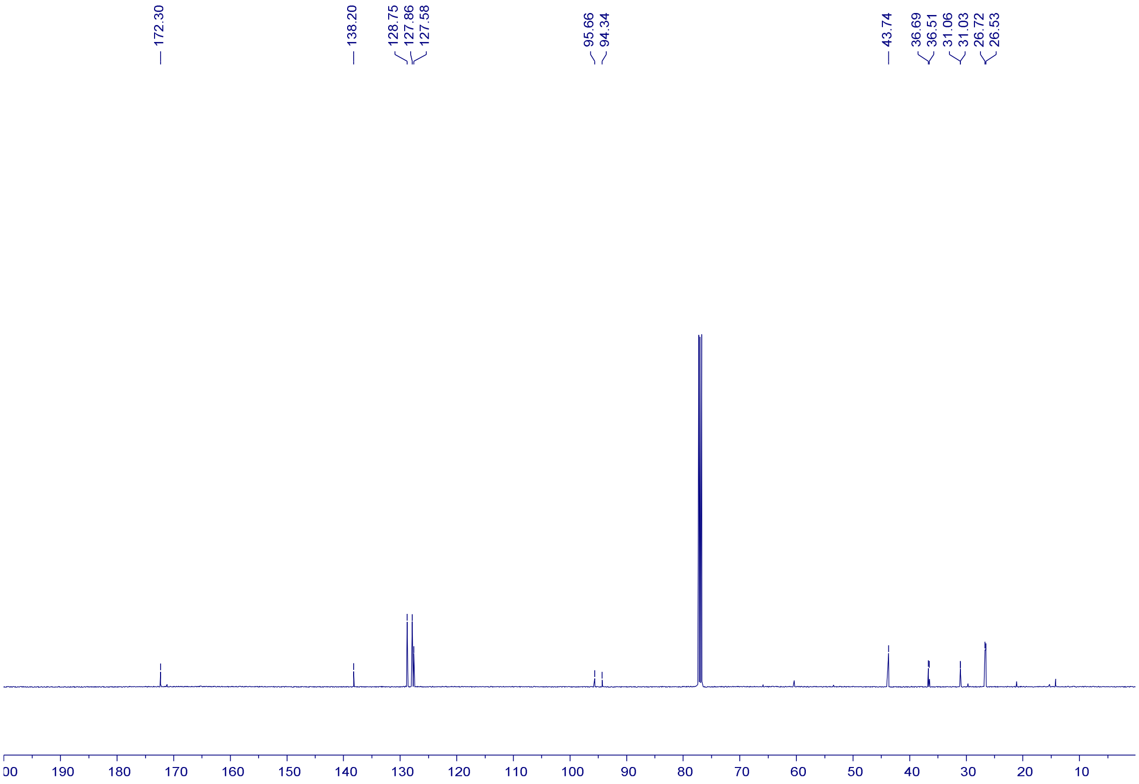

12 ^{19}F NMR (376 MHz, $CDCl_3$)

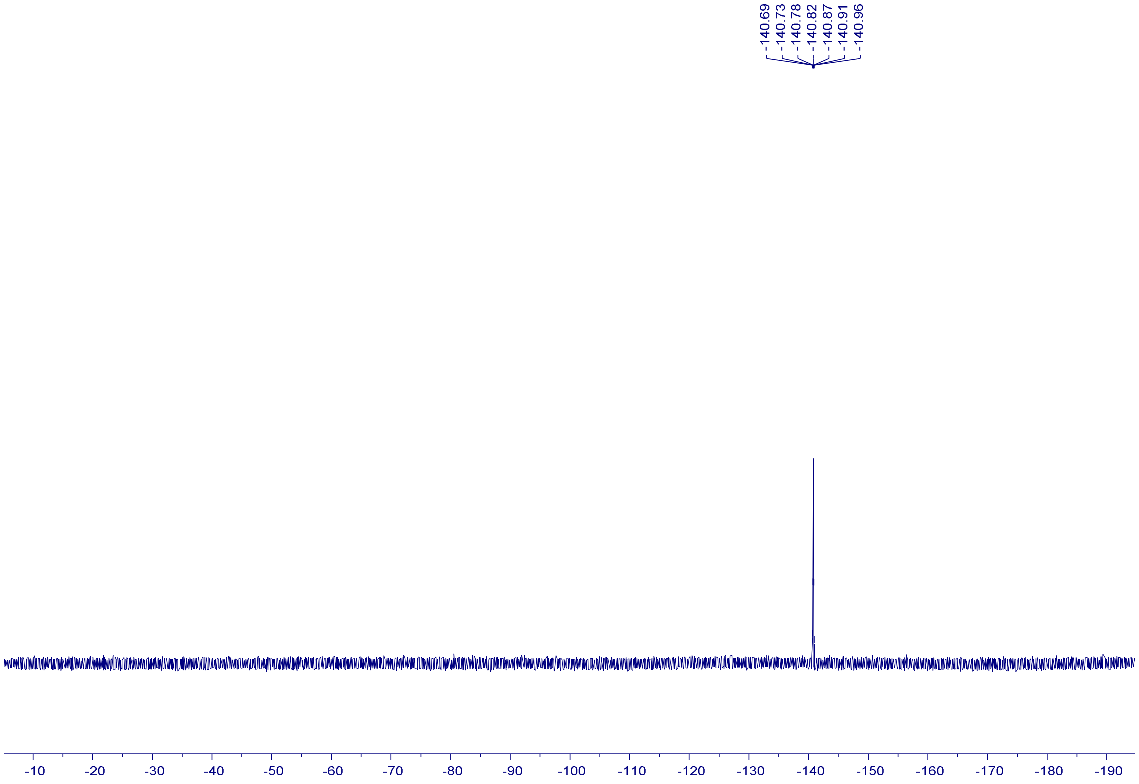

16 ^{1}H NMR (400 MHz, $CDCl_3$)

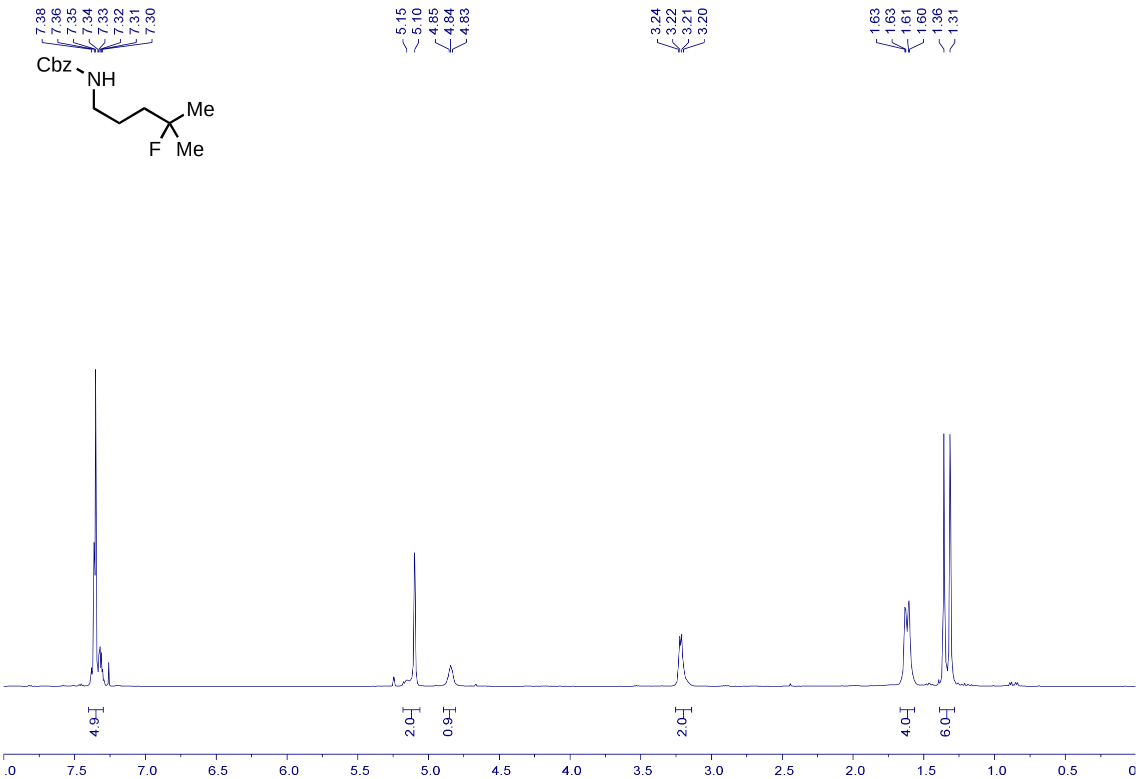

16 ^{13}C NMR (101 MHz, $CDCl_3$)

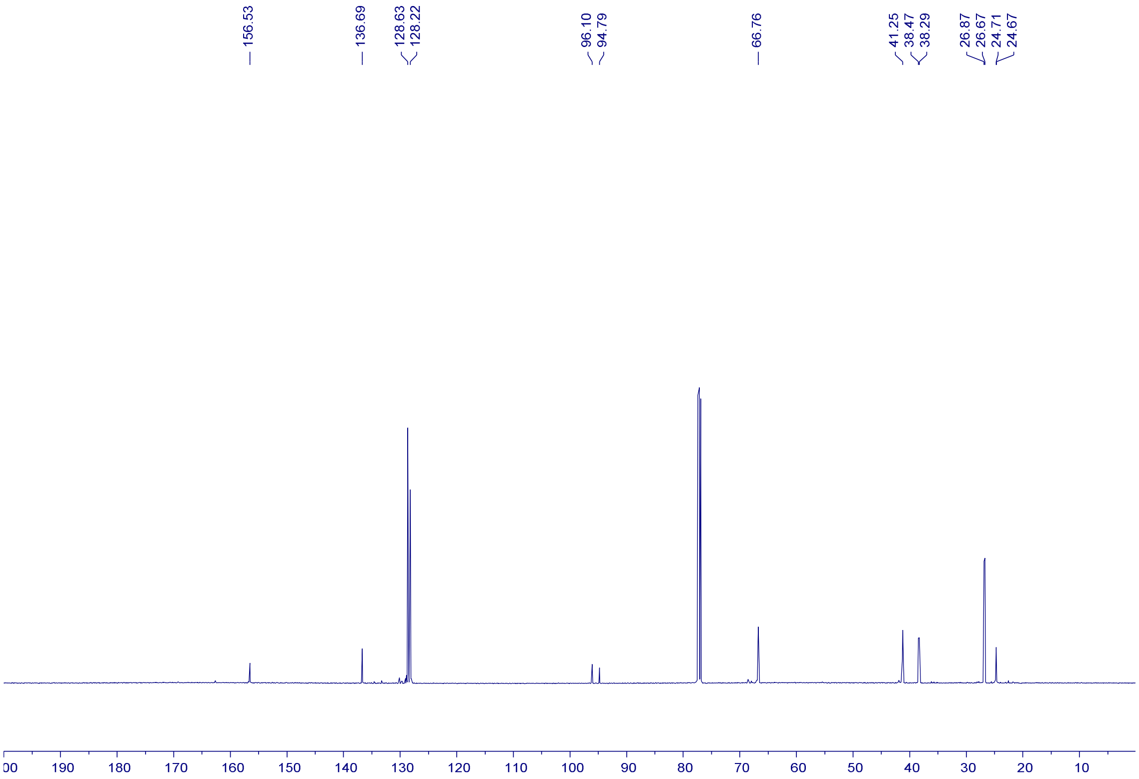

16 ^{19}F NMR (376 MHz, $CDCl_3$)

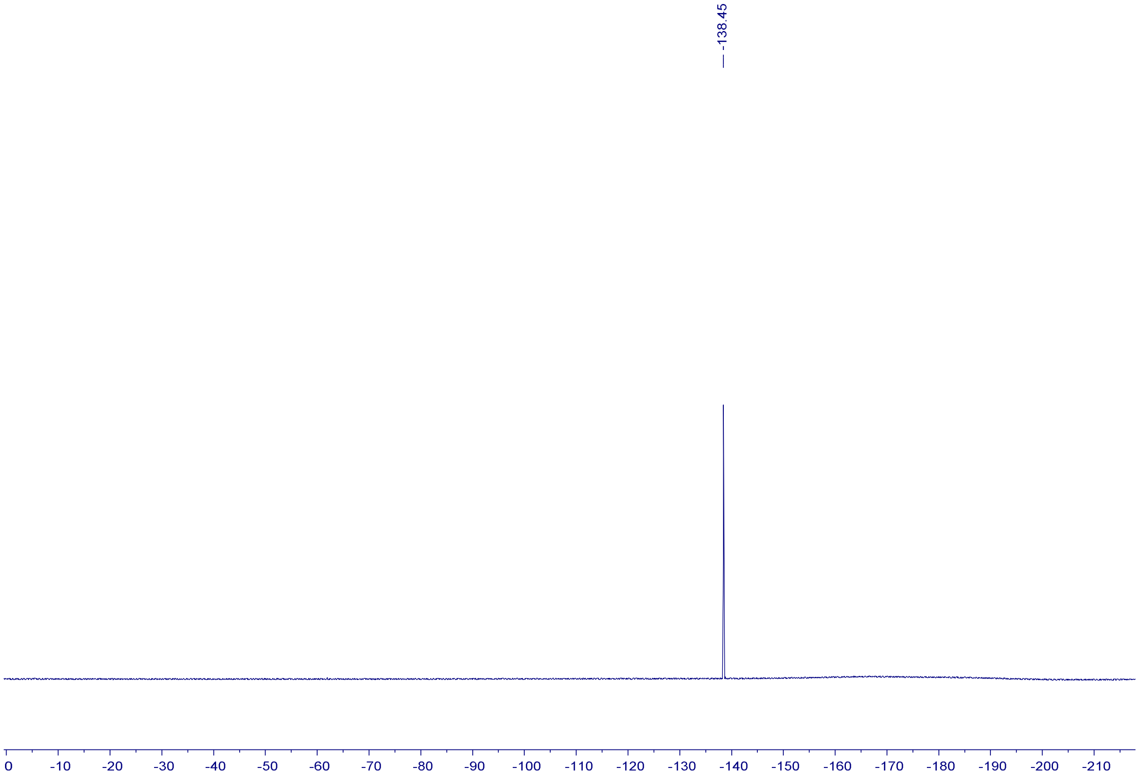

17 ^{1}H NMR (400 MHz, $CDCl_3$)

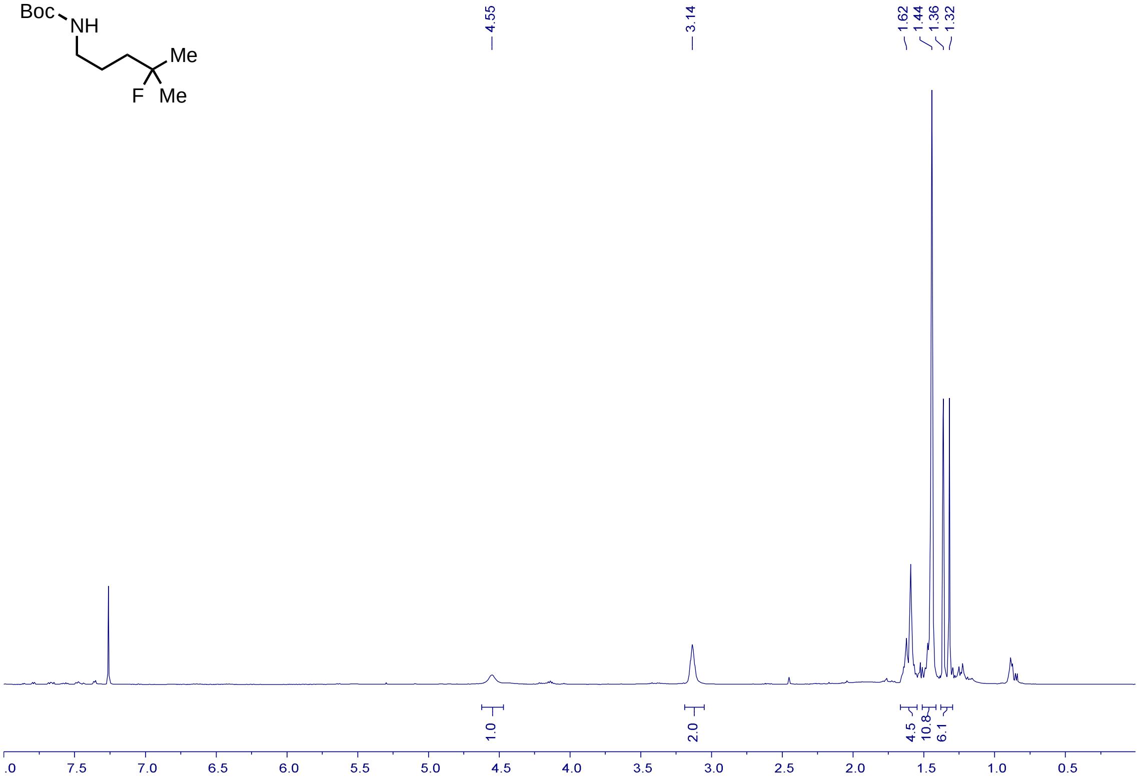

17 ^{13}C NMR (101 MHz, $CDCl_3$)

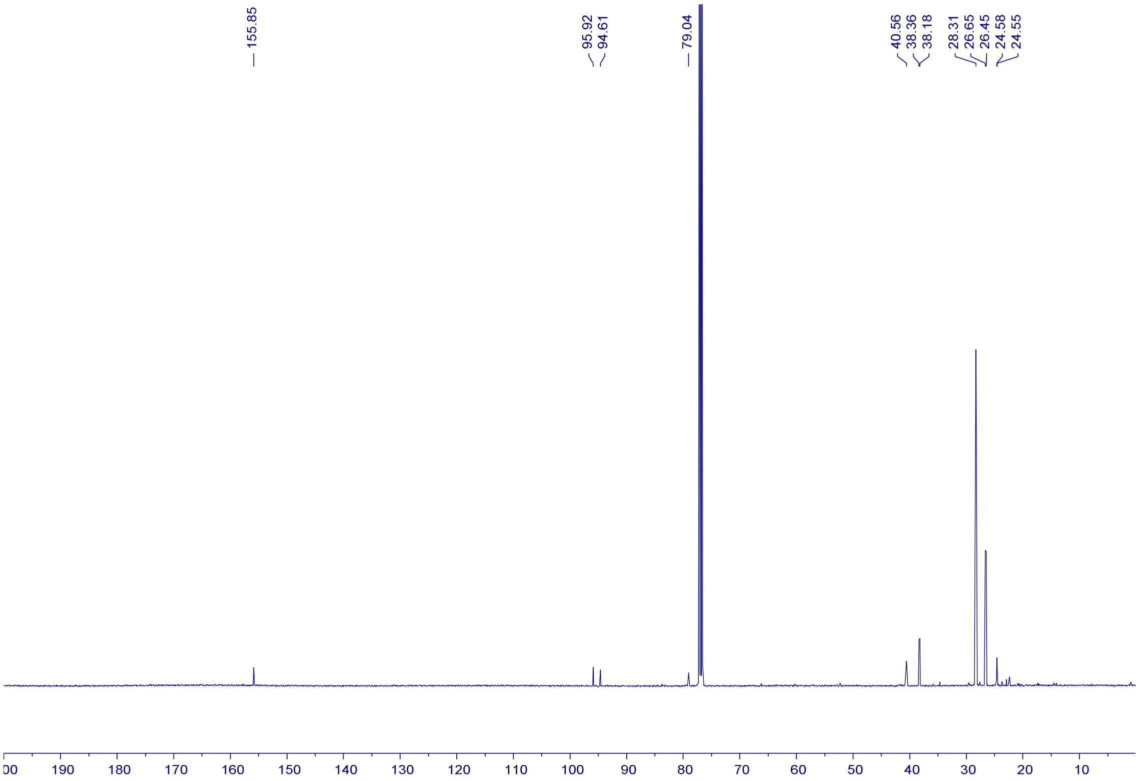

17 ^{19}F NMR (376 MHz, $CDCl_3$)

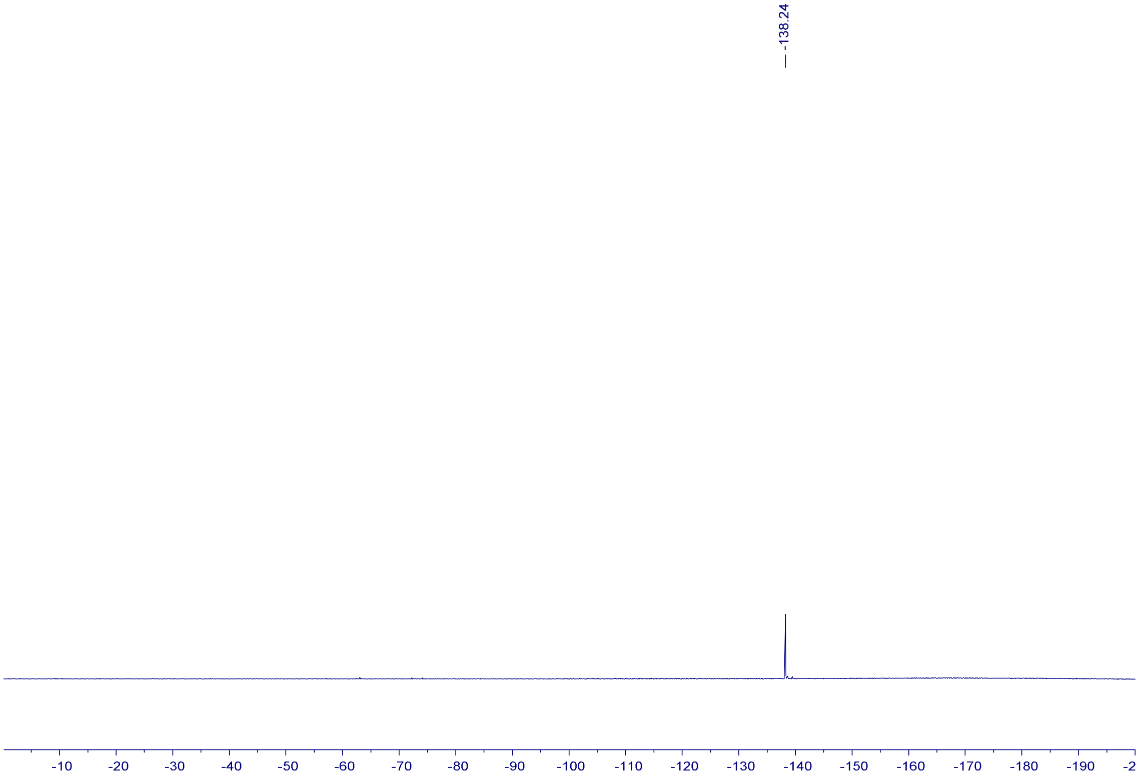

18 ^{1}H NMR (400 MHz, $CDCl_3$)

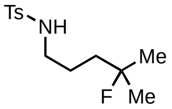

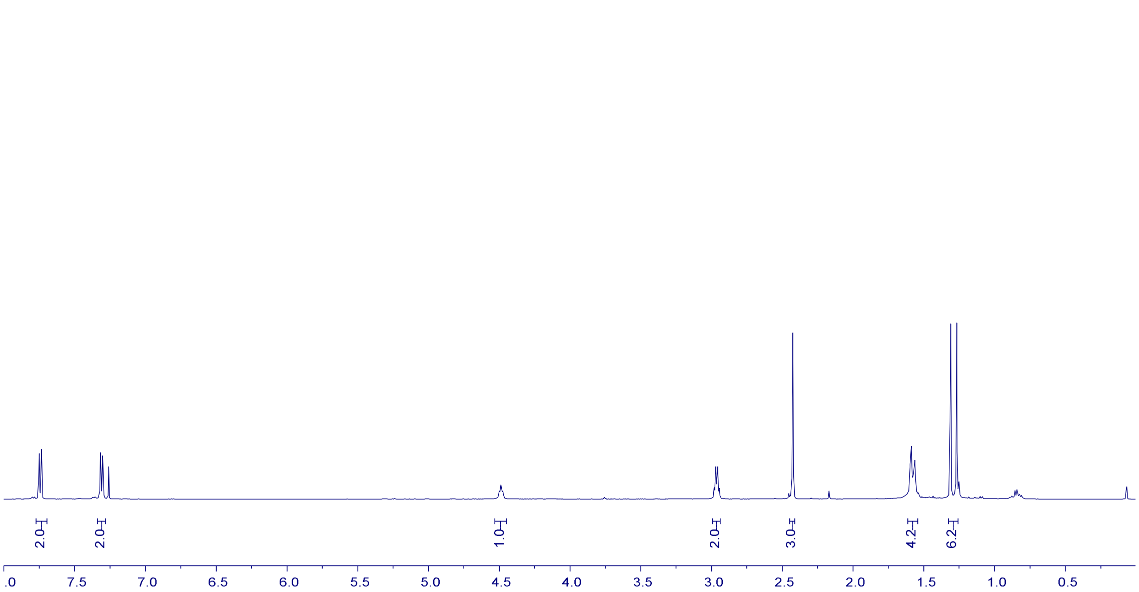

18 ^{13}C NMR (101 MHz, $CDCl_3$)

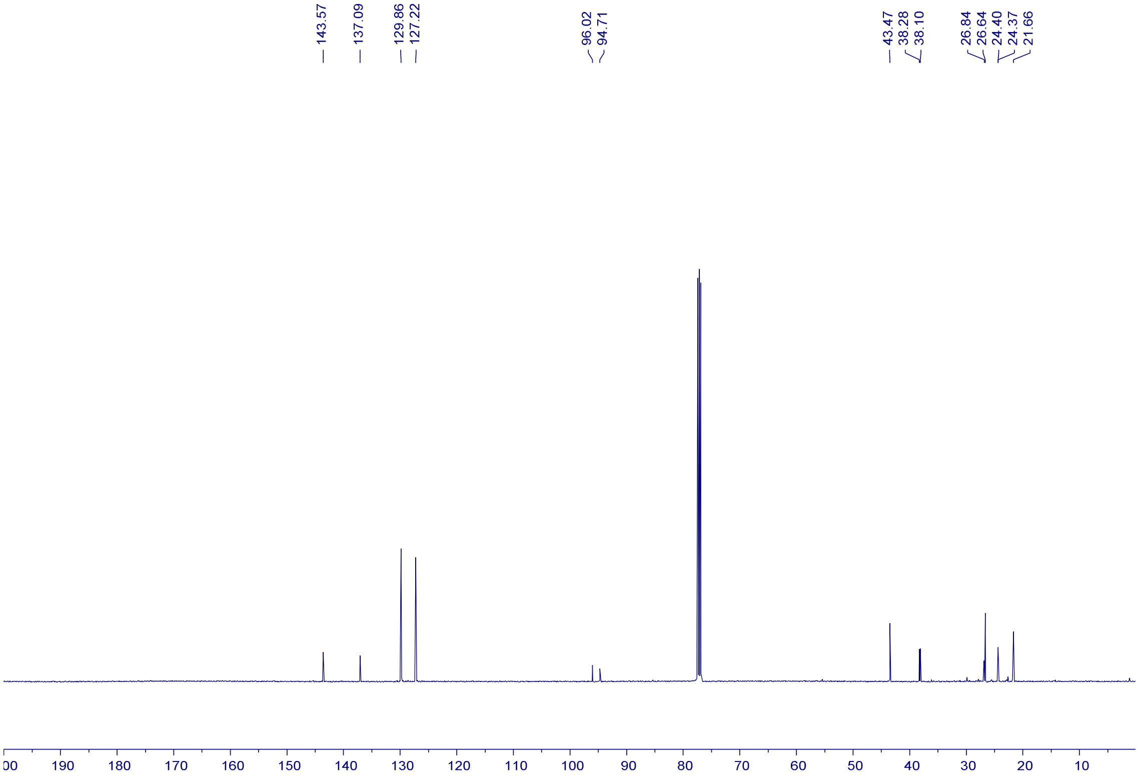

18 ^{19}F NMR (376 MHz, $CDCl_3$)

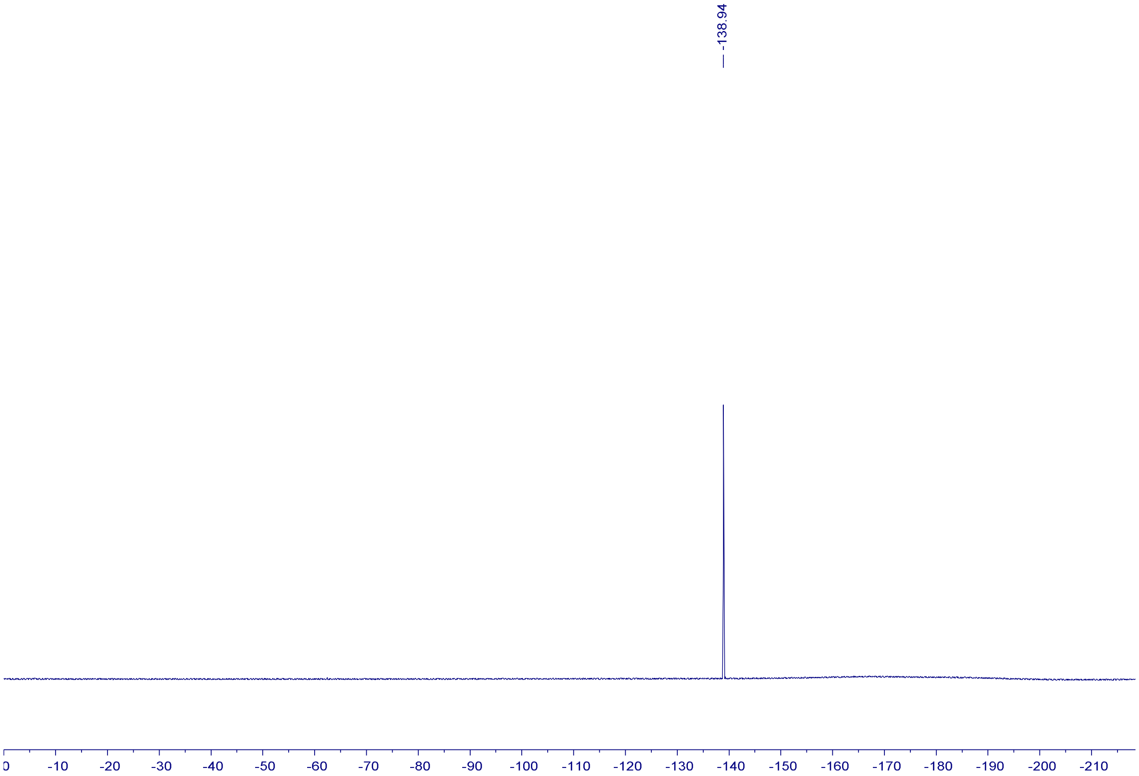

19 ^{1}H NMR (400 MHz, $CDCl_3$)

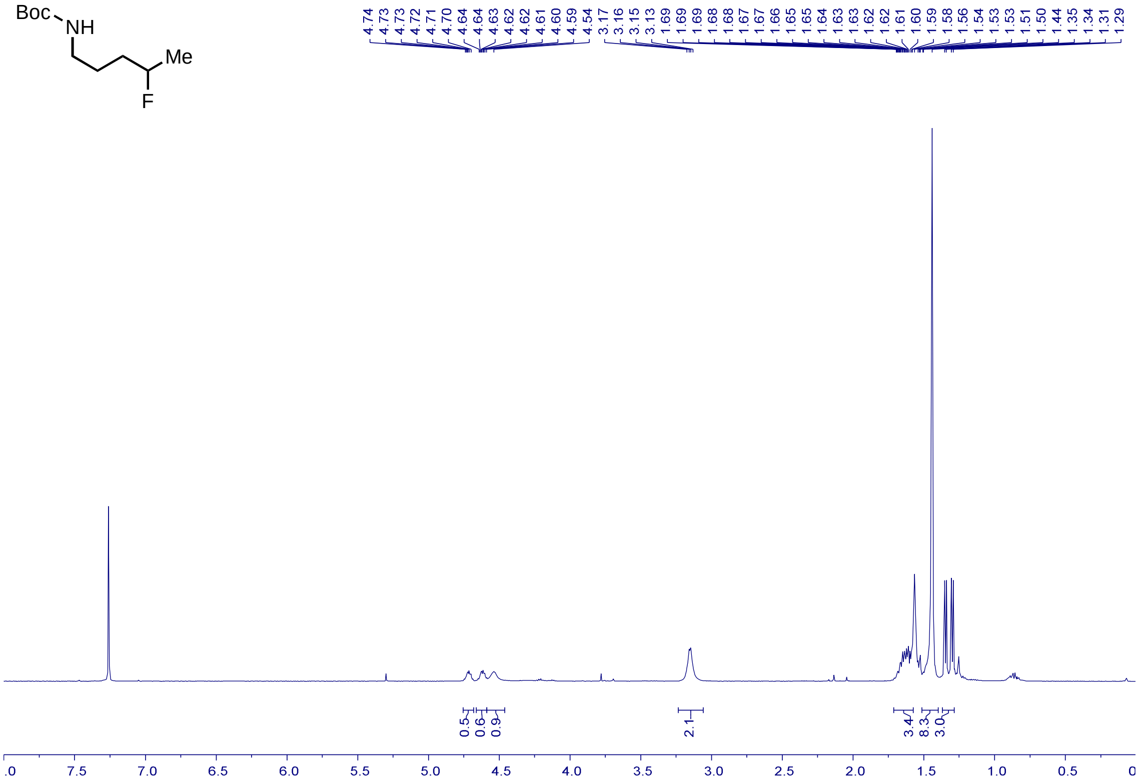

19 ^{13}C NMR (101 MHz, $CDCl_3$)

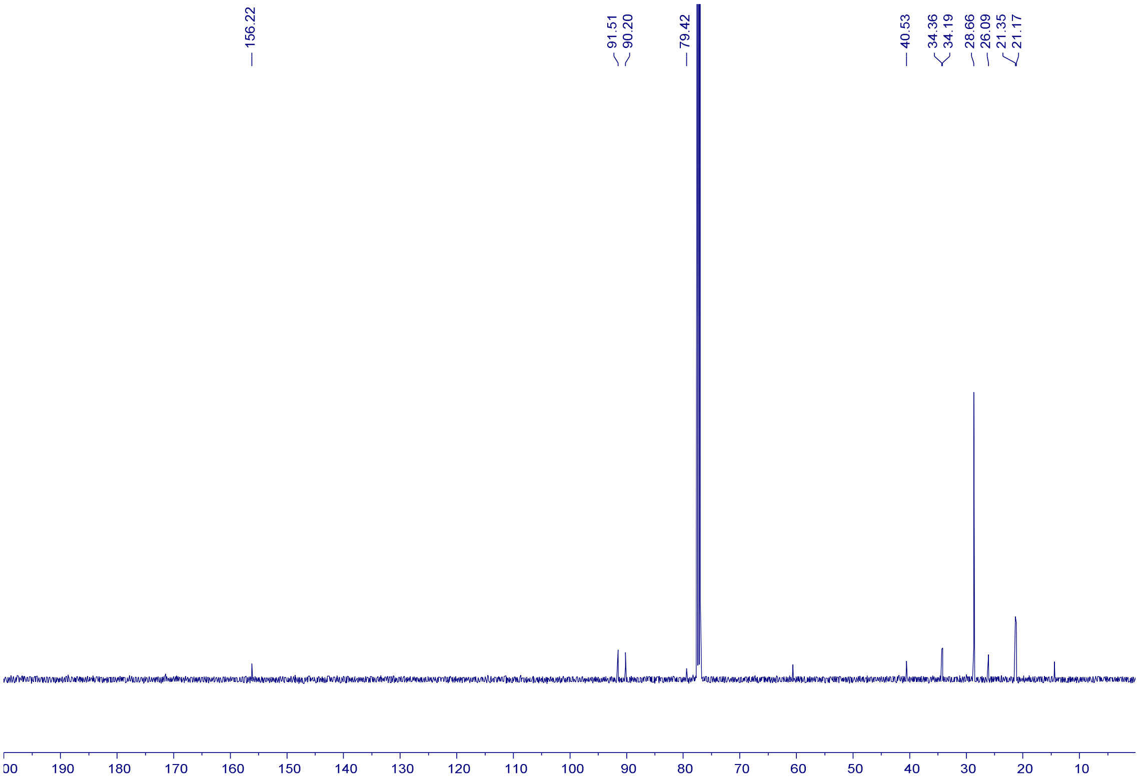

19 ^{19}F NMR (376 MHz, $CDCl_3$)

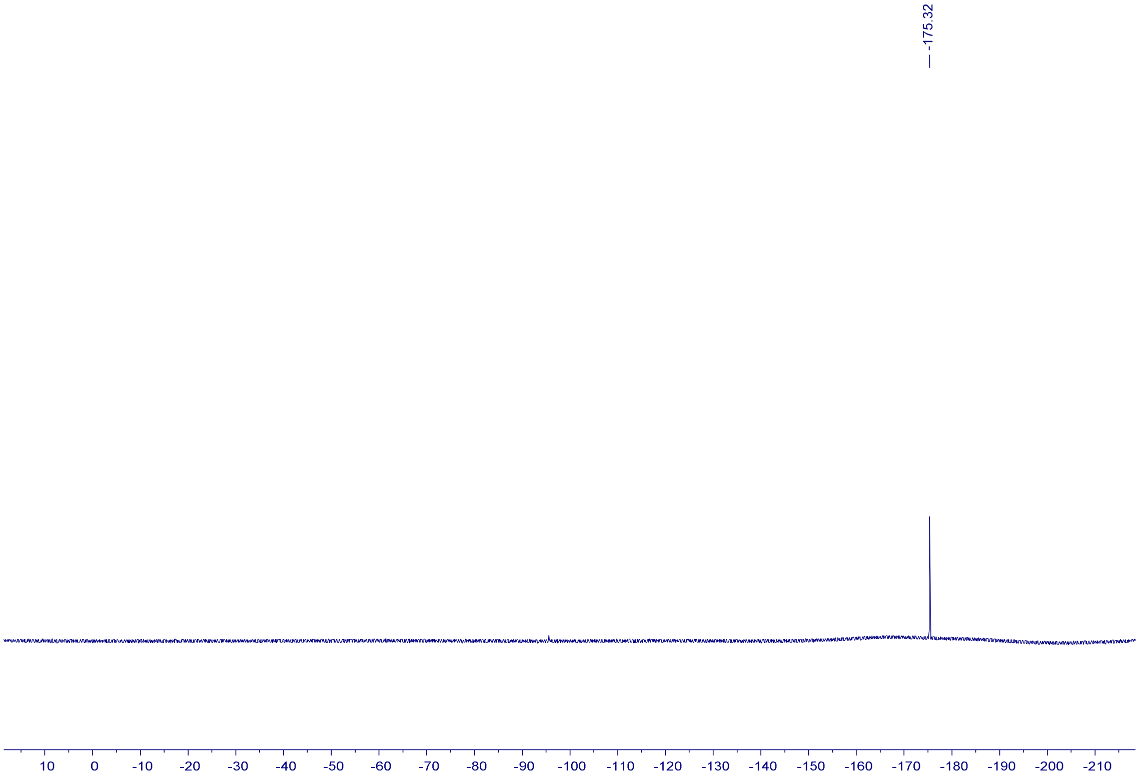

20 ^{1}H NMR (400 MHz, $CDCl_3$)

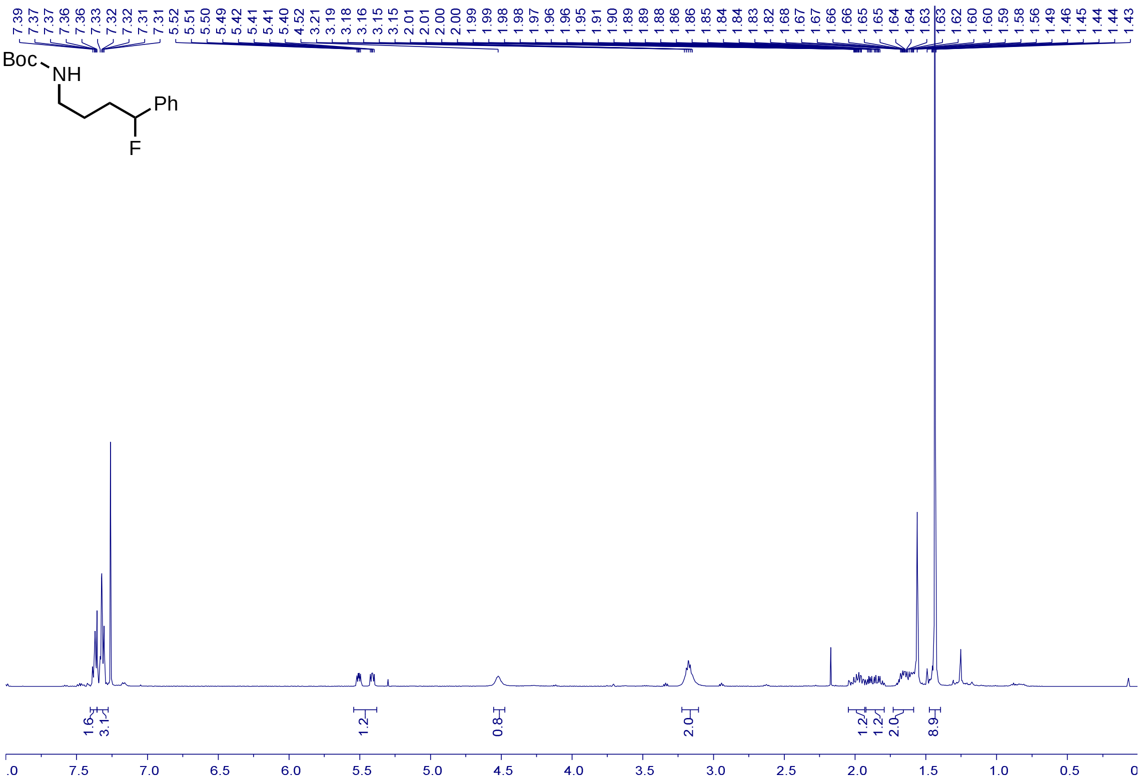

20 ^{13}C NMR (101 MHz, $CDCl_3$)

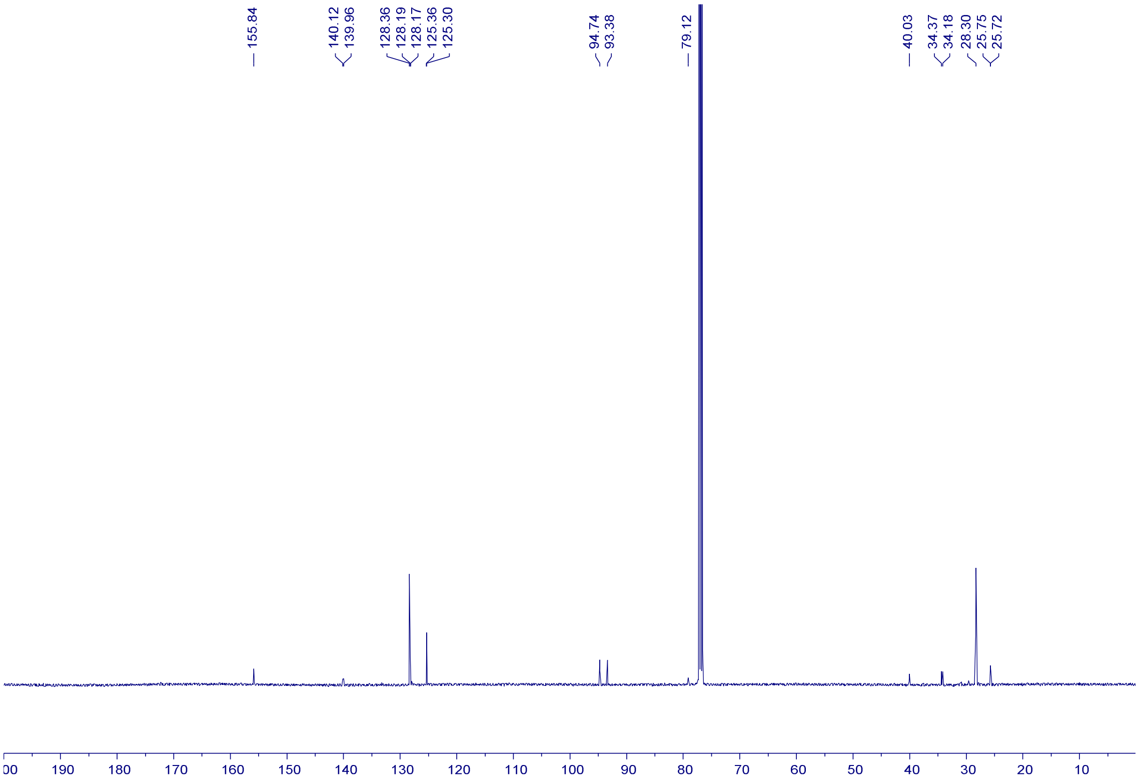

20 ^{19}F NMR (376 MHz, $CDCl_3$)

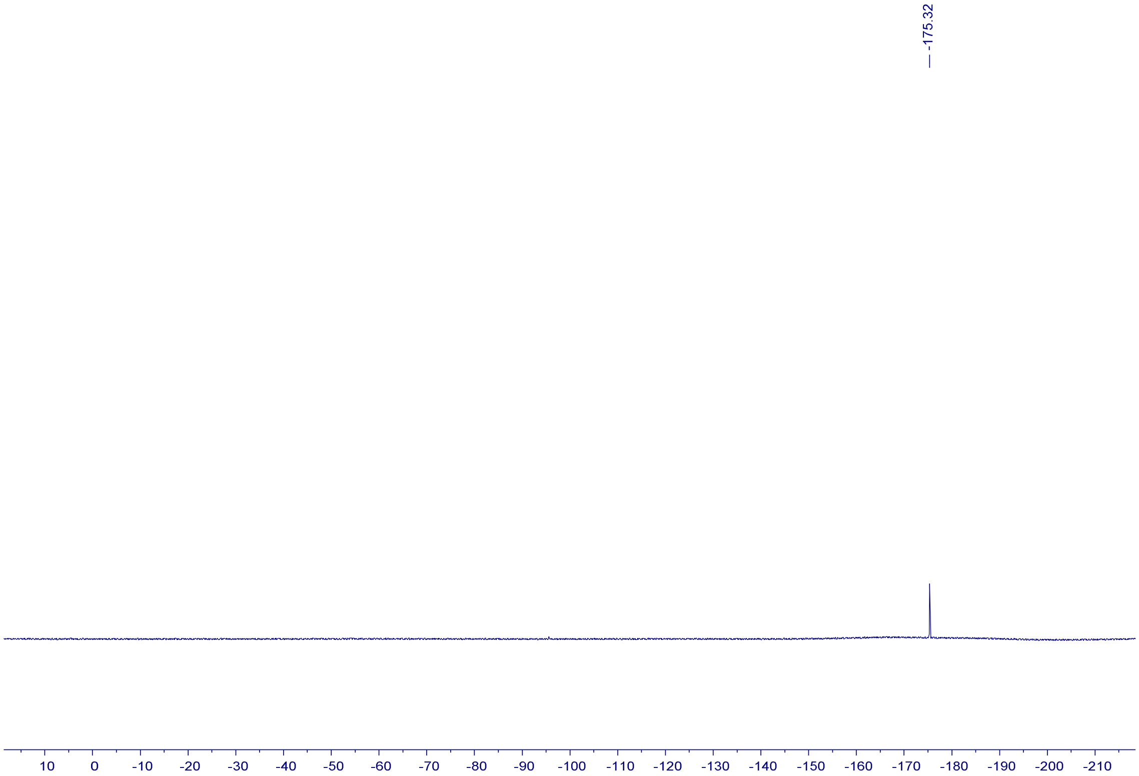

21 ^{1}H NMR (400 MHz, $CDCl_3$)

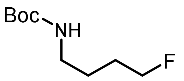

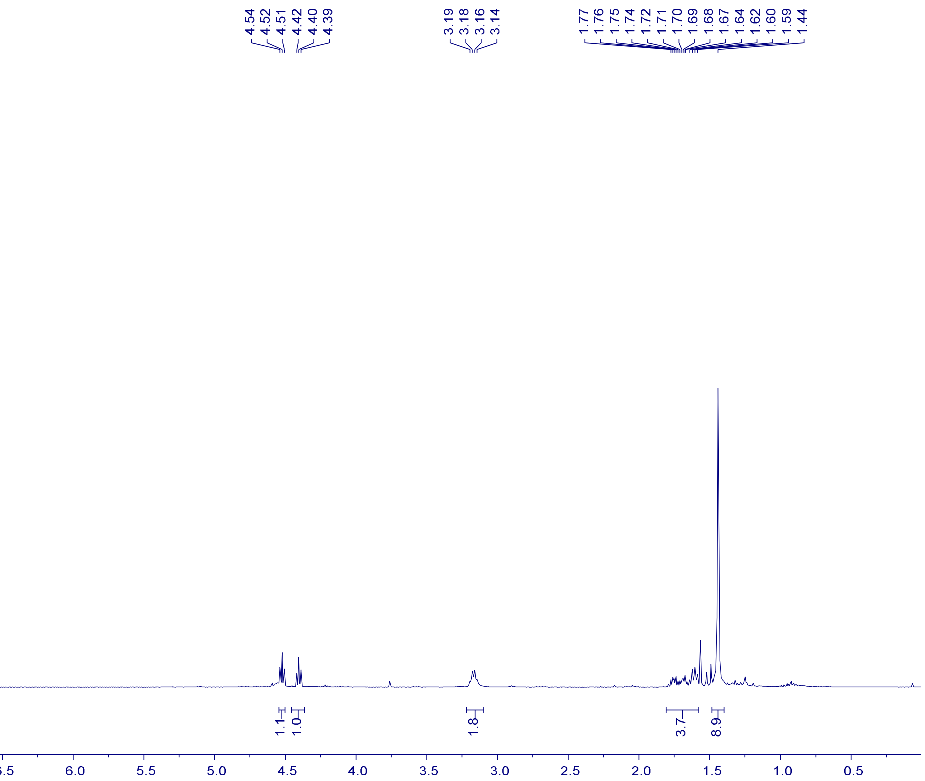

21 ^{13}C NMR (101 MHz, $CDCl_3$)

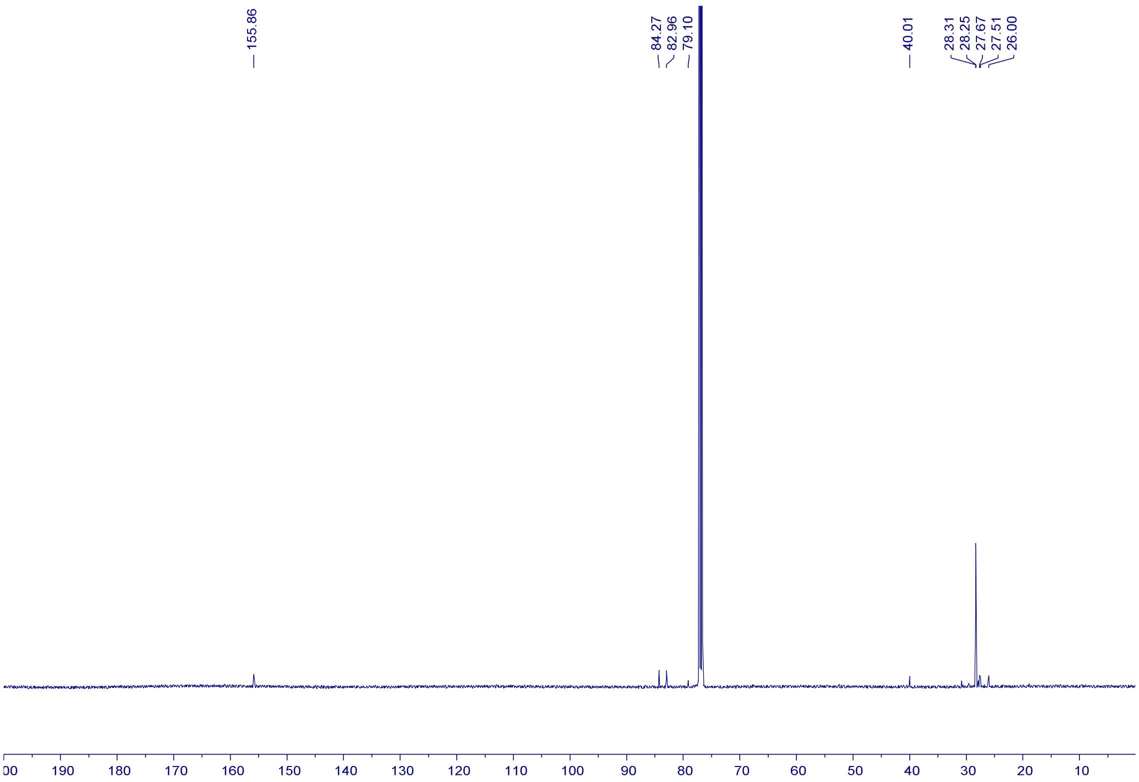

21 ^{19}F NMR (376 MHz, $CDCl_3$)

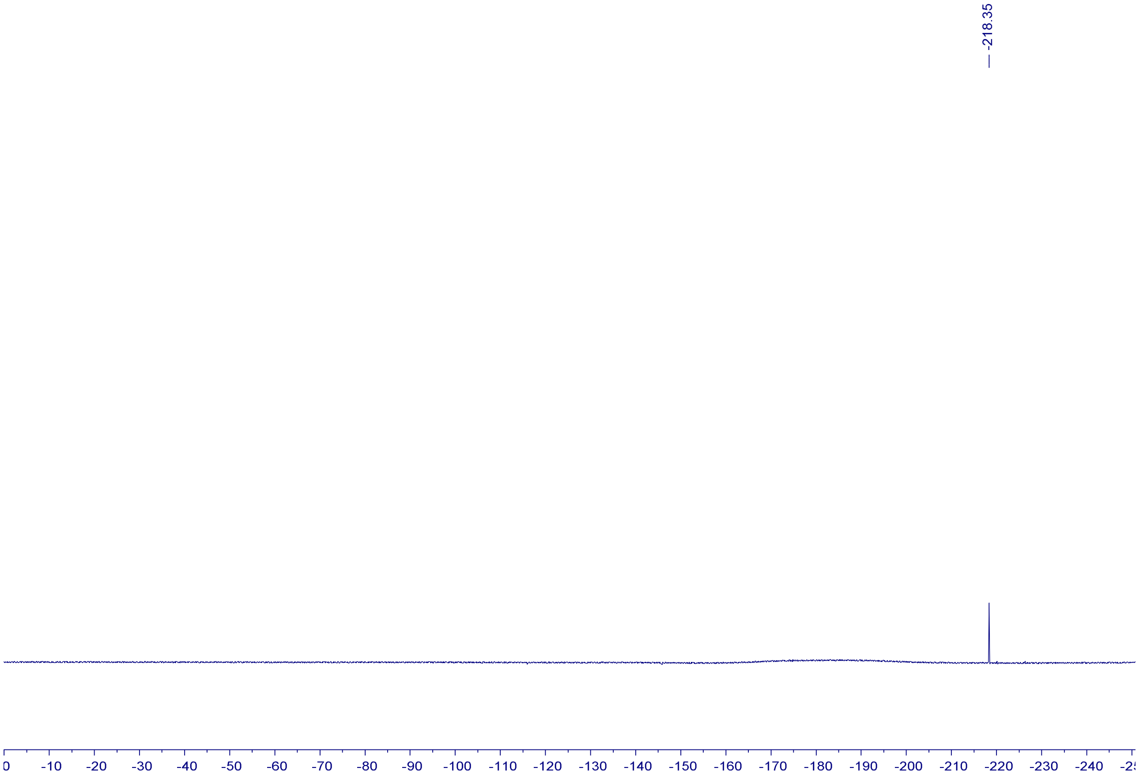

22 ^{1}H NMR (400 MHz, $CDCl_3$)

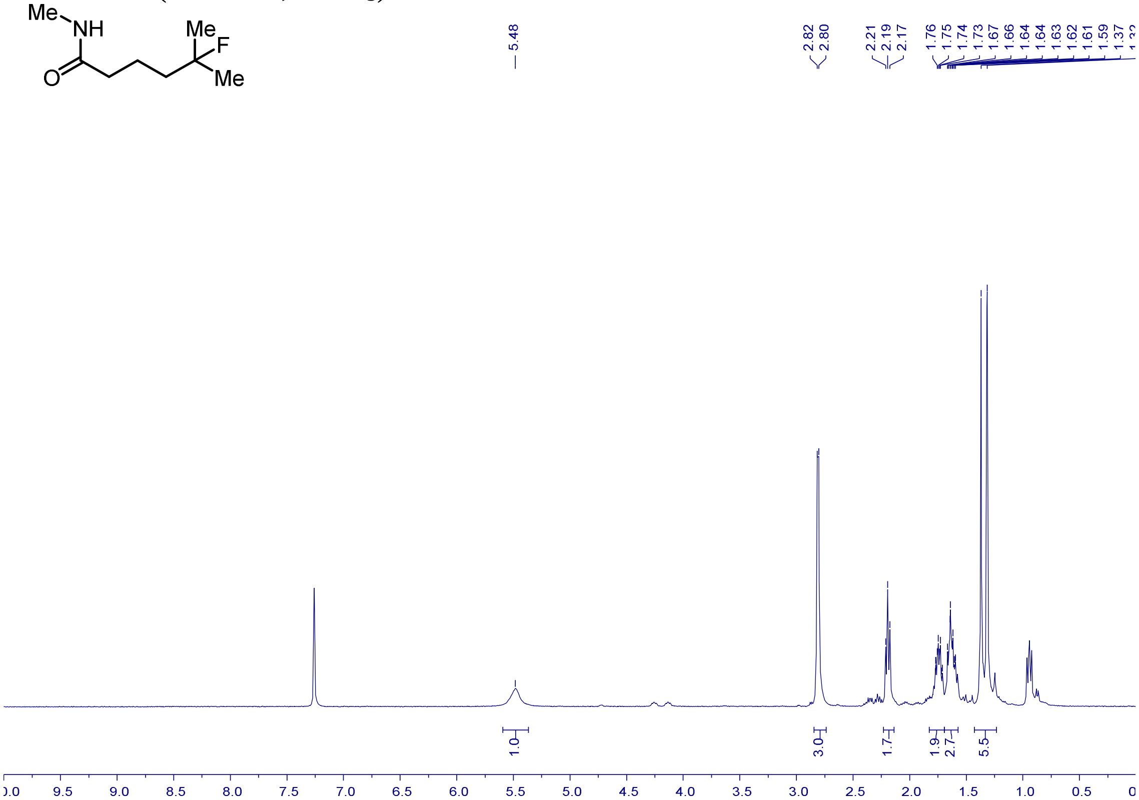

22 ^{13}C NMR (101 MHz, $CDCl_3$)

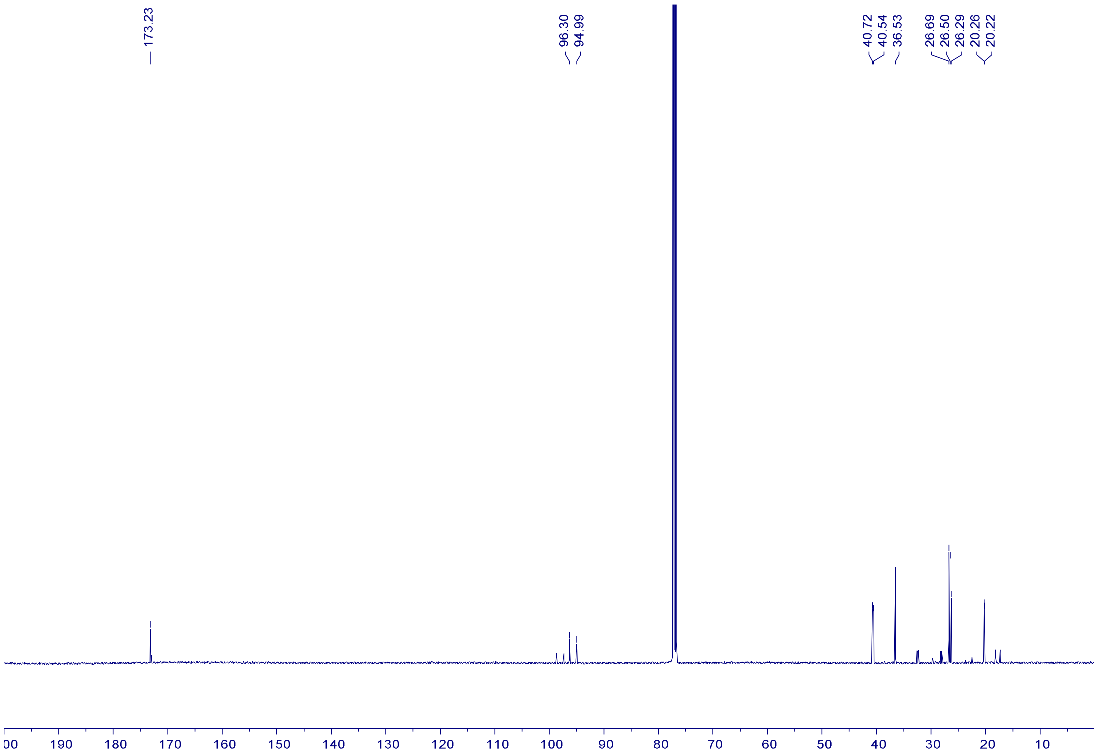

22 ^{19}F NMR (376 MHz, $CDCl_3$)

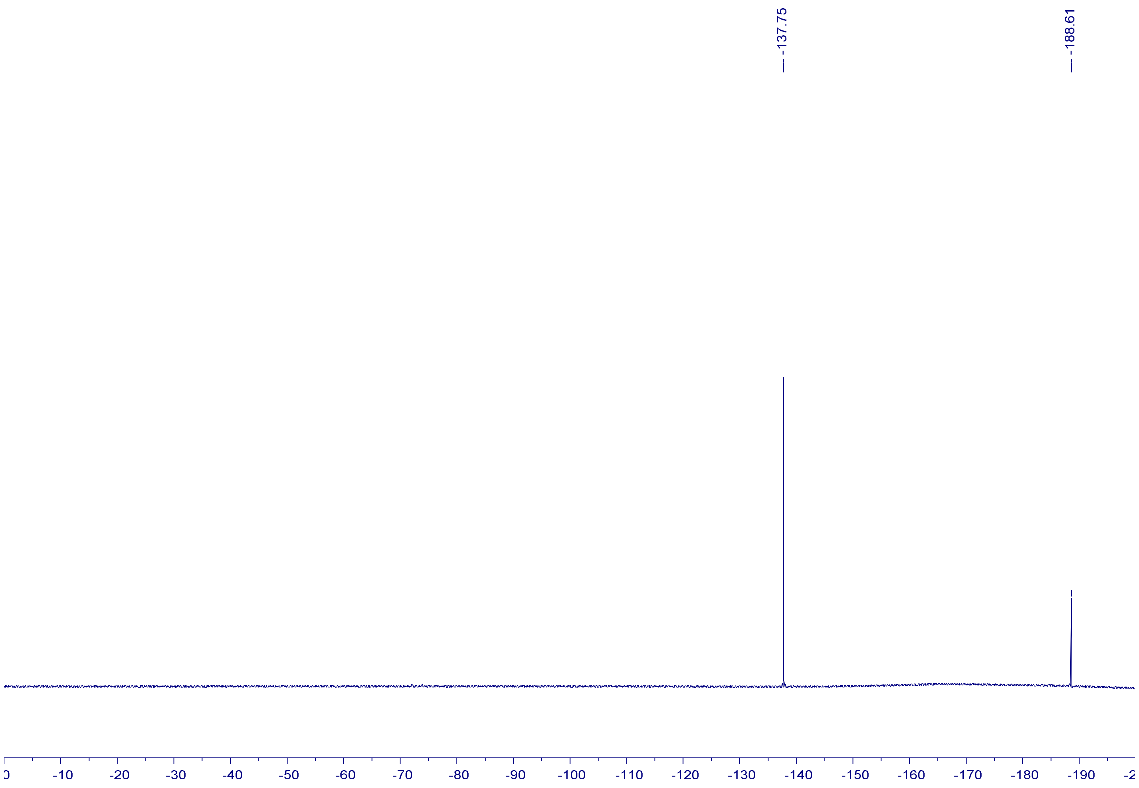

24 ^{1}H NMR (400 MHz, $CDCl_3$)

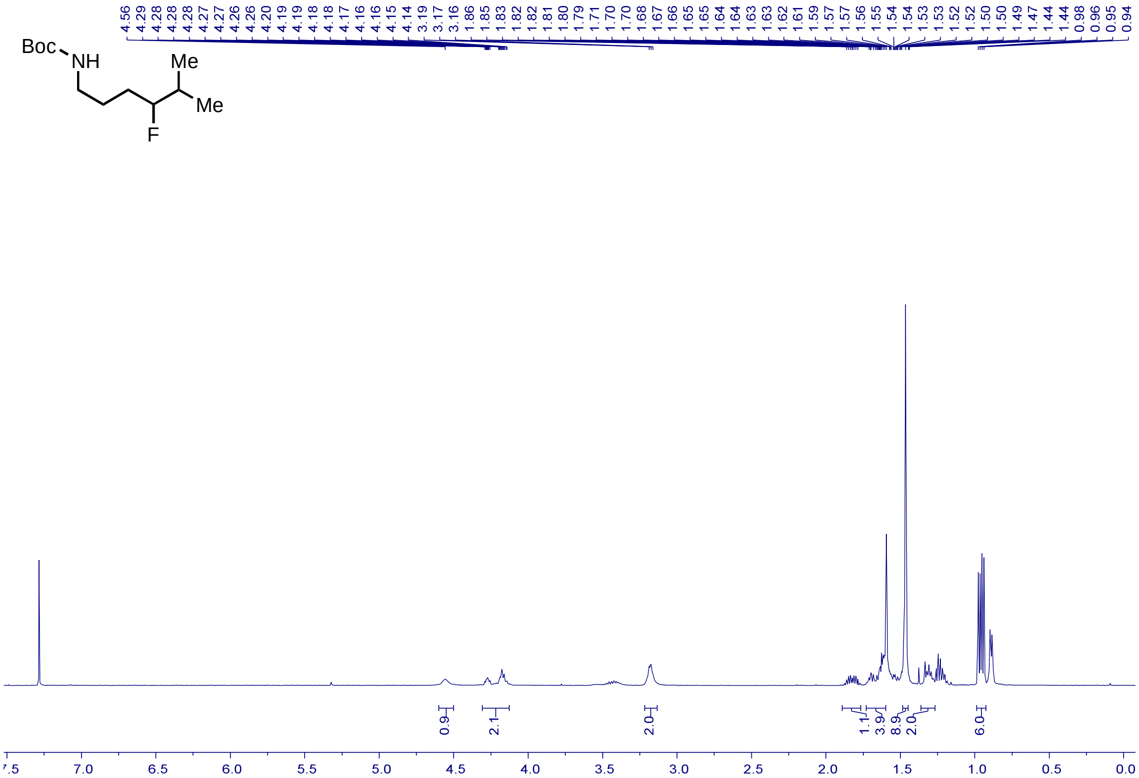

24 ^{13}C NMR (101 MHz, $CDCl_3$)

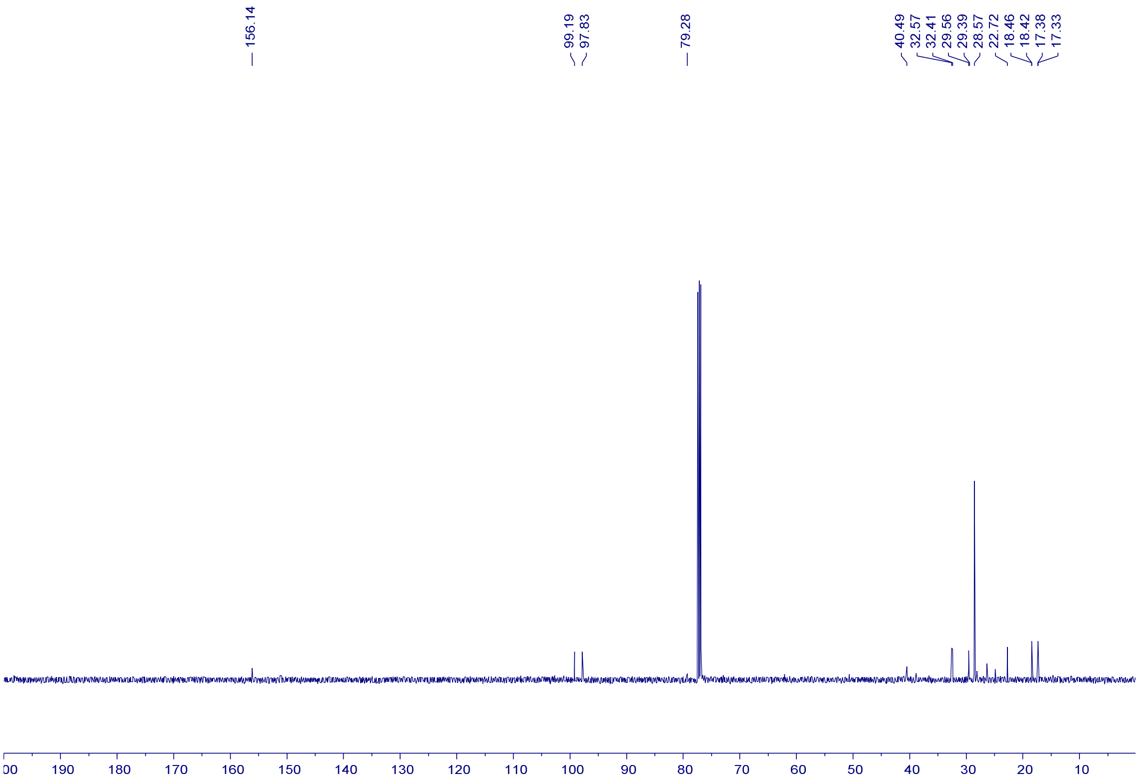

24 ^{19}F NMR (376 MHz, $CDCl_3$)

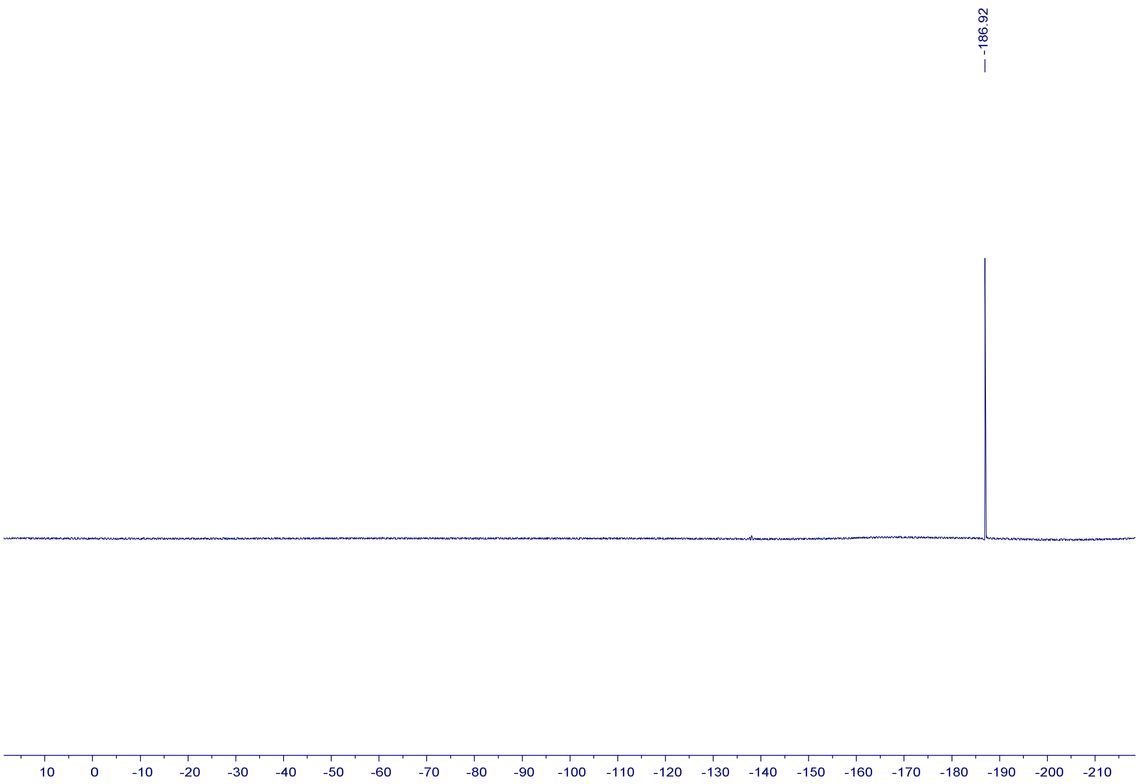

24’ ^{1}H NMR (400 MHz, $CDCl_3$)

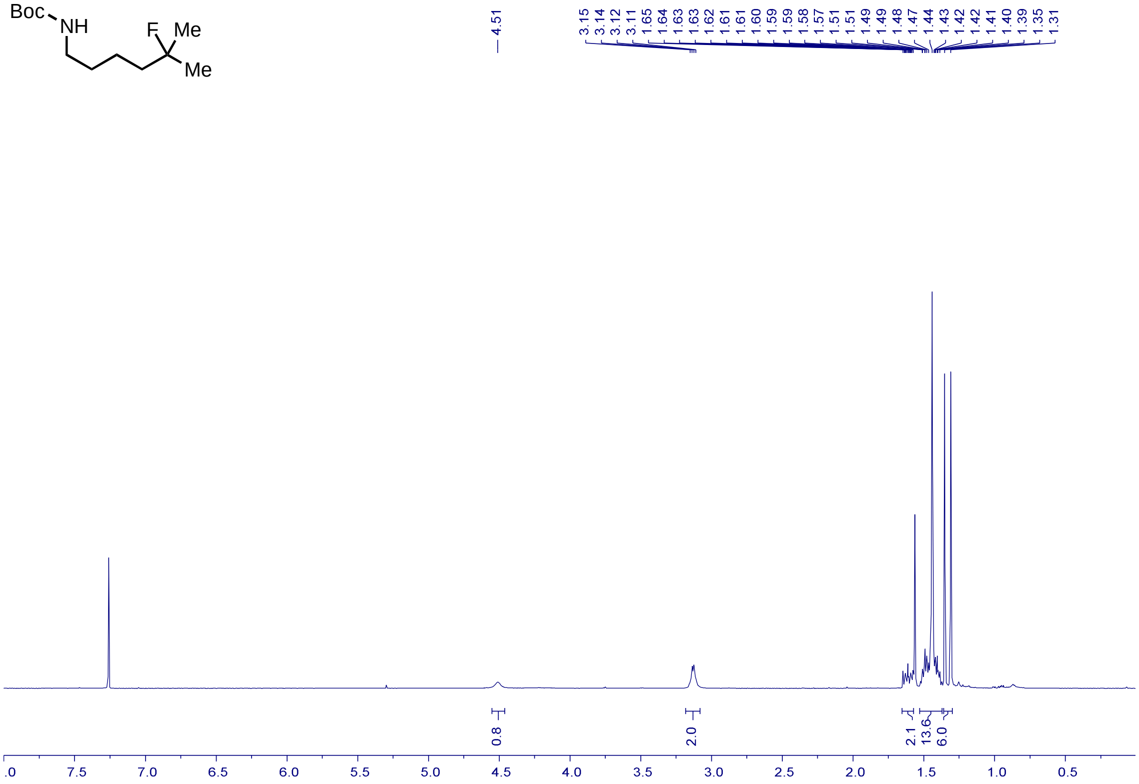

24’ ^{13}C NMR (101 MHz, $CDCl_3$)

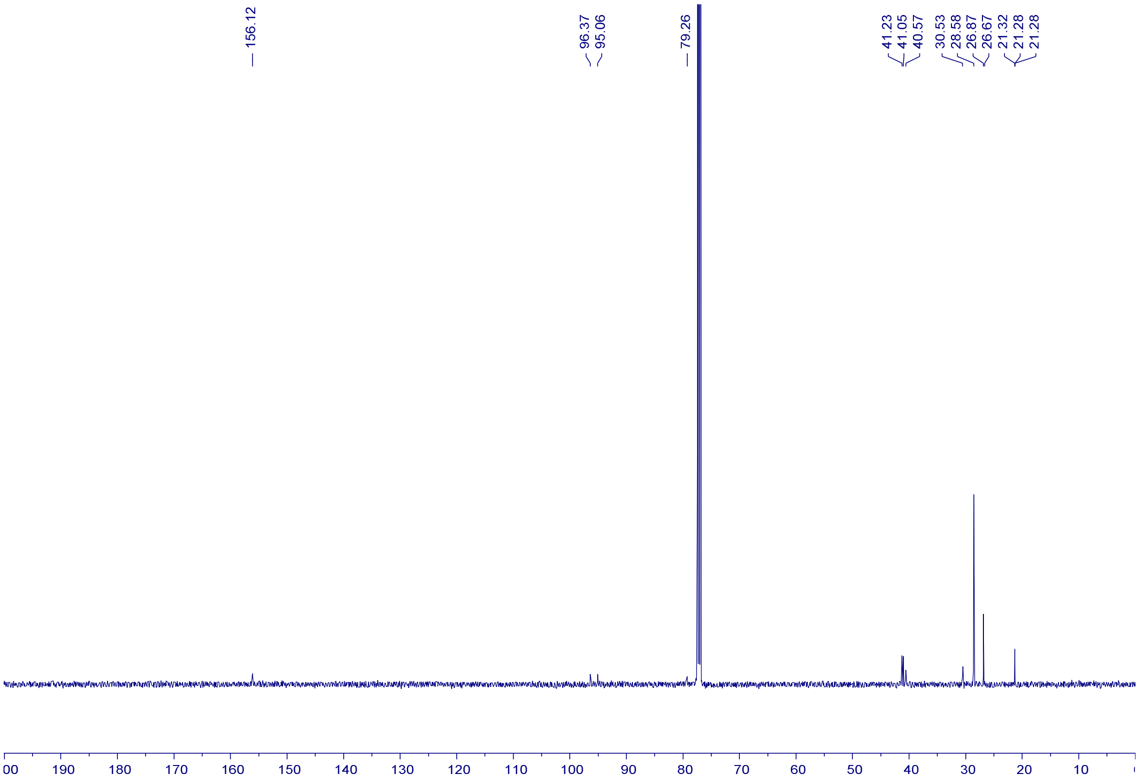

24 ^{19}F NMR (376 MHz, $CDCl_3$)

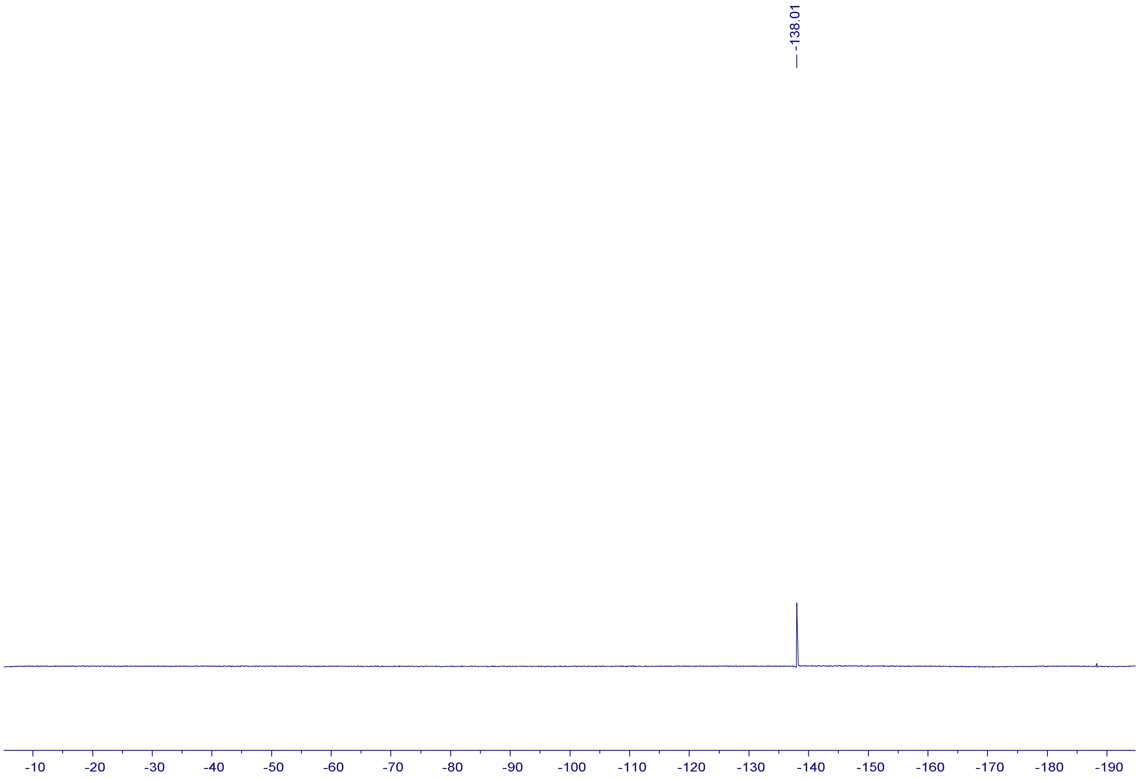

25 ^{1}H NMR (400 MHz, $CDCl_3$)

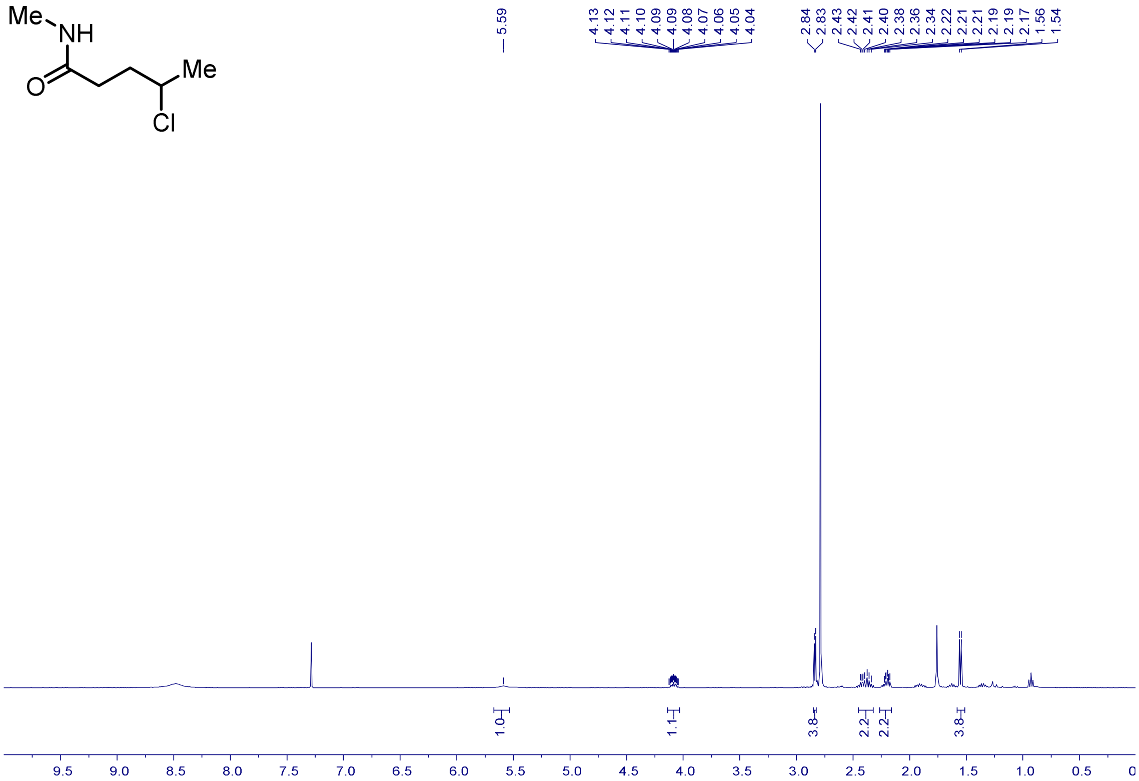

25 ^{13}C NMR (101 MHz, $CDCl_3$)

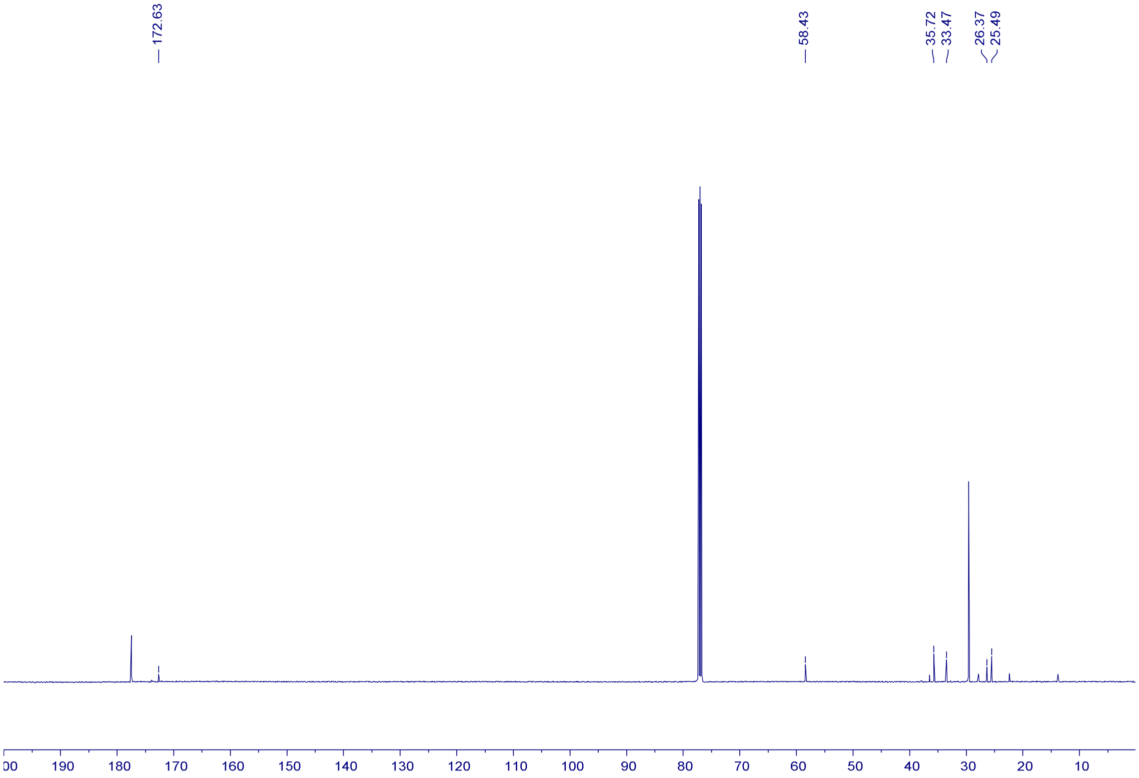

28 ^{1}H NMR (400 MHz, $CDCl_3$)

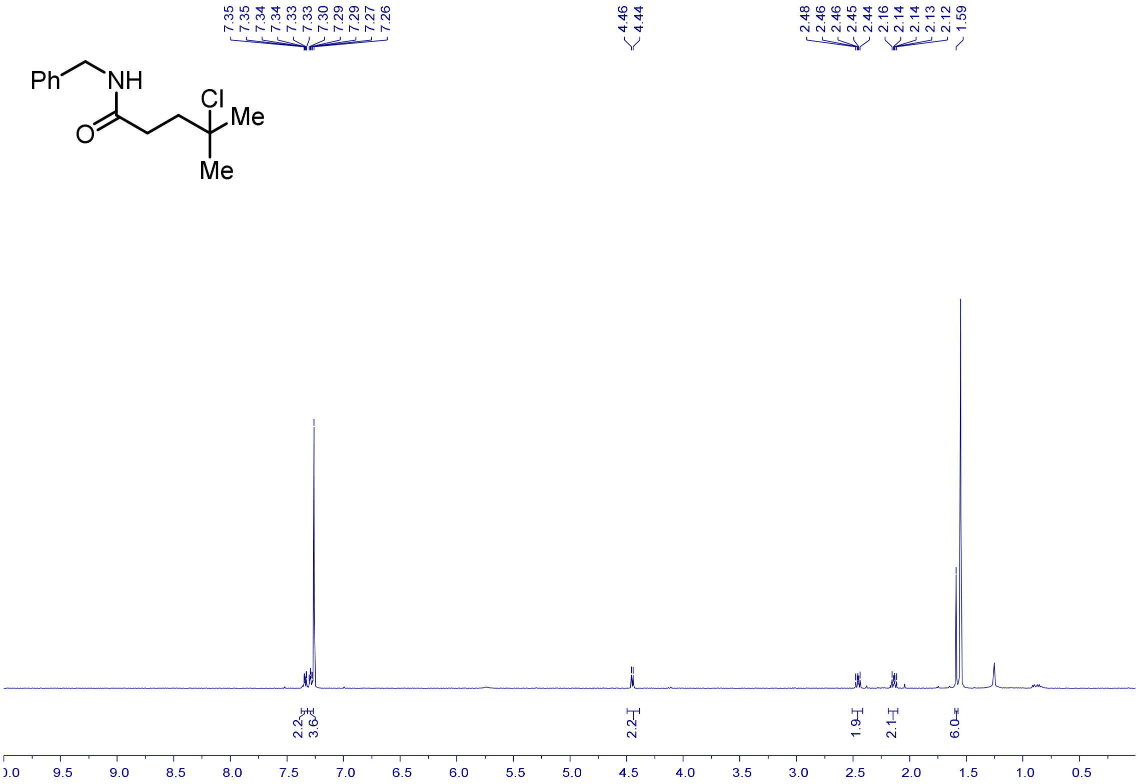

28 ^{13}C NMR (101 MHz, $CDCl_3$)

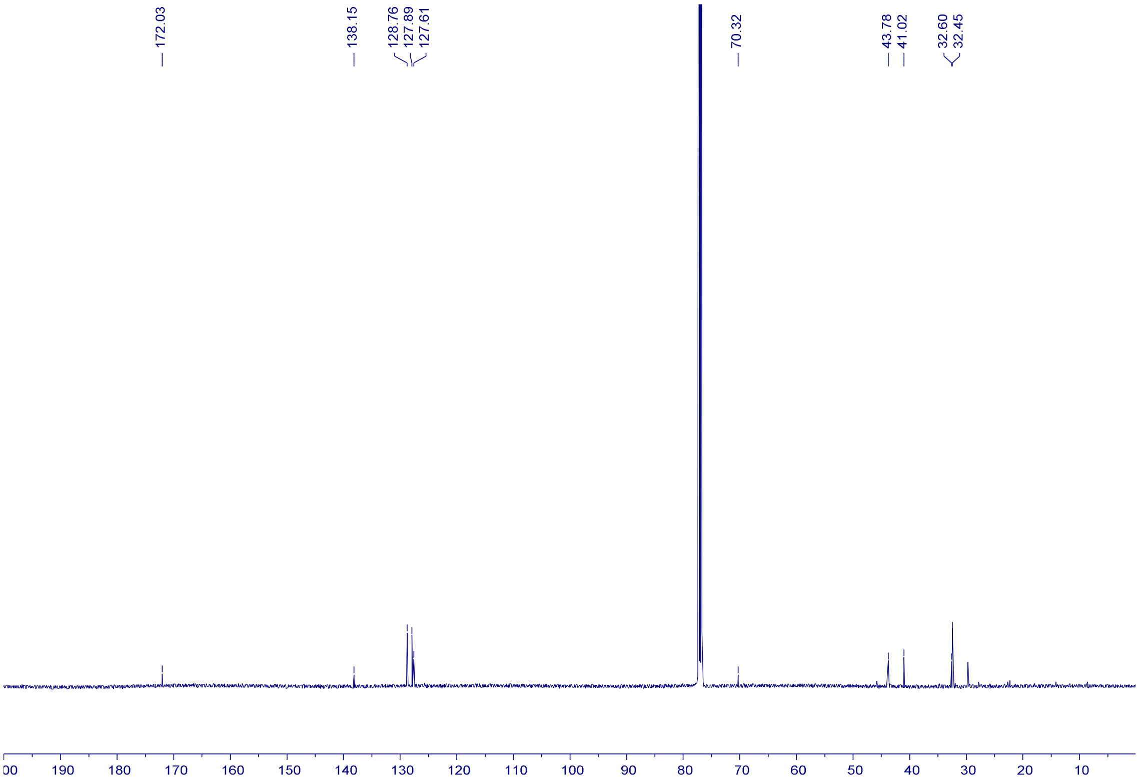

29 ^{1}H NMR (500 MHz, CDCl$_3$)

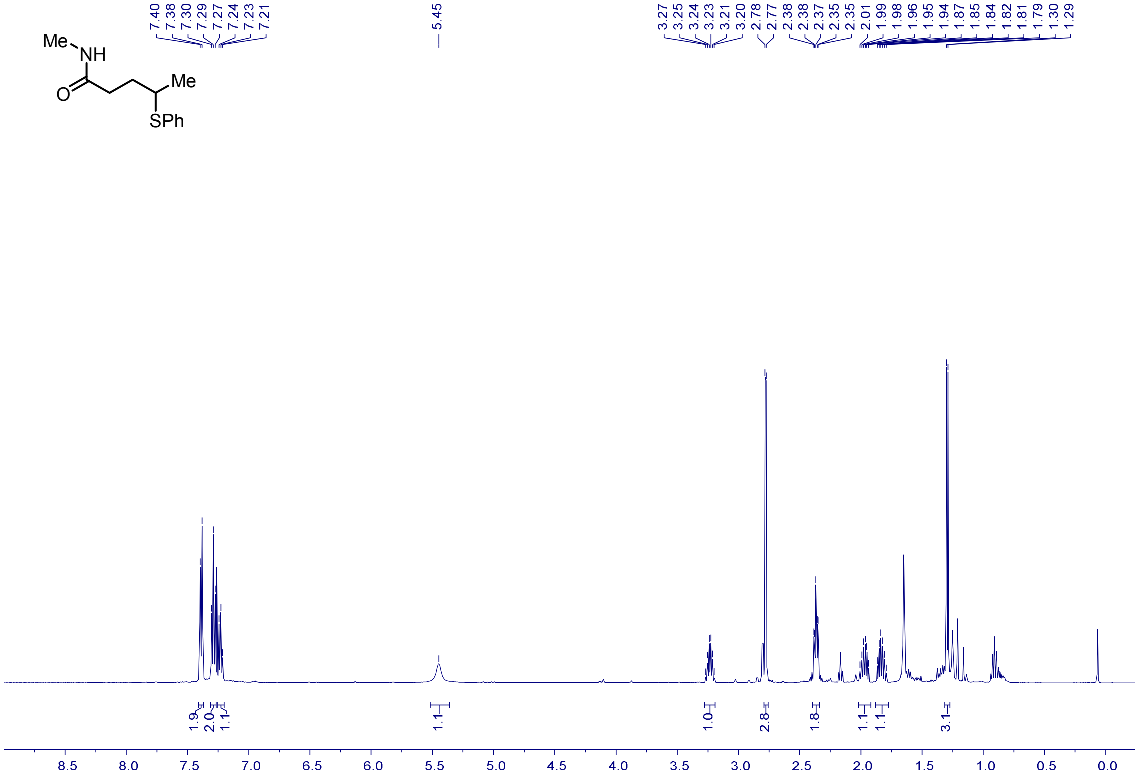

29 ^{13}C NMR (126 MHz, CDCl$_3$)

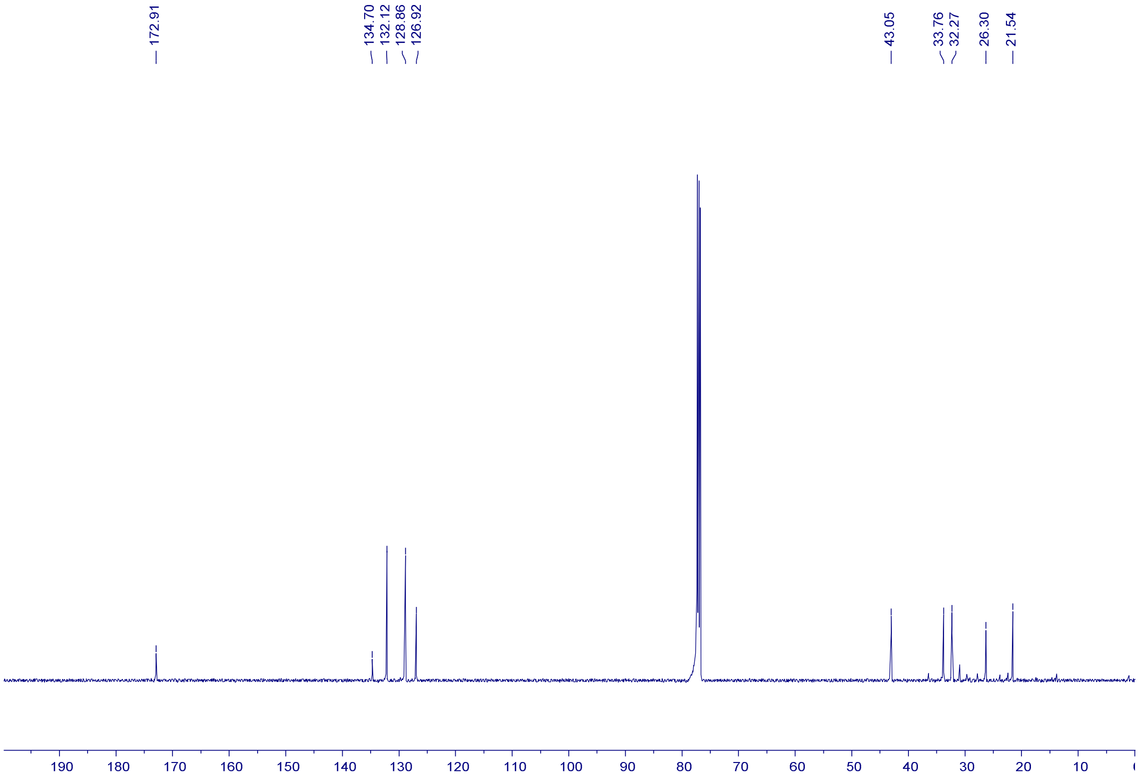

30 ^{1}H NMR (500 MHz, $CDCl_3$)

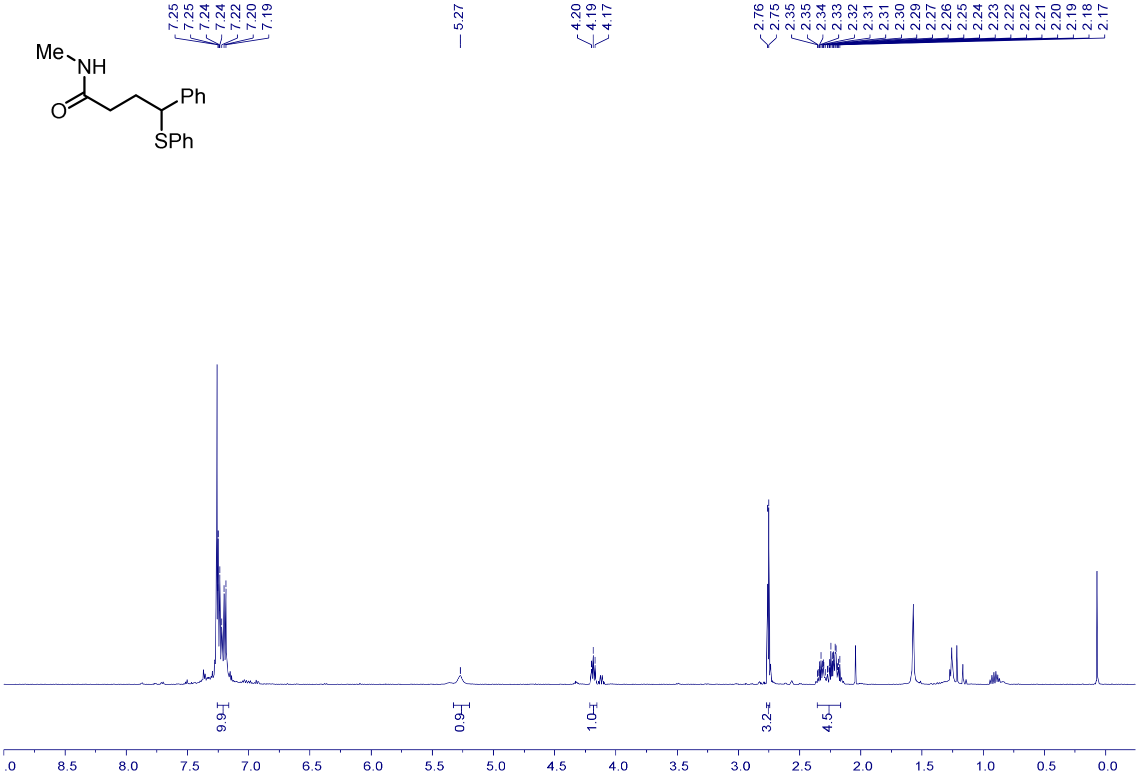

30 ^{13}C NMR (126 MHz, $CDCl_3$)

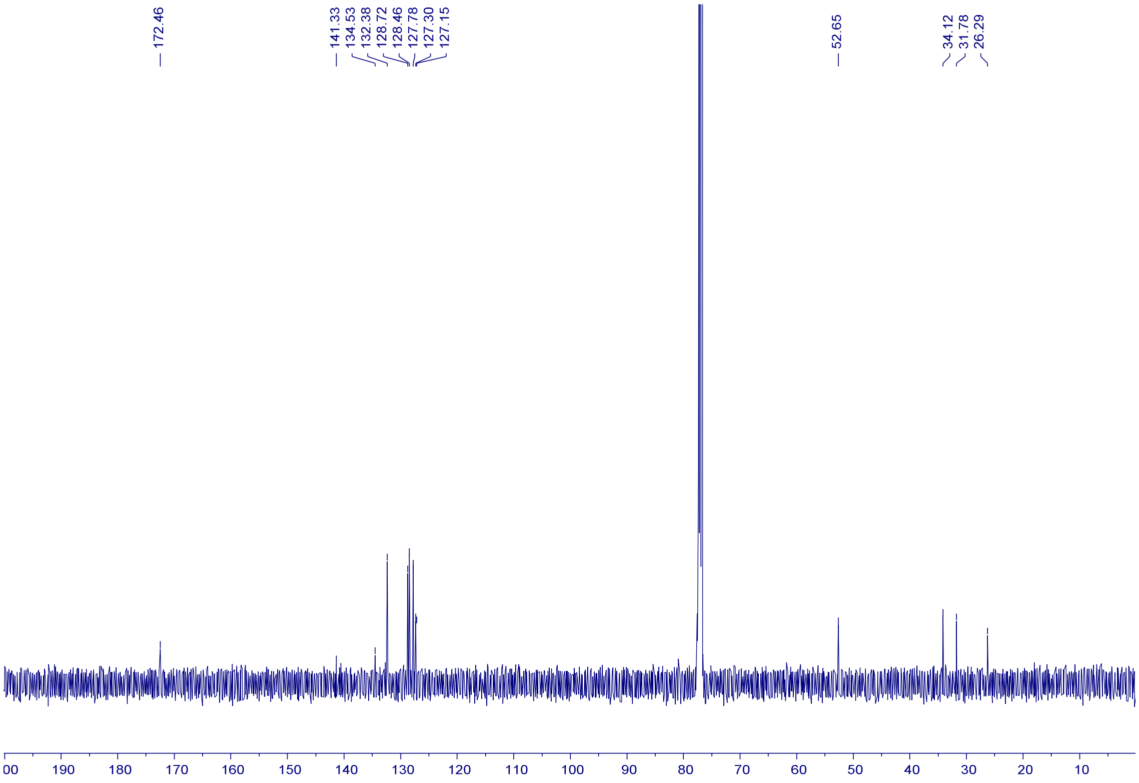

32 ^{1}H NMR (400 MHz, $CDCl_3$)

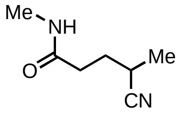

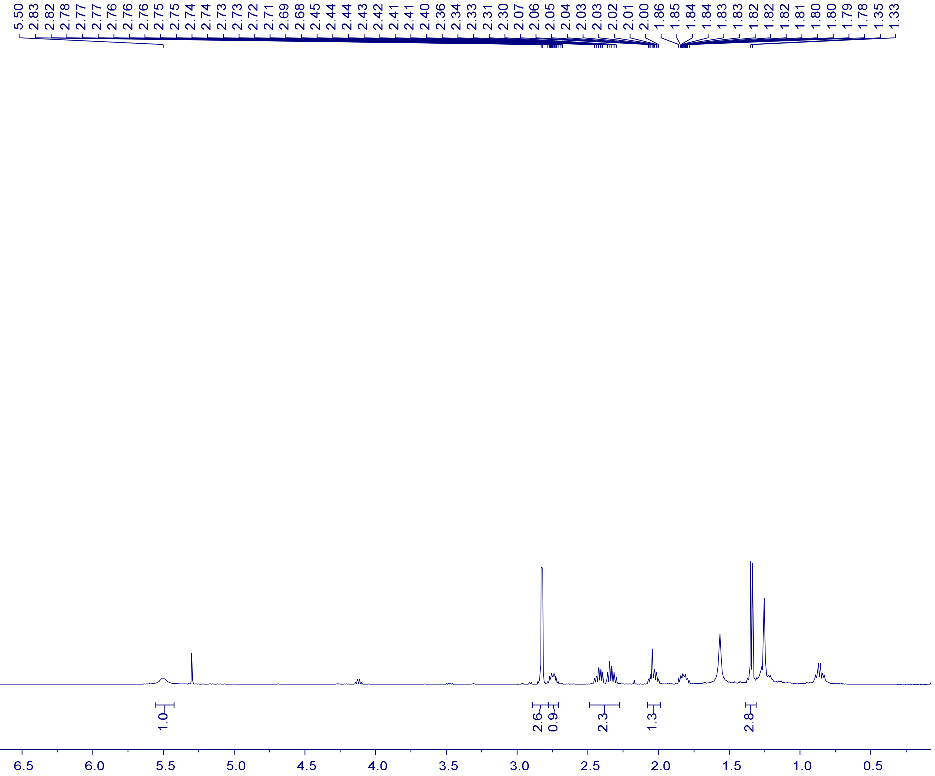

32 ^{13}C NMR (101 MHz, $CDCl_3$)

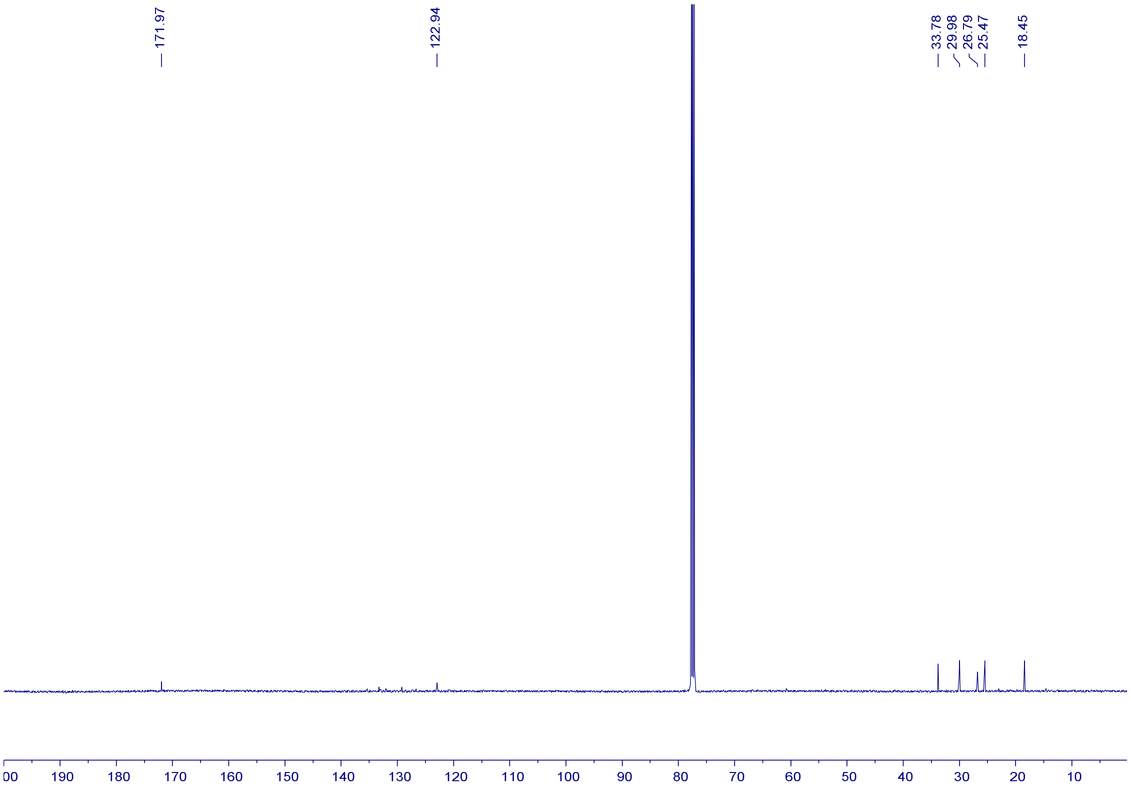

34 ^{1}H NMR (400 MHz, $CDCl_3$)

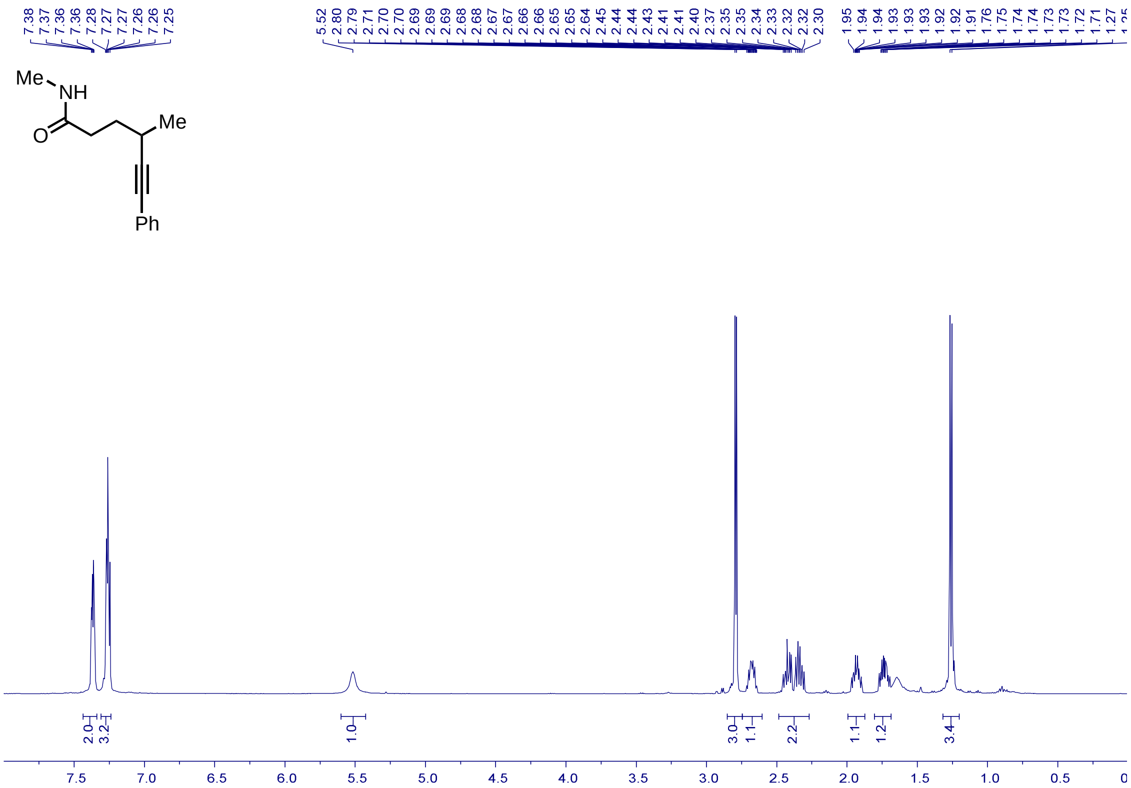

34 ^{13}C NMR (101 MHz, $CDCl_3$)

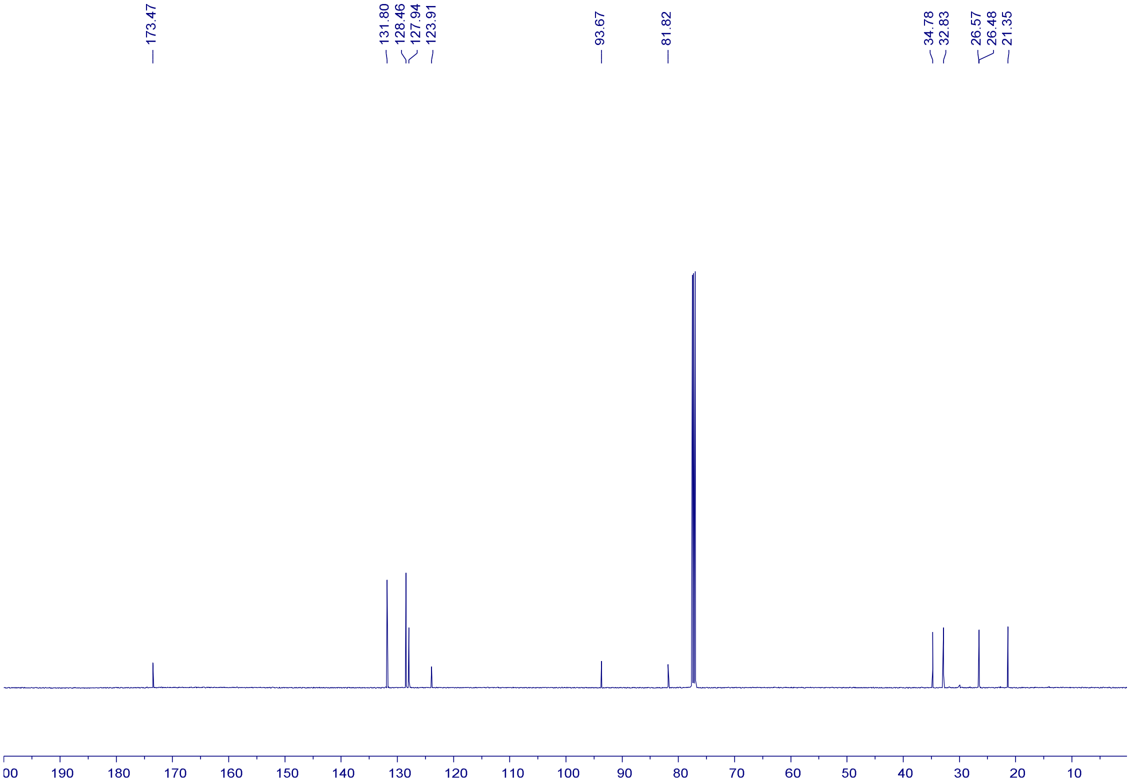

36 ^{1}H NMR (400 MHz, $CDCl_3$)

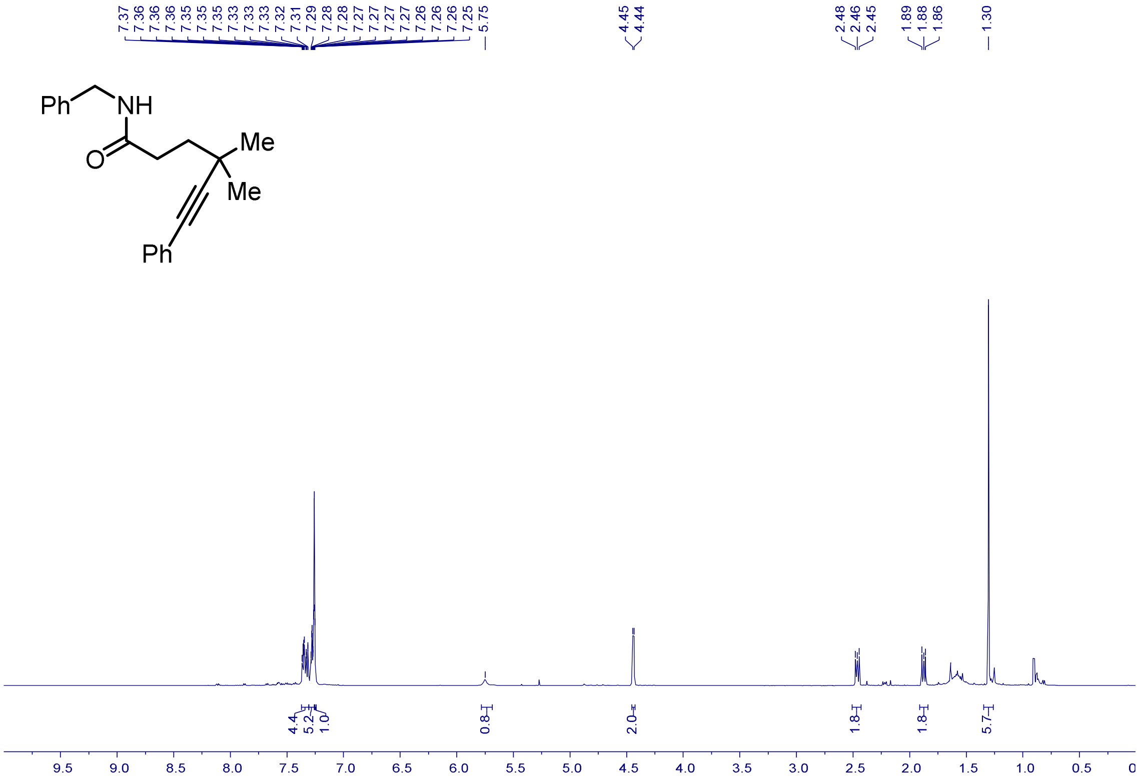

36 ^{13}C NMR (101 MHz, $CDCl_3$)

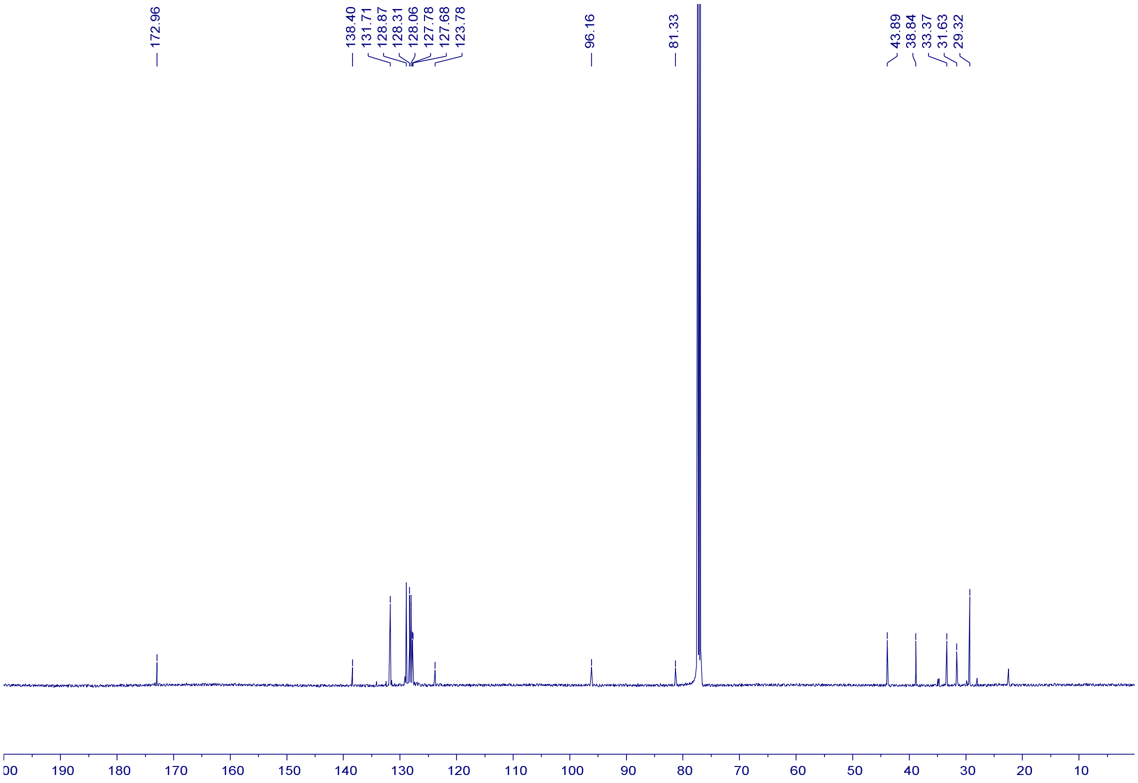

38 ^{1}H NMR (400 MHz, $CDCl_3$)

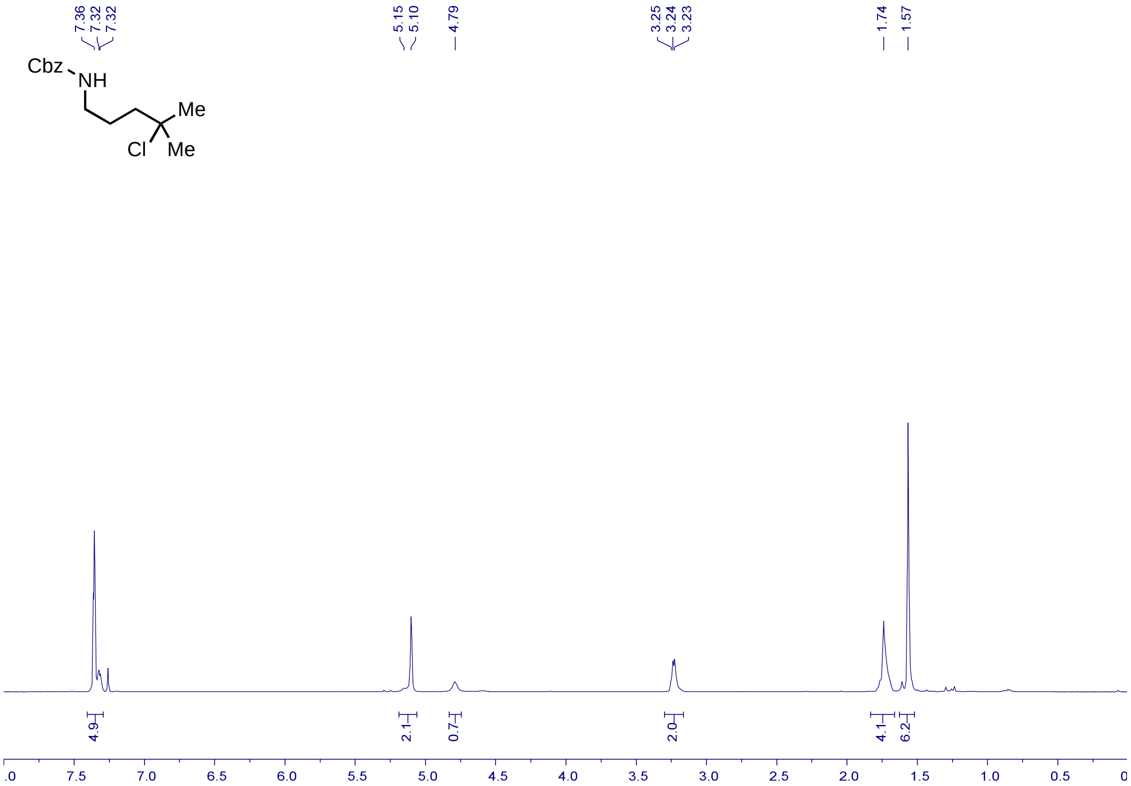

38 ^{13}C NMR (101 MHz, $CDCl_3$)

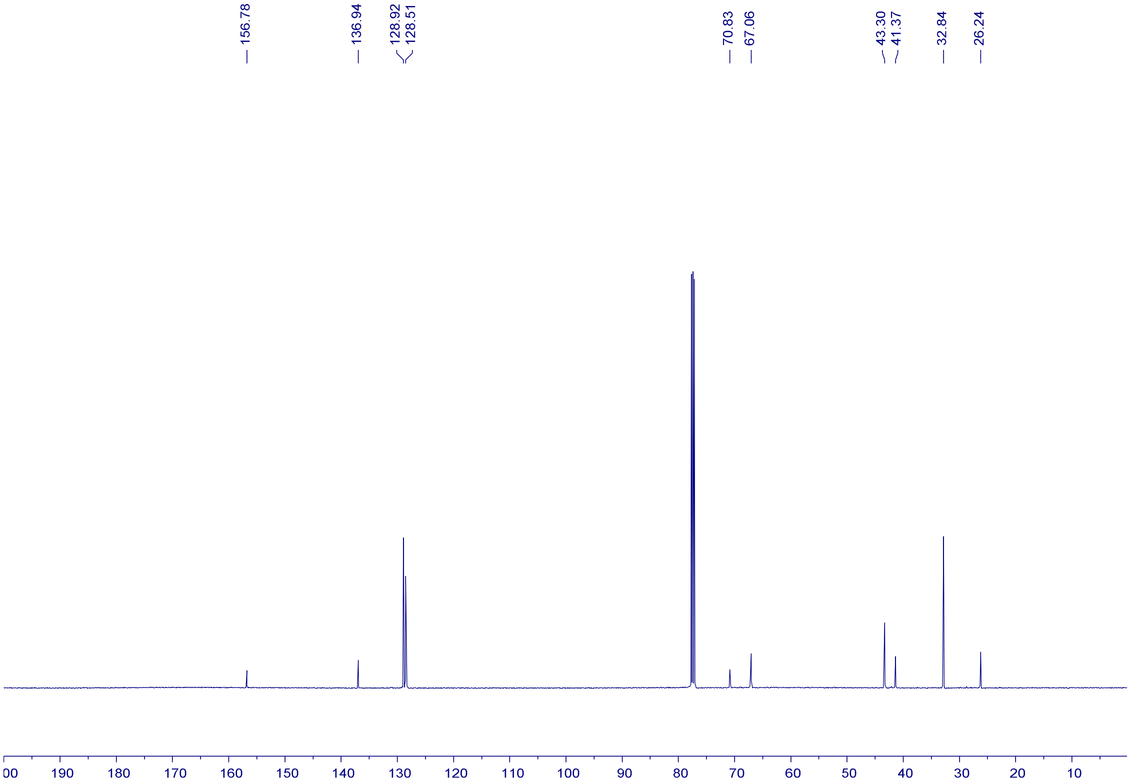

39 ^{1}H NMR (400 MHz, $CDCl_3$)

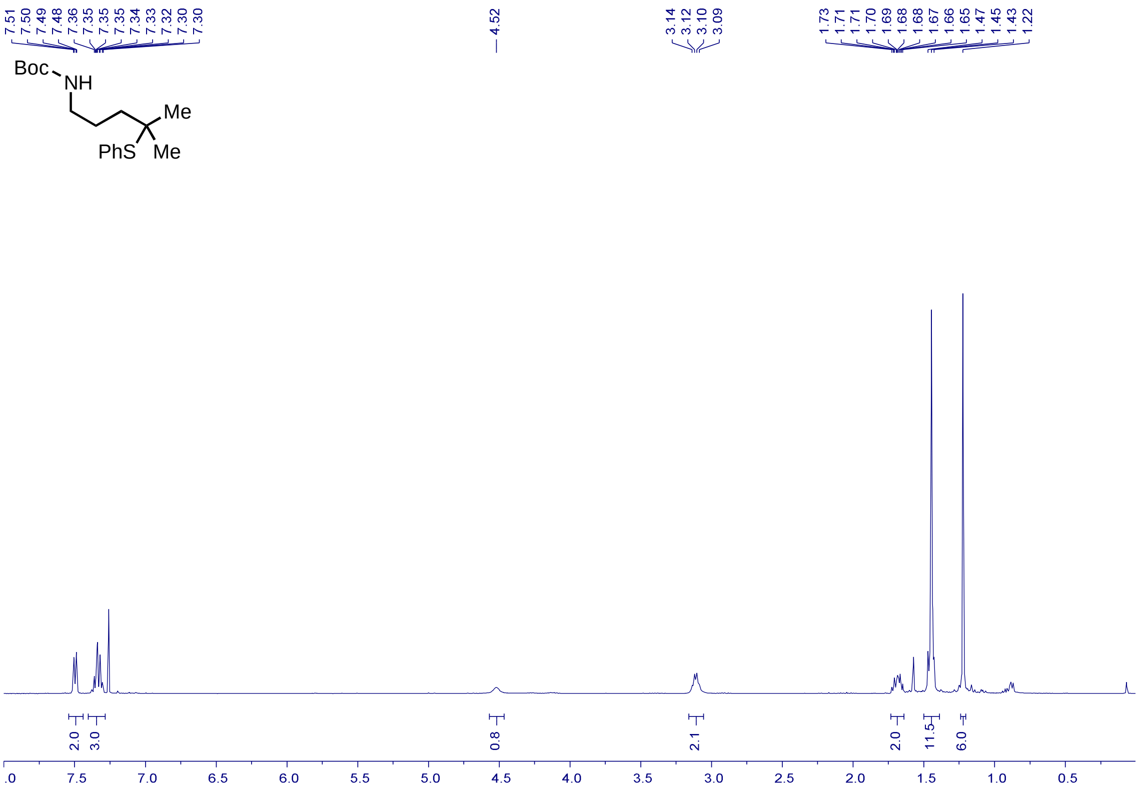

39 ^{13}C NMR (101 MHz, $CDCl_3$)

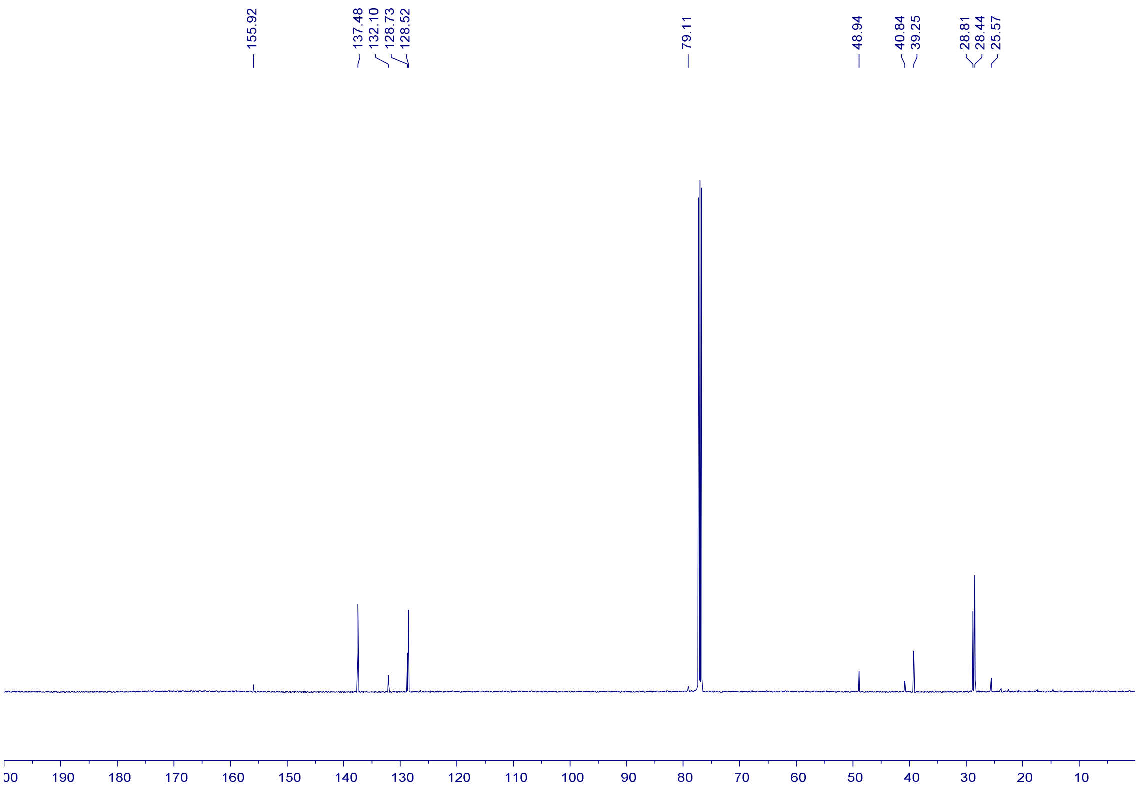

41 ^{1}H NMR (400 MHz, $CDCl_3$)

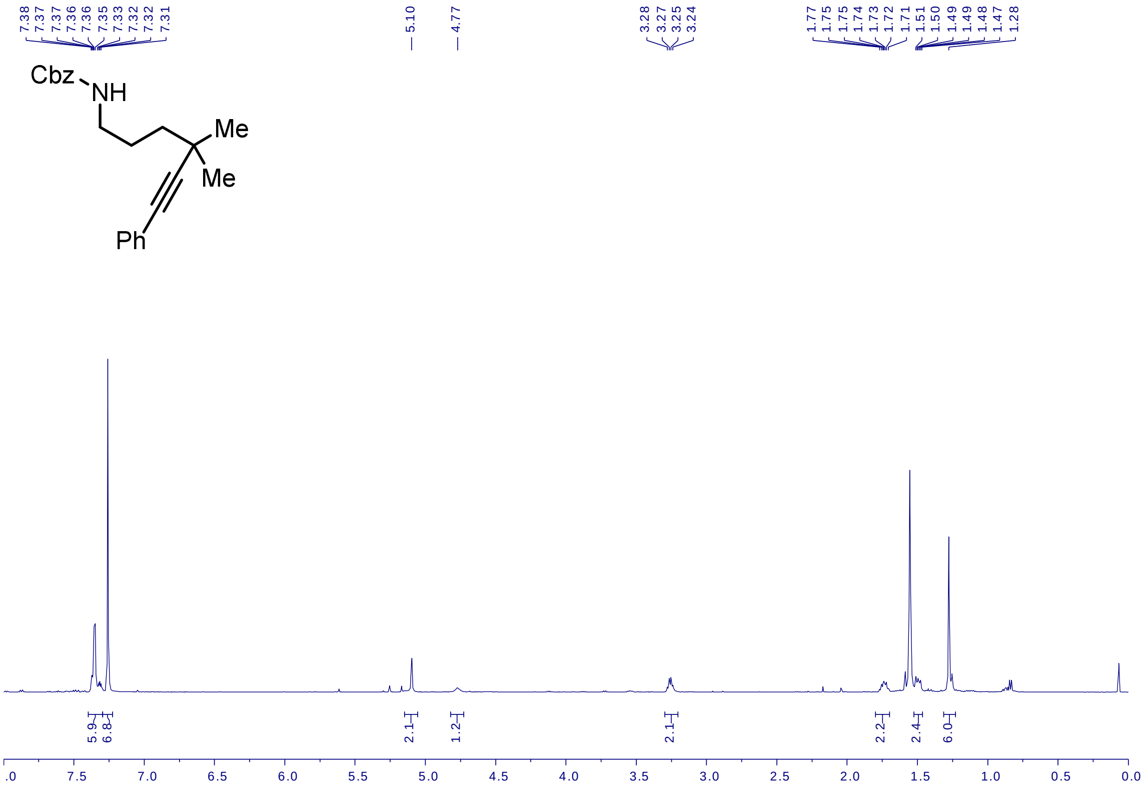

41 ^{13}C NMR (101 MHz, $CDCl_3$)

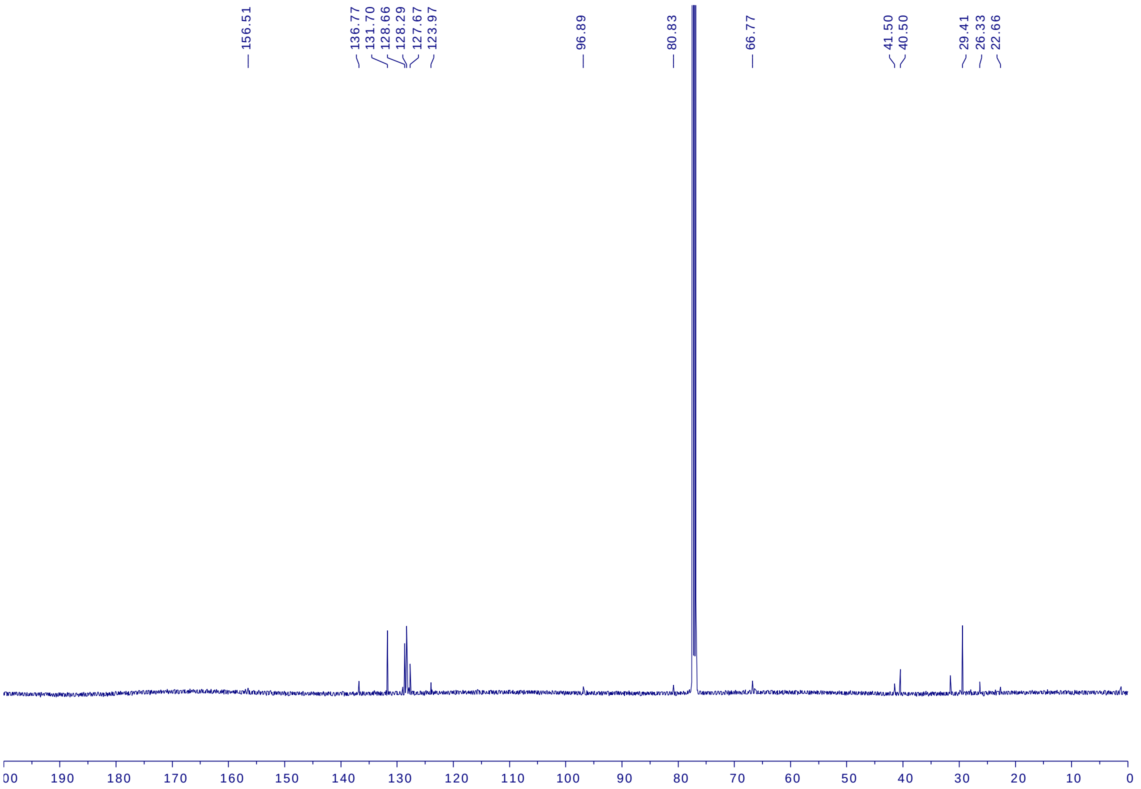

42 ^{1}H NMR (400 MHz, $CDCl_3$)

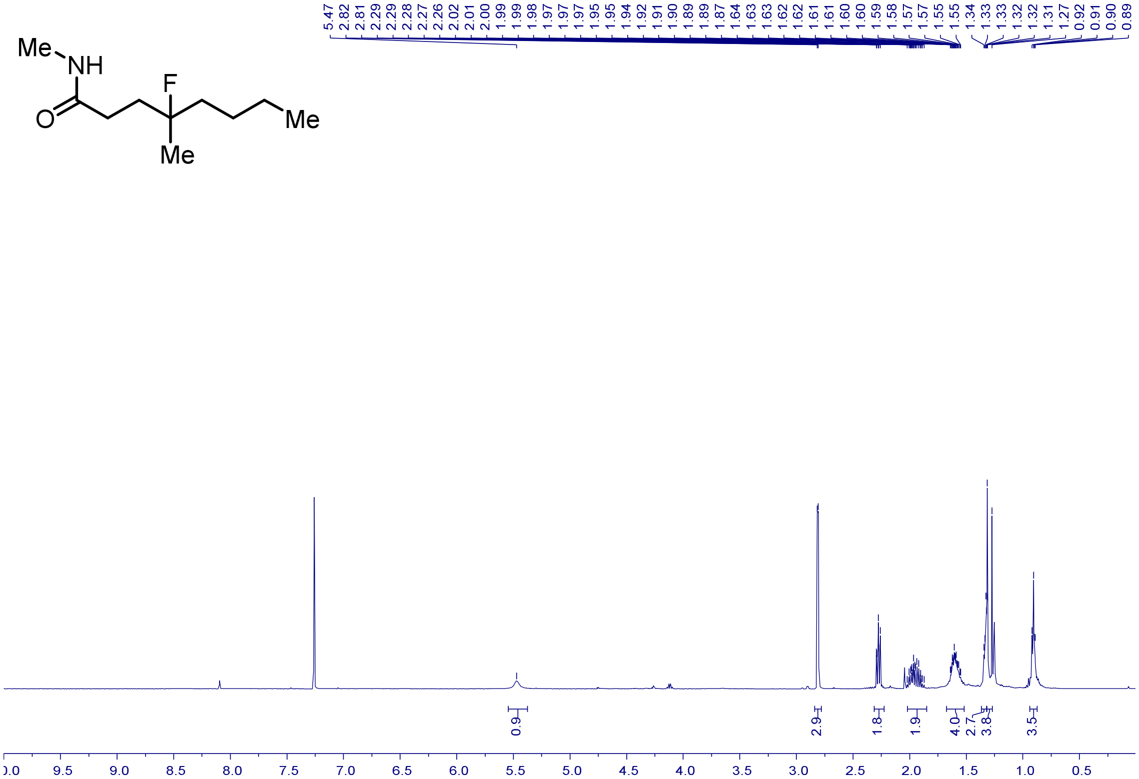

42 ^{13}C NMR (101 MHz, $CDCl_3$)

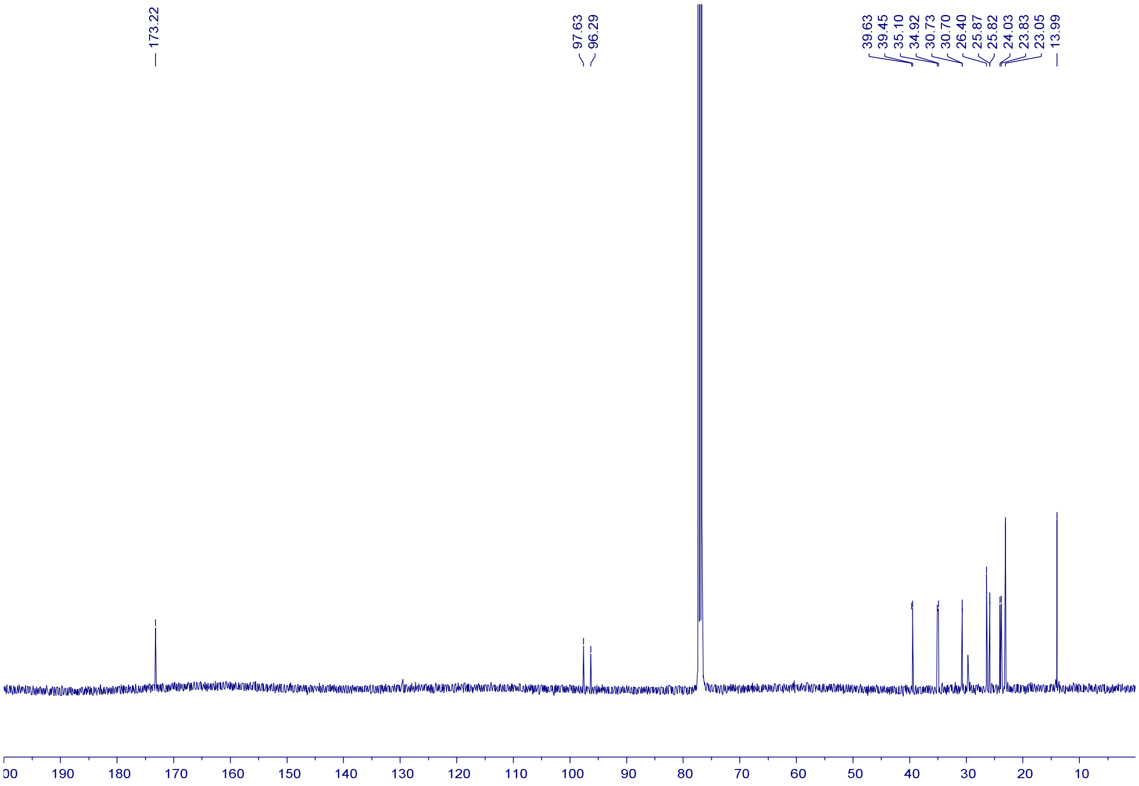

42 ^{19}F NMR (376 MHz, $CDCl_3$)

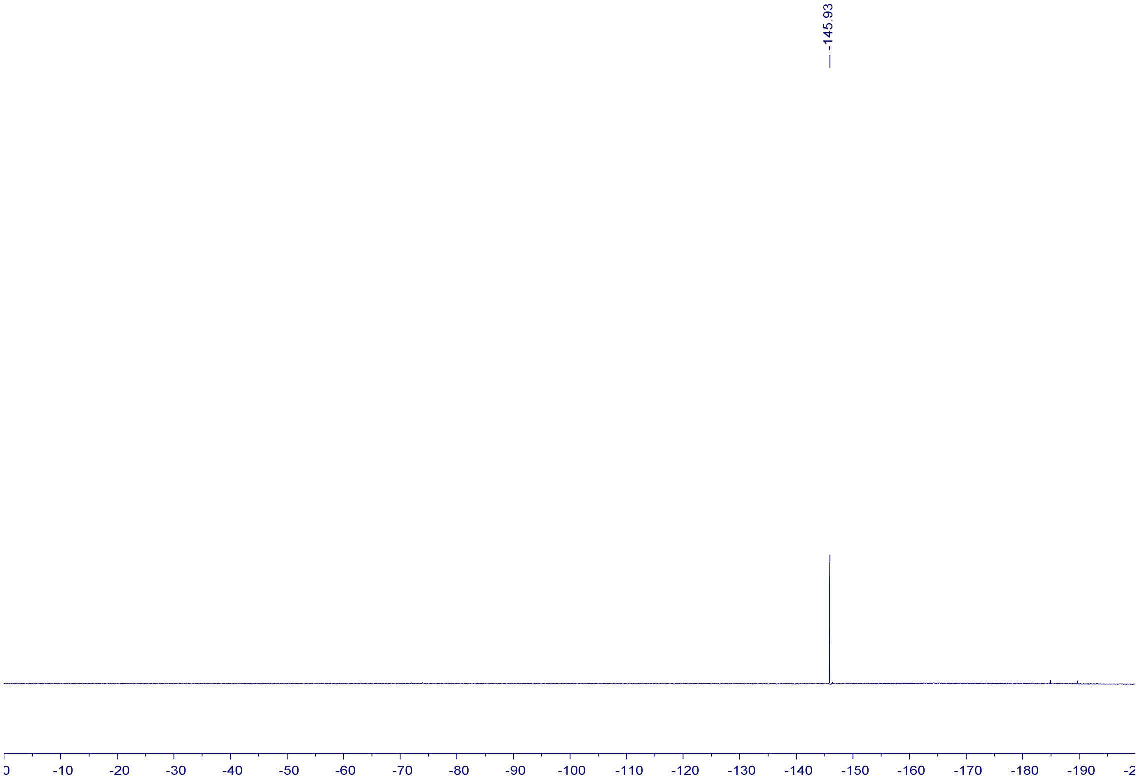

43 ^{1}H NMR (400 MHz, $CDCl_3$)

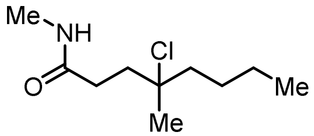

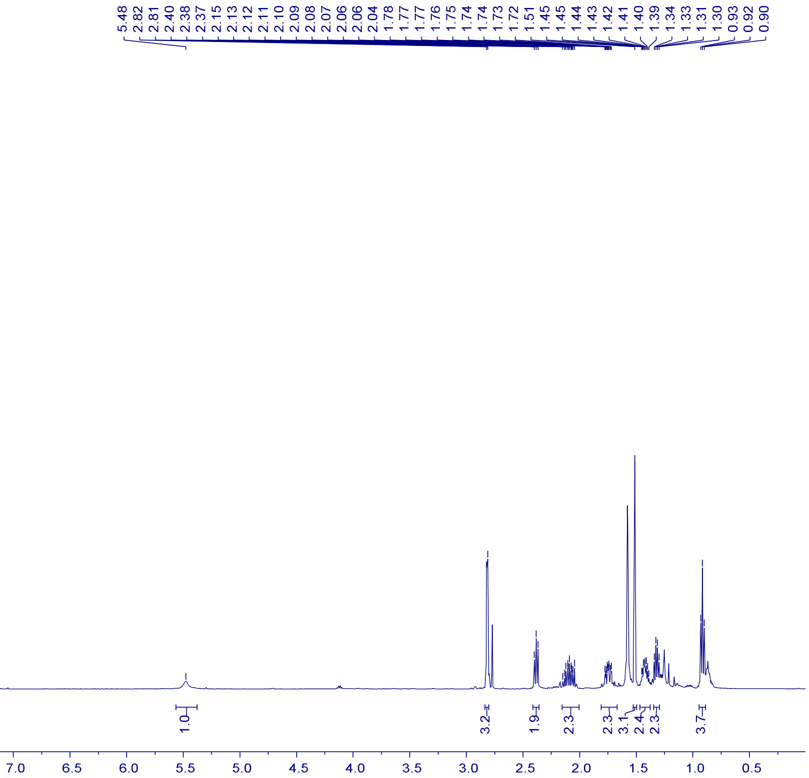

43 ^{13}C NMR (101 MHz, $CDCl_3$)

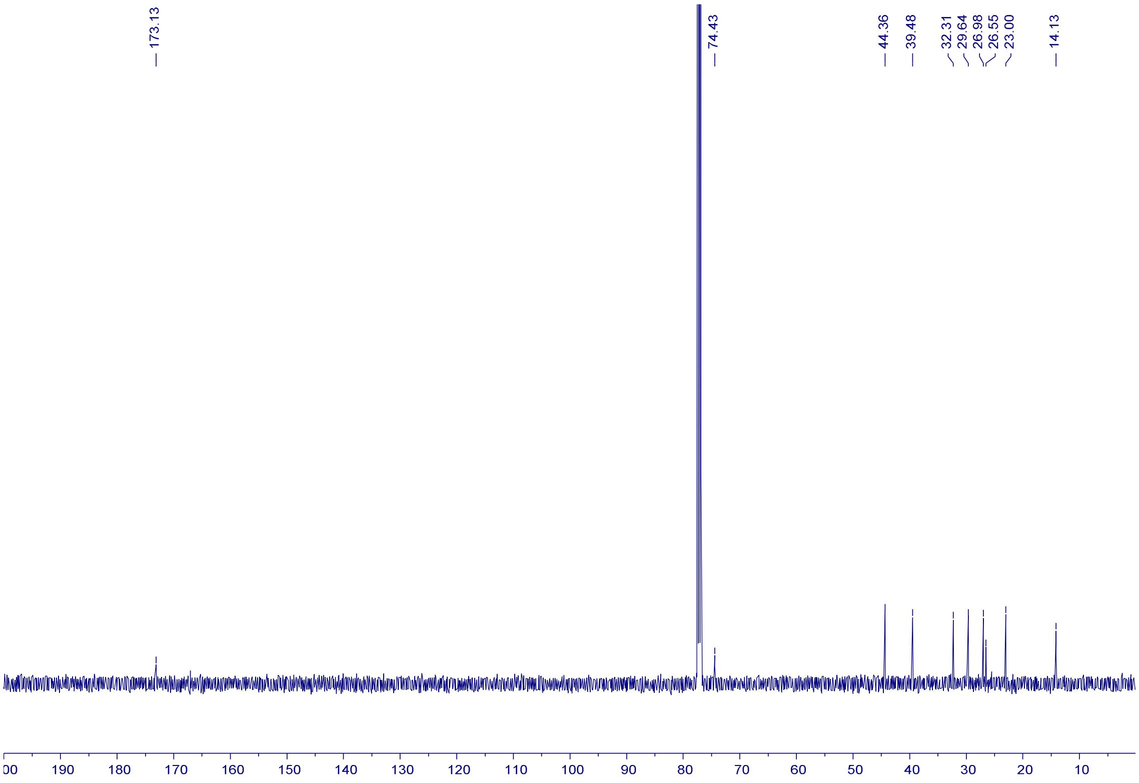

44 ^{1}H NMR (400 MHz, $CDCl_3$)

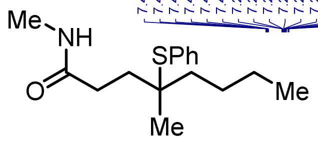

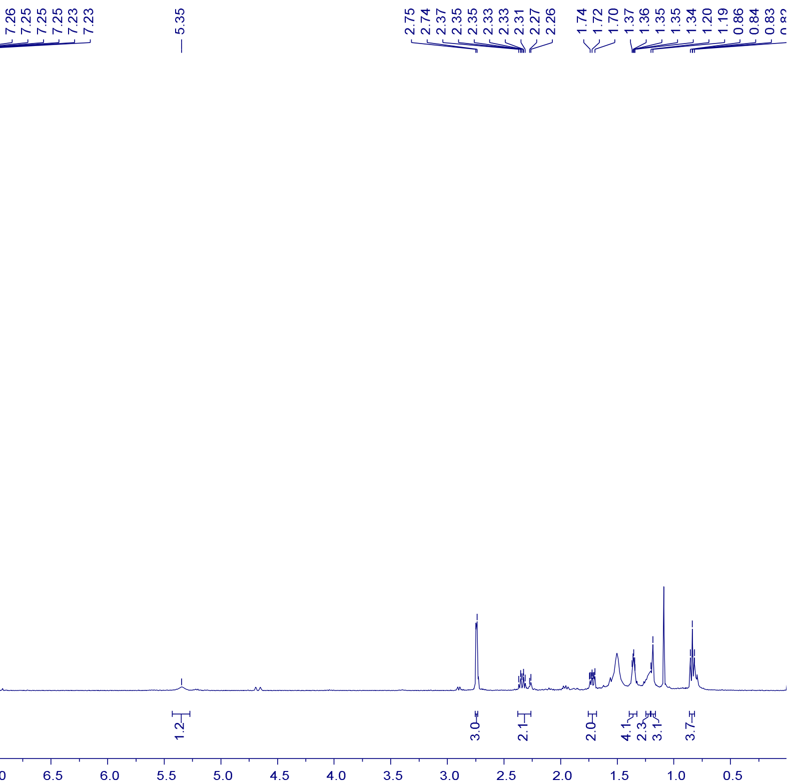

44 ^{13}C NMR (101 MHz, $CDCl_3$)

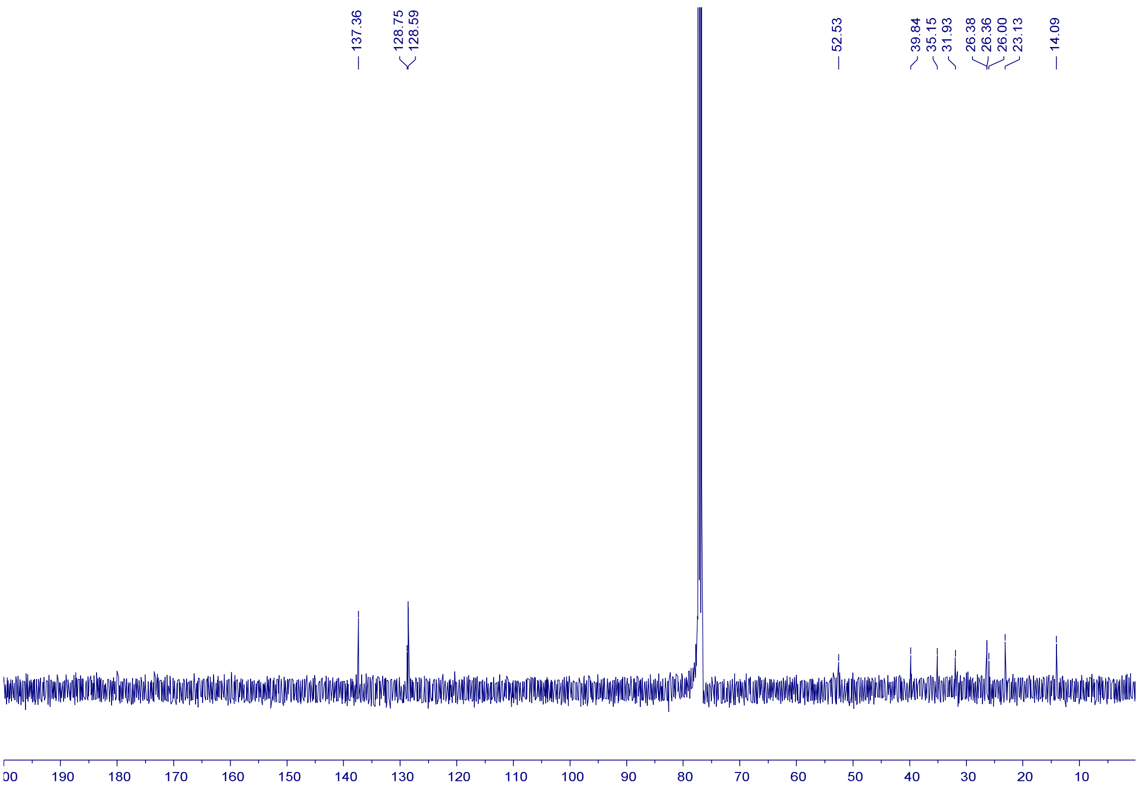

45 ^{1}H NMR (400 MHz, $CDCl_3$)

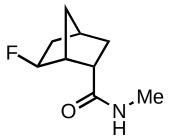

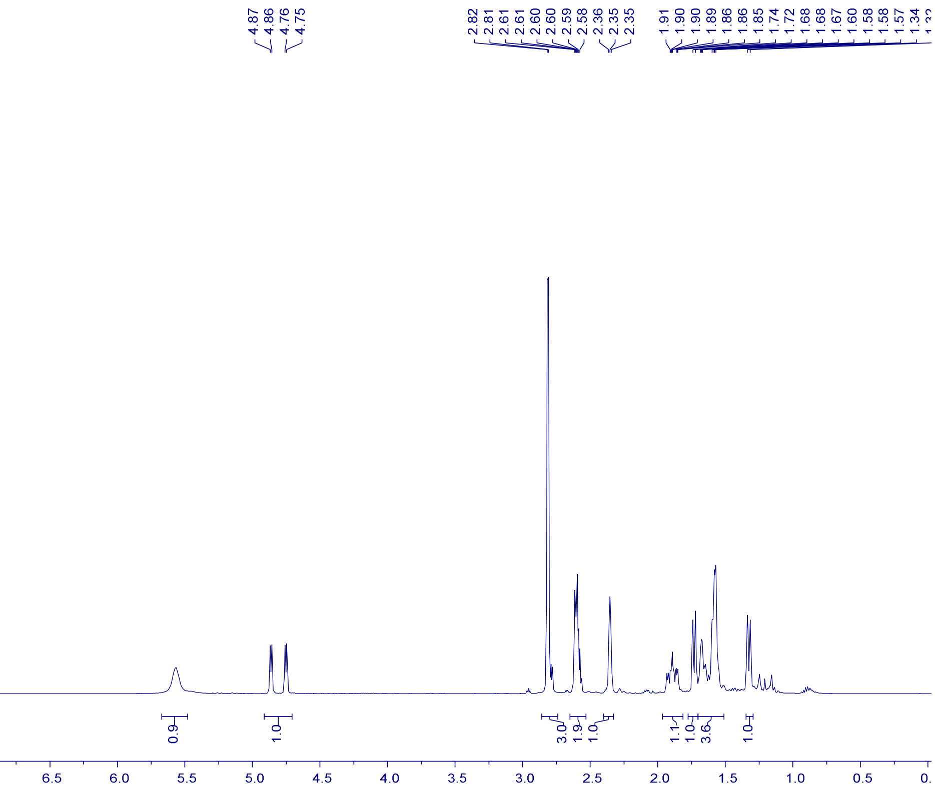

45 ^{13}C NMR (101 MHz, $CDCl_3$)

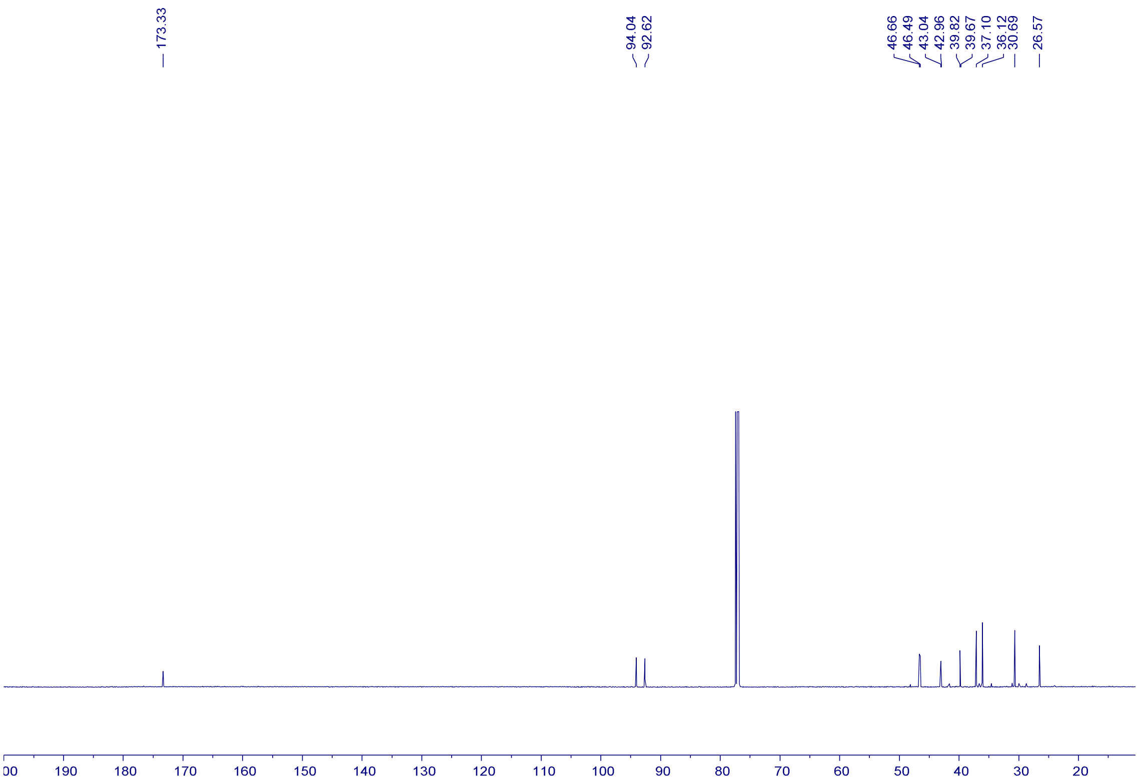

45 ^{19}F NMR (376 MHz, $CDCl_3$)

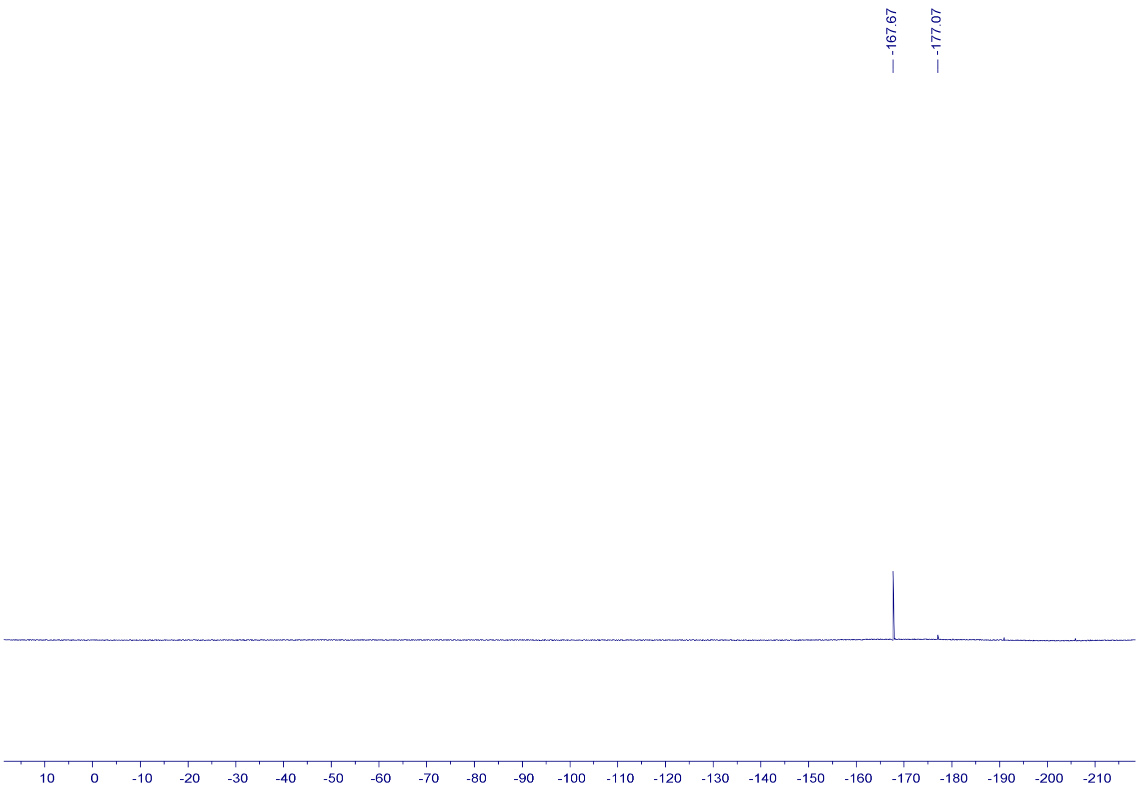

46 ^{1}H NMR (400 MHz, $CDCl_3$)

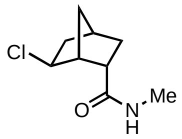

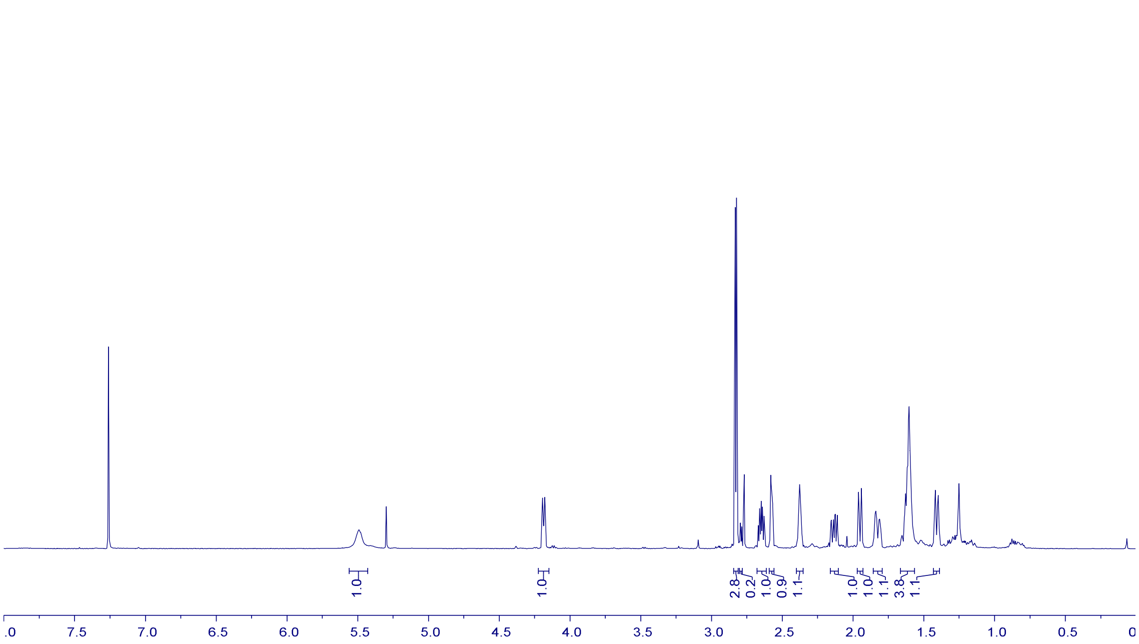

46 ^{13}C NMR (101 MHz, $CDCl_3$)

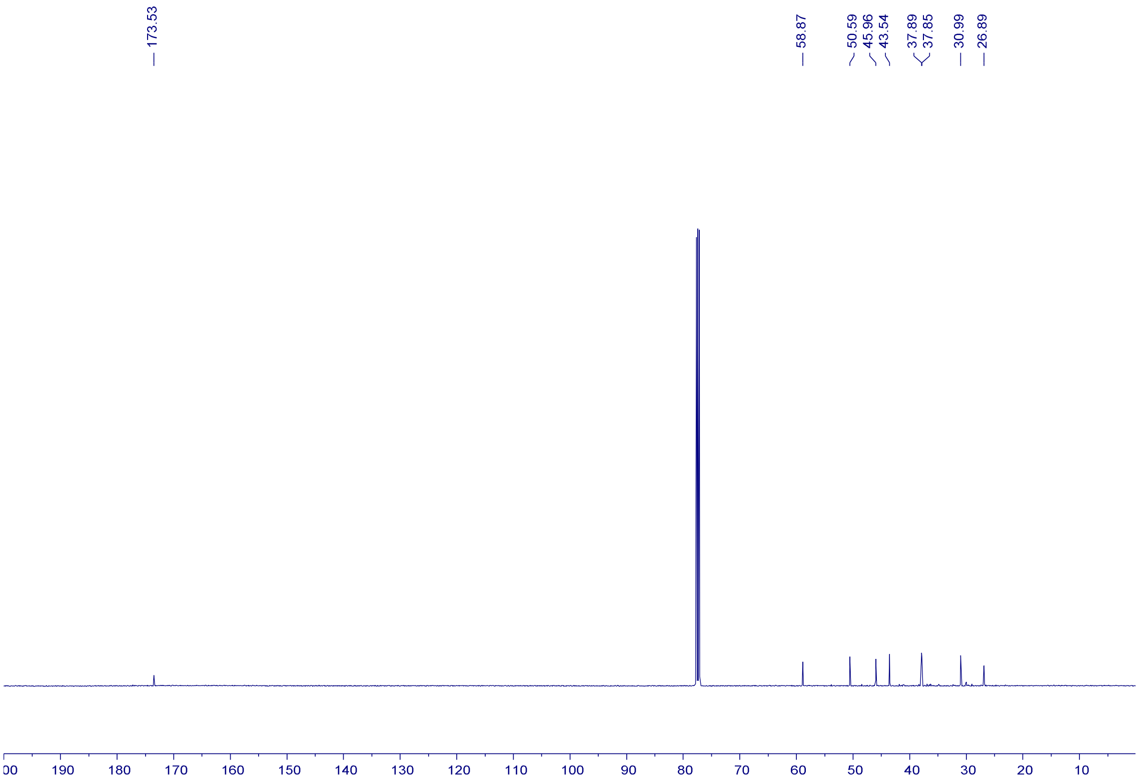

47 ^{1}H NMR (400 MHz, $CDCl_3$)

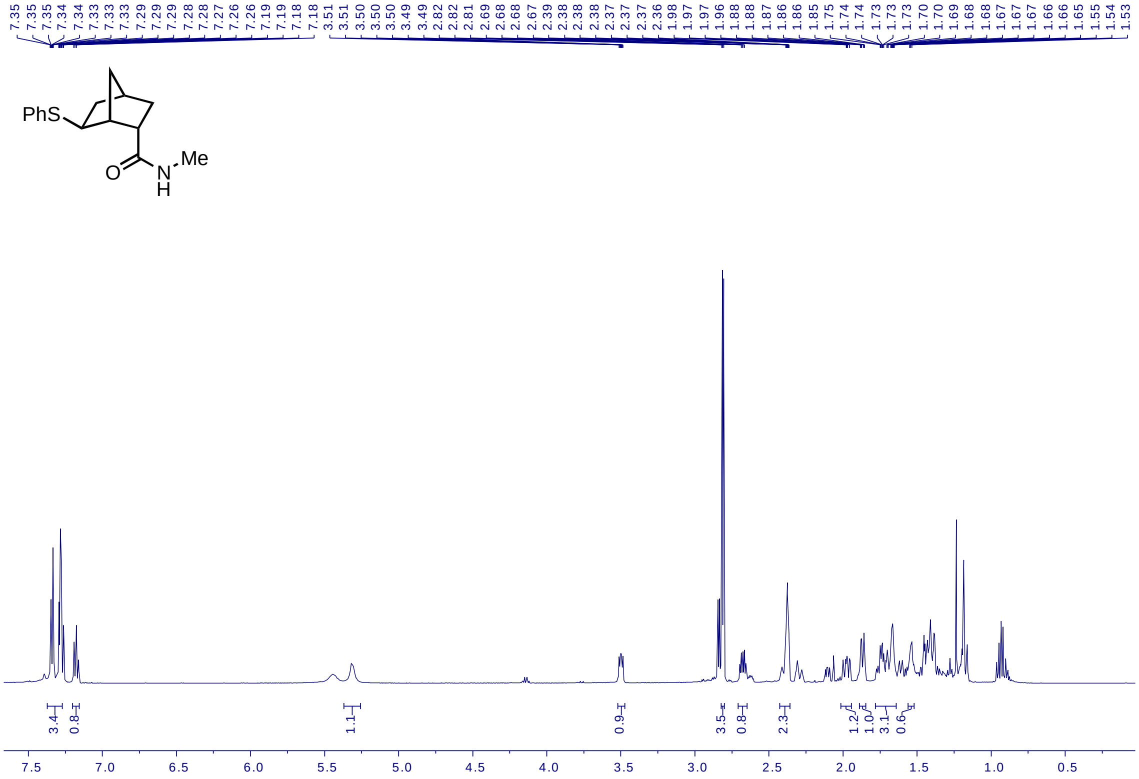

47 ^{13}C NMR (101 MHz, $CDCl_3$)

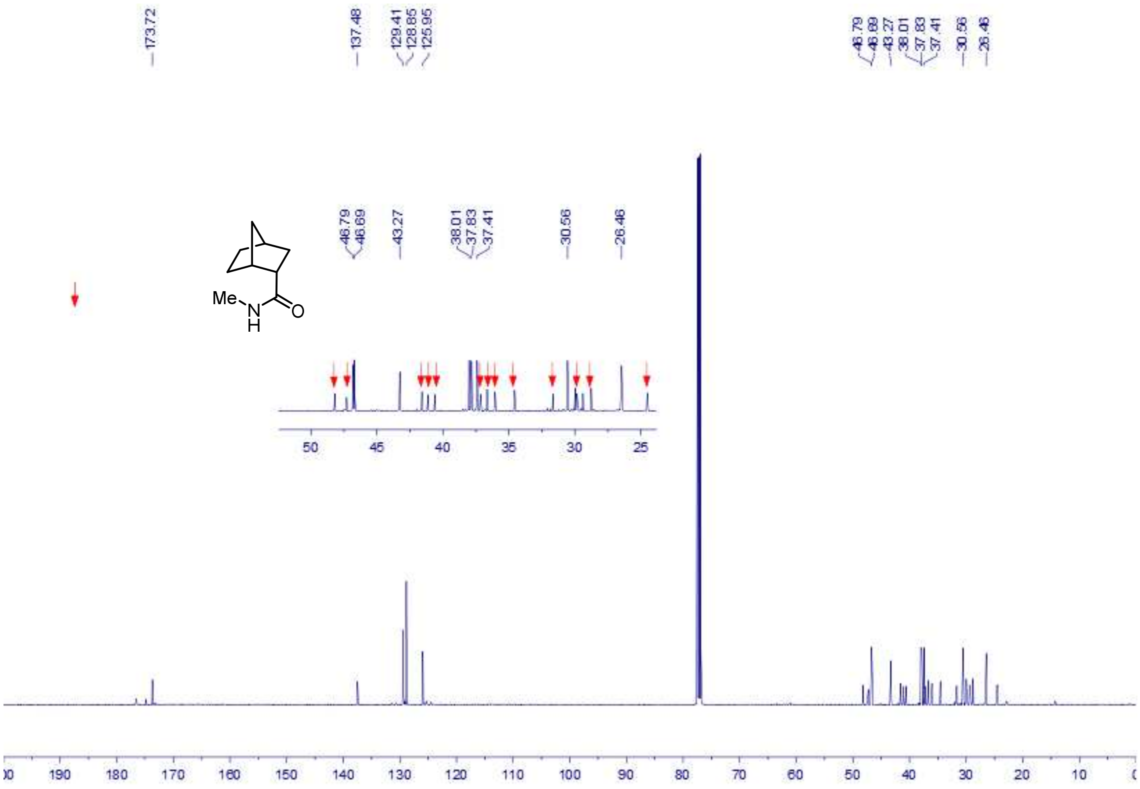

48 ^{1}H NMR (400 MHz, $CDCl_3$)

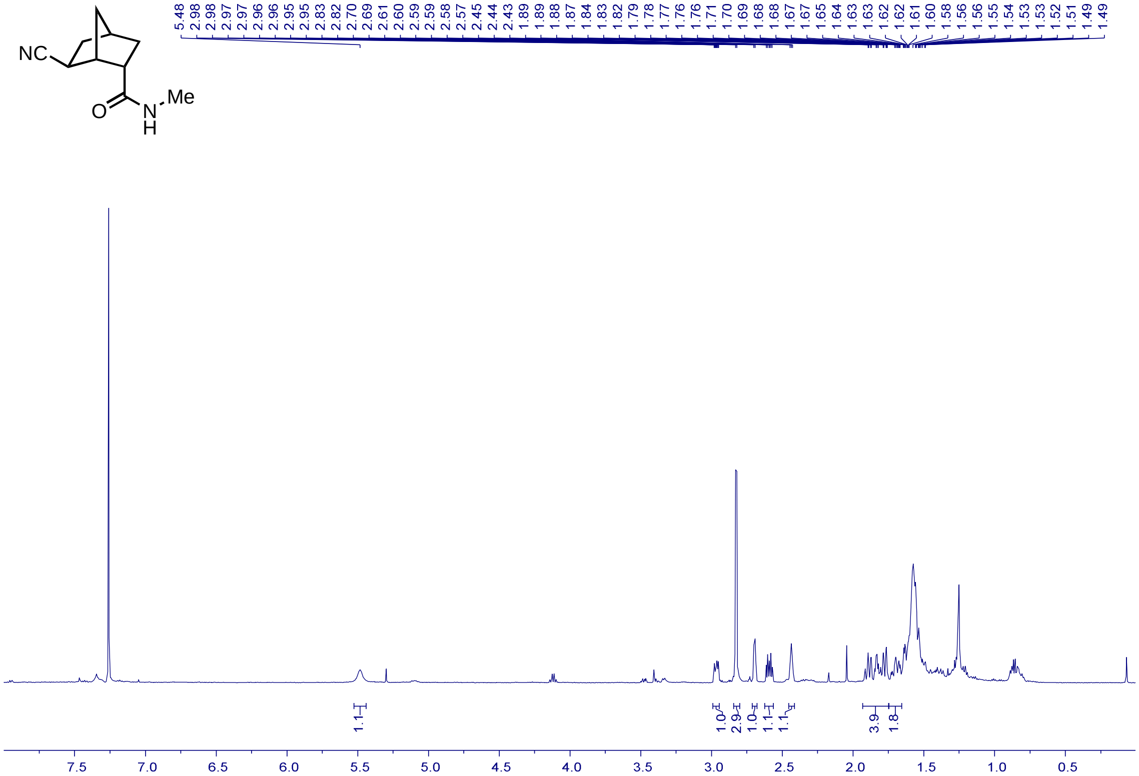

48 ^{13}C NMR (101 MHz, $CDCl_3$)

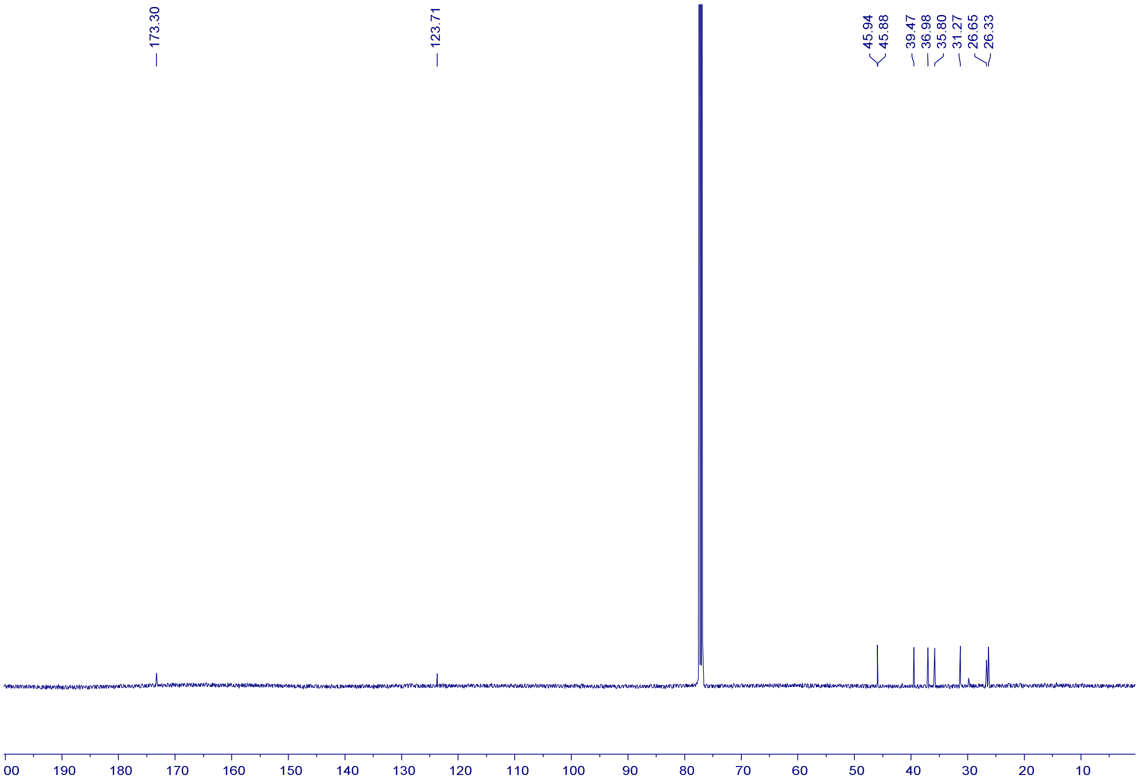

49 ^{1}H NMR (400 MHz, $CDCl_3$)

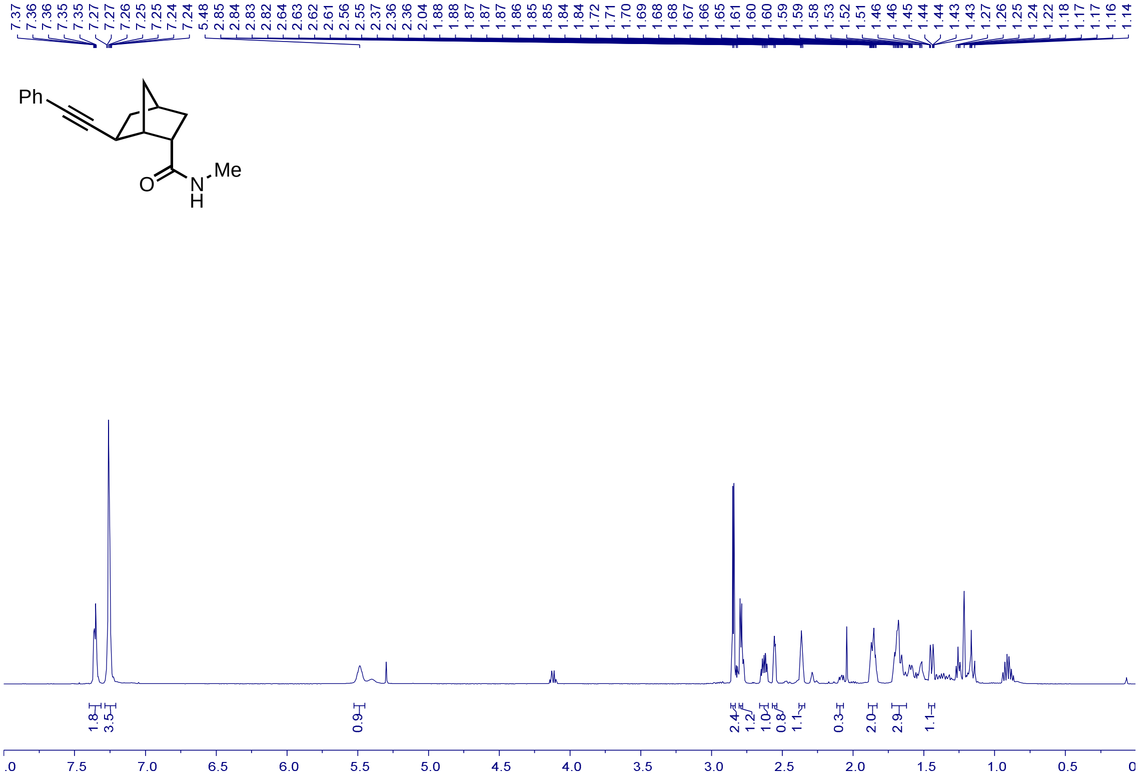

49 ^{13}C NMR (101 MHz, $CDCl_3$)

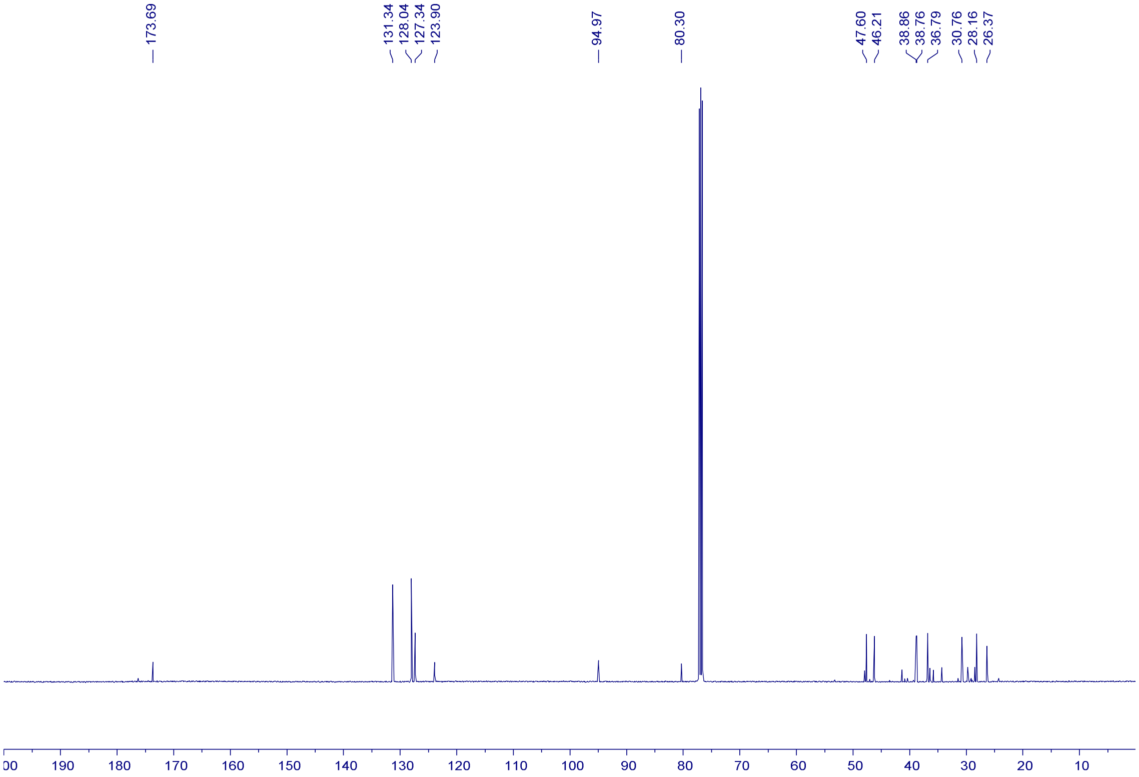

50 ^{1}H NMR (400 MHz, $CDCl_3$)

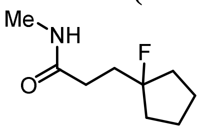

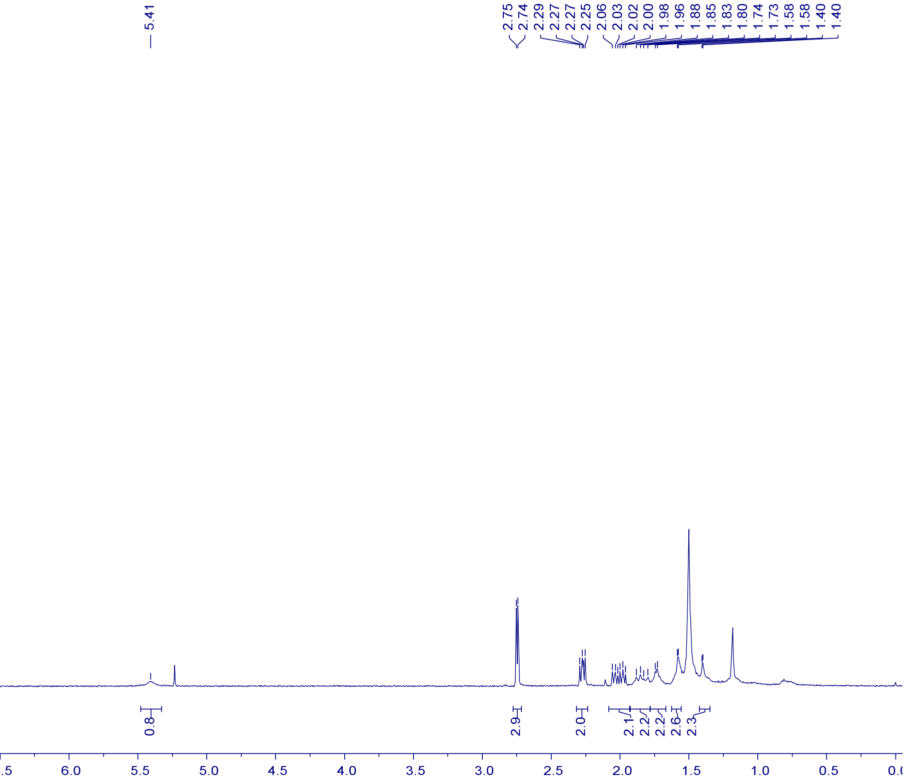

50 ^{13}C NMR (101 MHz, $CDCl_3$)

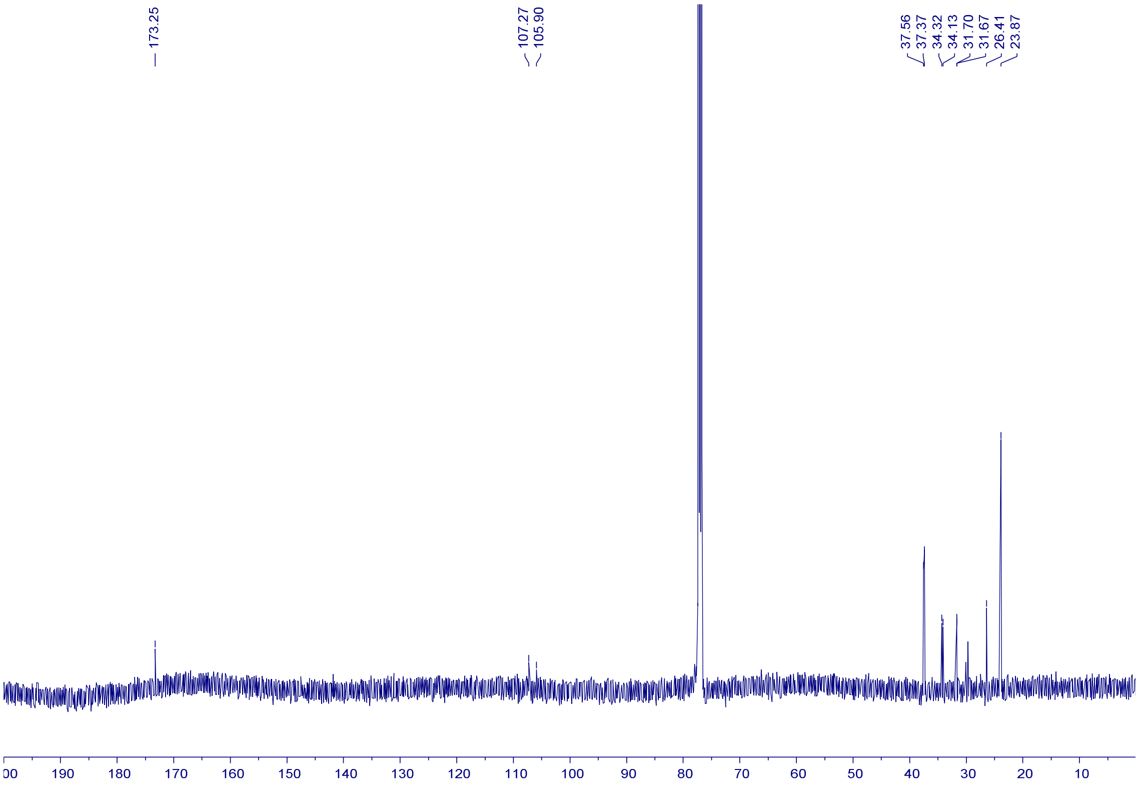

50 ^{19}F NMR (376 MHz, $CDCl_3$)

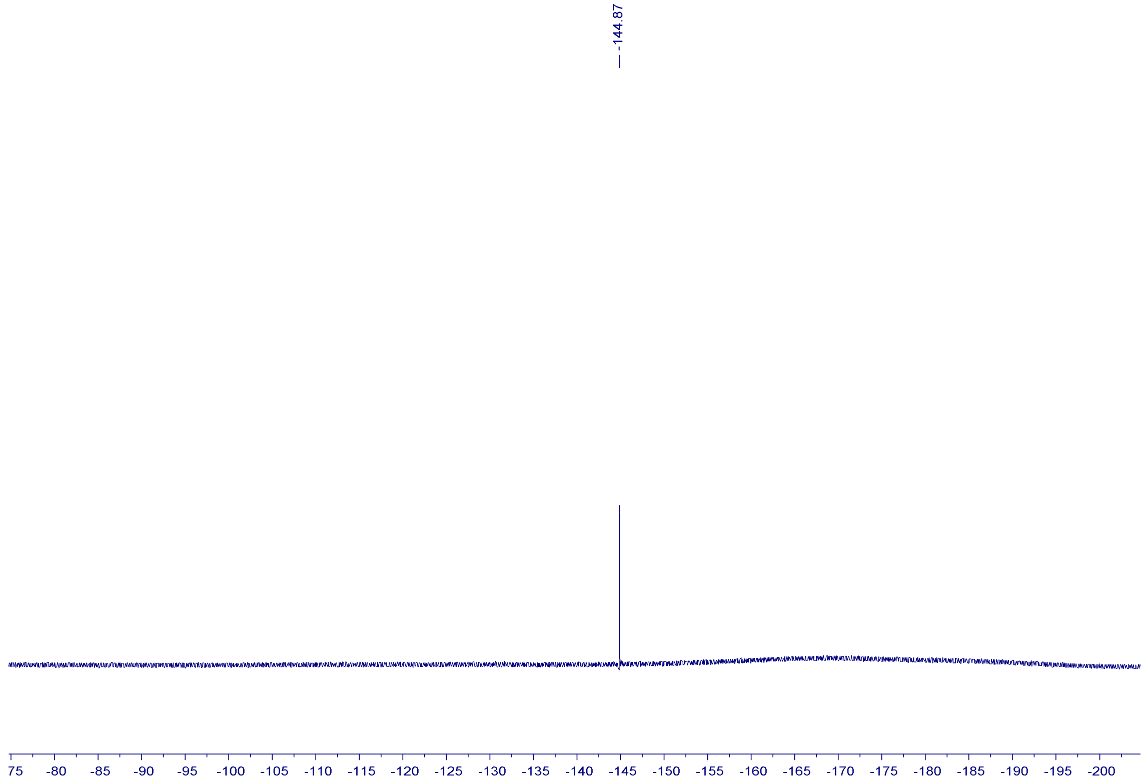

52 ^{1}H NMR (400 MHz, $CDCl_3$)

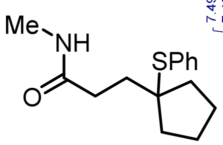

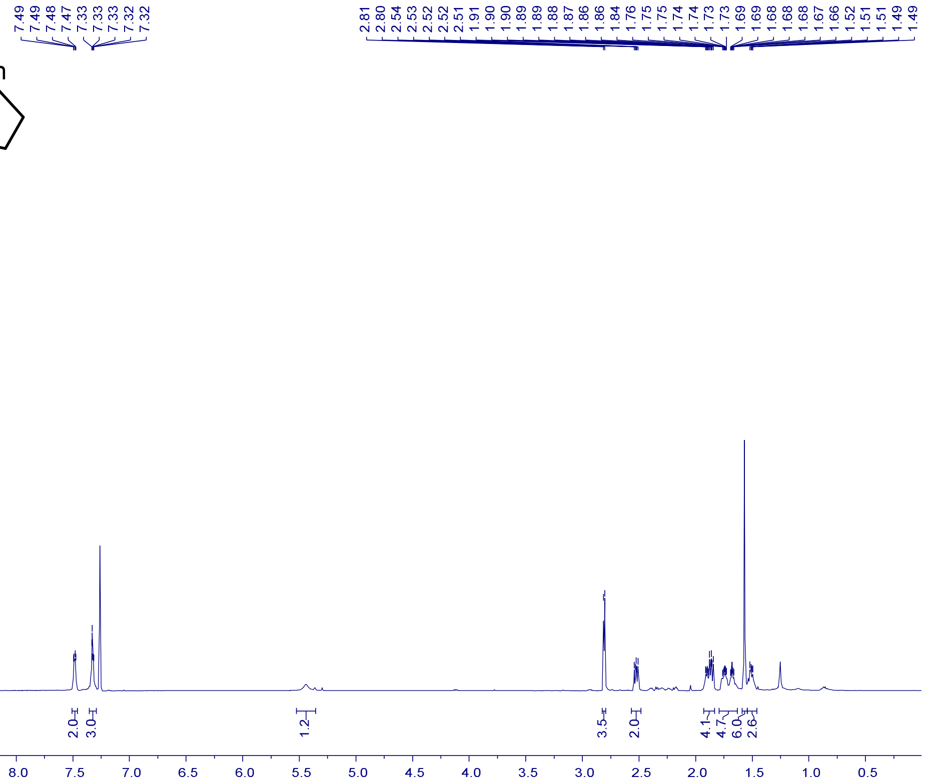

52 ^{13}C NMR (101 MHz, $CDCl_3$)

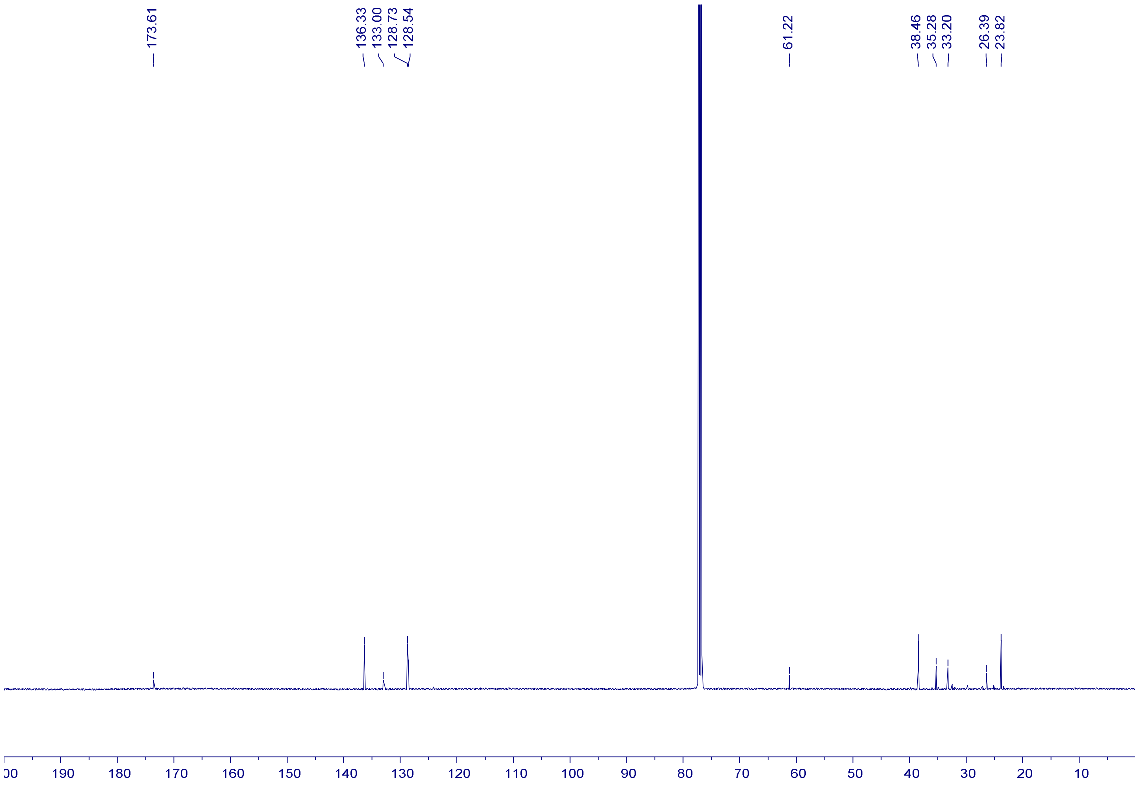

53 ^{1}H NMR (400 MHz, $CDCl_3$)

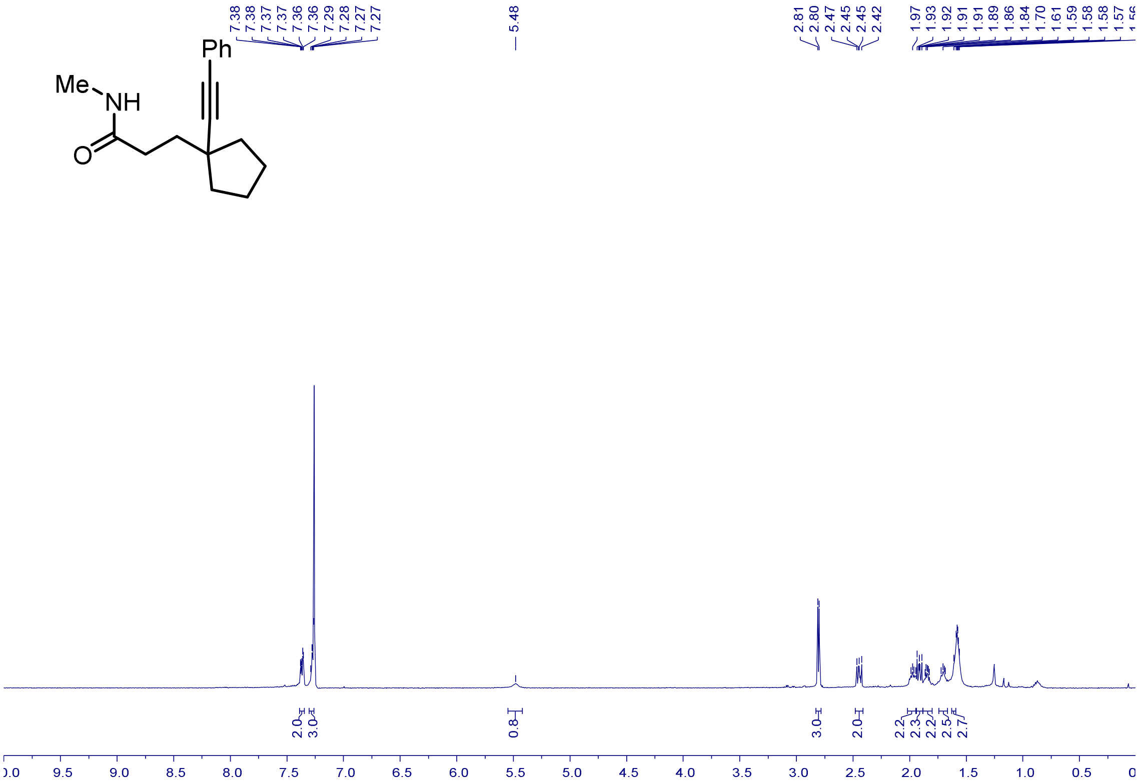

53 ^{13}C NMR (101 MHz, $CDCl_3$)

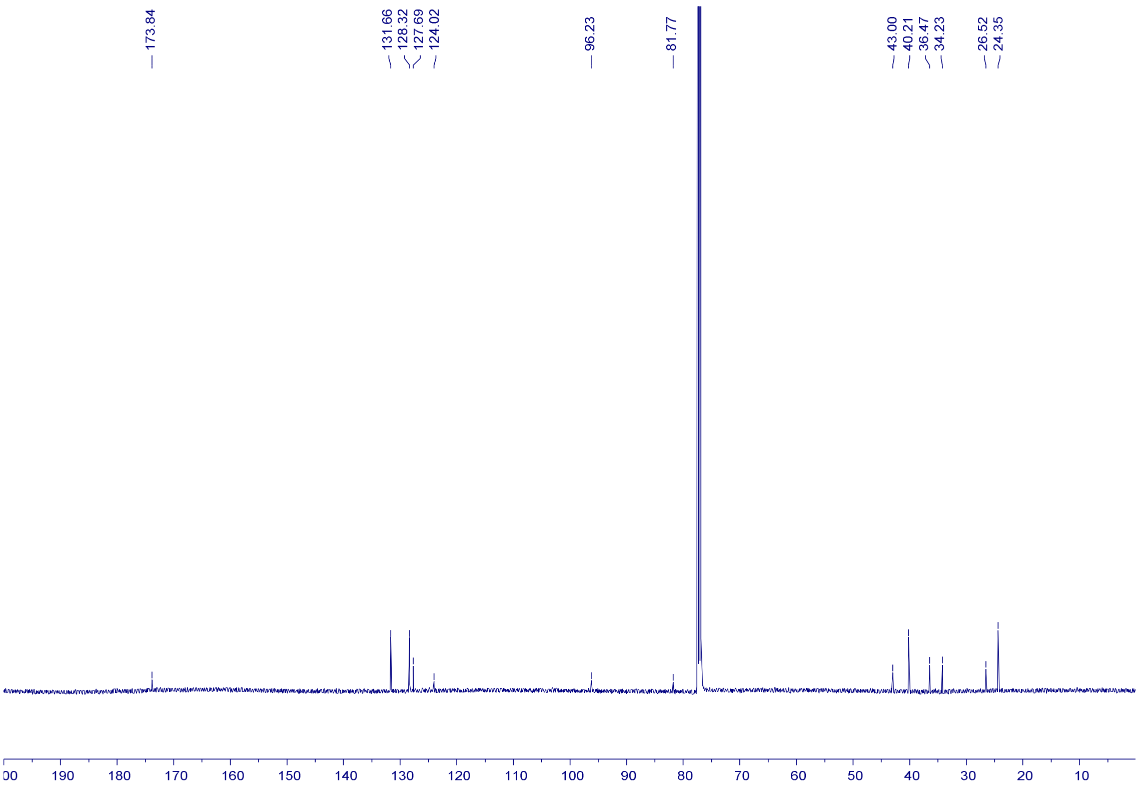

54 ^{1}H NMR (400 MHz, $CDCl_3$)

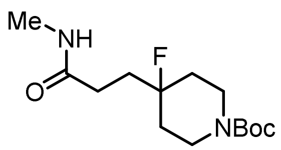

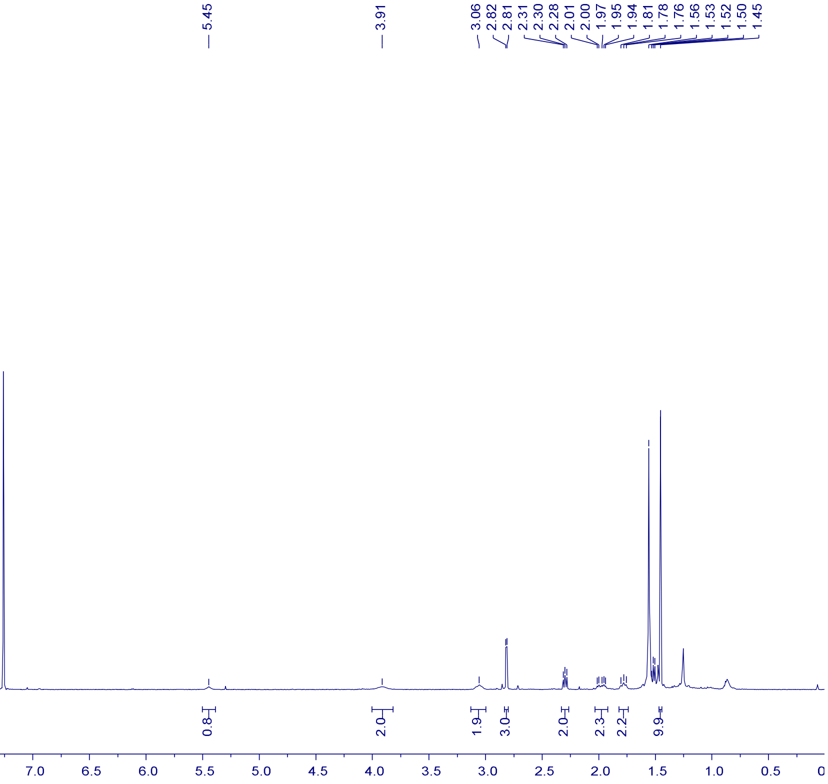

54 ^{13}C NMR (101 MHz, $CDCl_3$)

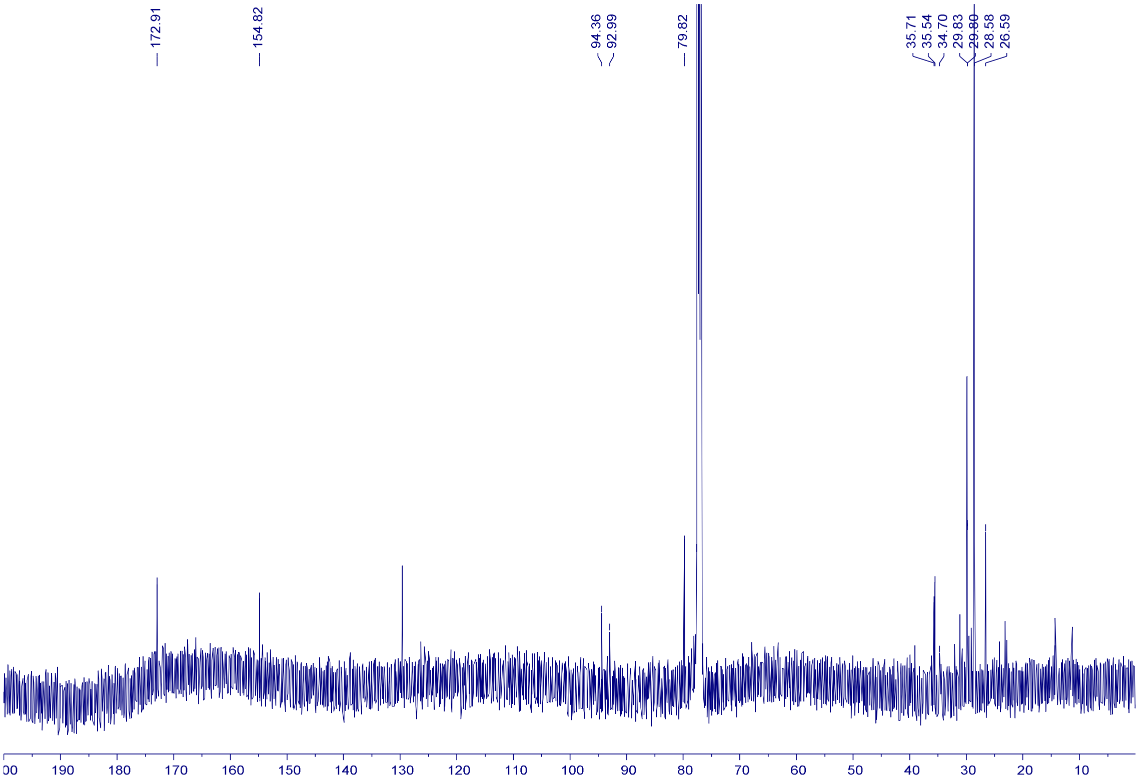

54 ^{19}F NMR (376 MHz, $CDCl_3$)

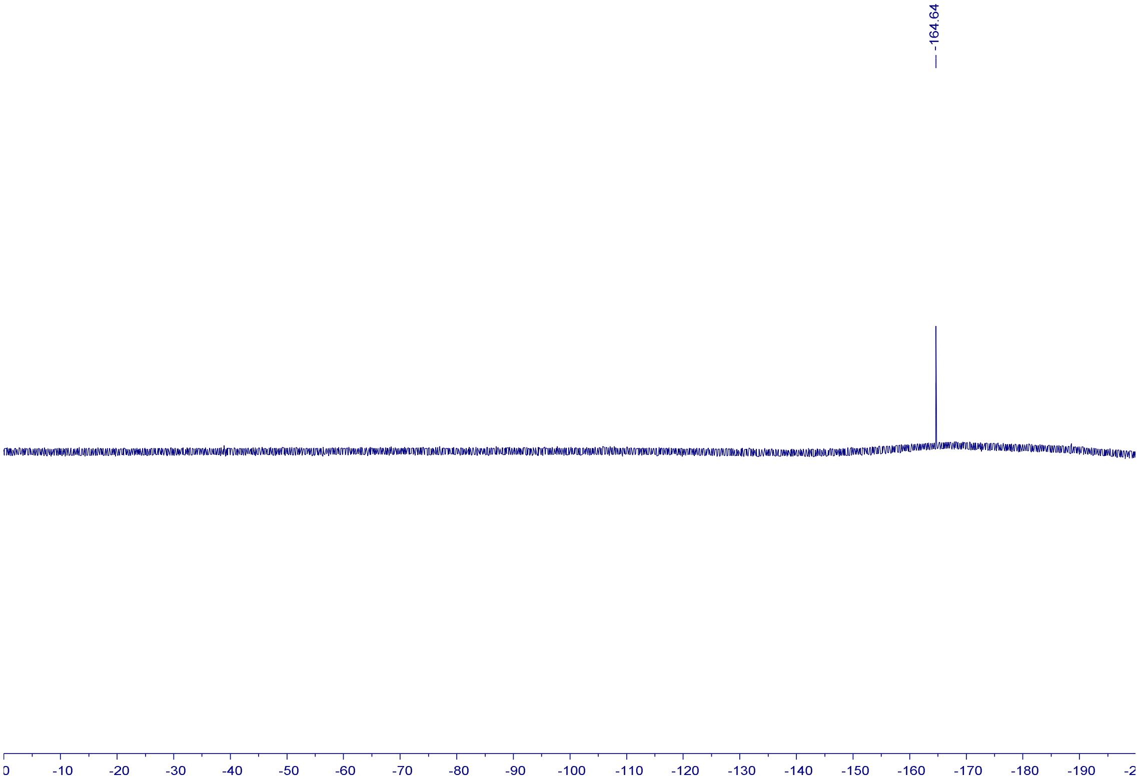

55 ^{1}H NMR (400 MHz, $CDCl_3$)

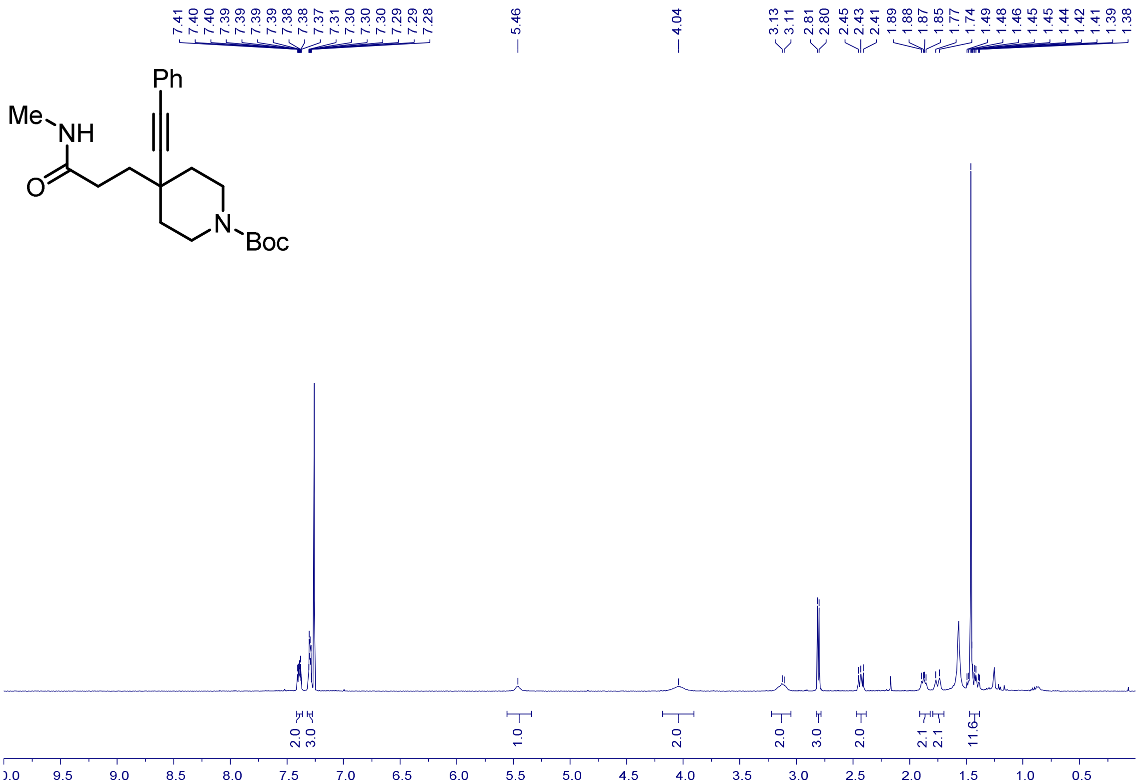

55 ^{13}C NMR (101 MHz, $CDCl_3$)

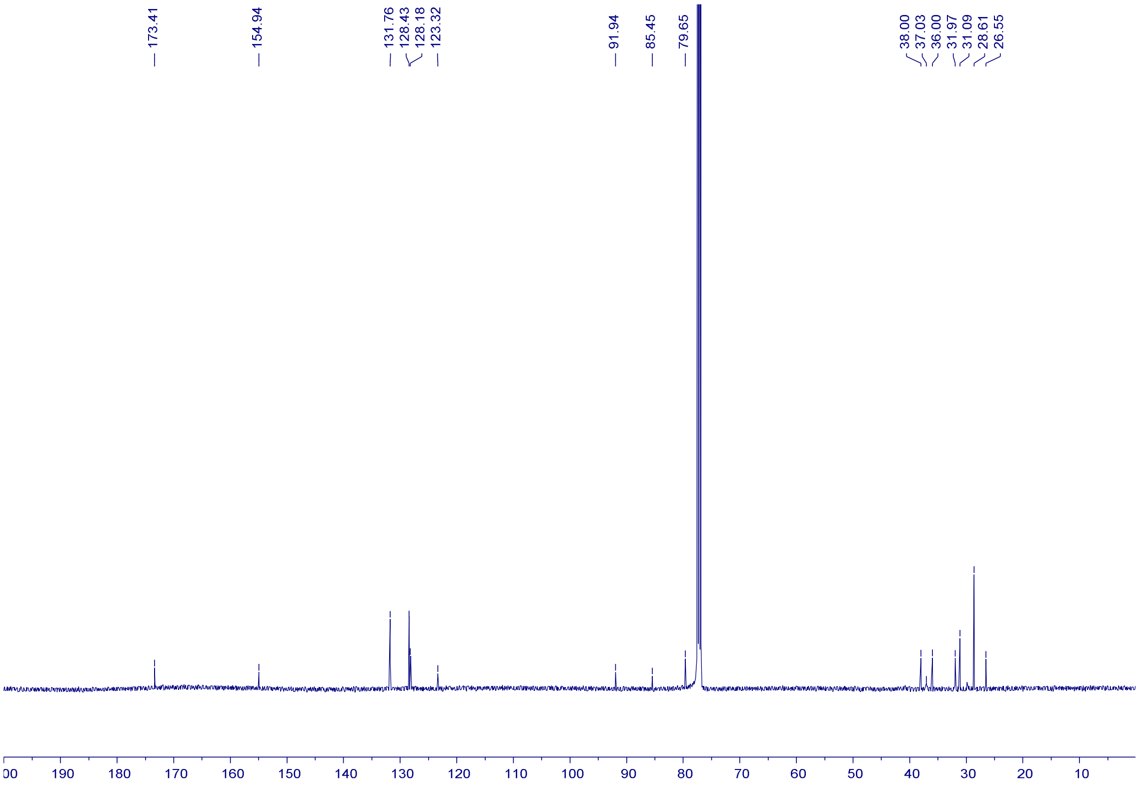

56 ^{1}H NMR (400 MHz, $CDCl_3$)

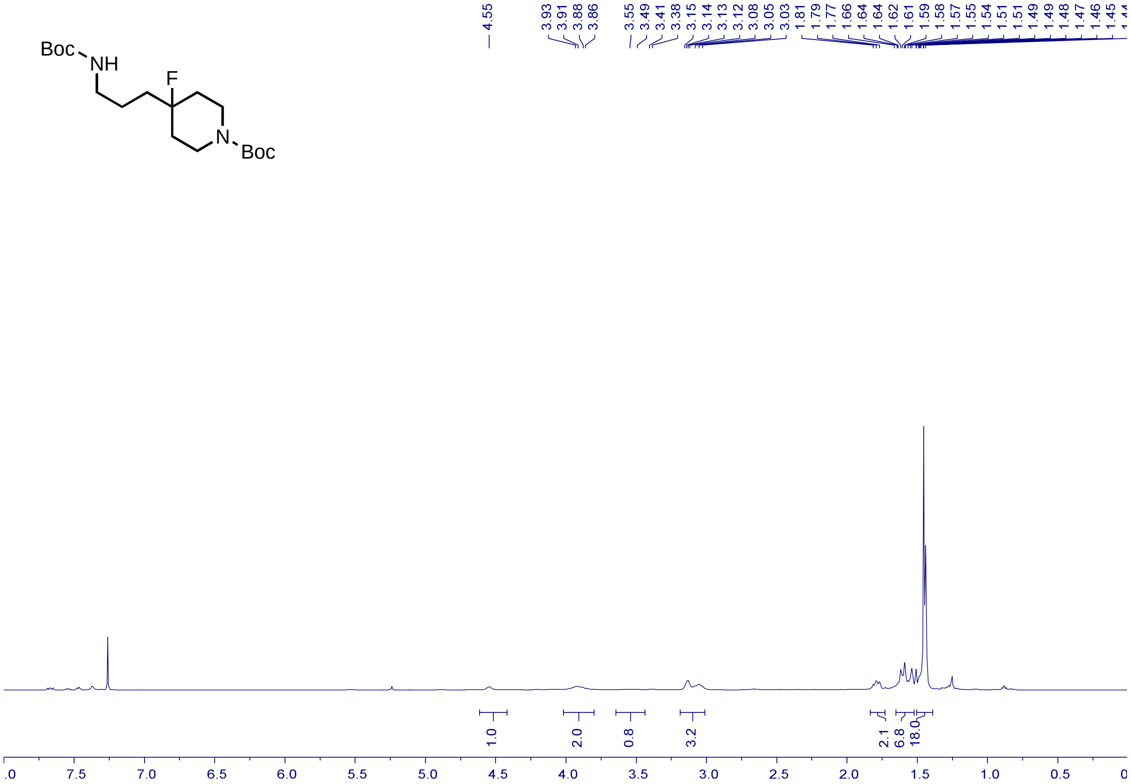

56 ^{13}C NMR (101 MHz, $CDCl_3$)

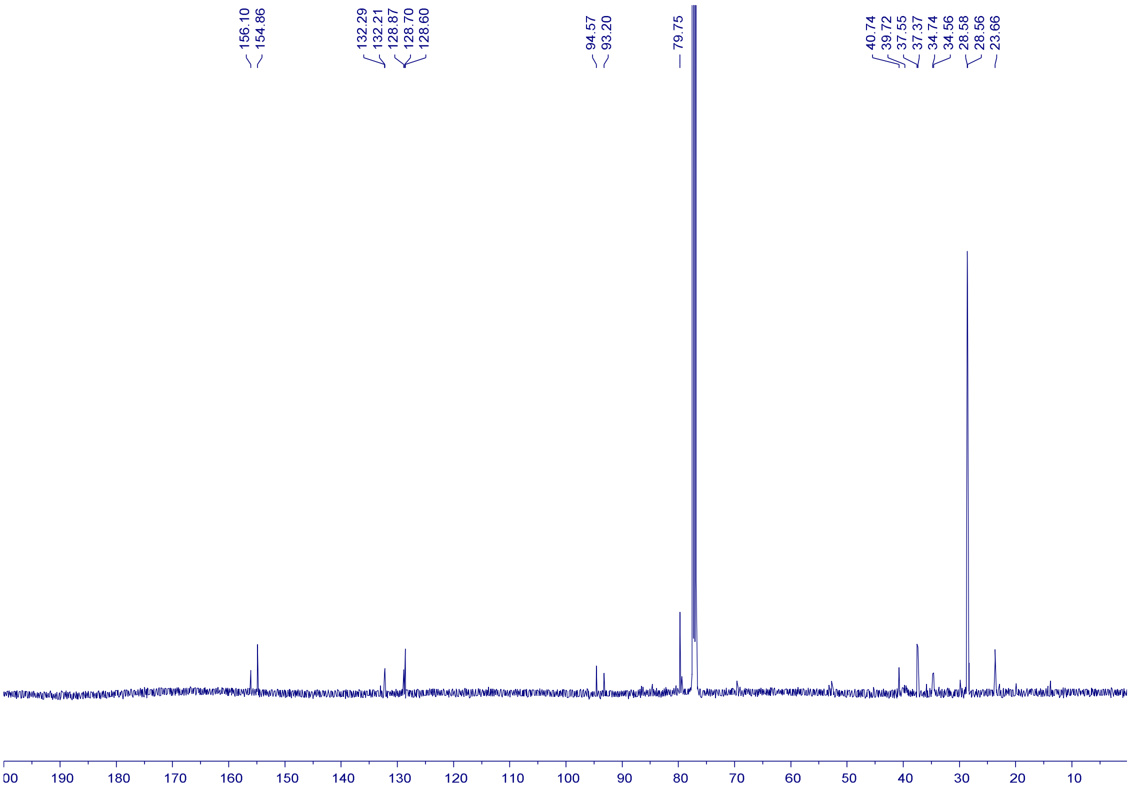

56 ^{19}F NMR (376 MHz, $CDCl_3$)

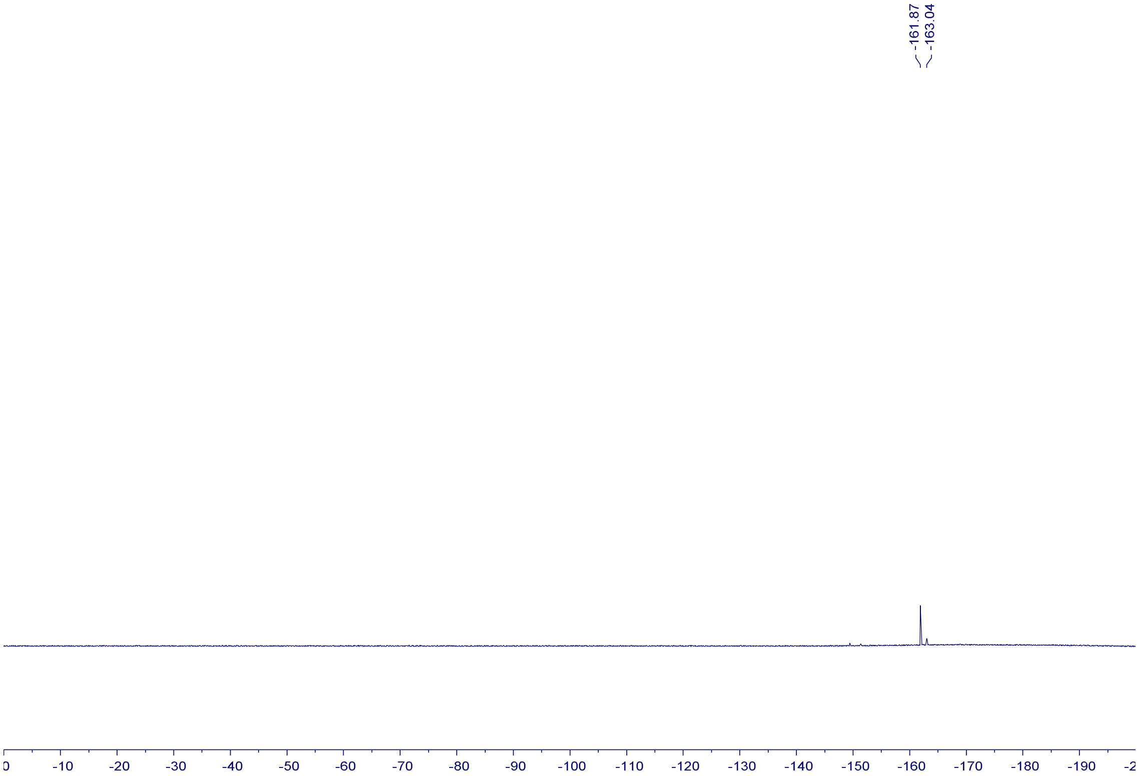

57 ^{1}H NMR (400 MHz, $CDCl_3$)

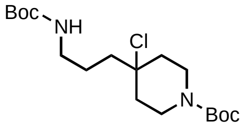

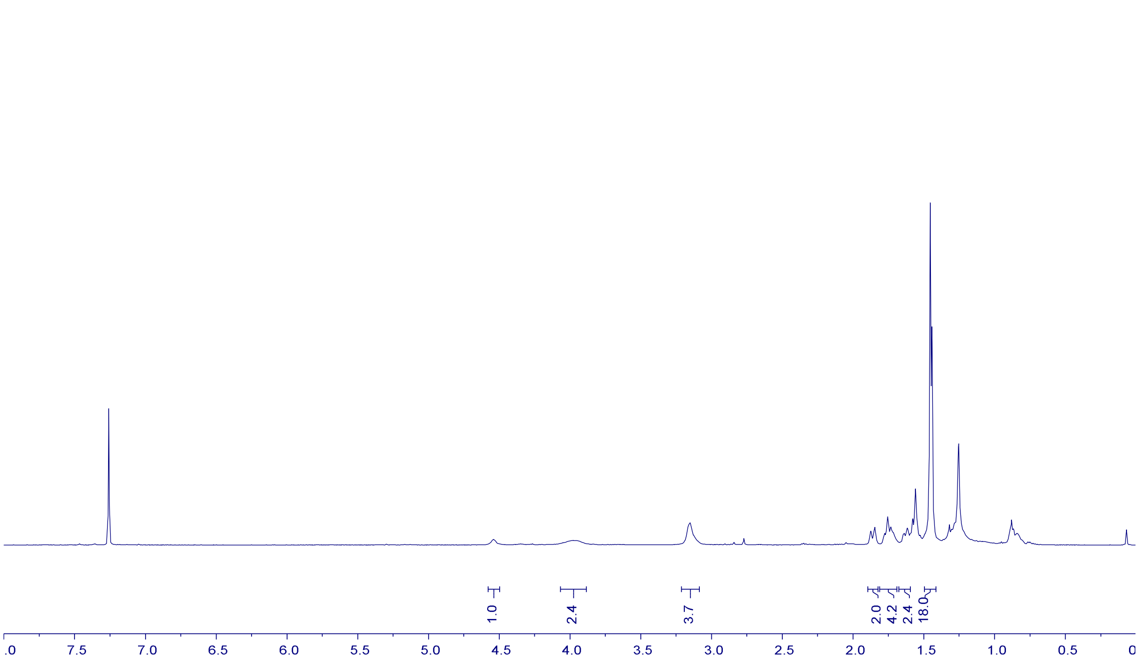

57 ^{13}C NMR (101 MHz, $CDCl_3$)

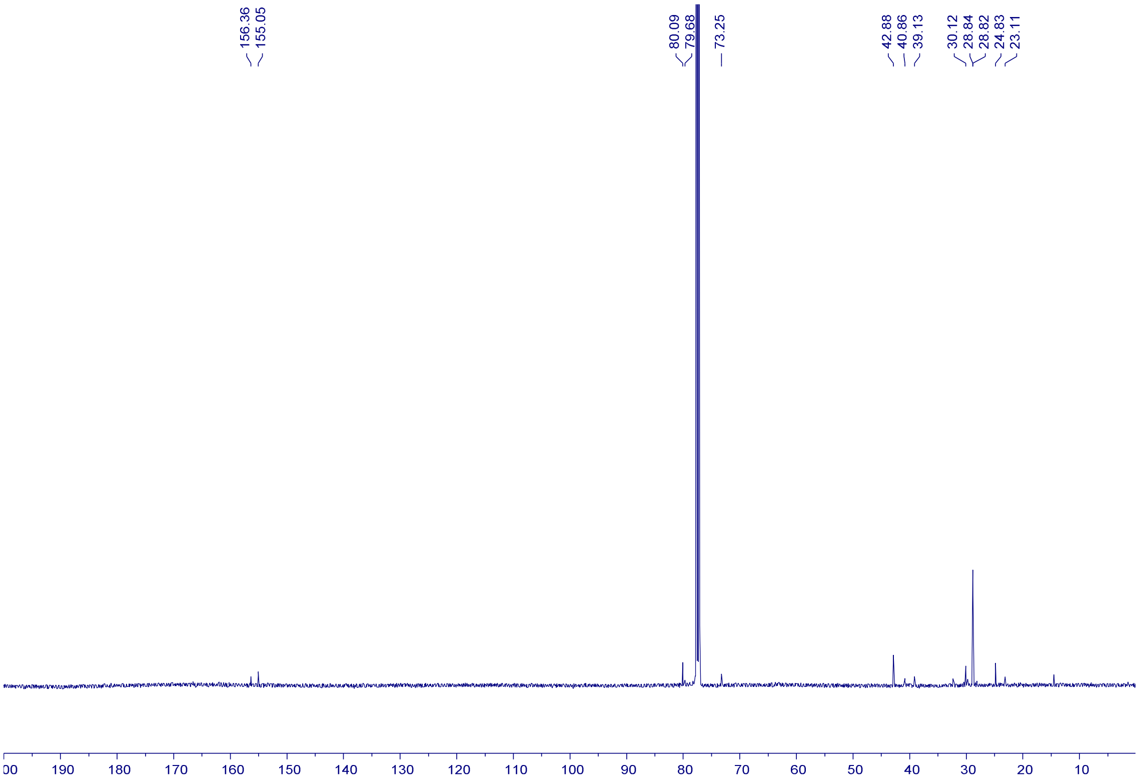

58 ^{1}H NMR (400 MHz, $CDCl_3$)

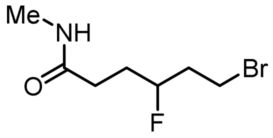

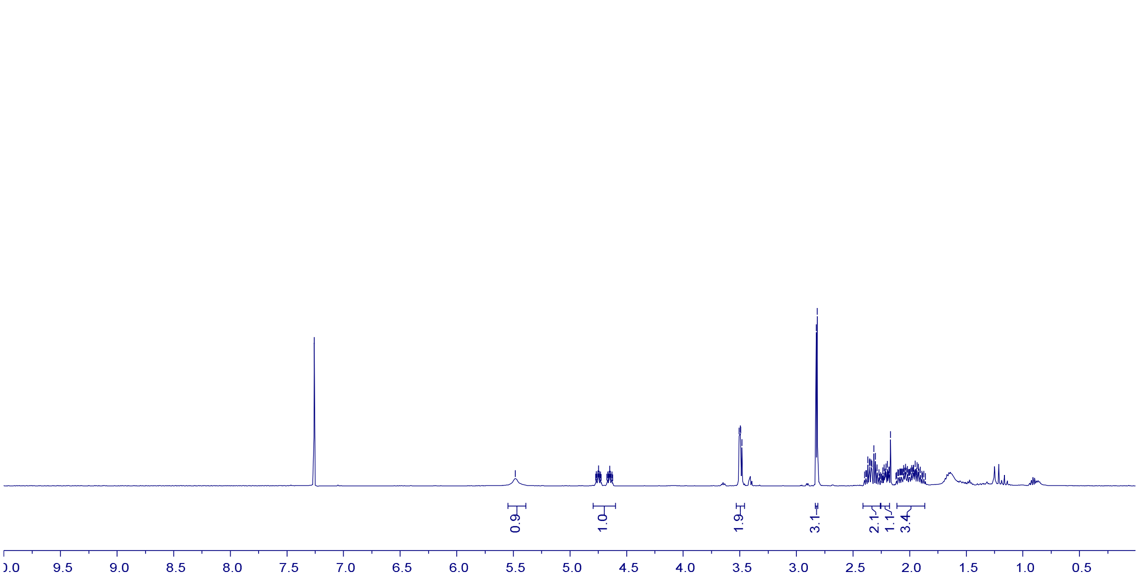

58 ^{13}C NMR (101 MHz, $CDCl_3$)

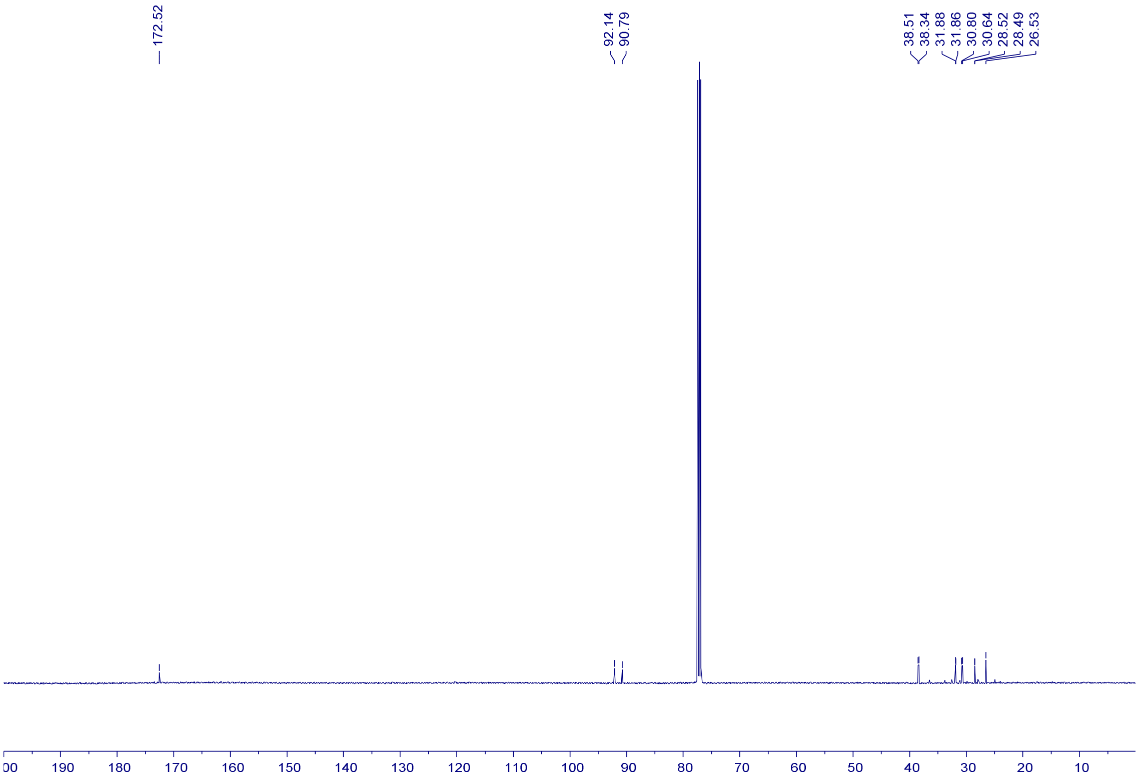

58 ^{19}F NMR (376 MHz, $CDCl_3$)

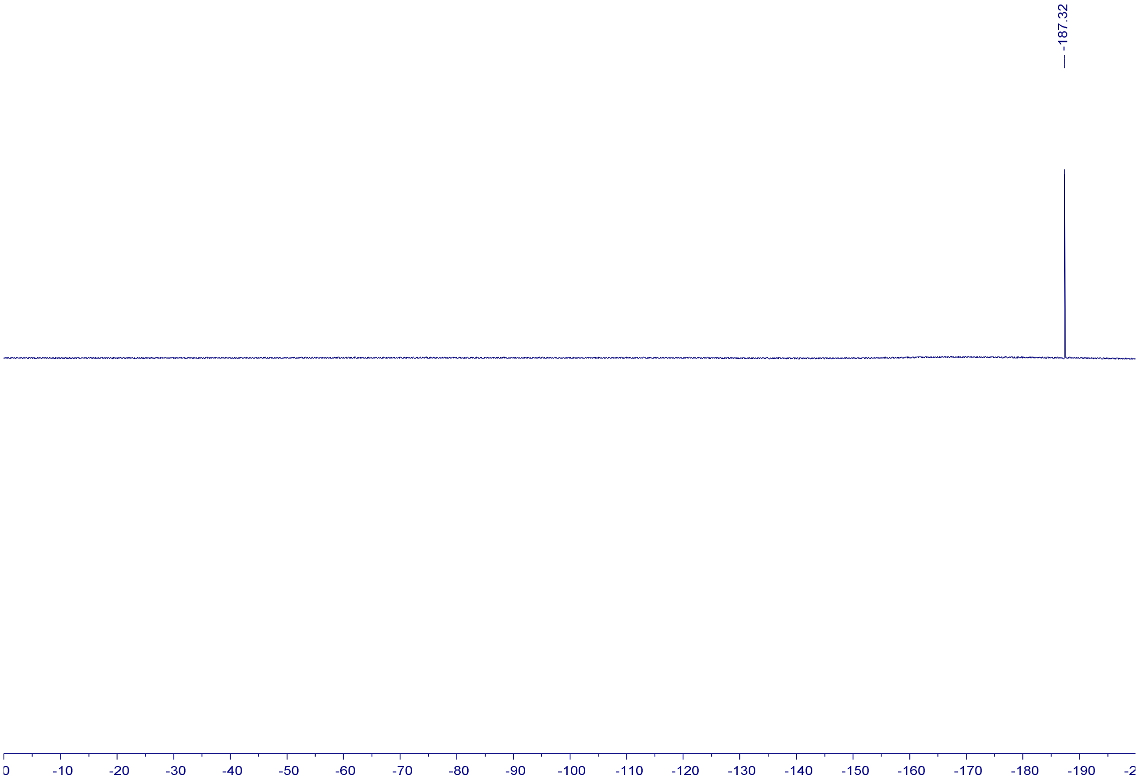

59 ^{1}H NMR (400 MHz, $CDCl_3$)

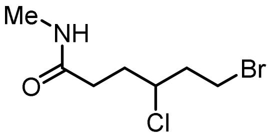

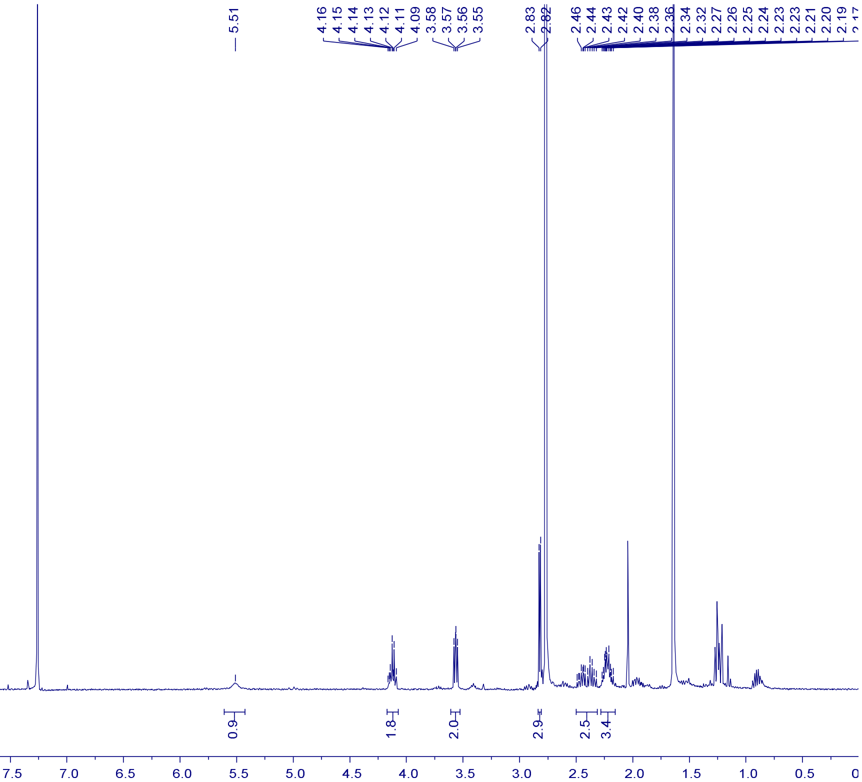

59 ^{13}C NMR (101 MHz, $CDCl_3$)

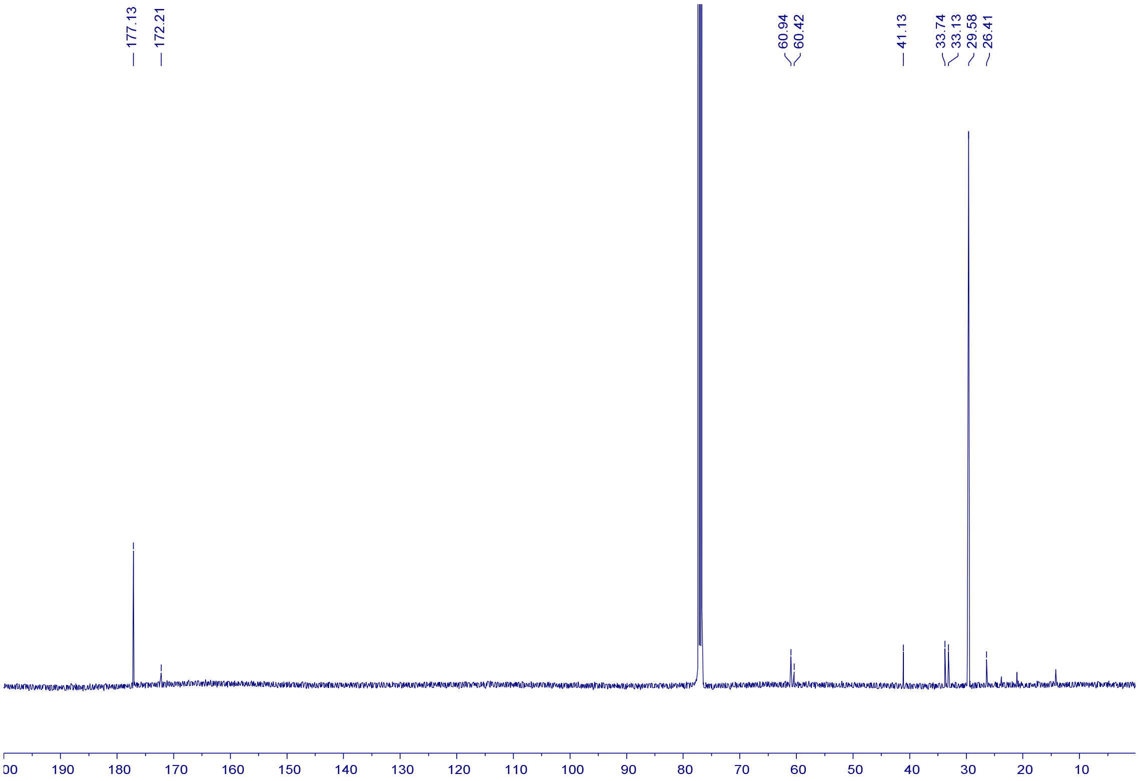

60 ^{1}H NMR (400 MHz, $CDCl_3$)

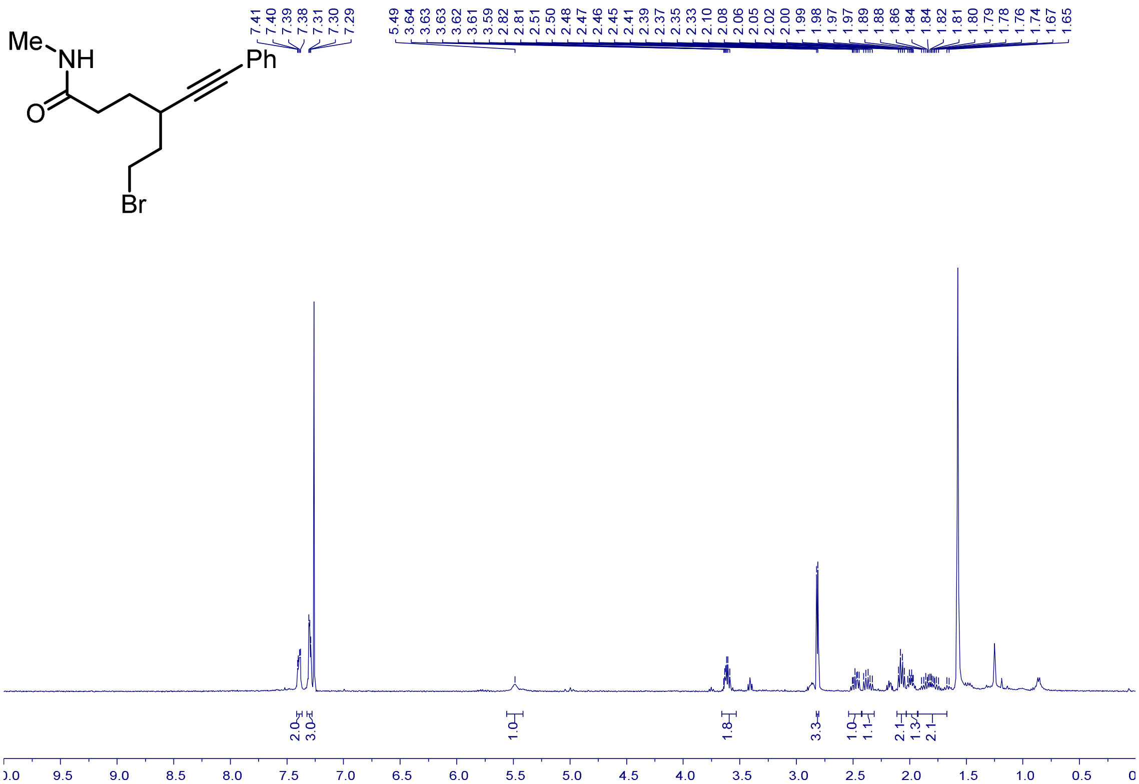

60 ^{13}C NMR (101 MHz, $CDCl_3$)

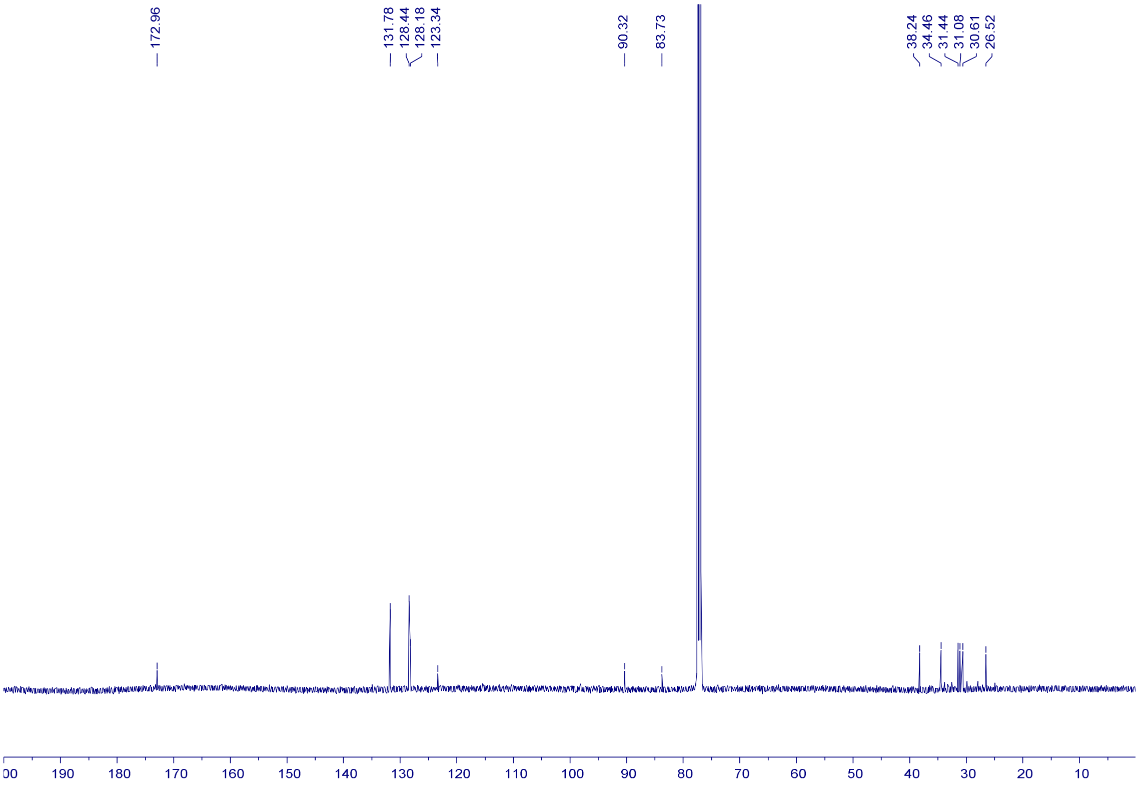

61 ^{1}H NMR (400 MHz, $CDCl_3$)

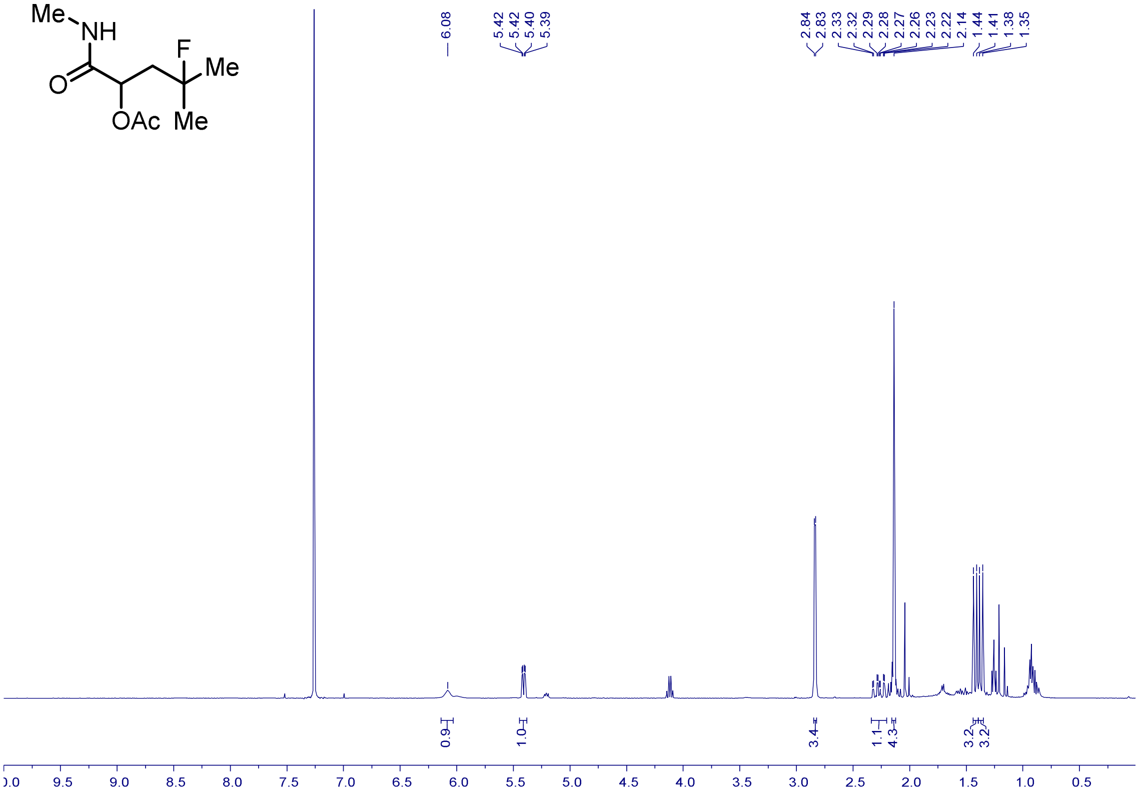

61 ^{13}C NMR (101 MHz, $CDCl_3$)

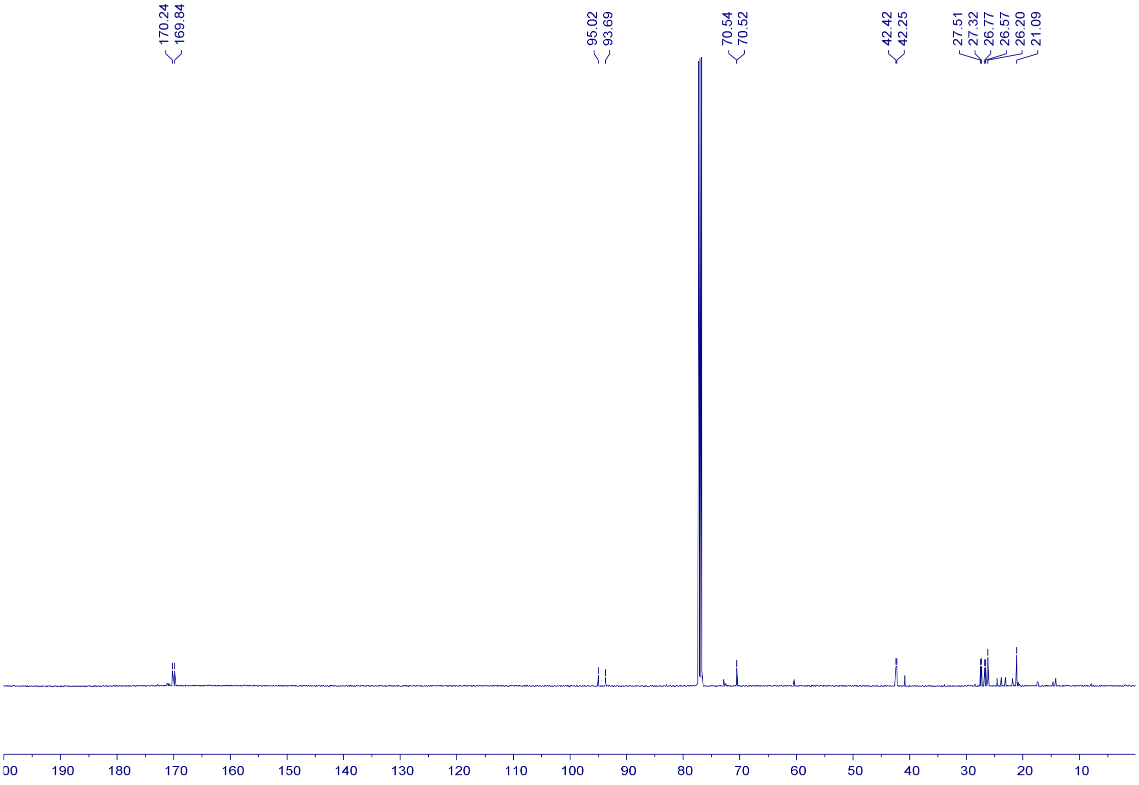

61 ^{19}F NMR (376 MHz, $CDCl_3$)

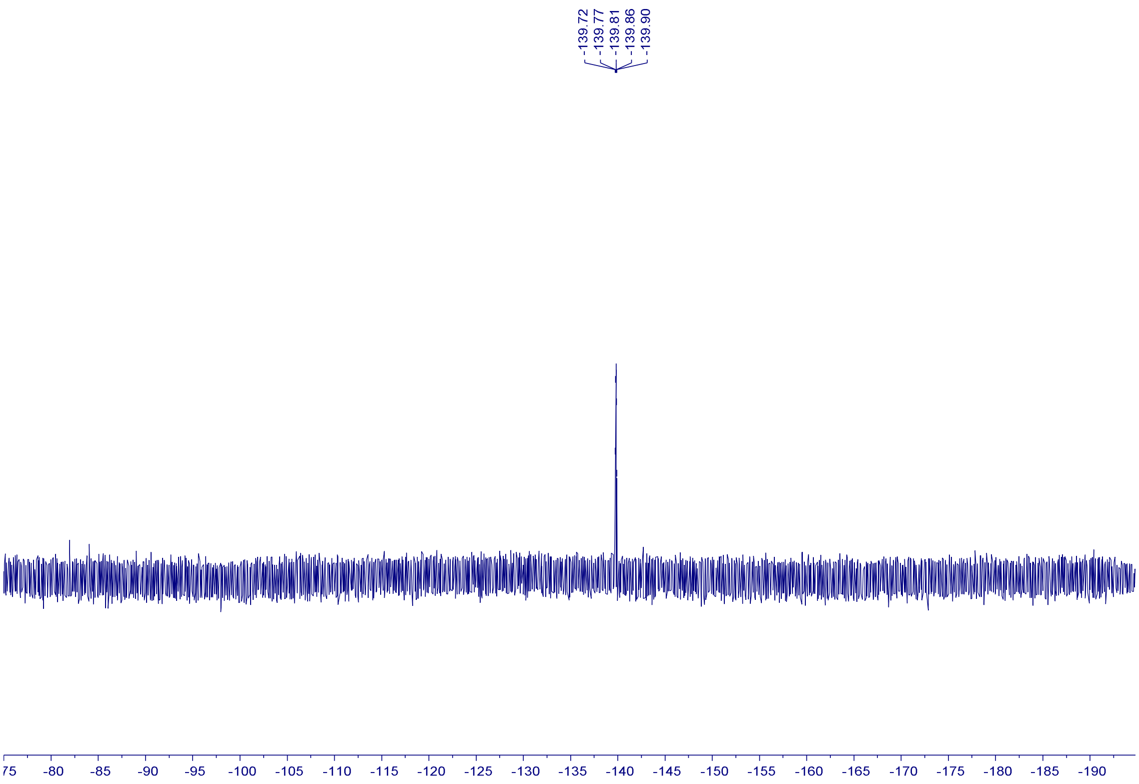

62 ^{1}H NMR (500 MHz, $CDCl_3$)

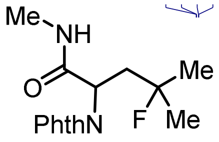

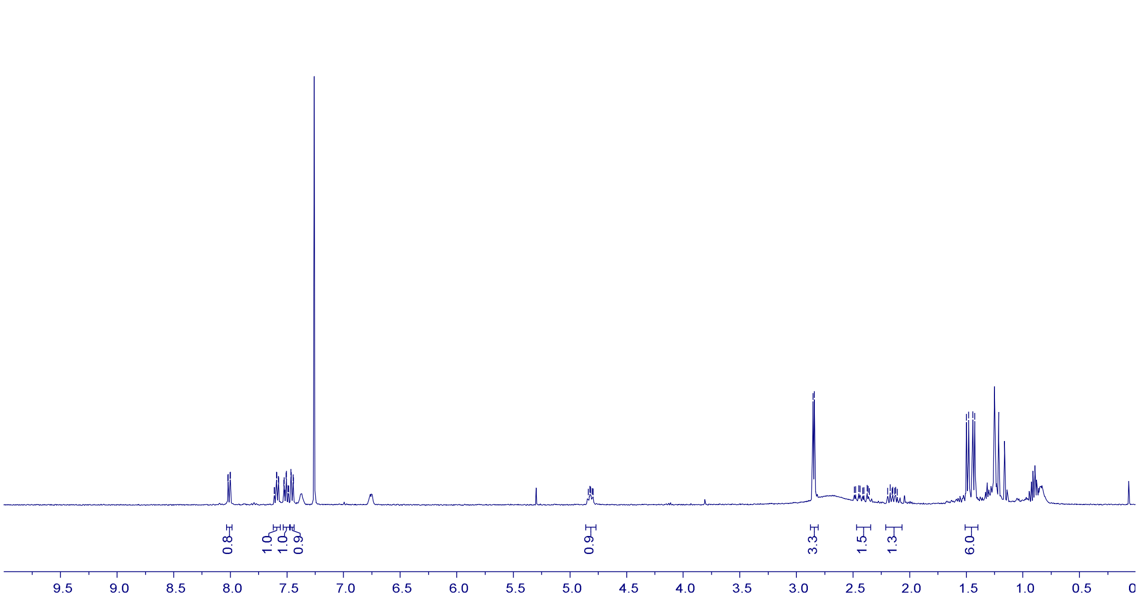

62 ^{13}C NMR (101 MHz, $CDCl_3$)

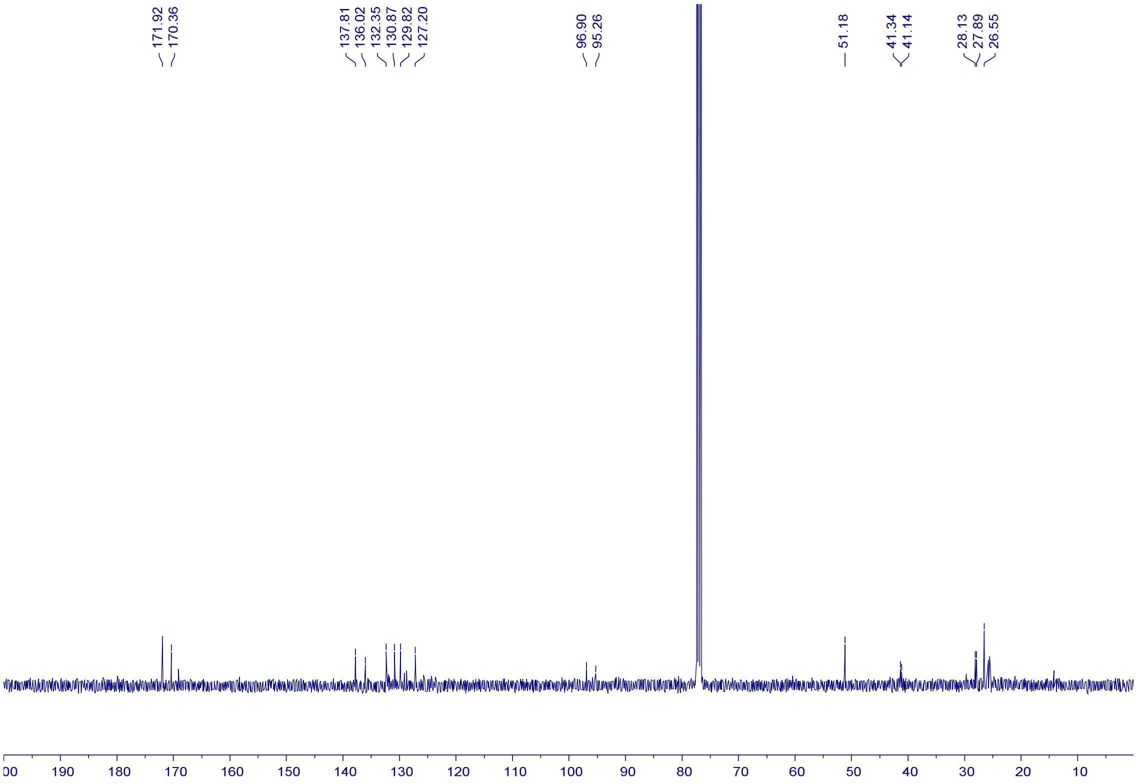

62 ^{19}F NMR (376 MHz, $CDCl_3$)

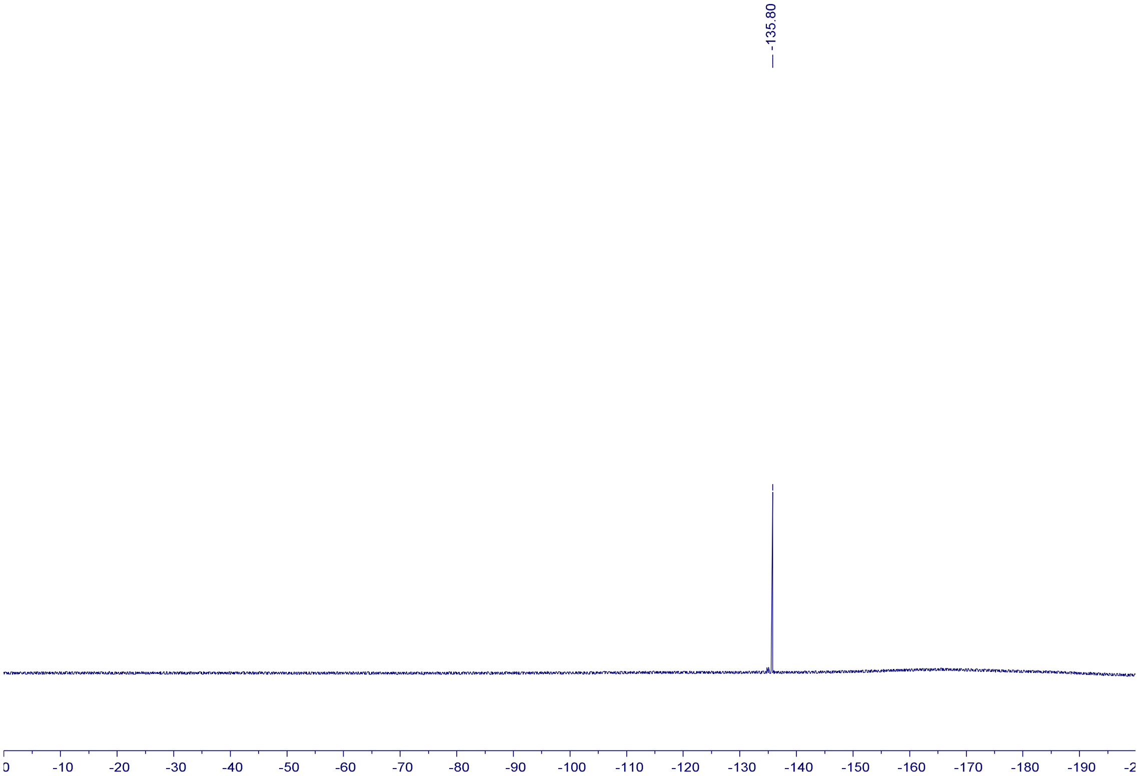

63 ^{1}H NMR (400 MHz, $CDCl_3$)

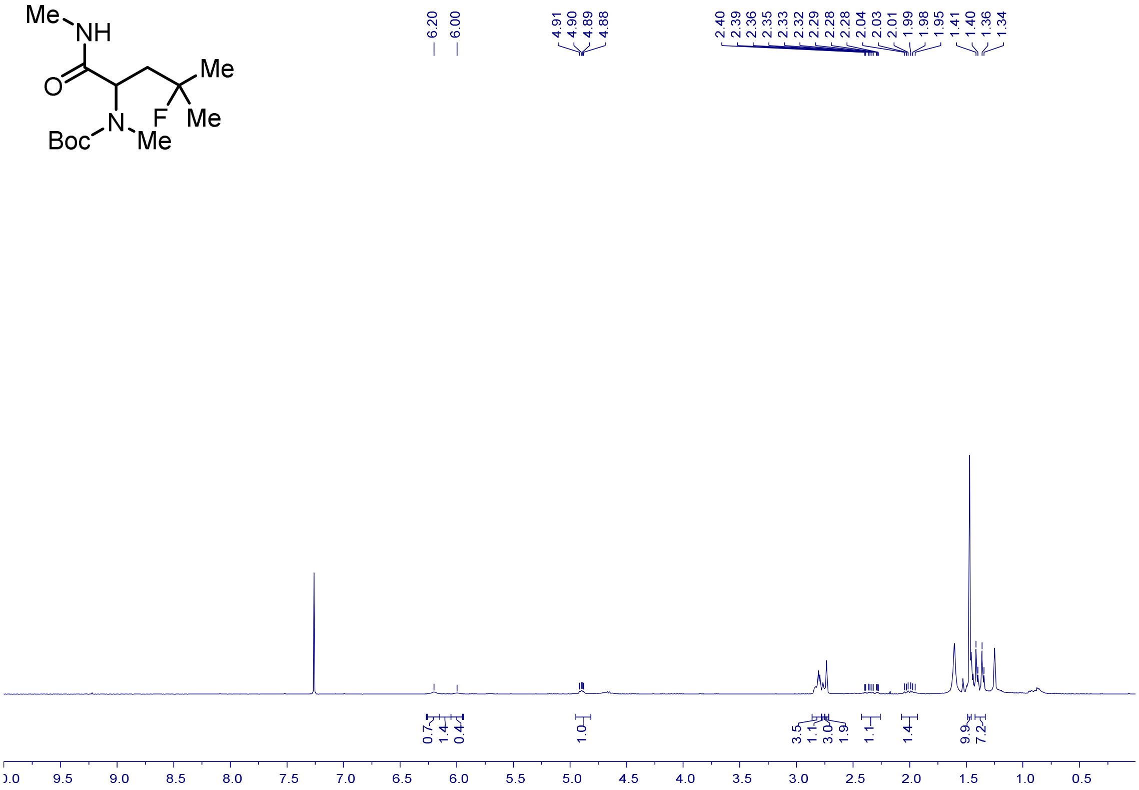

63 ^{13}C NMR (101 MHz, $CDCl_3$)

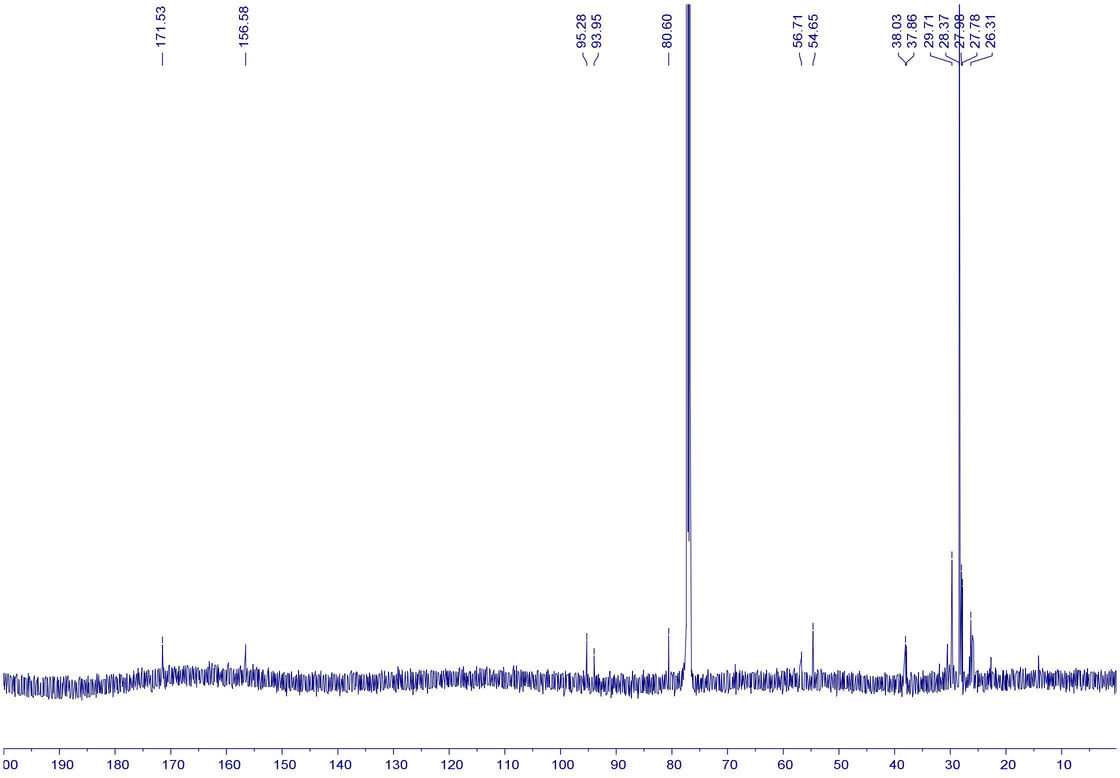

63^{19}F NMR (376 MHz, $CDCl_3$)

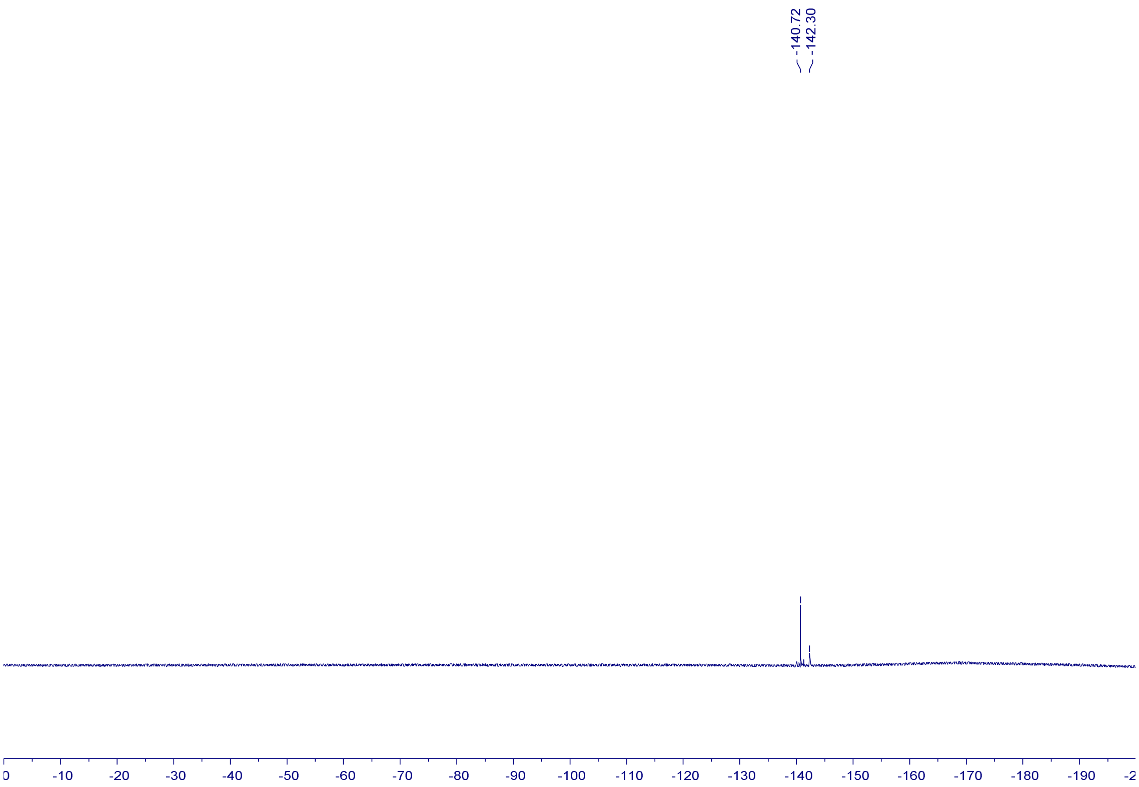

64 ^{1}H NMR (400 MHz, $CDCl_3$)

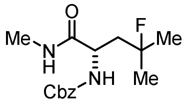

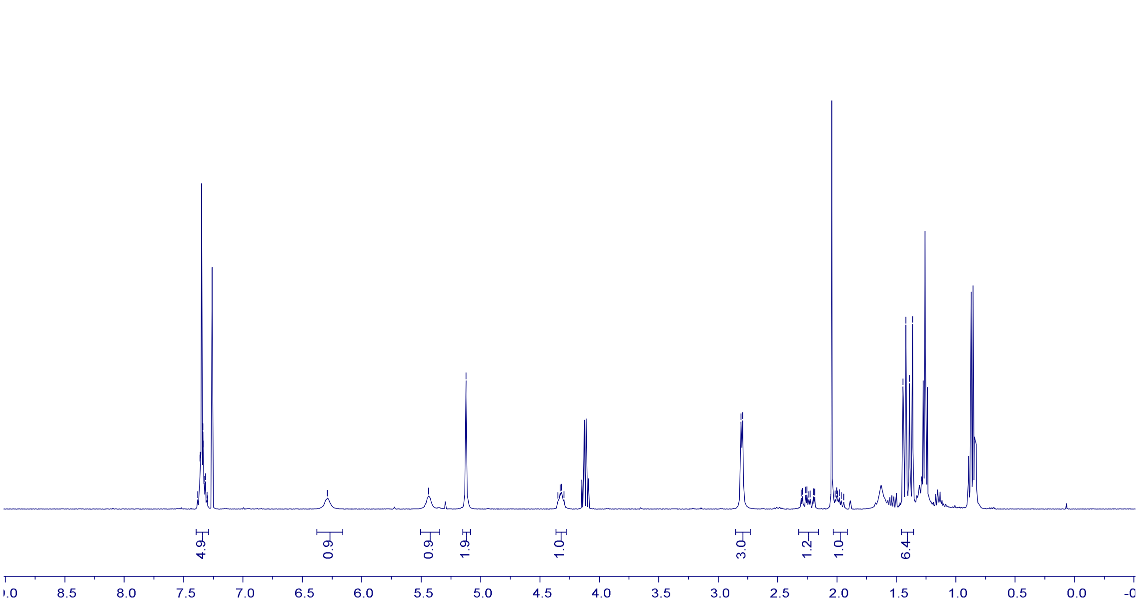

64 ^{13}C NMR (126 MHz, $CDCl_3$)

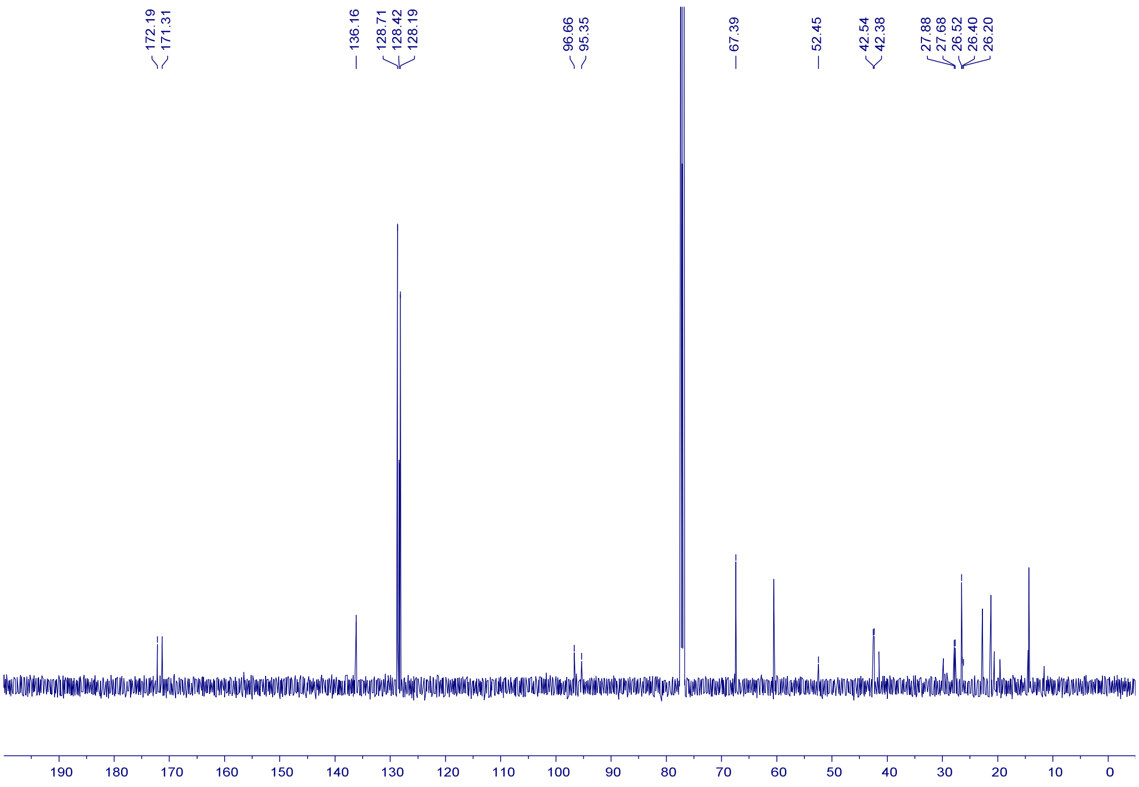

64 ^{19}F NMR (376 MHz, $CDCl_3$)

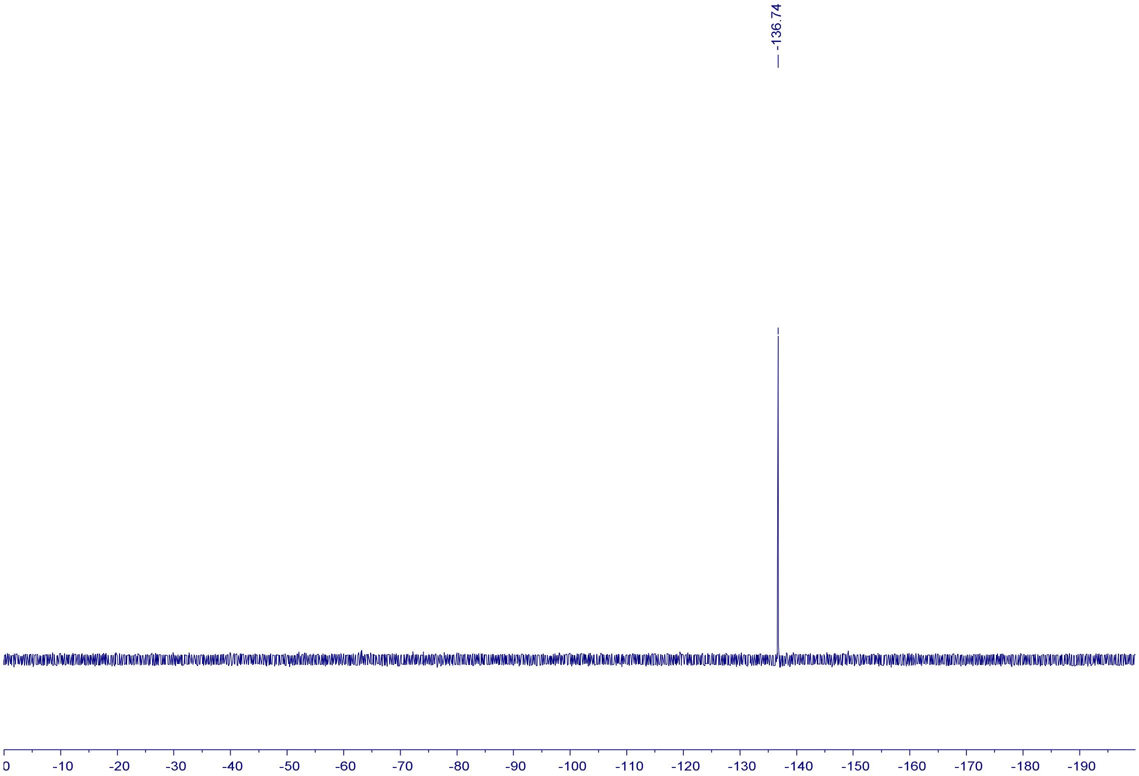

65 ^{1}H NMR (500 MHz, $CDCl_3$)

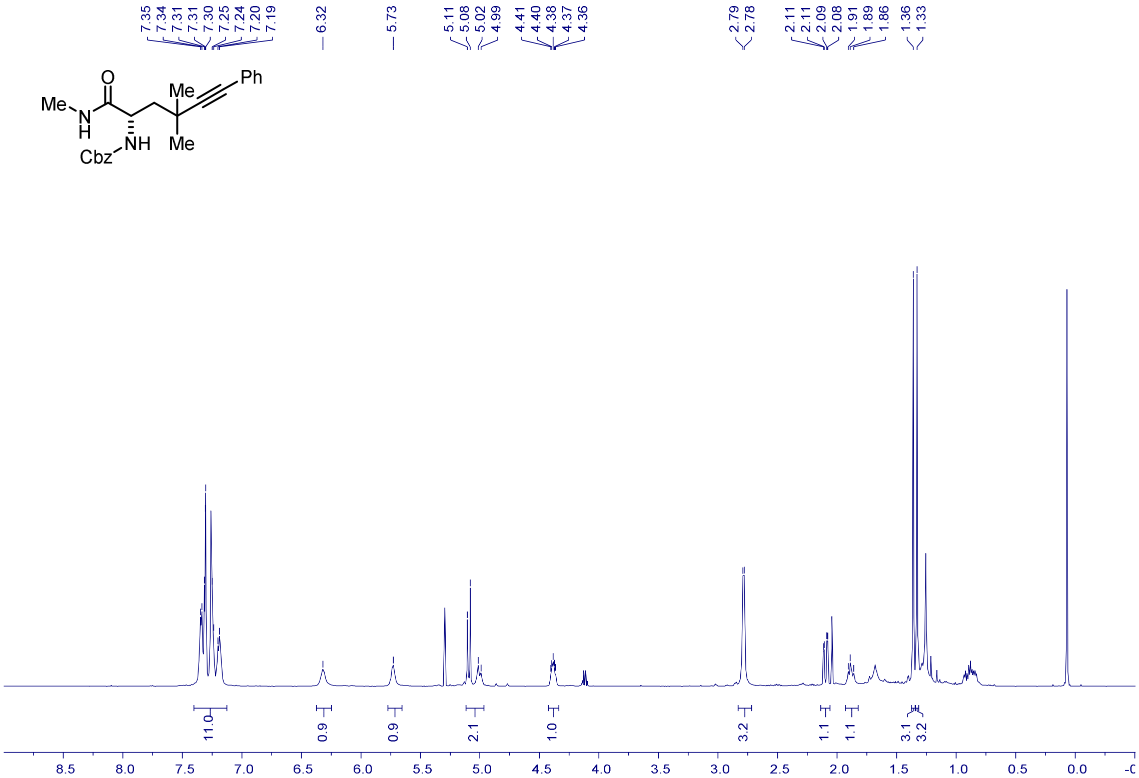

65 ^{13}C NMR (126 MHz, $CDCl_3$)

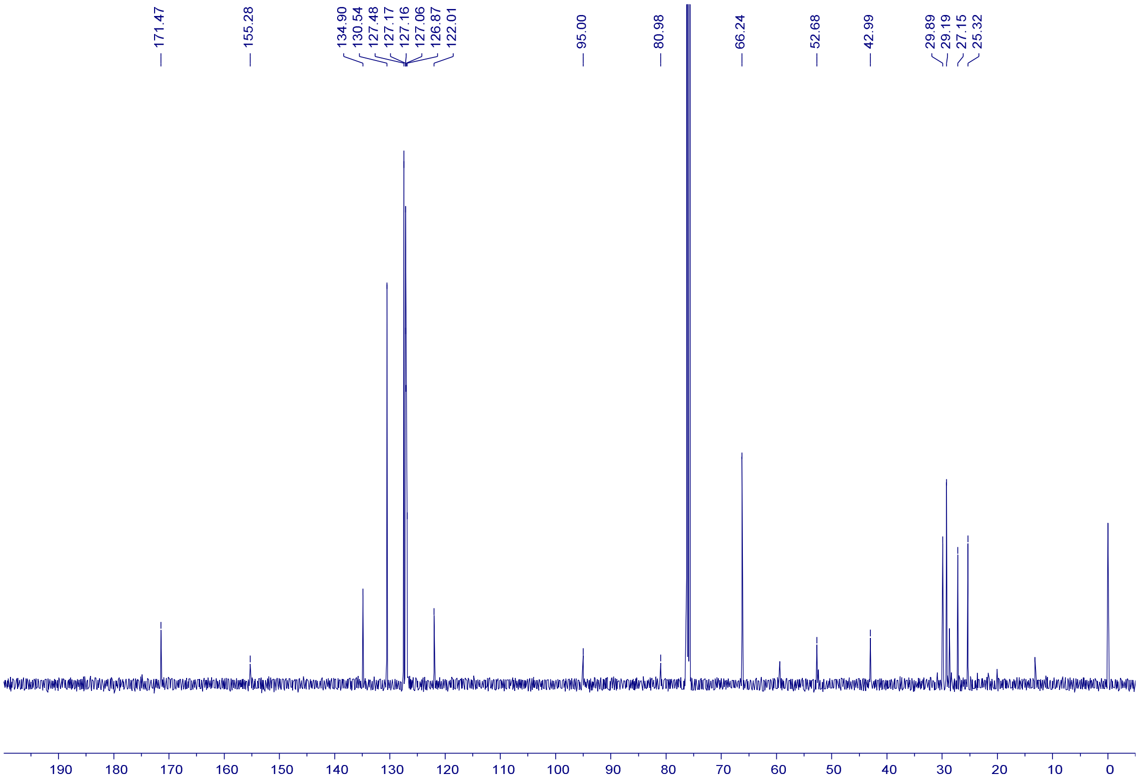

67 ^{1}H NMR (400 MHz, $CDCl_3$) first isomer

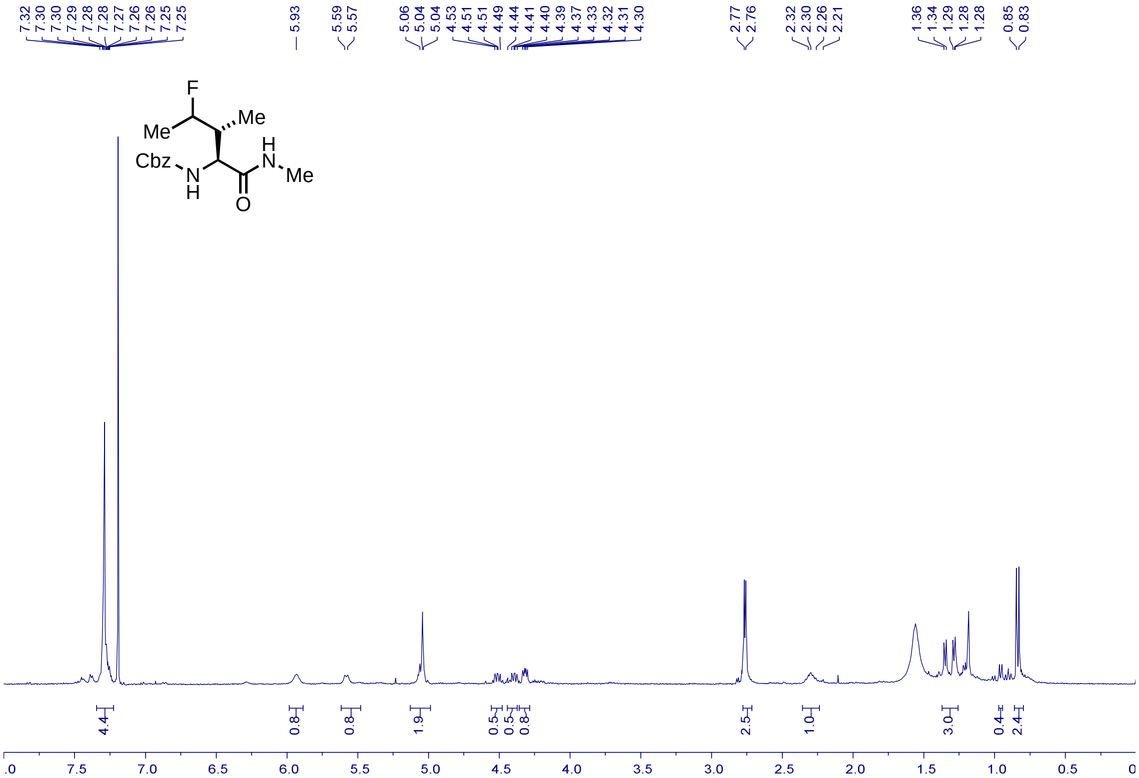

second isomer (mixture of isomers)

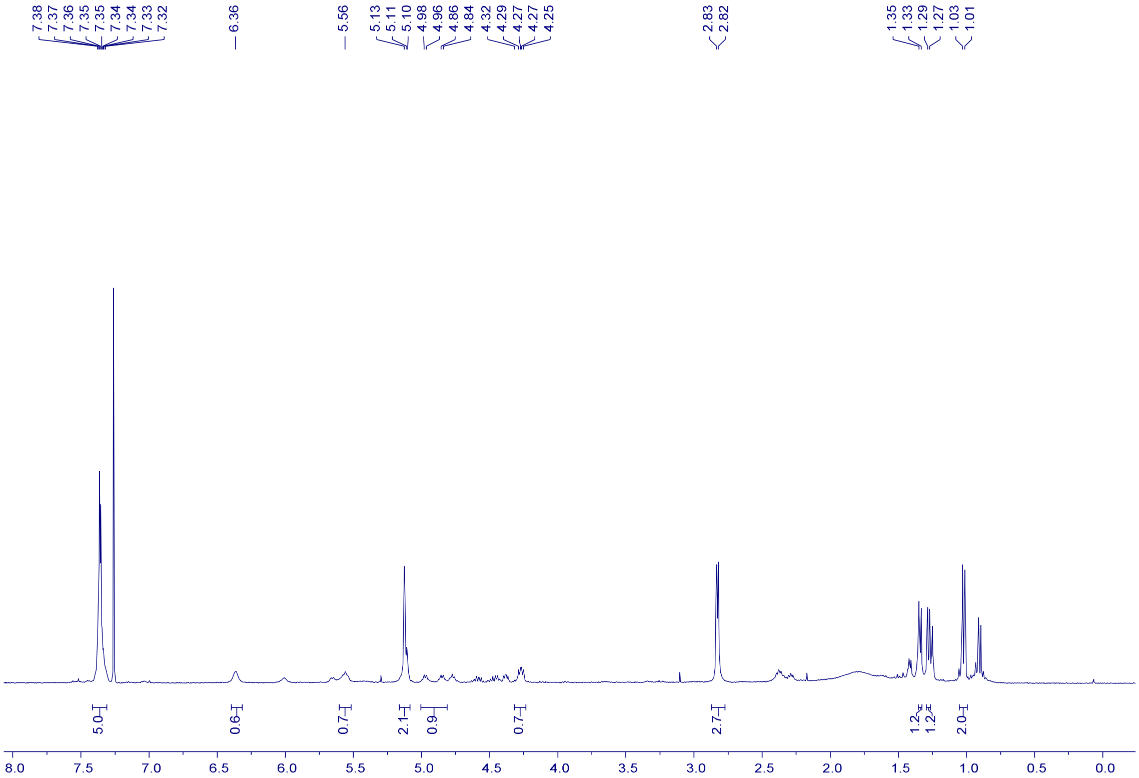

67 ^{13}C NMR (101 MHz, $CDCl_3$) first isomer

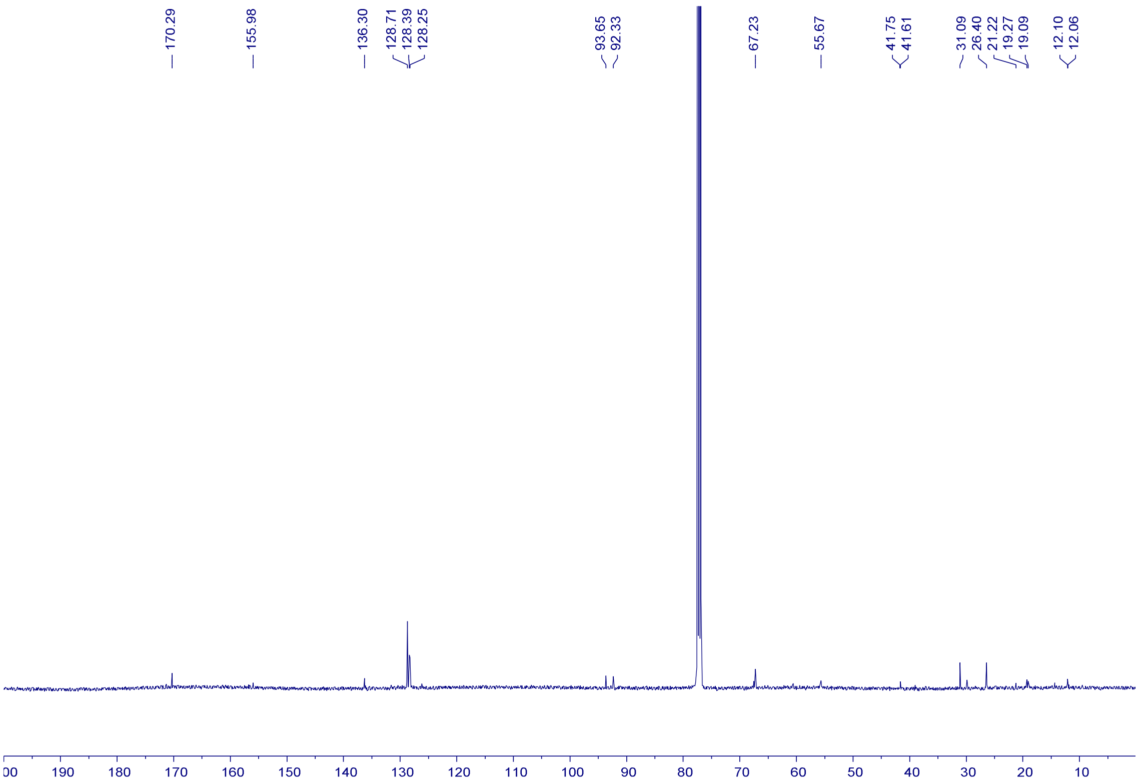

second isomer

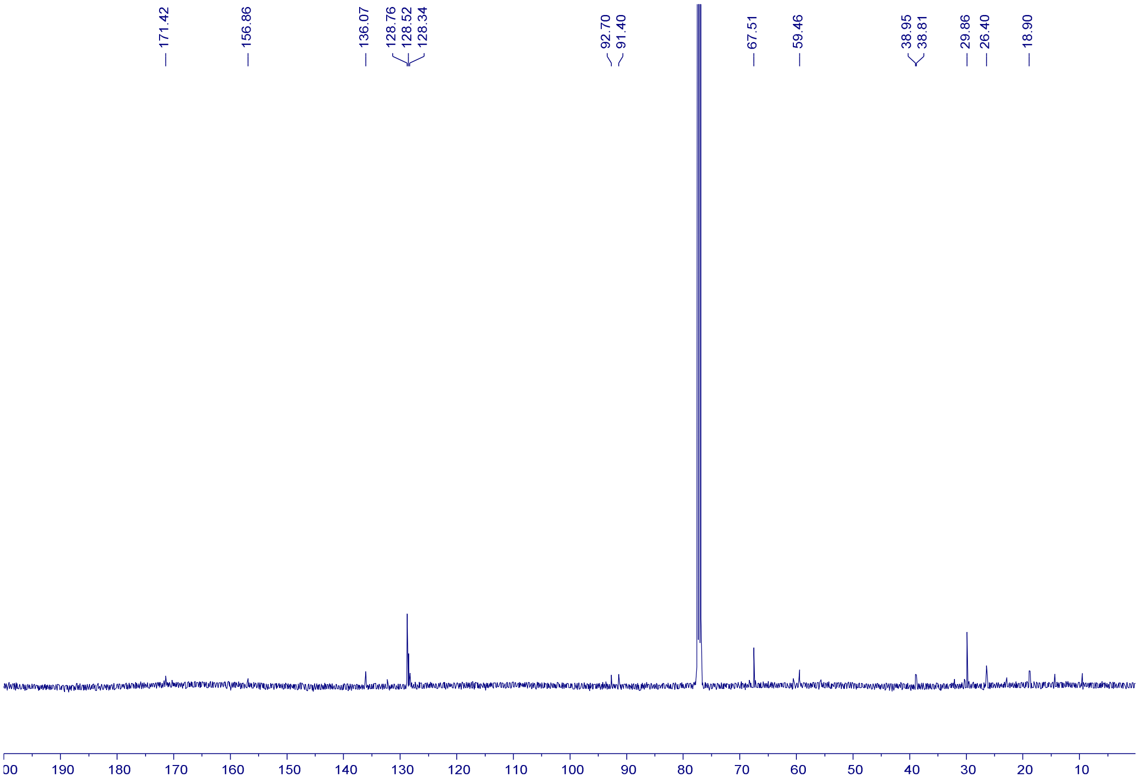

67 ^{19}F NMR (376 MHz, $CDCl_3$) first isomer

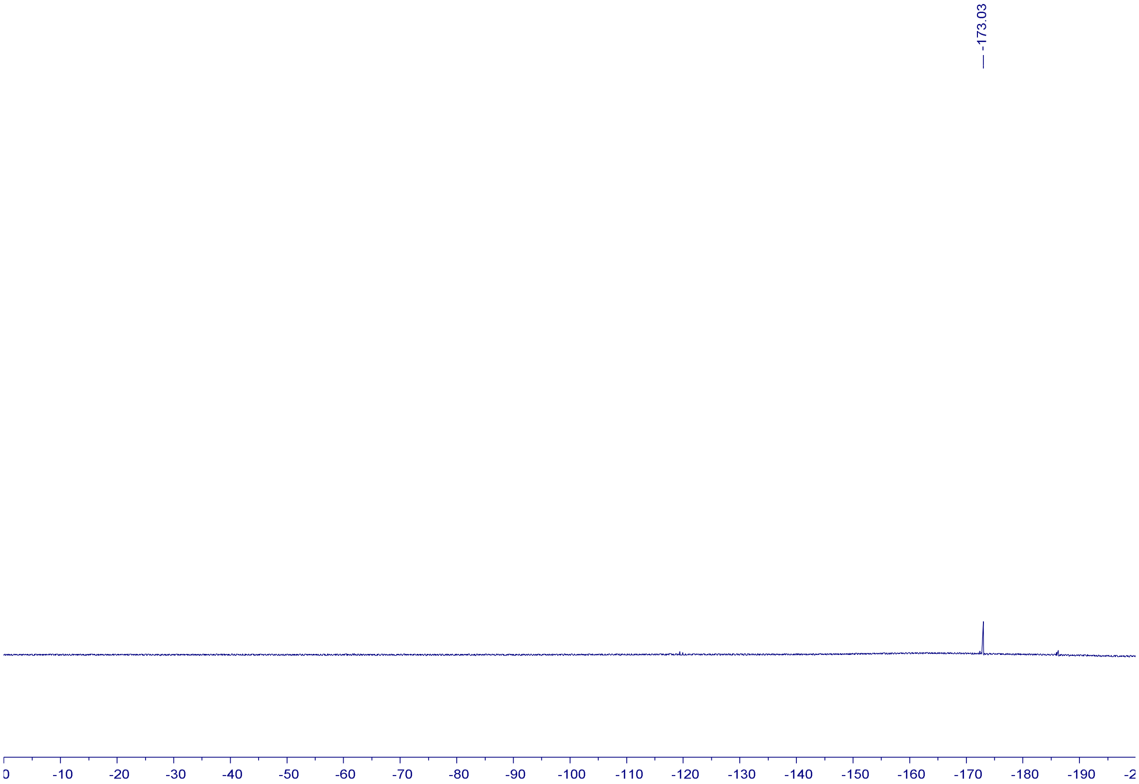

second isomer

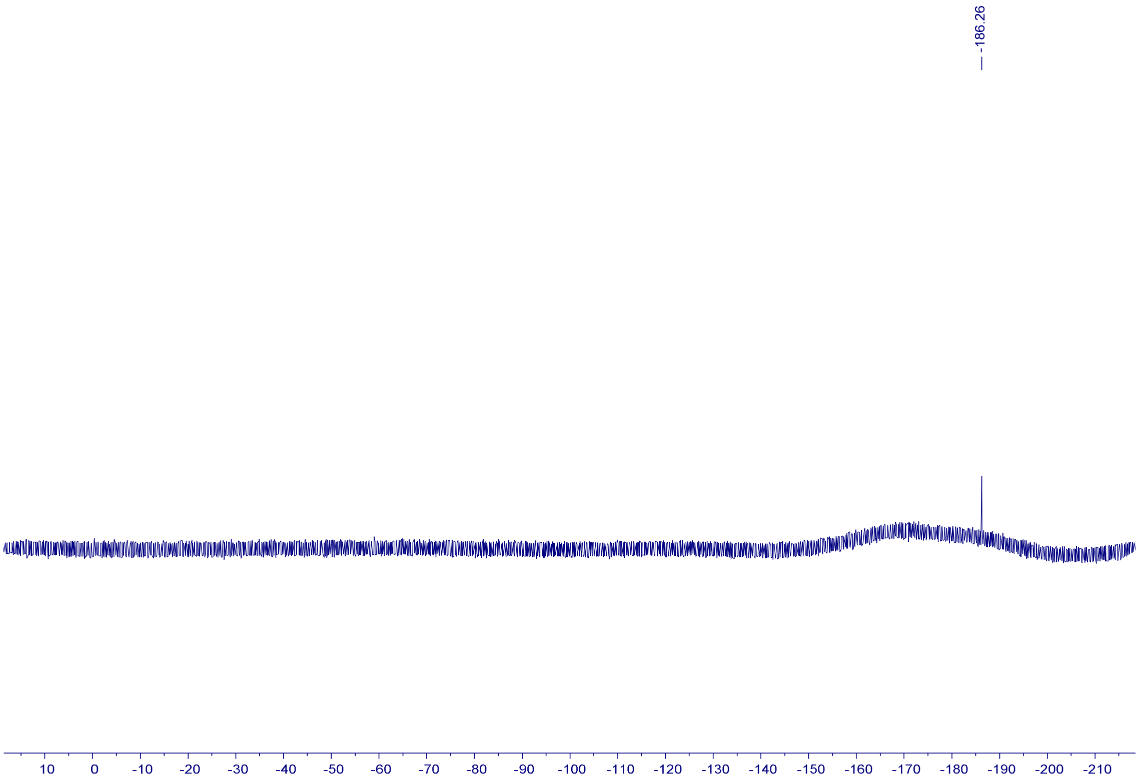

68 ^{1}H NMR (400 MHz, $CDCl_3$) first isomer

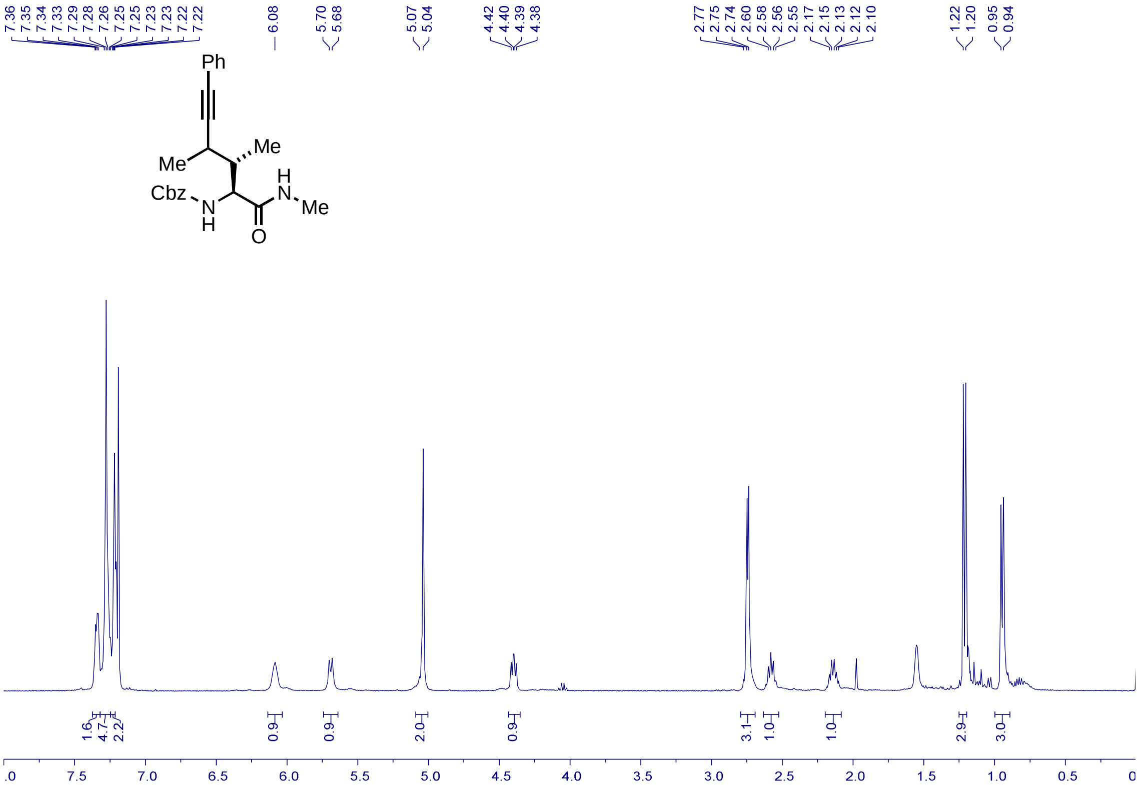

Second isomer

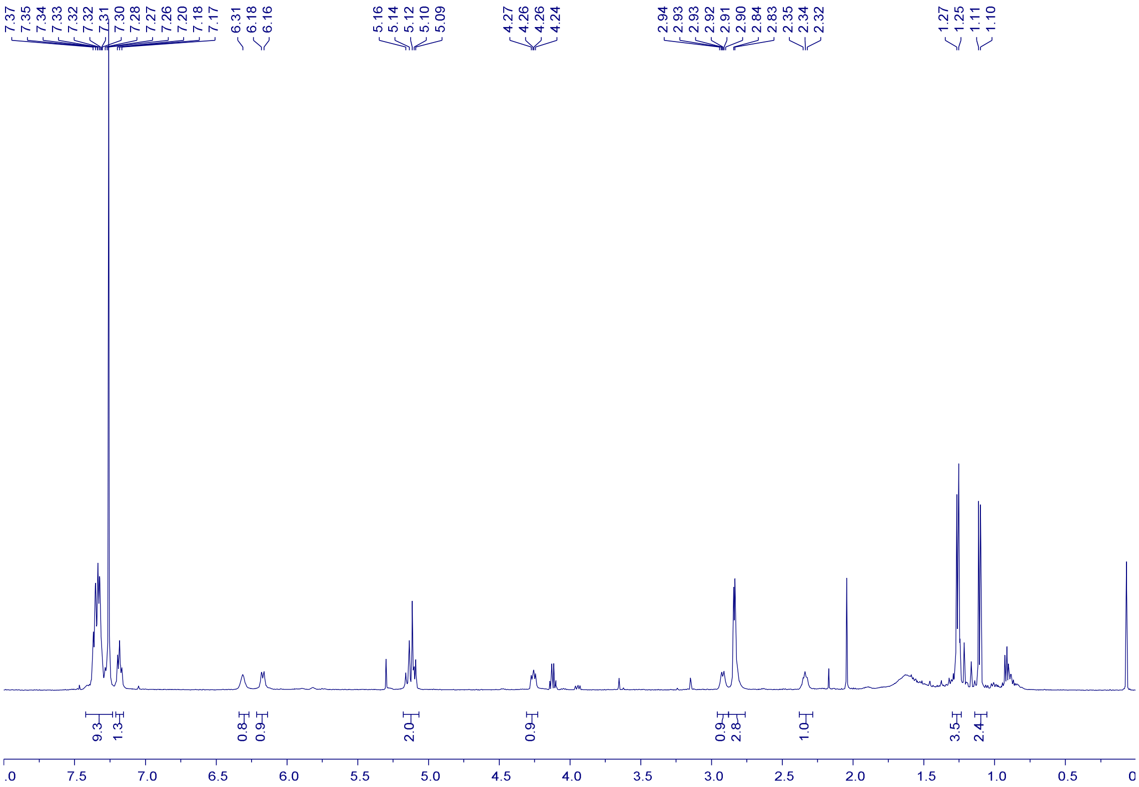

68 ^{13}C NMR (101 MHz, $CDCl_3$) first isomer

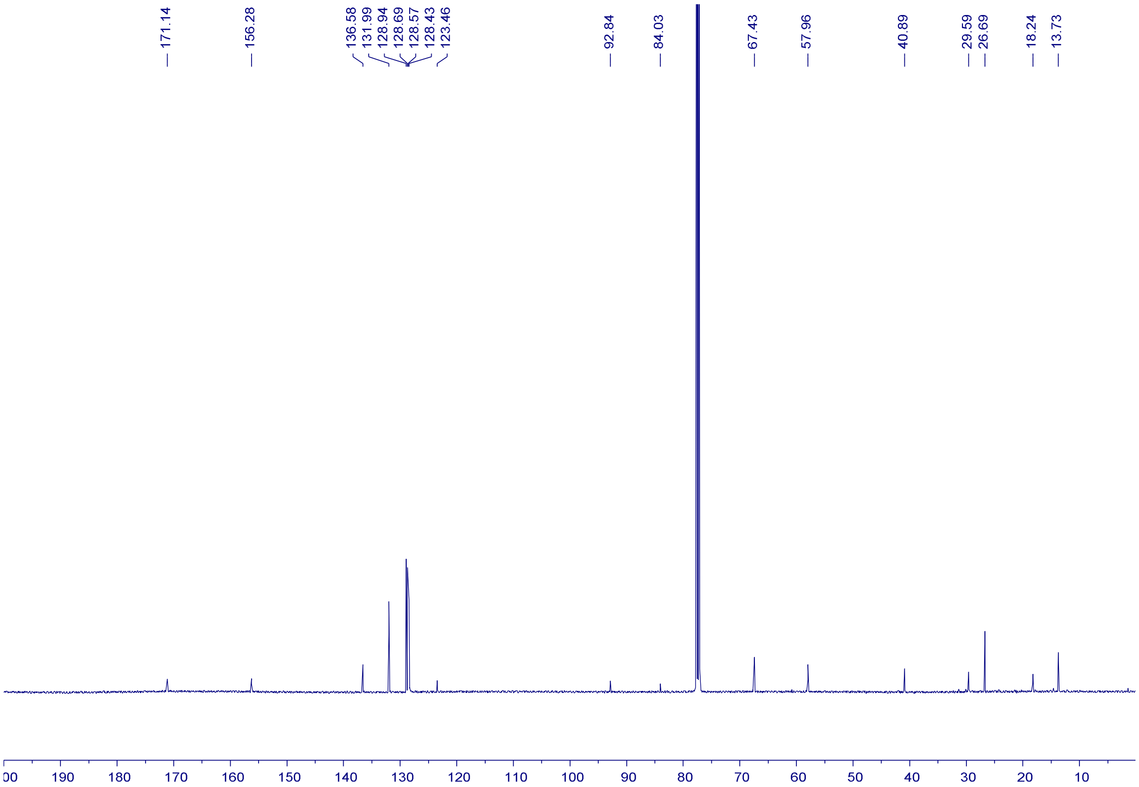

second isomer

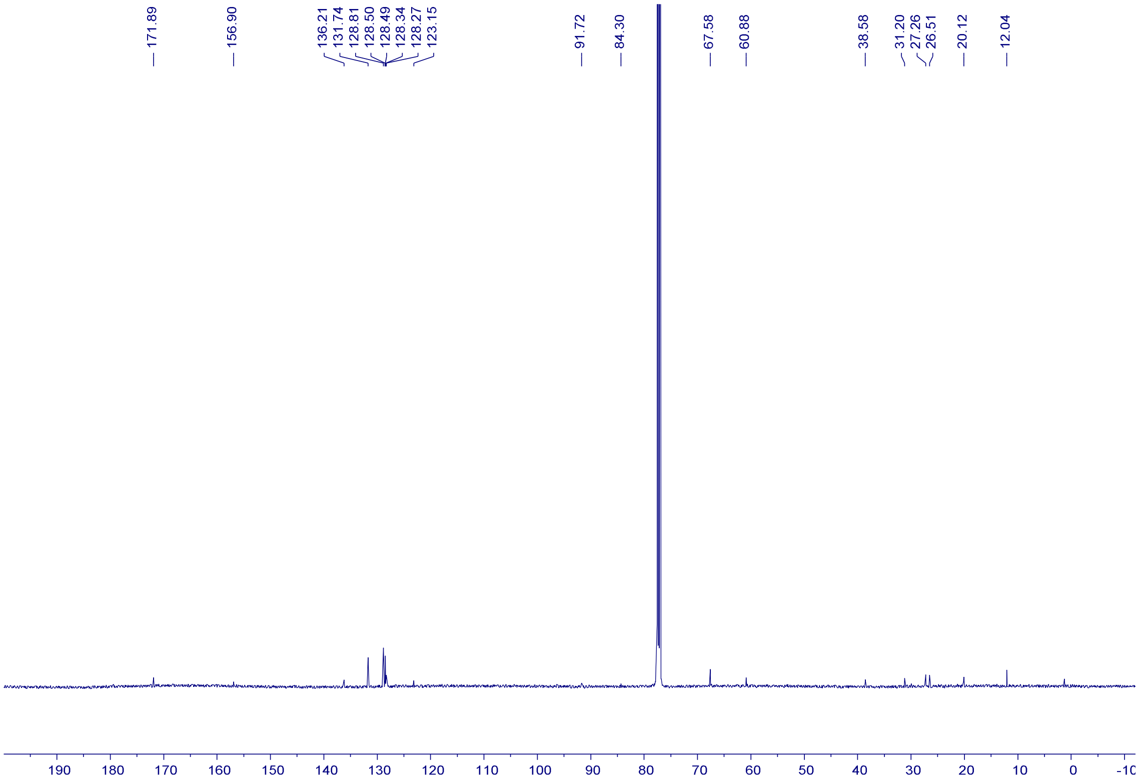

69 ^{1}H NMR (500 MHz, DMSO-d_6, 373K)

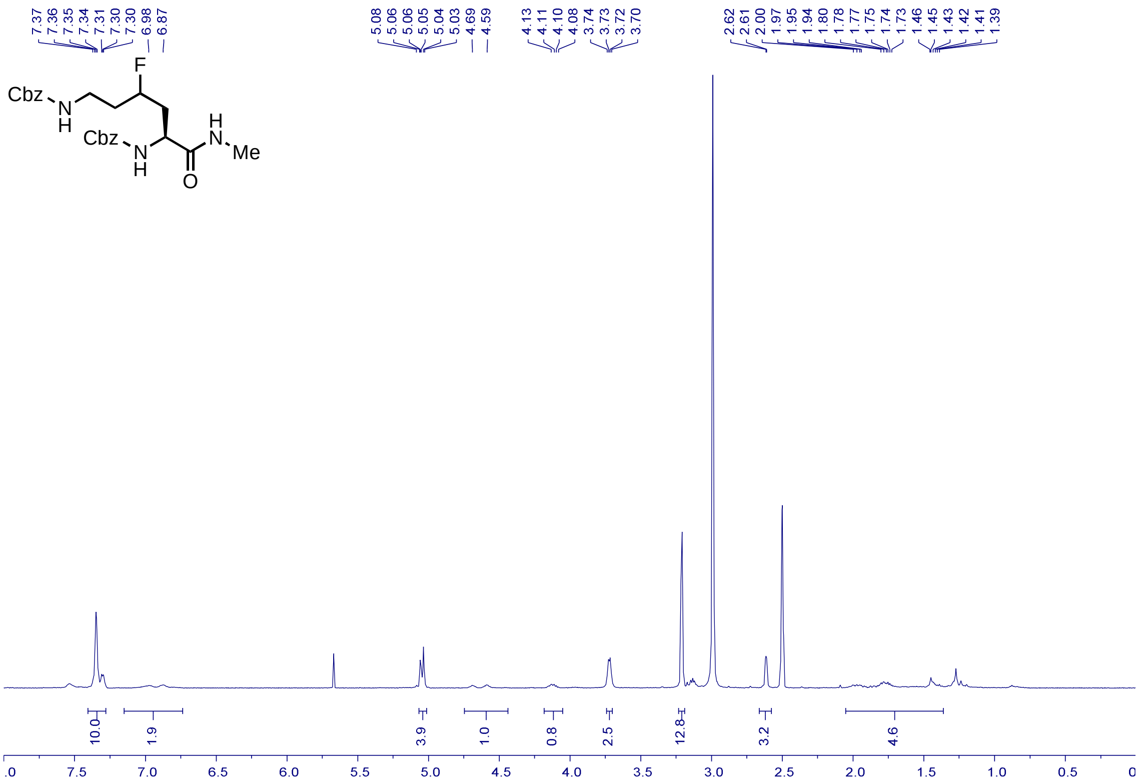

69 ^{13}C NMR (101 MHz, $CDCl_3$)

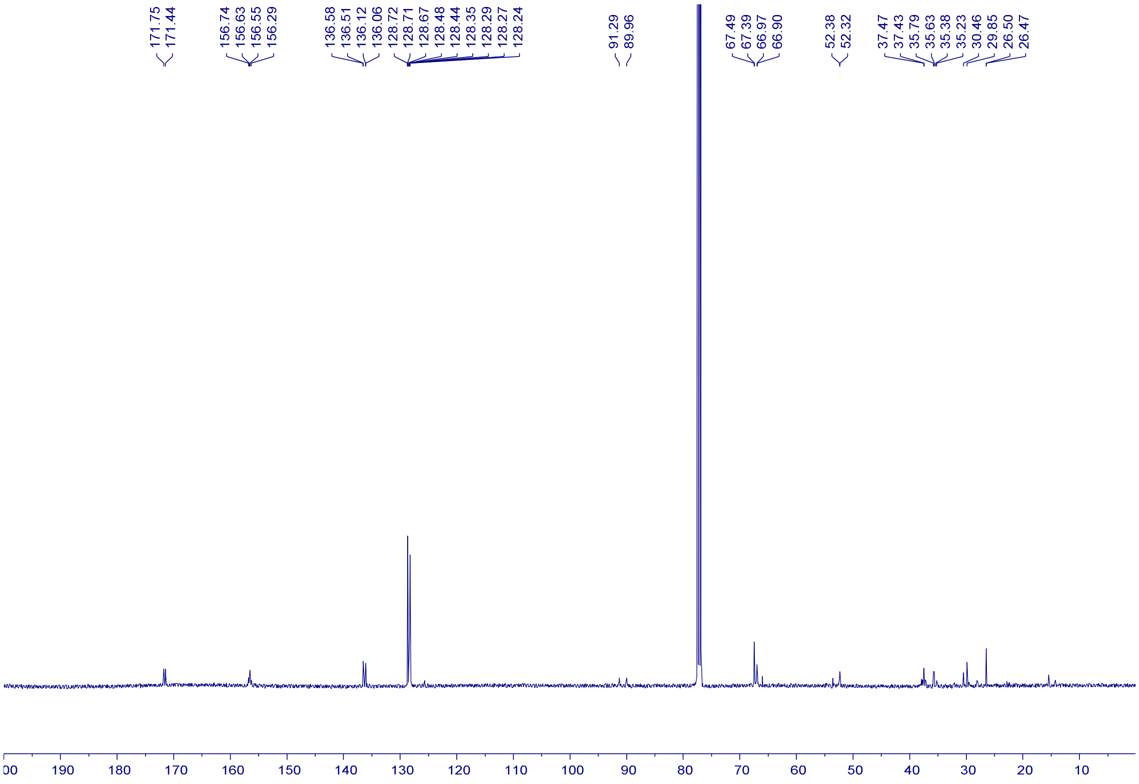

69 ^{19}F NMR (376 MHz, $CDCl_3$)

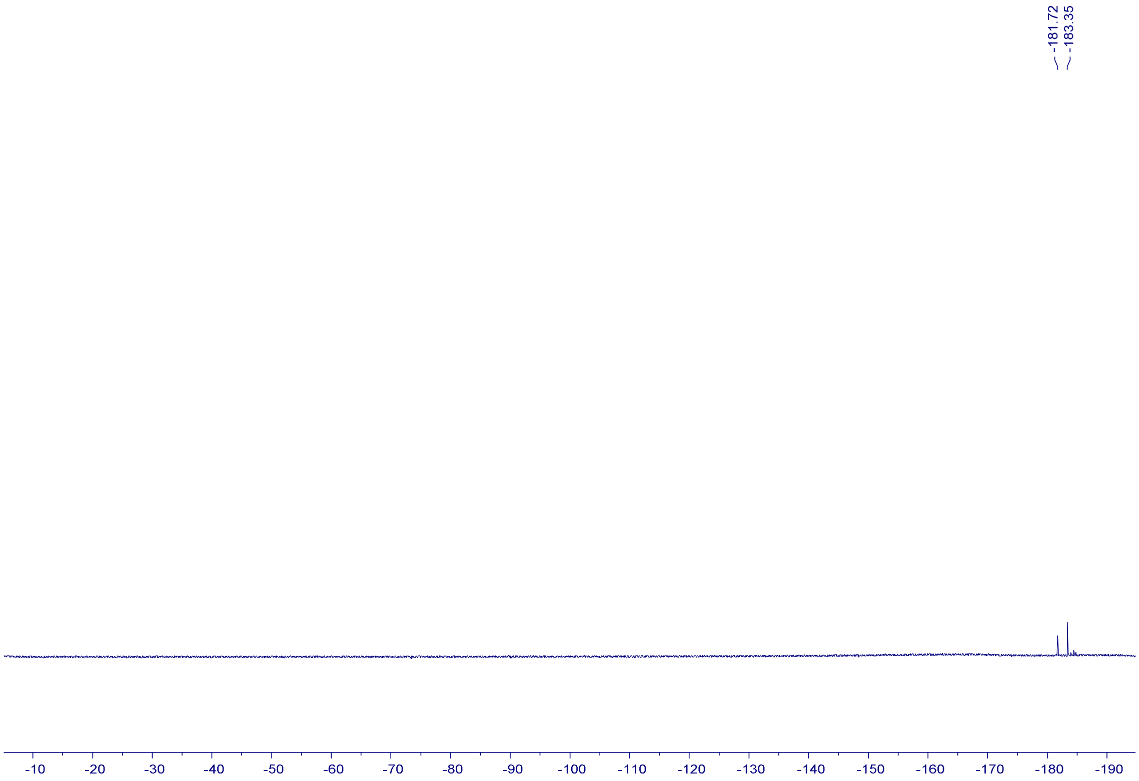

70 ^{1}H NMR (400 MHz, $CDCl_3$)

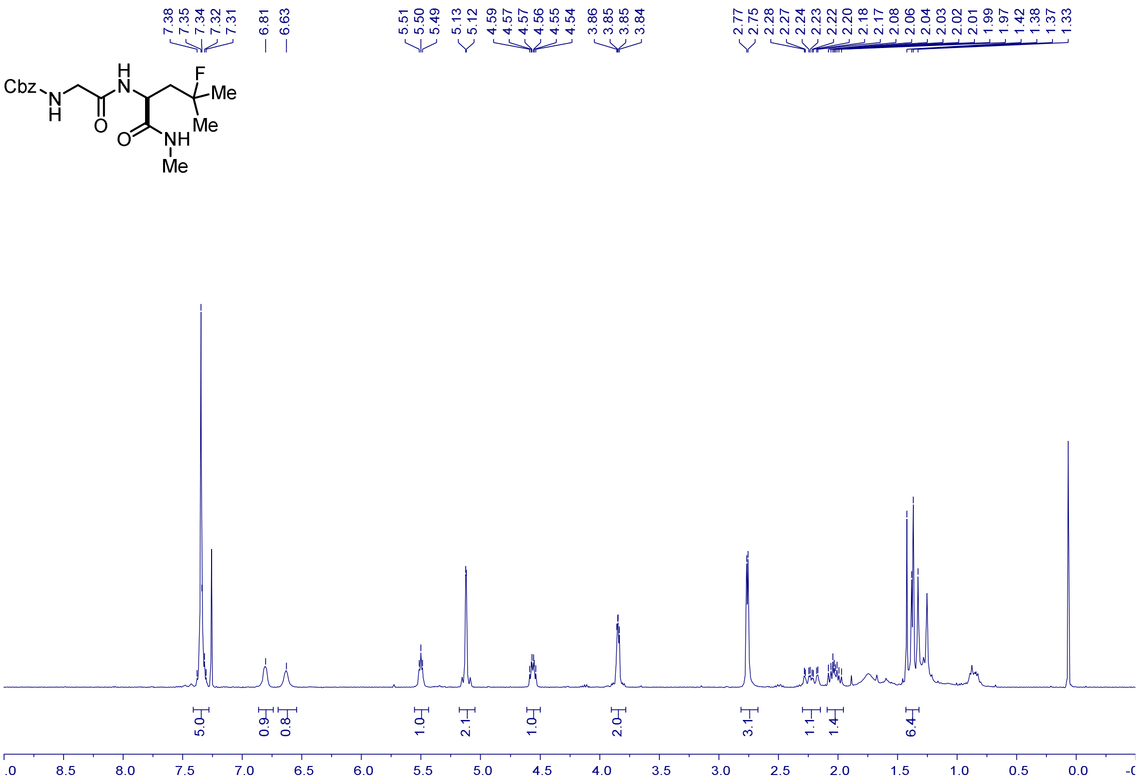

70 ^{13}C NMR (126 MHz, $CDCl_3$)

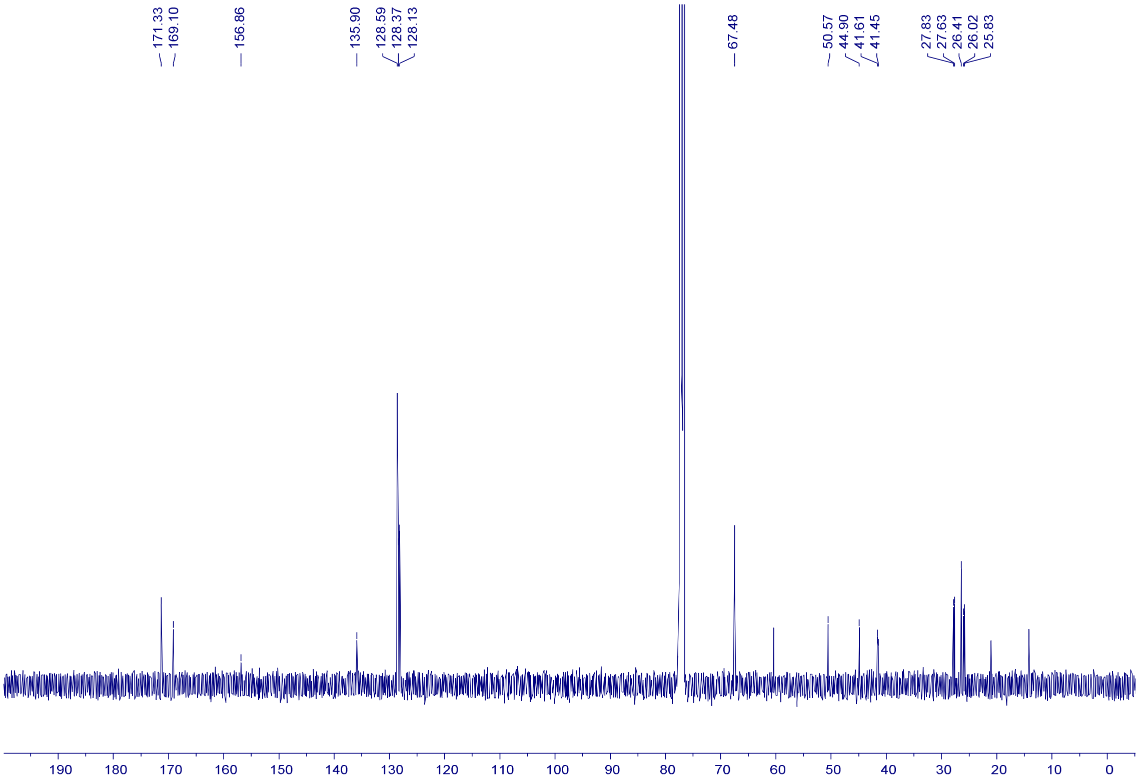

70 ^{19}F NMR (376 MHz, $CDCl_3$)

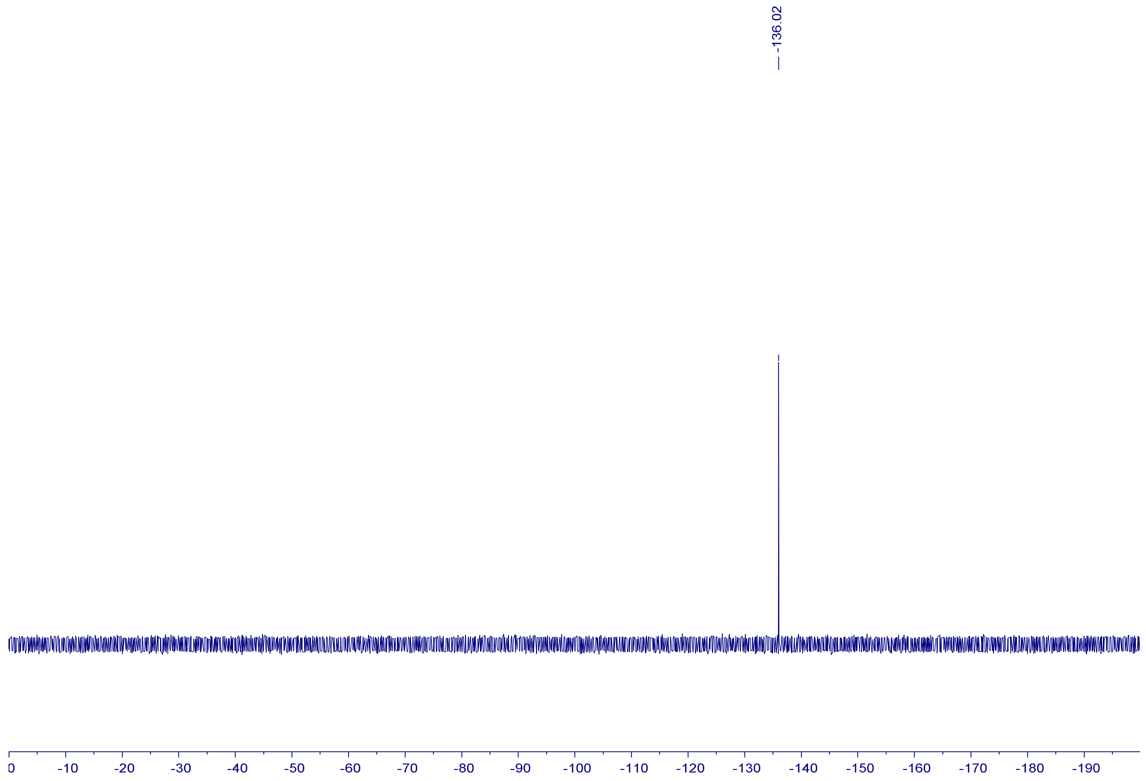

71 ^{1}H NMR (400 MHz, $CDCl_3$)

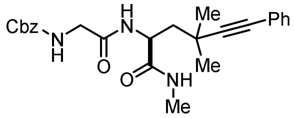

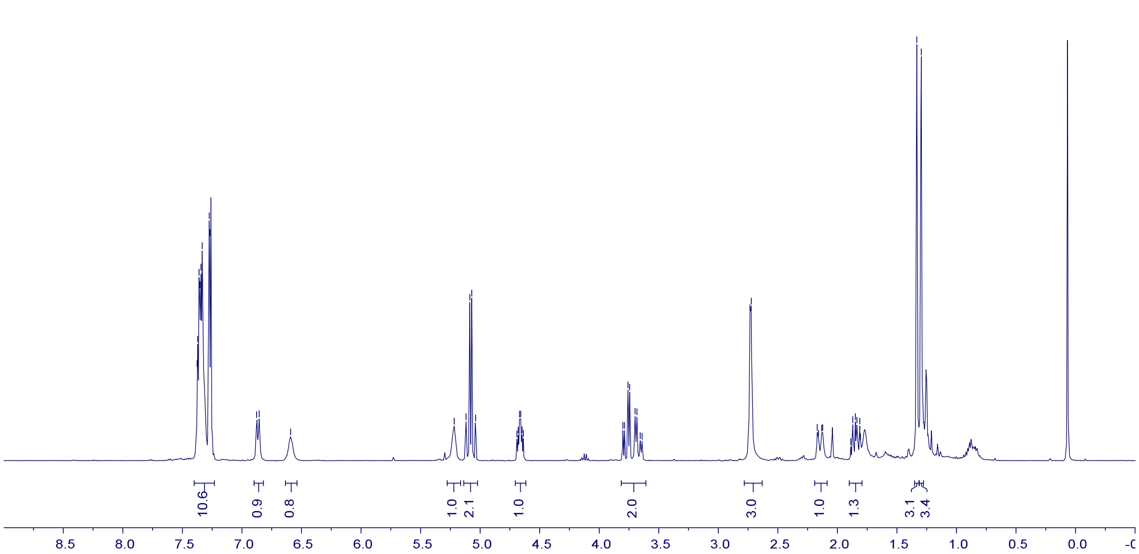

71 ^{13}C NMR (101 MHz, $CDCl_3$)

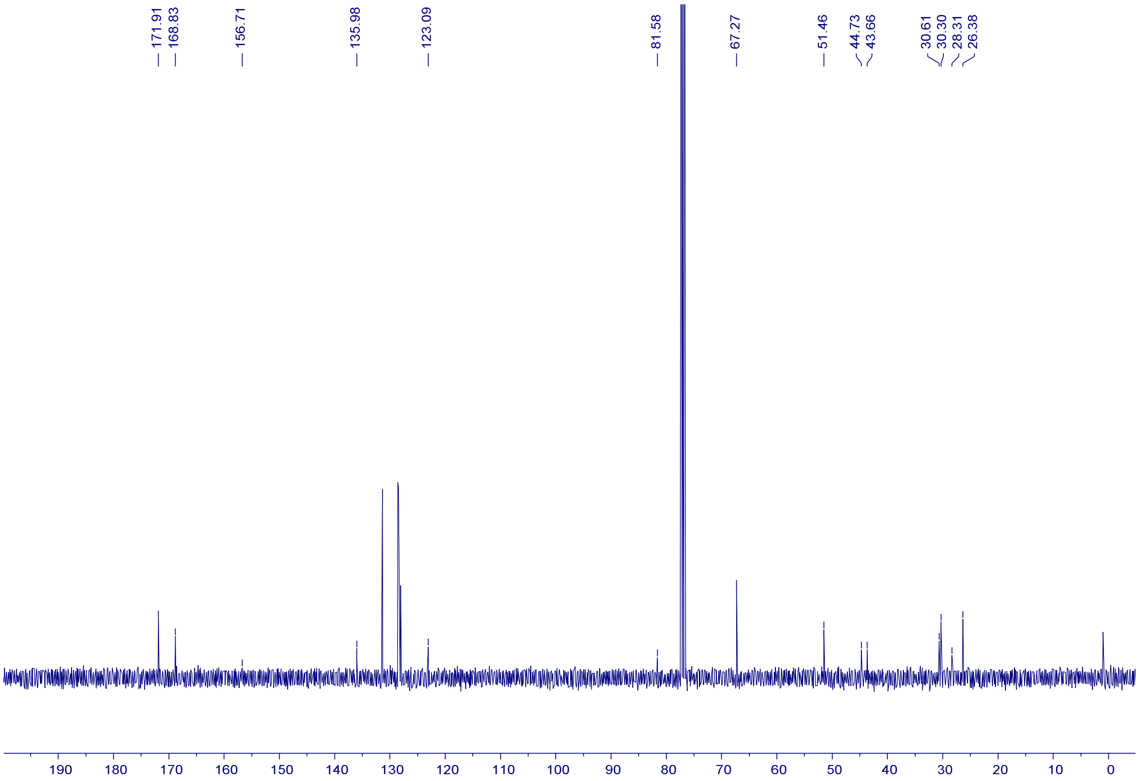

9 References

[1] X. Zhang, S. Guo, P. Tang, *Org. Chem. Front.* **2015**, *2*, 806.
[2] M. A. Cismenia, T. P. Yoon, *Chem. Sci.* **2015**, *6*, 5426.
[3] R. G. Parr, W. Yang, *Density-Functional Theory of Atoms and Molecules* **1989**, Oxford University Press, Oxford U.K.
[4] M. J. Frisch, G. W. Trucks, H. B. Schlegel, G. E. Scuseria, M. A. Robb, J. R. Cheeseman, G. Scalmani, V. BaronE, B. Mennucci, G. A. Petersson, H. Nakatsuji, M. Caricato, X. Li, H. P. Hratchian, A. F. Izmaylov, J. Bloino, G. Zheng, J. L. Sonnenberg, M. Hada, M. Ehara, K. Toyota, R. Fukuda, J. Hasegawa, M. Ishida, T. Nakajima, Y. Honda, O. Kitao, H. Nakai, T. Vreven, J. A. M. Jr., J. E. Peralta, F. Ogliaor, M. Bearpark, J. J. Heyd, E. Brothers, K. N. Kudin, V. N. taroverov, T. Keith, R. Kobayashi, J. Normand, K. Raghavachari, A. Rendell, J. C. Burant, S. S. Iyengar, J. Tomasi, M. CossI, N. Rega, J. M. Millam, M. Klene, J. E. Knox, J. B. CrossI, V. Bakken, C. Adamo, J. Jaramillo, R. Gomperts, R. E. Stratmann, O. Yazyev, A. J. Austin, R. Cammi, C. Pomelli, J. W. Ochterski, R. L. Martin, K. Morokuma, V. G. Zakrzewski, G. A. Voth, P. Salvador, J. J. Dannenberg, S. Dapprich, A. D. Daniels, O. Farkas, J. B. Foresman, J. V. Ortiz, J. Cioslowski, D. J. Fox, *Gaussian 09* **2013**, revision D.01; Gaussian, Inc.
[5] R. Denningtom, T. Keith, J. Millam, *GaussView* **2009**, version 5; Semichem Inc.: Shawnee Mission, KS, 2009.
[6] aP. J. Stephens, F. J. Devlin, C. F. Chabalowski, M. J. Frisch, *J. Chem. Phys.* **1994**, *98*, 11623; bA. D. Becke, *J. Chem. Phys.* **1993**, *98*, 1372; cA. D. Becke, *J. Phys. Chem.* **1993**, *98*, 5648; dC. Lee, W. Yang, R. G. Parr, *Phys. Rev. B* **1988**, *37*, 785.
[7] T. D. Svejstrup, W. Zawodny, J. J. Douglas, D. Bidgeli, N. S. Sheikh, D. Leonori, *Chem. Commun.* **2016**, *52*, 12302.
[8] F. D. Vleeschouwer, V. V. Speybroeck, M. Waroquier, P. Geerlings, F. D. Proft, *Org. Lett.* **2007**, *9*.
[9] F. L. Hirshfeld, *Theoret. Chim. Acta* **1977**, *44*, 129.
[10] J. Liu, S. Niwayame, Y. You, K. N. Houk, *J. Org. Chem.* **1998**, *63*, 1064.
